# CEBPA-regulated lncRNAs, new players in the study of acute myeloid leukemia

**DOI:** 10.1186/s13045-014-0069-1

**Published:** 2014-09-25

**Authors:** James M Hughes, Beatrice Salvatori, Federico M Giorgi, Irene Bozzoni, Alessandro Fatica

**Affiliations:** Department of Biology and Biotechnology C. Darwin, Sapienza University of Rome, 00185 Rome, Italy; Department of Systems Biology, Herbert Irving Comprehensive Cancer Center, Columbia University Medical Center, Columbia, New York USA; Center for Life Nano Science@Sapienza, Istituto Italiano di Tecnologia, 00161 Rome, Italy; Institute Pasteur Fondazione Cenci-Bolognetti, Sapienza University of Rome, 00185 Rome, Italy

**Keywords:** lncRNAs, CEBPA, AML

## Abstract

*CCAAT/enhancer-binding protein-α* (CEBPA) is a critical regulator of myeloid differentiation. Disruption of CEBPA function contributes to the development of acute myeloid leukemia (AML). CEBPA regulates a large number of protein coding genes of which several were shown to contribute to CEBPA function. In this study, we expand the analysis of CEBPA transcriptional targets to the newly identified class of long non-coding RNAs. We show that lncRNAs are a main component of the transcriptional program driven by C/EBPα and that many of these are also induced during granulocytic differentiation of AML cell lines supporting their relevance in proliferation arrest and differentiation.

**Electronic supplementary material:**

The online version of this article (doi:10.1186/s13045-014-0069-1) contains supplementary material, which is available to authorized users.

## To the Editor

LncRNAs participate in multiple networks controlling cell differentiation and development [1], with their expression already associated with cancer and several disorders [2]. To what degree C/EBPα regulates the expression of lncRNAs is still largely unknown.

**Figure 1:**
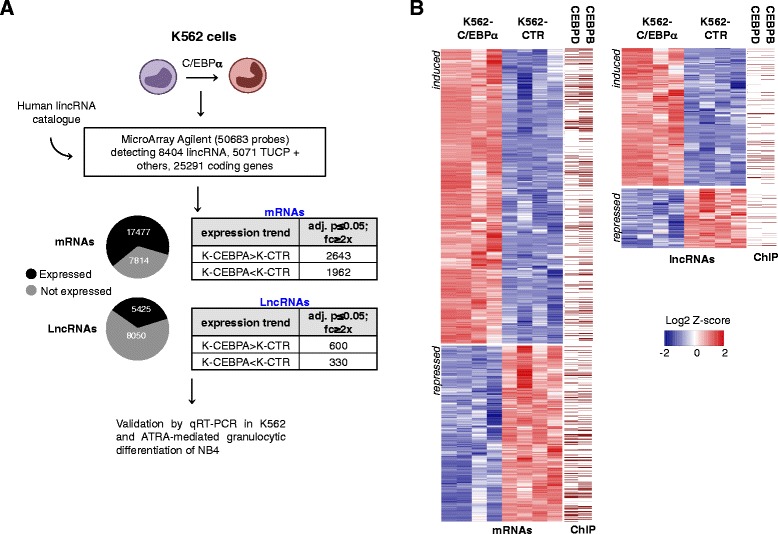
**LncRNAs are a component of the C/EBPα-regulated transcriptional network in AML. (A)** Experimental pipeline for identification of C/EBPα-regulated lncRNAs. See text and Additional file 1 for details **(B)** Heat maps of median centered log2 fold change values from microarray analysis of mRNAs and lncRNAs with significant differential expression (fold change ≥ 2 and adjusted p-value ≤ 0.05). Exact values are provided in Additional files 3 and 4: Table S1 and Table S2. Presence of CEBPB or CEBPD binding sites is indicated on the side of each heat maps.

**Figure 2:**
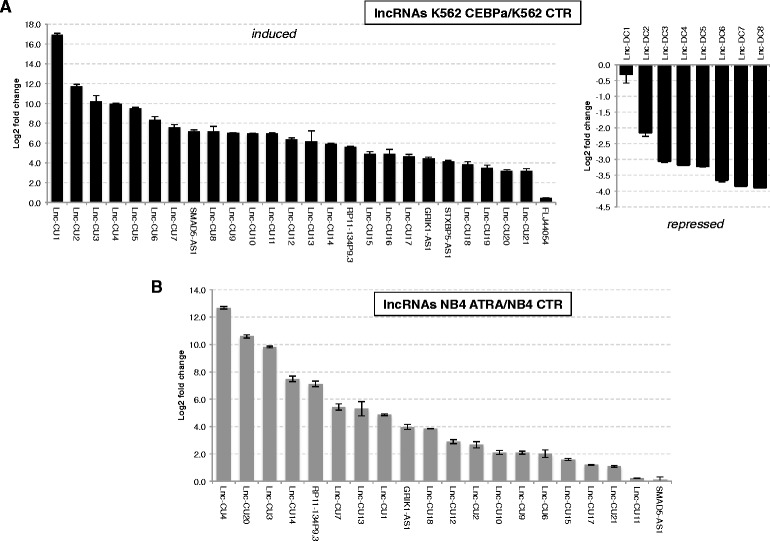
**Validation of selected lncRNAs. (A)** Quantitative real time RT-PCR analysis of selected lncRNAs in K562-C/EBPα treated for 48 hours with doxycycline. Values were compared to K562-CTR cells treated for the same time and normalized with HPRT mRNA. The histograms represent the log2 fold change of the relative expression ± SEM from three replicates. TCONS names and chromosomal positions are provided as Additional file 6: Table S3. **(B)** Quantitative real time RT-PCR analysis of selected lncRNAs in NB4 cells treated for 72 hours with all-trans-retinoic acid (ATRA). Values were compared to untreated NB4 cells and normalized with HPRT mRNA. The histograms represent the log2 fold change of the relative expression ± SEM from three replicates.

To investigate the effect of C/EBPα on the expression of lncRNAs we utilized the K562 AML cell line carrying a stable and Tet-on inducible CEBPA allele (Additional file 1 and Additional file 2: Figure S1). K562 cells lack endogenous C/EBPα and restoration of its expression induces proliferation arrest and granulocytic differentiation [3] (Additional file 1 and Additional file 2: Figure S1). Based on the expression of known C/EBPα transcriptional targets, we selected RNA extracted from 48 hours of induction (K562-C/EBPα) together with RNA extracted from control-induced cells (K562-CTR). Gene expression profiling was performed using the Agilent Whole Human Genome Oligo 8x60K v2 Microarrays from 4 biological replicates for each sample (Figure 1A). We identified 4605 mRNAs (2643 induced and 1962 repressed) and 930 lncRNAs (600 induced and 330 repressed) with significant differential expression (fold change ≥ 2 and p-value ≤ 0.05) between C/EBPα- and CTR- induced cells (Figure 1B and Additional file 1, Additional files 3 and 4: Tables S1 and S2). Appropriate expression patterns of many known coding transcriptional targets of C/EBPα confirmed the reliability of our gene expression analysis (Additional file 1 and Additional file 2: Figure S1). Gene set enrichment and Gene ontology analysis confirmed significant enrichment of known C/EBPα targets [4] (NES = 7.97, p = 1.65×10^−15^), coupled with down-regulation of cell cycle genes and upregulation of granulocytic differentiation pathways (Figure 2). Notably, we found the E2F1 motif to be negatively enriched in the promoters of C/EBPα repressed genes (NES = −7.18, p = 7.06×10^−13^), confirming the known role of C/EBPα in repressing E2F1 activity (Additional file 5: Figure S2). Expression of differentially induced lncRNAs was further validated by qRT-PCR in K562 -C/EBPα and -CTR cells (Figure 2A). When applicable, official lncRNA reference names were utilized. Otherwise, we refer to as lnc-CUs (lncRNA-C/EBPα-up-regulated) and lnc-DCs (lncRNA-C/EBPα down-regulated) for induced and repressed lncRNAs, respectively (Additional file 1 and Additional file 6: Table S3).

In order to annotate the presence of putative C/EBPα binding sites in the promoter of differentially expressed genes, we used previously generated ChIP data sets for CEBPB (C/EBPβ) and CEBPD (C/EBPδ) in K562 cells [5], which exhibit identical DNA-binding specificities with C/EBPα [6]. We found several coding and non-coding differentially expressed genes bound by either CEBPB or CEBPD in their putative promoter region within a distance of −5 kb from the TSS (Figure 1B and Additional file 1, Additional file 7: Figure S3, Additional file 8: Table S4, Additional file 9: Table S5, Additional file 10: Table S6 and Additional file 11: Table S7).

Different AML cell lines are widely used to study the block of differentiation in AML because they can be differentiated in mature and functional myeloid cells by treatment with specific agents. Thus, we analysed the expression of selected lncRNAs in NB4 cells, which are able to undergo granulocytic differentiation by treatment with *all-trans* retinoic acid (ATRA) [7]. Notably, the majority of validated C/EBPα-induced lncRNAs in K562 are also significantly upregulated by ATRA in NB4 (21 out of 26), suggesting that they may play a role in the differentiation process (Figure 2B). Nevertheless, upon validated lncRNAs repressed by C/EBPα treatment in K562, 6 out of 8 showed opposite trend while 2 were not significantly expressed in NB4 (data not shown). This behaviour still remains to be explained and extended to the study of more lncRNAs in NB4 cell line: we speculate it could be due to silencing of diverse cellular settings between K562 and NB4 cell lines.

In summary, this study shows that lncRNAs are a main component of the transcriptional program driven by C/EBPα. We identified more than 900 lncRNAs regulated by C/EBPα in K562. We confirmed that the majority of these are also induced during granulocytic differentiation of AML cell lines supporting their relevance in proliferation arrest and differentiation. How many of the lncRNAs identified in this study are directly involved in regulating differentiation programmes of AML is an interesting question that warrants further investigations.

Moreover, regardless of function, this work indicates that changes in lncRNAs expression might also have diagnostic applications in AML with CEBPA mutations.

## Additional files

Additional file 1:
**Materials and methods.**
Additional file 2: Figure S1. Effects of C/EBPα expression in K562 cells. (A) Growth curve of K562 cells containing CTR and CEBPA expression cassette, respectively, after induction with Doxycyline. As expected, cells induced with C/EBPα cease to proliferate, while the CTR empty vector cells continue to proliferate. (B) Western blot confirms the expression of endogenous C/EBPα in the CEBPA stable cell line, and not in the CTR empty vector cell line. (C) FACS analysis for the granulocytic marker CD11b shows the percentage of positive cells within the given population after 48 hours of Doxycycline induction. (D) qRT-PCR analysis of the expression of the granulocytic marker GCSFR after 48 hrs of induction. Values were normalized with HPRT mRNA. The histograms represent the fold change of the relative expression ± SEM from three replicates. (E) Known C/EBPα transcriptional targets identified in our microarray analysis. Additional file 3: Table S1. CEBPA-regulated lncRNAs with significant differential expression (absolute fold change ≥ 2 and adjusted P value ≤ 0.05) identified in K562. (A) Up-regulated lncRNAs. (B) Down-regulated lncRNAs. Additional file 4: Table S2. CEBPA-regulated mRNAs with significant differential expression (absolute fold change ≥ 2 and adjusted P value ≤ 0.05) identified in K562. (A) Up-regulated mRNAs. (B) Down-regulated mcRNAs. Additional file 5: Figure S2. GSEA on CEBPA-regulated mRNAs. The enrichment score (ES; y-axis) reflects the degree to which a gene set is overrepresented in K562 expressing CEBPA. Each solid bar represents 1 gene within a gene set. Lower panels (List values) illustrate log2 fold change for the gene set. The GSEA histograms for the gene sets CEBPA, E2F1, “granulocyte pathway” and “cell cycle” are shown with the normalized enrichment score (NES) and p-values. Additional file 6: Table S3. Chromosomal coordinates and TCONS names of validated C/EBPα -up regulated (Lnc-CUs) and -down-regulated (Lnc-DCs) lncRNAs. Additional file 7: Figure S3. Overlap between lncRNAs identified in this study used previously generated ChIP data sets for CEBPB and CEBPD in K562 cells. Additional file 8: Table S4. Intersection between CEBPB ChiP-seq data CEBPA upregulated lncRNAs. Additional file 9: Table S5. Intersection between CEBPD ChiP-seq data CEBPA upregulated lncRNAs. Additional file 10: Table S6. Intersection between CEBPB ChiP-seq data CEBPA downregulated lncRNAs. Additional file 11: Table S7. Intersection between CEBPD ChiP-seq data CEBPA downregulated lncRNAs.

## References

[1] Fatica A, Bozzoni I (2014). Long non-coding RNAs: new players in cell differentiation and development. Nat Rev Genet.

[2] Batista PJ, Chang HY (2013). Long noncoding RNAs: cellular address codes in development and disease. Cell.

[3] Tavor S, Park DJ, Gery S, Vuong PT, Gombart AF, Koeffler HP (2003). Restoration of C/EBPalpha expression in a BCR-ABL + cell line induces terminal granulocytic differentiation. J Biol Chem.

[4] Subramanian A, Tamayo P, Mootha VK, Mukherjee S, Ebert BL, Gillette MA, Paulovich A, Pomeroy SL, Golub TR, Lander ES, Mesirov JP (2005). Gene set enrichment analysis: A knowledge-based approach for interpreting genome-wide expression profiles. PNAS.

[5] Johnson DS, Mortazavi A, Myers RM, Wold B (2007). Genome-wide mapping of in vivo protein-DNA interactions. Science.

[6] Tsukada J, Yoshida Y, Kominato Y, Auron PE (2011). The CCAAT/enhancer (C/EBP) family of basic-leucine zipper (bZIP) transcription factors is a multifaceted highly-regulated system for gene regulation. Cytokine.

[7] Roussel MJ, Lanotte M (2001). Maturation sensitive and resistant t(15;17) NB4 cell lines as tools for APL physiopathology: nomenclature of cells and repertory of their known genetic alterations and phenotypes. Oncogene.

