# Supplementary material for: CEBPA-regulated lncRNAs, new players in the study of acute myeloid leukemia
Source: J Hematol Oncol. 2014 Sep 25;7:69. doi: 10.1186/s13045-014-0069-1 (PMC4177583; doi:10.1186/s13045-014-0069-1)
Supplement: Additional file 1: — Materials and methods. [file 13045_2014_69_MOESM1_ESM.doc]

**Materials and Methods**

**Cell culture and reagents**

K562 and NB4 cell lines were cultured at 37°C under an atmosphere containing 5% CO2 in RPMI 1640 medium supplemented with 1x Penicillin/Streptomicin solution, 1x L-glutamine and 10% Fetal Bovine Serum. Doxycycline (Dox) and retinoic acid (RA) were purchased from Sigma and utilized at a concentration of 200 ng/ml and 1 μM, respectively. Differentiation was assessed as previously described (1).

**Stable cell lines**

The CEBPA cDNA was amplified from pcDNA3.1-CEBPA plasmid (kindly provided by K. Nerlov) and subcloned in the enhanced PiggyBac (ePB) vector ePB-PURO (2) generating the ePB-PURO-CEBPA plasmid. This plasmid contains a TET-on system for inducible transgene expression. Helper and transposon plasmids were electroporated in K562 with the Neon Transfection System (Invitrogen) according to manufacturer instruction. Selection with 1μg/mL of puromycin (SIGMA) was initiated 2 days after transfection and maintained until resistant colonies became visible.

**RNA extraction and real-time qRT-PCR analysis**

Total RNA was extracted using TRIzol (Invitrogen) and the miRNeasy Mini Kit (Qiagen) according to manufacturer instructions. Reverse transcription to cDNA was performed with the miScript II RT Kit (Qiagen) according to the manufacturer instructions. Quantitative real-time PCR was performed using the miScript SYBR Green PCR kit (Qiagen) on an Applied Biosystems 7500 Fast Real Time PCR System. Reactions were performed in

triplicate using the SYBR green dye detection system and analyzed using 7500 Software v2.0.6 (Applied Biosystems). Relative expression levels of targets were determined using the comparative 2^∆∆Ct^ method with Invitrogen oligonucleotides probing for Hypoxanthine-guanine phosphoribosyltransferase (HPRT) mRNA levels as a reference.

**Microarray analysis**

Microarray analysis was provided by Miltenyi Biotech using the Agilent Whole Human Genome Oligo 8x60K v2, which includes reporters designed based on the catalogues of human lincRNAs (large intergenic non-coding RNAs) and TUCPs (transcripts of uncertain coding potential) developed at the Broad institute (9) (for details and specifications see http://www.genomics.agilent.com). For the linear T7-based amplification step, 100 ng of each total RNA sample was used. To produce Cy3-labeled cRNA, the RNA samples were amplified and labeled using the Agilent Low Input Quick Amp Labeling Kit (Agilent Technologies) following the manufacturer’s protocol. The hybridization procedure was performed according to the Agilent 60-mer oligo microarray processing protocol using the Agilent Gene Expression Hybridization Kit (Agilent Technologies). Briefly, 600 ng Cy3-labeled fragmented cRNA in hybridization buffer was hybridized overnight (17 hours, 65 °C) to Agilent W hole Human Genome Oligo Microarrays 8x60K V2 using Agilent’s recommended hybridization chamber and oven. Fluorescence signals of the hybridized Agilent Microarrays were detected using Agilent’s Microarray Scanner System (Agilent Technologies). The statistical tests were complemented by a non-statistical quantification of the mean expression difference between the two groups. A fold-change of +/- 2x was chosen for the selection of candidate reporters. In order to identify gens with significant differential expression, Student' t-tests (two-tailed, equal variance) were conducted on the pairwise group combinations of interest. In this test, a p-value is assigned to each Agilent reporter, which indicates whether there is a significant differential expression between the respective cell types or treatment conditions. A correction for multiple testing of the t-test p-values was conducted using the method of Benjamini and Hochberg. Statistically significant changes in expression are considered for genes with adjusted p-values of less than or equal to 0.05.

**Data access**

The microarray data have been deposited in NCBI's Gene Expression Omnibus (GEO) and are accessible through GEO Series accession number GSE58743.

**Gene Set Enrichment Analysis (GSEA)**

GSEA was performed with the “curated” gene sets available at GSEA website (<http://www.broad.mit.edu/gsea/>) (10). Significant gene sets were selected on the basis with nominal *P* value less than .05.

**ChIP-seq analysis for CEBPs binding sites**

Raw data from CEBPB and CEBPD ChIP-seq experiments in K562 were retrieved from the Encode Project Consortium and used to determine the distances between the transcription factor binding sites and the location of the nearest differentially expressed mRNA or lncRNA probes. Peak calling was performed using MACS version 2.0.6. The peak summit was used as the location parameter for distance computations. The peak intensity was calculated as the maximum pileup height extracted from BIGWIG file or pileup height at peak summit as returned by MACS. The number of CEBPA induced or repressed mRNA and lncRNA transcripts present on the microarray with a CEBPB or CEBPD binding site within a distance of -5 kb from the TSS was calculated.

**Primer List**

| **Transcript ID** | **Forward primer** | **Reverse primer** |
| --- | --- | --- |
| TCONS_00022787 | GGAAGCTGGAGAACTCTCAATAC | CTAAAGGGCAAAGCGCAAAC |
| TCONS_I2_00010170 | GCACAGAAGAGACCAGAGAATAG | TTGCAGAAGAGGGCACTG |
| TCONS_l2_00013854 | CCAGAAGGTGAGAGCCTAATAC | CCCATTCCTTGAGACGGTAA |
| TCONS_00023281 | GTCTGTAGCCCAAGCAGGAG | GACTTGGAGAGCTCGGTCAC |
| TCONS_00017790 | GCCCCAGAGAGATATGTCCA | ACAGGACGTGTTTCCCAAAG |
| TCONS_00011079 | CAGTGTGCAACCTTTGCAGT | GTGATCCCTGCTTTGTGGAT |
| TCONS_l2_00026920 | CTCGGACGTAGTTCACAGCA | TGGTGGACTTTACCGTGACA |
| TCONS_00022790 | GGAACCAAGGCCTTAGGAAG | CCACGCTGGAACAAGGTACT |
| TCONS_00000489 | AAATGCCATCTGAGGACCAG | CAGATGCCAACACAGGAAGA |
| TCONS_00021009 | AAACGCAAAGCTTCAAAAGG | AAGGAAATGCCTGTGCTGTT |
| TCONS_00012257 | ATTGGCTCCATCTCAAACCA | TCTCACCAGGTTCACACAGC |
| TCONS_00009724 | TACCATGGGCTACATGCTGA | AGCTTGCAGTGAACCGAGAT |
| STXBP5-AS1 | TTGGCGGAATACTCTGCTCT | GGGTCTACTTTCTGCCACCA |
| TCONS_00014726 | AAACAGCTGTCTGAACAAGTGG | CAGTGTGAGCCTCCTCAAGA |
| TCONS_00011670 | CTCCACCTGCTGTGTACTAATG | GGTTGTGGTGAGCCAAGAT |
| TCONS_l2_00024305 | CTGCGGGCTGATGAATTAGA | AGTGAGGTATGGCGTGAATG |
| TCONS_l2_00007295 | CAAGAAACAGGAGTGGAGAGAG | GCCATCTCAGAGTGACAACA |
| TCONS_00001757 | GAAAGCTCGCTCTCAGAAGAA | TCAGCCTAAGAGAAGACTCCA |
| TCONS_00006739 | CAGAAACATCTCTCCCGTCTTT | ATGTACGCAGGTGACGATTG |
| TCONS_00006729 | AGTACCTGGACCAAGAGAAGA | AAGTACCATGCTGTGGGAAG |
| TCONS_00001200 | GTTGGGACGGAGCATAACA | TAAACCAGTGCCAGCTACAG |
| LOC100506775 | CCAGAGCAACCTCGAAGAAA | GACACCACATGGAGAGTTAGTC |
| LINC00452 | AGCCTAGAGTGAGTGTTCCT | TATTGGCTCAGCTCGAACAC |
| SMAD5-AS1 | GGCAGAAACCAGGTCTAACTAC | GCGCTTATCGGTGGGAATTA |
| GRIK1-AS1 | TGTCTCAGAGCTATGGAGGAT | TCTGTGTCCTGGTTGTTTCTC |
| NR_027105.2 | ACTGCAAGGAGCTTGGAAAA | CTCCACACTCCCATCAGGTT |
| TCONS_00029080 | CAGCCACGACAGAGGATACA | CAAGACACAAAGCCCCTCTC |
| TCONS_00023505 | CCTAGGAGTTCTCCAGAGGAT | CCTTATCAGAGCAGACCACATT |
| TCONS_l2_00013005 | TCCCAGGAGCACCATAGAAA | GCAGATCTGAGGGAGGACTAAA |
| TCONS_00029079 | GACAGAAGGGAAGGAGAGAAAG | GTCTGGATTCAGTCTCACAGAG |
| TCONS_00028879 | ACTCCAGGCACTTGTTTGT | CCAGAGGGTAATTCTCCATTCC |
| TCONS_00013572 | GCCTCCTGAGTAGCTGAGATT | GATTGCTTGAGGCTAGGAGTTC |
| TCONS_00000171 | ACTGCGCCCAAGGATTT | GACACCAGAGTTCAGACCAAG |
| TCONS_00007503 | TGATGAGCCACAGGCATTT | GCTGACCAGACTTGTCTCTTT |
| GCSFR | AGCAACAAGACCTGGAGGATGGAA | AGAGCTGAAAGGGCCTGATGTTCT |
| HPRT | Qiagen QuantiTect Primer Assay catalogue number QT00059066 | |

**Immunoblot Analysis**

40 μg of whole cell extract was separated by 10% SDS-PAGE and electroblotted to nitrocellulose membrane (Protran, S&S). Immunoblots were incubated with antibodies to C/EBPα (sc-61, Santa Cruz Biotechnology) and GAPDH (sc-25778; Santa Cruz Biotechnology).

**Titles to tables**

**Supplementary Table S1.** CEBPA-regulated lncRNAs with significant differential expression (absolute fold change ≥ 2 and adjusted P value ≤ 0.05) identified in K562. (A) Up-regulated lncRNAs. (B) Down-regulated lncRNAs.

**Supplementary Table S2.** CEBPA-regulated mRNAs with significant differential expression (absolute fold change ≥ 2 and adjusted P value ≤ 0.05) identified in K562. (A) Up-regulated mRNAs. (B) Down-regulated mcRNAs.

**Supplementary Table S3.** Chromosomal coordinates and TCONS names of validated C/EBPα -up regulated (Lnc-CUs) and -down-regulated (Lnc-DCs) lncRNAs.

**Supplementary Table S4.** Intersection between CEBPB ChiP-seq data CEBPA upregulated lncRNAs.

**Supplementary Table S5.** Intersection between CEBPD ChiP-seq data CEBPA upregulated lncRNAs.

**Supplementary Table S6.** Intersection between CEBPB ChiP-seq data CEBPA downregulated lncRNAs.

**Supplementary Table S7.** Intersection between CEBPD ChiP-seq data CEBPA downregulated lncRNAs.

**Titles and legends to figures**

**Supplementary Figure S1**. Effects of C/EBPα expression in K562 cells. (A) Growth curve of K562 cells containing CTR and CEBPA expression cassette, respectively, after induction with Doxycyline. As expected, cells induced with C/EBPα cease to proliferate, while the CTR empty vector cells continue to proliferate. (B) Western blot confirms the expression of endogenous C/EBPα in the CEBPA stable cell line, and not in the CTR empty vector cell line. (C) FACS analysis for the granulocytic marker CD11b shows the percentage of positive cells within the given population after 48 hours of Doxycycline induction. (D) qRT-PCR analysis of the expression of the granulocytic marker GCSFR after 48 hrs of induction. Values were normalized with HPRT mRNA. The histograms represent the fold change of the relative expression ± SEM from three replicates. (E) Known C/EBPα transcriptional targets identified in our microarray analysis.

**Supplementary Figure 2.** GSEA on CEBPA-regulated mRNAs. The enrichment score (ES; y-axis) reflects the degree to which a gene set is overrepresented in K562 expressing CEBPA. Each solid bar represents 1 gene within a gene set. Lower panels (List values) illustrate log2 fold change for the gene set. The GSEA histograms for the gene sets CEBPA, E2F1, "granulocyte pathway" and "cell cycle" are shown with the normalized enrichment score (NES) and p-values.

**Supplementary Figure S3**. Overlap between lncRNAs identified in this study used previously generated ChIP data sets for CEBPB and CEBPD in K562 cells.

**References**

1. Salvatori B, Iosue I, Djodji Damas N, Mangiavacchi A, Chiaretti S, Messina M, Padula F, Guarini A, Bozzoni I, Fazi F, Fatica A: **Critical Role of c-Myc in Acute Myeloid Leukemia Involving Direct Regulation of miR-26a and Histone Methyltransferase EZH2.** *Genes Cancer* 2011; **2**:585-592.
2. Salvatori B, Iosue I, Mangiavacchi A, Loddo G, Padula F, Chiaretti S, Peragine N, Bozzoni I, Fazi F, Fatica A: **The microRNA-26a target E2F7 sustains cell proliferation and inhibits monocytic differentiation of acute myeloid leukemia cells.** *Cell Death Dis* 2012; **3**:e413.
3. Zhang P, Nelson E, Radomska HS, Iwasaki-Arai J, Akashi K, Friedman AD, Tenen DG: **Induction of granulocytic differentiation by 2 pathways.** *Blood* 2002; **99**:4406-4412.
4. Zhang P, Iwama A, Datta MW, Darlington GJ, Link DC, Tenen DG. **Upregulation of interleukin 6 and granulocyte colony-stimulating factor receptors by transcription factor CCAAT enhancer binding protein alpha (C/EBP alpha) is critical for granulopoiesis.** *J Exp Med* 1998; **188**:1173-1184.
5. Zhang DE, Hetherington CJ, Meyers S, Rhoades KL, Larson CJ, Chen HM, Hiebert
6. SW, Tenen D: **CCAAT enhancer-binding protein (C/EBP) and AML1 (CBF alpha2) synergistically activate the macrophage colony-stimulating factor receptor promoter.** *Mol Cell Biol* 1996; **16**:1231-1240.
7. Smith LT, Hohaus S, Gonzalez DA, Dziennis SE, Tenen DG: **PU.1 (Spi-1) and C/EBPalpha regulate the granulocyte colony-stimulating factor receptor promoter in myeloid cells.** *Blood* 1996; **88**:1234-1247.
8. Federzoni EA, Humbert M, Torbett BE, Behre G, Fey MF, Tschan MP: **CEBPA-dependent HK3 and KLF5 expression in primary AML and during AML differentiation.** *Sci Rep* 2014; **4**:4261.
9. Lidonnici MR, Audia A, Soliera AR, Prisco M, Ferrari-Amorotti G, Waldron T, Donato N, Zhang Y, Martinez RV, Holyoake TL, Calabretta B: **Expression of the transcriptional repressor Gfi-1 is regulated by C/EBP{alpha} and is involved in its proliferation and colony formation-inhibitory effects in p210BCR/ABL-expressing cells.** *Cancer Res* 2010; **70**:7949-7959.
10. Cabili MN, Trapnell C, Goff L, Koziol M, Tazon-Vega B, Regev A, Rinn JL: **Integrative annotation of human large intergenic noncoding RNAs reveals global properties and specific subclasses.** *Genes Dev* 2011; **25**:1915-1927.
11. Subramanian A, Tamayo P, Mootha VK, Mukherjee S, Ebert BL, Gillette MA, Paulovich A, Pomeroy SL, Golub TR, Lander ES, Mesirov JP: **Gene set enrichment analysis: a knowledge-based approach for interpreting genome-wide expression profiles.** *Proc Natl Acad Sci U S A* 2005; **102**:15545-15550.
