# Supplementary figures and images for: CEBPA-regulated lncRNAs, new players in the study of acute myeloid leukemia
Source: J Hematol Oncol. 2014 Sep 25;7:69. doi: 10.1186/s13045-014-0069-1 (PMC4177583; doi:10.1186/s13045-014-0069-1)

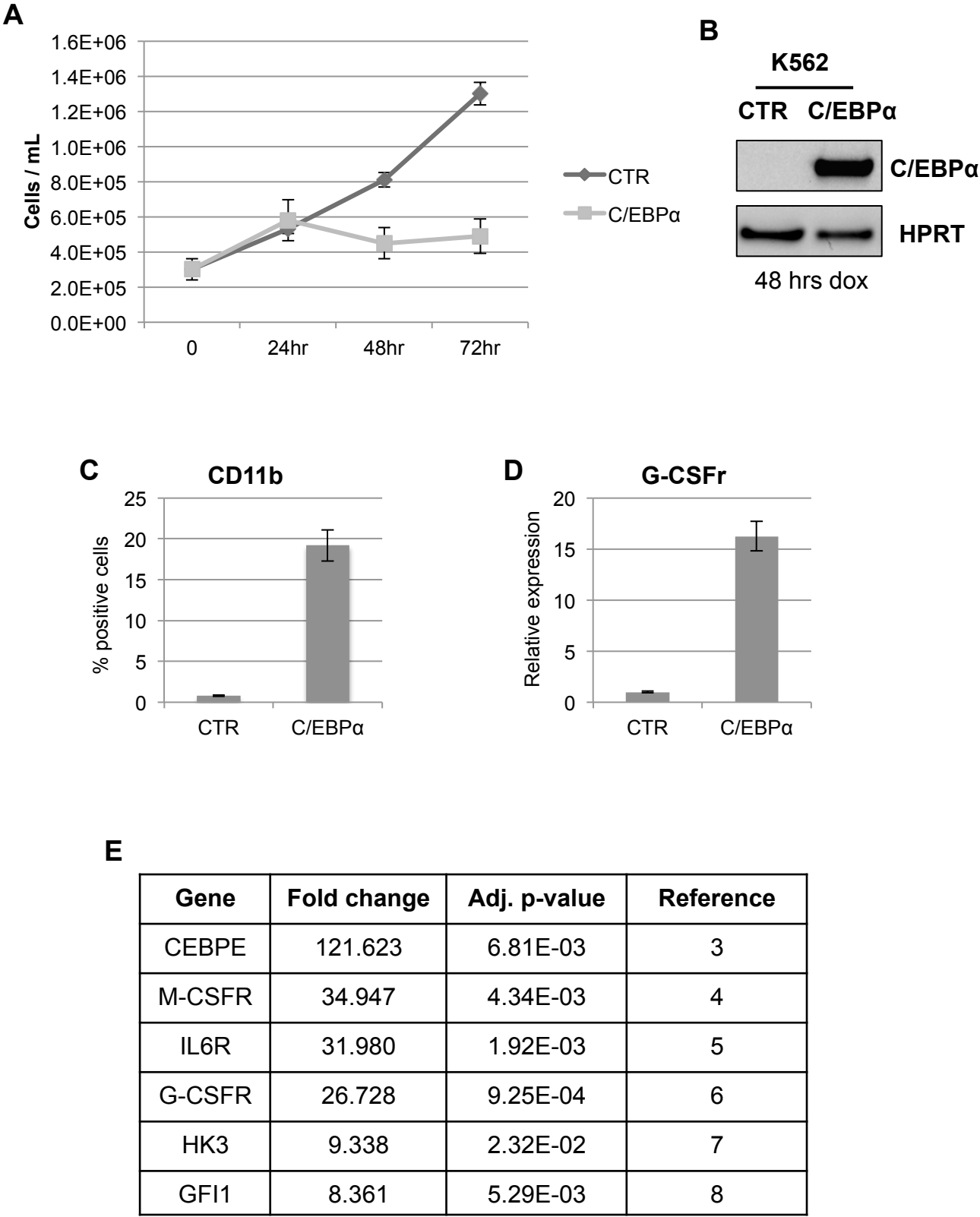

Supplement: Additional file 2: Figure S1. — Effects of C/EBPα expression in K562 cells. (A) Growth curve of K562 cells containing CTR and CEBPA expression cassette, respectively, after induction with Doxycyline. As expected, cells induced with C/EBPα cease to proliferate, while the CTR empty vector cells continue to proliferate. (B) Western blot confirms the expression of endogenous C/EBPα in the CEBPA stable cell line, and not in the CTR empty vector cell line. (C) FACS analysis for the granulocytic marker CD11b shows the percentage of positive cells within the given population after 48 hours of Doxycycline induction. (D) qRT-PCR analysis of the expression of the granulocytic marker GCSFR after 48 hrs of induction. Values were normalized with HPRT mRNA. The histograms represent the fold change of the relative expression ± SEM from three replicates. (E) Known C/EBPα transcriptional targets identified in our microarray analysis. [file 13045_2014_69_MOESM2_ESM.pdf]

## Slide 1
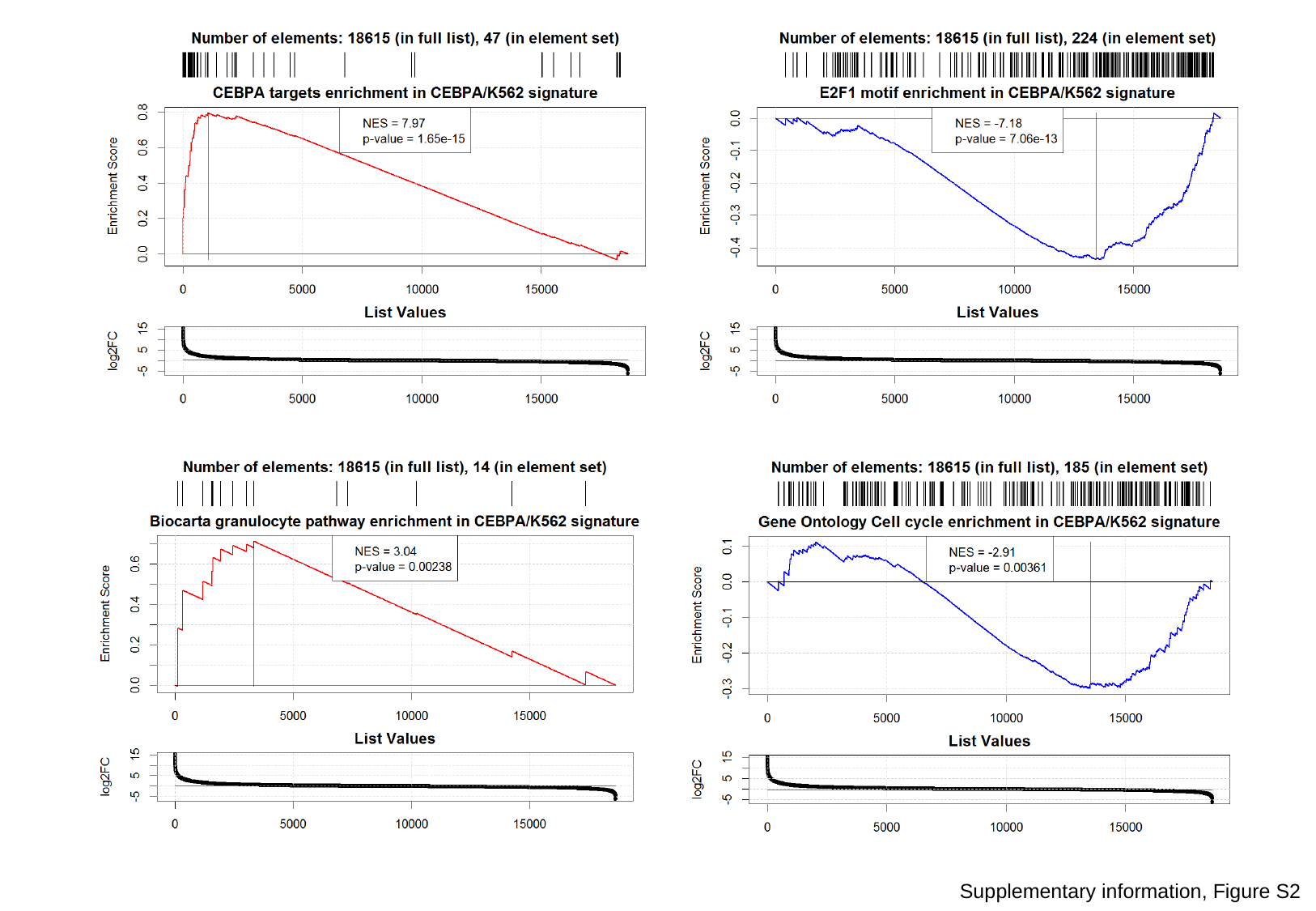

Supplementary information, Figure S2

Supplement: Additional file 5: Figure S2. — GSEA on CEBPA-regulated mRNAs. The enrichment score (ES; y-axis) reflects the degree to which a gene set is overrepresented in K562 expressing CEBPA. Each solid bar represents 1 gene within a gene set. Lower panels (List values) illustrate log2 fold change for the gene set. The GSEA histograms for the gene sets CEBPA, E2F1, “granulocyte pathway” and “cell cycle” are shown with the normalized enrichment score (NES) and p-values. [file 13045_2014_69_MOESM5_ESM.pptx]
