# Supplementary material for: CEBPA-regulated lncRNAs, new players in the study of acute myeloid leukemia
Source: J Hematol Oncol. 2014 Sep 25;7:69. doi: 10.1186/s13045-014-0069-1 (PMC4177583; doi:10.1186/s13045-014-0069-1)
Supplement: Additional file 3: Table S1. — CEBPA-regulated lncRNAs with significant differential expression (absolute fold change ≥ 2 and adjusted P value ≤ 0.05) identified in K562. (A) Up-regulated lncRNAs. (B) Down-regulated lncRNAs. [file 13045_2014_69_MOESM3_ESM.zip › 13045_2014_69_fig5/13045_2014_69_add5.pdf]

| Supplementary Table S1A. CEBPA-upregulated lncRNAs | | | | | | |
|---|---|---|---|---|---|---|
| **Name** | **Description** | **ProbeID** | **p.value** | **Adj.p.value** | **Fold_Change** | **Log2_Ratio** |
| VNN3 | Homo sapiens vanin 3 (VNN3), transcript variant 1, non-coding RNA [NR_028291] | A_23_P398449 | 1.11E-12 | 1.88E-08 | 82.17 | 6.361 |
| XLOC_005332 | BROAD Institute lincRNA (XLOC_005332), lincRNA [TCONS_00011845] | A_21_P0004892 | 4.04E-10 | 2.56E-06 | 11.949 | 3.579 |
| FLJ44054 | Homo sapiens uncharacterized LOC643365 (FLJ44054), non-coding RNA [NR_024609] | A_33_P3265314 | 9.29E-09 | 0.0000183 | 29.087 | 4.862 |
| LOC100507616 | PREDICTED: Homo sapiens hypothetical LOC100507616 (LOC100507616), miscRNA [XR_110328] | A_21_P0007844 | 8.82E-09 | 0.0000183 | 127.898 | 6.999 |
| PLIN2 | PREDICTED: Homo sapiens hypothetical LOC100509484 (LOC100509484), miscRNA [XR_132558] | A_21_P0014779 | 1.49E-08 | 0.0000244 | 40.3 | 5.333 |
| XLOC_005215 | BROAD Institute lincRNA (XLOC_005215), lincRNA [TCONS_00011753] | A_21_P0004865 | 2.15E-08 | 0.0000292 | 25.808 | 4.69 |
| XLOC_004100 | Q6D015_ERWCT (Q6D015) AraC-family transcriptional regulator, partial (5%) [THC2604688] | A_21_P0003517 | 2.02E-08 | 0.0000292 | 13.495 | 3.754 |
| XLOC_003734 | BROAD Institute lincRNA (XLOC_003734), lincRNA [TCONS_00008904] | A_21_P0003853 | 3.19E-08 | 0.0000344 | 95.877 | 6.583 |
| NUMB | BROAD Institute lincRNA (XLOC_011068), lincRNA [TCONS_00022790] | A_21_P0008460 | 4E-08 | 0.0000414 | 62.053 | 5.955 |
| XLOC_005151 | BROAD Institute lincRNA (XLOC_005151), lincRNA [TCONS_00011670] | A_21_P0004830 | 4.82E-08 | 0.0000461 | 21.618 | 4.434 |
| XLOC_012077 | BROAD Institute lincRNA (XLOC_012077), lincRNA [TCONS_00025271] | A_21_P0009210 | 5.13E-08 | 0.0000477 | 169.283 | 7.403 |
| SMAD5OS | Homo sapiens SMAD5 antisense RNA 1 (non-protein coding) (SMAD5-AS1), non-coding RNA [NR_026763] | A_23_P399797 | 5.77E-08 | 0.0000504 | 56.636 | 5.824 |
| NCRNA00110 | Homo sapiens GRIK1 antisense RNA 1 (non-protein coding) (GRIK1-AS1), non-coding RNA [NR_027021] | A_33_P3279362 | 7.92E-08 | 0.0000618 | 9.301 | 3.217 |
| LOC100506791 | PREDICTED: Homo sapiens hypothetical LOC100506791, transcript variant 1 (LOC100506791), miscRNA [XR_108578] | A_21_P0014143 | 1.41E-07 | 0.0000966 | 408.88 | 8.676 |
| DIRC3 | Homo sapiens disrupted in renal carcinoma 3 (DIRC3), non-coding RNA [NR_026597] | A_33_P3315134 | 1.93E-07 | 0.000115 | 144.826 | 7.178 |

| | | | | | | |
|---|---|---|---|---|---|---|
| XLOC_000539 | BROAD Institute lincRNA (XLOC_000539), lincRNA [TCONS_00000374] | A_21_P0001052 | 2.21E-07 | 0.000124 | 4.625 | 2.209 |
| XLOC_011513 | BROAD Institute lincRNA (XLOC_011513), lincRNA [TCONS_00023281] | A_21_P0008579 | 2.47E-07 | 0.000127 | 572.072 | 9.16 |
| XLOC_003317 | BROAD Institute lincRNA (XLOC_003317), lincRNA [TCONS_00005878] | A_21_P0002904 | 2.8E-07 | 0.000135 | 6.692 | 2.742 |
| XLOC_003513 | BROAD Institute lincRNA (XLOC_003513), lincRNA [TCONS_00008048] | A_21_P0003602 | 3.62E-07 | 0.000163 | 16.246 | 4.022 |
| LOC100128023 | Homo sapiens developmental pluripotency associated 2 pseudogene (LOC100128023), non-coding RNA [NR_027764] | A_21_P0000546 | 3.64E-07 | 0.000163 | 15.191 | 3.925 |
| XLOC_001412 | Unknown | A_19_P00321000 | 4.38E-07 | 0.000178 | 12.461 | 3.639 |
| XLOC_002473 | BROAD Institute lincRNA (XLOC_002473), lincRNA [TCONS_00004565] | A_21_P0002691 | 4.86E-07 | 0.000187 | 18.661 | 4.222 |
| XLOC_003234 | Q276A6_MYCFV (Q276A6) LemA precursor, partial (10%) [THC2678860] | A_21_P0003164 | 5.61E-07 | 0.000191 | 493.772 | 8.948 |
| XLOC_011670 | BROAD Institute lincRNA (XLOC_011670), lincRNA [TCONS_00024348] | A_21_P0008911 | 5.96E-07 | 0.000197 | 7.016 | 2.811 |
| XLOC_000555 | BROAD Institute lincRNA (XLOC_000555), lincRNA [TCONS_00001268] | A_21_P0001418 | 6.06E-07 | 0.000198 | 14.947 | 3.902 |
| CK300181 | PREDICTED: Homo sapiens hypothetical LOC100507507 (LOC100507507), miscRNA [XR_108783] | A_32_P19840 | 7.28E-07 | 0.000215 | 5.522 | 2.465 |
| XLOC_011289 | BROAD Institute lincRNA (XLOC_011289), lincRNA [TCONS_00023444] | A_21_P0008646 | 7E-07 | 0.000215 | 26.079 | 4.705 |
| LOC100505668 | PREDICTED: Homo sapiens hypothetical LOC100505668 (LOC100505668), miscRNA [XR_112556] | A_21_P0014715 | 7.12E-07 | 0.000215 | 10.496 | 3.392 |
| ANKRD26P1 | Homo sapiens ankyrin repeat domain 26 pseudogene 1 (ANKRD26P1), non-coding RNA [NR_026556] | A_33_P3291821 | 7.46E-07 | 0.000217 | 34.122 | 5.093 |
| LOC730101 | Homo sapiens uncharacterized LOC730101 (LOC730101), transcript variant 1, non-coding RNA [NR_024403] | A_24_P500422 | 8.21E-07 | 0.00023 | 6.643 | 2.732 |
| XLOC_006197 | BROAD Institute lincRNA (XLOC_006197), lincRNA [TCONS_00013557] | A_21_P0005412 | 8.74E-07 | 0.000238 | 3.87 | 1.952 |
| XLOC_006386 | BROAD Institute lincRNA (XLOC_006386), lincRNA [TCONS_00013744] | A_21_P0005482 | 8.93E-07 | 0.000239 | 3.243 | 1.697 |

| | | | | | | |
|---|---|---|---|---|---|---|
| XLOC_011068 | BROAD Institute lincRNA (XLOC_011068), lincRNA [TCONS_00022788] | A_21_P0008459 | 9.02E-07 | 0.000241 | 36.516 | 5.19 |
| A_19_P00800195 | Homo sapiens eukaryotic translation elongation factor 1 delta pseudogene 3 (EEF1DP3), non-coding RNA [NR_027062] | A_19_P00800195 | 9.24E-07 | 0.000245 | 8.997 | 3.169 |
| XLOC_001682 | BP423235 Homo sapiens small intestine Homo sapiens cDNA clone HIE06768r 3', mRNA sequence [BP423235] | A_21_P0001915 | 9.34E-07 | 0.000245 | 68.741 | 6.103 |
| GK3P | Homo sapiens glycerol kinase 3 pseudogene (GK3P), non-coding RNA [NR_026575] | A_21_P0012676 | 0.000001 | 0.000253 | 4.986 | 2.318 |
| XLOC_011064 | DA429566 COLON2 Homo sapiens cDNA clone COLON2005610 5', mRNA sequence [DA429566] | A_21_P0008456 | 1.07E-06 | 0.000258 | 14.257 | 3.834 |
| XLOC_004299 | BROAD Institute lincRNA (XLOC_004299), lincRNA [TCONS_00009877] | A_21_P0004230 | 1.12E-06 | 0.000265 | 30.384 | 4.925 |
| LOC100287559 | Homo sapiens uncharacterized LOC100287559 (LOC100287559), non-coding RNA [NR_040107] | A_21_P0000902 | 1.14E-06 | 0.000269 | 27.544 | 4.784 |
| C2orf73 | BROAD Institute lincRNA (XLOC_001473), lincRNA [TCONS_00003679] | A_21_P0002215 | 1.21E-06 | 0.000277 | 41.707 | 5.382 |
| LOC440040 | Homo sapiens glutamate receptor, metabotropic 5 pseudogene (LOC440040), non-coding RNA [NR_027044] | A_23_P150198 | 1.36E-06 | 0.0003 | 6.868 | 2.78 |
| XLOC_004874 | BROAD Institute lincRNA (XLOC_004874), lincRNA [TCONS_00010958] | A_21_P0004561 | 1.47E-06 | 0.000309 | 55.457 | 5.793 |
| LOC100507053 | Homo sapiens uncharacterized LOC100507053 (LOC100507053), non-coding RNA [NR_037884] | A_21_P0000700 | 1.65E-06 | 0.000328 | 18.179 | 4.184 |
| XLOC_004439 | BROAD Institute lincRNA (XLOC_004439), lincRNA [TCONS_00010003] | A_21_P0004268 | 1.66E-06 | 0.000328 | 3.194 | 1.676 |
| LOC100506291 | PREDICTED: Homo sapiens hypothetical LOC100506291, transcript variant 1 (LOC100506291), miscRNA [XR_110464] | A_21_P0014596 | 1.72E-06 | 0.000334 | 2.732 | 1.45 |
| LOC100129269 | Homo sapiens uncharacterized LOC100129269 (LOC100129269), non-coding RNA [NR_034126] | A_33_P3399768 | 1.79E-06 | 0.000344 | 3.711 | 1.892 |
| XLOC_004293 | BROAD Institute lincRNA (XLOC_004293), lincRNA [TCONS_00009352] | A_21_P0003971 | 1.97E-06 | 0.000373 | 21.852 | 4.45 |
| A_19_P00811196 | Homo sapiens uncharacterized LOC100505854 (LOC100505854), non-coding RNA [NR_038361] | A_19_P00811196 | 2.21E-06 | 0.000394 | 2.939 | 1.555 |

| | | | | | | |
|---|---|---|---|---|---|---|
| CLEC4GP1 | Homo sapiens C-type lectin domain family 4, member G pseudogene 1 (CLEC4GP1), non-coding RNA [NR_002931] | A_33_P3246178 | 2.21E-06 | 0.000394 | 8.764 | 3.132 |
| XLOC_005940 | BROAD Institute lincRNA (XLOC_005940), lincRNA [TCONS_00012374] | A_21_P0005094 | 2.25E-06 | 0.000397 | 6.219 | 2.637 |
| XLOC_l2_010650 | DB230865 TRACH3 Homo sapiens cDNA clone TRACH3024308 5', mRNA sequence [DB230865] | A_21_P0012723 | 2.35E-06 | 0.000402 | 6.04 | 2.595 |
| OR7E140P | PREDICTED: Homo sapiens hypothetical LOC729732 (LOC729732), miscRNA [XR_132886] | A_33_P3211793 | 2.66E-06 | 0.000423 | 11.914 | 3.575 |
| LOC100652787 | PREDICTED: Homo sapiens hypothetical LOC100652787 (LOC100652787), miscRNA [XR_132806] | A_21_P0014888 | 2.65E-06 | 0.000423 | 4.253 | 2.089 |
| A_19_P00317856 | Q6P4E4_HUMAN (Q6P4E4) RPL37A protein, partial (54%) [THC2593447] | A_19_P00317856 | 2.8E-06 | 0.00043 | 3.133 | 1.648 |
| XLOC_l2_001669 | ALU1_HUMAN (P39188) Alu subfamily J sequence contamination warning entry, partial (5%) [THC2689185] | A_21_P0010838 | 2.87E-06 | 0.000432 | 5.554 | 2.474 |
| XLOC_002921 | IL2-NT0102-160600-105-C03 NT0102 Homo sapiens cDNA, mRNA sequence [BF366239] | A_21_P0002805 | 2.88E-06 | 0.000432 | 4.64 | 2.214 |
| XLOC_l2_002560 | PREDICTED: Homo sapiens hypothetical LOC729732 (LOC729732), miscRNA [XR_132886] | A_21_P0011004 | 3.06E-06 | 0.000447 | 15.949 | 3.995 |
| LOC100505729 | PREDICTED: Homo sapiens hypothetical LOC100505729 (LOC100505729), miscRNA [XR_108524] | A_21_P0014131 | 3.14E-06 | 0.000455 | 8.914 | 3.156 |
| LOC100507466 | Homo sapiens uncharacterized LOC100507466 (LOC100507466), non-coding RNA [NR_038251] | A_21_P0000738 | 3.16E-06 | 0.000456 | 24.241 | 4.599 |
| XLOC_011510 | BROAD Institute lincRNA (XLOC_011510), lincRNA [TCONS_00024144] | A_21_P0008850 | 4.39E-06 | 0.000552 | 3.189 | 1.673 |
| XLOC_005327 | NM_105511 ATP binding {Arabidopsis thaliana} (exp=-1; wgp=0; cg=0), partial (4%) [THC2649843] | A_19_P00319254 | 4.46E-06 | 0.00056 | 7.724 | 2.949 |
| MGC45922 | Homo sapiens uncharacterized LOC284365 (MGC45922), non-coding RNA [NR_038359] | A_21_P0009655 | 4.59E-06 | 0.00057 | 3.399 | 1.765 |
| XLOC_l2_007571 | BROAD Institute lincRNA (XLOC_l2_007571), lincRNA [TCONS_l2_00014003] | A_21_P0011855 | 4.9E-06 | 0.00059 | 33.773 | 5.078 |

| | | | | | | |
|---|---|---|---|---|---|---|
| XLOC_l2_004866 | Q96C45_HUMAN (Q96C45) ULK4 protein, partial (17%) [THC2500926] | A_21_P0011363 | 5.19E-06 | 0.000609 | 19.537 | 4.288 |
| XLOC_l2_012621 | BROAD Institute lincRNA (XLOC_l2_012621), lincRNA [TCONS_l2_00024305] | A_21_P0013030 | 5.49E-06 | 0.000634 | 6.521 | 2.705 |
| LOC96610 | Homo sapiens BMS1 homolog, ribosome assembly protein (yeast) pseudogene (LOC96610), non-coding RNA [NR_027293] | A_33_P3376958 | 5.65E-06 | 0.00064 | 5.659 | 2.501 |
| C9orf110 | Homo sapiens long intergenic non-protein coding RNA 256B (LINC00256B), non-coding RNA [NR_024376] | A_32_P99347 | 6.38E-06 | 0.000694 | 4.308 | 2.107 |
| TRIM53 | Homo sapiens tripartite motif containing 53, pseudogene (TRIM53P), non-coding RNA [NR_028346] | A_24_P16353 | 6.43E-06 | 0.000695 | 5.464 | 2.45 |
| XLOC_l2_013530 | RST11549 Athersys RAGE Library Homo sapiens cDNA, mRNA sequence [BG192435] | A_21_P0013256 | 6.7E-06 | 0.000712 | 10.382 | 3.376 |
| XLOC_l2_014024 | BROAD Institute lincRNA (XLOC_l2_014024), lincRNA [TCONS_l2_00026920] | A_21_P0013351 | 7.05E-06 | 0.000732 | 145.791 | 7.188 |
| LOC100505616 | PREDICTED: Homo sapiens hypothetical LOC100505616, transcript variant 2 (LOC100505616), miscRNA [XR_110597] | A_21_P0014623 | 7.08E-06 | 0.000732 | 3.539 | 1.823 |
| XLOC_l2_013302 | BROAD Institute lincRNA (XLOC_l2_013302), lincRNA [TCONS_l2_00027067] | A_21_P0013374 | 7.47E-06 | 0.000748 | 16.998 | 4.087 |
| XLOC_l2_010569 | 603066169F1 NIH_MGC_118 Homo sapiens cDNA clone IMAGE:5215217 5', mRNA sequence [BI908343] | A_21_P0012570 | 7.6E-06 | 0.000755 | 6.924 | 2.792 |
| XLOC_005411 | BROAD Institute lincRNA (XLOC_005411), lincRNA [TCONS_00011910] | A_21_P0004916 | 8.19E-06 | 0.000797 | 11.701 | 3.549 |
| LOC100507195 | PREDICTED: Homo sapiens hypothetical LOC100507195 (LOC100507195), miscRNA [XR_110374] | A_21_P0014580 | 8.3E-06 | 0.000804 | 25.932 | 4.697 |
| AL133018 | Homo sapiens CTAGE family, member 11, pseudogene (CTAGE11P), non-coding RNA [NR_027466] | A_24_P332623 | 8.53E-06 | 0.00082 | 4.347 | 2.12 |
| XLOC_010172 | DB511330 RIKEN full-length enriched human cDNA library, testis Homo sapiens cDNA clone H013024E03 3', mRNA sequence [DB511330] | A_21_P0007888 | 8.61E-06 | 0.00082 | 12.563 | 3.651 |
| XLOC_l2_012570 | Q5LQX0_SILPO (Q5LQX0) Phosphoserine phosphatase , partial (5%) [THC2656110] | A_21_P0013025 | 8.58E-06 | 0.00082 | 35.873 | 5.165 |

| | | | | | | |
|---|---|---|---|---|---|---|
| MIG7 | PREDICTED: Homo sapiens hypothetical LOC100132240 (LOC100132240), miscRNA [XR_132800] | A_32_P63562 | 8.79E-06 | 0.00083 | 5.824 | 2.542 |
| XLOC_013942 | BROAD Institute lincRNA (XLOC_013942), lincRNA [TCONS_00029020] | A_21_P0010246 | 8.94E-06 | 0.000839 | 8.973 | 3.166 |
| XLOC_003378 | BROAD Institute lincRNA (XLOC_003378), lincRNA [TCONS_00006739] | A_21_P0003204 | 9.75E-06 | 0.000879 | 24.678 | 4.625 |
| XLOC_l2_006021 | DB117598 THYMU2 Homo sapiens cDNA clone THYMU2024382 5', mRNA sequence [DB117598] | A_21_P0011578 | 9.78E-06 | 0.00088 | 32.415 | 5.019 |
| LOC100506226 | PREDICTED: Homo sapiens hypothetical LOC100506226, transcript variant 2 (LOC100506226), miscRNA [XR_109085] | A_21_P0011038 | 1.01E-05 | 0.000895 | 18.787 | 4.232 |
| XLOC_l2_010386 | RST23433 Athersys RAGE Library Homo sapiens cDNA, mRNA sequence [BG204039] | A_21_P0012465 | 1.02E-05 | 0.000899 | 46.527 | 5.54 |
| XLOC_l2_005490 | BROAD Institute lincRNA (XLOC_l2_005490), lincRNA [TCONS_l2_00010170] | A_21_P0011482 | 1.09E-05 | 0.000919 | 124.899 | 6.965 |
| XLOC_006815 | BROAD Institute lincRNA (XLOC_006815), lincRNA [TCONS_00014726] | A_21_P0005704 | 1.11E-05 | 0.000925 | 7.813 | 2.966 |
| XLOC_013934 | BROAD Institute lincRNA (XLOC_013934), lincRNA [TCONS_00029012] | A_21_P0010241 | 1.12E-05 | 0.000925 | 3.873 | 1.954 |
| XLOC_005070 | BROAD Institute lincRNA (XLOC_005070), lincRNA [TCONS_00011079] | A_21_P0004578 | 1.15E-05 | 0.000942 | 161.335 | 7.334 |
| A_19_P00806499 | Homo sapiens SPG20 opposite strand (SPG20OS), transcript variant 1, non-coding RNA [NR_045180] | A_19_P00806499 | 1.19E-05 | 0.00095 | 7.971 | 2.995 |
| LOC100506252 | PREDICTED: Homo sapiens hypothetical LOC100506252 (LOC100506252), miscRNA [XR_109392] | A_21_P0014349 | 1.19E-05 | 0.00095 | 5.73 | 2.518 |
| AKR1CL1 | Homo sapiens aldo-keto reductase family 1, member C-like 1 (AKR1CL1), non-coding RNA [NR_027916] | A_21_P0010854 | 1.18E-05 | 0.00095 | 43.666 | 5.448 |
| XLOC_010385 | BROAD Institute lincRNA (XLOC_010385), lincRNA [TCONS_00021787] | A_21_P0008063 | 1.19E-05 | 0.00095 | 43.264 | 5.435 |
| LOC100652854 | PREDICTED: Homo sapiens hypothetical LOC100652854 (LOC100652854), miscRNA [XR_132672] | A_21_P0014825 | 1.21E-05 | 0.000961 | 30.43 | 4.927 |
| 1/2-SBSRNA4 | GB | A_21_P0012785 | 1.24E-05 | 0.000979 | 7.324 | 2.873 |

| | | | | | | |
|---|---|---|---|---|---|---|
| MEG8 | Homo sapiens maternally expressed 8 (non-protein coding) (MEG8), non-coding RNA [NR_024149] | A_33_P3360382 | 1.28E-05 | 0.000997 | 4.87 | 2.284 |
| XLOC_001683 | BX101448 Soares_testis_NHT Homo sapiens cDNA clone IMAGp998J084407, mRNA sequence [BX101448] | A_21_P0001916 | 1.34E-05 | 0.00102 | 10.664 | 3.415 |
| LOC100505994 | PREDICTED: Homo sapiens hypothetical LOC100505994 (LOC100505994), miscRNA [XR_108596] | A_21_P0004569 | 1.43E-05 | 0.00107 | 11.275 | 3.495 |
| XLOC_004009 | BROAD Institute lincRNA (XLOC_004009), lincRNA [TCONS_00007791] | A_21_P0003484 | 1.45E-05 | 0.00108 | 26.838 | 4.746 |
| XLOC_014364 | PT1.3_03_G05.r tumor1 Homo sapiens cDNA 5', mRNA sequence [AI525606] | A_21_P0010438 | 1.46E-05 | 0.00108 | 2.551 | 1.351 |
| LOC100507594 | PREDICTED: Homo sapiens hypothetical LOC100507594 (LOC100507594), miscRNA [XR_110327] | A_21_P0014567 | 0.000015 | 0.0011 | 10.248 | 3.357 |
| LOC100506922 | PREDICTED: Homo sapiens hypothetical LOC100506922 (LOC100506922), miscRNA [XR_109888] | A_21_P0011849 | 1.62E-05 | 0.00114 | 3.846 | 1.943 |
| LOC100507480 | PREDICTED: Homo sapiens hypothetical LOC100507480 (LOC100507480), miscRNA [XR_109174] | A_21_P0008607 | 1.61E-05 | 0.00114 | 6.407 | 2.68 |
| XLOC_000340 | BROAD Institute lincRNA (XLOC_000340), lincRNA [TCONS_00001094] | A_21_P0001699 | 1.63E-05 | 0.00114 | 34.655 | 5.115 |
| LOC338963 | Homo sapiens epididymal protein pseudogene (LOC338963), non-coding RNA [NR_034139] | A_33_P3390236 | 1.68E-05 | 0.00116 | 3.62 | 1.856 |
| XLOC_000375 | KSR1_HUMAN (Q8IVT5) Kinase suppressor of ras-1 (Kinase suppressor of ras), partial (8%) [THC2609213] | A_21_P0001701 | 1.69E-05 | 0.00117 | 11.148 | 3.479 |
| ENST00000397645 | Homo sapiens uncharacterized LOC647323 (LOC647323), non-coding RNA [NR_033944] | A_24_P376379 | 1.73E-05 | 0.00118 | 14.455 | 3.854 |
| XLOC_011228 | BROAD Institute lincRNA (XLOC_011228), lincRNA [TCONS_00023386] | A_21_P0008628 | 1.72E-05 | 0.00118 | 3.892 | 1.961 |
| XLOC_003676 | BC069124 interleukin 21 {Homo sapiens} (exp=-1; wgp=0; cg=0), partial (30%) [THC2606123] | A_21_P0003646 | 1.78E-05 | 0.0012 | 7.948 | 2.991 |
| XLOC_001980 | BROAD Institute lincRNA (XLOC_001980), lincRNA [TCONS_00004138] | A_21_P0002400 | 1.87E-05 | 0.00123 | 6.674 | 2.739 |

| | | | | | | |
|---|---|---|---|---|---|---|
| A_21_P0014861 | PREDICTED: Homo sapiens hypothetical LOC100652952 (LOC100652952), miscRNA [XR_132749] | A_21_P0014861 | 1.87E-05 | 0.00123 | 2.256 | 1.174 |
| XLOC_005664 | BROAD Institute lincRNA (XLOC_005664), lincRNA [TCONS_00012134] | A_21_P0005017 | 1.92E-05 | 0.00126 | 6.676 | 2.739 |
| XLOC_l2_010493 | BROAD Institute lincRNA (XLOC_l2_010493), lincRNA [TCONS_l2_00019671] | A_21_P0012477 | 2.05E-05 | 0.00131 | 4.056 | 2.02 |
| STAMBP | PREDICTED: Homo sapiens hypothetical LOC100652991 (LOC100652991), miscRNA [XR_132704] | A_21_P0014839 | 2.07E-05 | 0.00131 | 2.195 | 1.134 |
| A_19_P00318323 | Homo sapiens long intergenic non-protein coding RNA 340 (LINC00340), non-coding RNA [NR_015410] | A_19_P00318323 | 0.000021 | 0.00133 | 8.843 | 3.144 |
| XLOC_001868 | BROAD Institute lincRNA (XLOC_001868), lincRNA [TCONS_00003111] | A_21_P0001971 | 2.12E-05 | 0.00133 | 3.205 | 1.681 |
| A_19_P00317277 | Q1YA75_STAAU (Q1YA75) ABC transporter related, partial (5%) [THC2632354] | A_19_P00317277 | 2.28E-05 | 0.00139 | 27.866 | 4.8 |
| XLOC_010271 | BROAD Institute lincRNA (XLOC_010271), lincRNA [TCONS_00021009] | A_21_P0007811 | 2.39E-05 | 0.00143 | 25.274 | 4.66 |
| FLJ22536 | Homo sapiens long intergenic non-protein coding RNA 340 (LINC00340), non-coding RNA [NR_015410] | A_24_P838448 | 0.000024 | 0.00144 | 8.818 | 3.14 |
| FLJ23569 | Homo sapiens A1BG antisense RNA 1 (non-protein coding) (A1BG-AS1), non-coding RNA [NR_015380] | A_24_P721699 | 2.47E-05 | 0.00146 | 2.391 | 1.258 |
| hCG_38984 | PREDICTED: Homo sapiens hCG38984 (LOC345051), miscRNA [XR_109844] | A_33_P3340324 | 2.46E-05 | 0.00146 | 3.304 | 1.724 |
| XLOC_005900 | BROAD Institute lincRNA (XLOC_005900), lincRNA [TCONS_00012818] | A_21_P0005078 | 0.000025 | 0.00148 | 3.339 | 1.739 |
| A_21_P0014067 | PREDICTED: Homo sapiens hypothetical LOC100506775, transcript variant 2 (LOC100506775), miscRNA [XR_108333] | A_21_P0014067 | 2.59E-05 | 0.0015 | 39.952 | 5.32 |
| XLOC_007929 | BROAD Institute lincRNA (XLOC_007929), lincRNA [TCONS_00017139] | A_21_P0006482 | 2.69E-05 | 0.00153 | 10.468 | 3.388 |
| XLOC_000476 | BROAD Institute lincRNA (XLOC_000476), lincRNA [TCONS_00001210] | A_21_P0001390 | 2.66E-05 | 0.00153 | 8.784 | 3.135 |
| SEPT7P2 | BROAD Institute lincRNA (XLOC_l2_013808), lincRNA [TCONS_l2_00026542] | A_21_P0013299 | 2.66E-05 | 0.00153 | 2.697 | 1.432 |

| | | | | | | |
|---|---|---|---|---|---|---|
| LOC254128 | Homo sapiens uncharacterized LOC254128 (LOC254128), transcript variant 3, non-coding RNA [NR_037857] | A_33_P3576853 | 2.71E-05 | 0.00154 | 2.567 | 1.36 |
| C9orf109 | Homo sapiens long intergenic non-protein coding RNA 256A (LINC00256A), non-coding RNA [NR_024366] | A_24_P687326 | 2.74E-05 | 0.00155 | 3.359 | 1.748 |
| XLOC_008777 | AC007764 F22C12.23 {Arabidopsis thaliana} (exp=0; wgp=1; cg=0), partial (6%) [THC2737253] | A_21_P0006746 | 2.73E-05 | 0.00155 | 8.06 | 3.011 |
| XLOC_l2_007191 | BROAD Institute lincRNA (XLOC_l2_007191), lincRNA [TCONS_l2_00015236] | A_21_P0011792 | 2.77E-05 | 0.00156 | 46.971 | 5.554 |
| XLOC_000745 | AV715291 DCB Homo sapiens cDNA clone DCBCDE09 5', mRNA sequence [AV715291] | A_21_P0001109 | 2.83E-05 | 0.00158 | 39.553 | 5.306 |
| RP11-374F3.4 | Homo sapiens long intergenic non-protein coding RNA 426 (LINC00426), non-coding RNA [NR_024464] | A_33_P3333960 | 2.87E-05 | 0.00159 | 2.994 | 1.582 |
| XLOC_012537 | BROAD Institute lincRNA (XLOC_012537), lincRNA [TCONS_00025711] | A_21_P0009340 | 3.11E-05 | 0.00168 | 99.064 | 6.63 |
| LOC100131316 | PREDICTED: Homo sapiens hypothetical LOC100131316 (LOC100131316), miscRNA [XR_109900] | A_21_P0011857 | 3.27E-05 | 0.00172 | 13.872 | 3.794 |
| XLOC_l2_008221 | BROAD Institute lincRNA (XLOC_l2_008221), lincRNA [TCONS_l2_00014844] | A_21_P0011956 | 3.27E-05 | 0.00172 | 9.286 | 3.215 |
| A_19_P00320644 | Homo sapiens carbonic anhydrase VB pseudogene 1 (CA5BP1), non-coding RNA [NR_026551] | A_19_P00320644 | 3.33E-05 | 0.00174 | 4.664 | 2.222 |
| LOC647500 | PREDICTED: Homo sapiens hypothetical LOC100132057 (LOC100132057), miscRNA [XR_110830] | A_32_P515431 | 3.39E-05 | 0.00175 | 4.963 | 2.311 |
| XLOC_004644 | BROAD Institute lincRNA (XLOC_004644), lincRNA [TCONS_00010837] | A_21_P0004540 | 3.38E-05 | 0.00175 | 16.279 | 4.025 |
| XLOC_005810 | BROAD Institute lincRNA (XLOC_005810), lincRNA [TCONS_00012257] | A_19_P00316185 | 3.42E-05 | 0.00176 | 17.726 | 4.148 |
| HMGA1P4 | PREDICTED: Homo sapiens hypothetical LOC100506080 (LOC100506080), miscRNA [XR_108949] | A_21_P0014231 | 0.000035 | 0.00178 | 6.266 | 2.648 |
| XLOC_l2_001760 | Q2DTM3_9CHLR (Q2DTM3) DNA repair protein RadC, partial (5%) [THC2659873] | A_21_P0010843 | 3.51E-05 | 0.00178 | 6.646 | 2.732 |

| | | | | | | |
|---|---|---|---|---|---|---|
| XLOC_001856 | BROAD Institute lincRNA (XLOC_001856), lincRNA [TCONS_00004018] | A_21_P0002349 | 0.000036 | 0.00181 | 2.847 | 1.509 |
| XLOC_010921 | BROAD Institute lincRNA (XLOC_010921), lincRNA [TCONS_00022619] | A_21_P0008382 | 3.61E-05 | 0.00181 | 9.921 | 3.31 |
| XLOC_001595 | BROAD Institute lincRNA (XLOC_001595), lincRNA [TCONS_00003800] | A_21_P0002261 | 3.72E-05 | 0.00184 | 13.582 | 3.764 |
| XLOC_012021 | Q5ADQ7_CANAL (Q5ADQ7) Possible secreted protein, partial (6%) [THC2503158] | A_21_P0008887 | 3.84E-05 | 0.00187 | 2.857 | 1.514 |
| LOC643802 | BROAD Institute lincRNA (XLOC_l2_005490), lincRNA [TCONS_l2_00010465] | A_21_P0011505 | 3.88E-05 | 0.00188 | 10.825 | 3.436 |
| XLOC_l2_013741 | BROAD Institute lincRNA (XLOC_l2_013741), lincRNA [TCONS_l2_00026420] | A_21_P0013288 | 3.85E-05 | 0.00188 | 11.495 | 3.523 |
| LOC729603 | Homo sapiens calcium binding protein P22 pseudogene (LOC729603), non-coding RNA [NR_003288] | A_33_P3410859 | 3.98E-05 | 0.00191 | 2.325 | 1.217 |
| LOC100506328 | PREDICTED: Homo sapiens hypothetical LOC100506328 (LOC100506328), miscRNA [XR_109927] | A_21_P0011833 | 3.98E-05 | 0.00191 | 15.209 | 3.927 |
| CA5BL | Homo sapiens carbonic anhydrase VB pseudogene 1 (CA5BP1), non-coding RNA [NR_026551] | A_24_P136683 | 4.11E-05 | 0.00193 | 4.899 | 2.292 |
| CA5BP | Homo sapiens carbonic anhydrase VB pseudogene 1 (CA5BP1), non-coding RNA [NR_026551] | A_33_P3316061 | 4.09E-05 | 0.00193 | 4.977 | 2.315 |
| XLOC_l2_007456 | BROAD Institute lincRNA (XLOC_l2_007456), lincRNA [TCONS_l2_00013854] | A_21_P0011831 | 4.26E-05 | 0.00197 | 62.102 | 5.957 |
| XLOC_l2_001853 | BROAD Institute lincRNA (XLOC_l2_001853), lincRNA [TCONS_l2_00003387] | A_21_P0010857 | 4.38E-05 | 0.002 | 3.499 | 1.807 |
| LOC100506934 | PREDICTED: Homo sapiens hypothetical LOC100506934 (LOC100506934), miscRNA [XR_109940] | A_21_P0014461 | 4.52E-05 | 0.00204 | 23.698 | 4.567 |
| A_21_P0014744 | PREDICTED: Homo sapiens hypothetical LOC100287049 (LOC100287049), miscRNA [XR_132477] | A_21_P0014744 | 4.51E-05 | 0.00204 | 2.856 | 1.514 |
| GCFC1-AS1 | Homo sapiens GCFC1 antisense RNA 1 (non-protein coding) (GCFC1-AS1), transcript variant 2, non-coding RNA [NR_038880] | A_21_P0000824 | 4.68E-05 | 0.00208 | 2.977 | 1.574 |

| | | | | | | |
|---|---|---|---|---|---|---|
| XLOC_l2_006152 | Q6ZQH5_MOUSE (Q6ZQH5) MKIAA4250 protein (Fragment), partial (8%) [THC2556187] | A_21_P0011598 | 4.67E-05 | 0.00208 | 8.397 | 3.07 |
| XLOC_l2_011283 | BROAD Institute lincRNA (XLOC_l2_011283), lincRNA [TCONS_l2_00021309] | A_21_P0012683 | 4.71E-05 | 0.00208 | 5.309 | 2.408 |
| XLOC_l2_005194 | BROAD Institute lincRNA (XLOC_l2_005194), lincRNA [TCONS_l2_00009698] | A_21_P0011436 | 4.98E-05 | 0.00214 | 3.788 | 1.922 |
| XLOC_012515 | BROAD Institute lincRNA (XLOC_012515), lincRNA [TCONS_00025694] | A_21_P0009331 | 5.04E-05 | 0.00215 | 49.984 | 5.643 |
| XLOC_005315 | PREDICTED: Homo sapiens hypothetical LOC100505985 (LOC100505985), miscRNA [XR_108661] | A_21_P0004654 | 0.000051 | 0.00217 | 31.047 | 4.956 |
| LOC728052 | PREDICTED: Homo sapiens hypothetical LOC728052 (LOC728052), miscRNA [XR_108619] | A_21_P0013086 | 5.21E-05 | 0.00219 | 8.641 | 3.111 |
| BG207573 | PREDICTED: Homo sapiens hypothetical LOC100506220 (LOC100506220), miscRNA [XR_110895] | A_33_P3402211 | 5.36E-05 | 0.00224 | 20.234 | 4.339 |
| XLOC_l2_004306 | PREDICTED: Homo sapiens hypothetical LOC100507257 (LOC100507257), miscRNA [XR_111467] | A_21_P0011281 | 5.45E-05 | 0.00226 | 3.665 | 1.874 |
| XLOC_l2_004859 | PREDICTED: Homo sapiens golgin subfamily A member 8-like protein 2-like (LOC100652910), mRNA [XM_003403457] | A_21_P0011361 | 5.45E-05 | 0.00226 | 3.47 | 1.795 |
| XLOC_l2_001134 | AF164609 Gag-Pro-Pol protein {Homo sapiens} (exp=-1; wgp=0; cg=0), partial (6%) [THC2668242] | A_21_P0010778 | 6.24E-05 | 0.00247 | 24.526 | 4.616 |
| XLOC_010132 | BROAD Institute lincRNA (XLOC_010132), lincRNA [TCONS_00020847] | A_21_P0007726 | 6.51E-05 | 0.00252 | 9.376 | 3.229 |
| LOC100510007 | PREDICTED: Homo sapiens hypothetical LOC100510007 (LOC100510007), miscRNA [XR_110765] | A_21_P0014627 | 6.71E-05 | 0.00258 | 3.67 | 1.876 |
| XLOC_l2_007656 | BROAD Institute lincRNA (XLOC_l2_007656), lincRNA [TCONS_l2_00014098] | A_21_P0011867 | 6.72E-05 | 0.00258 | 5.344 | 2.418 |
| A_19_P00807628 | Homo sapiens uncharacterized LOC441172 (FLJ46906), non-coding RNA [NR_033896] | A_19_P00807628 | 7.09E-05 | 0.00267 | 4.365 | 2.126 |
| XLOC_013300 | BROAD Institute lincRNA (XLOC_013300), lincRNA [TCONS_00027300] | A_21_P0009763 | 7.13E-05 | 0.00268 | 3.907 | 1.966 |

| | | | | | | |
|---|---|---|---|---|---|---|
| XLOC_005147 | BROAD Institute lincRNA (XLOC_005147), lincRNA [TCONS_00011665] | A_21_P0004827 | 7.34E-05 | 0.00272 | 21.071 | 4.397 |
| XLOC_013274 | BROAD Institute lincRNA (XLOC_013274), lincRNA [TCONS_00027239] | A_21_P0009747 | 7.57E-05 | 0.00277 | 2.308 | 1.206 |
| BRD7P3 | Homo sapiens bromodomain containing 7 pseudogene 3 (BRD7P3), non-coding RNA [NR_002730] | A_33_P3313660 | 7.67E-05 | 0.00278 | 2.59 | 1.373 |
| XLOC_010117 | BROAD Institute lincRNA (XLOC_010117), lincRNA [TCONS_00021347] | A_21_P0007881 | 7.61E-05 | 0.00278 | 23.179 | 4.535 |
| XLOC_003125 | BROAD Institute lincRNA (XLOC_003125), lincRNA [TCONS_00006511] | A_21_P0003118 | 7.97E-05 | 0.00285 | 10.42 | 3.381 |
| XLOC_l2_013080 | STYFLIK complete fliJ sequence available under accession number M62408 {Salmonella typhimurium} (exp=-1; wgp=0; cg=0), partial (22%) [THC2635201] | A_21_P0013150 | 8.05E-05 | 0.00286 | 37.014 | 5.21 |
| XLOC_004924 | Q26195_PLAVI (Q26195) Pva1 protein, partial (11%) [THC2719986] | A_21_P0004566 | 8.11E-05 | 0.00287 | 35.402 | 5.146 |
| LOC100507309 | PREDICTED: Homo sapiens hypothetical LOC100507309 (LOC100507309), miscRNA [XR_109012] | A_21_P0014247 | 0.000082 | 0.00288 | 7.412 | 2.89 |
| NCRNA00171 | Homo sapiens ZNRD1 antisense RNA 1 (non-protein coding) (ZNRD1-AS1), non-coding RNA [NR_026751] | A_33_P3219840 | 8.24E-05 | 0.00289 | 4.346 | 2.12 |
| A_19_P00318883 | Homo sapiens uncharacterized LOC100505854 (LOC100505854), non-coding RNA [NR_038361] | A_19_P00318883 | 8.35E-05 | 0.00291 | 2.907 | 1.539 |
| XLOC_008114 | BROAD Institute lincRNA (XLOC_008114), lincRNA [TCONS_00017292] | A_21_P0006527 | 8.34E-05 | 0.00291 | 56.47 | 5.819 |
| XLOC_011379 | ALU7_HUMAN (P39194) Alu subfamily SQ sequence contamination warning entry, partial (28%) [THC2505609] | A_21_P0008692 | 8.33E-05 | 0.00291 | 16.67 | 4.059 |
| LOC100653206 | PREDICTED: Homo sapiens hypothetical LOC100653206 (LOC100653206), miscRNA [XR_133524] | A_21_P0014944 | 8.64E-05 | 0.00297 | 13.434 | 3.748 |
| XLOC_008766 | BROAD Institute lincRNA (XLOC_008766), lincRNA [TCONS_00017945] | A_21_P0006743 | 8.76E-05 | 0.00299 | 4.626 | 2.21 |
| LOC152225 | Homo sapiens uncharacterized LOC152225 (LOC152225), non-coding RNA [NR_026934] | A_33_P3395028 | 8.97E-05 | 0.00303 | 7.397 | 2.887 |

| | | | | | | |
|---|---|---|---|---|---|---|
| LOC100129550 | Homo sapiens uncharacterized LOC100129550 (LOC100129550), non-coding RNA [NR_024618] | A_33_P3413098 | 9.02E-05 | 0.00304 | 3.105 | 1.635 |
| A_19_P00322407 | Homo sapiens uncharacterized LOC729178 (LOC729178), non-coding RNA [NR_034115] | A_19_P00322407 | 9.06E-05 | 0.00304 | 14.597 | 3.868 |
| XLOC_l2_012836 | BROAD Institute lincRNA (XLOC_l2_012836), lincRNA [TCONS_l2_00024548] | A_21_P0013052 | 9.05E-05 | 0.00304 | 3.744 | 1.905 |
| FLJ36000 | Homo sapiens uncharacterized FLJ36000 (FLJ36000), non-coding RNA [NR_027084] | A_33_P3570193 | 9.11E-05 | 0.00305 | 207.952 | 7.7 |
| FAM27L | Homo sapiens family with sequence similarity 27-like (FAM27L), non-coding RNA [NR_028336] | A_32_P942508 | 9.13E-05 | 0.00306 | 18.47 | 4.207 |
| ASB9P1 | Homo sapiens ankyrin repeat and SOCS box containing 9 pseudogene 1 (ASB9P1), non-coding RNA [NR_033769] | A_21_P0000603 | 9.23E-05 | 0.00307 | 6.929 | 2.793 |
| XLOC_008395 | BROAD Institute lincRNA (XLOC_008395), lincRNA [TCONS_00017790] | A_21_P0006655 | 9.55E-05 | 0.00312 | 168.052 | 7.393 |
| XLOC_011880 | BROAD Institute lincRNA (XLOC_011880), lincRNA [TCONS_00024587] | A_21_P0009011 | 9.62E-05 | 0.00313 | 4.982 | 2.317 |
| XLOC_l2_003674 | BROAD Institute lincRNA (XLOC_l2_003674), lincRNA [TCONS_l2_00006780] | A_21_P0011138 | 9.74E-05 | 0.00315 | 6.167 | 2.625 |
| XLOC_l2_004611 | BROAD Institute lincRNA (XLOC_l2_004611), lincRNA [TCONS_l2_00009425] | A_21_P0011401 | 9.77E-05 | 0.00316 | 3.869 | 1.952 |
| XLOC_002283 | BROAD Institute lincRNA (XLOC_002283), lincRNA [TCONS_00004409] | A_21_P0002494 | 0.000099 | 0.00317 | 9.353 | 3.225 |
| XLOC_l2_007835 | BROAD Institute lincRNA (XLOC_l2_007835), lincRNA [TCONS_l2_00015638] | A_21_P0012052 | 9.85E-05 | 0.00317 | 2.65 | 1.406 |
| LOC100509105 | PREDICTED: Homo sapiens hypothetical LOC100509105 (LOC100509105), miscRNA [XR_113070] | A_21_P0014771 | 9.91E-05 | 0.00317 | 8.368 | 3.065 |
| XLOC_010811 | BROAD Institute lincRNA (XLOC_010811), lincRNA [TCONS_00022975] | A_21_P0008511 | 0.000101 | 0.0032 | 7.283 | 2.865 |
| LOC100652935 | PREDICTED: Homo sapiens hypothetical LOC100652935 (LOC100652935), miscRNA [XR_132567] | A_21_P0014785 | 0.000102 | 0.00321 | 4.082 | 2.029 |
| ENST00000399048 | Homo sapiens keratin 16 pseudogene 2 (KRT16P2), non-coding RNA [NR_029392] | A_32_P62963 | 0.000107 | 0.00332 | 9.511 | 3.25 |
| SNORA29 | Homo sapiens small nucleolar RNA, H/ACA box 29 (SNORA29), small nucleolar RNA [NR_002965] | A_21_P0000319 | 0.000107 | 0.00332 | 2.022 | 1.016 |

| | | | | | | |
|---|---|---|---|---|---|---|
| LOC100288432 | Homo sapiens uncharacterized LOC100288432 (LOC100288432), non-coding RNA [NR_038974] | A_21_P0000856 | 0.000108 | 0.00333 | 15.078 | 3.914 |
| XLOC_l2_001421 | BROAD Institute lincRNA (XLOC_l2_001421), lincRNA [TCONS_l2_00003862] | A_21_P0010800 | 0.00011 | 0.00335 | 6.284 | 2.652 |
| A_19_P00809055 | Homo sapiens uncharacterized LOC400657 (LOC400657), non-coding RNA [NR_024484] | A_19_P00809055 | 0.000118 | 0.00349 | 2.543 | 1.346 |
| XLOC_l2_000986 | BROAD Institute lincRNA (XLOC_l2_000986), lincRNA [TCONS_l2_00001335] | A_21_P0010631 | 0.000119 | 0.00351 | 16.197 | 4.018 |
| XLOC_006314 | qf49h02.x1 Soares_testis_NHT Homo sapiens cDNA clone IMAGE:1753395 3', mRNA sequence [AI198554] | A_21_P0005271 | 0.000122 | 0.00356 | 5.15 | 2.365 |
| LOC100132618 | Homo sapiens uncharacterized LOC100132618 (LOC100132618), non-coding RNA [NR_038420] | A_21_P0000793 | 0.000122 | 0.00356 | 9.767 | 3.288 |
| XLOC_012169 | BROAD Institute lincRNA (XLOC_012169), lincRNA [TCONS_00025349] | A_21_P0009236 | 0.000124 | 0.0036 | 4.146 | 2.052 |
| XLOC_l2_008151 | BROAD Institute lincRNA (XLOC_l2_008151), lincRNA [TCONS_l2_00014727] | A_21_P0011939 | 0.000125 | 0.00362 | 2.507 | 1.326 |
| XLOC_004861 | BROAD Institute lincRNA (XLOC_004861), lincRNA [TCONS_00009689] | A_21_P0004139 | 0.000128 | 0.00367 | 34.63 | 5.114 |
| XLOC_009828 | BROAD Institute lincRNA (XLOC_009828), lincRNA [TCONS_00020511] | A_21_P0007600 | 0.000128 | 0.00367 | 2.678 | 1.421 |
| XLOC_013787 | BROAD Institute lincRNA (XLOC_013787), lincRNA [TCONS_00028062] | A_21_P0009908 | 0.000129 | 0.00368 | 13.183 | 3.721 |
| LOC100270679 | Homo sapiens uncharacterized LOC100270679 (LOC100270679), non-coding RNA [NR_038394] | A_21_P0009835 | 0.000129 | 0.00368 | 3.163 | 1.661 |
| XLOC_l2_013853 | BROAD Institute lincRNA (XLOC_l2_013853), lincRNA [TCONS_l2_00026602] | A_21_P0013312 | 0.000129 | 0.00368 | 14.039 | 3.811 |
| LOC100505692 | PREDICTED: Homo sapiens hypothetical LOC100505692 (LOC100505692), miscRNA [XR_109302] | A_21_P0014327 | 0.000129 | 0.00368 | 3.961 | 1.986 |
| LOC441268 | PREDICTED: Homo sapiens hypothetical LOC100132046 (LOC100132046), miscRNA [XR_132817] | A_33_P3415648 | 0.000134 | 0.00376 | 2.759 | 1.464 |
| XLOC_001265 | Q2AB94_MONDO (Q2AB94) Bitter taste receptor, partial (6%) [THC2684977] | A_21_P0001244 | 0.000134 | 0.00376 | 4.561 | 2.189 |
| XLOC_013275 | ALU8_HUMAN (P39195) Alu subfamily SX sequence contamination warning entry, partial (15%) [THC2497887] | A_21_P0009749 | 0.000135 | 0.00378 | 3.776 | 1.917 |

| | | | | | | |
|---|---|---|---|---|---|---|
| XLOC_l2_001947 | BROAD Institute lincRNA (XLOC_l2_001947), lincRNA [TCONS_l2_00003529] | A_21_P0010869 | 0.000136 | 0.00379 | 14.532 | 3.861 |
| XLOC_l2_012378 | Q86SS8_HUMAN (Q86SS8) Dual specificity phosphatase 8 (DUSP8 protein), partial (4%) [THC2610707] | A_21_P0013122 | 0.000138 | 0.00382 | 3.522 | 1.817 |
| XLOC_009185 | BROAD Institute lincRNA (XLOC_009185), lincRNA [TCONS_00019359] | A_21_P0007225 | 0.000142 | 0.00389 | 2.791 | 1.481 |
| XLOC_l2_012678 | BROAD Institute lincRNA (XLOC_l2_012678), lincRNA [TCONS_l2_00024370] | A_21_P0013034 | 0.000144 | 0.00392 | 16.358 | 4.032 |
| XLOC_003787 | BROAD Institute lincRNA (XLOC_003787), lincRNA [TCONS_00008338] | A_21_P0003681 | 0.000146 | 0.00396 | 27.524 | 4.783 |
| LOC100506622 | PREDICTED: Homo sapiens hypothetical LOC100506622 (LOC100506622), miscRNA [XR_110105] | A_21_P0007907 | 0.000147 | 0.00398 | 7.145 | 2.837 |
| LOC100652974 | PREDICTED: Homo sapiens superoxide dismutase [Cu-Zn]-like (LOC100652974), miscRNA [XR_132617] | A_21_P0014803 | 0.000153 | 0.00406 | 2.814 | 1.493 |
| LOC100507331 | Homo sapiens uncharacterized LOC100507331 (LOC100507331), non-coding RNA [NR_038357] | A_21_P0000778 | 0.000154 | 0.00408 | 4.323 | 2.112 |
| XLOC_003872 | BROAD Institute lincRNA (XLOC_003872), lincRNA [TCONS_00007714] | A_21_P0003446 | 0.000156 | 0.0041 | 10.705 | 3.42 |
| XLOC_l2_005714 | BROAD Institute lincRNA (XLOC_l2_005714), lincRNA [TCONS_l2_00010636] | A_21_P0011534 | 0.000159 | 0.00415 | 60.807 | 5.926 |
| XLOC_012637 | BROAD Institute lincRNA (XLOC_012637), lincRNA [TCONS_00026293] | A_21_P0009463 | 0.000161 | 0.00418 | 13.458 | 3.75 |
| XLOC_l2_012871 | BROAD Institute lincRNA (XLOC_l2_012871), lincRNA [TCONS_l2_00024609] | A_21_P0013061 | 0.000167 | 0.00427 | 11.794 | 3.56 |
| DSCR10 | Homo sapiens Down syndrome critical region gene 10 (non-protein coding) (DSCR10), non-coding RNA [NR_027695] | A_33_P3436732 | 0.000171 | 0.00434 | 5.702 | 2.511 |
| CDC14C | Homo sapiens CDC14 cell division cycle 14 homolog C (S. cerevisiae) (CDC14C), non-coding RNA [NR_003595] | A_33_P3280421 | 0.000173 | 0.00435 | 4.488 | 2.166 |
| LOC283788 | Homo sapiens FSHD region gene 1 pseudogene (LOC283788), non-coding RNA [NR_027436] | A_33_P3310371 | 0.000172 | 0.00435 | 1.812 | 0.858 |
| XLOC_008317 | BROAD Institute lincRNA (XLOC_008317), lincRNA [TCONS_00017645] | A_21_P0006605 | 0.000173 | 0.00435 | 4.01 | 2.004 |

| | | | | | | |
|---|---|---|---|---|---|---|
| A_19_P00320729 | PREDICTED: Homo sapiens hypothetical LOC100509498 (LOC100509498), miscRNA [XR_111605] | A_19_P00320729 | 0.000176 | 0.00439 | 5.966 | 2.577 |
| XLOC_004361 | BROAD Institute lincRNA (XLOC_004361), lincRNA [TCONS_00009933] | A_21_P0004244 | 0.000177 | 0.00441 | 14.218 | 3.83 |
| XLOC_l2_013145 | 602561204F1 NIH_MGC_61 Homo sapiens cDNA clone IMAGE:4699057 5', mRNA sequence [BG532141] | A_21_P0013102 | 0.00018 | 0.00446 | 2.678 | 1.421 |
| C1orf118 | PREDICTED: Homo sapiens chromosome 1 open reading frame 118 (C1orf118), miscRNA [XR_110470] | A_24_P110780 | 0.000184 | 0.00453 | 4.514 | 2.174 |
| ELP2P | Homo sapiens diazepam binding inhibitor-like 5, pseudogene (DBIL5P), non-coding RNA [NR_024120] | A_23_P351535 | 0.000185 | 0.00454 | 3.862 | 1.949 |
| LOC100128164 | Homo sapiens four and a half LIM domains 1 pseudogene (LOC100128164), transcript variant 2, non-coding RNA [NR_024409] | A_33_P3412320 | 0.000185 | 0.00454 | 2.738 | 1.453 |
| XLOC_l2_000592 | C4BP_HUMAN (P04003) C4b-binding protein alpha chain precursor (C4bp) (Proline-rich protein) (PRP), partial (20%) [THC2718370] | A_21_P0010573 | 0.000186 | 0.00455 | 11.268 | 3.494 |
| XLOC_l2_007585 | RST32057 Athersys RAGE Library Homo sapiens cDNA, mRNA sequence [BG212470] | A_21_P0012025 | 0.000187 | 0.00456 | 4.657 | 2.219 |
| XLOC_l2_010451 | BROAD Institute lincRNA (XLOC_l2_010451), lincRNA [TCONS_l2_00019632] | A_21_P0012471 | 0.000193 | 0.00465 | 7.07 | 2.822 |
| XLOC_l2_010064 | BROAD Institute lincRNA (XLOC_l2_010064), lincRNA [TCONS_l2_00019087] | A_21_P0012427 | 0.000194 | 0.00466 | 3.117 | 1.64 |
| A_19_P00322409 | Homo sapiens uncharacterized LOC729178 (LOC729178), non-coding RNA [NR_034115] | A_19_P00322409 | 0.000195 | 0.00468 | 13.155 | 3.718 |
| XLOC_l2_013734 | BROAD Institute lincRNA (XLOC_l2_013734), lincRNA [TCONS_l2_00026411] | A_21_P0013285 | 0.000202 | 0.00479 | 3.942 | 1.979 |
| XLOC_l2_014711 | BROAD Institute lincRNA (XLOC_l2_014711), lincRNA [TCONS_l2_00028630] | A_21_P0013556 | 0.000204 | 0.00481 | 3.149 | 1.655 |
| MGC39372 | Homo sapiens serpin peptidase inhibitor, clade B (ovalbumin), member 9 pseudogene (MGC39372), non-coding RNA [NR_033851] | A_32_P183918 | 0.000205 | 0.00482 | 3.932 | 1.975 |
| LOC100506860 | PREDICTED: Homo sapiens hypothetical LOC100506860, transcript variant 2 (LOC100506860), miscRNA [XR_108814] | A_21_P0014201 | 0.000209 | 0.00487 | 7.064 | 2.82 |

| | | | | | | |
|---|---|---|---|---|---|---|
| XLOC_005152 | BROAD Institute lincRNA (XLOC_005152), lincRNA [TCONS_00011260] | A_21_P0004629 | 0.00022 | 0.00502 | 4.095 | 2.034 |
| LOC100506317 | PREDICTED: Homo sapiens hypothetical LOC100506317 (LOC100506317), miscRNA [XR_108389] | A_21_P0014086 | 0.00023 | 0.00515 | 12.523 | 3.646 |
| XLOC_001497 | DA767045 NTONG2 Homo sapiens cDNA clone NTONG2008452 5', mRNA sequence [DA767045] | A_21_P0001865 | 0.000231 | 0.00515 | 5.96 | 2.575 |
| LOC100506235 | PREDICTED: Homo sapiens hypothetical LOC100506235, transcript variant 2 (LOC100506235), miscRNA [XR_109987] | A_21_P0002212 | 0.000231 | 0.00515 | 4.236 | 2.083 |
| A_33_P3356517 | PREDICTED: Homo sapiens hypothetical LOC100652764 (LOC100652764), miscRNA [XR_132698] | A_33_P3356517 | 0.000233 | 0.00517 | 3.098 | 1.631 |
| A_19_P00322276 | Homo sapiens uncharacterized LOC285419 (LOC285419), transcript variant 1, non-coding RNA [NR_027105] | A_19_P00322276 | 0.000235 | 0.0052 | 4.445 | 2.152 |
| XLOC_l2_001890 | BROAD Institute lincRNA (XLOC_l2_001890), lincRNA [TCONS_l2_00003443] | A_21_P0010864 | 0.000239 | 0.00524 | 4.017 | 2.006 |
| XLOC_l2_014830 | BROAD Institute lincRNA (XLOC_l2_014830), lincRNA [TCONS_l2_00028797] | A_21_P0013580 | 0.000247 | 0.00534 | 2.222 | 1.152 |
| XLOC_005423 | BROAD Institute lincRNA (XLOC_005423), lincRNA [TCONS_00011354] | A_21_P0004678 | 0.000252 | 0.00542 | 7.009 | 2.809 |
| XLOC_008337 | BROAD Institute lincRNA (XLOC_008337), lincRNA [TCONS_00018082] | A_21_P0006808 | 0.000254 | 0.00545 | 9.591 | 3.262 |
| C6orf41 | Homo sapiens long intergenic non-protein coding RNA 240 (LINC00240), non-coding RNA [NR_026775] | A_33_P3220192 | 0.000256 | 0.00547 | 10.513 | 3.394 |
| XLOC_005209 | BROAD Institute lincRNA (XLOC_005209), lincRNA [TCONS_00012439] | A_21_P0004857 | 0.000262 | 0.00555 | 7.831 | 2.969 |
| XLOC_l2_013506 | BROAD Institute lincRNA (XLOC_l2_013506), lincRNA [TCONS_l2_00026092] | A_21_P0013254 | 0.000268 | 0.00562 | 8.868 | 3.149 |
| XLOC_010219 | BROAD Institute lincRNA (XLOC_010219), lincRNA [TCONS_00020953] | A_21_P0007774 | 0.000277 | 0.00572 | 19.878 | 4.313 |
| LINC00240 | Homo sapiens long intergenic non-protein coding RNA 240 (LINC00240), non-coding RNA [NR_026775] | A_21_P0004866 | 0.000276 | 0.00572 | 2.243 | 1.165 |

| | | | | | | |
|---|---|---|---|---|---|---|
| XLOC_006844 | nq53h01.s1 NCI_CGAP_Co10 Homo sapiens cDNA clone IMAGE:1147633 3', mRNA sequence [AA613028] | A_21_P0005721 | 0.00028 | 0.00576 | 3.341 | 1.74 |
| A_19_P00321183 | PREDICTED: Homo sapiens hypothetical LOC100506392 (LOC100506392), miscRNA [XR_110490] | A_19_P00321183 | 0.000286 | 0.00584 | 3.082 | 1.624 |
| XLOC_l2_013513 | ZN695_HUMAN (Q8IW36) Zinc finger protein 695 (Zinc finger protein SBZF3), partial (8%) [THC2641926] | A_21_P0013393 | 0.000286 | 0.00584 | 44.462 | 5.475 |
| LOC643699 | BROAD Institute lincRNA (XLOC_l2_004602), lincRNA [TCONS_l2_00008528] | A_21_P0011307 | 0.000292 | 0.00593 | 3.927 | 1.973 |
| KIAA1908 | Homo sapiens uncharacterized LOC114796 (KIAA1908), transcript variant 1, non-coding RNA [NR_027329] | A_23_P123086 | 0.000294 | 0.00595 | 2.251 | 1.171 |
| LOC100616530 | Homo sapiens tospeak (LOC100616530), transcript variant 8, non-coding RNA [NR_038208] | A_21_P0000723 | 0.000307 | 0.0061 | 2.595 | 1.376 |
| XLOC_l2_013931 | BT007113 asparagine synthetase {Homo sapiens} (exp=-1; wgp=0; cg=0), complete [THC2502767] | A_21_P0013338 | 0.000308 | 0.00612 | 2.441 | 1.288 |
| RP11-251J8.3 | Homo sapiens SPG20 opposite strand (SPG20OS), transcript variant 1, non-coding RNA [NR_045180] | A_23_P316085 | 0.000317 | 0.00623 | 4.202 | 2.071 |
| SNORA8 | Homo sapiens small nucleolar RNA, H/ACA box 8 (SNORA8), small nucleolar RNA [NR_002920] | A_21_P0000302 | 0.000319 | 0.00625 | 2.065 | 1.046 |
| LOC148824 | Homo sapiens uncharacterized LOC148824 (LOC148824), non-coding RNA [NR_027309] | A_21_P0000531 | 0.000319 | 0.00625 | 4.252 | 2.088 |
| MGC16121 | Homo sapiens uncharacterized protein MGC16121 (MGC16121), non-coding RNA [NR_024607] | A_33_P3402329 | 0.000323 | 0.00629 | 3.155 | 1.657 |
| XLOC_l2_000010 | Q6FTS4_CANGA (Q6FTS4) Similarity, partial (15%) [THC2660300] | A_21_P0010686 | 0.000327 | 0.00635 | 7.999 | 3 |
| XLOC_l2_009811 | ALU8_HUMAN (P39195) Alu subfamily SX sequence contamination warning entry, partial (13%) [THC2533436] | A_21_P0012386 | 0.00034 | 0.00651 | 9.504 | 3.249 |
| XLOC_l2_014602 | BROAD Institute lincRNA (XLOC_l2_014602), lincRNA [TCONS_l2_00028340] | A_21_P0013527 | 0.000342 | 0.00652 | 3.065 | 1.616 |

| | | | | | | |
|---|---|---|---|---|---|---|
| XLOC_007952 | BROAD Institute lincRNA (XLOC_007952), lincRNA [TCONS_00017415] | A_21_P0006559 | 0.000342 | 0.00652 | 4.181 | 2.064 |
| LOC100506548 | Homo sapiens uncharacterized LOC100506548 (LOC100506548), non-coding RNA [NR_037665] | A_21_P0000684 | 0.000342 | 0.00652 | 2.222 | 1.152 |
| LOC256880 | Homo sapiens uncharacterized LOC256880 (LOC256880), non-coding RNA [NR_002799] | A_33_P3294583 | 0.000343 | 0.00653 | 4.09 | 2.032 |
| LOC339240 | Homo sapiens keratin 42 pseudogene (KRT42P), non-coding RNA [NR_033415] | A_33_P3857239 | 0.000352 | 0.00663 | 14.983 | 3.905 |
| XLOC_l2_004371 | BROAD Institute lincRNA (XLOC_l2_004371), lincRNA [TCONS_l2_00008016] | A_21_P0011263 | 0.000354 | 0.00665 | 2.515 | 1.331 |
| LOC731789 | Homo sapiens uncharacterized LOC731789 (LOC731789), non-coding RNA [NR_026794] | A_32_P56397 | 0.000358 | 0.00669 | 3.096 | 1.63 |
| BC039455 | PREDICTED: Homo sapiens hypothetical LOC728769 (LOC728769), miscRNA [XR_108586] | A_33_P3781394 | 0.000358 | 0.00669 | 2.1 | 1.071 |
| LOC653390 | Homo sapiens RNA polymerase I transcription factor homolog (S. cerevisiae) pseudogene 2 (RRN3P2), non-coding RNA [NR_003369] | A_24_P475864 | 0.00036 | 0.00672 | 3.869 | 1.952 |
| XLOC_l2_008405 | AGENCOURT_10443904 NIH_MGC_82 Homo sapiens cDNA clone IMAGE:6619894 5', mRNA sequence [BU853167] | A_21_P0012102 | 0.000365 | 0.00679 | 3.599 | 1.847 |
| A_19_P00322310 | Homo sapiens uncharacterized LOC100505702 (LOC100505702), transcript variant 1, non-coding RNA [NR_038303] | A_19_P00322310 | 0.000366 | 0.0068 | 6.474 | 2.695 |
| A_19_P00325810 | Homo sapiens chromosome 6 open reading frame 147 (C6orf147), non-coding RNA [NR_027005] | A_19_P00325810 | 0.000367 | 0.0068 | 2.517 | 1.331 |
| ANXA2P1 | Homo sapiens annexin A2 pseudogene 1 (ANXA2P1), non-coding RNA [NR_001562] | A_24_P204244 | 0.00037 | 0.00684 | 3.756 | 1.909 |
| XLOC_000527 | PREDICTED: Homo sapiens hypothetical LOC100506696, transcript variant 2 (LOC100506696), miscRNA [XR_108330] | A_21_P0001050 | 0.000374 | 0.00688 | 2.676 | 1.42 |
| LOC100505971 | PREDICTED: Homo sapiens hypothetical LOC100505971 (LOC100505971), miscRNA [XR_110832] | A_21_P0014631 | 0.000374 | 0.00688 | 3.196 | 1.676 |
| XLOC_012467 | BROAD Institute lincRNA (XLOC_012467), lincRNA [TCONS_00025631] | A_21_P0009323 | 0.000376 | 0.00691 | 3.259 | 1.704 |

| | | | | | | |
|---|---|---|---|---|---|---|
| LOC100505963 | PREDICTED: Homo sapiens hypothetical LOC100505963 (LOC100505963), miscRNA [XR_109616] | A_21_P0014402 | 0.000377 | 0.00692 | 4.613 | 2.206 |
| A_19_P00323375 | Homo sapiens TTC28 antisense RNA 1 (non-protein coding) (TTC28-AS1), transcript variant 2, non-coding RNA [NR_026962] | A_19_P00323375 | 0.000386 | 0.00702 | 4.432 | 2.148 |
| XLOC_001286 | BROAD Institute lincRNA (XLOC_001286), lincRNA [TCONS_00001898] | A_21_P0001652 | 0.000386 | 0.00702 | 6.97 | 2.801 |
| A_21_P0014633 | PREDICTED: Homo sapiens hypothetical LOC100505824 (LOC100505824), miscRNA [XR_110827] | A_21_P0014633 | 0.000392 | 0.00708 | 2.167 | 1.116 |
| XLOC_002478 | BROAD Institute lincRNA (XLOC_002478), lincRNA [TCONS_00005330] | A_21_P0002539 | 0.000397 | 0.00714 | 3.99 | 1.996 |
| LOC100505978 | Homo sapiens uncharacterized LOC100505978 (LOC100505978), non-coding RNA [NR_038912] | A_21_P0000836 | 0.000397 | 0.00714 | 2.801 | 1.486 |
| A_19_P00324470 | Homo sapiens MIR143 host gene (non-protein coding) (MIR143HG), non-coding RNA [NR_027180] | A_19_P00324470 | 0.0004 | 0.00717 | 2.082 | 1.058 |
| ENST00000442512 | Homo sapiens carboxylesterase 1 pseudogene 2 (CES1P2), non-coding RNA [NR_033740] | A_33_P3283964 | 0.000402 | 0.0072 | 17.082 | 4.094 |
| XLOC_007370 | Unknown | A_21_P0006012 | 0.000403 | 0.00721 | 7.624 | 2.931 |
| XLOC_004144 | BROAD Institute lincRNA (XLOC_004144), lincRNA [TCONS_00008639] | A_21_P0003792 | 0.000422 | 0.00743 | 2.242 | 1.165 |
| XLOC_004832 | BROAD Institute lincRNA (XLOC_004832), lincRNA [TCONS_00010346] | A_21_P0004396 | 0.000427 | 0.00749 | 2.919 | 1.546 |
| XLOC_004063 | BROAD Institute lincRNA (XLOC_004063), lincRNA [TCONS_00007820] | A_21_P0003499 | 0.000428 | 0.0075 | 2.908 | 1.54 |
| XLOC_006390 | Q3KII3_PSEPF (Q3KII3) Inner-membrane translocator, partial (6%) [THC2773766] | A_21_P0005486 | 0.000428 | 0.0075 | 19.051 | 4.252 |
| A_19_P00322898 | Homo sapiens carbonic anhydrase VB pseudogene 1 (CA5BP1), non-coding RNA [NR_026551] | A_19_P00322898 | 0.000434 | 0.00756 | 2.649 | 1.406 |
| A_19_P00805212 | Homo sapiens chromosome 6 open reading frame 147 (C6orf147), non-coding RNA [NR_027005] | A_19_P00805212 | 0.000441 | 0.00763 | 2.707 | 1.437 |
| LOC646214 | Homo sapiens p21 protein (Cdc42/Rac)-activated kinase 2 pseudogene (LOC646214), non-coding RNA [NR_027053] | A_33_P3378707 | 0.000464 | 0.0079 | 4.269 | 2.094 |

| | | | | | | |
|---|---|---|---|---|---|---|
| LOC100506408 | PREDICTED: Homo sapiens hypothetical LOC100506408 (LOC100506408), miscRNA [XR_110210] | A_21_P0014525 | 0.000465 | 0.00791 | 13.828 | 3.789 |
| LOC100507128 | PREDICTED: Homo sapiens hypothetical LOC100507128 (LOC100507128), miscRNA [XR_109038] | A_21_P0014261 | 0.000467 | 0.00792 | 3.504 | 1.809 |
| XLOC_005217 | BROAD Institute lincRNA (XLOC_005217), lincRNA [TCONS_00011755] | A_21_P0004867 | 0.000469 | 0.00793 | 3.396 | 1.764 |
| A_19_P00320986 | PREDICTED: Homo sapiens hypothetical LOC100506661 (LOC100506661), miscRNA [XR_110931] | A_19_P00320986 | 0.000506 | 0.00831 | 2.672 | 1.418 |
| A_19_P00321391 | Homo sapiens uncharacterized LOC100422737 (LOC100422737), non-coding RNA [NR_033557] | A_19_P00321391 | 0.000514 | 0.00839 | 14.732 | 3.881 |
| A_19_P00320384 | PREDICTED: Homo sapiens hypothetical LOC100506661 (LOC100506661), miscRNA [XR_110931] | A_19_P00320384 | 0.000516 | 0.00841 | 2.02 | 1.015 |
| XLOC_l2_009456 | GGT1_HUMAN Gamma-glutamyltranspeptidase 1 precursor (Gamma-glutamyltransferase 1) (CD224 antigen) [Contains: Gamma-glutamyltranspeptidase 1 heavy chain; Gamma-glutamyltranspeptidase 1light chain]. {Homo sapiens} (exp=-1; wgp=-1; cg=-1) , complete [THC2483904] | A_21_P0012281 | 0.000517 | 0.00842 | 4.544 | 2.184 |
| XLOC_004568 | BROAD Institute lincRNA (XLOC_004568), lincRNA [TCONS_00009523] | A_21_P0004066 | 0.00052 | 0.00846 | 2.309 | 1.207 |
| ENST00000361350 | chromosome 1 open reading frame 191 [Source:HGNC Symbol;Acc:32328] [ENST00000361350] | A_33_P3216928 | 0.000526 | 0.00851 | 2.846 | 1.509 |
| XLOC_001125 | BROAD Institute lincRNA (XLOC_001125), lincRNA [TCONS_00001757] | A_21_P0001601 | 0.000546 | 0.00874 | 5.267 | 2.397 |
| LOC100506229 | Homo sapiens uncharacterized LOC100506229 (LOC100506229), transcript variant 1, non-coding RNA [NR_039975] | A_21_P0000869 | 0.000548 | 0.00876 | 3.654 | 1.869 |
| XLOC_004053 | BROAD Institute lincRNA (XLOC_004053), lincRNA [TCONS_00008555] | A_21_P0003759 | 0.000549 | 0.00877 | 14.68 | 3.876 |
| XLOC_005419 | BROAD Institute lincRNA (XLOC_005419), lincRNA [TCONS_00012566] | A_21_P0005127 | 0.000556 | 0.00882 | 10.791 | 3.432 |

| | | | | | | |
|---|---|---|---|---|---|---|
| XLOC_011586 | BROAD Institute lincRNA (XLOC_011586), lincRNA [TCONS_00023793] | A_21_P0008784 | 0.000567 | 0.00895 | 7.456 | 2.898 |
| XLOC_006555 | BROAD Institute lincRNA (XLOC_006555), lincRNA [TCONS_00013221] | A_21_P0005322 | 0.000572 | 0.00899 | 8.079 | 3.014 |
| XLOC_004600 | BROAD Institute lincRNA (XLOC_004600), lincRNA [TCONS_00010139] | A_21_P0004313 | 0.000578 | 0.00906 | 6.974 | 2.802 |
| XLOC_006887 | BROAD Institute lincRNA (XLOC_006887), lincRNA [TCONS_00014811] | A_21_P0005744 | 0.000598 | 0.00923 | 3.449 | 1.786 |
| LOC284900 | Homo sapiens TTC28 antisense RNA 1 (non-protein coding) (TTC28-AS1), transcript variant 2, non-coding RNA [NR_026962] | A_33_P3362143 | 0.000601 | 0.00927 | 4.16 | 2.057 |
| LOC100131089 | Homo sapiens uncharacterized LOC100131089 (LOC100131089), transcript variant 1, non-coding RNA [NR_040059] | A_33_P3356356 | 0.000603 | 0.00928 | 1.977 | 0.984 |
| A_19_P00321014 | PREDICTED: Homo sapiens hypothetical LOC100506661 (LOC100506661), miscRNA [XR_110931] | A_19_P00321014 | 0.000602 | 0.00928 | 2.763 | 1.466 |
| XLOC_009895 | BROAD Institute lincRNA (XLOC_009895), lincRNA [TCONS_00020579] | A_21_P0007635 | 0.00061 | 0.00934 | 5.648 | 2.498 |
| XLOC_010859 | Q21RF1_9BURK (Q21RF1) Biotin--acetyl-CoA-carboxylase ligase, partial (6%) [THC2728144] | A_21_P0008352 | 0.000609 | 0.00934 | 7.031 | 2.814 |
| LOC100132317 | Homo sapiens long intergenic non-protein coding RNA 266-1 (LINC00266-1), non-coding RNA [NR_040415] | A_23_P355776 | 0.000621 | 0.00944 | 2.791 | 1.481 |
| LOC100133612 | Homo sapiens uncharacterized LOC100133612 (LOC100133612), non-coding RNA [NR_024455] | A_21_P0010509 | 0.000631 | 0.00953 | 2.349 | 1.232 |
| XLOC_l2_006983 | Unknown | A_21_P0011732 | 0.00064 | 0.00962 | 3.584 | 1.842 |
| A_19_P00319625 | AB003177 proteasome subunit p27 {Homo sapiens} (exp=-1; wgp=0; cg=0), partial (9%) [THC2612751] | A_19_P00319625 | 0.00065 | 0.00972 | 7.711 | 2.947 |
| XLOC_l2_012847 | BROAD Institute lincRNA (XLOC_l2_012847), lincRNA [TCONS_l2_00024571] | A_21_P0013057 | 0.000651 | 0.00973 | 19.026 | 4.25 |
| XLOC_001744 | BROAD Institute lincRNA (XLOC_001744), lincRNA [TCONS_00003045] | A_21_P0001934 | 0.000652 | 0.00974 | 5.402 | 2.433 |
| XLOC_002616 | O87140_VIBCH (O87140) WbfS protein, partial (5%) [THC2668179] | A_21_P0002944 | 0.000653 | 0.00975 | 3.085 | 1.625 |

| | | | | | | |
|---|---|---|---|---|---|---|
| XLOC_003228 | Q4RES2_TETNG (Q4RES2) Chromosome 13 SCAF15122, whole genome shotgun sequence, partial (10%) [THC2639066] | A_21_P0003297 | 0.000663 | 0.00984 | 2.792 | 1.481 |
| LOC653075 | Homo sapiens golgin A8 family, member A pseudogene (LOC653075), non-coding RNA [NR_033933] | A_21_P0011355 | 0.000686 | 0.0101 | 3.702 | 1.888 |
| XLOC_007094 | BROAD Institute lincRNA (XLOC_007094), lincRNA [TCONS_00015020] | A_21_P0005841 | 0.000698 | 0.0102 | 5.399 | 2.433 |
| XLOC_l2_004072 | BROAD Institute lincRNA (XLOC_l2_004072), lincRNA [TCONS_l2_00007295] | A_21_P0011192 | 0.000711 | 0.0103 | 7.461 | 2.899 |
| LOC100287598 | PREDICTED: Homo sapiens hypothetical LOC100287598 (LOC100287598), miscRNA [XR_109252] | A_21_P0014316 | 0.000711 | 0.0103 | 3.061 | 1.614 |
| XLOC_008133 | 601273442F1 NIH_MGC_20 Homo sapiens cDNA clone IMAGE:3614530 5', mRNA sequence [BE386236] | A_21_P0006445 | 0.000712 | 0.0103 | 2.204 | 1.14 |
| XLOC_010031 | BROAD Institute lincRNA (XLOC_010031), lincRNA [TCONS_00020732] | A_21_P0007693 | 0.000739 | 0.0105 | 3.018 | 1.594 |
| LIMS3 | Homo sapiens LIM and senescent cell antigen-like domains 3-like (LIMS3L), transcript variant 2, non-coding RNA [NR_038099] | A_33_P3262854 | 0.000748 | 0.0106 | 2.474 | 1.307 |
| A_19_P00318977 | AB003177 proteasome subunit p27 {Homo sapiens} (exp=-1; wgp=0; cg=0), partial (9%) [THC2612751] | A_19_P00318977 | 0.000773 | 0.0108 | 8.401 | 3.07 |
| XLOC_003364 | BROAD Institute lincRNA (XLOC_003364), lincRNA [TCONS_00006729] | A_21_P0003199 | 0.000765 | 0.0108 | 9.053 | 3.178 |
| LOC400684 | Homo sapiens hypothetical gene supported by BC000922, mRNA (cDNA clone IMAGE:3447073), partial cds. [BC000922] | A_33_P3640101 | 0.000767 | 0.0108 | 1.919 | 0.94 |
| DPY19L1P1 | Homo sapiens dpy-19-like 1 pseudogene 1 (C. elegans) (DPY19L1P1), non-coding RNA [NR_036680] | A_24_P453921 | 0.000777 | 0.0109 | 3.112 | 1.638 |
| FLJ23867 | Homo sapiens uncharacterized protein FLJ23867 (FLJ23867), non-coding RNA [NR_026900] | A_33_P3220475 | 0.000784 | 0.0109 | 2.412 | 1.27 |
| XLOC_010825 | BROAD Institute lincRNA (XLOC_010825), lincRNA [TCONS_00022499] | A_21_P0008333 | 0.000776 | 0.0109 | 8.936 | 3.16 |
| XLOC_007722 | BROAD Institute lincRNA (XLOC_007722), lincRNA [TCONS_00015828] | A_21_P0006079 | 0.000778 | 0.0109 | 2.765 | 1.467 |

| | | | | | | |
|---|---|---|---|---|---|---|
| FLJ39582 | Homo sapiens THAP7 antisense RNA 1 (non-protein coding) (THAP7-AS1), transcript variant 2, non-coding RNA [NR_027052] | A_23_P404094 | 0.000797 | 0.011 | 2.016 | 1.012 |
| XLOC_005593 | BROAD Institute lincRNA (XLOC_005593), lincRNA [TCONS_00011440] | A_21_P0004723 | 0.000798 | 0.0111 | 3.748 | 1.906 |
| XLOC_001775 | BROAD Institute lincRNA (XLOC_001775), lincRNA [TCONS_00004960] | A_19_P00323103 | 0.000819 | 0.0112 | 2.205 | 1.141 |
| XLOC_009457 | BROAD Institute lincRNA (XLOC_009457), lincRNA [TCONS_00019652] | A_21_P0007359 | 0.000816 | 0.0112 | 6.613 | 2.725 |
| A_19_P00326865 | Homo sapiens chromosome 6 open reading frame 147 (C6orf147), non-coding RNA [NR_027005] | A_19_P00326865 | 0.000826 | 0.0113 | 2.558 | 1.355 |
| LOC400657 | Homo sapiens uncharacterized LOC400657 (LOC400657), non-coding RNA [NR_024484] | A_33_P3383816 | 0.000852 | 0.0115 | 3.544 | 1.825 |
| LOC100506024 | PREDICTED: Homo sapiens hypothetical LOC100506024 (LOC100506024), miscRNA [XR_108553] | A_21_P0014136 | 0.000853 | 0.0115 | 7.498 | 2.907 |
| LOC100505636 | PREDICTED: Homo sapiens hypothetical LOC100505636, transcript variant 1 (LOC100505636), miscRNA [XR_110309] | A_21_P0014562 | 0.000862 | 0.0116 | 2.593 | 1.374 |
| XLOC_012632 | BROAD Institute lincRNA (XLOC_012632), lincRNA [TCONS_00026290] | A_21_P0009460 | 0.000856 | 0.0116 | 9.505 | 3.249 |
| DPY19L2P3 | Homo sapiens dpy-19-like 2 pseudogene 3 (C. elegans) (DPY19L2P3), transcript variant 1, non-coding RNA [NR_036554] | A_33_P3626301 | 0.000873 | 0.0117 | 6.778 | 2.761 |
| XLOC_001496 | BROAD Institute lincRNA (XLOC_001496), lincRNA [TCONS_00004804] | A_21_P0002596 | 0.000872 | 0.0117 | 5.287 | 2.402 |
| LOC401068 | PREDICTED: Homo sapiens hypothetical LOC401068 (LOC401068), miscRNA [XR_110024] | A_21_P0014480 | 0.000889 | 0.0118 | 2.437 | 1.285 |
| XLOC_006195 | Q6NT14_HUMAN (Q6NT14) ZNF80 protein (Fragment), partial (11%) [THC2703001] | A_21_P0005196 | 0.000885 | 0.0118 | 4.544 | 2.184 |
| SNORD114-9 | Homo sapiens small nucleolar RNA, C/D box 114-9 (SNORD114-9), small nucleolar RNA [NR_003201] | A_21_P0000406 | 0.000901 | 0.0119 | 2.963 | 1.567 |
| A_19_P00320664 | Homo sapiens uncharacterized LOC441172 (FLJ46906), non-coding RNA [NR_033896] | A_19_P00320664 | 0.000913 | 0.012 | 4.469 | 2.16 |

| | | | | | | |
|---|---|---|---|---|---|---|
| ENST00000338857 | PREDICTED: Homo sapiens golgin-like (LOC441728), miscRNA [XR_109175] | A_33_P3259443 | 0.000946 | 0.0123 | 7.751 | 2.954 |
| XLOC_l2_011151 | Q5TF36_HUMAN (Q5TF36) REV3-like, catalytic subunit of DNA polymerase zeta (Yeast), partial (20%) [THC2610434] | A_21_P0012669 | 0.000957 | 0.0124 | 2.59 | 1.373 |
| RPL23AP13 | Homo sapiens ribosomal protein L23a pseudogene 32 (RPL23AP32), non-coding RNA [NR_002229] | A_23_P108932 | 0.000973 | 0.0125 | 5.863 | 2.552 |
| ENST00000397186 | Homo sapiens uncharacterized LOC645249 (LOC645249), non-coding RNA [NR_038835] | A_32_P185317 | 0.00097 | 0.0125 | 2.236 | 1.161 |
| XLOC_002643 | PREDICTED: Homo sapiens hypothetical LOC100506637 (LOC100506637), miscRNA [XR_110001] | A_21_P0002702 | 0.000984 | 0.0126 | 2.042 | 1.03 |
| LOC644135 | PREDICTED: Homo sapiens hypothetical LOC644135 (LOC644135), miscRNA [XR_112910] | A_33_P3378962 | 0.001 | 0.0127 | 3.985 | 1.995 |
| LOC374443 | Homo sapiens CLR pseudogene (LOC374443), non-coding RNA [NR_002814] | A_21_P0011036 | 0.00102 | 0.0129 | 2.6 | 1.378 |
| BREA2 | Homo sapiens breast cancer estrogen-induced apoptosis 2 (BREA2), non-coding RNA [NR_015445] | A_33_P3260500 | 0.00104 | 0.0131 | 3.844 | 1.943 |
| LOC100507495 | Homo sapiens uncharacterized LOC100507495 (LOC100507495), transcript variant 1, non-coding RNA [NR_040047] | A_21_P0000891 | 0.00106 | 0.0133 | 3.135 | 1.648 |
| LOC100506580 | PREDICTED: Homo sapiens hypothetical LOC100506580 (LOC100506580), miscRNA [XR_109204] | A_21_P0014296 | 0.00107 | 0.0133 | 7.812 | 2.966 |
| LOC389517 | Homo sapiens speedy homolog E8 (Xenopus laevis), pseudogene (SPDYE8P), non-coding RNA [NR_003664] | A_32_P202182 | 0.00109 | 0.0134 | 3.562 | 1.833 |
| hCG_1981531 | PREDICTED: Homo sapiens hCG1981531 (LOC728586), miscRNA [XR_110564] | A_24_P32627 | 0.00108 | 0.0134 | 18.246 | 4.189 |
| GOLGA8IP | Homo sapiens golgin A8 family, member I, pseudogene (GOLGA8IP), non-coding RNA [NR_024074] | A_21_P0011362 | 0.00113 | 0.0137 | 3.522 | 1.816 |
| LOC100507245 | PREDICTED: Homo sapiens hypothetical LOC100507245 (LOC100507245), miscRNA [XR_109043] | A_21_P0014264 | 0.00114 | 0.0138 | 3.795 | 1.924 |

| | | | | | | |
|---|---|---|---|---|---|---|
| FLJ38723 | PREDICTED: Homo sapiens hypothetical FLJ38723 (FLJ38723), miscRNA [XR_132593] | A_33_P3311845 | 0.00115 | 0.0139 | 5.922 | 2.566 |
| XLOC_005082 | BROAD Institute lincRNA (XLOC_005082), lincRNA [TCONS_00010555] | A_21_P0004469 | 0.00118 | 0.0141 | 4.42 | 2.144 |
| XLOC_008256 | BROAD Institute lincRNA (XLOC_008256), lincRNA [TCONS_00017386] | A_21_P0006553 | 0.0012 | 0.0142 | 2.604 | 1.38 |
| XLOC_l2_009140 | PREDICTED: Homo sapiens hypothetical protein LOC100652984 (LOC100652984), mRNA [XM_003403484] | A_21_P0012224 | 0.00119 | 0.0142 | 42.71 | 5.416 |
| LOC645431 | Homo sapiens uncharacterized LOC645431 (LOC645431), non-coding RNA [NR_024334] | A_23_P327156 | 0.00121 | 0.0143 | 8.372 | 3.066 |
| XLOC_006010 | BROAD Institute lincRNA (XLOC_006010), lincRNA [TCONS_00013378] | A_21_P0005361 | 0.00121 | 0.0143 | 5.588 | 2.482 |
| FLJ27354 | Homo sapiens uncharacterized LOC400761 (FLJ27354), non-coding RNA [NR_033981] | A_21_P0000908 | 0.00121 | 0.0143 | 3.009 | 1.589 |
| AK310300 | Homo sapiens uncharacterized LOC100506136 (LOC100506136), non-coding RNA [NR_038948] | A_33_P3390092 | 0.00123 | 0.0144 | 2.557 | 1.354 |
| XLOC_004342 | BX104209 NCI_CGAP_GC4 Homo sapiens cDNA clone IMAGp998A013735, mRNA sequence [BX104209] | A_21_P0003987 | 0.00122 | 0.0144 | 7.055 | 2.819 |
| XLOC_004229 | BROAD Institute lincRNA (XLOC_004229), lincRNA [TCONS_00008716] | A_21_P0003821 | 0.00123 | 0.0144 | 2.342 | 1.228 |
| XLOC_000550 | Unknown | A_21_P0001057 | 0.00123 | 0.0144 | 4.62 | 2.208 |
| LOC285084 | Homo sapiens uncharacterized LOC285084 (LOC285084), non-coding RNA [NR_038897] | A_21_P0001938 | 0.00125 | 0.0146 | 5.029 | 2.33 |
| XLOC_004725 | BROAD Institute lincRNA (XLOC_004725), lincRNA [TCONS_00010259] | A_21_P0004359 | 0.00127 | 0.0147 | 3.59 | 1.844 |
| A_19_P00321998 | AB003177 proteasome subunit p27 {Homo sapiens} (exp=-1; wgp=0; cg=0), partial (9%) [THC2612751] | A_19_P00321998 | 0.00129 | 0.0149 | 9.961 | 3.316 |
| LOC100507633 | PREDICTED: Homo sapiens hypothetical LOC100507633 (LOC100507633), miscRNA [XR_110437] | A_21_P0006781 | 0.00131 | 0.015 | 5.088 | 2.347 |
| XLOC_l2_000401 | BROAD Institute lincRNA (XLOC_l2_000401), lincRNA [TCONS_l2_00002174] | A_21_P0010722 | 0.00132 | 0.0151 | 4.114 | 2.04 |
| LOC100505592 | PREDICTED: Homo sapiens hypothetical LOC100505592 (LOC100505592), miscRNA [XR_109463] | A_21_P0009442 | 0.00132 | 0.0151 | 21.809 | 4.447 |

| | | | | | | |
|---|---|---|---|---|---|---|
| LOC100506910 | PREDICTED: Homo sapiens hypothetical LOC100506910 (LOC100506910), miscRNA [XR_108878] | A_21_P0014212 | 0.00135 | 0.0153 | 2.905 | 1.539 |
| XLOC_006283 | BROAD Institute lincRNA (XLOC_006283), lincRNA [TCONS_00014228] | A_21_P0005582 | 0.00136 | 0.0154 | 4.887 | 2.289 |
| LOC100506124 | Homo sapiens uncharacterized LOC100506124 (LOC100506124), non-coding RNA [NR_045375] | A_21_P0014934 | 0.00136 | 0.0154 | 3.048 | 1.608 |
| LOC100133039 | PREDICTED: Homo sapiens hypothetical LOC100133039 (LOC100133039), miscRNA [XR_110031] | A_33_P3291614 | 0.00138 | 0.0155 | 4.383 | 2.132 |
| A_19_P00317431 | Homo sapiens uncharacterized LOC285375 (LOC285375), non-coding RNA [NR_027103] | A_19_P00317431 | 0.00139 | 0.0156 | 4.288 | 2.1 |
| XLOC_010477 | LAGE3_HUMAN (Q14657) L antigen family member 3 (ITBA2 protein) (ESO-3 protein), partial (10%) [THC2621524] | A_21_P0007960 | 0.00139 | 0.0156 | 1.474 | 0.56 |
| XLOC_l2_003419 | BROAD Institute lincRNA (XLOC_l2_003419), lincRNA [TCONS_l2_00006314] | A_21_P0011103 | 0.00141 | 0.0157 | 2.2 | 1.137 |
| XLOC_002749 | BROAD Institute lincRNA (XLOC_002749), lincRNA [TCONS_00006148] | A_19_P00809368 | 0.00143 | 0.0158 | 4.152 | 2.054 |
| A_19_P00319537 | Homo sapiens uncharacterized LOC285419 (LOC285419), transcript variant 1, non-coding RNA [NR_027105] | A_19_P00319537 | 0.00142 | 0.0158 | 6.319 | 2.66 |
| LOC400680 | Homo sapiens uncharacterized LOC400680 (LOC400680), non-coding RNA [NR_037194] | A_21_P0000677 | 0.00143 | 0.0158 | 2.858 | 1.515 |
| XLOC_l2_010061 | BX111391 Soares_testis_NHT Homo sapiens cDNA clone IMAGp998D244108, mRNA sequence [BX111391] | A_21_P0012425 | 0.00143 | 0.0158 | 3.076 | 1.621 |
| XLOC_003805 | BROAD Institute lincRNA (XLOC_003805), lincRNA [TCONS_00008950] | A_21_P0003867 | 0.00144 | 0.0159 | 4.847 | 2.277 |
| XLOC_l2_001496 | BROAD Institute lincRNA (XLOC_l2_001496), lincRNA [TCONS_l2_00002904] | A_21_P0010806 | 0.00145 | 0.016 | 4.094 | 2.033 |
| XLOC_013028 | Q7Z6P5_HUMAN (Q7Z6P5) MCM3 minichromosome maintenance deficient 3 (S. cerevisiae) (Fragment), partial (9%) [THC2614689] | A_21_P0009702 | 0.00149 | 0.0162 | 2.492 | 1.317 |
| FLJ31104 | PREDICTED: Homo sapiens hypothetical LOC441072 (FLJ31104), miscRNA [XR_108600] | A_33_P3695899 | 0.00151 | 0.0163 | 3.176 | 1.667 |

| | | | | | | |
|---|---|---|---|---|---|---|
| RP11-165H20.1 | Homo sapiens CHIA-like pseudogene (RP11-165H20.1), non-coding RNA [NR_003928] | A_33_P3347528 | 0.00151 | 0.0163 | 7.12 | 2.832 |
| LOC400604 | Homo sapiens uncharacterized LOC400604 (LOC400604), non-coding RNA [NR_038458] | A_21_P0000812 | 0.00151 | 0.0163 | 2.038 | 1.027 |
| XLOC_l2_014579 | AGENCOURT_8492923 NIH_MGC_100 Homo sapiens cDNA clone IMAGE:6300203 5', mRNA sequence [BQ652687] | A_21_P0013522 | 0.0015 | 0.0163 | 3.389 | 1.761 |
| DA567289 | carbonic anhydrase VB pseudogene 1 [Source:HGNC Symbol;Acc:29544] [ENST00000380336] | A_33_P3316064 | 0.00152 | 0.0164 | 3.348 | 1.743 |
| KRT42P | Homo sapiens keratin 42 pseudogene (KRT42P), non-coding RNA [NR_033415] | A_33_P3341490 | 0.00155 | 0.0166 | 2.246 | 1.167 |
| XLOC_000462 | BROAD Institute lincRNA (XLOC_000462), lincRNA [TCONS_00000333] | A_21_P0001029 | 0.00155 | 0.0166 | 12.536 | 3.648 |
| LOC100506561 | PREDICTED: Homo sapiens hypothetical LOC100506561 (LOC100506561), miscRNA [XR_109505] | A_21_P0014376 | 0.00158 | 0.0168 | 3.23 | 1.691 |
| XLOC_000371 | wc24f11.x1 NCI_CGAP_Pr28 Homo sapiens cDNA clone IMAGE:2316141 3', mRNA sequence [AI678218] | A_21_P0001369 | 0.00163 | 0.0172 | 3.545 | 1.826 |
| XLOC_014399 | PREDICTED: Homo sapiens hypothetical LOC100506737 (LOC100506737), miscRNA [XR_109732] | A_21_P0010449 | 0.00164 | 0.0172 | 10.845 | 3.439 |
| LINC00202 | Homo sapiens long intergenic non-protein coding RNA 202 (LINC00202), non-coding RNA [NR_026795] | A_21_P0010860 | 0.00163 | 0.0172 | 9.794 | 3.292 |
| XLOC_010843 | Q4KVU0_9GOBI (Q4KVU0) NADH dehydrogenase subunit 1, partial (5%) [THC2611835] | A_21_P0008270 | 0.00165 | 0.0173 | 7.323 | 2.872 |
| XLOC_002035 | PREDICTED: Homo sapiens hypothetical LOC100505774 (LOC100505774), miscRNA [XR_109967] | A_21_P0002655 | 0.00166 | 0.0173 | 2.389 | 1.257 |
| XLOC_009649 | BROAD Institute lincRNA (XLOC_009649), lincRNA [TCONS_00020316] | A_21_P0007522 | 0.00165 | 0.0173 | 3.292 | 1.719 |
| XLOC_006187 | BROAD Institute lincRNA (XLOC_006187), lincRNA [TCONS_00013551] | A_21_P0005407 | 0.00167 | 0.0174 | 2.277 | 1.187 |
| XLOC_l2_005418 | BROAD Institute lincRNA (XLOC_l2_005418), lincRNA [TCONS_l2_00010042] | A_21_P0011459 | 0.0017 | 0.0176 | 3.699 | 1.887 |

| | | | | | | |
|---|---|---|---|---|---|---|
| XLOC_010706 | 1207289A reverse transcriptase related protein. {Homo sapiens} (exp=-1; wgp=-1; cg=-1), partial (93%) [THC2502639] | A_21_P0008205 | 0.00169 | 0.0176 | 5.336 | 2.416 |
| A_19_P00321382 | PREDICTED: Homo sapiens hypothetical LOC100506860, transcript variant 2 (LOC100506860), miscRNA [XR_108814] | A_19_P00321382 | 0.00173 | 0.0178 | 3.23 | 1.691 |
| A_19_P00810751 | Q2Z8W5_9GAMM (Q2Z8W5) Thiolase , partial (5%) [THC2770336] | A_19_P00810751 | 0.00174 | 0.0178 | 2.651 | 1.407 |
| LOC100506379 | PREDICTED: Homo sapiens hypothetical LOC100506379, transcript variant 1 (LOC100506379), miscRNA [XR_108671] | A_21_P0014165 | 0.00174 | 0.0178 | 5.387 | 2.429 |
| XLOC_l2_008140 | Q9DC07_MOUSE (Q9DC07) Adult male lung cDNA, RIKEN full-length enriched library, clone:1200007O21 product:LIM AND SH3 PROTEIN (LASP Homolog), full insert sequence (Nebulette), partial (11%) [THC2523793] | A_21_P0011935 | 0.00174 | 0.0178 | 3.029 | 1.599 |
| XLOC_007095 | BROAD Institute lincRNA (XLOC_007095), lincRNA [TCONS_00015021] | A_21_P0005842 | 0.00172 | 0.0178 | 3.87 | 1.952 |
| LOC646890 | Unknown | A_32_P23795 | 0.00173 | 0.0178 | 1.506 | 0.59 |
| XLOC_005273 | BROAD Institute lincRNA (XLOC_005273), lincRNA [TCONS_00011799] | A_19_P00806593 | 0.00178 | 0.0181 | 2.422 | 1.276 |
| FLJ21408 | Homo sapiens uncharacterized LOC400512 (FLJ21408), non-coding RNA [NR_037184] | A_21_P0000676 | 0.00181 | 0.0183 | 4.977 | 2.315 |
| LOC152578 | Homo sapiens uncharacterized LOC152578 (LOC152578), non-coding RNA [NR_040106] | A_33_P3679221 | 0.00185 | 0.0186 | 5.526 | 2.466 |
| XLOC_010525 | Q7Z5D8_HUMAN (Q7Z5D8) Homeobox C14 protein, partial (13%) [THC2670501] | A_21_P0008127 | 0.0019 | 0.0189 | 4.476 | 2.162 |
| XLOC_005410 | BROAD Institute lincRNA (XLOC_005410), lincRNA [TCONS_00011909] | A_21_P0004915 | 0.0019 | 0.0189 | 8.003 | 3 |
| ENST00000314246 | Homo sapiens golgin A8 family, member B pseudogene (LOC653061), non-coding RNA [NR_038843] | A_23_P140614 | 0.00191 | 0.019 | 3.451 | 1.787 |
| XLOC_l2_008343 | BROAD Institute lincRNA (XLOC_l2_008343), lincRNA [TCONS_l2_00016011] | A_21_P0012097 | 0.00198 | 0.0194 | 6.192 | 2.63 |
| XLOC_005462 | BROAD Institute lincRNA (XLOC_005462), lincRNA [TCONS_00011369] | A_21_P0004684 | 0.00199 | 0.0194 | 2.292 | 1.197 |
| XLOC_010989 | BROAD Institute lincRNA (XLOC_010989), lincRNA [TCONS_00022700] | A_21_P0008418 | 0.00201 | 0.0195 | 4.364 | 2.126 |

| | | | | | | |
|---|---|---|---|---|---|---|
| LOC100506578 | PREDICTED: Homo sapiens uncharacterized protein C12orf71-like (LOC100506578), miscRNA [XR_132581] | A_21_P0014789 | 0.00206 | 0.0198 | 2.625 | 1.392 |
| XLOC_004032 | BROAD Institute lincRNA (XLOC_004032), lincRNA [TCONS_00007807] | A_21_P0003488 | 0.00207 | 0.0199 | 11.645 | 3.542 |
| A_19_P00316333 | Homo sapiens uncharacterized LOC100506714 (LOC100506714), transcript variant 1, non-coding RNA [NR_038956] | A_19_P00316333 | 0.0021 | 0.0201 | 3.034 | 1.601 |
| FLJ32679 | Homo sapiens golgin A8 family, member F (GOLGA8F), non-coding RNA [NR_033351] | A_24_P264166 | 0.00212 | 0.0202 | 2.282 | 1.19 |
| LOC100505932 | PREDICTED: Homo sapiens hypothetical LOC100505932 (LOC100505932), miscRNA [XR_113037] | A_21_P0014730 | 0.00212 | 0.0202 | 2.243 | 1.165 |
| XLOC_014105 | GB | A_19_P00316110 | 0.00216 | 0.0204 | 3.405 | 1.768 |
| XLOC_004875 | BROAD Institute lincRNA (XLOC_004875), lincRNA [TCONS_00010371] | A_21_P0004409 | 0.00218 | 0.0205 | 7.861 | 2.975 |
| XLOC_011797 | Q3W9K3_9ACTO (Q3W9K3) Acyl transferase domain:Phosphopantetheine-binding domain, partial (3%) [THC2633387] | A_21_P0008978 | 0.00219 | 0.0206 | 3.875 | 1.954 |
| XLOC_006291 | BROAD Institute lincRNA (XLOC_006291), lincRNA [TCONS_00013626] | A_21_P0005441 | 0.00219 | 0.0206 | 2.564 | 1.358 |
| XLOC_l2_013293 | BROAD Institute lincRNA (XLOC_l2_013293), lincRNA [TCONS_l2_00025688] | A_21_P0013175 | 0.00219 | 0.0206 | 3.213 | 1.684 |
| GNASAS | Homo sapiens GNAS antisense RNA 1 (non-protein coding) (GNAS-AS1), antisense RNA [NR_002785] | A_33_P3214239 | 0.00222 | 0.0207 | 5.158 | 2.367 |
| XLOC_000390 | EST375352 MAGE resequences, MAGH Homo sapiens cDNA, mRNA sequence [AW963279] | A_21_P0001704 | 0.00224 | 0.0208 | 2.129 | 1.09 |
| XLOC_004064 | BROAD Institute lincRNA (XLOC_004064), lincRNA [TCONS_00007433] | A_21_P0003324 | 0.0023 | 0.0211 | 2.412 | 1.27 |
| LOC100190939 | Homo sapiens uncharacterized LOC100190939 (LOC100190939), non-coding RNA [NR_024458] | A_24_P110914 | 0.00236 | 0.0214 | 2.117 | 1.082 |
| XLOC_012847 | BROAD Institute lincRNA (XLOC_012847), lincRNA [TCONS_00026523] | A_21_P0009558 | 0.00235 | 0.0214 | 11.828 | 3.564 |
| LOC100506861 | PREDICTED: Homo sapiens hypothetical LOC100506861 (LOC100506861), miscRNA [XR_113160] | A_21_P0014737 | 0.00238 | 0.0215 | 3.101 | 1.633 |

| | | | | | | |
|---|---|---|---|---|---|---|
| LOC220429 | Homo sapiens CTAGE family, member 10, pseudogene (CTAGE10P), non-coding RNA [NR_003268] | A_33_P3340007 | 0.0024 | 0.0216 | 4.361 | 2.125 |
| C1orf144 | BROAD Institute lincRNA (XLOC_000062), lincRNA [TCONS_00000847] | A_21_P0001267 | 0.00239 | 0.0216 | 3.301 | 1.723 |
| LOC650226 | Homo sapiens ankyrin repeat domain 26 pseudogene (LOC650226), non-coding RNA [NR_029420] | A_21_P0013425 | 0.00241 | 0.0217 | 9.028 | 3.174 |
| XLOC_008141 | BROAD Institute lincRNA (XLOC_008141), lincRNA [TCONS_00017307] | A_21_P0006531 | 0.00241 | 0.0217 | 3.575 | 1.838 |
| XLOC_010069 | BROAD Institute lincRNA (XLOC_010069), lincRNA [TCONS_00020779] | A_21_P0007707 | 0.00243 | 0.0218 | 2.81 | 1.49 |
| ENST00000329081 | Homo sapiens POTE ankyrin domain family, member K, pseudogene (POTEKP), non-coding RNA [NR_033885] | A_32_P155776 | 0.00251 | 0.0222 | 2.328 | 1.219 |
| AL162073 | Homo sapiens uncharacterized LOC728431 (LOC728431), non-coding RNA [NR_038842] | A_32_P128391 | 0.00251 | 0.0222 | 2.974 | 1.573 |
| XLOC_008394 | BROAD Institute lincRNA (XLOC_008394), lincRNA [TCONS_00018124] | A_21_P0006836 | 0.00253 | 0.0223 | 27.826 | 4.798 |
| LOC728875 | Homo sapiens uncharacterized LOC728875 (LOC728875), non-coding RNA [NR_024584] | A_21_P0001705 | 0.00256 | 0.0225 | 3.796 | 1.924 |
| XLOC_009363 | BROAD Institute lincRNA (XLOC_009363), lincRNA [TCONS_00019165] | A_21_P0007155 | 0.00256 | 0.0225 | 2.586 | 1.371 |
| THC2620351 | PREDICTED: Homo sapiens hypothetical LOC100130548 (LOC100130548), miscRNA [XR_110579] | A_33_P3423450 | 0.00259 | 0.0226 | 2.843 | 1.508 |
| LOC100505678 | Homo sapiens uncharacterized LOC100505678 (LOC100505678), non-coding RNA [NR_040093] | A_21_P0000900 | 0.00261 | 0.0227 | 2.804 | 1.487 |
| XLOC_005582 | BROAD Institute lincRNA (XLOC_005582), lincRNA [TCONS_00011431] | A_21_P0004717 | 0.00265 | 0.0229 | 3.977 | 1.992 |
| FLJ46906 | Homo sapiens uncharacterized LOC441172 (FLJ46906), non-coding RNA [NR_033896] | A_33_P3340490 | 0.00266 | 0.023 | 4.841 | 2.275 |
| SNORA74B | Homo sapiens small nucleolar RNA, H/ACA box 74B (SNORA74B), small nucleolar RNA [NR_002988] | A_33_P3222367 | 0.00269 | 0.0231 | 2.059 | 1.042 |
| LOC100499489 | Homo sapiens uncharacterized LOC100499489 (LOC100499489), non-coding RNA [NR_036533] | A_21_P0000660 | 0.00275 | 0.0234 | 5.045 | 2.335 |

| | | | | | | |
|---|---|---|---|---|---|---|
| LOC100505905 | PREDICTED: Homo sapiens hypothetical LOC100505905, transcript variant 1 (LOC100505905), miscRNA [XR_108921] | A_21_P0014225 | 0.00278 | 0.0235 | 4.435 | 2.149 |
| LOC100506120 | PREDICTED: Homo sapiens hypothetical LOC100506120 (LOC100506120), miscRNA [XR_110570] | A_21_P0014616 | 0.00278 | 0.0235 | 4.436 | 2.149 |
| LOC100289211 | Homo sapiens uncharacterized LOC100289211 (LOC100289211), non-coding RNA [NR_038423] | A_21_P0000795 | 0.0028 | 0.0236 | 3.34 | 1.74 |
| LOC257358 | Homo sapiens uncharacterized LOC257358 (LOC257358), non-coding RNA [NR_026945] | A_21_P0004329 | 0.00284 | 0.0238 | 4.483 | 2.164 |
| LOC145694 | PREDICTED: Homo sapiens hypothetical LOC145694 (LOC145694), miscRNA [XR_109210] | A_33_P3441639 | 0.00287 | 0.0239 | 6.221 | 2.637 |
| XLOC_004860 | ALU1_HUMAN (P39188) Alu subfamily J sequence contamination warning entry, partial (12%) [THC2634258] | A_21_P0003939 | 0.00286 | 0.0239 | -1.032 | -0.045 |
| XLOC_005564 | BX088681 Soares_testis_NHT Homo sapiens cDNA clone IMAGp998F233332, mRNA sequence [BX088681] | A_21_P0004714 | 0.00287 | 0.0239 | 2.73 | 1.449 |
| XLOC_003087 | BROAD Institute lincRNA (XLOC_003087), lincRNA [TCONS_00006483] | A_21_P0003112 | 0.00306 | 0.0249 | 3.286 | 1.717 |
| A_19_P00321631 | BROAD Institute lincRNA (XLOC_l2_013080), lincRNA [TCONS_l2_00025449] | A_19_P00321631 | 0.00308 | 0.025 | 2.852 | 1.512 |
| LOC100653033 | PREDICTED: Homo sapiens hypothetical LOC100653033 (LOC100653033), miscRNA [XR_132874] | A_21_P0014907 | 0.00321 | 0.0255 | 3.164 | 1.662 |
| DKFZP564C196 | Homo sapiens uncharacterized LOC284649 (DKFZP564C196), non-coding RNA [NR_040063] | A_21_P0000896 | 0.0032 | 0.0255 | 3.912 | 1.968 |
| XLOC_l2_002433 | BROAD Institute lincRNA (XLOC_l2_002433), lincRNA [TCONS_l2_00004711] | A_21_P0010993 | 0.00324 | 0.0256 | 2.792 | 1.482 |
| XLOC_003993 | HSDRPI2 DNA-directed RNA polymerase I largest subunit {Homo sapiens} (exp=-1; wgp=0; cg=0), partial (4%) [THC2633082] | A_21_P0003748 | 0.00326 | 0.0257 | 3.526 | 1.818 |
| A_19_P00317412 | Homo sapiens uncharacterized LOC728431 (LOC728431), non-coding RNA [NR_038842] | A_19_P00317412 | 0.00328 | 0.0258 | 2.877 | 1.524 |
| XLOC_003824 | Q5GWG5_XANOR (Q5GWG5) IS1404 transposase, partial (8%) [THC2784508] | A_21_P0003435 | 0.00327 | 0.0258 | 4.483 | 2.164 |

| | | | | | | |
|---|---|---|---|---|---|---|
| NBR2 | Homo sapiens neighbor of BRCA1 gene 2 (non-protein coding) (NBR2), non-coding RNA [NR_003108] | A_24_P301454 | 0.00332 | 0.026 | 2.194 | 1.133 |
| LOC100505566 | PREDICTED: Homo sapiens hypothetical LOC100505566, transcript variant 1 (LOC100505566), miscRNA [XR_108515] | A_21_P0014129 | 0.00337 | 0.0261 | 6.437 | 2.686 |
| LOC100505545 | Homo sapiens uncharacterized LOC100505545 (LOC100505545), non-coding RNA [NR_038331] | A_21_P0000768 | 0.00344 | 0.0264 | 5.398 | 2.432 |
| XLOC_000643 | Unknown | A_21_P0000925 | 0.00351 | 0.0267 | 2.673 | 1.419 |
| LOC399744 | Homo sapiens uncharacterized LOC399744 (LOC399744), non-coding RNA [NR_024497] | A_33_P3248252 | 0.00355 | 0.0269 | 2.068 | 1.048 |
| XLOC_009080 | Unknown | A_21_P0007430 | 0.00356 | 0.0269 | 2.61 | 1.384 |
| XLOC_002977 | BROAD Institute lincRNA (XLOC_002977), lincRNA [TCONS_00005698] | A_19_P00318544 | 0.00358 | 0.027 | 3.965 | 1.987 |
| A_19_P00316479 | BROAD Institute lincRNA (XLOC_l2_013080), lincRNA [TCONS_l2_00025448] | A_19_P00316479 | 0.00359 | 0.0271 | 2.145 | 1.101 |
| DGCR9 | Homo sapiens DiGeorge syndrome critical region gene 9 (DGCR9), non-coding RNA [NR_024159] | A_33_P3216098 | 0.00375 | 0.0277 | 4.817 | 2.268 |
| XLOC_004723 | BROAD Institute lincRNA (XLOC_004723), lincRNA [TCONS_00010257] | A_21_P0004357 | 0.00377 | 0.0278 | 18.958 | 4.245 |
| XLOC_012258 | BROAD Institute lincRNA (XLOC_012258), lincRNA [TCONS_00025440] | A_21_P0009261 | 0.00379 | 0.0279 | 9.99 | 3.321 |
| LOC643072 | PREDICTED: Homo sapiens hypothetical LOC643072 (LOC643072), miscRNA [XR_108434] | A_21_P0014102 | 0.0038 | 0.028 | 4.06 | 2.022 |
| XLOC_013567 | BX094767 Soares_testis_NHT Homo sapiens cDNA clone IMAGp998G084495, mRNA sequence [BX094767] | A_21_P0009855 | 0.00383 | 0.0281 | 1.949 | 0.963 |
| XLOC_l2_011669 | Q5TF36_HUMAN (Q5TF36) REV3-like, catalytic subunit of DNA polymerase zeta (Yeast), partial (5%) [THC2660888] | A_21_P0012837 | 0.0039 | 0.0284 | 3.68 | 1.88 |
| C18orf18 | Homo sapiens chromosome 18 open reading frame 18 (C18orf18), non-coding RNA [NR_026849] | A_23_P377965 | 0.00395 | 0.0286 | 2.111 | 1.078 |
| XLOC_000839 | BROAD Institute lincRNA (XLOC_000839), lincRNA [TCONS_00001498] | A_21_P0001506 | 0.00396 | 0.0286 | 3.057 | 1.612 |
| XLOC_000909 | Q2QUZ4_ORYSA (Q2QUZ4) Tetraspanin family, partial (5%) [THC2731633] | A_21_P0001540 | 0.00395 | 0.0286 | 2.438 | 1.286 |

| | | | | | | |
|---|---|---|---|---|---|---|
| LOC100131733 | Homo sapiens uncharacterized LOC100131733 (LOC100131733), non-coding RNA [NR_038996] | A_32_P56249 | 0.00401 | 0.0288 | 3.289 | 1.718 |
| A_19_P00319917 | Homo sapiens uncharacterized LOC100506714 (LOC100506714), transcript variant 1, non-coding RNA [NR_038956] | A_19_P00319917 | 0.00408 | 0.0291 | 3.84 | 1.941 |
| XLOC_010791 | BROAD Institute lincRNA (XLOC_010791), lincRNA [TCONS_00022460] | A_21_P0008316 | 0.00408 | 0.0291 | 3.061 | 1.614 |
| XLOC_013506 | BROAD Institute lincRNA (XLOC_013506), lincRNA [TCONS_00027922] | A_21_P0009840 | 0.0041 | 0.0292 | 2.323 | 1.216 |
| XLOC_l2_011798 | GB | A_21_P0012958 | 0.0041 | 0.0292 | 2.345 | 1.23 |
| XLOC_010933 | BROAD Institute lincRNA (XLOC_010933), lincRNA [TCONS_00022632] | A_21_P0008390 | 0.0041 | 0.0292 | 4.597 | 2.201 |
| LOC100507670 | PREDICTED: Homo sapiens hypothetical LOC100507670 (LOC100507670), miscRNA [XR_108348] | A_21_P0014072 | 0.00414 | 0.0293 | 2.005 | 1.004 |
| XLOC_005681 | NM_074696 F21H7.4 {Caenorhabditis elegans} (exp=-1; wgp=0; cg=0), partial (6%) [THC2609574] | A_21_P0005162 | 0.00415 | 0.0294 | 2.41 | 1.269 |
| XLOC_l2_003909 | AF080231 polymerase {Homo sapiens} (exp=-1; wgp=0; cg=0), partial (25%) [THC2604413] | A_21_P0011219 | 0.00422 | 0.0296 | 2.421 | 1.275 |
| XLOC_012338 | ALU7_HUMAN (P39194) Alu subfamily SQ sequence contamination warning entry, partial (11%) [THC2721173] | A_21_P0009287 | 0.00432 | 0.0299 | 7.253 | 2.859 |
| C1orf213 | Homo sapiens chromosome 1 open reading frame 213 (C1orf213), transcript variant 2, non-coding RNA [NR_033691] | A_33_P3363720 | 0.00435 | 0.03 | 2.261 | 1.177 |
| LOC100505869 | PREDICTED: Homo sapiens hypothetical LOC100505869 (LOC100505869), miscRNA [XR_110454] | A_21_P0006788 | 0.00437 | 0.0301 | 4.422 | 2.145 |
| A_19_P00802257 | Homo sapiens uncharacterized LOC728431 (LOC728431), non-coding RNA [NR_038842] | A_19_P00802257 | 0.00439 | 0.0302 | 2.862 | 1.517 |
| HCG4 | Homo sapiens HLA complex group 4 (non-protein coding) (HCG4), non-coding RNA [NR_002139] | A_23_P254768 | 0.00446 | 0.0304 | 7.525 | 2.912 |
| LOC729444 | Homo sapiens uncharacterized LOC729444 (LOC729444), non-coding RNA [NR_038388] | A_21_P0012317 | 0.00446 | 0.0304 | 3.576 | 1.838 |
| DLEU2L | Homo sapiens deleted in lymphocytic leukemia 2-like (DLEU2L), non-coding RNA [NR_002771] | A_33_P3424204 | 0.00451 | 0.0306 | 3.083 | 1.625 |

| | | | | | | |
|---|---|---|---|---|---|---|
| XLOC_002554 | BROAD Institute lincRNA (XLOC_002554), lincRNA [TCONS_00004641] | A_21_P0002558 | 0.00453 | 0.0307 | 3.979 | 1.992 |
| SNORD114-21 | Homo sapiens small nucleolar RNA, C/D box 114-21 (SNORD114-21), small nucleolar RNA [NR_003214] | A_21_P0000418 | 0.00457 | 0.0309 | 3.571 | 1.836 |
| XLOC_009498 | BROAD Institute lincRNA (XLOC_009498), lincRNA [TCONS_00019700] | A_21_P0007370 | 0.0046 | 0.031 | 3.312 | 1.728 |
| XLOC_007354 | BROAD Institute lincRNA (XLOC_007703), lincRNA [TCONS_00016773] | A_21_P0006147 | 0.00472 | 0.0315 | 3.678 | 1.879 |
| XLOC_002968 | BROAD Institute lincRNA (XLOC_002968), lincRNA [TCONS_00005691] | A_21_P0002823 | 0.00476 | 0.0316 | 2.053 | 1.038 |
| XLOC_006980 | BROAD Institute lincRNA (XLOC_006980), lincRNA [TCONS_00015396] | A_21_P0005620 | 0.00488 | 0.032 | 2.638 | 1.399 |
| XLOC_001596 | BROAD Institute lincRNA (XLOC_001596), lincRNA [TCONS_00003801] | A_21_P0002262 | 0.00493 | 0.0321 | 3.062 | 1.614 |
| XLOC_009272 | BROAD Institute lincRNA (XLOC_009272), lincRNA [TCONS_00019459] | A_21_P0007273 | 0.005 | 0.0324 | 5.584 | 2.481 |
| LOC100505851 | PREDICTED: Homo sapiens hypothetical LOC100505851 (LOC100505851), miscRNA [XR_109588] | A_21_P0011733 | 0.00517 | 0.033 | 3.827 | 1.936 |
| LOC100505501 | PREDICTED: Homo sapiens hypothetical LOC100505501, transcript variant 2 (LOC100505501), miscRNA [XR_108911] | A_21_P0014222 | 0.0052 | 0.0331 | 9.278 | 3.214 |
| XLOC_001262 | Q13400_HUMAN (Q13400) HPMSR2, partial (6%) [THC2628110] | A_21_P0001243 | 0.00528 | 0.0334 | 4.649 | 2.217 |
| XLOC_l2_005553 | BROAD Institute lincRNA (XLOC_l2_005553), lincRNA [TCONS_l2_00010285] | A_21_P0011488 | 0.00528 | 0.0334 | 2.644 | 1.402 |
| LOC731424 | Homo sapiens uncharacterized LOC731424 (LOC731424), non-coding RNA [NR_037867] | A_21_P0000695 | 0.0053 | 0.0335 | 5.659 | 2.501 |
| LOC100131564 | Homo sapiens uncharacterized LOC100131564 (LOC100131564), non-coding RNA [NR_034089] | A_21_P0001549 | 0.0053 | 0.0335 | 2.104 | 1.073 |
| XLOC_000808 | Q2E3E3_ACICE (Q2E3E3) Pyrrolo-quinoline quinone, partial (4%) [THC2644607] | A_21_P0001123 | 0.00531 | 0.0336 | 2.936 | 1.554 |
| A_19_P00809139 | AGENCOURT_8492923 NIH_MGC_100 Homo sapiens cDNA clone IMAGE:6300203 5', mRNA sequence [BQ652687] | A_19_P00809139 | 0.00536 | 0.0337 | 2.473 | 1.306 |

| | | | | | | |
|---|---|---|---|---|---|---|
| LOC100505702 | Homo sapiens uncharacterized LOC100505702 (LOC100505702), transcript variant 1, non-coding RNA [NR_038303] | A_21_P0012594 | 0.00545 | 0.034 | 5.78 | 2.531 |
| XLOC_007093 | BROAD Institute lincRNA (XLOC_007093), lincRNA [TCONS_00015019] | A_21_P0005840 | 0.00547 | 0.0341 | 3.409 | 1.769 |
| XLOC_014101 | BROAD Institute lincRNA (XLOC_014101), lincRNA [TCONS_00028945] | A_19_P00322017 | 0.0055 | 0.0342 | 3.508 | 1.81 |
| XLOC_004859 | BROAD Institute lincRNA (XLOC_004859), lincRNA [TCONS_00010946] | A_21_P0004405 | 0.00561 | 0.0345 | 4.842 | 2.275 |
| GGT3P | Homo sapiens gamma-glutamyltransferase 3 pseudogene (GGT3P), non-coding RNA [NR_003267] | A_21_P0011817 | 0.00565 | 0.0347 | 5.073 | 2.343 |
| SCARNA15 | Homo sapiens small Cajal body-specific RNA 15 (SCARNA15), guide RNA [NR_003011] | A_21_P0000355 | 0.00569 | 0.0348 | 3.99 | 1.997 |
| XLOC_000475 | BROAD Institute lincRNA (XLOC_000475), lincRNA [TCONS_00001209] | A_21_P0001389 | 0.00585 | 0.0354 | 2.479 | 1.31 |
| XLOC_l2_013153 | BROAD Institute lincRNA (XLOC_l2_013153), lincRNA [TCONS_l2_00024967] | A_21_P0013108 | 0.00594 | 0.0357 | 3.721 | 1.896 |
| XLOC_l2_010723 | BROAD Institute lincRNA (XLOC_l2_010723), lincRNA [TCONS_l2_00021618] | A_19_P00319578 | 0.00596 | 0.0358 | 2.51 | 1.327 |
| ANXA2P3 | Homo sapiens annexin A2 pseudogene 3 (ANXA2P3), non-coding RNA [NR_001446] | A_24_P323114 | 0.00606 | 0.0362 | 3.176 | 1.667 |
| A_19_P00316743 | Homo sapiens uncharacterized LOC285375 (LOC285375), non-coding RNA [NR_027103] | A_19_P00316743 | 0.00608 | 0.0363 | 3.465 | 1.793 |
| XLOC_007494 | BROAD Institute lincRNA (XLOC_007494), lincRNA [TCONS_00016107] | A_21_P0006195 | 0.00622 | 0.0368 | 3.148 | 1.655 |
| XLOC_000757 | BROAD Institute lincRNA (XLOC_000757), lincRNA [TCONS_00001441] | A_21_P0001485 | 0.00624 | 0.0368 | 2.245 | 1.167 |
| BCAR4 | Homo sapiens breast cancer anti-estrogen resistance 4 (non-protein coding) (BCAR4), non-coding RNA [NR_024049] | A_32_P106933 | 0.00643 | 0.0375 | 4.63 | 2.211 |
| LOC100506686 | Homo sapiens uncharacterized LOC100506686 (LOC100506686), transcript variant 1, non-coding RNA [NR_040051] | A_21_P0000893 | 0.00645 | 0.0376 | 2.154 | 1.107 |
| LOC730227 | Homo sapiens uncharacterized LOC730227 (LOC730227), transcript variant 1, non-coding RNA [NR_034151] | A_33_P3411328 | 0.00656 | 0.0379 | 2.672 | 1.418 |

| | | | | | | |
|---|---|---|---|---|---|---|
| XLOC_I2_007135 | BROAD Institute lincRNA (XLOC_I2_007135), lincRNA [TCONS_I2_00013243] | A_21_P0011764 | 0.0067 | 0.0384 | 2.764 | 1.467 |
| A_19_P00321383 | PREDICTED: Homo sapiens hypothetical LOC100506860, transcript variant 2 (LOC100506860), miscRNA [XR_108814] | A_19_P00321383 | 0.00673 | 0.0385 | 5.635 | 2.494 |
| CTAGE7P | Homo sapiens CTAGE family, member 7, pseudogene (CTAGE7P), non-coding RNA [NR_044994] | A_21_P0010847 | 0.00675 | 0.0386 | 4.407 | 2.14 |
| LOC148709 | Homo sapiens actin pseudogene (LOC148709), non-coding RNA [NR_002929] | A_24_P84880 | 0.00681 | 0.0388 | 2.517 | 1.332 |
| XLOC_005052 | wu95d10.x1 NCI_CGAP_Kid3 Homo sapiens cDNA clone IMAGE:2527795 3', mRNA sequence [AW025248] | A_21_P0004575 | 0.00705 | 0.0396 | 3.061 | 1.614 |
| XLOC_013583 | zt15g10.s1 NCI_CGAP_GCB1 Homo sapiens cDNA clone IMAGE:713250 3', mRNA sequence [AA282947] | A_21_P0009866 | 0.00716 | 0.04 | 2.207 | 1.142 |
| XLOC_010367 | BROAD Institute lincRNA (XLOC_010367), lincRNA [TCONS_00021771] | A_21_P0008058 | 0.00716 | 0.04 | 2.525 | 1.336 |
| XLOC_009194 | BROAD Institute lincRNA (XLOC_009194), lincRNA [TCONS_00019370] | A_19_P00804359 | 0.00729 | 0.0404 | 2.914 | 1.543 |
| XLOC_I2_001664 | BROAD Institute lincRNA (XLOC_I2_001664), lincRNA [TCONS_I2_00003144] | A_21_P0010836 | 0.00728 | 0.0404 | 3.453 | 1.788 |
| C10orf110 | Homo sapiens IDI2 antisense RNA 1 (non-protein coding) (IDI2-AS1), transcript variant 4, non-coding RNA [NR_027709] | A_23_P24275 | 0.00733 | 0.0406 | 2.656 | 1.409 |
| XLOC_I2_004192 | BROAD Institute lincRNA (XLOC_I2_004192), lincRNA [TCONS_I2_00007731] | A_21_P0011244 | 0.00746 | 0.041 | 5.046 | 2.335 |
| LOC100507599 | PREDICTED: Homo sapiens hypothetical LOC100507599 (LOC100507599), miscRNA [XR_109753] | A_21_P0014432 | 0.00747 | 0.041 | 3.397 | 1.764 |
| XLOC_012759 | BROAD Institute lincRNA (XLOC_012759), lincRNA [TCONS_00026415] | A_21_P0009523 | 0.0075 | 0.0411 | 2.276 | 1.186 |
| XLOC_I2_013835 | BROAD Institute lincRNA (XLOC_I2_013835), lincRNA [TCONS_I2_00026573] | A_21_P0013305 | 0.00769 | 0.0418 | 4.89 | 2.29 |
| XLOC_I2_009292 | PREDICTED: Homo sapiens hypothetical LOC100506454 (LOC100506454), miscRNA [XR_109700] | A_21_P0012310 | 0.00771 | 0.0419 | 3.962 | 1.986 |

| | | | | | | |
|---|---|---|---|---|---|---|
| XLOC_000535 | BROAD Institute lincRNA (XLOC_000535), lincRNA [TCONS_00001250] | A_21_P0001407 | 0.00777 | 0.0421 | 2.337 | 1.225 |
| XLOC_I2_014694 | BROAD Institute lincRNA (XLOC_I2_014694), lincRNA [TCONS_I2_00028607] | A_21_P0013550 | 0.00791 | 0.0426 | 2.317 | 1.212 |
| FLJ38109 | Homo sapiens uncharacterized LOC386627 (FLJ38109), non-coding RNA [NR_037897] | A_21_P0000704 | 0.00791 | 0.0426 | 3.336 | 1.738 |
| XLOC_006725 | BROAD Institute lincRNA (XLOC_006725), lincRNA [TCONS_00014630] | A_19_P00327320 | 0.00801 | 0.0429 | 3.398 | 1.765 |
| XLOC_004529 | BROAD Institute lincRNA (XLOC_004529), lincRNA [TCONS_00009498] | A_21_P0004055 | 0.00809 | 0.0432 | 6.104 | 2.61 |
| A_19_P00316050 | PREDICTED: Homo sapiens hypothetical LOC100506860, transcript variant 1 (LOC100506860), miscRNA [XR_108813] | A_19_P00316050 | 0.00815 | 0.0434 | 2.051 | 1.036 |
| XLOC_008925 | BROAD Institute lincRNA (XLOC_008925), lincRNA [TCONS_00018589] | A_19_P00802238 | 0.00822 | 0.0436 | 2.337 | 1.225 |
| XLOC_012393 | PREDICTED: Homo sapiens hypothetical LOC100506974 (LOC100506974), miscRNA [XR_111668] | A_21_P0009299 | 0.00848 | 0.0444 | 2.877 | 1.524 |
| XLOC_005952 | DMGCR6 fibrillarin {Drosophila melanogaster} (exp=-1; wgp=0; cg=0), partial (32%) [THC2665498] | A_19_P00321403 | 0.00853 | 0.0445 | 4.939 | 2.304 |
| BSN-AS2 | Homo sapiens BSN antisense RNA 2 (non-protein coding) (BSN-AS2), non-coding RNA [NR_038866] | A_21_P0002714 | 0.00869 | 0.0451 | 2.677 | 1.421 |
| RN5-8S1 | Homo sapiens RNA, 5.8S ribosomal 1 (RN5-8S1), ribosomal RNA [NR_003285] | A_33_P3399064 | 0.00875 | 0.0453 | 2.1 | 1.07 |
| SNORA12 | Homo sapiens small nucleolar RNA, H/ACA box 12 (SNORA12), small nucleolar RNA [NR_002954] | A_21_P0000310 | 0.00885 | 0.0456 | 2.039 | 1.028 |
| XLOC_I2_002761 | BROAD Institute lincRNA (XLOC_I2_002761), lincRNA [TCONS_I2_00005177] | A_19_P00813192 | 0.00898 | 0.046 | 42.058 | 5.394 |
| LOC100506282 | PREDICTED: Homo sapiens hypothetical LOC100506282 (LOC100506282), miscRNA [XR_110899] | A_21_P0014650 | 0.00898 | 0.046 | 2.908 | 1.54 |
| XLOC_002870 | BROAD Institute lincRNA (XLOC_002870), lincRNA [TCONS_00006267] | A_21_P0003041 | 0.009 | 0.0461 | 4.069 | 2.025 |

| | | | | | | |
|---|---|---|---|---|---|---|
| ENST00000400141 | Homo sapiens transmembrane emp24 protein transport domain containing 11, pseudogene (TMED11P), non-coding RNA [NR_033768] | A_33_P3212395 | 0.00908 | 0.0464 | 4.799 | 2.263 |
| XLOC_004165 | BROAD Institute lincRNA (XLOC_004165), lincRNA [TCONS_00009166] | A_21_P0003908 | 0.00945 | 0.0476 | 2.138 | 1.096 |
| XLOC_003509 | BROAD Institute lincRNA (XLOC_003509), lincRNA [TCONS_00007514] | A_21_P0003351 | 0.00948 | 0.0478 | 2.694 | 1.43 |
| XLOC_004493 | BROAD Institute lincRNA (XLOC_004493), lincRNA [TCONS_00009475] | A_21_P0004041 | 0.00954 | 0.0479 | 10.904 | 3.447 |
| XLOC_008972 | BROAD Institute lincRNA (XLOC_008972), lincRNA [TCONS_00018061] | A_21_P0006797 | 0.00957 | 0.048 | 2.595 | 1.376 |
| XLOC_002347 | BX100678 NCI_CGAP_Co8 Homo sapiens cDNA clone IMAGp998C214725, mRNA sequence [BX100678] | A_21_P0002111 | 0.00971 | 0.0484 | 21.672 | 4.438 |
| XLOC_003709 | BROAD Institute lincRNA (XLOC_003709), lincRNA [TCONS_00007640] | A_21_P0003409 | 0.00977 | 0.0486 | 3.939 | 1.978 |
| LOC283050 | Homo sapiens uncharacterized LOC283050 (LOC283050), transcript variant 1, non-coding RNA [NR_024431] | A_33_P3394140 | 0.0098 | 0.0487 | 2.603 | 1.38 |
| XLOC_004607 | Unknown | A_21_P0003932 | 0.00981 | 0.0487 | 2.049 | 1.035 |
| ENST00000370548 | Homo sapiens uncharacterized LOC339524 (LOC339524), transcript variant 5, non-coding RNA [NR_026989] | A_32_P112592 | 0.00993 | 0.0491 | 3.797 | 1.925 |
| XLOC_005851 | BROAD Institute lincRNA (XLOC_005851), lincRNA [TCONS_00011573] | A_21_P0004779 | 0.00997 | 0.0492 | 4.19 | 2.067 |
| LOC100652951 | PREDICTED: Homo sapiens hypothetical LOC100652951 (LOC100652951), miscRNA [XR_132888] | A_21_P0014898 | 0.0102 | 0.0499 | 3.475 | 1.797 |
