# Supplementary material for: CEBPA-regulated lncRNAs, new players in the study of acute myeloid leukemia
Source: J Hematol Oncol. 2014 Sep 25;7:69. doi: 10.1186/s13045-014-0069-1 (PMC4177583; doi:10.1186/s13045-014-0069-1)
Supplement: Additional file 3: Table S1. — CEBPA-regulated lncRNAs with significant differential expression (absolute fold change ≥ 2 and adjusted P value ≤ 0.05) identified in K562. (A) Up-regulated lncRNAs. (B) Down-regulated lncRNAs. [file 13045_2014_69_MOESM3_ESM.zip › 13045_2014_69_fig5/13045_2014_69_add6.pdf]

Supplementary Table S1B. CEBPA-downregulated lncRNAs						
**Name**	**Description**	**ProbeID**	**p.value**	**Adj.p.value**	**Fold_Change**	**Log2_Ratio**
LOC100506995	PREDICTED: Homo sapiens hypothetical LOC100506995 (LOC100506995), miscRNA [XR_108482]	A_21_P0014114	6.7E-08	0.0000537	-4.244	-2.085
XLOC_003123	BROAD Institute lincRNA (XLOC_003123), lincRNA	A_21_P0002852	6.3E-07	0.000202	-5.775	-2.53
LOC100507567	Homo sapiens uncharacterized LOC100507567 (LOC100507567), non-coding RNA [NR_038237]	A_21_P0000732	2.9E-06	0.000432	-3.219	-1.687
MGC23284	Homo sapiens uncharacterized LOC197187 (MGC23284), transcript variant 1, non-coding RNA [NR_024402]	A_33_P3388938	3.9E-06	0.000512	-2.053	-1.038
A_19_P00801088	PREDICTED: Homo sapiens hypothetical LOC100289026 (LOC100289026), miscRNA [XR_110942]	A_19_P00801088	6.4E-06	0.000694	-10.37	-3.374
LOC730184	PREDICTED: Homo sapiens hypothetical LOC730184 (LOC730184), miscRNA [XR_109942]	A_33_P3397920	6.9E-06	0.000728	-7.6	-2.926
LOC100506691	PREDICTED: Homo sapiens hypothetical LOC100506691 (LOC100506691), miscRNA [XR_109143]	A_21_P0011069	9.9E-06	0.000889	-3.168	-1.664
ANKRD20A8P	Homo sapiens ankyrin repeat domain 20 family, member A8, pseudogene (ANKRD20A8P), non-coding RNA	A_21_P0010769	1.2E-05	0.000947	-6.231	-2.64
XLOC_011350	BROAD Institute lincRNA (XLOC_011350), lincRNA	A_21_P0008673	1.4E-05	0.00104	-13.913	-3.798
ANKRD20B	Homo sapiens ankyrin repeat domain 20 family, member A8, pseudogene (ANKRD20A8P), non-coding RNA	A_33_P3283599	1.5E-05	0.00111	-4.631	-2.211
LOC100505938	PREDICTED: Homo sapiens hypothetical LOC100505938 (LOC100505938), miscRNA [XR_108740]	A_21_P0014184	1.8E-05	0.0012	-2.966	-1.568
XLOC_009920	BROAD Institute lincRNA (XLOC_009920), lincRNA	A_21_P0007505	1.9E-05	0.00124	-3.987	-1.995
XLOC_l2_000399	BROAD Institute lincRNA (XLOC_l2_000399), lincRNA [TCONS_l2_00000563]	A_21_P0010555	2.2E-05	0.00134	-3.12	-1.642
MCM3APAS	Homo sapiens MCM3AP antisense RNA 1 (non-protein coding) (MCM3AP-AS1), antisense RNA [NR_002776]	A_23_P256694	2.3E-05	0.00139	-2.392	-1.258
XLOC_008088	603180829F1 NIH_MGC_121 Homo sapiens cDNA clone IMAGE:5245134 5', mRNA sequence [BI914892]	A_21_P0006520	3.2E-05	0.0017	-3.162	-1.661
LOC100272228	Homo sapiens uncharacterized LOC100131434 (LOC100131434), non-coding RNA [NR_027455]	A_33_P3290368	3.5E-05	0.00179	-2.244	-1.166
XLOC_011801	ALU6_HUMAN (P39193) Alu subfamily SP sequence contamination warning entry, partial (5%) [THC2629455]	A_21_P0008876	3.6E-05	0.00181	-5.509	-2.462
ZNF815	Homo sapiens zinc finger protein 815 (ZNF815), non-coding RNA [NR_023382]	A_33_P3357858	3.7E-05	0.00183	-2.566	-1.36
XLOC_014001	RST3117 Athersys RAGE Library Homo sapiens cDNA, mRNA sequence [BG184196]	A_21_P0010268	4.1E-05	0.00192	-6.323	-2.661

XLOC_l2_003610	HS1188J21 FSH primary response (LRPR1 homolog, rat) 1 {Homo sapiens} (exp=0; wgp=1; cg=0), partial (35%)	A_21_P0011117	4.1E-05	0.00193	-2.509	-1.327
C21orf67	Homo sapiens chromosome 21 open reading frame 67 (C21orf67), transcript variant 2, non-coding RNA	A_23_P211196	4.3E-05	0.00197	-2.802	-1.487
MGC2752	Homo sapiens CENPB DNA-binding domains containing 1 pseudogene (MGC2752), non-coding RNA [NR_026052]	A_33_P3399114	4.3E-05	0.00197	-2.066	-1.047
XLOC_001011	Unknown	A_21_P0001177	4.4E-05	0.00203	-2.436	-1.284
XLOC_l2_004315	BROAD Institute lincRNA (XLOC_l2_004315), lincRNA [TCONS_l2_00007925]	A_21_P0011258	4.7E-05	0.00208	-3.098	-1.631
XLOC_014003	BROAD Institute lincRNA (XLOC_014003), lincRNA	A_21_P0010269	5.7E-05	0.00233	-9.923	-3.311
A_19_P00316324	Homo sapiens uncharacterized LOC100506810 (LOC100506810), non-coding RNA [NR_038856]	A_19_P00316324	5.7E-05	0.00234	-7.939	-2.989
XLOC_000107	BROAD Institute lincRNA (XLOC_000107), lincRNA	A_21_P0000960	5.9E-05	0.00238	-3.932	-1.975
AACSL	Homo sapiens acetoacetyl-CoA synthetase pseudogene 1 (AACSP1), non-coding RNA [NR_024035]	A_33_P3302290	6.1E-05	0.00242	-3.718	-1.894
LOC100131347	Homo sapiens RAD52 motif 1 pseudogene (LOC100131347), non-coding RNA [NR_036551]	A_33_P3549091	6.1E-05	0.00242	-9.212	-3.204
XLOC_012505	BROAD Institute lincRNA (XLOC_012505), lincRNA	A_21_P0009193	6.3E-05	0.00248	-3.403	-1.767
RAET1K	Homo sapiens retinoic acid early transcript 1K pseudogene (RAET1K), non-coding RNA [NR_024045]	A_21_P0013163	6.4E-05	0.0025	-5.602	-2.486
LINC00173	Homo sapiens long intergenic non-protein coding RNA 173 (LINC00173), transcript variant 2, non-coding RNA	A_21_P0000535	6.5E-05	0.00252	-3.451	-1.787
XLOC_011618	BROAD Institute lincRNA (XLOC_011618), lincRNA	A_21_P0009091	6.9E-05	0.00263	-3.111	-1.637
XLOC_l2_000804	BROAD Institute lincRNA (XLOC_l2_000804), lincRNA [TCONS_l2_00001072]	A_21_P0010605	7E-05	0.00266	-3.967	-1.988
XLOC_l2_001929	Unknown	A_21_P0010867	7.3E-05	0.00271	-2.497	-1.32
LOC143666	Homo sapiens uncharacterized LOC143666 (LOC143666), non-coding RNA [NR_026967]	A_21_P0007153	7.7E-05	0.00278	-3.964	-1.987
XLOC_006223	BROAD Institute lincRNA (XLOC_006223), lincRNA	A_21_P0005421	8.1E-05	0.00286	-3.374	-1.754
LOC100131096	Homo sapiens uncharacterized LOC100131096 (LOC100131096), non-coding RNA [NR_040071]	A_24_P857624	8.1E-05	0.00287	-4.009	-2.003
XLOC_l2_014821	PREDICTED: Homo sapiens monofunctional C1-tetrahydrofolate synthase, mitochondrial-like	A_21_P0013699	8.7E-05	0.00299	-4.612	-2.205
LOC84989	Homo sapiens uncharacterized LOC84989 (LOC84989), non-coding RNA [NR_027182]	A_33_P3789382	8.9E-05	0.003	-2.601	-1.379
PSIMCT-1	Homo sapiens malignant T cell amplified sequence 1 pseudogene (PSIMCT-1), non-coding RNA [NR_003677]	A_33_P3296193	9.9E-05	0.00317	-3.508	-1.811

XLOC_l2_001089	BROAD Institute lincRNA (XLOC_l2_001089), lincRNA [TCONS_l2_00002639]	A_21_P0010772	0.0001	0.00318	-3.449	-1.786
AZFP	PREDICTED: Homo sapiens AML-associated zinc finger protein (AZFP), miscRNA [XR_109896]	A_33_P3213675	0.0001	0.00323	-3.92	-1.971
XLOC_014018	BROAD Institute lincRNA (XLOC_014018), lincRNA	A_21_P0010182	0.00011	0.00333	-12.102	-3.597
XLOC_l2_011204	BX104493 Soares fetal liver spleen 1NFLS Homo sapiens cDNA clone IMAGp998E24531, mRNA sequence	A_21_P0012781	0.00011	0.00333	-2.645	-1.403
XLOC_l2_012552	Q2Q5T5_MOUSE (Q2Q5T5) Embryonic stem cell-and germ cell-specific protein ESGP, complete [THC2642537]	A_21_P0013024	0.00011	0.00334	-6.246	-2.643
XLOC_l2_009539	BROAD Institute lincRNA (XLOC_l2_009539), lincRNA [TCONS_l2_00018033]	A_21_P0012298	0.00011	0.00335	-3.704	-1.889
A_19_P00321973	Q29HP5_DROPS (Q29HP5) GA14742-PA (Fragment), partial (9%) [THC2614488]	A_19_P00321973	0.00012	0.00349	-2.654	-1.408
XLOC_000101	O66122_STRCO (O66122) CprB (A-factor receptor homolog), partial (8%) [THC2662019]	A_21_P0001273	0.00013	0.00365	-3.466	-1.793
MAST4-AS1	PREDICTED: Homo sapiens MAST4 antisense RNA 1 (non-protein coding) (MAST4-AS1), miscRNA [XR_108604]	A_21_P0014149	0.00013	0.00376	-3.111	-1.637
XLOC_002612	AGENCOURT_10402397 NIH_MGC_82 Homo sapiens cDNA clone IMAGE:6617729 5', mRNA sequence	A_19_P00317368	0.00015	0.00399	-4.323	-2.112
XLOC_009437	BROAD Institute lincRNA (XLOC_009437), lincRNA	A_21_P0007352	0.00016	0.00409	-8.526	-3.092
HERC2P9	BROAD Institute lincRNA (XLOC_l2_004594), lincRNA [TCONS_l2_00008479]	A_21_P0011297	0.00016	0.00414	-2.176	-1.121
LOC541471	Homo sapiens uncharacterized LOC541471 (LOC541471), transcript variant 1, non-coding RNA [NR_015395]	A_33_P3269678	0.00016	0.00416	-2.198	-1.136
LOC644192	PREDICTED: Homo sapiens hypothetical LOC644192 (LOC644192), miscRNA [XR_109225]	A_21_P0014301	0.00017	0.00425	-2.444	-1.289
LINC00261	Homo sapiens long intergenic non-protein coding RNA 261 (LINC00261), non-coding RNA [NR_001558]	A_21_P0010016	0.00017	0.00429	-12.425	-3.635
XLOC_004775	RST35453 Athersys RAGE Library Homo sapiens cDNA, mRNA sequence [BG215776]	A_21_P0004376	0.00017	0.00432	-2.177	-1.122
XLOC_014002	BROAD Institute lincRNA (XLOC_014002), lincRNA	A_21_P0010318	0.00017	0.00437	-9.257	-3.21
XLOC_000175	BROAD Institute lincRNA (XLOC_000175), lincRNA	A_21_P0001291	0.00019	0.00455	-2.143	-1.099
XLOC_l2_013267	BROAD Institute lincRNA (XLOC_l2_013267), lincRNA [TCONS_l2_00025628]	A_21_P0013169	0.00021	0.00487	-2.49	-1.316
XLOC_006690	ik41b08.y1 HR85 islet Homo sapiens cDNA clone IMAGE: 5783390 5', mRNA sequence [BQ417053]	A_21_P0005647	0.00021	0.00491	-5.007	-2.324
XLOC_005339	BROAD Institute lincRNA (XLOC_005339), lincRNA	A_21_P0004896	0.00022	0.00497	-3.072	-1.619
XLOC_005116	BROAD Institute lincRNA (XLOC_005116), lincRNA	A_21_P0004622	0.00022	0.00498	-2.287	-1.194

XLOC_I2_006944	Q7R1K8_GIALA (Q7R1K8) GLP_28_72799_73185, partial (8%) [THC2750782]	A_21_P0011725	0.00022	0.00505	-2.144	-1.1
LOC100507312	PREDICTED: Homo sapiens hypothetical LOC100507312, transcript variant 1 (LOC100507312), miscRNA	A_21_P0010491	0.00022	0.00507	-2.922	-1.547
XLOC_001354	PREDICTED: Homo sapiens hypothetical LOC100506457 (LOC100506457), miscRNA [XR_108396]	A_21_P0001826	0.00023	0.00508	-4.882	-2.287
XLOC_003843	BX094246 Soares_testis_NHT Homo sapiens cDNA clone IMAGp998N201862, mRNA sequence [BX094246]	A_21_P0003881	0.00023	0.00512	-12.145	-3.602
LOC100132273	Homo sapiens uncharacterized LOC100132273 (LOC100132273), non-coding RNA [NR_034118]	A_33_P3440264	0.00023	0.00515	-3.088	-1.627
A_21_P0014431	PREDICTED: Homo sapiens hypothetical LOC100286925 (LOC100286925), miscRNA [XR_109748]	A_21_P0014431	0.00023	0.00516	-2.88	-1.526
LOC100652752	PREDICTED: Homo sapiens e3 ubiquitin-protein ligase HERC2-like (LOC100652752), miscRNA [XR_132609]	A_21_P0011466	0.00023	0.00516	-2.266	-1.18
XLOC_I2_012374	BROAD Institute lincRNA (XLOC_I2_012374), lincRNA [TCONS_I2_00025079]	A_19_P00801917	0.00025	0.00532	-2.059	-1.042
A_19_P00330814	Homo sapiens HOX transcript antisense RNA (non-protein coding) (HOTAIR), antisense RNA [NR_003716]	A_19_P00330814	0.00026	0.00552	-3.669	-1.875
XLOC_000746	TOP3_VIBCH (Q9KQF5) DNA topoisomerase 3 (DNA topoisomerase III) , partial (3%) [THC2543326]	A_21_P0001110	0.00027	0.00565	-2.035	-1.025
XLOC_I2_008203	Q33DX6_9HYME (Q33DX6) Ecdysone receptor B isoform (Fragment), partial (16%) [THC2691143]	A_21_P0011950	0.00028	0.00572	-2.905	-1.539
XLOC_I2_015849	BX111592 Soares_testis_NHT Homo sapiens cDNA clone IMAGp998D162621, mRNA sequence [BX111592]	A_21_P0013836	0.00029	0.00586	-4.108	-2.039
XLOC_I2_008888	AGENCOURT_10580380 NIH_MGC_128 Homo sapiens cDNA clone IMAGE:6710146 5', mRNA sequence	A_21_P0012143	0.00031	0.00615	-4.444	-2.152
LOC100506001	PREDICTED: Homo sapiens hypothetical LOC100506001 (LOC100506001), miscRNA [XR_109348]	A_21_P0014341	0.00032	0.00621	-3.956	-1.984
XLOC_I2_015476	BROAD Institute lincRNA (XLOC_I2_015476), lincRNA [TCONS_I2_00030169]	A_21_P0013756	0.00032	0.00621	-2.042	-1.03
LOC170425	Homo sapiens uncharacterized LOC170425 (LOC170425), non-coding RNA [NR_038220]	A_33_P3718352	0.00032	0.00625	-2.77	-1.47
XLOC_002174	Q86U25_HUMAN (Q86U25) Full-length cDNA clone CS0DA007YG23 of Neuroblastoma of Homo sapiens	A_21_P0001792	0.00033	0.00633	-6.761	-2.757
AP1B1P1	Homo sapiens adaptor-related protein complex 1, beta 1 subunit pseudogene 1 (AP1B1P1), non-coding RNA	A_21_P0012322	0.00033	0.00643	-2.079	-1.056
XLOC_012578	AF014461 EXO70 protein {Mus musculus} (exp=-1; wgp=0; cg=0), partial (21%) [THC2780952]	A_21_P0009365	0.00036	0.00667	-5.788	-2.533

LOC100505987	PREDICTED: Homo sapiens hypothetical LOC100505987 (LOC100505987), miscRNA [XR_110833]	A_21_P0014632	0.00037	0.00682	-3.089	-1.627
A_19_P00802154	PREDICTED: Homo sapiens hypothetical LOC100652827 (LOC100652827), miscRNA [XR_132519]	A_19_P00802154	0.00039	0.00702	-4.509	-2.173
ENST00000398976	Uncharacterized protein [Source:UniProtKB/TrEMBL;Acc:E7ES44]	A_33_P3355783	0.00039	0.00704	-2.567	-1.36
A_21_P0014087	PREDICTED: Homo sapiens hypothetical LOC100506405, transcript variant 2 (LOC100506405), miscRNA	A_21_P0014087	0.00041	0.00725	-4.336	-2.116
XLOC_l2_009320	BROAD Institute lincRNA (XLOC_l2_009320), lincRNA [TCONS_l2_00018190]	A_21_P0012318	0.00041	0.0073	-3.232	-1.693
XLOC_004677	BROAD Institute lincRNA (XLOC_004677), lincRNA	A_21_P0004083	0.00042	0.00736	-2.194	-1.133
KIAA1529	Homo sapiens SUGT1-1300002K09Rik pseudogene (LOC100499484), non-coding RNA [NR_036526]	A_33_P3362409	0.00042	0.00741	-3.826	-1.936
LOC100132356	Homo sapiens uncharacterized LOC100132356 (LOC100132356), non-coding RNA [NR_034127]	A_24_P942017	0.00042	0.00745	-2.411	-1.269
HCG4P6	Homo sapiens HLA complex group 4B (non-protein coding) (HCG4B), non-coding RNA [NR_001317]	A_23_P359376	0.00043	0.00756	-2.361	-1.24
LOC100289092	PREDICTED: Homo sapiens hypothetical LOC100289092 (LOC100289092), miscRNA [XR_109278]	A_21_P0014321	0.00045	0.00772	-3.579	-1.84
A_19_P00321166	Homo sapiens uncharacterized LOC100132356 (LOC100132356), non-coding RNA [NR_034127]	A_19_P00321166	0.00047	0.00793	-2.343	-1.229
THC2657157	PREDICTED: Homo sapiens hypothetical LOC100652995 (LOC100652995), miscRNA [XR_132578]	A_33_P3282364	0.00048	0.00801	-3.425	-1.776
XLOC_l2_002033	BROAD Institute lincRNA (XLOC_l2_002033), lincRNA [TCONS_l2_00004216]	A_21_P0010950	0.00049	0.00812	-2.832	-1.502
XLOC_004940	BROAD Institute lincRNA (XLOC_004940), lincRNA	A_21_P0004426	0.00049	0.00819	-3.232	-1.692
XLOC_006353	BROAD Institute lincRNA (XLOC_006353), lincRNA	A_21_P0005470	0.0005	0.00822	-2.084	-1.059
XLOC_001329	BROAD Institute lincRNA (XLOC_001329), lincRNA	A_21_P0002569	0.00052	0.00847	-3.271	-1.71
XLOC_000677	Unknown	A_21_P0001087	0.00053	0.0085	-3.886	-1.958
XLOC_l2_010267	BROAD Institute lincRNA (XLOC_l2_010267), lincRNA [TCONS_l2_00019377]	A_21_P0012452	0.00054	0.00863	-6.158	-2.623
LOC339352	PREDICTED: Homo sapiens putative ATP-binding domain-containing protein 3-like protein-like (LOC339352),	A_33_P3383059	0.00054	0.00866	-2.257	-1.174
XLOC_l2_004640	BROAD Institute lincRNA (XLOC_l2_004640), lincRNA [TCONS_l2_00008604]	A_21_P0011318	0.00054	0.00866	-4.404	-2.139
LOC100130547	PREDICTED: Homo sapiens hypothetical LOC100130547 (LOC100130547), miscRNA [XR_113275]	A_33_P3259938	0.00055	0.00881	-4.531	-2.18

LOC286467	Homo sapiens family with sequence similarity 195, member A pseudogene (LOC286467), non-coding RNA	A_21_P0013832	0.00059	0.00917	-3.541	-1.824
LOC100507564	Homo sapiens uncharacterized LOC100507564 (LOC100507564), non-coding RNA [NR_038953]	A_21_P0000851	0.00061	0.00935	-2.892	-1.532
LOC100653259	PREDICTED: Homo sapiens e3 ubiquitin-protein ligase HERC2-like (LOC100653259), miscRNA [XR_133099]	A_21_P0011471	0.00062	0.00946	-2.817	-1.494
HERC2P4	Homo sapiens hect domain and RLD 2 pseudogene 4 (HERC2P4), non-coding RNA [NR_002827]	A_32_P139738	0.00065	0.00968	-2.277	-1.187
XLOC_l2_013001	BROAD Institute lincRNA (XLOC_l2_013001), lincRNA [TCONS_l2_00025392]	A_19_P00808126	0.00066	0.0098	-1.891	-0.919
XLOC_013154	BROAD Institute lincRNA (XLOC_013154), lincRNA	A_21_P0009666	0.00067	0.00988	-5.39	-2.43
XLOC_005340	BROAD Institute lincRNA (XLOC_005340), lincRNA	A_21_P0004658	0.00068	0.00999	-3.887	-1.959
XLOC_l2_004594	BROAD Institute lincRNA (XLOC_l2_004594), lincRNA [TCONS_l2_00008470]	A_21_P0011293	0.00069	0.0101	-2.181	-1.125
XLOC_l2_015821	BROAD Institute lincRNA (XLOC_l2_015821), lincRNA [TCONS_l2_00030598]	A_21_P0013831	0.00069	0.0101	-3.186	-1.672
XLOC_l2_014048	BROAD Institute lincRNA (XLOC_l2_014048), lincRNA [TCONS_l2_00027518]	A_21_P0013456	0.00069	0.0101	-2.075	-1.053
XLOC_l2_007569	Unknown	A_21_P0011854	0.00069	0.0101	-4.225	-2.079
XLOC_012017	DB118899 THYMU2 Homo sapiens cDNA clone THYMU2026314 5', mRNA sequence [DB118899]	A_21_P0009067	0.0007	0.0102	-2.373	-1.247
LOC100507654	PREDICTED: Homo sapiens hypothetical LOC100507654 (LOC100507654), miscRNA [XR_109152]	A_21_P0014287	0.0007	0.0102	-3.701	-1.888
LOC100130219	PREDICTED: Homo sapiens hypothetical LOC100130219 (LOC100130219), miscRNA [XR_109115]	A_33_P3242109	0.00072	0.0103	-2.302	-1.203
LOC440335	Homo sapiens uncharacterized LOC440335 (LOC440335), transcript variant 2, non-coding RNA [NR_029454]	A_24_P229884	0.00073	0.0104	-2.578	-1.366
AK096286	Homo sapiens uncharacterized LOC100128881 (LOC100128881), non-coding RNA [NR_036480]	A_33_P3241299	0.00072	0.0104	-3.629	-1.86
LOC442245	Homo sapiens glutathione S-transferase mu 2 (muscle) pseudogene 1 (GSTM2P1), non-coding RNA [NR_002932]	A_23_P58869	0.00074	0.0105	-2.178	-1.123
EP400NL	Homo sapiens EP400 N-terminal like (EP400NL), non-coding RNA [NR_003290]	A_33_P3258953	0.00074	0.0105	-2.736	-1.452
XLOC_l2_011098	BROAD Institute lincRNA (XLOC_l2_011098), lincRNA [TCONS_l2_00021859]	A_21_P0012773	0.00074	0.0105	-2.756	-1.463
LOC100506660	Homo sapiens uncharacterized LOC100506660 (LOC100506660), non-coding RNA [NR_038927]	A_21_P0007523	0.00075	0.0107	-2.304	-1.204

BC071797	Homo sapiens ankyrin repeat domain 20 family, member A9, pseudogene (ANKRD20A9P), non-coding RNA	A_32_P13151	0.00076	0.0108	-3.237	-1.695
XLOC_l2_012081	Unknown	A_21_P0012910	0.00077	0.0108	-2.308	-1.207
A_19_P00809030	Q8IVV4_HUMAN (Q8IVV4) TPTEps1 protein (Fragment), partial (23%) [THC2604663]	A_19_P00809030	0.00078	0.0109	-2.553	-1.352
XLOC_014513	ALU6_HUMAN (P39193) Alu subfamily SP sequence contamination warning entry, partial (9%) [THC2502608]	A_21_P0010492	0.00079	0.011	-2.199	-1.137
XLOC_l2_001085	Unknown	A_21_P0010646	0.0008	0.011	-4.771	-2.254
LOC100131132	Uncharacterized proteincDNA FLJ43696 fis, clone TBAES2007964 [Source:UniProtKB/TrEMBL;Acc:Q6ZUH9]	A_33_P3267198	0.00081	0.0112	-2.252	-1.171
LOC201617	Homo sapiens uncharacterized LOC201617 (LOC201617), non-coding RNA [NR_038221]	A_33_P3585328	0.00082	0.0112	-5.73	-2.519
XLOC_l2_013580	BROAD Institute lincRNA (XLOC_l2_013580), lincRNA [TCONS_l2_00026180]	A_21_P0013264	0.00085	0.0115	-3.855	-1.947
XLOC_l2_004840	602291186F1 NIH_MGC_85 Homo sapiens cDNA clone IMAGE:4386188 5', mRNA sequence [BG107090]	A_21_P0011346	0.00086	0.0116	-7.58	-2.922
A_19_P00809372	PREDICTED: Homo sapiens hypothetical LOC100505634 (LOC100505634), miscRNA [XR_109905]	A_19_P00809372	0.00089	0.0119	-3.167	-1.663
LOC284889	Homo sapiens uncharacterized LOC284889 (LOC284889), non-coding RNA [NR_038911]	A_33_P3351894	0.00092	0.0121	-2.034	-1.025
LOC283922	Homo sapiens pyruvate dehydrogenase phosphatase regulatory subunit pseudogene (LOC283922), non-coding	A_33_P3246438	0.00094	0.0123	-2.111	-1.078
BACE1-AS	Homo sapiens BACE1 antisense RNA (non-protein coding) (BACE1-AS), antisense RNA [NR_037803]	A_21_P0000686	0.00097	0.0125	-2.31	-1.208
XLOC_l2_009614	Q70WD9_HUMAN (Q70WD9) Maab3 protein (Fragment), partial (9%) [THC2535972]	A_21_P0012365	0.00097	0.0125	-2.685	-1.425
XLOC_000778	BROAD Institute lincRNA (XLOC_000778), lincRNA	A_21_P0001121	0.00098	0.0126	-3.545	-1.826
XLOC_003839	BROAD Institute lincRNA (XLOC_003839), lincRNA	A_21_P0003704	0.00098	0.0126	-2.11	-1.077
LOC100130673	Homo sapiens phosphoribosyl pyrophosphate synthetase 2 pseudogene (LOC100130673), non-coding RNA	A_33_P3380071	0.001	0.0127	-4.103	-2.037
A_21_P0014351	PREDICTED: Homo sapiens hypothetical LOC100506325 (LOC100506325), miscRNA [XR_109396]	A_21_P0014351	0.001	0.0127	-5.2	-2.378
XLOC_000391	DKFZp686F2244_r1 686 (synonym: hlcc3) Homo sapiens cDNA clone DKFZp686F2244 5', mRNA sequence	A_21_P0001016	0.00102	0.0129	-2.197	-1.136
P2RX6P	Homo sapiens purinergic receptor P2X, ligand-gated ion channel, 6 pseudogene (P2RX6P), non-coding RNA	A_21_P0000292	0.00104	0.0131	-4.138	-2.049
NCRNA00107	Homo sapiens PPP2R3B antisense RNA 1 (non-protein coding) (PPP2R3B-AS1), transcript variant 1, non-coding	A_33_P3722568	0.00107	0.0133	-2.044	-1.032

XLOC_009146	BROAD Institute lincRNA (XLOC_009146), lincRNA	A_21_P0007215	0.00107	0.0133	-2.969	-1.57
C15orf51	Homo sapiens DNM1 pseudogene 46 (DNM1P46), non-coding RNA [NR_003260]	A_24_P50091	0.00109	0.0134	-2.815	-1.493
NCRNA00173	Homo sapiens long intergenic non-protein coding RNA 173 (LINC00173), transcript variant 1, non-coding RNA	A_33_P3331511	0.00108	0.0134	-2.711	-1.439
XLOC_009191	Q3SGJ5_THIDA (Q3SGJ5) Septum formation inhibitor MinC, partial (5%) [THC2622951]	A_21_P0007229	0.00109	0.0134	-3.39	-1.761
FLJ40504	Homo sapiens keratin 18 pseudogene 55 (KRT18P55), non-coding RNA [NR_028334]	A_23_P373708	0.00113	0.0137	-2.183	-1.127
hCG_2042718	PREDICTED: Homo sapiens ankyrin repeat domain-containing protein 20B-like (LOC644339), miscRNA	A_33_P3279660	0.00116	0.0139	-2.248	-1.169
A_21_P0014746	PREDICTED: Homo sapiens hypothetical LOC100652965 (LOC100652965), miscRNA [XR_132480]	A_21_P0014746	0.00116	0.0139	-5.99	-2.583
XLOC_010152	602137201F1 NIH_MGC_83 Homo sapiens cDNA clone IMAGE:4273440 5', mRNA sequence [BF674433]	A_21_P0007740	0.00116	0.0139	-7.521	-2.911
XLOC_004325	BROAD Institute lincRNA (XLOC_004325), lincRNA	A_21_P0003924	0.00116	0.0139	-1.507	-0.592
XLOC_009897	BROAD Institute lincRNA (XLOC_009897), lincRNA	A_21_P0007637	0.00115	0.0139	-4.336	-2.116
XLOC_000721	BROAD Institute lincRNA (XLOC_000721), lincRNA	A_21_P0001471	0.00115	0.0139	-2.238	-1.162
XLOC_l2_008163	BROAD Institute lincRNA (XLOC_l2_008163), lincRNA [TCONS_l2_00014743]	A_21_P0011941	0.00117	0.014	-2.198	-1.136
XLOC_006580	EST00103 Image cDNA Library pCMV-SPORT6 Homo sapiens cDNA clone IMAGE:3925213, mRNA sequence	A_21_P0005328	0.00119	0.0142	-2.387	-1.255
A_19_P00316423	PREDICTED: Homo sapiens L antigen family member 3-like (LOC646808), miscRNA [XR_132560]	A_19_P00316423	0.00122	0.0144	-2.179	-1.124
TTTY16	Homo sapiens testis-specific transcript, Y-linked 16 (non-protein coding) (TTTY16), non-coding RNA [NR_001552]	A_33_P3349495	0.00122	0.0144	-2.205	-1.141
XLOC_l2_011265	AGENCOURT_10401902 NIH_MGC_82 Homo sapiens cDNA clone IMAGE:6617809 5', mRNA sequence	A_21_P0012794	0.00123	0.0144	-5.335	-2.416
XLOC_004998	BROAD Institute lincRNA (XLOC_004998), lincRNA	A_21_P0004181	0.00125	0.0146	-3.427	-1.777
LOC100652738	PREDICTED: Homo sapiens hypothetical LOC100652738 (LOC100652738), miscRNA [XR_132901]	A_21_P0014904	0.00127	0.0147	-2.048	-1.034
LOC100505648	BROAD Institute lincRNA (XLOC_l2_004898), lincRNA [TCONS_l2_00009502]	A_21_P0011409	0.00129	0.0149	-2.255	-1.173
LOC646808	PREDICTED: Homo sapiens L antigen family member 3-like (LOC646808), miscRNA [XR_132560]	A_33_P3339253	0.00134	0.0152	-2.228	-1.156
psiTPTE22	Homo sapiens TPTE pseudogene (psiTPTE22), non-coding RNA [NR_001591]	A_33_P3213155	0.00136	0.0154	-2.446	-1.29

C8orf51	Homo sapiens chromosome 8 open reading frame 51 (C8orf51), non-coding RNA [NR_026785]	A_23_P71503	0.00142	0.0158	-2.815	-1.493
LOC441208	Homo sapiens zinc and ring finger 2 pseudogene 1 (ZNRF2P1), non-coding RNA [NR_003502]	A_24_P145009	0.00144	0.0159	-2.898	-1.535
XLOC_l2_003885	BROAD Institute lincRNA (XLOC_l2_003885), lincRNA [TCONS_l2_00007481]	A_21_P0011209	0.00147	0.0161	-2.585	-1.37
XLOC_002362	qe05g10.x1 Soares_testis_NHT Homo sapiens cDNA clone IMAGE:1738146 3', mRNA sequence [AI140623]	A_21_P0002118	0.0015	0.0163	-2.024	-1.017
XLOC_l2_012071	BROAD Institute lincRNA (XLOC_l2_012071), lincRNA [TCONS_l2_00022934]	A_21_P0012909	0.00154	0.0165	-2.853	-1.512
LOC100506732	PREDICTED: Homo sapiens hypothetical LOC100506732, transcript variant 2 (LOC100506732), miscRNA	A_21_P0014576	0.00157	0.0168	-2.727	-1.447
XLOC_002613	DA963975 STOMA2 Homo sapiens cDNA clone STOMA2001973 5', mRNA sequence [DA963975]	A_19_P00317885	0.0016	0.0169	-3.772	-1.915
XLOC_011837	BROAD Institute lincRNA (XLOC_011837), lincRNA	A_21_P0009119	0.00159	0.0169	-2.233	-1.159
XLOC_l2_015418	BROAD Institute lincRNA (XLOC_l2_015418), lincRNA [TCONS_l2_00029647]	A_21_P0013688	0.00163	0.0172	-5.415	-2.437
LOC100506314	Homo sapiens uncharacterized LOC100506314 (LOC100506314), non-coding RNA [NR_038920]	A_21_P0000838	0.00171	0.0177	-2.421	-1.276
XLOC_005167	602599163F1 NIH_MGC_87 Homo sapiens cDNA clone IMAGE:4708023 5', mRNA sequence [BG576820]	A_21_P0004632	0.00174	0.0178	-4.87	-2.284
XLOC_006925	Unknown	A_19_P00316753	0.00172	0.0178	-2.41	-1.269
HNRNPH1	HUM49KDA hnRNP H {Homo sapiens} (exp=-1; wgp=0; cg=0), partial (60%) [THC2585525]	A_21_P0004082	0.00178	0.0181	-2.585	-1.37
LOC100652757	PREDICTED: Homo sapiens hypothetical LOC100652757 (LOC100652757), miscRNA [XR_132878]	A_21_P0014894	0.0018	0.0183	-2.66	-1.412
XLOC_l2_005314	BROAD Institute lincRNA (XLOC_l2_005314), lincRNA [TCONS_l2_00009861]	A_21_P0011448	0.00183	0.0184	-2.85	-1.511
ZNF252	Homo sapiens zinc finger protein 252 (ZNF252), non-coding RNA [NR_023392]	A_23_P412186	0.00187	0.0187	-2.669	-1.416
XLOC_014158	BROAD Institute lincRNA (XLOC_014158), lincRNA	A_21_P0010392	0.00187	0.0187	-2.124	-1.086
XLOC_l2_015641	BROAD Institute lincRNA (XLOC_l2_015641), lincRNA [TCONS_l2_00030390]	A_21_P0013798	0.00187	0.0187	-2.603	-1.38
LOC100133315	Homo sapiens transient receptor potential cation channel, subfamily C, member 2-like (LOC100133315), non-coding	A_32_P437876	0.00188	0.0188	-3.699	-1.887
XLOC_l2_012919	BROAD Institute lincRNA (XLOC_l2_012919), lincRNA [TCONS_l2_00024658]	A_21_P0013068	0.00191	0.019	-2.68	-1.422
XLOC_000166	BROAD Institute lincRNA (XLOC_000166), lincRNA	A_21_P0001676	0.00198	0.0194	-5.223	-2.385

XLOC_012551	BROAD Institute lincRNA (XLOC_012551), lincRNA	A_21_P0009346	0.002	0.0195	-3.433	-1.78
LOC100506523	PREDICTED: Homo sapiens hypothetical LOC100506523 (LOC100506523), miscRNA [XR_109504]	A_21_P0014375	0.00202	0.0196	-2.102	-1.071
XLOC_006933	BROAD Institute lincRNA (XLOC_006933), lincRNA	A_21_P0005765	0.00204	0.0197	-5.684	-2.507
LOC100506201	PREDICTED: Homo sapiens hypothetical LOC100506201, transcript variant 4 (LOC100506201), miscRNA	A_21_P0014804	0.00206	0.0198	-3.208	-1.682
XLOC_008528	BROAD Institute lincRNA (XLOC_008528), lincRNA	A_21_P0006686	0.00214	0.0203	-3.434	-1.78
XLOC_012143	BROAD Institute lincRNA (XLOC_012143), lincRNA	A_21_P0009228	0.00215	0.0203	-2.14	-1.098
A_21_P0014761	PREDICTED: Homo sapiens hypothetical LOC100505644, transcript variant 2 (LOC100505644), miscRNA	A_21_P0014761	0.0022	0.0206	-2.246	-1.167
A_19_P00805840	Homo sapiens zinc finger protein 37B, pseudogene (ZNF37BP), non-coding RNA [NR_026777]	A_19_P00805840	0.00222	0.0207	-2.529	-1.339
FLJ34077	Homo sapiens uncharacterized LOC728558 (LOC728558), non-coding RNA [NR_038444]	A_33_P3400152	0.00223	0.0207	-3.112	-1.638
LOC100506174	PREDICTED: Homo sapiens hypothetical LOC100506174 (LOC100506174), miscRNA [XR_109495]	A_21_P0014372	0.00222	0.0207	-2.403	-1.265
XLOC_l2_012745	BROAD Institute lincRNA (XLOC_l2_012745), lincRNA [TCONS_l2_00024454]	A_21_P0013041	0.00235	0.0214	-4.096	-2.034
XLOC_012259	BROAD Institute lincRNA (XLOC_012259), lincRNA	A_21_P0009262	0.00253	0.0223	-2.866	-1.519
XLOC_012162	BC015704 joined to JAZF1 {Homo sapiens} (exp=-1; wgp=0; cg=0), partial (17%) [THC2573582]	A_21_P0009385	0.00254	0.0223	-2.754	-1.461
XLOC_013124	BROAD Institute lincRNA (XLOC_013124), lincRNA	A_21_P0009728	0.00255	0.0224	-2.086	-1.061
AQP7P1	Homo sapiens aquaporin 7 pseudogene 1 (AQP7P1), non-coding RNA [NR_002817]	A_33_P3245290	0.00261	0.0227	-7.885	-2.979
XLOC_013308	BROAD Institute lincRNA (XLOC_013308), lincRNA	A_21_P0009803	0.00264	0.0229	-2.356	-1.236
XLOC_l2_005793	Q7SG75_NEUCR (Q7SG75) Predicted protein, partial (3%) [THC2699069]	A_21_P0011544	0.00264	0.0229	-7.284	-2.865
XLOC_007858	BROAD Institute lincRNA (XLOC_007858), lincRNA	A_21_P0006321	0.00271	0.0232	-3.822	-1.935
XLOC_l2_008596	Q9H4R4_HUMAN (Q9H4R4) OTTHUMP00000030509, partial (63%) [THC2622778]	A_21_P0012114	0.00271	0.0232	-2.132	-1.092
MYO15B	Homo sapiens myosin XVB pseudogene (MYO15B), non-coding RNA [NR_003587]	A_32_P475513	0.00274	0.0233	-2.829	-1.5
A_19_P00316144	ALU2_HUMAN (P39189) Alu subfamily SB sequence contamination warning entry, partial (16%) [THC2577317]	A_19_P00316144	0.00273	0.0233	-4.038	-2.014
LOC728975	PREDICTED: Homo sapiens hypothetical LOC728975 (LOC728975), miscRNA [XR_110914]	A_33_P3337019	0.00273	0.0233	-2.33	-1.22
XLOC_005495	UI-E-EJ0-aio-k-13-0-UI.s1 UI-E-EJ0 Homo sapiens cDNA clone UI-E-EJ0-aio-k-13-0-UI 3', mRNA sequence	A_21_P0004693	0.00276	0.0234	-3.574	-1.838

XLOC_009509	PPN1_CANGA (Q6FMQ0) Endopolyphosphatase , partial (3%) [THC2656073]	A_21_P0007376	0.00276	0.0234	-2.135	-1.095
XLOC_002569	BROAD Institute lincRNA (XLOC_002569), lincRNA	A_19_P00318938	0.0028	0.0236	-2.643	-1.402
XLOC_006985	Q306F7_HUMAN (Q306F7) Down syndrome encephalopathy related protein 1, partial (15%)	A_21_P0005793	0.00291	0.0242	-2.323	-1.216
A_21_P0014162	PREDICTED: Homo sapiens hypothetical LOC100505730, transcript variant 2 (LOC100505730), miscRNA	A_21_P0014162	0.00299	0.0246	-2.17	-1.118
XLOC_014097	BROAD Institute lincRNA (XLOC_014097), lincRNA	A_21_P0010322	0.00303	0.0247	-2.083	-1.059
XLOC_013429	PREDICTED: Homo sapiens hepatocellular carcinoma-associated antigen HCA25a (LOC100506634), miscRNA	A_21_P0009778	0.00305	0.0248	-1.853	-0.89
XLOC_000837	AGENCOURT_10607577 NIH_MGC_126 Homo sapiens cDNA clone IMAGE:6725222 5', mRNA sequence	A_21_P0001132	0.00306	0.0249	-6.695	-2.743
XLOC_l2_008910	BROAD Institute lincRNA (XLOC_l2_008910), lincRNA [TCONS_l2_00016931]	A_21_P0012166	0.00306	0.0249	-4.148	-2.053
SNORA58	Homo sapiens small nucleolar RNA, H/ACA box 58 (SNORA58), small nucleolar RNA [NR_002985]	A_21_P0000339	0.00309	0.025	-2.054	-1.038
XLOC_l2_006994	BROAD Institute lincRNA (XLOC_l2_006994), lincRNA [TCONS_l2_00013005]	A_21_P0011735	0.00311	0.0251	-12.714	-3.668
A_19_P00320927	Homo sapiens uncharacterized LOC100292680 (LOC100292680), non-coding RNA [NR_028415]	A_19_P00320927	0.00312	0.0252	-5.606	-2.487
A_19_P00322939	Homo sapiens Pvt1 oncogene (non-protein coding) (PVT1), non-coding RNA [NR_003367]	A_19_P00322939	0.00315	0.0253	-2.66	-1.411
XLOC_012972	BROAD Institute lincRNA (XLOC_012972), lincRNA	A_21_P0009661	0.00323	0.0256	-2.619	-1.389
XLOC_l2_005187	BROAD Institute lincRNA (XLOC_l2_005187), lincRNA [TCONS_l2_00009686]	A_21_P0011432	0.00322	0.0256	-5.033	-2.331
ABCA11P	Homo sapiens ATP-binding cassette, sub-family A (ABC1), member 11, pseudogene (ABCA11P), non-coding RNA	A_23_P92602	0.00329	0.0258	-2.904	-1.538
XLOC_008370	BROAD Institute lincRNA (XLOC_008370), lincRNA	A_19_P00315529	0.00331	0.0259	-2.011	-1.008
LOC100505634	PREDICTED: Homo sapiens hypothetical LOC100505634 (LOC100505634), miscRNA [XR_109905]	A_21_P0014456	0.00333	0.026	-3.208	-1.681
FLJ43315	Homo sapiens asparagine synthetase pseudogene (FLJ43315), non-coding RNA [NR_033856]	A_21_P0010773	0.00347	0.0266	-2.721	-1.444
XLOC_013347	BROAD Institute lincRNA (XLOC_013347), lincRNA	A_21_P0009805	0.00354	0.0269	-2.089	-1.063
XLOC_l2_010636	BROAD Institute lincRNA (XLOC_l2_010636), lincRNA [TCONS_l2_00020480]	A_21_P0012581	0.00355	0.0269	-1.017	-0.025
LOC100506496	PREDICTED: Homo sapiens hypothetical LOC100506496 (LOC100506496), miscRNA [XR_110214]	A_21_P0014528	0.00354	0.0269	-2.84	-1.506

XLOC_l2_000727	BROAD Institute lincRNA (XLOC_l2_000727), lincRNA [TCONS_l2_00002393]	A_21_P0010752	0.00354	0.0269	-2.667	-1.415
LOC100130964	PREDICTED: Homo sapiens similar to hCG2045185 (LOC100130964), miscRNA [XR_132889]	A_32_P928190	0.00358	0.027	-2.674	-1.419
LOC100507351	Homo sapiens uncharacterized LOC100507351 (LOC100507351), non-coding RNA [NR_040050]	A_21_P0009180	0.00357	0.027	-3.486	-1.802
A_19_P00323005	ALU5_HUMAN (P39192) Alu subfamily SC sequence contamination warning entry, partial (26%) [THC2590176]	A_19_P00323005	0.0036	0.0271	-2.584	-1.369
XLOC_009494	BROAD Institute lincRNA (XLOC_009494), lincRNA	A_21_P0007368	0.00359	0.0271	-3.005	-1.587
LOC100506599	Homo sapiens uncharacterized LOC100506599 (LOC100506599), non-coding RNA [NR_038969]	A_21_P0013745	0.00364	0.0272	-2.388	-1.256
XLOC_012004	BROAD Institute lincRNA (XLOC_012004), lincRNA	A_21_P0009056	0.00367	0.0274	-1.707	-0.771
A_19_P00811286	Homo sapiens uncharacterized LOC441094 (FLJ42709), non-coding RNA [NR_021490]	A_19_P00811286	0.0038	0.028	-3.138	-1.65
XLOC_l2_015037	BROAD Institute lincRNA (XLOC_l2_015037), lincRNA [TCONS_l2_00029078]	A_19_P00327354	0.00383	0.0281	-2.382	-1.252
XLOC_006151	BROAD Institute lincRNA (XLOC_006151), lincRNA	A_21_P0005395	0.00386	0.0282	-2.082	-1.058
LOC100128278	PREDICTED: Homo sapiens hypothetical LOC100128278 (LOC100128278), miscRNA [XR_110306]	A_33_P3290303	0.00387	0.0283	-2.381	-1.252
LOC100335030	Homo sapiens FGFR1 oncogene partner 2 pseudogene (LOC100335030), non-coding RNA [NR_033267]	A_33_P3334826	0.00393	0.0285	-5.502	-2.46
LOC100507464	PREDICTED: Homo sapiens hypothetical LOC100507464 (LOC100507464), miscRNA [XR_108905]	A_21_P0014221	0.00394	0.0286	-2.824	-1.498
XLOC_l2_015160	BROAD Institute lincRNA (XLOC_l2_015160), lincRNA [TCONS_l2_00029272]	A_21_P0013628	0.00398	0.0287	-2.235	-1.16
A_19_P00801885	PREDICTED: Homo sapiens hCG1815504 (LOC440132), miscRNA [XR_132736]	A_19_P00801885	0.00404	0.029	-21.01	-4.393
XLOC_003710	601843220F1 NIH_MGC_54 Homo sapiens cDNA clone IMAGE:4063925 5', mRNA sequence [BF184174]	A_21_P0003410	0.00406	0.0291	-10.201	-3.351
LOC154822	BROAD Institute lincRNA (XLOC_006322), lincRNA	A_21_P0005276	0.00409	0.0292	-3.801	-1.927
A_19_P00322932	Homo sapiens uncharacterized LOC100506190 (LOC100506190), non-coding RNA [NR_038955]	A_19_P00322932	0.00416	0.0294	-2.113	-1.079
LOC440300	Homo sapiens chondroitin sulfate proteoglycan 4 pseudogene (LOC440297), non-coding RNA [NR_033579]	A_33_P3369029	0.00416	0.0294	-2.876	-1.524
XLOC_011152	BROAD Institute lincRNA (XLOC_011152), lincRNA	A_21_P0008551	0.00429	0.0298	-4.215	-2.076
LOC389834	Homo sapiens ankyrin repeat domain 57 pseudogene (LOC389834), non-coding RNA [NR_027420]	A_33_P3247534	0.00434	0.03	-1.572	-0.652
XLOC_000915	BROAD Institute lincRNA (XLOC_000915), lincRNA	A_21_P0001546	0.00437	0.0301	-2.066	-1.047

A_19_P00811533	Homo sapiens uncharacterized LOC440584 (FLJ32224), non-coding RNA [NR_033967]	A_19_P00811533	0.00443	0.0303	-4.119	-2.042
C6orf164	Homo sapiens chromosome 6 open reading frame 164 (C6orf164), non-coding RNA [NR_026784]	A_33_P3372674	0.00442	0.0303	-3.31	-1.727
AX748330	PREDICTED: Homo sapiens hypothetical locus LOC692247 (LOC692247), miscRNA [XR_109057]	A_33_P3589543	0.00442	0.0303	-3.076	-1.621
XLOC_006544	BROAD Institute lincRNA (XLOC_006544), lincRNA	A_21_P0005320	0.00443	0.0303	-2.021	-1.015
FAM95B1	family with sequence similarity 95, member B1 [Source:HGNC Symbol;Acc:32318] [ENST00000455995]	A_33_P3256287	0.00446	0.0304	-2.645	-1.404
LOC100009676	Homo sapiens uncharacterized LOC100009676 (LOC100009676), non-coding RNA [NR_024407]	A_23_P306479	0.00449	0.0305	-2.354	-1.235
XLOC_000133	BROAD Institute lincRNA (XLOC_000133), lincRNA	A_21_P0001671	0.00449	0.0305	-3.177	-1.668
XLOC_011383	BROAD Institute lincRNA (XLOC_011383), lincRNA	A_21_P0008695	0.00478	0.0316	-5.183	-2.374
A_19_P00320749	Homo sapiens Pvt1 oncogene (non-protein coding) (PVT1), non-coding RNA [NR_003367]	A_19_P00320749	0.00482	0.0317	-2.909	-1.541
A_19_P00318577	Q86TZ0_HUMAN (Q86TZ0) Full-length cDNA clone CS0DC023YN15 of Neuroblastoma of Homo sapiens (human) (Fragment), complete [THC2506480]	A_19_P00318577	0.00485	0.0318	-3.228	-1.691
XLOC_005663	Q3PAT9_PARDE (Q3PAT9) Amino acid ABC transporter, permease protein, 3-TM region, His/Glu/Gln/Arg/opine,	A_21_P0004742	0.0049	0.032	-2.964	-1.567
RPS15AP10	Homo sapiens ribosomal protein S15a pseudogene 10 (RPS15AP10), non-coding RNA [NR_026768]	A_33_P3357049	0.00493	0.0321	-2.061	-1.043
LOC100505471	PREDICTED: Homo sapiens putative uncharacterized protein encoded by NCRNA00269-like (LOC100505471),	A_21_P0014838	0.00494	0.0322	-2.228	-1.156
XLOC_l2_010602	BROAD Institute lincRNA (XLOC_l2_010602), lincRNA [TCONS_l2_00020446]	A_21_P0012578	0.00498	0.0323	-2.58	-1.367
A_21_P0014843	PREDICTED: Homo sapiens hypothetical LOC100507149 (LOC100507149), miscRNA [XR_132713]	A_21_P0014843	0.00511	0.0328	-5.81	-2.539
XLOC_004091	BX117931 Soares_testis_NHT Homo sapiens cDNA clone IMAGp998H224107, mRNA sequence [BX117931]	A_21_P0003773	0.00512	0.0328	-3.5	-1.807
LOC100506167	PREDICTED: Homo sapiens hypothetical LOC100506167, transcript variant 3 (LOC100506167), miscRNA	A_21_P0014259	0.00516	0.033	-2.37	-1.245
LOC619207	Homo sapiens scavenger receptor protein family member (LOC619207), non-coding RNA [NR_002934]	A_33_P3247204	0.00523	0.0332	-4.041	-2.015
LOC100506636	PREDICTED: Homo sapiens hypothetical LOC100506636 (LOC100506636), miscRNA [XR_108328]	A_21_P0014066	0.00524	0.0333	-2.205	-1.141
LOC100506025	Homo sapiens uncharacterized LOC100506025 (LOC100506025), transcript variant 1, non-coding RNA	A_21_P0000848	0.00535	0.0337	-9.572	-3.259

XLOC_I2_011272	BROAD Institute lincRNA (XLOC_I2_011272), lincRNA [TCONS_I2_00021299]	A_21_P0012680	0.00541	0.0339	-3.954	-1.983
KRT19P2	Homo sapiens keratin 19 pseudogene 2 (KRT19P2), non-coding RNA [NR_036685]	A_33_P3846653	0.0055	0.0342	-3.5	-1.807
LOC100131138	Homo sapiens uncharacterized LOC100131138 (LOC100131138), non-coding RNA [NR_036513]	A_21_P0007477	0.00551	0.0342	-3.278	-1.713
XLOC_004804	BROAD Institute lincRNA (XLOC_004804), lincRNA	A_21_P0004383	0.00551	0.0342	-4.07	-2.025
LOC100507265	PREDICTED: Homo sapiens hypothetical LOC100507265 (LOC100507265), miscRNA [XR_108421]	A_21_P0014096	0.0055	0.0342	-2.244	-1.166
LOC100506802	PREDICTED: Homo sapiens hypothetical LOC100506802, transcript variant 2 (LOC100506802), miscRNA	A_21_P0014847	0.00564	0.0347	-2.226	-1.154
XLOC_008981	BROAD Institute lincRNA (XLOC_008981), lincRNA	A_21_P0006799	0.00565	0.0347	-3.531	-1.82
UBE2Q2P2	Homo sapiens ubiquitin-conjugating enzyme E2Q family member 2 pseudogene 2 (UBE2Q2P2), non-coding RNA	A_21_P0011383	0.00571	0.0349	-3.095	-1.63
XLOC_000011	PREDICTED: Homo sapiens hypothetical LOC284600 (LOC284600), miscRNA [XR_108282]	A_21_P0000909	0.00584	0.0354	-5.111	-2.354
XLOC_009713	PREDICTED: Homo sapiens mucin-19-like (LOC100506072), mRNA [XM_003118944]	A_21_P0007830	0.00585	0.0354	-3.278	-1.713
XLOC_005281	BROAD Institute lincRNA (XLOC_005281), lincRNA	A_21_P0004649	0.00592	0.0357	-2.43	-1.281
SNORD98	Homo sapiens small nucleolar RNA, C/D box 98 (SNORD98), small nucleolar RNA [NR_003076]	A_21_P0000392	0.00592	0.0357	-3.22	-1.687
C21orf90	Homo sapiens chromosome 21 open reading frame 90 (C21orf90), transcript variant 1, non-coding RNA	A_24_P339858	0.00596	0.0358	-3.184	-1.671
PHF2P1	Homo sapiens PHD finger protein 2 pseudogene 1 (PHF2P1), non-coding RNA [NR_002801]	A_21_P0011210	0.00595	0.0358	-2.057	-1.041
A_21_P0014928	PREDICTED: Homo sapiens hypothetical LOC100653149 (LOC100653149), miscRNA [XR_133276]	A_21_P0014928	0.00619	0.0367	-2.074	-1.053
XLOC_011655	602133560F1 NIH_MGC_81 Homo sapiens cDNA clone IMAGE:4288575 5', mRNA sequence [BF575575]	A_21_P0008908	0.00647	0.0376	-5.696	-2.51
XLOC_I2_009571	BROAD Institute lincRNA (XLOC_I2_009571), lincRNA [TCONS_I2_00018070]	A_21_P0012299	0.00648	0.0377	-2.698	-1.432
A_19_P00318915	PG12A_MOUSE (Q9EPR2) Group XIIA secretory phospholipase A2 precursor (Phosphatidylcholine 2-acylhydrolase GXII) (GXII sPLA2) , partial (8%)	A_19_P00318915	0.00656	0.0379	-3.296	-1.721
A_33_P3216207	PREDICTED: Homo sapiens hypothetical LOC100506867 (LOC100506867), miscRNA [XR_108612]	A_33_P3216207	0.00669	0.0384	-3.254	-1.702
LOC100506659	PREDICTED: Homo sapiens hypothetical LOC100506659 (LOC100506659), miscRNA [XR_110228]	A_21_P0014534	0.00674	0.0386	-2.087	-1.062

LOC283440	Homo sapiens uncharacterized LOC283440 (LOC283440), non-coding RNA [NR_033958]	A_33_P3274832	0.00683	0.0389	-3.13	-1.646
XLOC_012605	BROAD Institute lincRNA (XLOC_012605), lincRNA	A_21_P0009437	0.00685	0.039	-5.853	-2.549
XLOC_001952	Unknown	A_19_P00316666	0.00693	0.0392	-1.512	-0.596
TSIX	Homo sapiens TSIX transcript, XIST antisense RNA (non-protein coding) (TSIX), antisense RNA [NR_003255]	A_21_P0006564	0.007	0.0395	-3.703	-1.889
XLOC_l2_007834	BROAD Institute lincRNA (XLOC_l2_007834), lincRNA [TCONS_l2_00015633]	A_19_P00321355	0.0071	0.0398	-1.46	-0.546
ZNF542	Homo sapiens zinc finger protein 542 (ZNF542), transcript variant 4, non-coding RNA [NR_003127]	A_24_P570583	0.00731	0.0405	-2.475	-1.307
XLOC_011691	BROAD Institute lincRNA (XLOC_011691), lincRNA	A_21_P0008921	0.00737	0.0407	-2.279	-1.189
C20orf56	Homo sapiens long intergenic non-protein coding RNA 261 (LINC00261), non-coding RNA [NR_001558]	A_32_P23125	0.00749	0.0411	-23.259	-4.54
XLOC_001676	BROAD Institute lincRNA (XLOC_001676), lincRNA	A_21_P0002611	0.00753	0.0412	-3.15	-1.655
XLOC_l2_009883	PREDICTED: Homo sapiens hypothetical LOC100131679 (LOC100131679), miscRNA [XR_108477]	A_21_P0012391	0.00756	0.0414	-2.176	-1.121
FTX	Homo sapiens FTX transcript, XIST regulator (non-protein coding) (FTX), non-coding RNA [NR_028379]	A_21_P0013866	0.00779	0.0422	-2.08	-1.056
A_19_P00321076	Homo sapiens Pvt1 oncogene (non-protein coding) (PVT1), non-coding RNA [NR_003367]	A_19_P00321076	0.00789	0.0425	-4.091	-2.032
XLOC_l2_003886	BROAD Institute lincRNA (XLOC_l2_003886), lincRNA [TCONS_l2_00007050]	A_21_P0011171	0.00792	0.0426	-3.612	-1.853
FLJ46446	Homo sapiens uncharacterized LOC100132741 (LOC100132741), non-coding RNA [NR_034004]	A_33_P3357097	0.00795	0.0427	-2.869	-1.521
XLOC_l2_014835	BROAD Institute lincRNA (XLOC_l2_014835), lincRNA [TCONS_l2_00028817]	A_21_P0013587	0.00807	0.0431	-2.208	-1.143
LOC100507930	PREDICTED: Homo sapiens hypothetical LOC100507930 (LOC100507930), miscRNA [XR_112623]	A_21_P0014719	0.00822	0.0436	-3.953	-1.983
NRADDP	Homo sapiens neurotrophin receptor associated death domain, pseudogene (NRADDP), non-coding RNA	A_33_P3386150	0.0083	0.0439	-2.168	-1.117
XLOC_l2_001462	BROAD Institute lincRNA (XLOC_l2_001462), lincRNA [TCONS_l2_00002879]	A_21_P0010804	0.00835	0.044	-2.006	-1.004
XLOC_013923	BROAD Institute lincRNA (XLOC_013923), lincRNA	A_21_P0010309	0.00838	0.0441	-2.866	-1.519
A_19_P00323022	Homo sapiens uncharacterized LOC100506190 (LOC100506190), non-coding RNA [NR_038955]	A_19_P00323022	0.00846	0.0443	-2.256	-1.174
LOC100507492	PREDICTED: Homo sapiens hypothetical LOC100507492 (LOC100507492), miscRNA [XR_109121]	A_21_P0014281	0.00849	0.0444	-2.262	-1.178

XLOC_000910	AY005129 EP2H {Homo sapiens} (exp=-1; wgp=0; cg=0), partial (88%) [THC2634266]	A_21_P0001541	0.00853	0.0445	-2.697	-1.431
XLOC_005881	RST43154 Athersys RAGE Library Homo sapiens cDNA, mRNA sequence [BG460568]	A_21_P0004792	0.00859	0.0447	-2.924	-1.548
HIVEP3	Homo sapiens human immunodeficiency virus type I enhancer binding protein 3 (HIVEP3), transcript variant 3,	A_21_P0000740	0.00865	0.045	-3.017	-1.593
A_19_P00321075	Homo sapiens Pvt1 oncogene (non-protein coding) (PVT1), non-coding RNA [NR_003367]	A_19_P00321075	0.00874	0.0452	-4.53	-2.18
A_19_P00801324	ALU8_HUMAN (P39195) Alu subfamily SX sequence contamination warning entry, partial (8%) [THC2612343]	A_19_P00801324	0.0088	0.0454	-3.234	-1.693
LOC100507651	Homo sapiens uncharacterized LOC100507651 (LOC100507651), non-coding RNA [NR_038235]	A_21_P0013487	0.0089	0.0457	-3.115	-1.639
A_19_P00318561	PREDICTED: Homo sapiens hypothetical LOC100506516 (LOC100506516), miscRNA [XR_108763]	A_19_P00318561	0.00894	0.0459	-2.613	-1.385
LOC100128371	PREDICTED: Homo sapiens hypothetical LOC100128371 (LOC100128371), miscRNA [XR_109287]	A_33_P3360785	0.00906	0.0463	-2.145	-1.101
XLOC_011367	DB323163 NT2NE2 Homo sapiens cDNA clone NT2NE2011878 3', mRNA sequence [DB323163]	A_21_P0008682	0.00923	0.0469	-3.225	-1.689
A_19_P00317180	Homo sapiens Pvt1 oncogene (non-protein coding) (PVT1), non-coding RNA [NR_003367]	A_19_P00317180	0.00933	0.0472	-4.112	-2.04
LOC339874	Homo sapiens uncharacterized LOC339874 (LOC339874), non-coding RNA [NR_038976]	A_33_P3323979	0.0094	0.0475	-2.234	-1.16
XLOC_l2_009312	BROAD Institute lincRNA (XLOC_l2_009312), lincRNA [TCONS_l2_00017629]	A_21_P0012253	0.00958	0.048	-2.155	-1.107
XLOC_008984	BROAD Institute lincRNA (XLOC_008984), lincRNA	A_21_P0007030	0.0097	0.0484	-4.011	-2.004
XLOC_003482	BROAD Institute lincRNA (XLOC_003482), lincRNA	A_19_P00812050	0.00985	0.0488	-2.784	-1.477
XLOC_l2_013462	BROAD Institute lincRNA (XLOC_l2_013462), lincRNA [TCONS_l2_00025979]	A_21_P0013235	0.00984	0.0488	-3.224	-1.689
XLOC_l2_004986	Q4RDL2_TETNG (Q4RDL2) Chromosome undetermined SCAF16222, whole genome shotgun sequence, partial	A_21_P0011380	0.00989	0.049	-2.68	-1.422
XLOC_002455	BROAD Institute lincRNA (XLOC_002455), lincRNA	A_19_P00810229	0.0102	0.0499	-2.586	-1.371
