# Supplementary material for: CEBPA-regulated lncRNAs, new players in the study of acute myeloid leukemia
Source: J Hematol Oncol. 2014 Sep 25;7:69. doi: 10.1186/s13045-014-0069-1 (PMC4177583; doi:10.1186/s13045-014-0069-1)
Supplement: Additional file 4: Table S2. — CEBPA-regulated mRNAs with significant differential expression (absolute fold change ≥ 2 and adjusted P value ≤ 0.05) identified in K562. (A) Up-regulated mRNAs. (B) Down-regulated mcRNAs. [file 13045_2014_69_MOESM4_ESM.zip › 13045_2014_69_add1/13045_2014_69_add7.pdf]

Supplementary Table S2A. CEBPA-upregulated coding genes						
**Name**	**Description**	**ProbeID**	**p.value**	**Adj.p.value**	**Fold_Change**	**Log2_Ratio**
ARG1	Homo sapiens arginase, liver (ARG1), transcript variant 1, mRNA [NM_001244438]	A_33_P3352382	4.84E-14	2.45E-09	12430.905	13.602
NLRP12	Homo sapiens NLR family, pyrin domain containing 12 (NLRP12), transcript variant 1, mRNA [NM_033297]	A_23_P101434	8.28E-13	1.88E-08	114.707	6.842
DCDC2	Homo sapiens doublecortin domain containing 2 (DCDC2), transcript variant 1, mRNA [NM_016356]	A_33_P3212109	3.77E-12	4.78E-08	31.674	4.985
HP	Homo sapiens haptoglobin (HP), transcript variant 1, mRNA [NM_005143]	A_23_P206760	1.32E-11	1.12E-07	5965.042	12.542
MTTP	Homo sapiens microsomal triglyceride transfer protein (MTTP), mRNA [NM_000253]	A_33_P3215457	2.88E-10	0.00000209	27.036	4.757
ORM1	Homo sapiens orosomucoid 1 (ORM1), mRNA [NM_000607]	A_23_P169494	4.87E-10	0.00000274	1536.829	10.586
EFCAB5	Homo sapiens EF-hand calcium binding domain 5 (EFCAB5), transcript variant 1, mRNA [NM_198529]	A_33_P3341399	5.61E-10	0.00000284	6.947	2.796
CHI3L1	Homo sapiens chitinase 3-like 1 (cartilage glycoprotein-39) (CHI3L1), mRNA [NM_001276]	A_23_P137665	1.83E-09	0.00000784	7921.402	12.952
S100A8	Homo sapiens S100 calcium binding protein A8 (S100A8), mRNA [NM_002964]	A_23_P434809	1.99E-09	0.00000784	40200.359	15.295
AKAP4	Homo sapiens A kinase (PRKA) anchor protein 4 (AKAP4), transcript variant 1, mRNA [NM_003886]	A_23_P11081	2.01E-09	0.00000784	1320.393	10.367
OSTalpha	Homo sapiens organic solute transporter alpha (OSTalpha), mRNA [NM_152672]	A_24_P385732	2.33E-09	0.00000844	107.703	6.751
SRPX	Homo sapiens sushi-repeat containing protein, X-linked (SRPX), transcript variant 1, mRNA [NM_006307]	A_23_P96383	3.68E-09	0.0000117	38.096	5.252
RELB	Homo sapiens v-rel reticuloendotheliosis viral oncogene homolog B (RELB), mRNA [NM_006509]	A_23_P55706	3.6E-09	0.0000117	15.773	3.979
SLC26A3	Homo sapiens solute carrier family 26, member 3 (SLC26A3), mRNA [NM_000111]	A_23_P123228	4.33E-09	0.0000129	1346.123	10.395
MEP1B	Homo sapiens meprin A, beta (MEP1B), mRNA [NM_005925]	A_23_P78353	5.27E-09	0.0000148	34.043	5.089
WHSC2	BC002764 Wolf-Hirschhorn syndrome candidate 2 protein {Homo sapiens} (exp=-1; wgp=0; cg=0), complete [THC2510256]	A_21_P0003711	5.54E-09	0.0000148	25.027	4.645
DNAJC5B	Homo sapiens DnaJ (Hsp40) homolog, subfamily C, member 5 beta (DNAJC5B), mRNA [NM_033105]	A_24_P355145	7.66E-09	0.0000169	169.616	7.406
FGR	Homo sapiens Gardner-Rasheed feline sarcoma viral (v-fgr) oncogene homolog (FGR), transcript variant 2, mRNA [NM_001042747]	A_23_P103932	7.34E-09	0.0000169	1405.376	10.457
RAB19	Homo sapiens RAB19, member RAS oncogene family (RAB19), mRNA [NM_001008749]	A_33_P3391240	6.86E-09	0.0000169	24.878	4.637
ORM2	Homo sapiens orosomucoid 2 (ORM2), mRNA [NM_000608]	A_23_P9485	7.28E-09	0.0000169	5851.592	12.515
A_33_P3258561	Unknown	A_33_P3258561	9.41E-09	0.0000183	467.899	8.87
LOC100133752	Unknown	A_32_P120183	1.15E-08	0.0000208	35.835	5.163
GATA3	Homo sapiens GATA binding protein 3 (GATA3), transcript variant 1, mRNA [NM_001002295]	A_33_P3360341	1.15E-08	0.0000208	14.302	3.838
ITGA8	Homo sapiens integrin, alpha 8 (ITGA8), mRNA [NM_003638]	A_23_P46781	1.23E-08	0.0000215	56.599	5.823
TNFAIP6	Homo sapiens tumor necrosis factor, alpha-induced protein 6 (TNFAIP6), mRNA [NM_007115]	A_23_P165624	1.31E-08	0.0000221	3640.621	11.83
PLCL2	Homo sapiens phospholipase C-like 2 (PLCL2), transcript variant 2, mRNA [NM_015184]	A_24_P281988	1.69E-08	0.0000268	19.024	4.25
CLEC12A	Homo sapiens C-type lectin domain family 12, member A (CLEC12A), transcript variant 1, mRNA [NM_138337]	A_23_P128470	2.27E-08	0.0000292	83.308	6.38
NLRC5	Homo sapiens NLR family, CARD domain containing 5 (NLRC5), mRNA [NM_032206]	A_23_P26583	2.36E-08	0.0000292	26.215	4.712
C19orf59	Homo sapiens chromosome 19 open reading frame 59 (C19orf59), mRNA [NM_174918]	A_23_P330561	2E-08	0.0000292	398.435	8.638
MNDA	Homo sapiens myeloid cell nuclear differentiation antigen (MNDA), mRNA [NM_002432]	A_23_P137935	2.2E-08	0.0000292	2851.916	11.478
SYT17	Homo sapiens synaptotagmin XVII (SYT17), mRNA [NM_016524]	A_23_P163697	2.22E-08	0.0000292	90.959	6.507
SEPT12	Homo sapiens septin 12 (SEPT12), transcript variant 2, mRNA [NM_144605]	A_23_P89101	2.35E-08	0.0000292	20.503	4.358
GRHL1	Homo sapiens grainyhead-like 1 (Drosophila) (GRHL1), mRNA [NM_198182]	A_33_P3230269	2.42E-08	0.0000292	8.09	3.016
QPCT	Homo sapiens glutaminyl-peptide cyclotransferase (QPCT), mRNA [NM_012413]	A_24_P71468	1.98E-08	0.0000292	7.283	2.865
CLEC4D	Homo sapiens C-type lectin domain family 4, member D (CLEC4D), mRNA [NM_080387]	A_33_P3258977	2.79E-08	0.0000325	7.427	2.893
C2orf27A	Homo sapiens chromosome 2 open reading frame 27A (C2orf27A), mRNA [NM_013310]	A_33_P3416881	2.82E-08	0.0000325	9.416	3.235
ENST00000397520	eukaryotic translation initiation factor 4E nuclear import factor 1 [Source:HGNC Symbol;Acc:16687] [ENST00000397520]	A_33_P3367577	2.91E-08	0.0000328	10.621	3.409
ECSM2	Homo sapiens endothelial cell-specific chemotaxis regulator (ECSCR), mRNA [NM_001077693]	A_23_P72651	3.13E-08	0.0000344	144.446	7.174
GPR109B	Homo sapiens hydroxycarboxylic acid receptor 3 (HCAR3), mRNA [NM_006018]	A_23_P64721	3.37E-08	0.0000356	4824.285	12.236
VNN1	Homo sapiens vanin 1 (VNN1), mRNA [NM_004666]	A_33_P3399571	4.18E-08	0.0000424	662.934	9.373

GPR97	Homo sapiens G protein-coupled receptor 97 (GPR97), mRNA [NM_170776]	A_23_P140760	4.28E-08	0.0000425	114.007	6.833
MEI1	Homo sapiens meiosis inhibitor 1 (MEI1), mRNA [NM_152513]	A_23_P211561	4.61E-08	0.0000449	5.039	2.333
SOD2	Homo sapiens superoxide dismutase 2, mitochondrial (SOD2), nuclear gene encoding mitochondrial protein, transcript variant 2, mRNA [NM_001024465]	A_23_P134176	5.27E-08	0.0000477	11.144	3.478
TMEM71	Homo sapiens transmembrane protein 71 (TMEM71), transcript variant 1, mRNA [NM_144649]	A_23_P321354	5.23E-08	0.0000477	440.84	8.784
LMOD3	Homo sapiens leiomodin 3 (fetal) (LMOD3), mRNA [NM_198271]	A_32_P795513	5.51E-08	0.000049	50.01	5.644
GPR84	Homo sapiens G protein-coupled receptor 84 (GPR84), mRNA [NM_020370]	A_23_P25155	6.02E-08	0.0000517	1283.571	10.326
HTRA4	Homo sapiens HtrA serine peptidase 4 (HTRA4), mRNA [NM_153692]	A_23_P44421	6.55E-08	0.0000535	161.212	7.333
QSOX1	Homo sapiens quiescin Q6 sulfhydryl oxidase 1 (QSOX1), transcript variant 2, mRNA [NM_001004128]	A_33_P3225273	6.48E-08	0.0000535	6.907	2.788
LAIR1	Homo sapiens leukocyte-associated immunoglobulin-like receptor 1 (LAIR1), transcript variant a, mRNA [NM_002287]	A_33_P3253394	7.63E-08	0.0000604	710.913	9.474
ST3GAL6	Homo sapiens ST3 beta-galactoside alpha-2,3-sialyltransferase 6 (ST3GAL6), mRNA [NM_006100]	A_23_P250800	8.38E-08	0.0000644	98.386	6.62
C5orf20	Homo sapiens chromosome 5 open reading frame 20 (C5orf20), mRNA [NM_130848]	A_23_P81441	1.09E-07	0.0000812	127.624	6.996
FCAR	Homo sapiens Fc fragment of IgA, receptor for (FCAR), transcript variant 3, mRNA [NM_133271]	A_24_P348265	1.09E-07	0.0000812	25.279	4.66
S100A12	Homo sapiens S100 calcium binding protein A12 (S100A12), mRNA [NM_005621]	A_33_P3385785	1.19E-07	0.0000862	20169.998	14.3
HPR	Homo sapiens haptoglobin-related protein (HPR), mRNA [NM_020995]	A_33_P3289236	1.33E-07	0.0000936	10931.621	13.416
GAN	Homo sapiens gigaxonin (GAN), mRNA [NM_022041]	A_23_P88873	1.36E-07	0.0000944	2.359	1.238
WIF1	Homo sapiens WNT inhibitory factor 1 (WIF1), mRNA [NM_007191]	A_32_P216520	1.44E-07	0.0000968	191.197	7.579
CCR1	Homo sapiens chemokine (C-C motif) receptor 1 (CCR1), mRNA [NM_001295]	A_24_P148717	1.47E-07	0.0000968	31.502	4.977
STX11	Homo sapiens syntaxin 11 (STX11), mRNA [NM_003764]	A_23_P156788	1.46E-07	0.0000968	70.522	6.14
CCDC149	Homo sapiens coiled-coil domain containing 149 (CCDC149), transcript variant 1, mRNA [NM_173463]	A_33_P3225843	1.65E-07	0.000107	98.68	6.625
SKAP1	Homo sapiens src kinase associated phosphoprotein 1 (SKAP1), transcript variant 1, mRNA [NM_003726]	A_23_P100730	1.78E-07	0.000113	40.225	5.33
DACT1	Homo sapiens dapper, antagonist of beta-catenin, homolog 1 (Xenopus laevis) (DACT1), transcript variant 1, mRNA [NM_016651]	A_23_P65518	1.86E-07	0.000114	20.424	4.352
CXCL10	Homo sapiens chemokine (C-X-C motif) ligand 10 (CXCL10), mRNA [NM_001565]	A_33_P3343175	1.82E-07	0.000114	335.693	8.391
DHRS9	Homo sapiens dehydrogenase/reductase (SDR family) member 9 (DHRS9), transcript variant 1, mRNA [NM_005771]	A_23_P56559	1.87E-07	0.000114	400.182	8.645
C9orf50	Homo sapiens chromosome 9 open reading frame 50 (C9orf50), mRNA [NM_199350]	A_23_P406785	1.99E-07	0.000117	68.108	6.09
TRAF1	Homo sapiens TNF receptor-associated factor 1 (TRAF1), transcript variant 1, mRNA [NM_005658]	A_24_P89891	2.01E-07	0.000117	10.109	3.338
ITGB2	Homo sapiens integrin, beta 2 (complement component 3 receptor 3 and 4 subunit) (ITGB2), transcript variant 1, mRNA [NM_000211]	A_23_P329573	2.05E-07	0.000118	38.878	5.281
RNF149	Homo sapiens ring finger protein 149 (RNF149), mRNA [NM_173647]	A_23_P120153	2.33E-07	0.000124	2.134	1.094
ROPN1L	Homo sapiens rhophilin associated tail protein 1-like (ROPN1L), transcript variant 1, mRNA [NM_031916]	A_23_P121885	2.29E-07	0.000124	32.623	5.028
RNASE2	Homo sapiens ribonuclease, RNase A family, 2 (liver, eosinophil-derived neurotoxin) (RNASE2), mRNA [NM_002934]	A_32_P1712	2.37E-07	0.000124	155.149	7.278
C2orf56	Homo sapiens chromosome 2 open reading frame 56 (C2orf56), nuclear gene encoding mitochondrial protein, transcript variant 1, mRNA [NM_144736]	A_23_P28318	2.38E-07	0.000124	2.713	1.44
CXCL1	Homo sapiens chemokine (C-X-C motif) ligand 1 (melanoma growth stimulating activity, alpha) (CXCL1), mRNA [NM_001511]	A_33_P3330264	2.29E-07	0.000124	61.068	5.932
C1orf101	Homo sapiens chromosome 1 open reading frame 101 (C1orf101), transcript variant 2, mRNA [NM_173807]	A_33_P3394243	2.26E-07	0.000124	49.138	5.619
ORMDL1	Homo sapiens ORM1-like 1 (S. cerevisiae) (ORMDL1), transcript variant 1, mRNA [NM_016467]	A_23_P120194	2.49E-07	0.000127	3.183	1.67
SAMD3	Homo sapiens sterile alpha motif domain containing 3 (SAMD3), transcript variant 1, mRNA [NM_001017373]	A_23_P93524	2.58E-07	0.000131	122.426	6.936
AVPI1	Homo sapiens arginine vasopressin-induced 1 (AVPI1), mRNA [NM_021732]	A_23_P1492	2.68E-07	0.000134	7.197	2.847
BTNL3	Homo sapiens butyrophilin-like 3 (BTNL3), mRNA [NM_197975]	A_23_P158297	2.78E-07	0.000135	4.857	2.28
ANXA1	Homo sapiens annexin A1 (ANXA1), mRNA [NM_000700]	A_23_P94501	2.76E-07	0.000135	7.744	2.953

CXCL2	Homo sapiens chemokine (C-X-C motif) ligand 2 (CXCL2), mRNA [NM_002089]	A_24_P257416	2.72E-07	0.000135	12.92	3.692
CDA	Homo sapiens cytidine deaminase (CDA), mRNA [NM_001785]	A_23_P34597	3.29E-07	0.000154	170.349	7.412
MYOT	Homo sapiens myotilin (MYOT), transcript variant 1, mRNA [NM_006790]	A_23_P110764	3.5E-07	0.000161	6.855	2.777
RS1	Homo sapiens cyclin-dependent kinase-like 5 (CDKL5), transcript variant I, mRNA [NM_003159]	A_24_P282343	3.75E-07	0.000167	13.463	3.751
AADACL2	Homo sapiens arylacetamide deacetylase-like 2 (AADACL2), mRNA [NM_207365]	A_23_P91970	3.92E-07	0.000171	122.239	6.934
A_33_P3376781	Unknown	A_33_P3376781	3.99E-07	0.000173	2.025	1.018
FAM160A1	Homo sapiens family with sequence similarity 160, member A1 (FAM160A1), mRNA [NM_001109977]	A_33_P3381318	4.03E-07	0.000173	11.716	3.55
ABCC6	Homo sapiens ATP-binding cassette, sub-family C (CFTR/MRP), member 6 (ABCC6), transcript variant 1, mRNA [NM_001171]	A_23_P100539	4.18E-07	0.000175	56.633	5.824
POLA1	Homo sapiens polymerase (DNA directed), alpha 1, catalytic subunit (POLA1), mRNA [NM_016937]	A_32_P1701	4.25E-07	0.000175	3.954	1.983
SLC36A4	Homo sapiens solute carrier family 36 (proton/amino acid symporter), member 4 (SLC36A4), mRNA [NM_152313]	A_23_P86838	4.18E-07	0.000175	4.241	2.084
PNLDC1	Homo sapiens poly(A)-specific ribonuclease (PARN)-like domain containing 1 (PNLDC1), mRNA [NM_173516]	A_23_P323243	4.24E-07	0.000175	9.453	3.241
CCDC83	Homo sapiens coiled-coil domain containing 83 (CCDC83), mRNA [NM_173556]	A_23_P356667	4.3E-07	0.000176	29.025	4.859
CNIH4	Homo sapiens cornichon homolog 4 (Drosophila) (CNIH4), mRNA [NM_014184]	A_23_P200507	4.68E-07	0.000182	3.387	1.76
SLC6A7	Homo sapiens solute carrier family 6 (neurotransmitter transporter, L-proline), member 7 (SLC6A7), mRNA [NM_014228]	A_33_P3261298	4.63E-07	0.000182	10.994	3.459
TCP1	Homo sapiens t-complex 1 (TCP1), transcript variant 1, mRNA [NM_030752]	A_33_P3349947	4.57E-07	0.000182	4.22	2.077
BDH1	Homo sapiens 3-hydroxybutyrate dehydrogenase, type 1 (BDH1), nuclear gene encoding mitochondrial protein, transcript variant 3, mRNA [NM_203314]	A_33_P3252359	4.61E-07	0.000182	112.081	6.808
ICOS	Homo sapiens inducible T-cell co-stimulator (ICOS), mRNA [NM_012092]	A_23_P371215	5.39E-07	0.000191	75.253	6.234
MYH2	Homo sapiens myosin, heavy chain 2, skeletal muscle, adult (MYH2), transcript variant 1, mRNA [NM_017534]	A_23_P38271	5.59E-07	0.000191	12.366	3.628
ECHDC2	Homo sapiens enoyl CoA hydratase domain containing 2 (ECHDC2), transcript variant 2, mRNA [NM_018281]	A_23_P200203	5.06E-07	0.000191	118.411	6.888
PIWIL2	Homo sapiens piwi-like 2 (Drosophila) (PIWIL2), transcript variant 2, mRNA [NM_018068]	A_23_P253074	5.19E-07	0.000191	21.842	4.449
TXNDC13	Homo sapiens thioredoxin-related transmembrane protein 4 (TMX4), mRNA [NM_021156]	A_24_P250535	5.28E-07	0.000191	4.155	2.055
C20orf132	Homo sapiens chromosome 20 open reading frame 132 (C20orf132), transcript variant 1, mRNA [NM_152503]	A_23_P421811	5.1E-07	0.000191	14.2	3.828
C3AR1	Homo sapiens complement component 3a receptor 1 (C3AR1), mRNA [NM_004054]	A_23_P2431	5.47E-07	0.000191	20.208	4.337
FPR1	Homo sapiens formyl peptide receptor 1 (FPR1), transcript variant 2, mRNA [NM_002029]	A_23_P38795	5.51E-07	0.000191	257.415	8.008
GK	Homo sapiens glycerol kinase (GK), transcript variant 4, mRNA [NM_001205019]	A_33_P3413840	5.3E-07	0.000191	4.817	2.268
THC2620859	HUMHAMRT reverse transcriptase {Homo sapiens} (exp=-1; wgp=0; cg=0), partial (7%) [THC2620859]	A_33_P3396431	5.47E-07	0.000191	10.35	3.372
SERPINE2	Homo sapiens serpin peptidase inhibitor, clade E (nexin, plasminogen activator inhibitor type 1), member 2 (SERPINE2), transcript variant 1, mRNA [NM_006216]	A_23_P50919	5.18E-07	0.000191	10.323	3.368
MAP2K6	Homo sapiens mitogen-activated protein kinase kinase 6 (MAP2K6), mRNA [NM_002758]	A_23_P207445	5.22E-07	0.000191	96.3	6.589
EMR3	Homo sapiens egf-like module containing, mucin-like, hormone receptor-like 3 (EMR3), mRNA [NM_032571]	A_23_P218549	5.57E-07	0.000191	75.036	6.23
PDZRN4	Homo sapiens PDZ domain containing ring finger 4 (PDZRN4), transcript variant 2, mRNA [NM_013377]	A_23_P105651	5.11E-07	0.000191	6.935	2.794
SS18L2	Homo sapiens synovial sarcoma translocation gene on chromosome 18-like 2 (SS18L2), mRNA [NM_016305]	A_23_P166698	5.73E-07	0.000194	2.099	1.07
BHMT	Homo sapiens betaine--homocysteine S-methyltransferase (BHMT), mRNA [NM_001713]	A_24_P257511	5.86E-07	0.000197	19.504	4.286
LOC389300	PREDICTED: Homo sapiens hypothetical protein LOC389300 (LOC389300), mRNA [XM_001714293]	A_21_P0004140	5.92E-07	0.000197	34.168	5.095
CYBB	Homo sapiens cytochrome b-245, beta polypeptide (CYBB), mRNA [NM_000397]	A_24_P365767	6E-07	0.000197	253.409	7.985
SPEF1	Homo sapiens sperm flagellar 1 (SPEF1), mRNA [NM_015417]	A_33_P3314643	6.45E-07	0.000206	11.728	3.552

GPR65	Homo sapiens G protein-coupled receptor 65 (GPR65), mRNA [NM_003608]	A_23_P14564	6.46E-07	0.000206	22.105	4.466
FCRLA	Homo sapiens Fc receptor-like A (FCRLA), transcript variant 2, mRNA [NM_032738]	A_23_P46039	6.78E-07	0.000213	450.448	8.815
TRIB1	Homo sapiens tribbles homolog 1 (Drosophila) (TRIB1), mRNA [NM_025195]	A_24_P252497	6.72E-07	0.000213	109.99	6.781
ENPP1	Homo sapiens ectonucleotide pyrophosphatase/phosphodiesterase 1 (ENPP1), mRNA [NM_006208]	A_23_P156880	6.96E-07	0.000215	65.851	6.041
OVOL1	Homo sapiens ovo-like 1(Drosophila) (OVOL1), mRNA [NM_004561]	A_23_P202810	7.24E-07	0.000215	18.577	4.215
C2orf12	Homo sapiens RNA binding motif, single stranded interacting protein 1 (RBMS1), transcript variant 1, mRNA [NM_016836]	A_23_P431410	7.16E-07	0.000215	27.528	4.783
SERPINB2	Homo sapiens serpin peptidase inhibitor, clade B (ovalbumin), member 2 (SERPINB2), transcript variant 2, mRNA [NM_002575]	A_24_P245379	7.25E-07	0.000215	22.69	4.504
TTC27	Homo sapiens tetratricopeptide repeat domain 27 (TTC27), transcript variant 1, mRNA [NM_017735]	A_23_P131227	7.22E-07	0.000215	3.492	1.804
RABGEF1	Homo sapiens RAB guanine nucleotide exchange factor (GEF) 1 (RABGEF1), mRNA [NM_014504]	A_24_P337746	7.27E-07	0.000215	2.659	1.411
LCA5L	Homo sapiens Leber congenital amaurosis 5-like (LCA5L), mRNA [NM_152505]	A_32_P48466	7.49E-07	0.000217	6.808	2.767
BOLA1	Homo sapiens bolA homolog 1 (E. coli) (BOLA1), mRNA [NM_016074]	A_33_P3354151	7.57E-07	0.000217	11.008	3.461
A_33_P3221059	Unknown	A_33_P3221059	7.5E-07	0.000217	3.003	1.586
IL1RN	Homo sapiens interleukin 1 receptor antagonist (IL1RN), transcript variant 4, mRNA [NM_173843]	A_33_P3246833	7.55E-07	0.000217	765.927	9.581
IDO2	Homo sapiens indoleamine 2,3-dioxygenase 2 (IDO2), mRNA [NM_194294]	A_24_P12690	7.8E-07	0.000221	33.224	5.054
GLIPR1	Homo sapiens GLI pathogenesis-related 1 (GLIPR1), mRNA [NM_006851]	A_33_P3418125	7.76E-07	0.000221	53.483	5.741
PPIL6	Homo sapiens peptidylprolyl isomerase (cyclophilin)-like 6 (PPIL6), transcript variant 1, mRNA [NM_173672]	A_33_P3211634	8.05E-07	0.000227	34.064	5.09
STOM	Homo sapiens stomatin (STOM), transcript variant 2, mRNA [NM_198194]	A_24_P141214	8.36E-07	0.000233	12.116	3.599
S100A9	Homo sapiens S100 calcium binding protein A9 (S100A9), mRNA [NM_002965]	A_23_P23048	8.52E-07	0.000236	9837.954	13.264
C12orf60	Homo sapiens chromosome 12 open reading frame 60 (C12orf60), mRNA [NM_175874]	A_23_P364544	8.63E-07	0.000238	10.207	3.351
IER3	Homo sapiens immediate early response 3 (IER3), mRNA [NM_003897]	A_23_P42257	8.73E-07	0.000238	8.123	3.022
SLC7A7	Homo sapiens solute carrier family 7 (amino acid transporter light chain, y+L system), member 7 (SLC7A7), transcript variant 3, mRNA [NM_001126106]	A_23_P99642	8.79E-07	0.000238	28.96	4.856
CSRP2	Homo sapiens cysteine and glycine-rich protein 2 (CSRP2), mRNA [NM_001321]	A_23_P44724	8.9E-07	0.000239	16.808	4.071
MRPS6	Homo sapiens mitochondrial ribosomal protein S6 (MRPS6), nuclear gene encoding mitochondrial protein, mRNA [NM_032476]	A_23_P102890	9.63E-07	0.00025	2.374	1.248
SAT1	Homo sapiens spermidine/spermine N1-acetyltransferase 1 (SAT1), transcript variant 1, mRNA [NM_002970]	A_23_P137016	9.6E-07	0.00025	9.311	3.219
LOC647131	Unknown	A_32_P88905	9.99E-07	0.000253	9.757	3.287
CES1	Homo sapiens carboxylesterase 1 (CES1), transcript variant 1, mRNA [NM_001025195]	A_33_P3241269	9.78E-07	0.000253	3583.656	11.807
ENTPD1	Homo sapiens ectonucleoside triphosphate diphosphohydrolase 1 (ENTPD1), transcript variant 1, mRNA [NM_001776]	A_33_P3218980	9.91E-07	0.000253	234.237	7.872
SOD1	Homo sapiens superoxide dismutase 1, soluble (SOD1), mRNA [NM_000454]	A_23_P154840	0.000001	0.000253	2.297	1.2
CKAP4	Homo sapiens cytoskeleton-associated protein 4 (CKAP4), mRNA [NM_006825]	A_23_P48056	1.02E-06	0.000257	24.931	4.64
ENST00000311208	keratin 17 pseudogene 1 [Source:HGNC Symbol;Acc:6428] [ENST00000399211]	A_24_P882732	1.04E-06	0.000258	48.897	5.612
AKAP12	Homo sapiens A kinase (PRKA) anchor protein 12 (AKAP12), transcript variant 2, mRNA [NM_144497]	A_23_P111311	1.08E-06	0.000258	13.687	3.775
SLC25A28	Homo sapiens solute carrier family 25, member 28 (SLC25A28), mRNA [NM_031212]	A_23_P75220	1.06E-06	0.000258	2.317	1.212
UBQLN2	Homo sapiens ubiquilin 2 (UBQLN2), mRNA [NM_013444]	A_23_P114164	1.06E-06	0.000258	2.001	1.001
PELO	Homo sapiens pelota homolog (Drosophila) (PELO), mRNA [NM_015946]	A_23_P58763	1.08E-06	0.000258	6.695	2.743
FAM9C	family with sequence similarity 9, member C [Source:HGNC Symbol;Acc:18405] [ENST00000333995]	A_32_P12202	1.08E-06	0.000258	148.078	7.21
C12orf55	chromosome 12 open reading frame 63 [Source:HGNC Symbol;Acc:24777] [ENST00000298953]	A_33_P3263518	1.08E-06	0.000258	12.315	3.622
PLAUR	Homo sapiens plasminogen activator, urokinase receptor (PLAUR), transcript variant 3, mRNA [NM_001005377]	A_23_P16469	1.07E-06	0.000258	12.683	3.665
CNDP1	Homo sapiens carnosine dipeptidase 1 (metallopeptidase M20 family) (CNDP1), mRNA [NM_032649]	A_23_P9869	1.11E-06	0.000264	75.815	6.244
HEXIM2	Homo sapiens hexamethylene bis-acetamide inducible 2 (HEXIM2), mRNA [NM_144608]	A_23_P377214	1.15E-06	0.00027	2.129	1.09

PACRG	Homo sapiens PARK2 co-regulated (PACRG), transcript variant 1, mRNA [NM_152410]	A_23_P170901	1.16E-06	0.000271	8.67	3.116
MOSC1	Homo sapiens MOCO sulphurase C-terminal domain containing 1 (MOSC1), nuclear gene encoding mitochondrial protein, mRNA [NM_022746]	A_33_P3247205	1.18E-06	0.000274	26.285	4.716
IL5RA	Homo sapiens interleukin 5 receptor, alpha (IL5RA), transcript variant 3, mRNA [NM_175725]	A_33_P3328254	1.2E-06	0.000276	70.414	6.138
LOC389634	PREDICTED: Homo sapiens hypothetical LOC729732 (LOC729732), miscRNA [XR_133475]	A_33_P3243500	1.22E-06	0.000279	16.908	4.08
hCG_1993592	Homo sapiens hypothetical LOC644903, mRNA (cDNA clone MGC:10701 IMAGE:3832541), complete cds. [BC004487]	A_33_P3325871	1.25E-06	0.000282	3.232	1.693
SLC16A4	Homo sapiens solute carrier family 16, member 4 (monocarboxylic acid transporter 5) (SLC16A4), transcript variant 1, mRNA [NM_004696]	A_33_P3227920	1.31E-06	0.000294	21.2	4.406
PDLIM5	Homo sapiens PDZ and LIM domain 5 (PDLIM5), transcript variant 5, mRNA [NM_001011516]	A_33_P3336925	1.32E-06	0.000295	7.809	2.965
NAALAD2	Homo sapiens N-acetylated alpha-linked acidic dipeptidase 2 (NAALAD2), mRNA [NM_005467]	A_23_P161583	1.33E-06	0.000296	35.397	5.146
ARMC3	Homo sapiens armadillo repeat containing 3 (ARMC3), mRNA [NM_173081]	A_33_P3220207	1.36E-06	0.0003	7.647	2.935
KRT14	Homo sapiens keratin 14 (KRT14), mRNA [NM_000526]	A_33_P3345534	1.38E-06	0.0003	73.125	6.192
EXDL1	Homo sapiens exonuclease 3'-5' domain containing 1 (EXD1), mRNA [NM_152596]	A_23_P376036	1.38E-06	0.0003	117.659	6.878
LOC389676	Homo sapiens uncharacterized LOC389676 (LOC389676), mRNA [NM_001242668]	A_33_P3295036	1.43E-06	0.000306	33.017	5.045
ALDH6A1	Homo sapiens aldehyde dehydrogenase 6 family, member A1 (ALDH6A1), nuclear gene encoding mitochondrial protein, mRNA [NM_005589]	A_23_P128967	1.42E-06	0.000306	8.994	3.169
AQP9	Homo sapiens aquaporin 9 (AQP9), mRNA [NM_020980]	A_23_P106362	1.43E-06	0.000306	1134.571	10.148
RNASE3	Homo sapiens ribonuclease, RNase A family, 3 (RNASE3), mRNA [NM_002935]	A_23_P163025	1.44E-06	0.000307	53.578	5.744
TREM1	Homo sapiens triggering receptor expressed on myeloid cells 1 (TREM1), transcript variant 1, mRNA [NM_018643]	A_23_P19333	1.48E-06	0.00031	30.022	4.908
FRY	Homo sapiens furry homolog (Drosophila) (FRY), mRNA [NM_023037]	A_23_P105862	1.53E-06	0.000315	53.734	5.748
SLC6A13	Homo sapiens solute carrier family 6 (neurotransmitter transporter, GABA), member 13 (SLC6A13), transcript variant 1, mRNA [NM_016615]	A_23_P64980	1.54E-06	0.000316	17.613	4.139
C12orf26	Homo sapiens chromosome 12 open reading frame 26 (C12orf26), mRNA [NM_032230]	A_23_P345928	1.61E-06	0.000326	7.681	2.941
PRPF3	Homo sapiens PRP3 pre-mRNA processing factor 3 homolog (S. cerevisiae) (PRPF3), mRNA [NM_004698]	A_23_P97573	1.6E-06	0.000326	2.401	1.264
CD109	Homo sapiens CD109 molecule (CD109), transcript variant 1, mRNA [NM_133493]	A_23_P331928	1.62E-06	0.000327	53.151	5.732
LIN54	Homo sapiens lin-54 homolog (C. elegans) (LIN54), transcript variant 1, mRNA [NM_194282]	A_33_P3851513	1.64E-06	0.000328	3.182	1.67
HAL	Homo sapiens histidine ammonia-lyase (HAL), mRNA [NM_002108]	A_23_P61637	1.68E-06	0.000329	49.527	5.63
ELMOD3	Homo sapiens ELMO/CED-12 domain containing 3 (ELMOD3), transcript variant 1, mRNA [NM_032213]	A_23_P154256	1.71E-06	0.000333	3.989	1.996
IPCEF1	Homo sapiens interaction protein for cytohesin exchange factors 1 (IPCEF1), transcript variant 1, mRNA [NM_001130699]	A_33_P3397995	1.9E-06	0.000361	18.893	4.24
CCK	Homo sapiens cholecystokinin (CCK), transcript variant 1, mRNA [NM_000729]	A_23_P425681	2.02E-06	0.000378	1256.714	10.295
IGSF2	Homo sapiens CD101 molecule (CD101), mRNA [NM_004258]	A_24_P131066	2.02E-06	0.000378	42.764	5.418
PTPMT1	Homo sapiens protein tyrosine phosphatase, mitochondrial 1 (PTPMT1), nuclear gene encoding mitochondrial protein, transcript variant 1, mRNA [NM_175732]	A_24_P372562	2.09E-06	0.000389	3.081	1.623
SLMO1	Homo sapiens slowmo homolog 1 (Drosophila) (SLMO1), transcript variant 2, mRNA [NM_006553]	A_23_P38677	2.14E-06	0.000392	2.776	1.473
HTR2A	Homo sapiens 5-hydroxytryptamine (serotonin) receptor 2A (HTR2A), transcript variant 1, mRNA [NM_000621]	A_24_P355967	2.13E-06	0.000392	9.205	3.202
LYRM4	Homo sapiens LYR motif containing 4 (LYRM4), transcript variant 1, mRNA [NM_020408]	A_23_P32328	2.18E-06	0.000394	2.779	1.474
KRT17P3	Unknown	A_24_P887857	2.16E-06	0.000394	54.379	5.765
THC2555232	Q80T73_MOUSE (Q80T73) MKIAA1930 protein (Fragment), partial (3%) [THC2555232]	A_33_P3351609	2.19E-06	0.000394	6.175	2.626
CYB5A	Homo sapiens cytochrome b5 type A (microsomal) (CYB5A), transcript variant 3, mRNA [NM_001190807]	A_33_P3311076	2.19E-06	0.000394	28.017	4.808
IL1RAP	Homo sapiens interleukin 1 receptor accessory protein (IL1RAP), transcript variant 1, mRNA [NM_002182]	A_23_P170857	2.19E-06	0.000394	21.536	4.429
SIKE1	Homo sapiens suppressor of IKBKE 1 (SIKE1), transcript variant 1, mRNA [NM_001102396]	A_33_P3211734	2.27E-06	0.000397	4.743	2.246
BCL2A1	Homo sapiens BCL2-related protein A1 (BCL2A1), transcript variant 1, mRNA [NM_004049]	A_23_P152002	2.25E-06	0.000397	1284.019	10.326

OLAH	Homo sapiens oleoyl-ACP hydrolase (OLAH), transcript variant 2, mRNA [NM_001039702]	A_23_P161458	2.29E-06	0.000397	246.569	7.946
HSD17B2	Homo sapiens hydroxysteroid (17-beta) dehydrogenase 2 (HSD17B2), mRNA [NM_002153]	A_23_P118065	2.28E-06	0.000397	128.881	7.01
RAP2C	Homo sapiens RAP2C, member of RAS oncogene family (RAP2C), mRNA [NM_021183]	A_23_P147826	2.26E-06	0.000397	4.794	2.261
BLNK	Homo sapiens B-cell linker (BLNK), transcript variant 1, mRNA [NM_013314]	A_33_P3363637	2.33E-06	0.000402	50.041	5.645
RPGR	Homo sapiens retinitis pigmentosa GTPase regulator (RPGR), transcript variant A, mRNA [NM_000328]	A_23_P136964	2.34E-06	0.000402	4.037	2.013
C4orf34	Homo sapiens chromosome 4 open reading frame 34 (C4orf34), mRNA [NM_174921]	A_23_P112634	2.37E-06	0.000404	4.579	2.195
B3GALT1	Homo sapiens UDP-Gal:betaGlcNAc beta 1,3-galactosyltransferase, polypeptide 1 (B3GALT1), mRNA [NM_020981]	A_23_P108564	2.46E-06	0.000407	3.108	1.636
RSPH3	Homo sapiens radial spoke 3 homolog (Chlamydomonas) (RSPH3), mRNA [NM_031924]	A_23_P59397	2.43E-06	0.000407	2.739	1.454
LOC100132701	Unknown	A_33_P3336562	2.46E-06	0.000407	12.831	3.682
VNN2	Homo sapiens vanin 2 (VNN2), transcript variant 1, mRNA [NM_004665]	A_23_P122724	2.45E-06	0.000407	33.204	5.053
DNHD2	Homo sapiens dynein, axonemal, heavy chain 12 (DNAH12), transcript variant 1, mRNA [NM_178504]	A_32_P14721	2.47E-06	0.000408	46.045	5.525
VGLL1	Homo sapiens vestigial like 1 (Drosophila) (VGLL1), mRNA [NM_016267]	A_23_P253123	2.5E-06	0.00041	140.919	7.139
PDE6H	Homo sapiens phosphodiesterase 6H, cGMP-specific, cone, gamma (PDE6H), mRNA [NM_006205]	A_23_P64913	2.51E-06	0.00041	363.852	8.507
DYSF	Homo sapiens dysferlin, limb girdle muscular dystrophy 2B (autosomal recessive) (DYSF), transcript variant 8, mRNA [NM_003494]	A_23_P39931	2.54E-06	0.000413	213.946	7.741
ALAS1	Homo sapiens aminolevulinate, delta-, synthase 1 (ALAS1), transcript variant 1, mRNA [NM_000688]	A_24_P191588	2.59E-06	0.000419	6.398	2.678
FLJ31715	Homo sapiens cDNA clone IMAGE:3831619. [BC022164]	A_33_P3777165	2.66E-06	0.000423	24.44	4.611
TFB1M	Homo sapiens transcription factor B1, mitochondrial (TFB1M), nuclear gene encoding mitochondrial protein, mRNA [NM_016020]	A_23_P93499	2.67E-06	0.000423	2.618	1.388
MRAP	Homo sapiens melanocortin 2 receptor accessory protein (MRAP), transcript variant 1, mRNA [NM_178817]	A_24_P126210	2.63E-06	0.000423	42.521	5.41
FCGR3A	Homo sapiens Fc fragment of IgG, low affinity IIIa, receptor (CD16a) (FCGR3A), transcript variant 1, mRNA [NM_000569]	A_23_P200728	2.73E-06	0.000425	158.419	7.308
SERPINB8	Homo sapiens serpin peptidase inhibitor, clade B (ovalbumin), member 8 (SERPINB8), transcript variant 3, mRNA [NM_001031848]	A_24_P147461	2.76E-06	0.000425	4.592	2.199
CNDP2	Homo sapiens CNDP dipeptidase 2 (metallopeptidase M20 family) (CNDP2), transcript variant 1, mRNA [NM_018235]	A_23_P89812	2.71E-06	0.000425	2.044	1.031
LOC729792	Homo sapiens mucin 22 (MUC22), mRNA [NM_001198815]	A_33_P3408232	2.7E-06	0.000425	17.659	4.142
PPP1R3D	Homo sapiens protein phosphatase 1, regulatory subunit 3D (PPP1R3D), mRNA [NM_006242]	A_33_P3423859	2.76E-06	0.000425	3.667	1.875
MPP4	Homo sapiens membrane protein, palmitoylated 4 (MAGUK p55 subfamily member 4) (MPP4), mRNA [NM_033066]	A_23_P131263	2.73E-06	0.000425	33.776	5.078
LOC442590	Homo sapiens speedy homolog E5 (Xenopus laevis) (SPDYE5), mRNA [NM_001099435]	A_32_P177040	2.86E-06	0.000432	8.978	3.166
TM2D2	Homo sapiens TM2 domain containing 2 (TM2D2), transcript variant 2, mRNA [NM_031940]	A_23_P502678	2.84E-06	0.000432	3.867	1.951
KILLIN	Homo sapiens killin, p53-regulated DNA replication inhibitor (KLLN), mRNA [NM_001126049]	A_33_P3267410	2.85E-06	0.000432	29.676	4.891
C2	Homo sapiens complement component 2 (C2), transcript variant 3, mRNA [NM_001178063]	A_33_P3404601	2.9E-06	0.000432	5.208	2.381
HSPA1L	Homo sapiens heat shock 70kDa protein 1-like (HSPA1L), mRNA [NM_005527]	A_23_P70547	2.89E-06	0.000432	5.588	2.482
OLR1	Homo sapiens oxidized low density lipoprotein (lectin-like) receptor 1 (OLR1), transcript variant 1, mRNA [NM_002543]	A_24_P124624	2.83E-06	0.000432	93.665	6.549
NFIL3	Homo sapiens nuclear factor, interleukin 3 regulated (NFIL3), mRNA [NM_005384]	A_23_P32253	2.89E-06	0.000432	12.81	3.679
TGIF1	Homo sapiens TGFB-induced factor homeobox 1 (TGIF1), transcript variant 1, mRNA [NM_170695]	A_21_P0000192	2.93E-06	0.000434	3.273	1.71
RAPH1	Homo sapiens Ras association (RalGDS/AF-6) and pleckstrin homology domains 1 (RAPH1), transcript variant 1, mRNA [NM_213589]	A_24_P924862	2.92E-06	0.000434	13.806	3.787
GPR109A	Homo sapiens hydroxycarboxylic acid receptor 2 (HCAR2), mRNA [NM_177551]	A_23_P329924	2.97E-06	0.000439	146.526	7.195
AKTIP	Homo sapiens AKT interacting protein (AKTIP), transcript variant 1, mRNA [NM_001012398]	A_21_P0012826	3.11E-06	0.000453	6.362	2.669
LOC100294501	Homo sapiens mucin 22 (MUC22), mRNA [NM_001198815]	A_33_P3214914	3.14E-06	0.000455	25.25	4.658
ABHD5	Homo sapiens abhydrolase domain containing 5 (ABHD5), mRNA [NM_016006]	A_23_P250294	3.18E-06	0.000458	4.795	2.262

IL3RA	Homo sapiens interleukin 3 receptor, alpha (low affinity) (IL3RA), mRNA [NM_002183]	A_32_P217750	3.2E-06	0.000459	79.693	6.316
LEKR1	Homo sapiens leucine, glutamate and lysine rich 1 (LEKR1), transcript variant 1, mRNA [NM_001004316]	A_33_P3299565	3.28E-06	0.000467	146.494	7.195
FRMD4B	Homo sapiens FERM domain containing 4B (FRMD4B), mRNA [NM_015123]	A_33_P3446495	3.3E-06	0.000468	16.203	4.018
ZDHHC3	Homo sapiens zinc finger, DHHC-type containing 3 (ZDHHC3), transcript variant 2, mRNA [NM_016598]	A_33_P3327479	3.32E-06	0.000469	2.262	1.177
ZC3H12A	Homo sapiens zinc finger CCCH-type containing 12A (ZC3H12A), mRNA [NM_025079]	A_33_P3214720	3.34E-06	0.00047	6.649	2.733
KIAA0408	Homo sapiens KIAA0408 (KIAA0408), mRNA [NM_014702]	A_23_P215048	3.47E-06	0.000483	7.814	2.966
LOC340970	Unknown	A_33_P3279193	3.49E-06	0.000483	12.712	3.668
THEM5	Homo sapiens thioesterase superfamily member 5 (THEM5), mRNA [NM_182578]	A_33_P3270826	3.49E-06	0.000483	100.339	6.649
CALD1	Homo sapiens caldesmon 1 (CALD1), transcript variant 1, mRNA [NM_033138]	A_24_P921366	3.48E-06	0.000483	36.511	5.19
TMEM170B	Homo sapiens transmembrane protein 170B (TMEM170B), mRNA [NM_001100829]	A_32_P178966	3.57E-06	0.000489	17.519	4.131
TMEM163	Homo sapiens transmembrane protein 163 (TMEM163), mRNA [NM_030923]	A_23_P39550	3.65E-06	0.000497	43.317	5.437
SIK1	Homo sapiens salt-inducible kinase 1 (SIK1), mRNA [NM_173354]	A_23_P132121	3.7E-06	0.0005	12.391	3.631
MAP3K5	Homo sapiens mitogen-activated protein kinase kinase kinase 5 (MAP3K5), mRNA [NM_005923]	A_23_P134125	3.68E-06	0.0005	3.004	1.587
CRISP2	Homo sapiens cysteine-rich secretory protein 2 (CRISP2), transcript variant 1, mRNA [NM_003296]	A_32_P173662	3.75E-06	0.000503	74.872	6.226
LOC151438	Homo sapiens cDNA FLJ31315 fis, clone LIVER1000303. [AK055877]	A_33_P3479449	3.75E-06	0.000503	141.638	7.146
CTNNA2	Homo sapiens catenin (cadherin-associated protein), alpha 2 (CTNNA2), transcript variant 1, mRNA [NM_004389]	A_23_P84736	3.81E-06	0.00051	7.586	2.923
SLC35A1	Homo sapiens solute carrier family 35 (CMP-sialic acid transporter), member A1 (SLC35A1), transcript variant 1, mRNA [NM_006416]	A_23_P58912	3.87E-06	0.000513	3.354	1.746
AKR1C1	Homo sapiens aldo-keto reductase family 1, member C1 (dihydrodiol dehydrogenase 1; 20-alpha (3-alpha)-hydroxysteroid dehydrogenase) (AKR1C1), mRNA [NM_001353]	A_23_P257971	3.91E-06	0.000517	30.454	4.929
TTC18	Homo sapiens tetratricopeptide repeat domain 18 (TTC18), mRNA [NM_145170]	A_23_P326931	3.93E-06	0.000519	2.909	1.54
A_33_P3296348	Unknown	A_33_P3296348	4.03E-06	0.000528	10.36	3.373
BC035135	Homo sapiens cDNA clone IMAGE:5263963. [BC035135]	A_33_P3251547	4.09E-06	0.000533	4.613	2.206
LOC100129878	Unknown	A_33_P3313864	4.09E-06	0.000533	23.875	4.577
CYP3A7	Homo sapiens cytochrome P450, family 3, subfamily A, polypeptide 7 (CYP3A7), mRNA [NM_000765]	A_33_P3318117	4.1E-06	0.000533	114.349	6.837
P2RY2	Homo sapiens purinergic receptor P2Y, G-protein coupled, 2 (P2RY2), transcript variant 1, mRNA [NM_176072]	A_23_P24903	4.13E-06	0.000535	37.327	5.222
SLAMF8	Homo sapiens SLAM family member 8 (SLAMF8), mRNA [NM_020125]	A_23_P200138	4.19E-06	0.000538	613.212	9.26
TRIM36	Homo sapiens tripartite motif containing 36 (TRIM36), transcript variant 1, mRNA [NM_018700]	A_23_P110569	4.18E-06	0.000538	7.364	2.88
A_33_P3286929	Unknown	A_33_P3286929	4.17E-06	0.000538	16.451	4.04
MAST2	Homo sapiens microtubule associated serine/threonine kinase 2 (MAST2), mRNA [NM_015112]	A_23_P126008	4.23E-06	0.000541	4.073	2.026
LOC153546	Homo sapiens cDNA FLJ31377 fis, clone NESOP1000087. [AK055939]	A_33_P3839334	4.25E-06	0.000543	10.845	3.439
UBL4B	Homo sapiens ubiquitin-like 4B (UBL4B), mRNA [NM_203412]	A_32_P112910	4.33E-06	0.000549	32.21	5.009
MYO7B	Homo sapiens myosin VIIB (MYO7B), mRNA [NM_001080527]	A_23_P209799	4.33E-06	0.000549	6.713	2.747
TNNC2	Homo sapiens troponin C type 2 (fast) (TNNC2), mRNA [NM_003279]	A_23_P131825	4.37E-06	0.000552	28.473	4.832
ELOVL3	Homo sapiens ELOVL fatty acid elongase 3 (ELOVL3), mRNA [NM_152310]	A_23_P149858	4.54E-06	0.000565	56.873	5.83
ITGA10	Homo sapiens integrin, alpha 10 (ITGA10), mRNA [NM_003637]	A_33_P3263432	4.52E-06	0.000565	18.192	4.185
RABEPK	Homo sapiens Rab9 effector protein with kelch motifs (RABEPK), transcript variant 1, mRNA [NM_005833]	A_24_P386323	4.53E-06	0.000565	3.045	1.606
VAMP4	Homo sapiens vesicle-associated membrane protein 4 (VAMP4), transcript variant 1, mRNA [NM_003762]	A_23_P61569	4.66E-06	0.000576	2.518	1.332
NRBF2	Homo sapiens nuclear receptor binding factor 2 (NRBF2), mRNA [NM_030759]	A_23_P63816	4.67E-06	0.000576	4.745	2.246
KIAA1715	Homo sapiens KIAA1715 (KIAA1715), mRNA [NM_030650]	A_32_P31771	4.72E-06	0.000581	3.808	1.929
MYOM1	Homo sapiens myomesin 1, 185kDa (MYOM1), transcript variant 1, mRNA [NM_003803]	A_23_P96271	4.75E-06	0.000583	176.11	7.46
KRT17	Homo sapiens keratin 17 (KRT17), mRNA [NM_000422]	A_23_P96158	4.89E-06	0.00059	91.732	6.519
LARP5	Homo sapiens La ribonucleoprotein domain family, member 4B (LARP4B), mRNA [NM_015155]	A_23_P405942	5.01E-06	0.000597	2.164	1.114
ARL9	Homo sapiens ADP-ribosylation factor-like 9 (ARL9), mRNA [NM_206919]	A_33_P3416882	5.05E-06	0.000599	3.19	1.673

NLRC4	Homo sapiens NLR family, CARD domain containing 4 (NLRC4), transcript variant 1, mRNA [NM_021209]	A_23_P119835	5.17E-06	0.000609	153.249	7.26
IFNGR1	Homo sapiens interferon gamma receptor 1 (IFNGR1), mRNA [NM_000416]	A_23_P8281	5.18E-06	0.000609	2.804	1.488
CTBP2	Homo sapiens C-terminal binding protein 2 (CTBP2), transcript variant 2, mRNA [NM_022802]	A_23_P63897	5.22E-06	0.000611	10.568	3.402
NSBP1	Homo sapiens high mobility group nucleosome binding domain 5 (HMGN5), mRNA [NM_030763]	A_23_P136909	5.33E-06	0.00062	5.917	2.565
RBMS1	Homo sapiens RNA binding motif, single stranded interacting protein 1 (RBMS1), transcript variant 1, mRNA [NM_016836]	A_33_P3271490	5.35E-06	0.00062	6.113	2.612
NPHP1	Homo sapiens nephronophthisis 1 (juvenile) (NPHP1), transcript variant 1, mRNA [NM_000272]	A_24_P88801	5.4E-06	0.000625	8.644	3.112
SPATS2L	Homo sapiens spermatogenesis associated, serine-rich 2-like (SPATS2L), transcript variant 2, mRNA [NM_001100422]	A_33_P3400389	5.59E-06	0.000639	3.892	1.961
TAPT1	Homo sapiens transmembrane anterior posterior transformation 1 (TAPT1), mRNA [NM_153365]	A_33_P3306177	5.6E-06	0.000639	3.491	1.804
PRL	Homo sapiens prolactin (PRL), transcript variant 1, mRNA [NM_000948]	A_32_P65616	5.61E-06	0.000639	4.44	2.151
LHX9	Homo sapiens LIM homeobox 9 (LHX9), transcript variant 1, mRNA [NM_020204]	A_33_P3365437	5.66E-06	0.00064	12.137	3.601
PLEKHB2	Homo sapiens pleckstrin homology domain containing, family B (evectins) member 2 (PLEKHB2), transcript variant 3, mRNA [NM_001100623]	A_24_P873414	5.7E-06	0.000642	1.585	0.665
PPARG	Homo sapiens peroxisome proliferator-activated receptor gamma (PPARG), transcript variant 3, mRNA [NM_138711]	A_23_P252062	5.7E-06	0.000642	7.658	2.937
ENST00000380859	aldo-keto reductase family 1, member C1 (dihydrodiol dehydrogenase 1; 20-alpha (3-alpha)-hydroxysteroid dehydrogenase) [Source:HGNC Symbol;Acc:384] [ENST00000380859]	A_33_P3365117	5.74E-06	0.000644	32.094	5.004
SNRPB2	Homo sapiens small nuclear ribonucleoprotein polypeptide B (SNRPB2), transcript variant 1, mRNA [NM_003092]	A_23_P40307	5.78E-06	0.000647	2.067	1.047
A_33_P3326817	Unknown	A_33_P3326817	5.84E-06	0.000652	2.677	1.42
SPON1	Homo sapiens spondin 1, extracellular matrix protein (SPON1), mRNA [NM_006108]	A_32_P133072	5.94E-06	0.00066	25.978	4.699
ACYP2	Homo sapiens acylphosphatase 2, muscle type (ACYP2), mRNA [NM_138448]	A_24_P336848	5.99E-06	0.000663	4.466	2.159
C14orf166	Homo sapiens chromosome 14 open reading frame 166 (C14orf166), mRNA [NM_016039]	A_23_P14482	5.99E-06	0.000663	2.214	1.146
PLEK	Homo sapiens pleckstrin (PLEK), mRNA [NM_002664]	A_23_P209678	6.1E-06	0.000674	61.927	5.953
LRRC9	Homo sapiens cDNA FLJ46156 fis, clone TESTI4001569. [AK128037]	A_33_P3228082	6.2E-06	0.000682	33.103	5.049
C15orf38	Homo sapiens chromosome 15 open reading frame 38 (C15orf38), mRNA [NM_182616]	A_24_P404033	6.29E-06	0.000687	14.492	3.857
MYH13	Homo sapiens myosin, heavy chain 13, skeletal muscle (MYH13), mRNA [NM_003802]	A_23_P89334	6.42E-06	0.000695	5.002	2.323
C3orf32	Homo sapiens chromosome 3 open reading frame 32 (C3orf32), mRNA [NM_015931]	A_23_P144005	6.52E-06	0.000699	3.334	1.737
TMPRSS11D	Homo sapiens transmembrane protease, serine 11D (TMPRSS11D), mRNA [NM_004262]	A_33_P3374678	6.5E-06	0.000699	49.153	5.619
ENST00000380464	perilipin 2 [Source:HGNC Symbol;Acc:248] [ENST00000380464]	A_33_P3258617	6.55E-06	0.0007	5.882	2.556
ADFP	Homo sapiens perilipin 2 (PLIN2), transcript variant 1, mRNA [NM_001122]	A_23_P134953	6.69E-06	0.000712	9.905	3.308
THC2679405	Q9VKG5_DROME (Q9VKG5) CG14930-PA (AT28291p), partial (5%) [THC2679405]	A_33_P3369716	6.99E-06	0.000732	107.618	6.75
LOC100287509	PREDICTED: Homo sapiens cytochrome c oxidase subunit 7B, mitochondrial-like (LOC100287509), mRNA [XM_002342119]	A_21_P0013924	7.07E-06	0.000732	2.131	1.092
NFKBIA	Homo sapiens nuclear factor of kappa light polypeptide gene enhancer in B-cells inhibitor, alpha (NFKBIA), mRNA [NM_020529]	A_23_P106002	7.04E-06	0.000732	3.015	1.592
COMMD1	Homo sapiens copper metabolism (Murr1) domain containing 1 (COMMD1), mRNA [NM_152516]	A_23_P389525	7.25E-06	0.000744	2.682	1.423
MAP2K3	Homo sapiens mitogen-activated protein kinase kinase 3 (MAP2K3), transcript variant B, mRNA [NM_145109]	A_33_P3234571	7.25E-06	0.000744	9.404	3.233
CASK	Homo sapiens calcium/calmodulin-dependent serine protein kinase (MAGUK family) (CASK), transcript variant 1, mRNA [NM_003688]	A_24_P288722	7.21E-06	0.000744	5.24	2.39
SLC22A16	Homo sapiens solute carrier family 22 (organic cation/carnitine transporter), member 16 (SLC22A16), mRNA [NM_033125]	A_23_P42004	7.29E-06	0.000745	39.498	5.304
ACE2	Homo sapiens angiotensin I converting enzyme (peptidyl-dipeptidase A) 2 (ACE2), mRNA [NM_021804]	A_23_P252981	7.34E-06	0.000746	13.598	3.765

AMPD3	Homo sapiens adenosine monophosphate deaminase 3 (AMPD3), transcript variant 3, mRNA [NM_001025390]	A_24_P304154	7.48E-06	0.000748	4.858	2.28
RAB2A	Homo sapiens RAB2A, member RAS oncogene family (RAB2A), transcript variant 1, mRNA [NM_002865]	A_23_P340251	7.56E-06	0.000754	2.238	1.162
KIF5C	Homo sapiens kinesin family member 5C (KIF5C), mRNA [NM_004522]	A_32_P154473	7.64E-06	0.000756	17.324	4.115
KHDC1	Homo sapiens KH homology domain containing 1 (KHDC1), transcript variant 2, mRNA [NM_030568]	A_24_P280762	7.84E-06	0.000773	14.873	3.895
CTAGE5	Homo sapiens CTAGE family, member 5 (CTAGE5), transcript variant 4, mRNA [NM_203356]	A_32_P116840	8.16E-06	0.000795	5.336	2.416
FAR2	Homo sapiens fatty acyl CoA reductase 2 (FAR2), mRNA [NM_018099]	A_23_P150903	8.16E-06	0.000795	24.687	4.626
PRSS35	Homo sapiens protease, serine, 35 (PRSS35), transcript variant 2, mRNA [NM_153362]	A_23_P42397	8.4E-06	0.000811	17.454	4.125
SIRT1	Homo sapiens sirtuin 1 (SIRT1), transcript variant 1, mRNA [NM_012238]	A_23_P98022	8.4E-06	0.000811	4.109	2.039
NEB	Homo sapiens nebulin (NEB), transcript variant 3, mRNA [NM_004543]	A_23_P146783	8.45E-06	0.000814	44.176	5.465
HERC4	Homo sapiens hect domain and RLD 4 (HERC4), transcript variant 1, mRNA [NM_022079]	A_24_P346807	8.57E-06	0.00082	2.359	1.238
NR4A2	Homo sapiens nuclear receptor subfamily 4, group A, member 2 (NR4A2), mRNA [NM_006186]	A_33_P3299066	8.54E-06	0.00082	7.651	2.936
UPB1	Homo sapiens ureidopropionase, beta (UPB1), mRNA [NM_016327]	A_23_P120822	8.67E-06	0.000823	8.967	3.165
HSPBAP1	Homo sapiens HSPB (heat shock 27kDa) associated protein 1 (HSPBAP1), mRNA [NM_024610]	A_23_P250118	8.81E-06	0.00083	3.906	1.966
GPNMB	Homo sapiens glycoprotein (transmembrane) nmb (GPNMB), transcript variant 1, mRNA [NM_001005340]	A_23_P134426	8.84E-06	0.000831	30.784	4.944
TIGD6	Homo sapiens tigger transposable element derived 6 (TIGD6), transcript variant 1, mRNA [NM_030953]	A_24_P337419	9.08E-06	0.000844	2.283	1.191
CTAGE4	Homo sapiens CTAGE family, member 4 (CTAGE4), mRNA [NM_198495]	A_24_P169773	9.03E-06	0.000844	4.335	2.116
FGD3	Homo sapiens FYVE, RhoGEF and PH domain containing 3 (FGD3), transcript variant 2, mRNA [NM_033086]	A_24_P153840	9.06E-06	0.000844	6.83	2.772
SCRG1	Homo sapiens stimulator of chondrogenesis 1 (SCRG1), mRNA [NM_007281]	A_23_P167159	9.22E-06	0.000854	13.359	3.74
BG204828	PREDICTED: Homo sapiens hypothetical protein LOC389300 (LOC389300), mRNA [XM_001714293]	A_33_P3298930	9.28E-06	0.000854	24.769	4.63
PRDM1	Homo sapiens PR domain containing 1, with ZNF domain (PRDM1), transcript variant 1, mRNA [NM_001198]	A_33_P3342081	9.27E-06	0.000854	13.616	3.767
ADAT2	Homo sapiens peroxisomal biogenesis factor 3 (PEX3), mRNA [NM_003630]	A_23_P259328	9.25E-06	0.000854	8.701	3.121
SLC31A1	Homo sapiens solute carrier family 31 (copper transporters), member 1 (SLC31A1), mRNA [NM_001859]	A_24_P928901	9.42E-06	0.00086	2.301	1.202
MXD1	Homo sapiens MAX dimerization protein 1 (MXD1), transcript variant 1, mRNA [NM_002357]	A_24_P379750	9.41E-06	0.00086	2.918	1.545
PIH1D2	Homo sapiens PIH1 domain containing 2 (PIH1D2), transcript variant 1, mRNA [NM_138789]	A_33_P3278058	9.4E-06	0.00086	11.706	3.549
TMEM38A	Homo sapiens transmembrane protein 38A (TMEM38A), mRNA [NM_024074]	A_23_P101392	9.43E-06	0.00086	5.801	2.536
MAK	Homo sapiens male germ cell-associated kinase (MAK), transcript variant 1, mRNA [NM_005906]	A_24_P363278	9.5E-06	0.000864	3.815	1.931
A_33_P3412488	Unknown	A_33_P3412488	9.71E-06	0.000877	5.641	2.496
CAV1	Homo sapiens caveolin 1, caveolae protein, 22kDa (CAV1), transcript variant 1, mRNA [NM_001753]	A_23_P134454	9.88E-06	0.000888	7.925	2.986
VAMP3	Homo sapiens vesicle-associated membrane protein 3 (cellubrevin) (VAMP3), mRNA [NM_004781]	A_24_P370887	0.00001	0.000892	3.345	1.742
IL17C	Homo sapiens interleukin 17C (IL17C), mRNA [NM_013278]	A_33_P3339625	1.03E-05	0.000899	20.763	4.376
TACC2	Homo sapiens transforming, acidic coiled-coil containing protein 2 (TACC2), transcript variant 1, mRNA [NM_206862]	A_23_P127186	1.03E-05	0.000899	7.306	2.869
GRM1	Homo sapiens glutamate receptor, metabotropic 1 (GRM1), transcript variant 1, mRNA [NM_000838]	A_23_P30976	1.03E-05	0.000899	18.494	4.209
TSGA10	Homo sapiens testis specific, 10 (TSGA10), transcript variant 1, mRNA [NM_025244]	A_23_P17103	1.04E-05	0.000901	13.017	3.702
DRP2	Homo sapiens dystrophin related protein 2 (DRP2), transcript variant 1, mRNA [NM_001939]	A_33_P3244753	1.04E-05	0.000901	6.084	2.605
XRCC6BP1	Homo sapiens XRCC6 binding protein 1 (XRCC6BP1), mRNA [NM_033276]	A_23_P53363	1.05E-05	0.000904	2.903	1.538
DNAL1	Homo sapiens dynein, axonemal, light chain 1 (DNAL1), transcript variant 1, mRNA [NM_031427]	A_23_P25913	1.05E-05	0.000904	9.152	3.194
A_19_P00319201	Homo sapiens coiled-coil domain containing 149 (CCDC149), transcript variant 1, mRNA [NM_173463]	A_19_P00319201	1.06E-05	0.000906	22.755	4.508
CLEC4A	Homo sapiens C-type lectin domain family 4, member A (CLEC4A), transcript variant 1, mRNA [NM_016184]	A_23_P48029	1.06E-05	0.000906	7.699	2.945
DNAJC13	Homo sapiens DnaJ (Hsp40) homolog, subfamily C, member 13 (DNAJC13), mRNA [NM_015268]	A_23_P166910	1.08E-05	0.000915	3.935	1.976
YPEL5	Homo sapiens yippee-like 5 (Drosophila) (YPEL5), transcript variant 4, mRNA [NM_016061]	A_23_P108835	1.08E-05	0.000915	3.319	1.731
A_19_P00316511	Homo sapiens coiled-coil domain containing 149 (CCDC149), transcript variant 1, mRNA [NM_173463]	A_19_P00316511	1.08E-05	0.000915	12.622	3.658

C8orf50	Homo sapiens chromosome 8 open reading frame 50, mRNA (cDNA clone IMAGE:5295006). [BC043205]	A_33_P3570974	1.09E-05	0.000919	5.5	2.459
EIF2B2	Homo sapiens eukaryotic translation initiation factor 2B, subunit 2 beta, 39kDa (EIF2B2), mRNA [NM_014239]	A_23_P25929	0.000011	0.000923	2.224	1.153
SYT11	Homo sapiens synaptotagmin XI (SYT11), mRNA [NM_152280]	A_24_P248240	0.000011	0.000923	22.47	4.49
DYNLT3	Homo sapiens dynein, light chain, Tctex-type 3 (DYNLT3), mRNA [NM_006520]	A_24_P91852	1.12E-05	0.000925	8.429	3.075
GLRX2	Homo sapiens glutaredoxin 2 (GLRX2), transcript variant 1, mRNA [NM_016066]	A_23_P160503	1.12E-05	0.000925	6.85	2.776
ANKRD6	Homo sapiens ankyrin repeat domain 6 (ANKRD6), transcript variant 2, mRNA [NM_001242811]	A_33_P3278455	1.11E-05	0.000925	11.692	3.547
CSF3R	Homo sapiens colony stimulating factor 3 receptor (granulocyte) (CSF3R), transcript variant 3, mRNA [NM_156039]	A_33_P3881262	1.12E-05	0.000925	26.728	4.74
ERRFI1	Homo sapiens ERBB receptor feedback inhibitor 1 (ERRFI1), mRNA [NM_018948]	A_23_P46470	1.12E-05	0.000925	8.994	3.169
CPNE6	Homo sapiens copine VI (neuronal) (CPNE6), mRNA [NM_006032]	A_23_P151598	1.11E-05	0.000925	19.223	4.265
ADCY4	Homo sapiens adenylate cyclase 4 (ADCY4), transcript variant 2, mRNA [NM_139247]	A_23_P381261	1.11E-05	0.000925	3.528	1.819
AP1M1	Homo sapiens adaptor-related protein complex 1, mu 1 subunit (AP1M1), transcript variant 2, mRNA [NM_032493]	A_23_P55802	1.14E-05	0.000936	2.615	1.387
BG204039	RST23433 Athersys RAGE Library Homo sapiens cDNA, mRNA sequence [BG204039]	A_33_P3270767	1.14E-05	0.000936	47.348	5.565
ITGA7	Homo sapiens integrin, alpha 7 (ITGA7), transcript variant 2, mRNA [NM_002206]	A_23_P128084	1.15E-05	0.000942	3.654	1.869
LOC100130357	Homo sapiens uncharacterized LOC100130357 (LOC100130357), mRNA [NM_001242698]	A_24_P359067	1.16E-05	0.000944	4.99	2.319
ALPL	Homo sapiens alkaline phosphatase, liver/bone/kidney (ALPL), transcript variant 1, mRNA [NM_000478]	A_24_P353619	1.17E-05	0.000947	31.894	4.995
TSPAN8	Homo sapiens tetraspanin 8 (TSPAN8), mRNA [NM_004616]	A_23_P36531	1.17E-05	0.000947	277.976	8.119
A_19_P00322904	Homo sapiens coiled-coil domain containing 149 (CCDC149), transcript variant 1, mRNA [NM_173463]	A_19_P00322904	1.19E-05	0.00095	75.632	6.241
DENND5A	Homo sapiens DENN/MADD domain containing 5A (DENND5A), transcript variant 1, mRNA [NM_015213]	A_23_P321201	1.19E-05	0.00095	2.84	1.506
ARL6IP5	Homo sapiens ADP-ribosylation-like factor 6 interacting protein 5 (ARL6IP5), mRNA [NM_006407]	A_24_P257348	0.000012	0.000956	2.236	1.161
LOC100129203	PREDICTED: Homo sapiens hypothetical LOC100129203 (LOC100129203), miscRNA [XR_110904]	A_21_P0014651	1.21E-05	0.000961	14.662	3.874
GALP	Homo sapiens galanin-like peptide (GALP), transcript variant 1, mRNA [NM_033106]	A_23_P39171	1.23E-05	0.000973	6.298	2.655
OSTbeta	Homo sapiens organic solute transporter beta (OSTBETA), mRNA [NM_178859]	A_23_P436284	1.26E-05	0.000987	21.561	4.43
COQ2	Homo sapiens coenzyme Q2 homolog, prenyltransferase (yeast) (COQ2), nuclear gene encoding mitochondrial protein, mRNA [NM_015697]	A_23_P124733	1.27E-05	0.000993	2.957	1.564
B4GALT4	Homo sapiens UDP-Gal:betaGlcNAc beta 1,4- galactosyltransferase, polypeptide 4 (B4GALT4), transcript variant 1, mRNA [NM_212543]	A_32_P103945	1.28E-05	0.000997	6.558	2.713
LNP1	Homo sapiens leukemia NUP98 fusion partner 1 (LNP1), mRNA [NM_001085451]	A_33_P3215739	1.28E-05	0.000997	6.471	2.694
GLT8D3	Homo sapiens glucoside xylosyltransferase 1 (GXYLT1), transcript variant 1, mRNA [NM_173601]	A_23_P336796	1.29E-05	0.001	5.544	2.471
AKT3	Homo sapiens v-akt murine thymoma viral oncogene homolog 3 (protein kinase B, gamma) (AKT3), transcript variant 1, mRNA [NM_005465]	A_24_P110983	1.33E-05	0.00102	10.05	3.329
C6orf81	Homo sapiens chromosome 6 open reading frame 81 (C6orf81), mRNA [NM_145028]	A_32_P146113	1.34E-05	0.00102	104.96	6.714
AMICA1	Homo sapiens adhesion molecule, interacts with CXADR antigen 1 (AMICA1), transcript variant 2, mRNA [NM_153206]	A_24_P192914	1.31E-05	0.00102	128.186	7.002
ITGAL	Homo sapiens integrin, alpha L (antigen CD11A (p180), lymphocyte function-associated antigen 1; alpha polypeptide) (ITGAL), transcript variant 1, mRNA [NM_002209]	A_23_P206806	1.37E-05	0.00104	12.107	3.598
LAPTM4A	Homo sapiens lysosomal protein transmembrane 4 alpha (LAPTM4A), mRNA [NM_014713]	A_23_P90659	1.38E-05	0.00105	2.231	1.158
A_19_P00810523	Homo sapiens NADH dehydrogenase (ubiquinone) 1 alpha subcomplex, assembly factor 2 (NDUFAF2), nuclear gene encoding mitochondrial protein, mRNA [NM_174889]	A_19_P00810523	1.39E-05	0.00105	2.503	1.324
LOC647121	Homo sapiens embigin (EMB), mRNA [NM_198449]	A_24_P684186	0.000014	0.00106	23.21	4.537
LOC644727	Homo sapiens cDNA FLJ38652 fis, clone HHDPC2008843. [AK095971]	A_33_P3289251	0.000014	0.00106	6.643	2.732
MCART1	Homo sapiens mitochondrial carrier triple repeat 1 (MCART1), nuclear gene encoding mitochondrial protein, transcript variant 1, mRNA [NM_033412]	A_23_P112512	1.43E-05	0.00107	3.07	1.618
SHROOM3	Homo sapiens shroom family member 3 (SHROOM3), mRNA [NM_020859]	A_33_P3336700	1.43E-05	0.00107	14.212	3.829

SPRY3	Homo sapiens sprouty homolog 3 (Drosophila) (SPRY3), mRNA [NM_005840]	A_23_P311010	1.46E-05	0.00108	8.084	3.015
EIF1B	Homo sapiens eukaryotic translation initiation factor 1B (EIF1B), mRNA [NM_005875]	A_23_P6891	1.46E-05	0.00108	2.031	1.022
FGF2	Homo sapiens fibroblast growth factor 2 (basic) (FGF2), mRNA [NM_002006]	A_33_P3379886	1.44E-05	0.00108	5.93	2.568
RASL11A	Homo sapiens RAS-like, family 11, member A (RASL11A), mRNA [NM_206827]	A_23_P14124	1.45E-05	0.00108	3.583	1.841
EFCAB7	Homo sapiens EF-hand calcium binding domain 7 (EFCAB7), mRNA [NM_032437]	A_32_P137266	1.47E-05	0.00109	9.318	3.22
GPRIN2	Homo sapiens G protein regulated inducer of neurite outgrowth 2 (GPRIN2), mRNA [NM_014696]	A_33_P3285299	1.48E-05	0.00109	10.774	3.429
LOC285771	Homo sapiens cDNA FLJ39868 fis, clone SPLEN2015415. [AK097187]	A_33_P3773261	1.48E-05	0.00109	2.739	1.454
TRIM49	Homo sapiens tripartite motif containing 49 (TRIM49), mRNA [NM_020358]	A_23_P1575	1.49E-05	0.0011	7.9	2.982
CCDC104	Homo sapiens coiled-coil domain containing 104 (CCDC104), mRNA [NM_080667]	A_23_P68327	1.52E-05	0.00111	3.035	1.602
VWA5A	Homo sapiens von Willebrand factor A domain containing 5A (VWA5A), transcript variant 2, mRNA [NM_198315]	A_33_P3305105	1.52E-05	0.00111	5.363	2.423
PRRT4	Homo sapiens proline-rich transmembrane protein 4 (PRRT4), transcript variant 1, mRNA [NM_001174164]	A_33_P3407529	1.53E-05	0.00111	27.097	4.76
PDHA1	Homo sapiens pyruvate dehydrogenase (lipoamide) alpha 1 (PDHA1), nuclear gene encoding mitochondrial protein, transcript variant 1, mRNA [NM_000284]	A_23_P251095	1.54E-05	0.00112	2.298	1.201
NCF2	Homo sapiens neutrophil cytosolic factor 2 (NCF2), transcript variant 1, mRNA [NM_000433]	A_23_P138194	1.57E-05	0.00113	21.24	4.409
SERF1B	Homo sapiens small EDRK-rich factor 1B (centromeric) (SERF1B), transcript variant 2, mRNA [NM_022978]	A_24_P935881	1.59E-05	0.00113	2.011	1.008
A_19_P00804093	Homo sapiens coiled-coil domain containing 149 (CCDC149), transcript variant 1, mRNA [NM_173463]	A_19_P00804093	1.57E-05	0.00113	33.757	5.077
SYNE2	Homo sapiens spectrin repeat containing, nuclear envelope 2 (SYNE2), transcript variant 5, mRNA [NM_182914]	A_33_P3210278	1.59E-05	0.00113	46.012	5.524
C11orf53	Homo sapiens chromosome 11 open reading frame 53 (C11orf53), mRNA [NM_198498]	A_33_P3237260	1.58E-05	0.00113	6.199	2.632
IL6R	Homo sapiens interleukin 6 receptor (IL6R), transcript variant 3, mRNA [NM_001206866]	A_21_P0000171	1.59E-05	0.00113	21.354	4.416
PDE6C	Homo sapiens phosphodiesterase 6C, cGMP-specific, cone, alpha prime (PDE6C), mRNA [NM_006204]	A_23_P98070	1.58E-05	0.00113	6.768	2.759
ANXA2	Homo sapiens annexin A2 (ANXA2), transcript variant 2, mRNA [NM_001002857]	A_23_P146644	1.56E-05	0.00113	2.98	1.575
ABTB2	Homo sapiens ankyrin repeat and BTB (POZ) domain containing 2 (ABTB2), mRNA [NM_145804]	A_23_P356616	1.57E-05	0.00113	4.242	2.085
GANC	Homo sapiens calpain 3, (p94) (CAPN3), transcript variant 1, mRNA [NM_000070]	A_23_P218190	1.59E-05	0.00113	15.861	3.987
OASL	Homo sapiens 2'-5'-oligoadenylate synthetase-like (OASL), transcript variant 1, mRNA [NM_003733]	A_23_P139786	1.64E-05	0.00114	74.651	6.222
ALDH3B1	Homo sapiens aldehyde dehydrogenase 3 family, member B1 (ALDH3B1), transcript variant 1, mRNA [NM_000694]	A_24_P218688	1.62E-05	0.00114	5.179	2.373
C12orf29	Homo sapiens chromosome 12 open reading frame 29 (C12orf29), mRNA [NM_001009894]	A_32_P46765	1.63E-05	0.00114	5.432	2.441
A_33_P3329740	Unknown	A_33_P3329740	1.64E-05	0.00114	3.435	1.78
AX748298	Homo sapiens interleukin 17 receptor A (IL17RA), mRNA [NM_014339]	A_33_P3540143	1.61E-05	0.00114	7.407	2.889
BRI3	Homo sapiens brain protein I3 (BRI3), transcript variant 2, mRNA [NM_001159491]	A_21_P0000022	1.62E-05	0.00114	5.342	2.417
ABCA5	Homo sapiens ATP-binding cassette, sub-family A (ABC1), member 5 (ABCA5), transcript variant 1, mRNA [NM_018672]	A_23_P78018	1.63E-05	0.00114	20.057	4.326
TNFRSF10B	Homo sapiens tumor necrosis factor receptor superfamily, member 10b (TNFRSF10B), transcript variant 1, mRNA [NM_003842]	A_24_P218265	1.65E-05	0.00115	3.259	1.705
GNL2	Homo sapiens guanine nucleotide binding protein-like 2 (nucleolar) (GNL2), mRNA [NM_013285]	A_23_P34578	1.65E-05	0.00115	2.761	1.465
INHBA	Homo sapiens inhibin, beta A (INHBA), mRNA [NM_002192]	A_23_P122924	1.67E-05	0.00116	2.349	1.232
YAP1	Homo sapiens Yes-associated protein 1 (YAP1), transcript variant 2, mRNA [NM_006106]	A_23_P104762	1.69E-05	0.00117	4.025	2.009
CHORDC1	Homo sapiens cysteine and histidine-rich domain (CHORD) containing 1 (CHORDC1), transcript variant 1, mRNA [NM_012124]	A_23_P138805	1.71E-05	0.00117	2.207	1.142
LOC100129778	Unknown	A_33_P3371450	1.69E-05	0.00117	22.529	4.494
CDADC1	Homo sapiens cytidine and dCMP deaminase domain containing 1 (CDADC1), transcript variant 2, mRNA [NM_001193478]	A_33_P3419545	0.000017	0.00117	2.616	1.388
C16orf70	Homo sapiens chromosome 16 open reading frame 70 (C16orf70), mRNA [NM_025187]	A_23_P206382	1.73E-05	0.00118	3.275	1.711

BCL11A	Homo sapiens B-cell CLL/lymphoma 11A (zinc finger protein) (BCL11A), transcript variant 1, mRNA [NM_022893]	A_24_P411186	1.76E-05	0.0012	30.394	4.926
SOAT1	Homo sapiens sterol O-acyltransferase 1 (SOAT1), nuclear gene encoding mitochondrial protein, transcript variant 688113, mRNA [NM_003101]	A_24_P216654	1.77E-05	0.0012	4.725	2.24
ROS1	Homo sapiens c-ros oncogene 1 , receptor tyrosine kinase (ROS1), mRNA [NM_002944]	A_23_P70278	1.78E-05	0.0012	11.054	3.466
BCL2L11	Homo sapiens BCL2-like 11 (apoptosis facilitator) (BCL2L11), transcript variant 9, mRNA [NM_207002]	A_33_P3251932	0.000018	0.00121	2.717	1.442
GOLGA1	Homo sapiens golgin A1 (GOLGA1), mRNA [NM_002077]	A_23_P258978	1.83E-05	0.00122	3.763	1.912
THC2772172	Unknown	A_33_P3257683	1.82E-05	0.00122	4.521	2.177
C10orf129	Homo sapiens chromosome 10 open reading frame 129 (C10orf129), mRNA [NM_207321]	A_33_P3246175	1.82E-05	0.00122	95.499	6.577
TNFRSF9	Homo sapiens tumor necrosis factor receptor superfamily, member 9 (TNFRSF9), mRNA [NM_001561]	A_23_P51936	1.82E-05	0.00122	67.26	6.072
MLKL	Homo sapiens mixed lineage kinase domain-like (MLKL), transcript variant 1, mRNA [NM_152649]	A_23_P61050	1.84E-05	0.00123	11.992	3.584
ANGPTL7	Homo sapiens angiopoietin-like 7 (ANGPTL7), mRNA [NM_021146]	A_23_P114862	1.87E-05	0.00123	6.264	2.647
XPA	Homo sapiens xeroderma pigmentosum, complementation group A (XPA), transcript variant 1, mRNA [NM_000380]	A_23_P60283	1.85E-05	0.00123	2.707	1.437
AOX1	Homo sapiens aldehyde oxidase 1 (AOX1), mRNA [NM_001159]	A_33_P3354414	1.86E-05	0.00123	22.706	4.505
KRT72	Homo sapiens keratin 72 (KRT72), transcript variant 1, mRNA [NM_080747]	A_32_P48825	1.87E-05	0.00123	13.047	3.706
GLRX	Homo sapiens glutaredoxin (thioltransferase) (GLRX), transcript variant 1, mRNA [NM_002064]	A_23_P69908	1.89E-05	0.00124	14.795	3.887
IL4R	Homo sapiens interleukin 4 receptor (IL4R), transcript variant 1, mRNA [NM_000418]	A_23_P129556	1.88E-05	0.00124	15.202	3.926
ZCCHC6	Homo sapiens zinc finger, CCHC domain containing 6 (ZCCHC6), transcript variant 1, mRNA [NM_024617]	A_23_P123727	1.93E-05	0.00126	4.109	2.039
STARD13	StAR-related lipid transfer (START) domain containing 13 [Source:HGNC Symbol;Acc:19164] [ENST00000487412]	A_33_P3382147	1.92E-05	0.00126	20.906	4.386
HPGD	Homo sapiens hydroxyprostaglandin dehydrogenase 15-(NAD) (HPGD), transcript variant 1, mRNA [NM_000860]	A_24_P71904	0.00002	0.00129	95.582	6.579
SORCS2	Homo sapiens sortilin-related VPS10 domain containing receptor 2 (SORCS2), mRNA [NM_020777]	A_23_P121665	2.01E-05	0.0013	7.284	2.865
INDO	Homo sapiens indoleamine 2,3-dioxygenase 1 (IDO1), mRNA [NM_002164]	A_23_P112026	2.02E-05	0.0013	4.929	2.301
CRISPLD2	Homo sapiens cysteine-rich secretory protein LCCL domain containing 2 (CRISPLD2), mRNA [NM_031476]	A_23_P106602	2.04E-05	0.00131	11.563	3.532
AKD1	Homo sapiens cDNA FLJ16163 fis, clone BRCAN2014229. [AK131244]	A_33_P3384078	2.07E-05	0.00131	9.017	3.173
FGF20	Homo sapiens fibroblast growth factor 20 (FGF20), mRNA [NM_019851]	A_33_P3400248	2.07E-05	0.00131	7.865	2.975
SSH2	Homo sapiens slingshot homolog 2 (Drosophila) (SSH2), mRNA [NM_033389]	A_33_P3363168	2.04E-05	0.00131	1.95	0.963
SLC22A14	Homo sapiens solute carrier family 22, member 14 (SLC22A14), mRNA [NM_004803]	A_33_P3287203	2.08E-05	0.00132	2.921	1.547
MEST	Homo sapiens mesoderm specific transcript homolog (mouse) (MEST), transcript variant 1, mRNA [NM_002402]	A_23_P156970	2.12E-05	0.00133	3.765	1.913
SLC31A2	Homo sapiens solute carrier family 31 (copper transporters), member 2 (SLC31A2), mRNA [NM_001860]	A_23_P217109	2.11E-05	0.00133	11.995	3.584
SLPI	Homo sapiens secretory leukocyte peptidase inhibitor (SLPI), mRNA [NM_003064]	A_24_P190472	2.14E-05	0.00134	718.19	9.488
PKD2L1	Homo sapiens polycystic kidney disease 2-like 1 (PKD2L1), mRNA [NM_016112]	A_23_P12554	2.16E-05	0.00134	36.898	5.205
VAMP1	Homo sapiens vesicle-associated membrane protein 1 (synaptobrevin 1) (VAMP1), transcript variant 3, mRNA [NM_016830]	A_23_P105545	2.17E-05	0.00135	5.561	2.475
SESN1	Homo sapiens sestrin 1 (SESN1), transcript variant 1, mRNA [NM_014454]	A_23_P93562	2.23E-05	0.00137	6.197	2.632
OTUD1	Homo sapiens OTU domain containing 1 (OTUD1), mRNA [NM_001145373]	A_32_P60459	2.26E-05	0.00138	4.278	2.097
ID4	Homo sapiens inhibitor of DNA binding 4, dominant negative helix-loop-helix protein (ID4), mRNA [NM_001546]	A_33_P3229370	2.25E-05	0.00138	7.863	2.975
NAMPT	Homo sapiens cDNA FLJ13279 fis, clone OVARC1001055, moderately similar to PRE-B CELL ENHANCING FACTOR PRECURSOR. [AK023341]	A_33_P3364869	2.28E-05	0.00139	14.403	3.848
DEPDC6	Homo sapiens DEP domain containing MTOR-interacting protein (DEPTOR), mRNA [NM_022783]	A_23_P60166	2.33E-05	0.00141	7.839	2.971
TMEM49	Homo sapiens vacuole membrane protein 1 (VMP1), mRNA [NM_030938]	A_33_P3407925	2.32E-05	0.00141	2.44	1.287
TMEM17	Homo sapiens transmembrane protein 17 (TMEM17), mRNA [NM_198276]	A_32_P206949	2.35E-05	0.00141	2.305	1.205

IGSF22	Homo sapiens immunoglobulin superfamily, member 22 (IGSF22), mRNA [NM_173588]	A_32_P486620	2.38E-05	0.00143	8.535	3.093
LOC729083	Homo sapiens, clone IMAGE:4779414, mRNA. [BC038366]	A_33_P3718734	2.42E-05	0.00144	12.604	3.656
KLF5	Homo sapiens Kruppel-like factor 5 (intestinal) (KLF5), mRNA [NM_001730]	A_23_P53891	2.46E-05	0.00146	114.368	6.838
MYPN	Homo sapiens myopalladin (MYPN), mRNA [NM_032578]	A_23_P104438	2.49E-05	0.00147	3.31	1.727
SAP30L	Homo sapiens SAP30-like (SAP30L), transcript variant 1, mRNA [NM_024632]	A_23_P368101	2.51E-05	0.00148	2.33	1.22
ZBTB43	Homo sapiens zinc finger and BTB domain containing 43 (ZBTB43), transcript variant 1, mRNA [NM_014007]	A_33_P3349414	2.53E-05	0.00148	2.702	1.434
LOC100130413	D86966 Start codon is not identified similarto human ZFY protein. {Homo sapiens} (exp=-1; wgp=0; cg=0), partial (3%) [THC2652081]	A_33_P3349496	2.52E-05	0.00148	4.084	2.03
EFHA2	Homo sapiens EF-hand domain family, member A2 (EFHA2), mRNA [NM_181723]	A_24_P194688	2.51E-05	0.00148	8.992	3.169
NAT12	Homo sapiens N(alpha)-acetyltransferase 30, NatC catalytic subunit (NAA30), mRNA [NM_001011713]	A_24_P66679	2.59E-05	0.0015	2.197	1.135
C6orf211	Homo sapiens chromosome 6 open reading frame 211 (C6orf211), mRNA [NM_024573]	A_23_P254472	2.57E-05	0.0015	3.955	1.984
FHOD3	Homo sapiens formin homology 2 domain containing 3 (FHOD3), mRNA [NM_025135]	A_32_P34444	2.58E-05	0.0015	45.854	5.519
CTSK	Homo sapiens cathepsin K (CTSK), mRNA [NM_000396]	A_23_P34744	0.000026	0.0015	5.808	2.538
MAPK14	Homo sapiens mitogen-activated protein kinase 14 (MAPK14), transcript variant 3, mRNA [NM_139013]	A_24_P283288	2.61E-05	0.00151	4.164	2.058
RBM35A	Homo sapiens epithelial splicing regulatory protein 1 (ESRP1), transcript variant 1, mRNA [NM_017697]	A_23_P259127	2.67E-05	0.00153	4.58	2.195
MTRR	Homo sapiens 5-methyltetrahydrofolate-homocysteine methyltransferase reductase (MTRR), transcript variant 1, mRNA [NM_002454]	A_33_P3304377	2.69E-05	0.00153	3.443	1.784
TSPYL1	Homo sapiens TSPY-like 1 (TSPYL1), mRNA [NM_003309]	A_24_P76879	2.74E-05	0.00155	2.063	1.045
CXCL3	Homo sapiens chemokine (C-X-C motif) ligand 3 (CXCL3), mRNA [NM_002090]	A_24_P183150	2.75E-05	0.00155	34.624	5.114
WDR19	Homo sapiens WD repeat domain 19 (WDR19), mRNA [NM_025132]	A_33_P3268618	2.77E-05	0.00156	4.754	2.249
PEX3	Homo sapiens peroxisomal biogenesis factor 3 (PEX3), mRNA [NM_003630]	A_33_P3243588	2.78E-05	0.00156	10.714	3.421
TRIM67	Homo sapiens tripartite motif containing 67 (TRIM67), mRNA [NM_001004342]	A_33_P3235043	2.84E-05	0.00158	96.318	6.59
ITGA1	Homo sapiens integrin, alpha 1 (ITGA1), mRNA [NM_181501]	A_33_P3353791	2.83E-05	0.00158	29.329	4.874
UPK1B	Homo sapiens uroplakin 1B (UPK1B), mRNA [NM_006952]	A_24_P200219	2.87E-05	0.00159	19.639	4.296
KIAA1033	Homo sapiens KIAA1033 (KIAA1033), mRNA [NM_015275]	A_32_P153725	2.88E-05	0.0016	2.344	1.229
DMBT1	Homo sapiens deleted in malignant brain tumors 1 (DMBT1), transcript variant 2, mRNA [NM_007329]	A_23_P86599	0.000029	0.00161	4.593	2.2
DLX2	Homo sapiens distal-less homeobox 2 (DLX2), mRNA [NM_004405]	A_23_P28598	2.91E-05	0.00161	8.679	3.118
THC2521366	Unknown	A_33_P3403708	2.92E-05	0.00161	6.034	2.593
KLHL10	Homo sapiens kelch-like 10 (Drosophila) (KLHL10), mRNA [NM_152467]	A_23_P27128	2.93E-05	0.00162	43.631	5.447
ADH5P4	alcohol dehydrogenase 5 (class III), chi polypeptide, pseudogene 4 [Source:HGNC Symbol;Acc:21377] [ENST00000444694]	A_24_P280873	2.93E-05	0.00162	2.425	1.278
C14orf147	Homo sapiens serine palmitoyltransferase, small subunit A (SPTSSA), mRNA [NM_138288]	A_23_P311150	2.94E-05	0.00162	2.875	1.524
EIF5A2	Homo sapiens eukaryotic translation initiation factor 5A2 (EIF5A2), mRNA [NM_020390]	A_24_P380022	2.97E-05	0.00163	2.041	1.029
TAS1R3	taste receptor, type 1, member 3 [Source:HGNC Symbol;Acc:15661] [ENST00000339381]	A_33_P3359084	2.98E-05	0.00163	3.106	1.635
XPR1	Homo sapiens xenotropic and polytropic retrovirus receptor 1 (XPR1), transcript variant 1, mRNA [NM_004736]	A_24_P336417	2.99E-05	0.00163	3.035	1.602
RHOXF1	Homo sapiens Rhox homeobox family, member 1 (RHOXF1), mRNA [NM_139282]	A_23_P85082	3.01E-05	0.00164	13.298	3.733
LOC100130127	Unknown	A_32_P101844	3.01E-05	0.00164	3.694	1.885
CLLU1OS	Homo sapiens chronic lymphocytic leukemia up-regulated 1 opposite strand (CLLU1OS), mRNA [NM_001025232]	A_33_P3396404	3.01E-05	0.00164	10.045	3.328
CAMP	Homo sapiens cathelicidin antimicrobial peptide (CAMP), mRNA [NM_004345]	A_23_P253791	3.05E-05	0.00165	213.88	7.741
ZFYVE26	Homo sapiens zinc finger, FYVE domain containing 26 (ZFYVE26), mRNA [NM_015346]	A_24_P32118	3.15E-05	0.00168	2.916	1.544
MAT1A	Homo sapiens methionine adenosyltransferase I, alpha (MAT1A), mRNA [NM_000429]	A_23_P23996	3.13E-05	0.00168	25.178	4.654
FRMD3	Homo sapiens FERM domain containing 3 (FRMD3), transcript variant 1, mRNA [NM_174938]	A_33_P3363420	3.16E-05	0.00169	4.607	2.204
KRT24	Homo sapiens keratin 24 (KRT24), mRNA [NM_019016]	A_23_P4387	3.23E-05	0.00172	18.92	4.242

ENST00000377837	hairy and enhancer of split 2 (Drosophila) [Source:HGNC Symbol;Acc:16005] [ENST00000377836]	A_23_P201687	0.000033	0.00173	23.231	4.538
CCDC59	Homo sapiens coiled-coil domain containing 59 (CCDC59), transcript variant 1, mRNA [NM_014167]	A_23_P105664	3.34E-05	0.00174	2.057	1.04
ANG	Homo sapiens angiogenin, ribonuclease, RNase A family, 5 (ANG), transcript variant 1, mRNA [NM_001145]	A_33_P3236177	3.35E-05	0.00174	10.54	3.398
CYP27A1	Homo sapiens cytochrome P450, family 27, subfamily A, polypeptide 1 (CYP27A1), nuclear gene encoding mitochondrial protein, mRNA [NM_000784]	A_33_P3361422	3.35E-05	0.00174	17.615	4.139
RRAGA	Homo sapiens Ras-related GTP binding A (RRAGA), mRNA [NM_006570]	A_23_P169117	3.35E-05	0.00174	2.276	1.186
RRAGC	Homo sapiens Ras-related GTP binding C (RRAGC), mRNA [NM_022157]	A_23_P97623	3.33E-05	0.00174	2.469	1.304
C10orf79	Homo sapiens WD repeat domain 96 (WDR96), mRNA [NM_025145]	A_23_P333038	3.38E-05	0.00175	8.892	3.152
A_19_P00323737	Homo sapiens protein tyrosine phosphatase, non-receptor type 14 (PTPN14), mRNA [NM_005401]	A_19_P00323737	3.39E-05	0.00175	2.882	1.527
PTP4A1	Homo sapiens protein tyrosine phosphatase type IVA, member 1 (PTP4A1), mRNA [NM_003463]	A_23_P81770	3.38E-05	0.00175	2.213	1.146
SLN	Homo sapiens sarcolipin (SLN), mRNA [NM_003063]	A_23_P150343	3.44E-05	0.00176	127.458	6.994
HKDC1	Homo sapiens hexokinase domain containing 1 (HKDC1), mRNA [NM_025130]	A_23_P202427	3.45E-05	0.00176	68.71	6.102
CCDC13	Homo sapiens coiled-coil domain containing 13 (CCDC13), mRNA [NM_144719]	A_33_P3328716	3.41E-05	0.00176	6.556	2.713
FAM65B	Homo sapiens family with sequence similarity 65, member B (FAM65B), transcript variant 1, mRNA [NM_014722]	A_24_P941359	3.47E-05	0.00177	18.058	4.175
SPAG1	Homo sapiens sperm associated antigen 1 (SPAG1), transcript variant 1, mRNA [NM_003114]	A_23_P146066	3.54E-05	0.00179	10.488	3.391
SERAC1	Homo sapiens serine active site containing 1 (SERAC1), mRNA [NM_032861]	A_33_P3777584	3.56E-05	0.00179	10.909	3.447
GOLGA7	Homo sapiens golgin A7 (GOLGA7), transcript variant 2, mRNA [NM_001002296]	A_23_P71440	3.59E-05	0.0018	2.281	1.19
CALCRL	Homo sapiens calcitonin receptor-like (CALCRL), mRNA [NM_005795]	A_33_P3306110	3.57E-05	0.0018	4.376	2.13
APOBEC3A	Homo sapiens apolipoprotein B mRNA editing enzyme, catalytic polypeptide-like 3A (APOBEC3A), transcript variant 1, mRNA [NM_145699]	A_32_P9543	3.61E-05	0.00181	8.281	3.05
ATL1	Homo sapiens atlastin GTPase 1 (ATL1), transcript variant 2, mRNA [NM_181598]	A_23_P88351	3.64E-05	0.00182	10.26	3.359
EPB41L5	Homo sapiens erythrocyte membrane protein band 4.1 like 5 (EPB41L5), transcript variant 3, mRNA [NM_001184938]	A_33_P3248325	0.000037	0.00183	3.846	1.943
SMPDL3A	Homo sapiens sphingomyelin phosphodiesterase, acid-like 3A (SMPDL3A), mRNA [NM_006714]	A_23_P72117	3.69E-05	0.00183	4.568	2.192
MOSC2	Homo sapiens MOCO sulphurase C-terminal domain containing 2 (MOSC2), mRNA [NM_017898]	A_33_P3219434	3.72E-05	0.00184	45.494	5.508
PLAC8	Homo sapiens placenta-specific 8 (PLAC8), transcript variant 2, mRNA [NM_016619]	A_24_P183128	3.73E-05	0.00184	25.121	4.651
TWIST2	Homo sapiens twist homolog 2 (Drosophila), mRNA (cDNA clone MGC:117334 IMAGE:6021554), complete cds. [BC103755]	A_23_P21324	3.74E-05	0.00185	45.656	5.513
CTD-2267G17.3	Homo sapiens X antigen family, member 2B (XAGE2B), mRNA [NM_001079538]	A_23_P34031	3.75E-05	0.00185	1425.238	10.477
CYP19A1	Homo sapiens cytochrome P450, family 19, subfamily A, polypeptide 1 (CYP19A1), transcript variant 2, mRNA [NM_031226]	A_33_P3351371	3.76E-05	0.00185	101.505	6.665
SH3BP5	Homo sapiens SH3-domain binding protein 5 (BTK-associated) (SH3BP5), transcript variant 1, mRNA [NM_004844]	A_24_P148750	3.77E-05	0.00185	12.865	3.685
UBXN2B	Homo sapiens UBX domain protein 2B (UBXN2B), mRNA [NM_001077619]	A_32_P148796	3.82E-05	0.00187	2.58	1.367
ZMYND15	Homo sapiens zinc finger, MYND-type containing 15 (ZMYND15), transcript variant 2, mRNA [NM_032265]	A_23_P89570	0.000038	0.00187	3.029	1.599
A_19_P00315633	Unknown	A_19_P00315633	3.82E-05	0.00187	3.431	1.779
HMHA1	Homo sapiens histocompatibility (minor) HA-1 (HMHA1), mRNA [NM_012292]	A_33_P3318414	3.84E-05	0.00187	6.439	2.687
PSPHP1	Unknown	A_21_P0013198	3.84E-05	0.00187	2.837	1.504
HBB	Homo sapiens hemoglobin, beta (HBB), mRNA [NM_000518]	A_23_P203558	3.88E-05	0.00188	30.586	4.935
CDH4	Homo sapiens cadherin 4, type 1, R-cadherin (retinal) (CDH4), transcript variant 1, mRNA [NM_001794]	A_23_P17593	3.88E-05	0.00188	12.591	3.654
ZAR1L	Homo sapiens zygote arrest 1-like (ZAR1L), mRNA [NM_001136571]	A_33_P3313476	3.92E-05	0.00189	2.648	1.405
SAA4	Homo sapiens serum amyloid A4, constitutive (SAA4), mRNA [NM_006512]	A_23_P87238	3.95E-05	0.0019	13.09	3.71
PRNP	Homo sapiens prion protein (PRNP), transcript variant 1, mRNA [NM_000311]	A_23_P109143	3.94E-05	0.0019	4.304	2.106
LOC730202	Homo sapiens cDNA clone IMAGE:3958634, partial cds. [BC019017]	A_33_P3288074	4.02E-05	0.00191	3.652	1.869
NHSL2	NHS-like 2 [Source:HGNC Symbol;Acc:33737] [ENST00000373677]	A_33_P3260747	3.99E-05	0.00191	5.456	2.448

PCDHA1	Homo sapiens protocadherin alpha 1 (PCDHA1), transcript variant 2, mRNA [NM_031410]	A_33_P3403693	3.99E-05	0.00191	3.729	1.899
VEPH1	Homo sapiens ventricular zone expressed PH domain homolog 1 (zebrafish) (VEPH1), transcript variant 4, mRNA [NM_001167915]	A_21_P0000069	0.00004	0.00191	179.537	7.488
TMSB4X	Homo sapiens thymosin beta 4, X-linked (TMSB4X), mRNA [NM_021109]	A_24_P374516	4.05E-05	0.00192	5.138	2.361
FGD5	Homo sapiens FYVE, RhoGEF and PH domain containing 5 (FGD5), mRNA [NM_152536]	A_33_P3364180	4.03E-05	0.00192	83.405	6.382
NCOA4	Homo sapiens nuclear receptor coactivator 4 (NCOA4), transcript variant 1, mRNA [NM_001145260]	A_33_P3270034	4.08E-05	0.00192	2.496	1.319
FPR2	Homo sapiens formyl peptide receptor 2 (FPR2), transcript variant 1, mRNA [NM_001462]	A_23_P55649	4.04E-05	0.00192	71.505	6.16
CASP10	Homo sapiens caspase 10, apoptosis-related cysteine peptidase (CASP10), transcript variant 1, mRNA [NM_032977]	A_33_P3383283	0.000041	0.00193	7.82	2.967
CCDC135	Homo sapiens coiled-coil domain containing 135 (CCDC135), mRNA [NM_032269]	A_23_P129367	4.17E-05	0.00195	4.031	2.011
ECHDC1	Homo sapiens enoyl CoA hydratase domain containing 1 (ECHDC1), transcript variant 2, mRNA [NM_018479]	A_23_P82206	4.16E-05	0.00195	5.932	2.569
C3	Homo sapiens complement component 3 (C3), mRNA [NM_000064]	A_23_P101407	0.000042	0.00196	11.833	3.565
UTS2	Homo sapiens urotensin 2 (UTS2), transcript variant 1, mRNA [NM_021995]	A_23_P63343	4.19E-05	0.00196	17.951	4.166
C1orf102	Homo sapiens organic solute carrier partner 1 (OSCP1), transcript variant 1, mRNA [NM_145047]	A_23_P103433	4.22E-05	0.00196	4.165	2.058
FBN1	Homo sapiens fibrillin 1 (FBN1), mRNA [NM_000138]	A_33_P3348239	4.19E-05	0.00196	8.745	3.128
MUT	Homo sapiens methylmalonyl CoA mutase (MUT), nuclear gene encoding mitochondrial protein, mRNA [NM_000255]	A_23_P400235	4.21E-05	0.00196	3.477	1.798
KIAA1009	Homo sapiens KIAA1009 (KIAA1009), mRNA [NM_014895]	A_23_P145424	4.27E-05	0.00197	7.016	2.811
HPS4	Homo sapiens Hermansky-Pudlak syndrome 4 (HPS4), transcript variant 1, mRNA [NM_022081]	A_23_P109442	4.27E-05	0.00197	4.855	2.28
SNX24	Homo sapiens sorting nexin 24 (SNX24), mRNA [NM_014035]	A_33_P3702364	4.27E-05	0.00197	3.633	1.861
TIFAB	Homo sapiens TRAF-interacting protein with forkhead-associated domain, family member B (TIFAB), mRNA [NM_001099221]	A_33_P3380383	4.26E-05	0.00197	36.173	5.177
CA2	Homo sapiens carbonic anhydrase II (CA2), mRNA [NM_000067]	A_23_P8913	4.31E-05	0.00199	14.617	3.87
MTUS2	Homo sapiens microtubule associated tumor suppressor candidate 2 (MTUS2), transcript variant 1, mRNA [NM_001033602]	A_33_P3293858	4.37E-05	0.002	18.543	4.213
TLR8	Homo sapiens toll-like receptor 8 (TLR8), mRNA [NM_138636]	A_23_P73837	4.38E-05	0.002	15.36	3.941
PTPLA	Homo sapiens protein tyrosine phosphatase-like (proline instead of catalytic arginine), member A (PTPLA), mRNA [NM_014241]	A_23_P161352	4.36E-05	0.002	6.589	2.72
PION	Homo sapiens pigeon homolog (Drosophila) (PION), mRNA [NM_017439]	A_23_P416894	4.52E-05	0.00204	11.08	3.47
ANKRD58	Homo sapiens ankyrin repeat domain 58 (ANKRD58), mRNA [NM_001105576]	A_32_P123255	4.52E-05	0.00204	29.43	4.879
IGFBP2	Homo sapiens insulin-like growth factor binding protein 2, 36kDa (IGFBP2), mRNA [NM_000597]	A_23_P119943	4.49E-05	0.00204	3.901	1.964
HMCN1	Homo sapiens hemicentin 1 (HMCN1), mRNA [NM_031935]	A_23_P148990	4.57E-05	0.00206	14.54	3.862
TNFSF14	Homo sapiens tumor necrosis factor (ligand) superfamily, member 14 (TNFSF14), transcript variant 1, mRNA [NM_003807]	A_24_P237036	4.63E-05	0.00207	7.927	2.987
MYBPC1	Homo sapiens myosin binding protein C, slow type (MYBPC1), transcript variant 2, mRNA [NM_206819]	A_23_P128362	4.64E-05	0.00207	5.283	2.401
DHRS12	Homo sapiens dehydrogenase/reductase (SDR family) member 12 (DHRS12), transcript variant 1, mRNA [NM_001031719]	A_33_P3342633	4.61E-05	0.00207	4.044	2.016
HEATR5B	Homo sapiens HEAT repeat containing 5B (HEATR5B), mRNA [NM_019024]	A_32_P389118	4.68E-05	0.00208	3.089	1.627
CTSC	Homo sapiens cathepsin C (CTSC), transcript variant 2, mRNA [NM_148170]	A_33_P3283480	4.68E-05	0.00208	2.651	1.407
RP2	Homo sapiens retinitis pigmentosa 2 (X-linked recessive) (RP2), mRNA [NM_006915]	A_23_P22433	4.66E-05	0.00208	3.898	1.963
RABL2A	Homo sapiens RAB, member of RAS oncogene family-like 2A (RABL2A), transcript variant 1, mRNA [NM_013412]	A_24_P250650	0.000047	0.00208	3.414	1.772
GYG1	Homo sapiens glycogenin 1 (GYG1), transcript variant 1, mRNA [NM_004130]	A_23_P384517	4.77E-05	0.00209	13.32	3.736
RP11-94I2.2	Homo sapiens neuroblastoma breakpoint family, member 11 (NBPF11), mRNA [NM_183372]	A_32_P149492	4.72E-05	0.00209	2.827	1.499
RFPL4A	Homo sapiens ret finger protein-like 4A (RFPL4A), mRNA [NM_001145014]	A_33_P3366127	4.75E-05	0.00209	114.026	6.833

MSRB2	Homo sapiens methionine sulfoxide reductase B2 (MSRB2), mRNA [NM_012228]	A_33_P3278318	4.72E-05	0.00209	2.218	1.149
PCGF6	Homo sapiens polycomb group ring finger 6 (PCGF6), transcript variant 1, mRNA [NM_001011663]	A_23_P115703	4.77E-05	0.00209	4.332	2.115
SDCBP2	Homo sapiens syndecan binding protein (syntenin) 2 (SDCBP2), transcript variant 1, mRNA [NM_080489]	A_23_P131899	4.85E-05	0.00211	4.418	2.143
C9orf95	Homo sapiens chromosome 9 open reading frame 95 (C9orf95), transcript variant 1, mRNA [NM_017881]	A_23_P32036	4.82E-05	0.00211	3.638	1.863
AGMAT	Homo sapiens agmatine ureohydrolase (agmatinase) (AGMAT), mRNA [NM_024758]	A_23_P103720	4.84E-05	0.00211	20.059	4.326
SYTL3	Homo sapiens synaptotagmin-like 3 (SYTL3), transcript variant 3, mRNA [NM_001009991]	A_24_P291826	4.83E-05	0.00211	13.23	3.726
TRIM71	Homo sapiens tripartite motif containing 71 (TRIM71), mRNA [NM_001039111]	A_33_P3354429	4.84E-05	0.00211	3.537	1.823
KRT23	Homo sapiens keratin 23 (histone deacetylase inducible) (KRT23), mRNA [NM_015515]	A_23_P78248	4.83E-05	0.00211	79.466	6.312
CLDN12	Homo sapiens claudin 12 (CLDN12), transcript variant 3, mRNA [NM_012129]	A_23_P157268	0.000048	0.00211	4.615	2.206
TBC1D15	Homo sapiens TBC1 domain family, member 15 (TBC1D15), transcript variant 1, mRNA [NM_022771]	A_23_P139558	4.88E-05	0.00212	2.109	1.077
SLC12A2	Homo sapiens solute carrier family 12 (sodium/potassium/chloride transporters), member 2 (SLC12A2), mRNA [NM_001046]	A_32_P25437	4.86E-05	0.00212	6.184	2.629
S100A11	Homo sapiens S100 calcium binding protein A11 (S100A11), mRNA [NM_005620]	A_23_P126593	4.92E-05	0.00213	7.335	2.875
BMX	Homo sapiens BMX non-receptor tyrosine kinase (BMX), transcript variant 2, mRNA [NM_001721]	A_23_P253602	0.000049	0.00213	218.033	7.768
BCL6	Homo sapiens B-cell CLL/lymphoma 6 (BCL6), transcript variant 2, mRNA [NM_001130845]	A_23_P57856	4.98E-05	0.00214	6.839	2.774
ENST00000320216	integrin, beta 2 (complement component 3 receptor 3 and 4 subunit) [Source:HGNC Symbol;Acc:6155] [ENST00000545414]	A_23_P430411	4.95E-05	0.00214	11.401	3.511
LRRC27	Homo sapiens leucine rich repeat containing 27 (LRRC27), transcript variant 4, mRNA [NM_001143759]	A_33_P3304963	4.95E-05	0.00214	2.91	1.541
FNTA	Homo sapiens farnesyltransferase, CAAX box, alpha (FNTA), transcript variant 1, mRNA [NM_002027]	A_23_P24926	4.96E-05	0.00214	2.412	1.27
C9	Homo sapiens complement component 9 (C9), mRNA [NM_001737]	A_32_P203917	0.00005	0.00215	3.495	1.805
SPOCD1	Homo sapiens SPOC domain containing 1 (SPOCD1), mRNA [NM_144569]	A_23_P431388	5.01E-05	0.00215	4.955	2.309
CCDC92	Homo sapiens coiled-coil domain containing 92 (CCDC92), mRNA [NM_025140]	A_23_P98900	5.03E-05	0.00215	5.262	2.396
SLC10A3	Homo sapiens solute carrier family 10 (sodium/bile acid cotransporter family), member 3 (SLC10A3), transcript variant 1, mRNA [NM_019848]	A_33_P3222892	5.04E-05	0.00215	2.777	1.474
TNIK	Homo sapiens TRAF2 and NCK interacting kinase (TNIK), transcript variant 1, mRNA [NM_015028]	A_24_P350576	5.03E-05	0.00215	4.199	2.07
MAP3K8	Homo sapiens mitogen-activated protein kinase kinase kinase 8 (MAP3K8), transcript variant 1, mRNA [NM_005204]	A_23_P23947	5.01E-05	0.00215	6.79	2.763
LOC390595	Homo sapiens ubiquitin associated protein 1-like (UBAP1L), mRNA [NM_001163692]	A_24_P481783	5.08E-05	0.00216	2.669	1.416
TMEM88	Homo sapiens transmembrane protein 88 (TMEM88), mRNA [NM_203411]	A_23_P77859	5.08E-05	0.00216	2.092	1.065
SEMA3C	Homo sapiens sema domain, immunoglobulin domain (Ig), short basic domain, secreted, (semaphorin) 3C (SEMA3C), mRNA [NM_006379]	A_23_P256473	5.15E-05	0.00218	103.85	6.698
MGC11082	Homo sapiens cDNA clone IMAGE:30412101. [BC094703]	A_33_P3774867	5.14E-05	0.00218	6.584	2.719
C10orf118	Homo sapiens chromosome 10 open reading frame 118 (C10orf118), mRNA [NM_018017]	A_24_P942694	5.19E-05	0.00219	5.138	2.361
INTS12	Homo sapiens integrator complex subunit 12 (INTS12), transcript variant 1, mRNA [NM_020395]	A_23_P81087	5.18E-05	0.00219	2.002	1.002
THC2552587	Q96I88_HUMAN (Q96I88) THADA protein (Fragment), partial (8%) [THC2552587]	A_33_P3421611	5.18E-05	0.00219	8.07	3.013
MUC22	Homo sapiens mucin 22 (MUC22), mRNA [NM_001198815]	A_21_P0004641	5.24E-05	0.0022	8.267	3.047
PTDSS1	Homo sapiens phosphatidylserine synthase 1 (PTDSS1), mRNA [NM_014754]	A_23_P168868	5.35E-05	0.00224	2.495	1.319
TCN1	Homo sapiens transcobalamin I (vitamin B12 binding protein, R binder family) (TCN1), mRNA [NM_001062]	A_23_P64372	5.41E-05	0.00225	133.732	7.063
LYG1	Homo sapiens lysozyme G-like 1 (LYG1), mRNA [NM_174898]	A_23_P165707	5.54E-05	0.00229	3.28	1.713
CXorf21	Homo sapiens chromosome X open reading frame 21 (CXorf21), mRNA [NM_025159]	A_23_P62227	5.55E-05	0.00229	9.175	3.198
CDO1	Homo sapiens cysteine dioxygenase, type I (CDO1), mRNA [NM_001801]	A_23_P30294	5.61E-05	0.00231	5.29	2.403
VSTM1	Homo sapiens V-set and transmembrane domain containing 1 (VSTM1), mRNA [NM_198481]	A_33_P3514487	0.000056	0.00231	14.181	3.826
OPN3	Homo sapiens opsin 3 (OPN3), mRNA [NM_014322]	A_33_P3293474	5.59E-05	0.00231	7.306	2.869
C6orf150	Homo sapiens Mab-21 domain containing 1 (MB21D1), mRNA [NM_138441]	A_32_P1173	5.69E-05	0.00233	6.969	2.801
KIAA0319L	KIAA0319-like [Source:HGNC Symbol;Acc:30071] [ENST00000485551]	A_24_P314597	5.71E-05	0.00233	3.88	1.956
UGP2	Homo sapiens UDP-glucose pyrophosphorylase 2 (UGP2), transcript variant 1, mRNA [NM_006759]	A_23_P253046	5.68E-05	0.00233	3.781	1.919

IFNGR2	Homo sapiens interferon gamma receptor 2 (interferon gamma transducer 1) (IFNGR2), mRNA [NM_005534]	A_23_P29036	5.69E-05	0.00233	2.034	1.024
CHAC2	Homo sapiens ChaC, cation transport regulator homolog 2 (E. coli) (CHAC2), mRNA [NM_001008708]	A_32_P194264	5.78E-05	0.00235	4.066	2.024
CAST	Homo sapiens calpastatin (CAST), transcript variant 6, mRNA [NM_001042440]	A_23_P213518	5.86E-05	0.00237	2.102	1.072
DNAH11	Homo sapiens dynein, axonemal, heavy chain 11 (DNAH11), mRNA [NM_003777]	A_32_P19806	5.84E-05	0.00237	4.66	2.22
CRLS1	Homo sapiens cardiolipin synthase 1 (CRLS1), transcript variant 1, mRNA [NM_019095]	A_33_P3502315	5.84E-05	0.00237	2.014	1.01
DACT2	Homo sapiens dapper, antagonist of beta-catenin, homolog 2 (Xenopus laevis) (DACT2), mRNA [NM_214462]	A_24_P289260	5.86E-05	0.00237	15.454	3.95
DKFZp313P036	Homo sapiens u3 small nucleolar ribonucleoprotein protein MPP10-like (LOC643802), mRNA [NM_001207030]	A_24_P221968	0.000059	0.00238	44.064	5.462
DA571569	DA571569 HEMBA1 Homo sapiens cDNA clone HEMBA1006859 5', mRNA sequence [DA571569]	A_33_P3363085	0.000059	0.00238	8.281	3.05
UBN1	Homo sapiens ubinuclein 1 (UBN1), transcript variant 1, mRNA [NM_016936]	A_23_P54900	0.000059	0.00238	2.054	1.038
NKIRAS1	Homo sapiens NFKB inhibitor interacting Ras-like 1 (NKIRAS1), mRNA [NM_020345]	A_23_P338519	5.94E-05	0.00239	4.572	2.193
OAS1	Homo sapiens 2'-5'-oligoadenylate synthetase 1, 40/46kDa (OAS1), transcript variant 2, mRNA [NM_002534]	A_23_P64828	5.99E-05	0.0024	43.349	5.438
ATXN1	Homo sapiens ataxin 1 (ATXN1), transcript variant 1, mRNA [NM_000332]	A_24_P294842	0.000061	0.00243	7.419	2.891
SRPK1	Homo sapiens SRSF protein kinase 1 (SRPK1), transcript variant 1, mRNA [NM_003137]	A_23_P19543	6.09E-05	0.00243	2.606	1.382
VPS8	Homo sapiens vacuolar protein sorting 8 homolog (S. cerevisiae) (VPS8), transcript variant 1, mRNA [NM_001009921]	A_24_P357709	6.11E-05	0.00244	5.179	2.373
NBPF14	Homo sapiens neuroblastoma breakpoint family, member 14 (NBPF14), mRNA [NM_015383]	A_23_P359430	6.18E-05	0.00246	2.104	1.073
PADI4	Homo sapiens peptidyl arginine deiminase, type IV (PADI4), mRNA [NM_012387]	A_23_P138262	6.24E-05	0.00247	7.322	2.872
AKAP5	Homo sapiens A kinase (PRKA) anchor protein 5 (AKAP5), mRNA [NM_004857]	A_23_P106103	6.23E-05	0.00247	19.204	4.263
C6orf103	Homo sapiens chromosome 6 open reading frame 103 (C6orf103), mRNA [NM_024694]	A_23_P70643	6.31E-05	0.00248	6.605	2.724
DLEC1	Homo sapiens deleted in lung and esophageal cancer 1 (DLEC1), transcript variant DLEC1-N1, mRNA [NM_007335]	A_23_P18282	6.33E-05	0.00249	29.519	4.884
LCOR	Homo sapiens ligand dependent nuclear receptor corepressor (LCOR), transcript variant 1, mRNA [NM_032440]	A_21_P0000079	6.33E-05	0.00249	2.698	1.432
ISOC1	Homo sapiens isochorismatase domain containing 1 (ISOC1), mRNA [NM_016048]	A_23_P250982	6.43E-05	0.00251	2.086	1.061
PCMTD2	HSU37230 ribosomal protein L23a {Homo sapiens} (exp=-1; wgp=0; cg=0), partial (46%) [THC2591738]	A_21_P0012528	6.43E-05	0.00251	2.715	1.441
IFI16	Homo sapiens interferon, gamma-inducible protein 16 (IFI16), transcript variant 2, mRNA [NM_005531]	A_23_P160025	6.44E-05	0.00251	15.422	3.947
TMSL3	Homo sapiens thymosin beta 4, X-linked (TMSB4X), mRNA [NM_021109]	A_24_P143189	6.51E-05	0.00252	4.351	2.121
GYS2	Homo sapiens glycogen synthase 2 (liver) (GYS2), mRNA [NM_021957]	A_33_P3360311	6.48E-05	0.00252	7.54	2.915
HABP4	Homo sapiens hyaluronan binding protein 4 (HABP4), mRNA [NM_014282]	A_33_P3592015	6.52E-05	0.00252	2.406	1.267
RFTN1	Homo sapiens raftlin, lipid raft linker 1 (RFTN1), mRNA [NM_015150]	A_23_P315571	6.52E-05	0.00252	36.834	5.203
MED8	Homo sapiens mediator complex subunit 8 (MED8), transcript variant 3, mRNA [NM_052877]	A_23_P46170	6.61E-05	0.00255	4.528	2.179
ARMC9	Homo sapiens armadillo repeat containing 9 (ARMC9), mRNA [NM_025139]	A_23_P209731	6.61E-05	0.00255	4.569	2.192
C1orf161	Homo sapiens mab-21-like 3 (C. elegans) (MAB21L3), mRNA [NM_152367]	A_23_P343366	6.69E-05	0.00258	3.089	1.627
DCP1B	Homo sapiens DCP1 decapping enzyme homolog B (S. cerevisiae) (DCP1B), mRNA [NM_152640]	A_23_P313512	6.74E-05	0.00258	2.302	1.203
TSN	Homo sapiens translin (TSN), mRNA [NM_004622]	A_23_P28105	6.74E-05	0.00258	2.05	1.036
SLC26A2	Homo sapiens solute carrier family 26 (sulfate transporter), member 2 (SLC26A2), mRNA [NM_000112]	A_33_P3277447	0.000067	0.00258	2.881	1.527
CTSS	Homo sapiens cathepsin S (CTSS), transcript variant 1, mRNA [NM_004079]	A_24_P242646	6.81E-05	0.0026	23.761	4.571
HVCN1	Homo sapiens hydrogen voltage-gated channel 1 (HVCN1), transcript variant 1, mRNA [NM_001040107]	A_33_P3368014	6.84E-05	0.00261	9.011	3.172
IL17B	Homo sapiens interleukin 17B (IL17B), mRNA [NM_014443]	A_23_P167479	6.91E-05	0.00263	16.969	4.085
TOX4	Homo sapiens TOX high mobility group box family member 4 (TOX4), mRNA [NM_014828]	A_23_P404134	6.95E-05	0.00264	2.482	1.312
FAM162B	Homo sapiens family with sequence similarity 162, member B (FAM162B), mRNA [NM_001085480]	A_23_P145054	7.05E-05	0.00266	15.559	3.96
BIN3	Homo sapiens bridging integrator 3 (BIN3), mRNA [NM_018688]	A_23_P43086	0.00007	0.00266	2.276	1.186

TMEM169	Homo sapiens transmembrane protein 169 (TMEM169), transcript variant 3, mRNA [NM_138390]	A_24_P929388	7.03E-05	0.00266	5.438	2.443
ITGAV	Homo sapiens integrin, alpha V (vitronectin receptor, alpha polypeptide, antigen CD51) (ITGAV), transcript variant 1, mRNA [NM_002210]	A_23_P50907	7.06E-05	0.00266	3.758	1.91
TSPAN7	Homo sapiens tetraspanin 7 (TSPAN7), mRNA [NM_004615]	A_23_P114185	0.000071	0.00267	2.005	1.004
PDE6A	Homo sapiens phosphodiesterase 6A, cGMP-specific, rod, alpha (PDE6A), mRNA [NM_000440]	A_23_P81590	7.11E-05	0.00267	7.197	2.847
FCGR1B	Homo sapiens Fc fragment of IgG, high affinity Ib, receptor (CD64) (FCGR1B), transcript variant 3, mRNA [NM_001244910]	A_21_P0010561	7.12E-05	0.00268	41.12	5.362
NSUN3	Homo sapiens NOP2/Sun domain family, member 3 (NSUN3), mRNA [NM_022072]	A_23_P21785	7.25E-05	0.00271	2.873	1.522
TDRD7	Homo sapiens tudor domain containing 7 (TDRD7), mRNA [NM_014290]	A_23_P123672	7.24E-05	0.00271	3.145	1.653
RGAG1	Homo sapiens retrotransposon gag domain containing 1 (RGAG1), mRNA [NM_020769]	A_23_P306352	7.32E-05	0.00272	12.484	3.642
SASH3	Homo sapiens SAM and SH3 domain containing 3 (SASH3), mRNA [NM_018990]	A_24_P237443	7.34E-05	0.00272	17.326	4.115
SDC4	Homo sapiens syndecan 4 (SDC4), mRNA [NM_002999]	A_23_P109034	7.32E-05	0.00272	4.435	2.149
TLR7	Homo sapiens toll-like receptor 7 (TLR7), mRNA [NM_016562]	A_23_P85240	7.31E-05	0.00272	14.084	3.816
GPR171	Homo sapiens G protein-coupled receptor 171 (GPR171), mRNA [NM_013308]	A_23_P253317	7.35E-05	0.00272	5.695	2.51
ACOXL	Homo sapiens acyl-CoA oxidase-like (ACOXL), mRNA [NM_001142807]	A_33_P3395008	7.38E-05	0.00273	6.899	2.786
PECAM1	Homo sapiens platelet/endothelial cell adhesion molecule (PECAM1), mRNA [NM_000442]	A_23_P252471	7.42E-05	0.00274	2.408	1.268
UGT2B11	Homo sapiens UDP glucuronosyltransferase 2 family, polypeptide B11 (UGT2B11), mRNA [NM_001073]	A_23_P212968	7.53E-05	0.00276	10.242	3.356
UAP1	Homo sapiens UDP-N-acteylglucosamine pyrophosphorylase 1 (UAP1), mRNA [NM_003115]	A_23_P160460	7.49E-05	0.00276	5.628	2.493
CYP3A5	Homo sapiens cytochrome P450, family 3, subfamily A, polypeptide 5 (CYP3A5), transcript variant 1, mRNA [NM_000777]	A_23_P8801	7.51E-05	0.00276	12.839	3.682
TLR6	Homo sapiens toll-like receptor 6 (TLR6), mRNA [NM_006068]	A_23_P256561	7.53E-05	0.00276	2.378	1.249
SRFBP1	Homo sapiens serum response factor binding protein 1 (SRFBP1), mRNA [NM_152546]	A_23_P435002	7.61E-05	0.00278	3.741	1.904
FILIP1	Homo sapiens filamin A interacting protein 1 (FILIP1), mRNA [NM_015687]	A_23_P436369	7.61E-05	0.00278	6.21	2.635
GAL3ST1	Homo sapiens galactose-3-O-sulfotransferase 1 (GAL3ST1), mRNA [NM_004861]	A_23_P120863	7.62E-05	0.00278	7.256	2.859
ARSB	Homo sapiens arylsulfatase B (ARSB), transcript variant 2, mRNA [NM_198709]	A_24_P205213	7.64E-05	0.00278	2.881	1.527
TIMM23	Homo sapiens translocase of inner mitochondrial membrane 23 homolog (yeast) (TIMM23), nuclear gene encoding mitochondrial protein, mRNA [NM_006327]	A_33_P3403361	7.67E-05	0.00278	4.075	2.027
THC2505774	Unknown	A_33_P3414302	7.68E-05	0.00278	4.211	2.074
RETN	Homo sapiens resistin (RETN), transcript variant 1, mRNA [NM_020415]	A_33_P3350863	7.73E-05	0.00279	24.461	4.612
XX-FW88277B6.1	Homo sapiens cancer/testis antigen family 45, member A1 (CT45A1), mRNA [NM_001017417]	A_32_P780817	7.79E-05	0.0028	15.238	3.93
CRIP3	Homo sapiens cysteine-rich protein 3 (CRIP3), mRNA [NM_206922]	A_33_P3325404	7.78E-05	0.0028	13.795	3.786
CCDC125	Homo sapiens coiled-coil domain containing 125 (CCDC125), mRNA [NM_176816]	A_33_P3271599	7.76E-05	0.0028	4.778	2.256
CASP1	Homo sapiens caspase 1, apoptosis-related cysteine peptidase (interleukin 1, beta, convertase) (CASP1), transcript variant alpha, mRNA [NM_033292]	A_23_P202978	7.76E-05	0.0028	1393.609	10.445
GBP3	Homo sapiens guanylate binding protein 3 (GBP3), mRNA [NM_018284]	A_23_P51487	7.74E-05	0.0028	21.45	4.423
IQCD	Homo sapiens IQ motif containing D (IQCD), mRNA [NM_138451]	A_24_P390060	7.88E-05	0.00283	4.637	2.213
TTC39C	Homo sapiens tetratricopeptide repeat domain 39C (TTC39C), transcript variant 2, mRNA [NM_153211]	A_23_P401098	7.87E-05	0.00283	11.974	3.582
LOC100131431	Q5S3G4_PIG (Q5S3G4) Mitochondrial cytochrome c oxidase subunit Vb, partial (63%) [THC2670107]	A_33_P3266025	0.000079	0.00283	5.748	2.523
GGPS1	Homo sapiens geranylgeranyl diphosphate synthase 1 (GGPS1), transcript variant 2, mRNA [NM_001037277]	A_33_P3258041	7.89E-05	0.00283	2.811	1.491
COMP	Homo sapiens cartilage oligomeric matrix protein (COMP), mRNA [NM_000095]	A_24_P264943	7.93E-05	0.00284	2.275	1.186
DDOST	Homo sapiens PTEN induced putative kinase 1 (PINK1), nuclear gene encoding mitochondrial protein, mRNA [NM_032409]	A_23_P23194	7.96E-05	0.00285	2.38	1.251
RP5-1000E10.4	Homo sapiens suppressor of IKBKE 1 (SIKE1), transcript variant 1, mRNA [NM_001102396]	A_24_P937855	8.04E-05	0.00286	3.482	1.8
TRPM6	Homo sapiens transient receptor potential cation channel, subfamily M, member 6 (TRPM6), transcript variant a, mRNA [NM_017662]	A_24_P410463	8.06E-05	0.00286	3.654	1.87

ZNF493	Homo sapiens zinc finger protein 493 (ZNF493), transcript variant 3, mRNA [NM_001076678]	A_32_P109036	8.14E-05	0.00287	3.556	1.83
TIPRL	Homo sapiens TIP41, TOR signaling pathway regulator-like (S. cerevisiae) (TIPRL), transcript variant 1, mRNA [NM_152902]	A_23_P327426	8.15E-05	0.00287	2.997	1.583
CETN2	Homo sapiens centrin, EF-hand protein, 2 (CETN2), mRNA [NM_004344]	A_23_P73493	8.13E-05	0.00287	3.133	1.647
C7orf53	Homo sapiens chromosome 7 open reading frame 53 (C7orf53), transcript variant 1, mRNA [NM_182597]	A_24_P380679	8.17E-05	0.00288	3.719	1.895
TNPO1	Homo sapiens transportin 1 (TNPO1), transcript variant 1, mRNA [NM_002270]	A_21_P0011577	8.26E-05	0.00289	2.355	1.235
DKK4	Homo sapiens dickkopf homolog 4 (Xenopus laevis) (DKK4), mRNA [NM_014420]	A_23_P94275	8.35E-05	0.00291	19.343	4.274
NUS1	Homo sapiens nuclear undecaprenyl pyrophosphate synthase 1 homolog (S. cerevisiae) (NUS1), mRNA [NM_138459]	A_21_P0011126	8.37E-05	0.00292	1.997	0.998
OSR2	Homo sapiens odd-skipped related 2 (Drosophila) (OSR2), transcript variant 2, mRNA [NM_053001]	A_24_P55496	8.44E-05	0.00293	6.937	2.794
CCDC138	Homo sapiens coiled-coil domain containing 138 (CCDC138), mRNA [NM_144978]	A_23_P311144	8.47E-05	0.00294	3.288	1.717
PDP1	Homo sapiens pyruvate dehyrogenase phosphatase catalytic subunit 1 (PDP1), nuclear gene encoding mitochondrial protein, transcript variant 2, mRNA [NM_001161779]	A_33_P3369761	8.46E-05	0.00294	6.241	2.642
FAM114A1	Homo sapiens family with sequence similarity 114, member A1 (FAM114A1), transcript variant 1, mRNA [NM_138389]	A_23_P132915	8.53E-05	0.00295	4.588	2.198
A_19_P00320842	Homo sapiens chromosome 17 open reading frame 51 (C17orf51), mRNA [NM_001113434]	A_19_P00320842	8.54E-05	0.00295	2.175	1.121
DHRS7	Homo sapiens dehydrogenase/reductase (SDR family) member 7 (DHRS7), mRNA [NM_016029]	A_23_P117506	8.52E-05	0.00295	3.433	1.779
M6PRBP1	Homo sapiens perilipin 3 (PLIN3), transcript variant 1, mRNA [NM_005817]	A_23_P101707	8.58E-05	0.00296	2.261	1.177
A_19_P00321063	Q5SRJ3_HUMAN (Q5SRJ3) Zinc finger protein 452 (Fragment), partial (3%) [THC2529829]	A_19_P00321063	8.59E-05	0.00296	18.718	4.226
BC033643	Homo sapiens cDNA clone IMAGE:5562656. [BC033643]	A_33_P3382271	8.62E-05	0.00297	2.557	1.354
TDRD9	Homo sapiens tudor domain containing 9 (TDRD9), mRNA [NM_153046]	A_32_P208350	8.75E-05	0.00299	239.731	7.905
PLEKHM3	Homo sapiens pleckstrin homology domain containing, family M, member 3 (PLEKHM3), mRNA [NM_001080475]	A_32_P89310	8.79E-05	0.00299	2.987	1.579
FEM1C	Homo sapiens fem-1 homolog c (C. elegans) (FEM1C), mRNA [NM_020177]	A_33_P3383871	8.79E-05	0.00299	3.072	1.619
AK096220	zinc finger, DHHC-type containing 11B [Source:HGNC Symbol;Acc:32962] [ENST00000522356]	A_33_P3345643	0.000088	0.00299	3.059	1.613
SLC25A24	Homo sapiens solute carrier family 25 (mitochondrial carrier; phosphate carrier), member 24 (SLC25A24), nuclear gene encoding mitochondrial protein, transcript variant 2, mRNA [NM_213651]	A_23_P74799	8.72E-05	0.00299	2.823	1.497
MUCL1	Homo sapiens mucin-like 1 (MUCL1), mRNA [NM_058173]	A_23_P150979	8.86E-05	0.003	7.03	2.814
A_19_P00315718	Homo sapiens family with sequence similarity 200, member B (FAM200B), mRNA [NM_001145191]	A_19_P00315718	8.82E-05	0.003	2.239	1.163
MTHFD1	Homo sapiens methylenetetrahydrofolate dehydrogenase (NADP+ dependent) 1, methenyltetrahydrofolate cyclohydrolase, formyltetrahydrofolate synthetase (MTHFD1), mRNA [NM_005956]	A_23_P117494	8.84E-05	0.003	2.114	1.08
TPH2	Homo sapiens tryptophan hydroxylase 2 (TPH2), mRNA [NM_173353]	A_24_P401787	8.89E-05	0.00301	54.004	5.755
IL13RA1	Homo sapiens interleukin 13 receptor, alpha 1 (IL13RA1), mRNA [NM_001560]	A_24_P280113	0.000089	0.00301	4.58	2.195
CARS2	Homo sapiens cysteinyl-tRNA synthetase 2, mitochondrial (putative) (CARS2), nuclear gene encoding mitochondrial protein, mRNA [NM_024537]	A_23_P128624	8.95E-05	0.00302	2.469	1.304
CLEC1A	Homo sapiens C-type lectin domain family 1, member A (CLEC1A), mRNA [NM_016511]	A_33_P3295333	9.07E-05	0.00304	36.141	5.176
SYNE1	Homo sapiens spectrin repeat containing, nuclear envelope 1 (SYNE1), transcript variant 1, mRNA [NM_182961]	A_23_P500861	9.18E-05	0.00306	49.122	5.618
ACSL1	Homo sapiens acyl-CoA synthetase long-chain family member 1 (ACSL1), mRNA [NM_001995]	A_23_P110212	9.22E-05	0.00307	10.156	3.344
MEF2C	Homo sapiens myocyte enhancer factor 2C (MEF2C), transcript variant 1, mRNA [NM_002397]	A_23_P320739	9.26E-05	0.00308	4.059	2.021
GLRXL	Unknown	A_24_P126741	9.29E-05	0.00308	17.097	4.096
LST1	Homo sapiens leukocyte specific transcript 1 (LST1), transcript variant 1, mRNA [NM_007161]	A_24_P103469	9.31E-05	0.00308	7.638	2.933
GP9	Homo sapiens glycoprotein IX (platelet) (GP9), mRNA [NM_000174]	A_33_P3383226	0.000093	0.00308	4.384	2.132
C4orf26	Homo sapiens chromosome 4 open reading frame 26 (C4orf26), transcript variant 2, mRNA [NM_178497]	A_23_P386268	9.26E-05	0.00308	10.919	3.449
RAB43	Homo sapiens RAB43, member RAS oncogene family (RAB43), transcript variant 1, mRNA [NM_198490]	A_24_P277295	9.32E-05	0.00308	2.857	1.515
MOCS2	Homo sapiens molybdenum cofactor synthesis 2 (MOCS2), transcript variant 3, mRNA [NM_004531]	A_23_P92967	9.39E-05	0.00309	2.281	1.19

LOC90826	Homo sapiens protein arginine methyltransferase 10 (putative) (PRMT10), mRNA [NM_138364]	A_23_P41541	9.38E-05	0.00309	3.222	1.688
LOC100131988	coenzyme Q5 homolog, methyltransferase (S. cerevisiae) [Source:HGNC Symbol;Acc:28722] [ENST00000551769]	A_33_P3213512	9.44E-05	0.00309	2.528	1.338
HEY2	Homo sapiens hairy/enhancer-of-split related with YRPW motif 2 (HEY2), mRNA [NM_012259]	A_24_P363408	9.42E-05	0.00309	6.01	2.587
HLA-A	Homo sapiens major histocompatibility complex, class I, A (HLA-A), transcript variant 1, mRNA [NM_002116]	A_23_P408353	9.38E-05	0.00309	3.659	1.872
SETD7	Homo sapiens SET domain containing (lysine methyltransferase) 7 (SETD7), mRNA [NM_030648]	A_24_P251841	0.000095	0.00311	6.612	2.725
SLC26A4	Homo sapiens solute carrier family 26, member 4 (SLC26A4), mRNA [NM_000441]	A_23_P331560	9.58E-05	0.00312	31.463	4.976
CLDN22	Homo sapiens claudin 22 (CLDN22), mRNA [NM_001111319]	A_33_P3291816	9.59E-05	0.00312	3.952	1.982
LOC100133124	PREDICTED: Homo sapiens cytochrome c oxidase subunit 7B, mitochondrial-like (LOC100133124), mRNA [XM_002342108]	A_21_P0013923	9.57E-05	0.00312	2.036	1.026
CYTIP	Homo sapiens cytohesin 1 interacting protein (CYTIP), mRNA [NM_004288]	A_23_P90626	9.57E-05	0.00312	63.765	5.995
SPATA1	spermatogenesis associated 1 [Source:HGNC Symbol;Acc:14682] [ENST00000431031]	A_23_P103897	9.64E-05	0.00313	3.363	1.75
ZC3H14	Homo sapiens zinc finger CCCH-type containing 14 (ZC3H14), transcript variant 1, mRNA [NM_024824]	A_24_P241330	9.65E-05	0.00313	2.038	1.027
FAM82A1	Homo sapiens family with sequence similarity 82, member A1 (FAM82A1), transcript variant 1, mRNA [NM_144713]	A_24_P131392	9.68E-05	0.00314	3.115	1.639
MAMLD1	Homo sapiens mastermind-like domain containing 1 (MAMLD1), transcript variant 2, mRNA [NM_005491]	A_23_P251075	9.76E-05	0.00316	4.997	2.321
PARP8	Homo sapiens poly (ADP-ribose) polymerase family, member 8 (PARP8), transcript variant 3, mRNA [NM_001178056]	A_21_P0010776	9.87E-05	0.00317	4.026	2.01
SEPT14	Homo sapiens septin 14 (SEPT14), mRNA [NM_207366]	A_21_P0011898	9.87E-05	0.00317	2.65	1.406
C12orf63	Unknown	A_21_P0011061	9.85E-05	0.00317	17.603	4.138
CACYBP	Homo sapiens calcyclin binding protein (CACYBP), transcript variant 1, mRNA [NM_014412]	A_32_P114574	0.0001	0.00318	2.296	1.199
SLC19A2	Homo sapiens solute carrier family 19 (thiamine transporter), member 2 (SLC19A2), mRNA [NM_006996]	A_23_P160466	0.0001	0.00318	2.708	1.437
ACOT12	Homo sapiens acyl-CoA thioesterase 12 (ACOT12), mRNA [NM_130767]	A_23_P44207	0.000101	0.0032	13.764	3.783
TNFSF13B	Homo sapiens tumor necrosis factor (ligand) superfamily, member 13b (TNFSF13B), transcript variant 1, mRNA [NM_006573]	A_23_P14174	0.000101	0.0032	11.49	3.522
CHRNA6	Homo sapiens cholinergic receptor, nicotinic, alpha 6 (CHRNA6), transcript variant 1, mRNA [NM_004198]	A_23_P157593	0.000101	0.0032	12.33	3.624
JARID1B	Homo sapiens lysine (K)-specific demethylase 5B (KDM5B), mRNA [NM_006618]	A_32_P70724	0.000102	0.00321	2.193	1.133
RABGAP1	Homo sapiens RAB GTPase activating protein 1 (RABGAP1), mRNA [NM_012197]	A_24_P252575	0.000102	0.00321	2.074	1.052
ATG12	Homo sapiens ATG12 autophagy related 12 homolog (S. cerevisiae) (ATG12), transcript variant 1, mRNA [NM_004707]	A_23_P20970	0.000103	0.00323	2.193	1.133
C1orf201	Homo sapiens chromosome 1 open reading frame 201 (C1orf201), transcript variant 3, mRNA [NM_178122]	A_24_P133162	0.000103	0.00323	3.501	1.808
BMP6	Homo sapiens bone morphogenetic protein 6 (BMP6), mRNA [NM_001718]	A_23_P19624	0.000103	0.00323	14.34	3.842
SERPINI1	Homo sapiens serpin peptidase inhibitor, clade I (neuroserpin), member 1 (SERPINI1), transcript variant 1, mRNA [NM_005025]	A_23_P166929	0.000103	0.00323	38.579	5.27
TRIM55	Homo sapiens tripartite motif containing 55 (TRIM55), transcript variant 2, mRNA [NM_033058]	A_33_P3850216	0.000103	0.00323	17.376	4.119
CLK1	Homo sapiens CDC-like kinase 1 (CLK1), transcript variant 1, mRNA [NM_004071]	A_23_P16817	0.000103	0.00323	2.037	1.027
BMP8A	Homo sapiens bone morphogenetic protein 8a (BMP8A), mRNA [NM_181809]	A_23_P85922	0.000106	0.0033	4.195	2.069
C6orf199	Homo sapiens adenylate kinase domain containing 1 (AKD1), transcript variant 2, mRNA [NM_145025]	A_23_P348749	0.000106	0.0033	18.201	4.186
C21orf91	Homo sapiens chromosome 21 open reading frame 91 (C21orf91), transcript variant 2, mRNA [NM_017447]	A_24_P125839	0.000107	0.00332	3.156	1.658
ENST00000398832	Uncharacterized protein [Source:UniProtKB/TrEMBL;Acc:A8MZ04] [ENST00000398832]	A_32_P207767	0.000108	0.00333	5.34	2.417
MAP6D1	Homo sapiens MAP6 domain containing 1 (MAP6D1), mRNA [NM_024871]	A_23_P80839	0.000108	0.00333	2.229	1.157
ENST00000333156	Putative golgin subfamily A member 6-like protein 11 [Source:UniProtKB/Swiss-Prot;Acc:A6NCC3] [ENST00000333156]	A_24_P316019	0.000109	0.00334	2.581	1.368
VCPIP1	Homo sapiens valosin containing protein (p97)/p47 complex interacting protein 1 (VCPIP1), mRNA [NM_025054]	A_33_P3223467	0.000109	0.00334	2.371	1.246

ZDHHC11	Homo sapiens zinc finger, DHHC-type containing 11 (ZDHHC11), mRNA [NM_024786]	A_33_P3344204	0.000109	0.00334	3.315	1.729
C17orf97	Homo sapiens chromosome 17 open reading frame 97 (C17orf97), mRNA [NM_001013672]	A_33_P3356320	0.000109	0.00334	5.709	2.513
EEA1	Homo sapiens early endosome antigen 1 (EEA1), mRNA [NM_003566]	A_23_P76159	0.00011	0.00335	3.219	1.687
TIAM2	Homo sapiens T-cell lymphoma invasion and metastasis 2 (TIAM2), transcript variant 1, mRNA [NM_012454]	A_24_P303454	0.00011	0.00335	3.541	1.824
MOSPD2	Homo sapiens motile sperm domain containing 2 (MOSPD2), transcript variant 1, mRNA [NM_152581]	A_23_P406986	0.00011	0.00335	2.159	1.111
AK7	Homo sapiens adenylate kinase 7 (AK7), mRNA [NM_152327]	A_23_P105963	0.000111	0.00337	3.743	1.904
A_19_P00322856	Homo sapiens family with sequence similarity 200, member B (FAM200B), mRNA [NM_001145191]	A_19_P00322856	0.000112	0.00338	2.206	1.142
CR591103	full-length cDNA clone CS0DL001YG15 of B cells (Ramos cell line) Cot 25-normalized of Homo sapiens (human) [CR591103]	A_33_P3336557	0.000112	0.00338	6.048	2.597
SEC61G	Homo sapiens Sec61 gamma subunit (SEC61G), transcript variant 1, mRNA [NM_014302]	A_23_P71241	0.000112	0.00338	2.782	1.476
TMEM133	Homo sapiens transmembrane protein 133 (TMEM133), mRNA [NM_032021]	A_23_P150325	0.000112	0.00338	6.016	2.589
IQCK	Homo sapiens IQ motif containing K (IQCK), mRNA [NM_153208]	A_23_P324523	0.000113	0.0034	2.181	1.125
PEX11B	Homo sapiens peroxisomal biogenesis factor 11 beta (PEX11B), transcript variant 1, mRNA [NM_003846]	A_24_P88554	0.000114	0.00341	2.191	1.132
MSL3	Homo sapiens male-specific lethal 3 homolog (Drosophila) (MSL3), transcript variant 1, mRNA [NM_078629]	A_23_P217778	0.000114	0.00341	2.443	1.289
PPAT	Homo sapiens phosphoribosyl pyrophosphate amidotransferase (PPAT), mRNA [NM_002703]	A_24_P123347	0.000114	0.00341	2.104	1.073
IFRD1	Homo sapiens interferon-related developmental regulator 1 (IFRD1), transcript variant 2, mRNA [NM_001007245]	A_24_P137897	0.000114	0.00341	5.545	2.471
CDH26	Homo sapiens cadherin 26 (CDH26), transcript variant b, mRNA [NM_021810]	A_23_P502957	0.000114	0.00341	20.197	4.336
FAM119A	Homo sapiens methyltransferase like 21A (METTL21A), transcript variant 1, mRNA [NM_145280]	A_23_P209337	0.000116	0.00346	2.935	1.553
A_19_P00808208	Homo sapiens cell division cycle associated 7-like (CDCA7L), transcript variant 2, mRNA [NM_001127370]	A_19_P00808208	0.000116	0.00346	4.93	2.302
CANX	Homo sapiens calnexin (CANX), transcript variant 1, mRNA [NM_001746]	A_24_P345993	0.000116	0.00346	4.608	2.204
ENST00000395453	SR-related CTD-associated factor 11 [Source:HGNC Symbol;Acc:10784] [ENST00000395453]	A_33_P3283196	0.000117	0.00348	3.215	1.685
NAV2	Homo sapiens neuron navigator 2 (NAV2), transcript variant 1, mRNA [NM_182964]	A_23_P52727	0.000118	0.00349	6.795	2.765
ARID5B	Homo sapiens AT rich interactive domain 5B (MRF1-like) (ARID5B), transcript variant 1, mRNA [NM_032199]	A_33_P3324980	0.000118	0.00349	7.994	2.999
C10orf114	Homo sapiens chromosome 10 open reading frame 114 (C10orf114), mRNA [NM_001010911]	A_32_P86739	0.00012	0.00353	3.809	1.93
HSD17B3	Homo sapiens hydroxysteroid (17-beta) dehydrogenase 3 (HSD17B3), mRNA [NM_000197]	A_33_P3418662	0.00012	0.00353	9.749	3.285
ENPP2	Homo sapiens ectonucleotide pyrophosphatase/phosphodiesterase 2 (ENPP2), transcript variant 1, mRNA [NM_006209]	A_23_P94338	0.00012	0.00353	33.05	5.047
MREG	Homo sapiens melanoregulin (MREG), mRNA [NM_018000]	A_24_P351283	0.00012	0.00353	3.239	1.695
LRRK2	Homo sapiens cDNA FLJ45829 fis, clone NT2RP8006452. [AK127729]	A_33_P3389872	0.000121	0.00354	3.794	1.924
CDCA7L	Homo sapiens cell division cycle associated 7-like (CDCA7L), transcript variant 1, mRNA [NM_018719]	A_24_P274795	0.000121	0.00354	5.027	2.33
XAGE3	Homo sapiens X antigen family, member 3 (XAGE3), transcript variant 2, mRNA [NM_130776]	A_23_P114349	0.000123	0.00358	10.82	3.436
CAMSAP1L1	Homo sapiens calmodulin regulated spectrin-associated protein family, member 2 (CAMSAP2), mRNA [NM_203459]	A_23_P406702	0.000123	0.00358	4.552	2.187
GCS1	Homo sapiens mannosyl-oligosaccharide glucosidase (MOGS), transcript variant 1, mRNA [NM_006302]	A_23_P108662	0.000124	0.0036	2.024	1.017
SQRDL	Homo sapiens sulfide quinone reductase-like (yeast) (SQRDL), nuclear gene encoding mitochondrial protein, mRNA [NM_021199]	A_23_P3221	0.000125	0.00362	20.313	4.344
LRRC17	Homo sapiens leucine rich repeat containing 17 (LRRC17), transcript variant 2, mRNA [NM_005824]	A_23_P253958	0.000125	0.00362	11.62	3.539
AIM2	Homo sapiens absent in melanoma 2 (AIM2), mRNA [NM_004833]	A_32_P44394	0.000125	0.00362	23.311	4.543
C11orf88	Homo sapiens chromosome 11 open reading frame 88 (C11orf88), transcript variant 1, mRNA [NM_207430]	A_32_P225345	0.000126	0.00365	7.441	2.895
GPR18	Homo sapiens G protein-coupled receptor 18 (GPR18), transcript variant 1, mRNA [NM_005292]	A_23_P14165	0.000126	0.00365	35.574	5.153
ARMC2	Homo sapiens armadillo repeat containing 2 (ARMC2), mRNA [NM_032131]	A_24_P320604	0.000127	0.00366	7.769	2.958
C3orf19	Homo sapiens chromosome 3 open reading frame 19 (C3orf19), mRNA [NM_016474]	A_33_P3294881	0.000127	0.00366	2.672	1.418

KRT27	Homo sapiens keratin 27 (KRT27), mRNA [NM_181537]	A_23_P38584	0.000127	0.00366	6.292	2.653
SPINK2	Homo sapiens serine peptidase inhibitor, Kazal type 2 (acrosin-trypsin inhibitor) (SPINK2), mRNA [NM_021114]	A_23_P155688	0.000128	0.00367	16.497	4.044
HAND1	Homo sapiens heart and neural crest derivatives expressed 1 (HAND1), mRNA [NM_004821]	A_23_P58770	0.000128	0.00367	5.177	2.372
AGTPBP1	Homo sapiens ATP/GTP binding protein 1 (AGTPBP1), mRNA [NM_015239]	A_23_P169278	0.000129	0.00368	5.236	2.388
FBXO16	Homo sapiens F-box protein 16 (FBXO16), mRNA [NM_172366]	A_23_P168847	0.00013	0.0037	4.788	2.259
STBD1	Homo sapiens starch binding domain 1 (STBD1), mRNA [NM_003943]	A_23_P254079	0.000131	0.00372	4.801	2.263
CASC1	Homo sapiens cancer susceptibility candidate 1 (CASC1), transcript variant 3, mRNA [NM_001082972]	A_33_P3378790	0.000132	0.00374	4.969	2.313
DDAH1	Homo sapiens dimethylarginine dimethylaminohydrolase 1 (DDAH1), transcript variant 1, mRNA [NM_012137]	A_23_P201386	0.000133	0.00375	3.843	1.942
UXS1	Homo sapiens UDP-glucuronate decarboxylase 1 (UXS1), mRNA [NM_025076]	A_23_P67829	0.000133	0.00375	2.491	1.317
HELB	Homo sapiens helicase (DNA) B (HELB), mRNA [NM_033647]	A_23_P2294	0.000133	0.00375	2.813	1.492
NCF1	Homo sapiens neutrophil cytosolic factor 1 (NCF1), mRNA [NM_000265]	A_33_P3211432	0.000133	0.00375	61.317	5.938
VPS54	Homo sapiens vacuolar protein sorting 54 homolog (S. cerevisiae) (VPS54), transcript variant 1, mRNA [NM_016516]	A_23_P131737	0.000134	0.00376	2.122	1.086
LOC100133264	Unknown	A_33_P3405043	0.000134	0.00376	7.868	2.976
GPR183	Homo sapiens G protein-coupled receptor 183 (GPR183), mRNA [NM_004951]	A_23_P25566	0.000134	0.00376	4.034	2.012
RSU1	Homo sapiens Ras suppressor protein 1 (RSU1), transcript variant 1, mRNA [NM_012425]	A_33_P3390335	0.000135	0.00378	4.553	2.187
PGM3	Homo sapiens phosphoglucomutase 3 (PGM3), transcript variant 4, mRNA [NM_001199919]	A_21_P0000147	0.000136	0.00379	2.246	1.167
MAP7D3	Homo sapiens MAP7 domain containing 3 (MAP7D3), transcript variant 1, mRNA [NM_024597]	A_23_P11160	0.000136	0.00379	5.148	2.364
EXPH5	Homo sapiens exophilin 5 (EXPH5), mRNA [NM_015065]	A_23_P403335	0.000137	0.00381	3.021	1.595
NINJ2	Homo sapiens ninjurin 2 (NINJ2), mRNA [NM_016533]	A_23_P48109	0.000137	0.00381	3.835	1.939
METTL9	Homo sapiens methyltransferase like 9 (METTL9), transcript variant 1, mRNA [NM_016025]	A_24_P202139	0.000137	0.00381	6.083	2.605
LOC388813	PREDICTED: Homo sapiens uncharacterized protein ENSP00000383407-like (LOC388813), mRNA [XM_003403737]	A_33_P3346067	0.000137	0.00381	3.439	1.782
AHI1	Homo sapiens Abelson helper integration site 1 (AHI1), transcript variant 2, mRNA [NM_017651]	A_24_P38143	0.000138	0.00382	3.928	1.974
UBD	Homo sapiens ubiquitin D (UBD), mRNA [NM_006398]	A_23_P81898	0.000138	0.00382	41.992	5.392
RDH5	Homo sapiens retinol dehydrogenase 5 (11-cis/9-cis) (RDH5), transcript variant 2, mRNA [NM_002905]	A_24_P218814	0.000138	0.00382	2.848	1.51
AMACR	Homo sapiens alpha-methylacyl-CoA racemase (AMACR), nuclear gene encoding mitochondrial protein, transcript variant 3, mRNA [NM_001167595]	A_33_P3313245	0.00014	0.00386	4.669	2.223
RAB1A	Homo sapiens RAB1A, member RAS oncogene family (RAB1A), transcript variant 1, mRNA [NM_004161]	A_33_P3371055	0.000141	0.00388	2.137	1.096
MMP1	Homo sapiens matrix metallopeptidase 1 (interstitial collagenase) (MMP1), transcript variant 1, mRNA [NM_002421]	A_23_P1691	0.000141	0.00388	16.406	4.036
ABCC11	Homo sapiens ATP-binding cassette, sub-family C (CFTR/MRP), member 11 (ABCC11), transcript variant 2, mRNA [NM_033151]	A_23_P141076	0.000142	0.00389	6.454	2.69
IL18BP	Homo sapiens interleukin 18 binding protein (IL18BP), transcript variant A, mRNA [NM_173042]	A_33_P3228322	0.000142	0.00389	3.671	1.876
RNF20	Homo sapiens ring finger protein 20 (RNF20), mRNA [NM_019592]	A_24_P98385	0.000143	0.00391	2.926	1.549
GLT8D4	Homo sapiens glucoside xylosyltransferase 2 (GXYLT2), mRNA [NM_001080393]	A_24_P118196	0.000143	0.00391	5.122	2.357
FLJ38773	Homo sapiens cDNA FLJ38773 fis, clone KIDNE2018071. [AK096092]	A_33_P3343412	0.000143	0.00391	9.037	3.176
TKT	Homo sapiens transketolase (TKT), transcript variant 1, mRNA [NM_001064]	A_23_P92082	0.000143	0.00391	2.761	1.465
C9orf103	Homo sapiens chromosome 9 open reading frame 103 (C9orf103), transcript variant 1, mRNA [NM_001001551]	A_23_P123732	0.000144	0.00392	2.494	1.318
PNMA1	Homo sapiens paraneoplastic antigen MA1 (PNMA1), mRNA [NM_006029]	A_23_P99771	0.000145	0.00394	2.389	1.256
SAV1	Homo sapiens salvador homolog 1 (Drosophila) (SAV1), mRNA [NM_021818]	A_24_P287473	0.000146	0.00396	2.824	1.498
HSPA6	Homo sapiens heat shock 70kDa protein 6 (HSP70B') (HSPA6), mRNA [NM_002155]	A_23_P114903	0.000146	0.00396	3.961	1.986
FAM45A	Homo sapiens family with sequence similarity 45, member A (FAM45A), mRNA [NM_207009]	A_32_P138004	0.000147	0.00398	4.097	2.035

LRRC37A3	Homo sapiens leucine rich repeat containing 37, member A3 (LRRC37A3), mRNA [NM_199340]	A_33_P3226465	0.000147	0.00398	1.985	0.989
LOC729867	Homo sapiens cDNA FLJ35980 fis, clone TESTI2013546. [AK093299]	A_33_P3284858	0.000148	0.00399	3.778	1.918
ARHGAP17	Homo sapiens Rho GTPase activating protein 17 (ARHGAP17), transcript variant 1, mRNA [NM_001006634]	A_24_P401739	0.000148	0.00399	2.221	1.151
EHD4	Homo sapiens EH-domain containing 4 (EHD4), mRNA [NM_139265]	A_23_P163458	0.000149	0.004	4.97	2.313
LOC100506451	Unknown	A_21_P0007694	0.000149	0.004	35.73	5.159
ETS2	Homo sapiens v-ets erythroblastosis virus E26 oncogene homolog 2 (avian) (ETS2), mRNA [NM_005239]	A_24_P314179	0.000149	0.004	16.325	4.029
SCG2	Homo sapiens secretogranin II (SCG2), mRNA [NM_003469]	A_24_P88696	0.000149	0.004	3.832	1.938
HIGD1C	Homo sapiens HIG1 hypoxia inducible domain family, member 1C (HIGD1C), mRNA [NM_001109619]	A_33_P3252539	0.000152	0.00405	11.398	3.511
CELA1	Homo sapiens chymotrypsin-like elastase family, member 1 (CELA1), mRNA [NM_001971]	A_33_P3313810	0.000152	0.00405	17.774	4.152
BTG1	Homo sapiens B-cell translocation gene 1, anti-proliferative (BTG1), mRNA [NM_001731]	A_23_P87560	0.000152	0.00405	3.778	1.918
GLYATL1	Homo sapiens glycine-N-acyltransferase-like 1 (GLYATL1), transcript variant 1, mRNA [NM_080661]	A_23_P370666	0.000153	0.00406	12.792	3.677
XRCC5	Homo sapiens X-ray repair complementing defective repair in Chinese hamster cells 5 (double-strand-break rejoining) (XRCC5), mRNA [NM_021141]	A_24_P345498	0.000153	0.00406	2.275	1.186
VSNL1	Homo sapiens visinin-like 1 (VSNL1), mRNA [NM_003385]	A_23_P209978	0.000153	0.00406	6.243	2.642
STAB2	Homo sapiens stabilin 2 (STAB2), mRNA [NM_017564]	A_23_P162607	0.000153	0.00406	6.365	2.67
UBXN11	Homo sapiens UBX domain protein 11 (UBXN11), transcript variant 2, mRNA [NM_183008]	A_33_P3394031	0.000154	0.00408	3.075	1.621
ADIPOR2	Homo sapiens adiponectin receptor 2 (ADIPOR2), mRNA [NM_024551]	A_23_P48121	0.000154	0.00408	2.315	1.211
ELOVL5	Homo sapiens ELOVL fatty acid elongase 5 (ELOVL5), transcript variant 4, mRNA [NM_001242831]	A_33_P3273719	0.000155	0.00409	3.893	1.961
AADACL1	Homo sapiens neutral cholesterol ester hydrolase 1 (NCEH1), transcript variant 2, mRNA [NM_020792]	A_23_P132644	0.000156	0.0041	2.846	1.509
KIF1B	Homo sapiens kinesin family member 1B (KIF1B), transcript variant 2, mRNA [NM_183416]	A_24_P649624	0.000156	0.0041	2.368	1.243
ENST00000390301	immunoglobulin lambda variable 1-36 [Source:HGNC Symbol;Acc:5876] [ENST00000390301]	A_33_P3372647	0.000156	0.0041	3.001	1.586
TMEM63A	Homo sapiens transmembrane protein 63A (TMEM63A), mRNA [NM_014698]	A_23_P200489	0.000157	0.00412	2.385	1.254
MSRA	Homo sapiens methionine sulfoxide reductase A (MSRA), transcript variant 1, mRNA [NM_012331]	A_23_P61426	0.000159	0.00415	8.022	3.004
DENND2C	Homo sapiens DENN/MADD domain containing 2C (DENND2C), mRNA [NM_198459]	A_24_P6467	0.000159	0.00415	4.755	2.249
CD302	Homo sapiens CD302 molecule (CD302), transcript variant 1, mRNA [NM_014880]	A_23_P131435	0.000161	0.00418	63.974	5.999
NECAB2	Homo sapiens N-terminal EF-hand calcium binding protein 2 (NECAB2), mRNA [NM_019065]	A_23_P66011	0.000162	0.00419	8.411	3.072
BATF3	Homo sapiens basic leucine zipper transcription factor, ATF-like 3 (BATF3), mRNA [NM_018664]	A_23_P160720	0.000163	0.00421	15.981	3.998
CAPRIN2	Homo sapiens caprin family member 2 (CAPRIN2), transcript variant 1, mRNA [NM_001002259]	A_23_P87532	0.000163	0.00421	2.83	1.501
FLJ26332	Homo sapiens cDNA FLJ26332 fis, clone HRT02453. [AK129842]	A_33_P3781228	0.000163	0.00421	8.496	3.087
FTO	Homo sapiens fat mass and obesity associated (FTO), mRNA [NM_001080432]	A_23_P113184	0.000165	0.00424	2.681	1.423
AP3S1	Homo sapiens adaptor-related protein complex 3, sigma 1 subunit (AP3S1), mRNA [NM_001284]	A_33_P3429576	0.000166	0.00425	2.764	1.467
PTX3	Homo sapiens pentraxin 3, long (PTX3), mRNA [NM_002852]	A_23_P121064	0.000166	0.00425	227.936	7.832
COMMD8	Homo sapiens COMM domain containing 8 (COMMD8), mRNA [NM_017845]	A_23_P44257	0.000167	0.00427	2.114	1.08
UBE2E1	Homo sapiens ubiquitin-conjugating enzyme E2E 1 (UBE2E1), transcript variant 1, mRNA [NM_003341]	A_23_P6963	0.000168	0.00429	2.313	1.21
DKFZp761E198	Homo sapiens uncharacterized protein DKFZp761E198 (DKFZp761E198), mRNA [NM_138368]	A_24_P9883	0.000169	0.00431	3.379	1.756
AKR1C4	Homo sapiens aldo-keto reductase family 1, member C4 (chlordecone reductase; 3-alpha hydroxysteroid dehydrogenase, type I; dihydrodiol dehydrogenase 4) (AKR1C4), mRNA [NM_001818]	A_33_P3272291	0.00017	0.00432	10.263	3.359
AK127966	Homo sapiens cDNA FLJ46080 fis, clone TESTI2004971. [AK127966]	A_33_P3212716	0.00017	0.00432	2.805	1.488
HNF1B	Homo sapiens HNF1 homeobox B (HNF1B), transcript variant 1, mRNA [NM_000458]	A_24_P330822	0.000171	0.00434	4.46	2.157
CSF1R	Homo sapiens colony stimulating factor 1 receptor (CSF1R), mRNA [NM_005211]	A_23_P110791	0.000171	0.00434	34.947	5.127
CTAGE1	Homo sapiens cutaneous T-cell lymphoma-associated antigen 1 (CTAGE1), transcript variant 1, mRNA [NM_172241]	A_24_P305223	0.000171	0.00434	4.977	2.315
TRIM26	Homo sapiens tripartite motif containing 26 (TRIM26), transcript variant 1, mRNA [NM_003449]	A_23_P214587	0.000172	0.00435	6.347	2.666
SDCCAG1	Homo sapiens nuclear export mediator factor (NEMF), mRNA [NM_004713]	A_23_P140328	0.000172	0.00435	2.234	1.159

SLC25A14	Homo sapiens solute carrier family 25 (mitochondrial carrier, brain), member 14 (SLC25A14), nuclear gene encoding mitochondrial protein, transcript variant long, mRNA [NM_003951]	A_23_P135966	0.000173	0.00435	2.102	1.072
LOC100127983	Homo sapiens uncharacterized protein LOC100127983 (LOC100127983), mRNA [NM_001190972]	A_24_P683011	0.000173	0.00435	2.813	1.492
PHACTR1	Homo sapiens phosphatase and actin regulator 1 (PHACTR1), transcript variant 1, mRNA [NM_030948]	A_32_P52018	0.000172	0.00435	2.892	1.532
NFKB1	Homo sapiens nuclear factor of kappa light polypeptide gene enhancer in B-cells 1 (NFKB1), transcript variant 1, mRNA [NM_003998]	A_23_P30024	0.000173	0.00435	2.275	1.186
KYNU	Homo sapiens kynureninase (KYNU), transcript variant 2, mRNA [NM_001032998]	A_24_P11506	0.000174	0.00437	6.648	2.733
NPHP3	Homo sapiens nephronophthisis 3 (adolescent) (NPHP3), mRNA [NM_153240]	A_33_P3228072	0.000175	0.00438	3.367	1.751
C7orf46	Homo sapiens chromosome 7 open reading frame 46 (C7orf46), transcript variant 1, mRNA [NM_199136]	A_33_P3308137	0.000175	0.00438	3.919	1.971
TIPARP	Homo sapiens TCDD-inducible poly(ADP-ribose) polymerase (TIPARP), transcript variant 2, mRNA [NM_015508]	A_23_P143845	0.000176	0.00439	2.819	1.495
MMP14	Homo sapiens matrix metallopeptidase 14 (membrane-inserted) (MMP14), mRNA [NM_004995]	A_24_P82106	0.000177	0.00441	2.208	1.142
RBP7	Homo sapiens retinol binding protein 7, cellular (RBP7), mRNA [NM_052960]	A_24_P165423	0.000177	0.00441	6.513	2.703
C6orf206	Homo sapiens radial spoke head 9 homolog (Chlamydomonas) (RSPH9), transcript variant 1, mRNA [NM_152732]	A_24_P55225	0.000178	0.00442	170.489	7.414
MAPK10	Homo sapiens mitogen-activated protein kinase 10 (MAPK10), transcript variant 3, mRNA [NM_138980]	A_23_P45025	0.000179	0.00444	16.259	4.023
SNORA20	Homo sapiens small nucleolar RNA, H/ACA box 20 (SNORA20), small nucleolar RNA [NR_002960]	A_21_P0000316	0.00018	0.00446	3.895	1.962
LOC285762	Homo sapiens cDNA FLJ39782 fis, clone SPLEN2002175. [AK097101]	A_33_P3773195	0.000181	0.00448	3.304	1.724
SLC17A5	Homo sapiens solute carrier family 17 (anion/sugar transporter), member 5 (SLC17A5), mRNA [NM_012434]	A_33_P3416321	0.000182	0.00449	5.298	2.405
LMO4	Homo sapiens LIM domain only 4 (LMO4), mRNA [NM_006769]	A_33_P3298425	0.000184	0.00453	3.06	1.613
DIMT1L	Homo sapiens DIM1 dimethyladenosine transferase 1 homolog (S. cerevisiae) (DIMT1), mRNA [NM_014473]	A_23_P58529	0.000184	0.00453	2.445	1.29
ACAD11	Homo sapiens acyl-CoA dehydrogenase family, member 11 (ACAD11), mRNA [NM_032169]	A_23_P212497	0.000186	0.00455	2.68	1.422
UPP1	Homo sapiens uridine phosphorylase 1 (UPP1), transcript variant 2, mRNA [NM_181597]	A_23_P351275	0.000186	0.00455	24.222	4.598
REC8	Homo sapiens REC8 homolog (yeast) (REC8), transcript variant 2, mRNA [NM_001048205]	A_33_P3278941	0.000187	0.00456	2.308	1.207
FSTL3	Homo sapiens follistatin-like 3 (secreted glycoprotein) (FSTL3), mRNA [NM_005860]	A_33_P3318796	0.000187	0.00456	2.477	1.309
KMO	Homo sapiens kynurenine 3-monooxygenase (kynurenine 3-hydroxylase) (KMO), mRNA [NM_003679]	A_23_P200838	0.000187	0.00456	2.768	1.469
CYS1	Homo sapiens cystin 1 (CYS1), mRNA [NM_001037160]	A_33_P3398156	0.000188	0.00458	6.354	2.668
UBTD2	Homo sapiens ubiquitin domain containing 2 (UBTD2), mRNA [NM_152277]	A_33_P3220565	0.000188	0.00458	10.826	3.436
SMG1	Homo sapiens smg-1 homolog, phosphatidylinositol 3-kinase-related kinase (C. elegans) (SMG1), mRNA [NM_015092]	A_24_P244952	0.000188	0.00458	3.297	1.721
CDS1	Homo sapiens CDP-diacylglycerol synthase (phosphatidate cytidylyltransferase) 1 (CDS1), mRNA [NM_001263]	A_23_P7250	0.00019	0.00461	15.232	3.929
CR620599	Uncharacterized protein [Source:UniProtKB/TrEMBL;Acc:F5H305] [ENST00000538264]	A_32_P88349	0.00019	0.00461	7.889	2.98
RAB27B	Homo sapiens RAB27B, member RAS oncogene family (RAB27B), mRNA [NM_004163]	A_21_P0009481	0.00019	0.00461	20.264	4.341
KCNMB4	Homo sapiens potassium large conductance calcium-activated channel, subfamily M, beta member 4 (KCNMB4), mRNA [NM_014505]	A_23_P64792	0.00019	0.00461	9.133	3.191
RGPD1	Homo sapiens RANBP2-like and GRIP domain containing 1 (RGPD1), mRNA [NM_001024457]	A_32_P776626	0.00019	0.00461	2.421	1.276
LIN28B	Homo sapiens lin-28 homolog B (C. elegans) (LIN28B), mRNA [NM_001004317]	A_33_P3220615	0.000192	0.00464	3.331	1.736
CSGALNACT2	Homo sapiens chondroitin sulfate N-acetylgalactosaminyltransferase 2 (CSGALNACT2), mRNA [NM_018590]	A_23_P149892	0.000193	0.00465	3.547	1.827
GON4L	Homo sapiens gon-4-like (C. elegans) (GON4L), transcript variant 2, mRNA [NM_032292]	A_33_P3221119	0.000194	0.00466	2.309	1.207
LCK	Homo sapiens lymphocyte-specific protein tyrosine kinase (LCK), transcript variant 2, mRNA [NM_005356]	A_33_P3382746	0.000194	0.00466	28.085	4.812
NQO2	Unknown	A_21_P0004718	0.000195	0.00468	4.405	2.139
C10orf97	Homo sapiens family with sequence similarity 188, member A (FAM188A), mRNA [NM_024948]	A_23_P23983	0.000197	0.00471	2.209	1.143

BG218865	PREDICTED: Homo sapiens phosphodiesterase 4D interacting protein-like (LOC645262), mRNA [XM_003403419]	A_32_P42725	0.000199	0.00474	8.555	3.097
NDUFAF2	Homo sapiens NADH dehydrogenase (ubiquinone) 1 alpha subcomplex, assembly factor 2 (NDUFAF2), nuclear gene encoding mitochondrial protein, mRNA [NM_174889]	A_23_P345942	0.000199	0.00474	2.364	1.241
FER1L6	Homo sapiens fer-1-like 6 (C. elegans) (FER1L6), mRNA [NM_001039112]	A_33_P3262171	0.0002	0.00476	6.531	2.707
ITIH2	Homo sapiens inter-alpha-trypsin inhibitor heavy chain 2 (ITIH2), mRNA [NM_002216]	A_23_P202053	0.000203	0.0048	4.368	2.127
ACCN3	Homo sapiens amiloride-sensitive cation channel 3 (ACCN3), transcript variant 3, mRNA [NM_020322]	A_23_P93658	0.000203	0.0048	2.407	1.267
MMP8	Homo sapiens matrix metallopeptidase 8 (neutrophil collagenase) (MMP8), mRNA [NM_002424]	A_23_P24493	0.000204	0.00481	33.693	5.074
TNRC8	Homo sapiens calcium/calmodulin-dependent serine protein kinase (MAGUK family) (CASK), transcript variant 1, mRNA [NM_003688]	A_32_P168464	0.000206	0.00483	2.894	1.533
DPYD	Homo sapiens dihydropyrimidine dehydrogenase (DPYD), transcript variant 2, mRNA [NM_001160301]	A_24_P410610	0.000208	0.00486	21.851	4.45
ADHFE1	Homo sapiens alcohol dehydrogenase, iron containing, 1 (ADHFE1), nuclear gene encoding mitochondrial protein, mRNA [NM_144650]	A_23_P157569	0.000208	0.00486	6.805	2.767
PCDH9	Homo sapiens protocadherin 9, mRNA (cDNA clone MGC:167030 IMAGE:8860363), complete cds. [BC150296]	A_33_P3236661	0.000208	0.00486	8.685	3.118
ZCWPW1	Homo sapiens zinc finger, CW type with PWWP domain 1 (ZCWPW1), mRNA [NM_017984]	A_23_P70897	0.000209	0.00487	2.853	1.513
LOC439911	Homo sapiens cDNA FLJ45542 fis, clone BRTHA2033320. [AK127450]	A_33_P3551349	0.000209	0.00487	2.236	1.161
MAOB	Homo sapiens monoamine oxidase B (MAOB), nuclear gene encoding mitochondrial protein, mRNA [NM_000898]	A_23_P85015	0.000209	0.00487	4.45	2.154
CTNNAL1	Homo sapiens catenin (cadherin-associated protein), alpha-like 1 (CTNNAL1), mRNA [NM_003798]	A_23_P157795	0.00021	0.00488	2.147	1.102
WDR78	Homo sapiens WD repeat domain 78 (WDR78), transcript variant 2, mRNA [NM_207014]	A_23_P200670	0.000211	0.00489	5.011	2.325
RPGRIP1	Homo sapiens retinitis pigmentosa GTPase regulator interacting protein 1 (RPGRIP1), mRNA [NM_020366]	A_23_P88278	0.000211	0.00489	19.055	4.252
RAB21	Homo sapiens RAB21, member RAS oncogene family (RAB21), mRNA [NM_014999]	A_23_P128166	0.000211	0.00489	2.702	1.434
FBXW10	Homo sapiens F-box and WD repeat domain containing 10 (FBXW10), mRNA [NM_031456]	A_23_P218358	0.000212	0.00491	4.086	2.031
OFD1	Homo sapiens oral-facial-digital syndrome 1 (OFD1), mRNA [NM_003611]	A_24_P134653	0.000213	0.00493	2.052	1.037
CCDC46	Homo sapiens centrosomal protein 112kDa (CEP112), transcript variant 1, mRNA [NM_145036]	A_23_P83976	0.000214	0.00495	3.59	1.844
C5AR1	Homo sapiens complement component 5a receptor 1 (C5AR1), mRNA [NM_001736]	A_23_P153562	0.000214	0.00495	15.074	3.914
ZSWIM7	Homo sapiens zinc finger, SWIM-type containing 7 (ZSWIM7), transcript variant 1, mRNA [NM_001042697]	A_33_P3269723	0.000215	0.00497	2.235	1.161
LOC401312	Homo sapiens LOC401318, mRNA (cDNA clone IMAGE:4827688). [BC042871]	A_33_P3455790	0.000216	0.00498	23.615	4.562
SLC4A5	Homo sapiens solute carrier family 4, sodium bicarbonate cotransporter, member 5 (SLC4A5), transcript variant c, mRNA [NM_133478]	A_33_P3241393	0.000217	0.00499	2.748	1.459
A_33_P3224800	Unknown	A_33_P3224800	0.000217	0.00499	33.782	5.078
NLRP11	Homo sapiens NLR family, pyrin domain containing 11 (NLRP11), mRNA [NM_145007]	A_32_P27479	0.000218	0.005	10.487	3.391
C20orf26	Homo sapiens chromosome 20 open reading frame 26 (C20orf26), transcript variant 1, mRNA [NM_015585]	A_32_P4262	0.000218	0.005	3.371	1.753
CR597597	full-length cDNA clone CS0DI013YN06 of Placenta Cot 25-normalized of Homo sapiens (human) [CR597597]	A_32_P76627	0.000218	0.005	10.031	3.326
ANKK1	Homo sapiens ankyrin repeat and kinase domain containing 1 (ANKK1), mRNA [NM_178510]	A_33_P3336053	0.000218	0.005	10.548	3.399
FH	Homo sapiens fumarate hydratase (FH), nuclear gene encoding mitochondrial protein, mRNA [NM_000143]	A_23_P34733	0.000218	0.005	2.349	1.232
LOC221946	Homo sapiens hypothetical protein LOC221946, mRNA (cDNA clone IMAGE:4825439). [BC044606]	A_33_P3503408	0.00022	0.00502	7.082	2.824
SLC12A1	Homo sapiens solute carrier family 12 (sodium/potassium/chloride transporters), member 1 (SLC12A1), transcript variant 1, mRNA [NM_000338]	A_33_P3282454	0.00022	0.00502	11.23	3.489
DAAM1	Homo sapiens dishevelled associated activator of morphogenesis 1 (DAAM1), mRNA [NM_014992]	A_23_P54116	0.000221	0.00504	2.15	1.104
ACOT1	Homo sapiens acyl-CoA thioesterase 1 (ACOT1), mRNA [NM_001037161]	A_24_P161036	0.000223	0.00505	6.474	2.695
TRIM49L	Homo sapiens ring finger protein 18-like (LOC399939), mRNA [NM_001206627]	A_33_P3374970	0.000222	0.00505	7.032	2.814
A_19_P00320451	Homo sapiens KH homology domain containing 1 (KHDC1), transcript variant 1, mRNA [NM_001251874]	A_19_P00320451	0.000223	0.00505	3.151	1.656
HIBADH	Homo sapiens 3-hydroxyisobutyrate dehydrogenase (HIBADH), mRNA [NM_152740]	A_23_P335428	0.000224	0.00507	2.692	1.429

CCDC109A	Homo sapiens mitochondrial calcium uniporter (MCU), nuclear gene encoding mitochondrial protein, mRNA [NM_138357]	A_23_P346405	0.000225	0.00508	2.791	1.481
RNF32	Homo sapiens ring finger protein 32 (RNF32), transcript variant 3, mRNA [NM_030936]	A_23_P19816	0.000225	0.00508	3.467	1.794
GRIN3A	Homo sapiens glutamate receptor, ionotropic, N-methyl-D-aspartate 3A (GRIN3A), mRNA [NM_133445]	A_23_P347541	0.000226	0.00509	23.7	4.567
COX7A2L	Homo sapiens cytochrome c oxidase subunit VIIa polypeptide 2 like (COX7A2L), nuclear gene encoding mitochondrial protein, mRNA [NM_004718]	A_24_P88800	0.000226	0.00509	3.395	1.764
SERF1A	Homo sapiens small EDRK-rich factor 1A (telomeric) (SERF1A), transcript variant 2, mRNA [NM_022968]	A_24_P346126	0.000227	0.0051	2.103	1.073
FAM45B	Homo sapiens family with sequence similarity 45, member A (FAM45A), mRNA [NM_207009]	A_24_P98555	0.000227	0.0051	3.978	1.992
INSL5	Homo sapiens insulin-like 5 (INSL5), mRNA [NM_005478]	A_23_P51479	0.000227	0.0051	3171.58	11.631
PEX11A	Homo sapiens peroxisomal biogenesis factor 11 alpha (PEX11A), mRNA [NM_003847]	A_23_P37560	0.000228	0.00512	3.814	1.931
NOP10	Homo sapiens NOP10 ribonucleoprotein homolog (yeast) (NOP10), mRNA [NM_018648]	A_23_P54477	0.000232	0.00516	2.929	1.551
GPR113	Homo sapiens G protein-coupled receptor 113 (GPR113), transcript variant 3, mRNA [NM_153835]	A_24_P85881	0.000232	0.00516	5.983	2.581
C5orf39	Homo sapiens chromosome 5 open reading frame 39 (C5orf39), mRNA [NM_001014279]	A_33_P3299279	0.000233	0.00517	7.961	2.993
LOC643454	Homo sapiens adaptor-related protein complex 3, sigma 1 subunit pseudogene, mRNA (cDNA clone IMAGE:8862705). [BC157883]	A_33_P3429575	0.000234	0.00519	2.789	1.48
CFL2	Homo sapiens cofilin 2 (muscle) (CFL2), transcript variant 1, mRNA [NM_021914]	A_24_P373152	0.000235	0.0052	3.444	1.784
PRR13	Homo sapiens proline rich 13 (PRR13), transcript variant 3, mRNA [NM_001005354]	A_23_P139575	0.000236	0.00521	2.048	1.034
FBXO27	Homo sapiens F-box protein 27 (FBXO27), mRNA [NM_178820]	A_24_P113264	0.000236	0.00521	3.019	1.594
GOLGA6L6	Homo sapiens golgin A6 family-like 6 (GOLGA6L6), mRNA [NM_001145004]	A_33_P3220025	0.000237	0.00522	7.235	2.855
ENST00000357508	Homo sapiens chromosome 1 open reading frame 228 (C1orf228), mRNA [NM_001145636]	A_32_P453321	0.000239	0.00524	3.1	1.632
A_33_P3421515	Unknown	A_33_P3421515	0.000238	0.00524	5.31	2.409
TNFRSF11A	Homo sapiens tumor necrosis factor receptor superfamily, member 11a, NFKB activator (TNFRSF11A), mRNA [NM_003839]	A_23_P390518	0.000239	0.00524	6.671	2.738
ATP2C1	Homo sapiens ATPase, Ca++ transporting, type 2C, member 1 (ATP2C1), transcript variant 3, mRNA [NM_001001485]	A_24_P137376	0.00024	0.00526	3.296	1.721
C4orf16	Homo sapiens adaptor-related protein complex 1 associated regulatory protein (AP1AR), transcript variant 1, mRNA [NM_018569]	A_23_P69791	0.000241	0.00526	2.851	1.511
ENST00000367534	actin related protein 2/3 complex, subunit 5, 16kDa [Source:HGNC Symbol;Acc:708] [ENST00000367534]	A_33_P3420235	0.00024	0.00526	4.213	2.075
ENST00000440451	coiled-coil domain containing 162, pseudogene [Source:HGNC Symbol;Acc:21565] [ENST00000440451]	A_33_P3333232	0.000241	0.00526	8.424	3.074
SCN3B	Homo sapiens sodium channel, voltage-gated, type III, beta (SCN3B), transcript variant 1, mRNA [NM_018400]	A_33_P3402404	0.000242	0.00527	4.653	2.218
ENST00000303697	Homo sapiens doublecortin domain containing 5 (DCDC5), mRNA [NM_020869]	A_23_P413862	0.000243	0.00529	3.518	1.815
GFI1	Homo sapiens growth factor independent 1 transcription repressor (GFI1), transcript variant 1, mRNA [NM_005263]	A_23_P257365	0.000243	0.00529	8.361	3.064
IKIP	Homo sapiens IKBKB interacting protein (IKBIP), transcript variant 1, mRNA [NM_153687]	A_23_P303210	0.000245	0.00532	1.677	0.746
LOC100292420	Unknown	A_33_P3338071	0.000246	0.00532	2.856	1.514
FLJ22662	Homo sapiens phospholipase B domain containing 1 (PLBD1), mRNA [NM_024829]	A_23_P87709	0.000246	0.00532	7.671	2.939
C8orf37	Homo sapiens chromosome 8 open reading frame 37 (C8orf37), mRNA [NM_177965]	A_33_P3293207	0.000248	0.00535	2.284	1.192
FLJ27352	Homo sapiens uncharacterized LOC145788 (FLJ27352), mRNA [NM_001198784]	A_32_P447001	0.00025	0.00538	2.943	1.557
PCNXL2	Homo sapiens pecanex-like 2 (Drosophila) (PCNXL2), transcript variant 1, mRNA [NM_014801]	A_23_P200260	0.00025	0.00538	4.019	2.007
COPZ2	Homo sapiens coatomer protein complex, subunit zeta 2 (COPZ2), mRNA [NM_016429]	A_23_P101093	0.000255	0.00546	4.149	2.053
MINA	Homo sapiens MYC induced nuclear antigen (MINA), transcript variant 3, mRNA [NM_032778]	A_24_P388570	0.000256	0.00547	2.422	1.276
PTGER4	Homo sapiens prostaglandin E receptor 4 (subtype EP4) (PTGER4), mRNA [NM_000958]	A_23_P148047	0.000256	0.00547	14.022	3.81
WHDC1	Homo sapiens WAS protein homolog associated with actin, golgi membranes and microtubules (WHAMM), mRNA [NM_001080435]	A_24_P307827	0.000258	0.00549	2.695	1.43
ENST00000254579	Homo sapiens dynein heavy chain domain 1 (DNHD1), transcript variant 1, mRNA [NM_144666]	A_32_P360193	0.000258	0.00549	4.496	2.169

MUC20	Homo sapiens mucin 20, cell surface associated (MUC20), transcript variant S, mRNA [NM_001098516]	A_33_P3226775	0.000257	0.00549	3.077	1.621
RNASE4	Homo sapiens ribonuclease, RNase A family, 4 (RNASE4), transcript variant 2, mRNA [NM_002937]	A_23_P205531	0.000258	0.00549	8.187	3.033
CYBRD1	Homo sapiens cytochrome b reductase 1 (CYBRD1), transcript variant 1, mRNA [NM_024843]	A_23_P209564	0.000258	0.00549	3.182	1.67
C4orf27	Homo sapiens chromosome 4 open reading frame 27 (C4orf27), mRNA [NM_017867]	A_24_P49371	0.000263	0.00556	2.938	1.555
RAB7L1	Homo sapiens RAB7, member RAS oncogene family-like 1 (RAB7L1), transcript variant 1, mRNA [NM_003929]	A_23_P126939	0.000263	0.00556	4.312	2.108
RNF112	Homo sapiens ring finger protein 112 (RNF112), mRNA [NM_007148]	A_23_P107116	0.000264	0.00557	3.982	1.994
C20orf177	Homo sapiens chromosome 20 open reading frame 177 (C20orf177), transcript variant 2, mRNA [NM_001190826]	A_23_P68505	0.000265	0.00558	2.983	1.577
A_19_P00320948	Unknown	A_19_P00320948	0.000265	0.00558	2.237	1.162
LOC100288600	MMFIBRLNA fibrillarin {Mus musculus} (exp=-1; wgp=0; cg=0), partial (8%) [THC2638856]	A_33_P3356413	0.000265	0.00558	4.369	2.127
PTGER3	Homo sapiens prostaglandin E receptor 3 (subtype EP3) (PTGER3), transcript variant 9, mRNA [NM_198719]	A_33_P3265739	0.000266	0.00559	2.89	1.531
SPTAN1	Homo sapiens spectrin, alpha, non-erythrocytic 1 (alpha-fodrin) (SPTAN1), transcript variant 1, mRNA [NM_001130438]	A_33_P3373560	0.000266	0.00559	5.089	2.347
ODZ1	Homo sapiens odz, odd Oz/ten-m homolog 1 (Drosophila) (ODZ1), transcript variant 1, mRNA [NM_001163278]	A_33_P3283863	0.000268	0.00562	6.039	2.594
ABCB11	Homo sapiens ATP-binding cassette, sub-family B (MDR/TAP), member 11 (ABCB11), mRNA [NM_003742]	A_23_P39703	0.000269	0.00564	5.13	2.359
NEK3	Homo sapiens NIMA (never in mitosis gene a)-related kinase 3 (NEK3), transcript variant 1, mRNA [NM_002498]	A_23_P333705	0.00027	0.00565	8.369	3.065
EIF4E3	Homo sapiens eukaryotic translation initiation factor 4E family member 3 (EIF4E3), transcript variant 2, mRNA [NM_173359]	A_24_P943113	0.000271	0.00566	4.54	2.183
VPS37A	Homo sapiens vacuolar protein sorting 37 homolog A (S. cerevisiae) (VPS37A), transcript variant 1, mRNA [NM_152415]	A_23_P366328	0.000272	0.00568	2.023	1.017
FLJ33065	Homo sapiens cDNA FLJ33065 fis, clone TRACH2000081. [AK057627]	A_33_P3780123	0.000274	0.00571	3.038	1.603
CRYBG3	beta-gamma crystallin domain containing 3 [Source:HGNC Symbol;Acc:34427] [ENST00000485253]	A_23_P303810	0.000277	0.00572	2.722	1.445
TP53I11	Homo sapiens tumor protein p53 inducible protein 11 (TP53I11), mRNA [NM_001076787]	A_24_P160969	0.000275	0.00572	10.675	3.416
SLC7A2	Homo sapiens solute carrier family 7 (cationic amino acid transporter, y+ system), member 2 (SLC7A2), transcript variant 2, mRNA [NM_001008539]	A_33_P3316539	0.000277	0.00572	3.838	1.94
CASZ1	Homo sapiens castor zinc finger 1 (CASZ1), transcript variant 1, mRNA [NM_001079843]	A_33_P3338300	0.000277	0.00572	3.361	1.749
AK123930	Homo sapiens cDNA FLJ41936 fis, clone PERIC2005370. [AK123930]	A_33_P3399881	0.000277	0.00572	5.417	2.437
EGLN3	Unknown	A_21_P0008419	0.000277	0.00572	4.603	2.203
GLDC	Homo sapiens glycine dehydrogenase (decarboxylating) (GLDC), nuclear gene encoding mitochondrial protein, mRNA [NM_000170]	A_23_P123596	0.000277	0.00572	2.01	1.007
TMED5	Homo sapiens transmembrane emp24 protein transport domain containing 5 (TMED5), transcript variant 1, mRNA [NM_016040]	A_24_P54178	0.000275	0.00572	2.58	1.367
ANKRD28	Homo sapiens ankyrin repeat domain 28 (ANKRD28), transcript variant 1, mRNA [NM_015199]	A_24_P217365	0.000276	0.00572	2.771	1.471
C6orf147	Homo sapiens KH homology domain containing 1 (KHDC1), transcript variant 1, mRNA [NM_001251874]	A_33_P3248842	0.000279	0.00575	3.101	1.633
LPO	Homo sapiens lactoperoxidase (LPO), transcript variant 1, mRNA [NM_006151]	A_23_P100583	0.000283	0.0058	17.497	4.129
IL6	Homo sapiens interleukin 6 (interferon, beta 2) (IL6), mRNA [NM_000600]	A_23_P71037	0.000285	0.00583	14.555	3.863
LOC100134937	Homo sapiens cDNA clone IMAGE:5263734. [BC035129]	A_33_P3427239	0.000286	0.00584	4.686	2.228
CCNG2	Homo sapiens cyclin G2 (CCNG2), mRNA [NM_004354]	A_33_P3385870	0.000289	0.00588	2.682	1.423
KIF27	Homo sapiens kinesin family member 27 (KIF27), mRNA [NM_017576]	A_24_P109071	0.000289	0.00588	10.852	3.44
STAT1	Homo sapiens signal transducer and activator of transcription 1, 91kDa (STAT1), transcript variant beta, mRNA [NM_139266]	A_24_P274270	0.000292	0.00593	4.23	2.081
ANXA3	Homo sapiens annexin A3 (ANXA3), mRNA [NM_005139]	A_23_P121716	0.000293	0.00594	16.104	4.009

CD55	Homo sapiens CD55 molecule, decay accelerating factor for complement (Cromer blood group) (CD55), transcript variant 1, mRNA [NM_000574]	A_24_P188377	0.000293	0.00594	3.053	1.61
DNHD1	Homo sapiens dynein heavy chain domain 1 (DNHD1), transcript variant 1, mRNA [NM_144666]	A_23_P316472	0.000294	0.00595	4.131	2.046
GFOD2	Homo sapiens glucose-fructose oxidoreductase domain containing 2 (GFOD2), transcript variant 1, mRNA [NM_030819]	A_23_P3574	0.000295	0.00596	2.604	1.38
MRPL18	Homo sapiens mitochondrial ribosomal protein L18 (MRPL18), nuclear gene encoding mitochondrial protein, mRNA [NM_014161]	A_23_P8339	0.000295	0.00596	2.055	1.039
SLC1A3	Homo sapiens solute carrier family 1 (glial high affinity glutamate transporter), member 3 (SLC1A3), transcript variant 1, mRNA [NM_004172]	A_24_P286114	0.000295	0.00596	17.495	4.129
ARSI	Homo sapiens arylsulfatase family, member I (ARSI), mRNA [NM_001012301]	A_23_P19030	0.000296	0.00597	3.513	1.813
ZBTB24	Homo sapiens zinc finger and BTB domain containing 24 (ZBTB24), transcript variant 1, mRNA [NM_014797]	A_23_P134147	0.000296	0.00597	2.021	1.015
MIPEP	Homo sapiens mitochondrial intermediate peptidase (MIPEP), nuclear gene encoding mitochondrial protein, mRNA [NM_005932]	A_33_P3404411	0.000299	0.00602	2.09	1.064
PTPLB	protein tyrosine phosphatase-like (proline instead of catalytic arginine), member b [Source:HGNC Symbol;Acc:9640] [ENST00000383657]	A_24_P135753	0.000299	0.00602	2.697	1.431
CCDC88B	Homo sapiens coiled-coil domain containing 88B (CCDC88B), mRNA [NM_032251]	A_23_P24389	0.0003	0.00603	2.026	1.018
TAF5	Homo sapiens TAF5 RNA polymerase II, TATA box binding protein (TBP)-associated factor, 100kDa (TAF5), mRNA [NM_006951]	A_23_P52311	0.000301	0.00604	3.066	1.617
VPS4B	Homo sapiens vacuolar protein sorting 4 homolog B (S. cerevisiae) (VPS4B), mRNA [NM_004869]	A_24_P322369	0.000302	0.00606	3.198	1.677
SLCO4C1	Homo sapiens solute carrier organic anion transporter family, member 4C1 (SLCO4C1), mRNA [NM_180991]	A_32_P154342	0.000303	0.00607	3.042	1.605
MOSPD1	Homo sapiens motile sperm domain containing 1 (MOSPD1), mRNA [NM_019556]	A_23_P73835	0.000304	0.00608	3.588	1.843
ZDBF2	Homo sapiens zinc finger, DBF-type containing 2 (ZDBF2), mRNA [NM_020923]	A_32_P68504	0.000305	0.00609	4.993	2.32
DDX4	Homo sapiens DEAD (Asp-Glu-Ala-Asp) box polypeptide 4 (DDX4), transcript variant 1, mRNA [NM_024415]	A_23_P256624	0.000307	0.0061	23.299	4.542
WIPI1	Homo sapiens WD repeat domain, phosphoinositide interacting 1 (WIPI1), mRNA [NM_017983]	A_23_P141394	0.000307	0.0061	7.89	2.98
THC2662344	Unknown	A_33_P3280009	0.000307	0.0061	2.2	1.138
RIN2	Homo sapiens Ras and Rab interactor 2 (RIN2), transcript variant 2, mRNA [NM_018993]	A_24_P305570	0.000307	0.0061	12.675	3.664
GABRE	Homo sapiens gamma-aminobutyric acid (GABA) A receptor, epsilon (GABRE), mRNA [NM_004961]	A_23_P159775	0.000309	0.00613	2.229	1.156
SOBP	Homo sapiens sine oculis binding protein homolog (Drosophila) (SOBP), mRNA [NM_018013]	A_33_P3363485	0.00031	0.00614	1.291	0.368
C1orf162	Homo sapiens chromosome 1 open reading frame 162 (C1orf162), mRNA [NM_174896]	A_23_P412562	0.000311	0.00615	3.434	1.78
HSDL2	Homo sapiens hydroxysteroid dehydrogenase like 2 (HSDL2), transcript variant 1, mRNA [NM_032303]	A_23_P169197	0.000312	0.00616	2.286	1.193
BST1	Homo sapiens bone marrow stromal cell antigen 1 (BST1), mRNA [NM_004334]	A_23_P7325	0.000313	0.00618	4.655	2.219
SIX3	Homo sapiens SIX homeobox 3 (SIX3), mRNA [NM_005413]	A_24_P34611	0.000315	0.0062	2.334	1.223
SLC15A4	Homo sapiens solute carrier family 15, member 4 (SLC15A4), mRNA [NM_145648]	A_24_P13311	0.000315	0.0062	2.367	1.243
ADPRHL2	Homo sapiens ADP-ribosylhydrolase like 2 (ADPRHL2), nuclear gene encoding mitochondrial protein, mRNA [NM_017825]	A_23_P34568	0.000315	0.0062	2.49	1.316
LOC441294	Homo sapiens CTAGE family, member 15, pseudogene (CTAGE15P), mRNA [NM_001008747]	A_23_P402952	0.000318	0.00624	4.165	2.058
LRMP	Homo sapiens lymphoid-restricted membrane protein (LRMP), transcript variant 1, mRNA [NM_006152]	A_23_P98910	0.000322	0.00629	4.526	2.178
C12orf28	chromosome 12 open reading frame 28 [Source:HGNC Symbol;Acc:26316] [ENST00000299350]	A_32_P424761	0.000322	0.00629	3.344	1.742
C19orf38	Homo sapiens chromosome 19 open reading frame 38 (C19orf38), mRNA [NM_001136482]	A_33_P3411945	0.000322	0.00629	8.57	3.099
PTEN	Homo sapiens phosphatase and tensin homolog (PTEN), mRNA [NM_000314]	A_23_P98085	0.000323	0.00629	2.857	1.514
ENST00000375322	mitogen-activated protein kinase kinase kinase 8 [Source:HGNC Symbol;Acc:6860] [ENST00000375322]	A_33_P3246505	0.000326	0.00634	3.711	1.892
CCNJL	Homo sapiens cyclin J-like (CCNJL), mRNA [NM_024565]	A_23_P7684	0.000326	0.00634	10.023	3.325

CC2D2B	Homo sapiens coiled-coil and C2 domain containing 2B (CC2D2B), transcript variant 1, mRNA [NM_001159747]	A_33_P3222233	0.000327	0.00635	3.32	1.731
RDH10	Homo sapiens retinol dehydrogenase 10 (all-trans) (RDH10), mRNA [NM_172037]	A_32_P25050	0.000329	0.00637	3.256	1.703
FIG4	Homo sapiens FIG4 homolog, SAC1 lipid phosphatase domain containing (S. cerevisiae) (FIG4), mRNA [NM_014845]	A_23_P145541	0.000333	0.00643	6.102	2.609
ENST00000394253	ubiquitin specific peptidase 49 [Source:HGNC Symbol;Acc:20078] [ENST00000394253]	A_33_P3389967	0.000336	0.00647	4.195	2.069
SRGAP1	Homo sapiens SLIT-ROBO Rho GTPase activating protein 1 (SRGAP1), mRNA [NM_020762]	A_23_P162449	0.000337	0.00649	10.309	3.366
LOC728142	MED14 antisense RNA 1 (non-protein coding) [Source:HGNC Symbol;Acc:40162] [ENST00000456333]	A_32_P167396	0.000339	0.00651	7.978	2.996
ACAA2	Homo sapiens acetyl-CoA acyltransferase 2 (ACAA2), nuclear gene encoding mitochondrial protein, mRNA [NM_006111]	A_23_P89799	0.00034	0.00651	3.118	1.64
NAGS	Homo sapiens N-acetylglutamate synthase (NAGS), mRNA [NM_153006]	A_32_P32739	0.000341	0.00652	4.544	2.184
RRN3	Homo sapiens RRN3 RNA polymerase I transcription factor homolog (S. cerevisiae) (RRN3), mRNA [NM_018427]	A_24_P382017	0.000342	0.00652	2.818	1.495
MFAP3	Homo sapiens microfibrillar-associated protein 3 (MFAP3), transcript variant 3, mRNA [NM_001242336]	A_33_P3374463	0.000341	0.00652	2.554	1.352
PIGZ	Homo sapiens phosphatidylinositol glycan anchor biosynthesis, class Z (PIGZ), mRNA [NM_025163]	A_23_P143935	0.000344	0.00653	2.15	1.104
TTC39A	Homo sapiens tetratricopeptide repeat domain 39A (TTC39A), transcript variant 2, mRNA [NM_001080494]	A_23_P160214	0.000343	0.00653	3.743	1.904
MAPK1	Homo sapiens mitogen-activated protein kinase 1 (MAPK1), transcript variant 2, mRNA [NM_138957]	A_23_P257895	0.000343	0.00653	2.638	1.399
TIMM23B	Unknown	A_33_P3403356	0.000344	0.00653	4.408	2.14
IL17D	Homo sapiens interleukin 17D (IL17D), mRNA [NM_138284]	A_23_P345692	0.000346	0.00656	3.134	1.648
GSTA3	Homo sapiens glutathione S-transferase alpha 3 (GSTA3), mRNA [NM_000847]	A_33_P3257891	0.000349	0.00659	6.142	2.619
NR3C1	Homo sapiens nuclear receptor subfamily 3, group C, member 1 (glucocorticoid receptor) (NR3C1), transcript variant 5, mRNA [NM_001018077]	A_33_P3767927	0.000349	0.00659	2.837	1.504
SNX21	Homo sapiens sorting nexin family member 21 (SNX21), transcript variant 4, mRNA [NM_001042633]	A_24_P318897	0.000349	0.00659	2.817	1.494
NOS2	Homo sapiens nitric oxide synthase 2, inducible (NOS2), mRNA [NM_000625]	A_23_P502464	0.000354	0.00665	6.622	2.727
GCKR	Homo sapiens glucokinase (hexokinase 4) regulator (GCKR), mRNA [NM_001486]	A_23_P119886	0.000355	0.00667	3.425	1.776
MME	Homo sapiens membrane metallo-endopeptidase (MME), transcript variant 2b, mRNA [NM_007289]	A_33_P3370094	0.000356	0.00667	8.273	3.048
EML5	Homo sapiens echinoderm microtubule associated protein like 5 (EML5), mRNA [NM_183387]	A_33_P3230558	0.000356	0.00667	6.906	2.788
DIRC2	Homo sapiens disrupted in renal carcinoma 2 (DIRC2), mRNA [NM_032839]	A_23_P80778	0.000356	0.00667	2.137	1.096
NFKBIZ	Homo sapiens nuclear factor of kappa light polypeptide gene enhancer in B-cells inhibitor, zeta (NFKBIZ), transcript variant 1, mRNA [NM_031419]	A_23_P212089	0.000355	0.00667	9.209	3.203
AGBL2	Homo sapiens ATP/GTP binding protein-like 2 (AGBL2), mRNA [NM_024783]	A_32_P167705	0.000357	0.00668	4.073	2.026
ENST00000361500	PREDICTED: Homo sapiens similar to nucleolar protein family A, member 3 (LOC100289143), mRNA [XM_002343200]	A_24_P927474	0.000358	0.00669	3.559	1.832
CADPS	Homo sapiens Ca++-dependent secretion activator (CADPS), transcript variant 3, mRNA [NM_183393]	A_23_P69326	0.000361	0.00673	5.068	2.341
FBXW7	Homo sapiens F-box and WD repeat domain containing 7 (FBXW7), transcript variant 1, mRNA [NM_033632]	A_24_P174613	0.000361	0.00673	2.515	1.331
NBPF10	Homo sapiens cDNA FLJ43804 fis, clone TESTI4000970. [AK125792]	A_33_P3253476	0.000362	0.00674	4.126	2.045
C12orf54	Homo sapiens chromosome 12 open reading frame 54 (C12orf54), mRNA [NM_152319]	A_23_P405878	0.000365	0.00679	5.803	2.537
CCDC42	Homo sapiens coiled-coil domain containing 42 (CCDC42), transcript variant 1, mRNA [NM_144681]	A_23_P402176	0.000366	0.0068	6.455	2.691
THBS4	Homo sapiens thrombospondin 4 (THBS4), mRNA [NM_003248]	A_24_P260443	0.000366	0.0068	4.704	2.234
TNFSF10	Homo sapiens tumor necrosis factor (ligand) superfamily, member 10 (TNFSF10), transcript variant 1, mRNA [NM_003810]	A_23_P121253	0.000367	0.0068	14.899	3.897
CEBPE	Homo sapiens CCAAT/enhancer binding protein (C/EBP), epsilon (CEBPE), mRNA [NM_001805]	A_33_P3387991	0.000368	0.00681	121.623	6.926
PKP2	Homo sapiens plakophilin 2 (PKP2), transcript variant 2b, mRNA [NM_004572]	A_33_P3405728	0.000368	0.00681	3.819	1.933
PSEN1	Homo sapiens presenilin 1 (PSEN1), transcript variant 1, mRNA [NM_000021]	A_23_P205686	0.000369	0.00682	2.117	1.082
ADSS	Homo sapiens adenylosuccinate synthase (ADSS), mRNA [NM_001126]	A_23_P201432	0.00037	0.00684	4.028	2.01

SERPINB9	Homo sapiens serpin peptidase inhibitor, clade B (ovalbumin), member 9 (SERPINB9), mRNA [NM_004155]	A_24_P295010	0.000372	0.00687	2.716	1.441
SEZ6L	Homo sapiens seizure related 6 homolog (mouse)-like (SEZ6L), transcript variant 1, mRNA [NM_021115]	A_23_P80242	0.000374	0.00688	8.926	3.158
CSMD1	Homo sapiens CUB and Sushi multiple domains 1 (CSMD1), mRNA [NM_033225]	A_33_P3230244	0.000374	0.00688	6.24	2.642
IL12B	Homo sapiens interleukin 12B (natural killer cell stimulatory factor 2, cytotoxic lymphocyte maturation factor 2, p40) (IL12B), mRNA [NM_002187]	A_23_P7560	0.000373	0.00688	8.92	3.157
APBB1IP	Homo sapiens amyloid beta (A4) precursor protein-binding, family B, member 1 interacting protein (APBB1IP), mRNA [NM_019043]	A_23_P401700	0.000377	0.00692	54.179	5.76
USO1	Homo sapiens USO1 vesicle docking protein homolog (yeast) (USO1), mRNA [NM_003715]	A_23_P29855	0.00038	0.00695	3.631	1.86
SEPX1	Homo sapiens selenoprotein X, 1 (SEPX1), mRNA [NM_016332]	A_23_P129486	0.000382	0.00698	4.482	2.164
TTLL5	Homo sapiens tubulin tyrosine ligase-like family, member 5 (TTLL5), mRNA [NM_015072]	A_24_P321752	0.000383	0.00699	5.167	2.369
MFSD10	Homo sapiens major facilitator superfamily domain containing 10 (MFSD10), transcript variant 1, mRNA [NM_001120]	A_23_P41246	0.000384	0.007	2.355	1.236
KLHL32	Homo sapiens kelch-like 32 (Drosophila) (KLHL32), mRNA [NM_052904]	A_33_P3340655	0.000385	0.00701	2.605	1.381
OR8G2	Homo sapiens olfactory receptor, family 8, subfamily G, member 2 (OR8G2), mRNA [NM_001007249]	A_33_P3262089	0.000388	0.00704	2.636	1.399
CPD	Homo sapiens carboxypeptidase D (CPD), transcript variant 1, mRNA [NM_001304]	A_24_P53282	0.000387	0.00704	3.366	1.751
KIAA0556	Homo sapiens KIAA0556 (KIAA0556), mRNA [NM_015202]	A_23_P381203	0.00039	0.00706	2.474	1.307
LOC729684	Unknown	A_24_P662366	0.000391	0.00707	3.013	1.591
SERINC1	Homo sapiens serine incorporator 1 (SERINC1), mRNA [NM_020755]	A_24_P129232	0.000392	0.00708	2.741	1.455
LOC283116	Homo sapiens tripartite motif-containing protein LOC642612-like (LOC283116), mRNA [NM_001206626]	A_21_P0011007	0.000392	0.00708	3.471	1.795
SH2D3C	Homo sapiens SH2 domain containing 3C (SH2D3C), transcript variant 1, mRNA [NM_170600]	A_33_P3293918	0.000396	0.00714	2.644	1.403
BX117927	BX117927 Soares_NFL_T_GBC_S1 Homo sapiens cDNA clone IMAGp998E153901, mRNA sequence [BX117927]	A_33_P3246950	0.000398	0.00715	4.671	2.224
NIT2	Homo sapiens nitrilase family, member 2 (NIT2), mRNA [NM_020202]	A_24_P120346	0.0004	0.00717	2.425	1.278
NUP98	Homo sapiens nucleoporin 98kDa (NUP98), transcript variant 1, mRNA [NM_016320]	A_23_P203586	0.00041	0.00729	2.066	1.047
ALPK1	Homo sapiens alpha-kinase 1 (ALPK1), transcript variant 1, mRNA [NM_025144]	A_23_P133133	0.000414	0.00734	5.706	2.513
PHLPPL	Homo sapiens PH domain and leucine rich repeat protein phosphatase 2 (PHLPP2), mRNA [NM_015020]	A_23_P418234	0.000417	0.00738	3.023	1.596
SDCBP	Homo sapiens syndecan binding protein (syntenin) (SDCBP), transcript variant 1, mRNA [NM_005625]	A_23_P157580	0.000418	0.0074	2.767	1.468
C20orf3	Homo sapiens chromosome 20 open reading frame 3 (C20orf3), mRNA [NM_020531]	A_24_P297078	0.000421	0.00742	8.131	3.023
SIPA1L1	Homo sapiens signal-induced proliferation-associated 1 like 1 (SIPA1L1), mRNA [NM_015556]	A_23_P76969	0.000421	0.00742	2.512	1.329
SH3D19	Homo sapiens SH3 domain containing 19 (SH3D19), transcript variant 1, mRNA [NM_001009555]	A_23_P33364	0.000422	0.00743	5.019	2.328
HCP5P10	Homo sapiens HLA complex P5 pseudogene 10, mRNA (cDNA clone IMAGE:5588965). [BC035659]	A_33_P3399988	0.000425	0.00746	3.224	1.689
STIM1	Homo sapiens stromal interaction molecule 1 (STIM1), mRNA [NM_003156]	A_23_P53162	0.000427	0.00749	2.571	1.363
TCP11L2	Homo sapiens t-complex 11 (mouse)-like 2 (TCP11L2), mRNA [NM_152772]	A_23_P419107	0.000428	0.0075	4.673	2.224
CENPQ	Homo sapiens centromere protein Q (CENPQ), mRNA [NM_018132]	A_23_P70328	0.000429	0.00751	3.564	1.834
PRMT5	Homo sapiens protein arginine methyltransferase 5 (PRMT5), transcript variant 2, mRNA [NM_001039619]	A_23_P76851	0.00043	0.00752	3.762	1.911
FGGY	Homo sapiens FGGY carbohydrate kinase domain containing (FGGY), transcript variant 2, mRNA [NM_018291]	A_32_P209094	0.000431	0.00753	3.239	1.696
MYH15	Homo sapiens myosin, heavy chain 15 (MYH15), mRNA [NM_014981]	A_24_P11900	0.000431	0.00753	5.612	2.489
RNF13	Homo sapiens ring finger protein 13 (RNF13), transcript variant 1, mRNA [NM_007282]	A_21_P0014626	0.000431	0.00753	2.609	1.384
FOS	Homo sapiens FBJ murine osteosarcoma viral oncogene homolog (FOS), mRNA [NM_005252]	A_23_P106194	0.000432	0.00754	33.351	5.06
BC066989	Homo sapiens cDNA clone IMAGE:5267335. [BC066989]	A_32_P130577	0.000433	0.00755	2.176	1.122
TFB2M	Homo sapiens transcription factor B2, mitochondrial (TFB2M), nuclear gene encoding mitochondrial protein, mRNA [NM_022366]	A_23_P45940	0.000435	0.00757	2.438	1.286
RGPD5	Homo sapiens RANBP2-like and GRIP domain containing 5 (RGPD5), transcript variant 1, mRNA [NM_005054]	A_23_P218637	0.000435	0.00757	2.036	1.026

RTCD1	Homo sapiens RNA terminal phosphate cyclase domain 1 (RTCD1), transcript variant 2, mRNA [NM_003729]	A_23_P258621	0.000438	0.00761	2.064	1.046
PPP1R2	Homo sapiens protein phosphatase 1, regulatory (inhibitor) subunit 2 (PPP1R2), mRNA [NM_006241]	A_24_P174367	0.000438	0.00761	2.257	1.175
SPATA7	Homo sapiens spermatogenesis associated 7 (SPATA7), transcript variant 1, mRNA [NM_018418]	A_33_P3374563	0.000439	0.00762	2.68	1.422
SATL1	Homo sapiens spermidine/spermine N1-acetyl transferase-like 1 (SATL1), mRNA [NM_001012980]	A_33_P3223602	0.00044	0.00763	7.94	2.989
THC2685534	ALU7_HUMAN (P39194) Alu subfamily SQ sequence contamination warning entry, partial (9%) [THC2685534]	A_33_P3342792	0.000443	0.00765	2.521	1.334
DENND1B	Homo sapiens DENN/MADD domain containing 1B (DENND1B), transcript variant 4, mRNA [NM_001195216]	A_21_P0000124	0.000442	0.00765	3.341	1.74
NOMO1	NODAL modulator 1 [Source:HGNC Symbol;Acc:30060] [ENST00000287667]	A_33_P3299705	0.000445	0.00768	3.026	1.597
CHRM5	Homo sapiens cholinergic receptor, muscarinic 5 (CHRM5), mRNA [NM_012125]	A_23_P117873	0.000446	0.00769	7.783	2.96
LOC129293	Homo sapiens chromosome 2 open reading frame 89 (C2orf89), mRNA [NM_001080824]	A_23_P56703	0.000448	0.00771	4.896	2.291
C22orf9	Homo sapiens KIAA0930 (KIAA0930), transcript variant 1, mRNA [NM_015264]	A_23_P315589	0.00045	0.00774	2.601	1.379
BICD1	Homo sapiens bicaudal D homolog 1 (Drosophila) (BICD1), transcript variant 1, mRNA [NM_001714]	A_33_P3256425	0.000452	0.00777	3.179	1.669
ATG7	Homo sapiens ATG7 autophagy related 7 homolog (S. cerevisiae) (ATG7), transcript variant 1, mRNA [NM_006395]	A_23_P143987	0.000454	0.00779	1.995	0.996
IER3IP1	Homo sapiens immediate early response 3 interacting protein 1 (IER3IP1), mRNA [NM_016097]	A_23_P4474	0.000455	0.0078	3.006	1.588
ENST00000368941	FIG4 homolog, SAC1 lipid phosphatase domain containing (S. cerevisiae) [Source:HGNC Symbol;Acc:16873] [ENST00000368941]	A_33_P3337977	0.000456	0.00782	4.958	2.31
REEP5	Homo sapiens receptor accessory protein 5 (REEP5), mRNA [NM_005669]	A_23_P350045	0.000457	0.00783	2.21	1.144
GPER	Homo sapiens G protein-coupled estrogen receptor 1 (GPER), transcript variant 3, mRNA [NM_001039966]	A_33_P3345812	0.000458	0.00784	3.312	1.728
FSIP1	Homo sapiens fibrous sheath interacting protein 1 (FSIP1), mRNA [NM_152597]	A_23_P353125	0.00046	0.00786	2.193	1.133
PARL	Homo sapiens presenilin associated, rhomboid-like (PARL), nuclear gene encoding mitochondrial protein, transcript variant 1, mRNA [NM_018622]	A_33_P3335451	0.00046	0.00786	1.606	0.684
ARHGAP5	Homo sapiens Rho GTPase activating protein 5 (ARHGAP5), transcript variant 1, mRNA [NM_001030055]	A_33_P3242483	0.000466	0.00791	3.162	1.661
LOC727808	Homo sapiens cDNA FLJ42952 fis, clone BRSTN2008283. [AK124942]	A_33_P3346032	0.000465	0.00791	2.807	1.489
THC2686131	Unknown	A_33_P3411372	0.000466	0.00791	11.493	3.523
C17orf60	Homo sapiens mast cell immunoglobulin-like receptor 1 (MILR1), mRNA [NM_001085423]	A_24_P33982	0.000468	0.00793	6.415	2.681
ITSN1	Homo sapiens intersectin 1 (SH3 domain protein) (ITSN1), transcript variant 2, mRNA [NM_001001132]	A_23_P252962	0.000471	0.00794	2.294	1.198
AGAP7	Homo sapiens ArfGAP with GTPase domain, ankyrin repeat and PH domain 7 (AGAP7), mRNA [NM_001077685]	A_24_P729905	0.000472	0.00796	3.924	1.972
UGT2B4	Homo sapiens UDP glucuronosyltransferase 2 family, polypeptide B4 (UGT2B4), mRNA [NM_021139]	A_23_P386912	0.000473	0.00796	9.82	3.296
MEP1A	Homo sapiens meprin A, alpha (PABA peptide hydrolase) (MEP1A), mRNA [NM_005588]	A_24_P154868	0.00048	0.00803	11.618	3.538
SLAMF1	Homo sapiens signaling lymphocytic activation molecule family member 1 (SLAMF1), mRNA [NM_003037]	A_23_P62647	0.000479	0.00803	3.126	1.645
LOC100131000	Homo sapiens cDNA FLJ42370 fis, clone UTERU2030280. [AK124361]	A_33_P3245484	0.00048	0.00803	2.462	1.3
A2M	Homo sapiens alpha-2-macroglobulin (A2M), mRNA [NM_000014]	A_23_P116898	0.00048	0.00803	3.894	1.961
ALOX5AP	Homo sapiens arachidonate 5-lipoxygenase-activating protein (ALOX5AP), transcript variant 1, mRNA [NM_001629]	A_24_P347378	0.000482	0.00805	79.121	6.306
NAF1	Homo sapiens nuclear assembly factor 1 homolog (S. cerevisiae) (NAF1), transcript variant 1, mRNA [NM_138386]	A_24_P223163	0.000484	0.00808	2.411	1.27
DB161231	DB161231 THYMU3 Homo sapiens cDNA clone THYMU3041680 5', mRNA sequence [DB161231]	A_33_P3209244	0.000484	0.00808	4.693	2.23
RGS2	Homo sapiens regulator of G-protein signaling 2, 24kDa (RGS2), mRNA [NM_002923]	A_23_P114947	0.000485	0.00809	8.31	3.055
TUBB2A	Homo sapiens tubulin, beta 2A class IIa (TUBB2A), mRNA [NM_001069]	A_23_P19291	0.000486	0.00811	2.279	1.188
DUSP5	Homo sapiens dual specificity phosphatase 5 (DUSP5), mRNA [NM_004419]	A_23_P150018	0.000489	0.00814	2.988	1.579
LYRM1	Homo sapiens LYR motif containing 1 (LYRM1), transcript variant 1, mRNA [NM_020424]	A_23_P54929	0.000489	0.00814	3.522	1.816
C20orf106	Homo sapiens chromosome 20 open reading frame 106 (C20orf106), mRNA [NM_001012971]	A_24_P323941	0.00049	0.00815	3.763	1.912

NUDT7	Homo sapiens nudix (nucleoside diphosphate linked moiety X)-type motif 7 (NUDT7), transcript variant 3, mRNA [NM_001243657]	A_23_P3602	0.00049	0.00815	3.055	1.611
ENST00000398948	Down syndrome critical region gene 4 [Source:HGNC Symbol;Acc:3045] [ENST00000398948]	A_33_P3301932	0.000491	0.00816	5.613	2.489
MGAT4A	Homo sapiens mannosyl (alpha-1,3-)-glycoprotein beta-1,4-N-acetylglucosaminyltransferase, isozyme A (MGAT4A), transcript variant 1, mRNA [NM_012214]	A_23_P28507	0.000493	0.00819	7.435	2.894
C17orf67	Homo sapiens chromosome 17 open reading frame 67 (C17orf67), mRNA [NM_001085430]	A_24_P712562	0.000494	0.00819	3.518	1.815
DDR2	Homo sapiens discoidin domain receptor tyrosine kinase 2 (DDR2), transcript variant 1, mRNA [NM_001014796]	A_23_P452	0.000496	0.00822	4.92	2.299
C11orf54	Homo sapiens chromosome 11 open reading frame 54 (C11orf54), mRNA [NM_014039]	A_24_P201404	0.000497	0.00822	2.729	1.449
ENST00000367101	serine active site containing 1 [Source:HGNC Symbol;Acc:21061] [ENST00000367101]	A_33_P3418576	0.000496	0.00822	3.965	1.987
ENST00000366856	cornichon homolog 4 (Drosophila) [Source:HGNC Symbol;Acc:25013] [ENST00000366856]	A_33_P3318746	0.000496	0.00822	3.124	1.644
LAMA2	Homo sapiens laminin, alpha 2 (LAMA2), transcript variant 1, mRNA [NM_000426]	A_23_P70719	0.000497	0.00822	20.319	4.345
CHRNA5	Homo sapiens cholinergic receptor, nicotinic, alpha 5 (CHRNA5), mRNA [NM_000745]	A_23_P88691	0.0005	0.00825	5.367	2.424
TMEM67	Homo sapiens transmembrane protein 67 (TMEM67), transcript variant 1, mRNA [NM_153704]	A_23_P429581	0.0005	0.00825	16.159	4.014
NALCN	Homo sapiens sodium leak channel, non-selective (NALCN), mRNA [NM_052867]	A_33_P3230166	0.000503	0.00829	3.985	1.995
LOC286052	Homo sapiens cDNA FLJ37785 fis, clone BRHIP2028330. [AK095104]	A_33_P3783235	0.000504	0.0083	4.114	2.041
LOC285043	Homo sapiens, clone IMAGE:5173647, mRNA. [BC033511]	A_33_P3546033	0.000506	0.00831	3.492	1.804
LIMS1	Homo sapiens LIM and senescent cell antigen-like domains 1 (LIMS1), transcript variant 2, mRNA [NM_004987]	A_23_P210358	0.000507	0.00832	2.106	1.075
MEIS1	Homo sapiens Meis homeobox 1 (MEIS1), mRNA [NM_002398]	A_24_P319736	0.000508	0.00833	2.696	1.431
FBXO15	Homo sapiens F-box protein 15 (FBXO15), transcript variant 1, mRNA [NM_152676]	A_23_P342709	0.000509	0.00834	14.934	3.901
LATS2	Homo sapiens LATS, large tumor suppressor, homolog 2 (Drosophila) (LATS2), mRNA [NM_014572]	A_24_P70002	0.00051	0.00835	2.552	1.351
KIAA1045	Homo sapiens KIAA1045 (KIAA1045), mRNA [NM_015297]	A_23_P319232	0.000512	0.00837	12.236	3.613
AP4S1	Homo sapiens adaptor-related protein complex 4, sigma 1 subunit (AP4S1), transcript variant 1, mRNA [NM_007077]	A_24_P12281	0.000514	0.00839	3.321	1.732
NXPH3	Homo sapiens neurexophilin 3 (NXPH3), mRNA [NM_007225]	A_24_P150931	0.000515	0.0084	4.919	2.298
IL1B	Homo sapiens interleukin 1, beta (IL1B), mRNA [NM_000576]	A_23_P79518	0.000517	0.00842	26.601	4.733
TTC35	Homo sapiens tetratricopeptide repeat domain 35 (TTC35), mRNA [NM_014673]	A_23_P60002	0.000522	0.00846	2.024	1.017
PPP1R14C	Homo sapiens protein phosphatase 1, regulatory (inhibitor) subunit 14C (PPP1R14C), mRNA [NM_030949]	A_23_P45011	0.000521	0.00846	7.92	2.986
RAB9A	Homo sapiens RAB9A, member RAS oncogene family (RAB9A), transcript variant 1, mRNA [NM_004251]	A_23_P45389	0.000522	0.00846	3.106	1.635
GBP5	Homo sapiens guanylate binding protein 5 (GBP5), transcript variant 1, mRNA [NM_052942]	A_23_P74290	0.000522	0.00846	49.411	5.627
BC053652	gamma-glutamyltransferase 3 pseudogene [Source:HGNC Symbol;Acc:4252] [ENST00000412448]	A_33_P3357283	0.000526	0.00851	4.027	2.01
TMEM104	Homo sapiens transmembrane protein 104 (TMEM104), mRNA [NM_017728]	A_24_P284324	0.000531	0.00857	2.144	1.1
FMO2	Homo sapiens flavin containing monooxygenase 2 (non-functional) (FMO2), mRNA [NM_001460]	A_33_P3275702	0.000531	0.00857	6.241	2.642
RAB31	Homo sapiens RAB31, member RAS oncogene family (RAB31), mRNA [NM_006868]	A_24_P236799	0.000536	0.00863	3.462	1.792
SLC6A16	Homo sapiens solute carrier family 6, member 16 (SLC6A16), mRNA [NM_014037]	A_23_P130735	0.000538	0.00865	5.623	2.491
FLJ32810	Homo sapiens Rho GTPase activating protein 42 (ARHGAP42), mRNA [NM_152432]	A_33_P3243332	0.000538	0.00865	3.716	1.894
C17orf71	Homo sapiens smg-8 homolog, nonsense mediated mRNA decay factor (C. elegans) (SMG8), mRNA [NM_018149]	A_23_P66563	0.000538	0.00865	2.507	1.326
AKIRIN2	Homo sapiens akirin 2 (AKIRIN2), mRNA [NM_018064]	A_23_P428827	0.00054	0.00866	2.258	1.175
SMARCD3	Homo sapiens SWI/SNF related, matrix associated, actin dependent regulator of chromatin, subfamily d, member 3 (SMARCD3), transcript variant 2, mRNA [NM_003078]	A_23_P122852	0.000543	0.0087	5.253	2.393
ZNF391	Homo sapiens zinc finger protein 391 (ZNF391), mRNA [NM_001076781]	A_33_P3220090	0.000543	0.0087	5.291	2.404
S1PR1	Homo sapiens sphingosine-1-phosphate receptor 1 (S1PR1), mRNA [NM_001400]	A_23_P404481	0.000544	0.00871	20.893	4.385
ANKRD42	Homo sapiens ankyrin repeat domain 42 (ANKRD42), mRNA [NM_182603]	A_32_P69166	0.000546	0.00874	2.058	1.041
TNFSF4	Homo sapiens tumor necrosis factor (ligand) superfamily, member 4 (TNFSF4), mRNA [NM_003326]	A_23_P126836	0.00055	0.00878	3.332	1.736

SAGE1	Homo sapiens sarcoma antigen 1 (SAGE1), mRNA [NM_018666]	A_23_P21943	0.000552	0.0088	3.255	1.703
C7orf16	Homo sapiens protein phosphatase 1, regulatory subunit 17 (PPP1R17), transcript variant 1, mRNA [NM_006658]	A_23_P145724	0.000553	0.00881	87.807	6.456
ARID3B	Homo sapiens AT rich interactive domain 3B (BRIGHT-like) (ARID3B), mRNA [NM_006465]	A_23_P88580	0.000555	0.00881	2.296	1.199
PAGE2B	Homo sapiens P antigen family, member 2B (PAGE2B), mRNA [NM_001015038]	A_33_P3420862	0.000553	0.00881	8.083	3.015
EIF2C3	Homo sapiens eukaryotic translation initiation factor 2C, 3 (EIF2C3), transcript variant 1, mRNA [NM_024852]	A_33_P3244882	0.000557	0.00883	2.572	1.363
ACAD8	Homo sapiens acyl-CoA dehydrogenase family, member 8 (ACAD8), nuclear gene encoding mitochondrial protein, mRNA [NM_014384]	A_23_P47426	0.000559	0.00886	4.224	2.079
GABARAPL1	Homo sapiens GABA(A) receptor-associated protein like 1 (GABARAPL1), mRNA [NM_031412]	A_33_P3812669	0.000564	0.00892	2.908	1.54
CHIT1	Homo sapiens chitinase 1 (chitotriosidase) (CHIT1), mRNA [NM_003465]	A_23_P126278	0.000564	0.00892	55.249	5.788
DEFA3	Homo sapiens defensin, alpha 3, neutrophil-specific (DEFA3), mRNA [NM_005217]	A_23_P31816	0.000568	0.00895	7.983	2.997
ME2	Homo sapiens malic enzyme 2, NAD(+)-dependent, mitochondrial (ME2), nuclear gene encoding mitochondrial protein, transcript variant 1, mRNA [NM_002396]	A_23_P321261	0.000568	0.00895	2.11	1.077
OSGIN2	Homo sapiens oxidative stress induced growth inhibitor family member 2 (OSGIN2), transcript variant 2, mRNA [NM_004337]	A_23_P82859	0.000576	0.00904	3.122	1.642
SLAIN1	Homo sapiens SLAIN motif family, member 1 (SLAIN1), transcript variant 1, mRNA [NM_001040153]	A_23_P348146	0.000577	0.00905	3.34	1.74
KIAA1024	Homo sapiens KIAA1024 (KIAA1024), mRNA [NM_015206]	A_33_P3421490	0.000579	0.00906	4.684	2.228
TMEM123	Homo sapiens transmembrane protein 123 (TMEM123), mRNA [NM_052932]	A_24_P309415	0.000583	0.0091	2.335	1.223
CFLAR	Homo sapiens CASP8 and FADD-like apoptosis regulator (CFLAR), transcript variant 8, mRNA [NM_001202519]	A_21_P0001952	0.000589	0.00915	2.305	1.205
TSLP	Homo sapiens thymic stromal lymphopoietin (TSLP), transcript variant 1, mRNA [NM_033035]	A_33_P3221783	0.00059	0.00916	8.099	3.018
RPS27L	Homo sapiens ribosomal protein S27-like (RPS27L), mRNA [NM_015920]	A_23_P14734	0.00059	0.00916	2.781	1.475
FA2H	Homo sapiens fatty acid 2-hydroxylase (FA2H), mRNA [NM_024306]	A_23_P49448	0.000591	0.00917	5.284	2.402
FOLR3	Homo sapiens folate receptor 3 (gamma) (FOLR3), mRNA [NM_000804]	A_24_P252996	0.0006	0.00925	8.525	3.092
IL21R	Homo sapiens interleukin 21 receptor (IL21R), transcript variant 2, mRNA [NM_181078]	A_24_P227927	0.000599	0.00925	6.333	2.663
ENST00000373010	ubiquitin specific peptidase 49 [Source:HGNC Symbol;Acc:20078] [ENST00000373009]	A_33_P3389973	0.000603	0.00928	4.554	2.187
HEG1	Homo sapiens HEG homolog 1 (zebrafish) (HEG1), mRNA [NM_020733]	A_32_P166693	0.000604	0.00929	5.248	2.392
PPBP	Homo sapiens pro-platelet basic protein (chemokine (C-X-C motif) ligand 7) (PPBP), mRNA [NM_002704]	A_23_P121596	0.000609	0.00934	231.615	7.856
PAGE2	Homo sapiens P antigen family, member 2 (prostate associated) (PAGE2), mRNA [NM_207339]	A_32_P70927	0.000612	0.00936	7.824	2.968
A_33_P3217619	Unknown	A_33_P3217619	0.000614	0.00937	3.061	1.614
NCCRP1	Homo sapiens non-specific cytotoxic cell receptor protein 1 homolog (zebrafish) (NCCRP1), mRNA [NM_001001414]	A_33_P3411477	0.000614	0.00937	24.386	4.608
LOC283387	Homo sapiens hypothetical protein LOC283387, mRNA (cDNA clone IMAGE:4821676). [BC032840]	A_33_P3797403	0.000616	0.00938	3.761	1.911
CT45A1	Homo sapiens cancer/testis antigen family 45, member A1 (CT45A1), mRNA [NM_001017417]	A_21_P0013834	0.000616	0.00938	5.948	2.572
SPATA13	Homo sapiens spermatogenesis associated 13 (SPATA13), transcript variant 2, mRNA [NM_153023]	A_23_P390116	0.000618	0.00941	5.495	2.458
ENST00000329309	Homo sapiens uncharacterized LOC389493 (LOC389493), mRNA [NM_001145712]	A_32_P42964	0.000619	0.00942	2.241	1.164
DPCD	Homo sapiens deleted in primary ciliary dyskinesia homolog (mouse) (DPCD), mRNA [NM_015448]	A_33_P3286046	0.000619	0.00942	2.12	1.084
DNM3	Homo sapiens dynamin 3 (DNM3), transcript variant 1, mRNA [NM_015569]	A_23_P371266	0.000622	0.00945	4.435	2.149
EU030677	T cell receptor alpha constant [Source:HGNC Symbol;Acc:12029] [ENST00000478163]	A_33_P3237674	0.000623	0.00946	7.808	2.965
VIM	Homo sapiens vimentin (VIM), mRNA [NM_003380]	A_23_P161190	0.000627	0.00949	2.848	1.51
P2RX5	Homo sapiens purinergic receptor P2X, ligand-gated ion channel, 5 (P2RX5), transcript variant 1, mRNA [NM_002561]	A_23_P433785	0.000627	0.00949	2.503	1.324
MYLK4	Homo sapiens myosin light chain kinase family, member 4 (MYLK4), mRNA [NM_001012418]	A_33_P3252939	0.000629	0.00952	8.882	3.151
BC041646	chromosome 11 open reading frame 72 [Source:HGNC Symbol;Acc:26915] [ENST00000333139]	A_33_P3256902	0.000629	0.00952	2.145	1.101
FRZB	Homo sapiens frizzled-related protein (FRZB), mRNA [NM_001463]	A_23_P363778	0.00063	0.00952	6.168	2.625

BDKRB1	Homo sapiens bradykinin receptor B1 (BDKRB1), mRNA [NM_000710]	A_23_P128744	0.000631	0.00953	8.512	3.089
ACSL4	Homo sapiens acyl-CoA synthetase long-chain family member 4 (ACSL4), transcript variant 1, mRNA [NM_004458]	A_23_P217564	0.000642	0.00964	2.363	1.241
C2orf21	Homo sapiens unc-80 homolog (C. elegans) (UNC80), transcript variant 1, mRNA [NM_032504]	A_24_P817863	0.000643	0.00965	2.942	1.557
ADM	Homo sapiens adrenomedullin (ADM), mRNA [NM_001124]	A_23_P127948	0.000649	0.00972	11.477	3.521
P2RY1	Homo sapiens purinergic receptor P2Y, G-protein coupled, 1 (P2RY1), mRNA [NM_002563]	A_33_P3417150	0.000651	0.00973	2.624	1.392
LOXL2	Homo sapiens lysyl oxidase-like 2 (LOXL2), mRNA [NM_002318]	A_23_P111995	0.000654	0.00975	15.641	3.967
PLA2G10	Homo sapiens phospholipase A2, group X (PLA2G10), mRNA [NM_003561]	A_23_P88767	0.000653	0.00975	20.337	4.346
TMTC1	Homo sapiens transmembrane and tetratricopeptide repeat containing 1 (TMTC1), transcript variant 2, mRNA [NM_175861]	A_23_P203957	0.000653	0.00975	7.197	2.847
NDFIP1	Homo sapiens Nedd4 family interacting protein 1 (NDFIP1), mRNA [NM_030571]	A_24_P209113	0.000655	0.00976	2.52	1.333
KDM5B	Homo sapiens lysine (K)-specific demethylase 5B (KDM5B), mRNA [NM_006618]	A_33_P3276455	0.000656	0.00977	2.209	1.143
TP53INP2	Homo sapiens tumor protein p53 inducible nuclear protein 2 (TP53INP2), mRNA [NM_021202]	A_24_P357465	0.000657	0.00978	3.49	1.803
LRRC39	Homo sapiens leucine rich repeat containing 39 (LRRC39), mRNA [NM_144620]	A_23_P330578	0.000657	0.00978	2.42	1.275
MMP27	Homo sapiens matrix metallopeptidase 27 (MMP27), mRNA [NM_022122]	A_33_P3398316	0.000658	0.00979	14.229	3.831
TBK1	Homo sapiens TANK-binding kinase 1, mRNA (cDNA clone IMAGE:3938647), partial cds. [BC009864]	A_24_P918175	0.000663	0.00984	9.021	3.173
PLEKHA5	Homo sapiens pleckstrin homology domain containing, family A member 5 (PLEKHA5), transcript variant 4, mRNA [NM_001190860]	A_33_P3328772	0.000663	0.00984	4.432	2.148
LNX2	Homo sapiens ligand of numb-protein X 2 (LNX2), mRNA [NM_153371]	A_23_P402287	0.000668	0.00989	3.192	1.674
ITGAX	Homo sapiens integrin, alpha X (complement component 3 receptor 4 subunit) (ITGAX), mRNA [NM_000887]	A_23_P312132	0.000669	0.00989	14.249	3.833
SNTB2	Homo sapiens syntrophin, beta 2 (dystrophin-associated protein A1, 59kDa, basic component 2) (SNTB2), mRNA [NM_006750]	A_33_P3331906	0.000668	0.00989	3.189	1.673
HLA-F	Homo sapiens major histocompatibility complex, class I, F (HLA-F), transcript variant 2, mRNA [NM_018950]	A_33_P3379939	0.000669	0.00989	2.564	1.358
PNRC1	Homo sapiens proline-rich nuclear receptor coactivator 1 (PNRC1), mRNA [NM_006813]	A_33_P3279831	0.00067	0.00989	6.264	2.647
LARS2	Homo sapiens leucyl-tRNA synthetase 2, mitochondrial (LARS2), nuclear gene encoding mitochondrial protein, mRNA [NM_015340]	A_23_P212397	0.000669	0.00989	2.171	1.118
SRGN	Homo sapiens serglycin (SRGN), transcript variant 1, mRNA [NM_002727]	A_23_P86653	0.000682	0.01	11.098	3.472
THC2682657	Q4T5G2_TETNG (Q4T5G2) Chromosome undetermined SCAF9279, whole genome shotgun sequence. (Fragment), partial (5%) [THC2682657]	A_33_P3259943	0.000683	0.01	6.36	2.669
S100P	Homo sapiens S100 calcium binding protein P (S100P), mRNA [NM_005980]	A_23_P58266	0.000692	0.0101	56.411	5.818
LOC285147	Homo sapiens cDNA FLJ37202 fis, clone BRALZ2006734. [AK094521]	A_33_P3579984	0.000686	0.0101	4.025	2.009
LOC157562	Homo sapiens mRNA; cDNA DKFZp686N0244 (from clone DKFZp686N0244). [BX649145]	A_33_P3685572	0.000688	0.0101	2.543	1.346
COX11	Homo sapiens cDNA FLJ42819 fis, clone BRCAN2015464. [AK124809]	A_33_P3289113	0.000694	0.0101	2.023	1.017
LCN2	Homo sapiens lipocalin 2 (LCN2), mRNA [NM_005564]	A_23_P169437	0.000695	0.0101	50.571	5.66
HS3ST3B1	heparan sulfate (glucosamine) 3-O-sulfotransferase 3B1 [Source:HGNC Symbol;Acc:5198] [ENST00000360954]	A_24_P23625	0.000686	0.0101	2.514	1.33
LPIN2	Homo sapiens lipin 2 (LPIN2), mRNA [NM_014646]	A_24_P301557	0.000694	0.0101	2.216	1.148
LOC100128276	PREDICTED: Homo sapiens hypothetical protein LOC100508384 (LOC100508384), mRNA [XM_003119544]	A_33_P3326447	0.000701	0.0102	7.565	2.919
A_19_P00322906	Homo sapiens chromosome 17 open reading frame 51 (C17orf51), mRNA [NM_001113434]	A_19_P00322906	0.0007	0.0102	2.39	1.257
ZUFSP	Homo sapiens zinc finger with UFM1-specific peptidase domain (ZUFSP), mRNA [NM_145062]	A_32_P109522	0.000709	0.0103	3.02	1.595
TBRG1	Homo sapiens transforming growth factor beta regulator 1 (TBRG1), transcript variant 1, mRNA [NM_032811]	A_24_P21752	0.000713	0.0103	2.95	1.561

SLC16A9	Homo sapiens solute carrier family 16, member 9 (monocarboxylic acid transporter 9) (SLC16A9), mRNA [NM_194298]	A_23_P115726	0.000712	0.0103	3.765	1.913
HLX	Homo sapiens H2.0-like homeobox (HLX), mRNA [NM_021958]	A_23_P126266	0.000714	0.0103	15.228	3.929
SEC24A	Homo sapiens SEC24 family, member A (S. cerevisiae) (SEC24A), transcript variant 1, mRNA [NM_021982]	A_33_P3331085	0.00072	0.0103	2.071	1.05
C11orf91	Homo sapiens chromosome 11 open reading frame 91 (C11orf91), mRNA [NM_001166692]	A_33_P3409746	0.000711	0.0103	8.974	3.166
HES2	hairy and enhancer of split 2 (Drosophila) [Source:HGNC Symbol;Acc:16005] [ENST00000377836]	A_33_P3376365	0.000715	0.0103	3.108	1.636
LOC729178	Homo sapiens cDNA clone IMAGE:5266307. [BC035182]	A_33_P3214052	0.000712	0.0103	9.466	3.243
C11orf75	Homo sapiens chromosome 11 open reading frame 75 (C11orf75), mRNA [NM_020179]	A_23_P75430	0.000726	0.0104	2.889	1.531
AMY1C	Homo sapiens amylase, alpha 1C (salivary) (AMY1C), mRNA [NM_001008219]	A_23_P23611	0.000725	0.0104	9.092	3.185
FAM50B	Homo sapiens family with sequence similarity 50, member B (FAM50B), mRNA [NM_012135]	A_23_P8240	0.000724	0.0104	10.771	3.429
CILP	Homo sapiens cartilage intermediate layer protein, nucleotide pyrophosphohydrolase (CILP), mRNA [NM_003613]	A_23_P151895	0.000722	0.0104	6.056	2.598
ENST00000290390	Homo sapiens chromosome 2 open reading frame 81 (C2orf81), mRNA [NM_001145054]	A_23_P131449	0.00074	0.0105	2.714	1.44
UGCG	Homo sapiens UDP-glucose ceramide glucosyltransferase (UGCG), mRNA [NM_003358]	A_23_P313389	0.000734	0.0105	2.775	1.472
MEIG1	Homo sapiens meiosis expressed gene 1 homolog (mouse) (MEIG1), mRNA [NM_001080836]	A_32_P175715	0.000741	0.0105	2.583	1.369
E2F5	Homo sapiens E2F transcription factor 5, p130-binding (E2F5), transcript variant 1, mRNA [NM_001951]	A_23_P31721	0.00074	0.0105	2.316	1.212
GLCCI1	Homo sapiens glucocorticoid induced transcript 1 (GLCCI1), mRNA [NM_138426]	A_23_P336198	0.000742	0.0105	2.944	1.558
LOC100130819	DA197111 BRASW1 Homo sapiens cDNA clone BRASW1000106 5', mRNA sequence [DA197111]	A_33_P3393091	0.00074	0.0105	4.384	2.132
DKFZp547G183	Homo sapiens mRNA; cDNA DKFZp547G183 (from clone DKFZp547G183). [AL359570]	A_33_P3732466	0.000732	0.0105	2.117	1.082
C9orf9	Homo sapiens chromosome 9 open reading frame 9 (C9orf9), mRNA [NM_018956]	A_23_P32135	0.000739	0.0105	3.729	1.899
PSTPIP2	Homo sapiens proline-serine-threonine phosphatase interacting protein 2 (PSTPIP2), mRNA [NM_024430]	A_24_P322353	0.000731	0.0105	3.167	1.663
LRRC6	Homo sapiens leucine rich repeat containing 6 (LRRC6), mRNA [NM_012472]	A_23_P112004	0.000753	0.0106	4.341	2.118
LOC100128386	Homo sapiens cDNA FLJ42335 fis, clone TUTER2001286. [AK124326]	A_33_P3361267	0.00075	0.0106	4.357	2.123
FOXL1	Homo sapiens forkhead box L1 (FOXL1), mRNA [NM_005250]	A_33_P3355503	0.000753	0.0106	4.345	2.12
VSIG10	Homo sapiens V-set and immunoglobulin domain containing 10 (VSIG10), mRNA [NM_019086]	A_33_P3250133	0.000743	0.0106	5.435	2.442
DNAJB9	Homo sapiens DnaJ (Hsp40) homolog, subfamily B, member 9 (DNAJB9), mRNA [NM_012328]	A_23_P258944	0.000748	0.0106	3.526	1.818
PLK2	Homo sapiens polo-like kinase 2 (PLK2), transcript variant 1, mRNA [NM_006622]	A_23_P30254	0.000749	0.0106	11.312	3.5
IFFO2	Homo sapiens intermediate filament family orphan 2 (IFFO2), mRNA [NM_001136265]	A_23_P418031	0.000748	0.0106	7.032	2.814
STAT3	Homo sapiens signal transducer and activator of transcription 3 (acute-phase response factor) (STAT3), transcript variant 3, mRNA [NM_213662]	A_24_P116805	0.000753	0.0106	2.523	1.335
RNF185	Homo sapiens ring finger protein 185 (RNF185), transcript variant 1, mRNA [NM_152267]	A_32_P15421	0.000759	0.0107	2.109	1.077
TMEM140	Homo sapiens transmembrane protein 140 (TMEM140), mRNA [NM_018295]	A_24_P372134	0.000759	0.0107	3.094	1.629
TMEM184C	Homo sapiens transmembrane protein 184C (TMEM184C), mRNA [NM_018241]	A_23_P18739	0.000775	0.0108	3.447	1.785
ADC	Homo sapiens arginine decarboxylase (ADC), mRNA [NM_052998]	A_24_P11462	0.000784	0.0109	11.797	3.56
EGR2	Homo sapiens early growth response 2 (EGR2), transcript variant 1, mRNA [NM_000399]	A_23_P46936	0.000784	0.0109	4.771	2.254
TRPV1	Homo sapiens transient receptor potential cation channel, subfamily V, member 1 (TRPV1), transcript variant 3, mRNA [NM_080706]	A_23_P375281	0.000777	0.0109	3.775	1.917
TMEM31	Homo sapiens transmembrane protein 31 (TMEM31), mRNA [NM_182541]	A_23_P352717	0.000792	0.011	6.145	2.619
GBE1	Homo sapiens glucan (1,4-alpha-), branching enzyme 1 (GBE1), mRNA [NM_000158]	A_23_P121082	0.0008	0.0111	2.955	1.563
CCDC80	Homo sapiens coiled-coil domain containing 80 (CCDC80), transcript variant 1, mRNA [NM_199511]	A_23_P58082	0.000805	0.0111	22.341	4.482
SP9	Sp9 transcription factor homolog (mouse) [Source:HGNC Symbol;Acc:30690] [ENST00000394967]	A_33_P3383184	0.000804	0.0111	4.258	2.09
ANKRD22	Homo sapiens ankyrin repeat domain 22 (ANKRD22), mRNA [NM_144590]	A_33_P3407880	0.000799	0.0111	25.985	4.7
MCL1	Homo sapiens myeloid cell leukemia sequence 1 (BCL2-related) (MCL1), nuclear gene encoding mitochondrial protein, transcript variant 1, mRNA [NM_021960]	A_24_P336759	0.000804	0.0111	2.19	1.131
MANSC1	Homo sapiens MANSC domain containing 1 (MANSC1), mRNA [NM_018050]	A_23_P162211	0.000804	0.0111	2.326	1.218
FCHSD2	Homo sapiens FCH and double SH3 domains 2 (FCHSD2), mRNA [NM_014824]	A_23_P368278	0.000823	0.0112	2.046	1.033

BCAP29	Homo sapiens B-cell receptor-associated protein 29 (BCAP29), transcript variant 2, mRNA [NM_018844]	A_33_P3287562	0.000813	0.0112	3.618	1.855
TMEM38B	Homo sapiens transmembrane protein 38B (TMEM38B), mRNA [NM_018112]	A_33_P3401428	0.000818	0.0112	1.743	0.802
TESSP1	Homo sapiens protease, serine, 41 (PRSS41), mRNA [NM_001135086]	A_33_P3416634	0.000815	0.0112	16.47	4.042
MAP3K2	Homo sapiens mitogen-activated protein kinase kinase kinase 2 (MAP3K2), mRNA [NM_006609]	A_33_P3415440	0.000827	0.0113	2.021	1.015
C17orf51	K-EST0059882 S4SNU1 Homo sapiens cDNA clone S4SNU1-24-F04 5', mRNA sequence [BM782690]	A_21_P0011533	0.000826	0.0113	5.17	2.37
SGEF	Homo sapiens Rho guanine nucleotide exchange factor (GEF) 26 (ARHGEF26), transcript variant 1, mRNA [NM_001251962]	A_32_P5276	0.000837	0.0114	6.401	2.678
RETSAT	Homo sapiens retinol saturase (all-trans-retinol 13,14-reductase) (RETSAT), mRNA [NM_017750]	A_23_P209944	0.000849	0.0115	2.525	1.336
ZNF280C	Homo sapiens zinc finger protein 280C (ZNF280C), mRNA [NM_017666]	A_23_P314642	0.000843	0.0115	2.08	1.057
MRAS	Homo sapiens muscle RAS oncogene homolog (MRAS), transcript variant 1, mRNA [NM_012219]	A_24_P88850	0.000852	0.0115	4.426	2.146
TRIM50	Homo sapiens tripartite motif containing 50 (TRIM50), mRNA [NM_178125]	A_23_P308136	0.000863	0.0116	3.436	1.781
DUSP16	Homo sapiens dual specificity phosphatase 16 (DUSP16), mRNA [NM_030640]	A_24_P189739	0.000866	0.0116	3.63	1.86
A_19_P00316404	Homo sapiens chromosome 17 open reading frame 51 (C17orf51), mRNA [NM_001113434]	A_19_P00316404	0.000855	0.0116	2.27	1.183
IRF1	Homo sapiens interferon regulatory factor 1 (IRF1), mRNA [NM_002198]	A_23_P41765	0.000858	0.0116	2.482	1.311
C9orf98	Homo sapiens adenylate kinase 8 (AK8), mRNA [NM_152572]	A_23_P83200	0.000875	0.0117	2.512	1.329
TIAM1	Homo sapiens T-cell lymphoma invasion and metastasis 1 (TIAM1), mRNA [NM_003253]	A_33_P3381751	0.000877	0.0117	9.051	3.178
RP11-403I13.9	PREDICTED: Homo sapiens phosphodiesterase 4D interacting protein-like (LOC645262), mRNA [XM_003403419]	A_33_P3376205	0.000871	0.0117	3.212	1.684
RORA	Homo sapiens RAR-related orphan receptor A (RORA), transcript variant 2, mRNA [NM_134260]	A_23_P26124	0.000874	0.0117	21.638	4.436
TANK	Homo sapiens TRAF family member-associated NFKB activator (TANK), transcript variant 1, mRNA [NM_004180]	A_23_P154306	0.00088	0.0118	2.675	1.419
GNS	Homo sapiens glucosamine (N-acetyl)-6-sulfatase (GNS), mRNA [NM_002076]	A_33_P3261167	0.000887	0.0118	2.104	1.073
SPI1	Homo sapiens spleen focus forming virus (SFFV) proviral integration oncogene spi1 (SPI1), transcript variant 1, mRNA [NM_001080547]	A_33_P3489646	0.000887	0.0118	2.101	1.071
MYH8	Homo sapiens myosin, heavy chain 8, skeletal muscle, perinatal (MYH8), mRNA [NM_002472]	A_33_P3262729	0.000888	0.0118	4.943	2.305
RHOH	Homo sapiens ras homolog gene family, member H (RHOH), mRNA [NM_004310]	A_23_P58132	0.00088	0.0118	2.427	1.279
PFN2	Homo sapiens profilin 2 (PFN2), transcript variant 1, mRNA [NM_053024]	A_23_P253301	0.00089	0.0118	6.038	2.594
FIGF	Homo sapiens c-fos induced growth factor (vascular endothelial growth factor D) (FIGF), mRNA [NM_004469]	A_23_P45185	0.000902	0.0119	15.571	3.961
CCDC28A	Homo sapiens coiled-coil domain containing 28A (CCDC28A), mRNA [NM_015439]	A_23_P31085	0.000892	0.0119	2.393	1.259
LOC728675	Unknown	A_33_P3382125	0.000892	0.0119	3.026	1.597
MSRB3	Homo sapiens methionine sulfoxide reductase B3 (MSRB3), nuclear gene encoding mitochondrial protein, transcript variant 3, mRNA [NM_001193460]	A_33_P3278313	0.000899	0.0119	5.815	2.54
SLC16A10	Homo sapiens solute carrier family 16, member 10 (aromatic amino acid transporter) (SLC16A10), mRNA [NM_018593]	A_24_P98047	0.000902	0.0119	16.826	4.073
PDSS2	Homo sapiens prenyl (decaprenyl) diphosphate synthase, subunit 2 (PDSS2), mRNA [NM_020381]	A_33_P3214456	0.000909	0.012	2.552	1.352
MDGA1	Homo sapiens MAM domain containing glycosylphosphatidylinositol anchor 1 (MDGA1), mRNA [NM_153487]	A_23_P310460	0.000905	0.012	10.243	3.357
SEC63	Homo sapiens SEC63 homolog (S. cerevisiae) (SEC63), mRNA [NM_007214]	A_23_P214977	0.000908	0.012	2.5	1.322
VIT	Homo sapiens vitrin (VIT), transcript variant 1, mRNA [NM_053276]	A_23_P56578	0.000915	0.0121	3.389	1.761
AF339771	Homo sapiens clone IMAGE:1257951, mRNA sequence. [AF339771]	A_32_P231493	0.000921	0.0121	3.08	1.623
DAPP1	Homo sapiens dual adaptor of phosphotyrosine and 3-phosphoinositides (DAPP1), mRNA [NM_014395]	A_23_P255444	0.00093	0.0122	3.338	1.739
DECR1	Homo sapiens 2,4-dienoyl CoA reductase 1, mitochondrial (DECR1), nuclear gene encoding mitochondrial protein, mRNA [NM_001359]	A_24_P269619	0.000937	0.0122	2.546	1.348
ATP6V1B2	Homo sapiens ATPase, H+ transporting, lysosomal 56/58kDa, V1 subunit B2 (ATP6V1B2), mRNA [NM_001693]	A_23_P31844	0.000932	0.0122	2.346	1.23

STX19	Homo sapiens syntaxin 19 (STX19), mRNA [NM_001001850]	A_33_P3269208	0.000935	0.0122	4.582	2.196
RAB6A	Homo sapiens RAB6A, member RAS oncogene family, mRNA (cDNA clone IMAGE:5752233), partial cds. [BC044241]	A_33_P3393088	0.000929	0.0122	4.553	2.187
MNX1	Homo sapiens motor neuron and pancreas homeobox 1 (MNX1), transcript variant 1, mRNA [NM_005515]	A_32_P6015	0.000935	0.0122	3.514	1.813
TLR1	Homo sapiens toll-like receptor 1 (TLR1), mRNA [NM_003263]	A_23_P10873	0.000926	0.0122	4.24	2.084
EFCAB3	Homo sapiens EF-hand calcium binding domain 3 (EFCAB3), transcript variant 2, mRNA [NM_173503]	A_32_P515971	0.000927	0.0122	4.044	2.016
SAP30	Homo sapiens Sin3A-associated protein, 30kDa (SAP30), mRNA [NM_003864]	A_23_P121602	0.000947	0.0123	2.271	1.183
SLC22A15	Homo sapiens solute carrier family 22, member 15 (SLC22A15), mRNA [NM_018420]	A_23_P388900	0.000958	0.0124	28.561	4.836
CALR3	Homo sapiens calreticulin 3 (CALR3), mRNA [NM_145046]	A_23_P27538	0.000956	0.0124	4.036	2.013
UNC5CL	Homo sapiens unc-5 homolog C (C. elegans)-like (UNC5CL), mRNA [NM_173561]	A_23_P428298	0.000961	0.0125	3.847	1.944
CRADD	Homo sapiens CASP2 and RIPK1 domain containing adaptor with death domain (CRADD), mRNA [NM_003805]	A_32_P29806	0.000967	0.0125	2.405	1.266
C16orf52	Homo sapiens chromosome 16 open reading frame 52, mRNA (cDNA clone IMAGE:4838212). [BC027604]	A_23_P339053	0.000961	0.0125	2.567	1.36
BEX2	Homo sapiens brain expressed X-linked 2 (BEX2), transcript variant 1, mRNA [NM_001168399]	A_33_P3245178	0.000967	0.0125	2.042	1.03
FLJ39534	Homo sapiens cDNA FLJ33945 fis, clone CTONG2018279. [AK091264]	A_33_P3649236	0.000965	0.0125	2.387	1.255
DRAM	Homo sapiens DNA-damage regulated autophagy modulator 1 (DRAM1), mRNA [NM_018370]	A_23_P99163	0.000969	0.0125	3.009	1.589
PERP	Homo sapiens PERP, TP53 apoptosis effector (PERP), mRNA [NM_022121]	A_23_P214950	0.000983	0.0126	12.666	3.663
EIF4E	Homo sapiens eukaryotic translation initiation factor 4E (EIF4E), transcript variant 1, mRNA [NM_001968]	A_23_P118643	0.000975	0.0126	2.131	1.091
SGPL1	Homo sapiens sphingosine-1-phosphate lyase 1 (SGPL1), mRNA [NM_003901]	A_33_P3327847	0.000981	0.0126	2.259	1.176
KBTBD3	Homo sapiens kelch repeat and BTB (POZ) domain containing 3 (KBTBD3), transcript variant 2, mRNA [NM_198439]	A_23_P127557	0.00099	0.0127	2.805	1.488
OTP	Homo sapiens orthopedia homeobox (OTP), mRNA [NM_032109]	A_23_P84230	0.001	0.0127	133.11	7.056
ENST00000272035	Homo sapiens tubulin, beta 8 class VIII (TUBB8), transcript variant 1, mRNA [NM_177987]	A_32_P16315	0.001	0.0127	3.677	1.878
LOC81691	Homo sapiens exonuclease NEF-sp (LOC81691), transcript variant 1, mRNA [NM_030941]	A_23_P49459	0.00101	0.0128	3.933	1.976
LOC100132541	Q96BH4_HUMAN (Q96BH4) GLT8D3 protein (Fragment), partial (91%) [THC2772510]	A_33_P3283848	0.00101	0.0128	3.448	1.786
MYBPH	Homo sapiens myosin binding protein H (MYBPH), mRNA [NM_004997]	A_23_P148737	0.00102	0.0129	13.32	3.736
CARD6	Homo sapiens caspase recruitment domain family, member 6 (CARD6), mRNA [NM_032587]	A_23_P41854	0.00102	0.0129	2.417	1.273
KLF2	Homo sapiens Kruppel-like factor 2 (lung) (KLF2), mRNA [NM_016270]	A_23_P119196	0.00103	0.013	4.261	2.091
PGD	Homo sapiens phosphogluconate dehydrogenase (PGD), mRNA [NM_002631]	A_23_P126623	0.00103	0.013	2.611	1.385
LAMP2	Homo sapiens lysosomal-associated membrane protein 2 (LAMP2), transcript variant B, mRNA [NM_013995]	A_23_P416608	0.00104	0.0131	2.611	1.385
SIRPB2	Homo sapiens signal-regulatory protein beta 2 (SIRPB2), transcript variant 1, mRNA [NM_001122962]	A_24_P202840	0.00104	0.0131	8.547	3.095
C14orf101	Homo sapiens chromosome 14 open reading frame 101 (C14orf101), mRNA [NM_017799]	A_33_P3245389	0.00104	0.0131	2.214	1.146
LTB4R	Homo sapiens leukotriene B4 receptor (LTB4R), transcript variant 1, mRNA [NM_181657]	A_23_P151791	0.00104	0.0131	2.286	1.193
APAF1	Homo sapiens apoptotic peptidase activating factor 1 (APAF1), transcript variant 3, mRNA [NM_181861]	A_23_P36611	0.00104	0.0131	2.233	1.159
FBXL3	Homo sapiens F-box and leucine-rich repeat protein 3 (FBXL3), mRNA [NM_012158]	A_23_P140069	0.00107	0.0133	2.085	1.06
EMB	Homo sapiens embigin (EMB), mRNA [NM_198449]	A_33_P3359900	0.00107	0.0133	9.341	3.224
LUC7L3	LUC7-like 3 (S. cerevisiae) [Source:HGNC Symbol;Acc:24309] [ENST00000311571]	A_33_P3246997	0.00107	0.0133	2.699	1.432
PANK1	Homo sapiens pantothenate kinase 1 (PANK1), transcript variant alpha, mRNA [NM_148977]	A_33_P3227788	0.00107	0.0133	2.223	1.152
AP1S2	Homo sapiens adaptor-related protein complex 1, sigma 2 subunit (AP1S2), mRNA [NM_003916]	A_23_P217384	0.00107	0.0133	3.876	1.955
FLJ20674	Homo sapiens V-set and immunoglobulin domain containing 10 (VSIG10), mRNA [NM_019086]	A_23_P72059	0.00106	0.0133	4.621	2.208
MFI2	Homo sapiens antigen p97 (melanoma associated) identified by monoclonal antibodies 133.2 and 96.5 (MFI2), transcript variant 1, mRNA [NM_005929]	A_23_P212042	0.00109	0.0134	2.548	1.349
RGS5	Homo sapiens regulator of G-protein signaling 5 (RGS5), transcript variant 1, mRNA [NM_003617]	A_33_P3243093	0.00108	0.0134	3.003	1.586
LITAF	Homo sapiens lipopolysaccharide-induced TNF factor (LITAF), transcript variant 1, mRNA [NM_004862]	A_23_P3532	0.00108	0.0134	4.745	2.246

TGFBR3	Homo sapiens transforming growth factor, beta receptor III (TGFBR3), transcript variant 1, mRNA [NM_003243]	A_23_P200780	0.00109	0.0134	2.159	1.11
AMN1	Homo sapiens antagonist of mitotic exit network 1 homolog (S. cerevisiae) (AMN1), transcript variant 1, mRNA [NM_001113402]	A_24_P100830	0.00109	0.0134	3.27	1.709
COL28A1	Homo sapiens collagen, type XXVIII, alpha 1 (COL28A1), mRNA [NM_001037763]	A_33_P3243787	0.0011	0.0135	5.611	2.488
RIBC1	Homo sapiens RIB43A domain with coiled-coils 1 (RIBC1), transcript variant 1, mRNA [NM_001031745]	A_23_P73667	0.00111	0.0136	1.52	0.604
C21orf66	Homo sapiens GC-rich sequence DNA-binding factor 1 (GCFC1), transcript variant 2, mRNA [NM_013329]	A_23_P211064	0.00112	0.0136	3.382	1.758
FAM190B	Homo sapiens family with sequence similarity 190, member B (FAM190B), mRNA [NM_018999]	A_33_P3214314	0.00111	0.0136	2.035	1.025
TTN	titin [Source:HGNC Symbol;Acc:12403] [ENST00000392423]	A_33_P3329433	0.00111	0.0136	3.546	1.826
ARMC10	Homo sapiens armadillo repeat containing 10 (ARMC10), transcript variant A, mRNA [NM_031905]	A_33_P3213432	0.00111	0.0136	2.164	1.113
LOC100134409	PREDICTED: Homo sapiens double homeobox protein 4-like (LOC100134409), mRNA [XM_001718943]	A_33_P3356696	0.00111	0.0136	3.604	1.85
KIDINS220	Homo sapiens kinase D-interacting substrate, 220kDa (KIDINS220), mRNA [NM_020738]	A_24_P97145	0.00112	0.0136	2.503	1.323
USP3	Homo sapiens ubiquitin specific peptidase 3 (USP3), mRNA [NM_006537]	A_33_P3231110	0.00113	0.0137	1.822	0.866
JMJD7-PLA2G4B	Homo sapiens JMJD7-PLA2G4B readthrough (JMJD7-PLA2G4B), transcript variant 1, mRNA [NM_005090]	A_23_P403424	0.00113	0.0137	2.421	1.276
CTSO	Homo sapiens cathepsin O (CTSO), mRNA [NM_001334]	A_33_P3418025	0.00114	0.0138	3.34	1.74
STOX1	Homo sapiens storkhead box 1 (STOX1), transcript variant 1, mRNA [NM_152709]	A_23_P344481	0.00115	0.0139	3.085	1.625
HORMAD1	Homo sapiens HORMA domain containing 1 (HORMAD1), transcript variant 1, mRNA [NM_032132]	A_32_P199884	0.00115	0.0139	4.041	2.015
HDC	Homo sapiens histidine decarboxylase (HDC), mRNA [NM_002112]	A_23_P117662	0.00116	0.0139	4.719	2.238
ENST00000310109	Homo sapiens lipoyl(octanoyl) transferase 2 (putative) (LIPT2), nuclear gene encoding mitochondrial protein, mRNA [NM_001144869]	A_23_P24922	0.00115	0.0139	2.222	1.152
GREB1	Homo sapiens GREB1 protein, mRNA (cDNA clone IMAGE:6729261), partial cds. [BC071853]	A_33_P3379406	0.00116	0.0139	3.3	1.722
LOC339988	Homo sapiens cDNA clone IMAGE:5217034. [BC041468]	A_33_P3429242	0.00116	0.0139	4.39	2.134
LOC728463	Unknown	A_33_P3365988	0.00115	0.0139	5.232	2.387
AREG	Homo sapiens amphiregulin (AREG), mRNA [NM_001657]	A_23_P259071	0.00116	0.0139	8.64	3.111
ANKRD10	Homo sapiens ankyrin repeat domain 10 (ANKRD10), mRNA [NM_017664]	A_23_P205046	0.00115	0.0139	2.229	1.156
OTOA	Homo sapiens otoancorin (OTOA), transcript variant 2, mRNA [NM_170664]	A_32_P52519	0.00117	0.014	3.268	1.708
RAVER2	Homo sapiens ribonucleoprotein, PTB-binding 2 (RAVER2), mRNA [NM_018211]	A_23_P328323	0.00117	0.014	2.03	1.022
NECAP1	Homo sapiens NECAP endocytosis associated 1 (NECAP1), transcript variant 1, mRNA [NM_015509]	A_33_P3247629	0.00117	0.014	2.393	1.259
ENST00000244070	protein phosphatase 4, regulatory subunit 1-like [Source:HGNC Symbol;Acc:15755] [ENST00000244070]	A_33_P3280597	0.00117	0.014	3.202	1.679
POLB	Homo sapiens polymerase (DNA directed), beta (POLB), mRNA [NM_002690]	A_32_P34552	0.00118	0.0141	2.077	1.055
TG	Homo sapiens thyroglobulin (TG), mRNA [NM_003235]	A_23_P32454	0.00118	0.0141	3.985	1.995
TPM4	Homo sapiens tropomyosin 4 (TPM4), transcript variant 2, mRNA [NM_003290]	A_23_P141974	0.00118	0.0141	3.783	1.919
LOC150786	Homo sapiens RAB6C-like (WTH3DI), mRNA [NM_001077637]	A_33_P3323904	0.00118	0.0141	5.303	2.407
TNFAIP3	Homo sapiens tumor necrosis factor, alpha-induced protein 3 (TNFAIP3), mRNA [NM_006290]	A_24_P157926	0.00118	0.0141	5.824	2.542
SYNPO	Homo sapiens synaptopodin (SYNPO), transcript variant 1, mRNA [NM_007286]	A_23_P344531	0.0012	0.0142	4.839	2.275
VAV3	Homo sapiens vav 3 guanine nucleotide exchange factor (VAV3), transcript variant 1, mRNA [NM_006113]	A_23_P201551	0.00119	0.0142	10.732	3.424
MFSD9	Homo sapiens major facilitator superfamily domain containing 9 (MFSD9), mRNA [NM_032718]	A_33_P3272189	0.0012	0.0142	2.187	1.129
LOC645225	Unknown	A_33_P3417305	0.0012	0.0142	3.696	1.886
ENST00000401931	interleukin 8 [Source:HGNC Symbol;Acc:6025] [ENST00000401931]	A_33_P3243230	0.00119	0.0142	13.309	3.734
ADRB2	Homo sapiens adrenergic, beta-2-, receptor, surface (ADRB2), mRNA [NM_000024]	A_23_P145024	0.0012	0.0142	81.095	6.342
NRCAM	Homo sapiens neuronal cell adhesion molecule (NRCAM), transcript variant 1, mRNA [NM_001037132]	A_24_P252364	0.0012	0.0142	67.562	6.078
C7orf34	Homo sapiens chromosome 7 open reading frame 34 (C7orf34), mRNA [NM_178829]	A_23_P157316	0.00121	0.0143	3.434	1.78
A_33_P3304748	Unknown	A_33_P3304748	0.00121	0.0143	2.585	1.37
MAEA	Homo sapiens macrophage erythroblast attacher (MAEA), transcript variant 1, mRNA [NM_001017405]	A_23_P18490	0.00121	0.0143	2.262	1.178
LOC100133089	Homo sapiens cDNA FLJ45867 fis, clone OCBBF3003745. [AK127766]	A_33_P3356371	0.00123	0.0144	2.355	1.236

SNAPC1	Homo sapiens small nuclear RNA activating complex, polypeptide 1, 43kDa (SNAPC1), mRNA [NM_003082]	A_33_P3316223	0.00123	0.0144	2.403	1.265
ALDH2	Homo sapiens aldehyde dehydrogenase 2 family (mitochondrial) (ALDH2), nuclear gene encoding mitochondrial protein, transcript variant 1, mRNA [NM_000690]	A_23_P36753	0.00122	0.0144	5.294	2.404
SLC30A7	Homo sapiens solute carrier family 30 (zinc transporter), member 7 (SLC30A7), transcript variant 1, mRNA [NM_133496]	A_24_P563545	0.00122	0.0144	2.09	1.063
ANKRD35	Homo sapiens ankyrin repeat domain 35 (ANKRD35), mRNA [NM_144698]	A_23_P325690	0.00122	0.0144	4.061	2.022
CASP6	Homo sapiens caspase 6, apoptosis-related cysteine peptidase (CASP6), transcript variant alpha, mRNA [NM_001226]	A_23_P500799	0.00122	0.0144	2.573	1.363
ATG3	Homo sapiens ATG3 autophagy related 3 homolog (S. cerevisiae) (ATG3), mRNA [NM_022488]	A_23_P212706	0.00124	0.0145	2.556	1.354
CORIN	Homo sapiens corin, serine peptidase (CORIN), mRNA [NM_006587]	A_23_P81131	0.00124	0.0145	6.974	2.802
DSP	Homo sapiens desmoplakin (DSP), transcript variant 1, mRNA [NM_004415]	A_33_P3402565	0.00124	0.0145	4.68	2.226
USP49	Homo sapiens ubiquitin specific peptidase 49 (USP49), mRNA [NM_018561]	A_23_P331770	0.00124	0.0145	3.007	1.588
CAV2	Homo sapiens caveolin 2 (CAV2), transcript variant 1, mRNA [NM_001233]	A_24_P925040	0.00124	0.0145	4.932	2.302
LTA4H	Homo sapiens leukotriene A4 hydrolase (LTA4H), mRNA [NM_000895]	A_23_P388670	0.00125	0.0146	2.07	1.049
NRAP	Homo sapiens nebulin-related anchoring protein (NRAP), transcript variant 2, mRNA [NM_198060]	A_23_P402765	0.00125	0.0146	23.446	4.551
GPR115	Homo sapiens G protein-coupled receptor 115 (GPR115), mRNA [NM_153838]	A_23_P7866	0.00125	0.0146	5.876	2.555
CRYBA1	Homo sapiens crystallin, beta A1 (CRYBA1), mRNA [NM_005208]	A_23_P4254	0.00127	0.0147	5.773	2.529
ITGB5	Homo sapiens integrin, beta 5 (ITGB5), mRNA [NM_002213]	A_23_P166633	0.00126	0.0147	3.453	1.788
HBEGF	Homo sapiens heparin-binding EGF-like growth factor (HBEGF), mRNA [NM_001945]	A_24_P140608	0.00127	0.0147	4.203	2.071
SCUBE2	Homo sapiens signal peptide, CUB domain, EGF-like 2 (SCUBE2), transcript variant 1, mRNA [NM_020974]	A_23_P105144	0.00128	0.0148	5.865	2.552
LONRF3	Homo sapiens LON peptidase N-terminal domain and ring finger 3 (LONRF3), transcript variant 1, mRNA [NM_001031855]	A_23_P114414	0.00128	0.0148	4.343	2.119
TPD52L1	Homo sapiens tumor protein D52-like 1 (TPD52L1), transcript variant 4, mRNA [NM_001003397]	A_33_P3398065	0.00128	0.0148	4.555	2.187
CLEC2B	Homo sapiens C-type lectin domain family 2, member B (CLEC2B), mRNA [NM_005127]	A_33_P3332970	0.00128	0.0148	5.319	2.411
SLC9A1	Homo sapiens solute carrier family 9 (sodium/hydrogen exchanger), member 1 (SLC9A1), mRNA [NM_003047]	A_24_P71373	0.0013	0.015	3.224	1.689
POR	Homo sapiens P450 (cytochrome) oxidoreductase (POR), mRNA [NM_000941]	A_24_P29723	0.0013	0.015	3.049	1.609
ASPA	Homo sapiens aspartoacylase (ASPA), transcript variant 1, mRNA [NM_000049]	A_23_P164436	0.00132	0.0151	6.499	2.7
GALM	Homo sapiens galactose mutarotase (aldose 1-epimerase) (GALM), mRNA [NM_138801]	A_24_P212539	0.00132	0.0151	2.217	1.149
YJEFN3	Homo sapiens YjeF N-terminal domain containing 3 (YJEFN3), nuclear gene encoding mitochondrial protein, transcript variant 1, mRNA [NM_198537]	A_33_P3382944	0.00132	0.0151	3.292	1.719
CCT8L2	Homo sapiens chaperonin containing TCP1, subunit 8 (theta)-like 2 (CCT8L2), mRNA [NM_014406]	A_23_P29110	0.00133	0.0152	6.309	2.657
ZC3H12C	Homo sapiens zinc finger CCCH-type containing 12C (ZC3H12C), mRNA [NM_033390]	A_23_P388993	0.00133	0.0152	4.125	2.045
HSD17B12	Homo sapiens hydroxysteroid (17-beta) dehydrogenase 12 (HSD17B12), mRNA [NM_016142]	A_23_P47377	0.00133	0.0152	2.018	1.013
TRPC3	Homo sapiens transient receptor potential cation channel, subfamily C, member 3 (TRPC3), transcript variant 2, mRNA [NM_003305]	A_23_P41455	0.00134	0.0152	2.625	1.392
ZYX	Homo sapiens zyxin (ZYX), transcript variant 1, mRNA [NM_003461]	A_23_P254888	0.00135	0.0153	2.68	1.422
MPZL1	Homo sapiens myelin protein zero-like 1 (MPZL1), transcript variant 1, mRNA [NM_003953]	A_23_P476	0.00135	0.0153	2.048	1.034
BHLHE41	Homo sapiens basic helix-loop-helix family, member e41 (BHLHE41), mRNA [NM_030762]	A_23_P139500	0.00136	0.0154	22.117	4.467
JAZF1	Homo sapiens JAZF zinc finger 1 (JAZF1), mRNA [NM_175061]	A_32_P36694	0.00136	0.0154	4.399	2.137
HFE	Homo sapiens hemochromatosis (HFE), transcript variant 1, mRNA [NM_000410]	A_24_P111996	0.00136	0.0154	4.942	2.305
FAM8A1	Homo sapiens family with sequence similarity 8, member A1 (FAM8A1), mRNA [NM_016255]	A_23_P133648	0.00137	0.0154	4.665	2.222
UCN2	Homo sapiens urocortin 2 (UCN2), mRNA [NM_033199]	A_33_P3279629	0.00137	0.0154	6.513	2.703
ERMN	Homo sapiens ermin, ERM-like protein (ERMN), transcript variant 2, mRNA [NM_020711]	A_33_P3250165	0.00137	0.0154	4.223	2.078
AZI2	Homo sapiens 5-azacytidine induced 2 (AZI2), transcript variant 1, mRNA [NM_022461]	A_33_P3322283	0.00136	0.0154	3.16	1.66

KLF14	Homo sapiens Kruppel-like factor 14 (KLF14), mRNA [NM_138693]	A_33_P3374947	0.00137	0.0154	3.648	1.867
NKG7	Homo sapiens natural killer cell group 7 sequence (NKG7), mRNA [NM_005601]	A_23_P119042	0.00137	0.0154	6.337	2.664
NEK11	Homo sapiens NIMA (never in mitosis gene a)- related kinase 11 (NEK11), transcript variant 2, mRNA [NM_145910]	A_23_P155301	0.00136	0.0154	3.719	1.895
KIAA0430	Homo sapiens KIAA0430 (KIAA0430), transcript variant 1, mRNA [NM_014647]	A_23_P26674	0.00138	0.0155	2.077	1.055
FAM21C	Homo sapiens family with sequence similarity 21, member C (FAM21C), transcript variant 1, mRNA [NM_015262]	A_23_P376799	0.00139	0.0156	2.306	1.206
ZDHHC23	Homo sapiens zinc finger, DHHC-type containing 23 (ZDHHC23), mRNA [NM_173570]	A_23_P350689	0.00139	0.0156	3.333	1.737
FRMD6	Homo sapiens FERM domain containing 6 (FRMD6), transcript variant 1, mRNA [NM_001042481]	A_24_P330303	0.00141	0.0157	8.927	3.158
COMMD6	Homo sapiens COMM domain containing 6 (COMMD6), transcript variant 1, mRNA [NM_203497]	A_32_P114215	0.0014	0.0157	2.113	1.079
MBD3L2	Homo sapiens methyl-CpG binding domain protein 3-like 2 (MBD3L2), mRNA [NM_144614]	A_23_P378450	0.00141	0.0157	4.381	2.131
LOC647195	Unknown	A_24_P110273	0.0014	0.0157	2.478	1.309
SIK2	Homo sapiens salt-inducible kinase 2 (SIK2), mRNA [NM_015191]	A_33_P3216933	0.0014	0.0157	2.519	1.333
TIMP2	Homo sapiens TIMP metallopeptidase inhibitor 2 (TIMP2), mRNA [NM_003255]	A_33_P3382177	0.00141	0.0157	6.302	2.656
NT5C2	Homo sapiens 5'-nucleotidase, cytosolic II (NT5C2), transcript variant 1, mRNA [NM_012229]	A_23_P97906	0.00143	0.0158	2.274	1.185
PECR	Homo sapiens peroxisomal trans-2-enoyl-CoA reductase (PECR), mRNA [NM_018441]	A_23_P91140	0.00143	0.0158	3.331	1.736
SLC28A2	Homo sapiens solute carrier family 28 (sodium-coupled nucleoside transporter), member 2 (SLC28A2), mRNA [NM_004212]	A_23_P48816	0.00142	0.0158	7.772	2.958
MANBA	Homo sapiens mannosidase, beta A, lysosomal (MANBA), mRNA [NM_005908]	A_23_P258698	0.00143	0.0158	2.789	1.48
SLC16A3	Homo sapiens solute carrier family 16, member 3 (monocarboxylic acid transporter 4) (SLC16A3), transcript variant 2, mRNA [NM_001042422]	A_23_P158725	0.00143	0.0158	3.325	1.733
CYB5D1	Homo sapiens cytochrome b5 domain containing 1 (CYB5D1), mRNA [NM_144607]	A_33_P3387045	0.00143	0.0158	2.174	1.121
ST5	Homo sapiens suppression of tumorigenicity 5 (ST5), transcript variant 1, mRNA [NM_005418]	A_23_P24884	0.00142	0.0158	4.965	2.312
CX3CR1	Homo sapiens chemokine (C-X3-C motif) receptor 1 (CX3CR1), transcript variant 4, mRNA [NM_001337]	A_23_P407565	0.00143	0.0158	4.154	2.054
GPR56	Homo sapiens G protein-coupled receptor 56 (GPR56), transcript variant 3, mRNA [NM_201525]	A_23_P206280	0.00143	0.0158	2.096	1.068
ADH5	Homo sapiens alcohol dehydrogenase 5 (class III), chi polypeptide (ADH5), mRNA [NM_000671]	A_23_P18692	0.00144	0.0159	2.566	1.359
TNFRSF17	Homo sapiens tumor necrosis factor receptor superfamily, member 17 (TNFRSF17), mRNA [NM_001192]	A_23_P37736	0.00144	0.0159	4.252	2.088
MAP2K5	Homo sapiens mitogen-activated protein kinase kinase 5 (MAP2K5), transcript variant 2, mRNA [NM_002757]	A_24_P356130	0.00144	0.0159	2.198	1.136
ZSCAN5B	Homo sapiens zinc finger and SCAN domain containing 5B (ZSCAN5B), mRNA [NM_001080456]	A_33_P3406651	0.00146	0.016	8.346	3.061
TJP3	Homo sapiens tight junction protein 3 (zona occludens 3) (TJP3), mRNA [NM_014428]	A_23_P108157	0.00145	0.016	3.205	1.681
HIF3A	Homo sapiens hypoxia inducible factor 3, alpha subunit (HIF3A), transcript variant 2, mRNA [NM_022462]	A_23_P338534	0.00147	0.0161	8.441	3.077
RNF122	Homo sapiens ring finger protein 122 (RNF122), mRNA [NM_024787]	A_23_P134744	0.00147	0.0161	2.393	1.259
GBP1	Homo sapiens guanylate binding protein 1, interferon-inducible (GBP1), mRNA [NM_002053]	A_23_P62890	0.00147	0.0161	18.365	4.199
MYO5A	Homo sapiens myosin VA (heavy chain 12, myoxin) (MYO5A), transcript variant 1, mRNA [NM_000259]	A_24_P255218	0.00148	0.0162	3.455	1.788
CLEC1B	Homo sapiens C-type lectin domain family 1, member B (CLEC1B), transcript variant 1, mRNA [NM_016509]	A_33_P3236065	0.00148	0.0162	4.206	2.073
RNF135	Homo sapiens ring finger protein 135 (RNF135), transcript variant 1, mRNA [NM_032322]	A_23_P252283	0.0015	0.0163	9.075	3.182
IL1R1	Homo sapiens interleukin 1 receptor, type I (IL1R1), mRNA [NM_000877]	A_33_P3396389	0.0015	0.0163	3.574	1.838
IGSF6	Homo sapiens immunoglobulin superfamily, member 6 (IGSF6), mRNA [NM_005849]	A_33_P3372004	0.0015	0.0163	5.186	2.375
FOXJ2	Homo sapiens forkhead box J2 (FOXJ2), mRNA [NM_018416]	A_24_P21985	0.00151	0.0163	2.778	1.474
LRRC50	Homo sapiens dynein, axonemal, assembly factor 1 (DNAAF1), mRNA [NM_178452]	A_23_P54612	0.00151	0.0163	2.802	1.487
SIAH1	Homo sapiens seven in absentia homolog 1 (Drosophila) (SIAH1), transcript variant 1, mRNA [NM_003031]	A_33_P3270509	0.00152	0.0164	2.08	1.057
TRD@	T cell receptor delta constant [Source:HGNC Symbol;Acc:12253] [ENST00000390477]	A_33_P3246838	0.00152	0.0164	21.383	4.418
NFE4	Homo sapiens transcription factor NF-E4 (NFE4), mRNA [NM_001085386]	A_33_P3360814	0.00152	0.0164	6.963	2.8
FYB	Homo sapiens FYN binding protein (FYB), transcript variant 1, mRNA [NM_001465]	A_24_P393740	0.00152	0.0164	9.492	3.247

GRAMD3	Homo sapiens GRAM domain containing 3 (GRAMD3), transcript variant 2, mRNA [NM_023927]	A_23_P22350	0.00153	0.0165	7.308	2.869
DYDC2	Homo sapiens DPY30 domain containing 2 (DYDC2), mRNA [NM_032372]	A_23_P75063	0.00154	0.0165	4.572	2.193
NPC1	Homo sapiens Niemann-Pick disease, type C1 (NPC1), mRNA [NM_000271]	A_23_P107587	0.00153	0.0165	4.349	2.121
PYGL	Homo sapiens phosphorylase, glycogen, liver (PYGL), transcript variant 1, mRNA [NM_002863]	A_23_P48676	0.00154	0.0165	4.774	2.255
LOC100506859	PREDICTED: Homo sapiens serine/threonine-protein kinase Nek5-like (LOC100506859), mRNA [XM_003118889]	A_21_P0013974	0.00153	0.0165	3.497	1.806
DIRAS3	Homo sapiens DIRAS family, GTP-binding RAS-like 3 (DIRAS3), mRNA [NM_004675]	A_23_P149121	0.00154	0.0165	4.928	2.301
FMR1NB	Homo sapiens fragile X mental retardation 1 neighbor (FMR1NB), mRNA [NM_152578]	A_32_P99019	0.00157	0.0168	3.726	1.898
CLEC4M	Homo sapiens C-type lectin domain family 4, member M (CLEC4M), transcript variant 2, mRNA [NM_001144904]	A_23_P208482	0.00157	0.0168	6.4	2.678
THC2526807	Unknown	A_33_P3281651	0.00158	0.0168	5.315	2.41
A_19_P00324604	Homo sapiens chromosome 17 open reading frame 51 (C17orf51), mRNA [NM_001113434]	A_19_P00324604	0.00158	0.0168	2.901	1.537
NCOA7	Homo sapiens nuclear receptor coactivator 7 (NCOA7), transcript variant 1, mRNA [NM_181782]	A_24_P12435	0.00158	0.0168	2.403	1.265
MED30	Homo sapiens mediator complex subunit 30 (MED30), mRNA [NM_080651]	A_23_P31866	0.00158	0.0168	2.711	1.439
ALDH7A1	Homo sapiens aldehyde dehydrogenase 7 family, member A1 (ALDH7A1), nuclear gene encoding mitochondrial protein, transcript variant 1, mRNA [NM_001182]	A_23_P70231	0.00159	0.0169	4.933	2.302
SCARB2	Homo sapiens scavenger receptor class B, member 2 (SCARB2), transcript variant 2, mRNA [NM_001204255]	A_33_P3410459	0.00159	0.0169	2.682	1.424
EVI2B	Homo sapiens ecotropic viral integration site 2B (EVI2B), mRNA [NM_006495]	A_23_P66694	0.0016	0.0169	11.565	3.532
LRRC28	Homo sapiens leucine rich repeat containing 28 (LRRC28), mRNA [NM_144598]	A_33_P3301034	0.00161	0.017	3.38	1.757
PRPH	Homo sapiens peripherin (PRPH), mRNA [NM_006262]	A_23_P13713	0.00162	0.0171	3.375	1.755
TRIM34	Homo sapiens tripartite motif containing 34 (TRIM34), transcript variant 4, mRNA [NM_001003827]	A_24_P398323	0.00164	0.0172	2.311	1.209
SQSTM1	Homo sapiens sequestosome 1 (SQSTM1), transcript variant 1, mRNA [NM_003900]	A_23_P81399	0.00164	0.0172	2.119	1.083
DPPA2	Homo sapiens developmental pluripotency associated 2 (DPPA2), mRNA [NM_138815]	A_23_P405885	0.00166	0.0173	3.548	1.827
LOC100132815	HCG2000535Uncharacterized proteincDNA FLJ32177 fis, clone PLACE6001294 [Source:UniProtKB/TrEMBL;Acc:Q96ML8] [ENST00000397094]	A_32_P85042	0.00165	0.0173	2.325	1.218
AX747850	Homo sapiens cDNA FLJ35685 fis, clone SPLEN2019257. [AK093004]	A_33_P3735158	0.00166	0.0173	2.035	1.025
A_19_P00804596	Homo sapiens chromosome 17 open reading frame 51 (C17orf51), mRNA [NM_001113434]	A_19_P00804596	0.00165	0.0173	2.309	1.208
A_19_P00808120	Homo sapiens family with sequence similarity 200, member B (FAM200B), mRNA [NM_001145191]	A_19_P00808120	0.00166	0.0173	3.592	1.845
MGC29506	Homo sapiens marginal zone B and B1 cell-specific protein (MZB1), mRNA [NM_016459]	A_23_P84596	0.00165	0.0173	2.135	1.094
C1QTNF3	Homo sapiens C1q and tumor necrosis factor related protein 3 (C1QTNF3), transcript variant 2, mRNA [NM_181435]	A_23_P122068	0.00167	0.0174	3.843	1.942
CSF2RA	Homo sapiens colony stimulating factor 2 receptor, alpha, low-affinity (granulocyte-macrophage) (CSF2RA), transcript variant 6, mRNA [NM_172249]	A_23_P501985	0.00168	0.0175	8.885	3.151
TMEM65	Homo sapiens transmembrane protein 65 (TMEM65), mRNA [NM_194291]	A_33_P3494748	0.00168	0.0175	3.939	1.978
LEPREL1	Homo sapiens leprecan-like 1 (LEPREL1), transcript variant 1, mRNA [NM_018192]	A_23_P69179	0.0017	0.0176	6.06	2.599
SUNC1	Homo sapiens Sad1 and UNC84 domain containing 3 (SUN3), transcript variant 1, mRNA [NM_001030019]	A_23_P329962	0.00169	0.0176	4.555	2.187
RNF6	Homo sapiens ring finger protein (C3H2C3 type) 6 (RNF6), transcript variant 1, mRNA [NM_005977]	A_23_P162734	0.00169	0.0176	2.309	1.207
CDC14B	Homo sapiens CDC14 cell division cycle 14 homolog B (S. cerevisiae) (CDC14B), transcript variant 2, mRNA [NM_033331]	A_23_P216679	0.00169	0.0176	3.17	1.665
STAU2	Homo sapiens staufen, RNA binding protein, homolog 2 (Drosophila) (STAU2), transcript variant 1, mRNA [NM_001164380]	A_24_P374634	0.00171	0.0177	2.692	1.429
AK021933	Homo sapiens cDNA FLJ11871 fis, clone HEMBA1007052. [AK021933]	A_24_P84781	0.00173	0.0178	2.884	1.528
PIGY	Homo sapiens phosphatidylinositol glycan anchor biosynthesis, class Y (PIGY), nuclear gene encoding mitochondrial protein, transcript variant 2, mRNA [NM_001042616]	A_24_P140391	0.00173	0.0178	2.003	1.002
HK2	Homo sapiens hexokinase 2 (HK2), mRNA [NM_000189]	A_32_P175739	0.00172	0.0178	5.549	2.472

ENST00000373551	golgin A1 [Source:HGNC Symbol;Acc:4424] [ENST00000373551]	A_33_P3271460	0.00174	0.0178	2.922	1.547
A_19_P00316405	Homo sapiens chromosome 17 open reading frame 51 (C17orf51), mRNA [NM_001113434]	A_19_P00316405	0.00174	0.0178	2.221	1.151
FAM200B	Homo sapiens family with sequence similarity 200, member B (FAM200B), mRNA [NM_001145191]	A_33_P3411145	0.00172	0.0178	3.351	1.745
IL10RB	Homo sapiens interleukin 10 receptor, beta (IL10RB), mRNA [NM_000628]	A_24_P322741	0.00172	0.0178	2.339	1.226
ARHGEF6	Homo sapiens Rac/Cdc42 guanine nucleotide exchange factor (GEF) 6 (ARHGEF6), mRNA [NM_004840]	A_24_P228875	0.00173	0.0178	4.634	2.212
TMEM116	BROAD Institute lincRNA (XLOC_010203), lincRNA [TCONS_00021409]	A_21_P0007892	0.00175	0.0179	1.238	0.308
GSG1	Homo sapiens germ cell associated 1 (GSG1), transcript variant 1, mRNA [NM_031289]	A_23_P139864	0.00178	0.0181	3.492	1.804
TBCC	Homo sapiens tubulin folding cofactor C (TBCC), mRNA [NM_003192]	A_24_P397584	0.00177	0.0181	2.369	1.245
KLHL30	Homo sapiens kelch-like 30 (Drosophila) (KLHL30), mRNA [NM_198582]	A_33_P3243702	0.00177	0.0181	4.622	2.208
LTF	Homo sapiens lactotransferrin (LTF), transcript variant 1, mRNA [NM_002343]	A_23_P166848	0.00179	0.0182	30.307	4.922
TNFAIP8	Homo sapiens tumor necrosis factor, alpha-induced protein 8 (TNFAIP8), transcript variant 1, mRNA [NM_014350]	A_32_P219520	0.00181	0.0183	3.21	1.683
KIAA1370	Homo sapiens KIAA1370 (KIAA1370), mRNA [NM_019600]	A_23_P99853	0.0018	0.0183	3.753	1.908
MTHFS	Homo sapiens 5,10-methenyltetrahydrofolate synthetase (5-formyltetrahydrofolate cyclo-ligase) (MTHFS), transcript variant 1, mRNA [NM_006441]	A_23_P163380	0.0018	0.0183	3.04	1.604
ANK3	Homo sapiens ankyrin 3, node of Ranvier (ankyrin G) (ANK3), transcript variant 1, mRNA [NM_020987]	A_23_P202269	0.00181	0.0183	2.764	1.467
CFHR2	Homo sapiens complement factor H-related 2 (CFHR2), mRNA [NM_005666]	A_23_P951	0.00182	0.0184	7.919	2.985
ETV5	Homo sapiens ets variant 5 (ETV5), mRNA [NM_004454]	A_32_P30649	0.00182	0.0184	2.229	1.156
TRPM2	Homo sapiens transient receptor potential cation channel, subfamily M, member 2 (TRPM2), transcript variant 1, mRNA [NM_003307]	A_24_P27977	0.00183	0.0184	13.121	3.714
LRP2	Homo sapiens low density lipoprotein receptor-related protein 2 (LRP2), mRNA [NM_004525]	A_33_P3244728	0.00184	0.0185	3.786	1.921
IL17RA	Homo sapiens interleukin 17 receptor A (IL17RA), mRNA [NM_014339]	A_23_P17706	0.00184	0.0185	3.825	1.936
ADCY10	Homo sapiens adenylate cyclase 10 (soluble) (ADCY10), transcript variant 1, mRNA [NM_018417]	A_23_P126313	0.00186	0.0186	6.14	2.618
C6orf204	Homo sapiens chromosome 6 open reading frame 204 (C6orf204), transcript variant 2, mRNA [NM_206921]	A_33_P3407339	0.00185	0.0186	6.04	2.595
THC2691182	BC060845 l(3)mbt-like 3, isoform b {Homo sapiens} (exp=-1; wgp=0; cg=0), partial (8%) [THC2691182]	A_33_P3385686	0.00185	0.0186	2.143	1.1
S1PR3	Homo sapiens sphingosine-1-phosphate receptor 3 (S1PR3), mRNA [NM_005226]	A_33_P3281283	0.00185	0.0186	4.058	2.021
RIMS1	Homo sapiens regulating synaptic membrane exocytosis 1 (RIMS1), transcript variant 1, mRNA [NM_014989]	A_33_P3218832	0.00185	0.0186	4.502	2.17
KBTBD10	Homo sapiens kelch repeat and BTB (POZ) domain containing 10 (KBTBD10), mRNA [NM_006063]	A_23_P17190	0.00187	0.0187	7.667	2.939
A_33_P3310774	Homo sapiens GDCG4p14.2 mRNA, complete cds, alternatively spliced. [JN120858]	A_33_P3310774	0.00187	0.0187	9.918	3.31
EGFL6	Homo sapiens EGF-like-domain, multiple 6 (EGFL6), transcript variant 2, mRNA [NM_001167890]	A_33_P3290709	0.00187	0.0187	5.077	2.344
EGR1	Homo sapiens early growth response 1 (EGR1), mRNA [NM_001964]	A_23_P214080	0.00189	0.0188	8.197	3.035
CALB1	Homo sapiens calbindin 1, 28kDa (CALB1), mRNA [NM_004929]	A_23_P43197	0.00189	0.0188	30.756	4.943
KRT31	Homo sapiens keratin 31 (KRT31), mRNA [NM_002277]	A_23_P107465	0.00188	0.0188	8.959	3.163
SLU7	Homo sapiens SLU7 splicing factor homolog (S. cerevisiae) (SLU7), mRNA [NM_006425]	A_24_P174824	0.0019	0.0189	2.064	1.046
HSH2D	Homo sapiens hematopoietic SH2 domain containing (HSH2D), mRNA [NM_032855]	A_23_P153372	0.00193	0.0191	3.859	1.948
FBXO32	Homo sapiens F-box protein 32 (FBXO32), transcript variant 1, mRNA [NM_058229]	A_33_P3378126	0.00195	0.0192	2.77	1.47
KLF7	Homo sapiens Kruppel-like factor 7 (ubiquitous) (KLF7), mRNA [NM_003709]	A_23_P67980	0.00195	0.0192	3.542	1.824
GOLGA8E	Homo sapiens golgin A8 family, member E (GOLGA8E), non-coding RNA [NR_033350]	A_32_P119569	0.00196	0.0193	4.36	2.124
ROGDI	Homo sapiens rogdi homolog (Drosophila) (ROGDI), mRNA [NM_024589]	A_23_P100499	0.00196	0.0193	2.759	1.464
HBP1	Homo sapiens HMG-box transcription factor 1 (HBP1), transcript variant 2, mRNA [NM_012257]	A_23_P215787	0.00197	0.0193	2.089	1.063
BCAR3	Homo sapiens breast cancer anti-estrogen resistance 3 (BCAR3), mRNA [NM_003567]	A_23_P97394	0.00199	0.0194	6.459	2.691
STRC	Homo sapiens stereocilin (STRC), mRNA [NM_153700]	A_23_P163238	0.00199	0.0194	5.74	2.521
ART3	Homo sapiens ADP-ribosyltransferase 3 (ART3), transcript variant 3, mRNA [NM_001130017]	A_33_P3723448	0.00198	0.0194	6.369	2.671
CR595314	Homo sapiens primary neuroblastoma cDNA, clone:Nbla00204, full insert sequence. [AB073651]	A_33_P3399480	0.00198	0.0194	3.748	1.906

ARHGEF3	Homo sapiens Rho guanine nucleotide exchange factor (GEF) 3 (ARHGEF3), transcript variant 3, mRNA [NM_019555]	A_23_P143885	0.00198	0.0194	2.336	1.224
FLJ41603	Homo sapiens Rho guanine nucleotide exchange factor (GEF) 37 (ARHGEF37), mRNA [NM_001001669]	A_32_P222695	0.002	0.0195	3.001	1.586
FILIP1L	Homo sapiens filamin A interacting protein 1-like (FILIP1L), transcript variant 1, mRNA [NM_182909]	A_23_P252052	0.002	0.0195	2.24	1.164
CECR6	Homo sapiens cat eye syndrome chromosome region, candidate 6 (CECR6), transcript variant 1, mRNA [NM_031890]	A_23_P259344	0.00201	0.0195	4.201	2.071
DT932733	Homo sapiens uncharacterized LOC644100 (LOC644100), mRNA [NM_001195581]	A_33_P3222009	0.002	0.0195	5.487	2.456
CD47	Homo sapiens CD47 molecule (CD47), transcript variant 2, mRNA [NM_198793]	A_23_P6935	0.002	0.0195	2.104	1.073
CCDC103	family with sequence similarity 187, member A [Source:HGNC Symbol;Acc:35153] [ENST00000331733]	A_23_P26928	0.00204	0.0197	2.273	1.184
RFPL4B	Homo sapiens ret finger protein-like 4B (RFPL4B), mRNA [NM_001013734]	A_33_P3397835	0.00203	0.0197	4.554	2.187
C10orf57	Homo sapiens chromosome 10 open reading frame 57 (C10orf57), mRNA [NM_025125]	A_23_P97853	0.00205	0.0198	3.299	1.722
IDS	Homo sapiens iduronate 2-sulfatase (IDS), transcript variant 2, mRNA [NM_006123]	A_23_P217475	0.00205	0.0198	2.041	1.03
AK3	Homo sapiens adenylate kinase 3 (AK3), nuclear gene encoding mitochondrial protein, transcript variant 1, mRNA [NM_016282]	A_33_P3263061	0.00205	0.0198	2.009	1.007
GRK4	Homo sapiens G protein-coupled receptor kinase 4 (GRK4), transcript variant 3, mRNA [NM_001004057]	A_23_P212756	0.00205	0.0198	4.472	2.161
ELL3	Homo sapiens elongation factor RNA polymerase II-like 3 (ELL3), mRNA [NM_025165]	A_24_P47988	0.00208	0.0199	14.137	3.821
TTC26	Homo sapiens tetratricopeptide repeat domain 26 (TTC26), transcript variant 1, mRNA [NM_024926]	A_23_P257668	0.00208	0.0199	2.428	1.28
DNAH10	Homo sapiens dynein, axonemal, heavy chain 10 (DNAH10), mRNA [NM_207437]	A_32_P228268	0.00208	0.0199	6.606	2.724
SEC16A	Homo sapiens SEC16 homolog A (S. cerevisiae) (SEC16A), mRNA [NM_014866]	A_23_P251303	0.00207	0.0199	2.01	1.007
OSBPL6	Homo sapiens oxysterol binding protein-like 6 (OSBPL6), transcript variant 1, mRNA [NM_032523]	A_23_P108823	0.00207	0.0199	2.435	1.284
LOC283767	Homo sapiens golgin A6 family-like 6 (GOLGA6L6), mRNA [NM_001145004]	A_24_P50972	0.00207	0.0199	4.987	2.318
SAMD8	Homo sapiens sterile alpha motif domain containing 8 (SAMD8), transcript variant 2, mRNA [NM_144660]	A_23_P63870	0.00209	0.02	2.187	1.129
ADAMTS9	Homo sapiens ADAM metallopeptidase with thrombospondin type 1 motif, 9 (ADAMTS9), mRNA [NM_182920]	A_32_P196263	0.0021	0.0201	4.897	2.292
LOC100131646	CR992331 RZPD no.9016 Homo sapiens cDNA clone RZPDp9016A0141 5', mRNA sequence [CR992331]	A_32_P150300	0.0021	0.0201	2.565	1.359
FDX1	Homo sapiens ferredoxin 1 (FDX1), nuclear gene encoding mitochondrial protein, mRNA [NM_004109]	A_33_P3343690	0.00211	0.0201	2.976	1.574
CYP3A43	Homo sapiens cytochrome P450, family 3, subfamily A, polypeptide 43 (CYP3A43), transcript variant 1, mRNA [NM_022820]	A_23_P215828	0.0021	0.0201	2.811	1.491
KIF21A	Homo sapiens kinesin family member 21A (KIF21A), transcript variant 2, mRNA [NM_017641]	A_23_P113462	0.00212	0.0202	3.211	1.683
LOC284242	Homo sapiens, clone IMAGE:5745916, mRNA. [BC035844]	A_33_P3683362	0.00213	0.0202	2.674	1.419
ENST00000340561	acyl-CoA oxidase-like [Source:HGNC Symbol;Acc:25621] [ENST00000340561]	A_33_P3274049	0.00215	0.0203	4.305	2.106
GSTA4	Homo sapiens glutathione S-transferase alpha 4 (GSTA4), mRNA [NM_001512]	A_23_P110941	0.00214	0.0203	3.204	1.68
CLEC3B	Homo sapiens C-type lectin domain family 3, member B (CLEC3B), mRNA [NM_003278]	A_23_P69497	0.00215	0.0203	2.022	1.016
FANCE	Homo sapiens Fanconi anemia, complementation group E (FANCE), mRNA [NM_021922]	A_23_P42335	0.00216	0.0204	2.322	1.215
TMEM158	Homo sapiens transmembrane protein 158 (gene/pseudogene) (TMEM158), mRNA [NM_015444]	A_23_P369899	0.00217	0.0204	3.085	1.625
PSPH	Homo sapiens phosphoserine phosphatase (PSPH), mRNA [NM_004577]	A_32_P78816	0.00216	0.0204	2.023	1.017
WNT6	Homo sapiens wingless-type MMTV integration site family, member 6 (WNT6), mRNA [NM_006522]	A_23_P119916	0.00218	0.0205	2.122	1.085
ENPP4	Homo sapiens ectonucleotide pyrophosphatase/phosphodiesterase 4 (putative) (ENPP4), mRNA [NM_014936]	A_23_P70318	0.0022	0.0206	15.449	3.949
RHBDF2	Homo sapiens rhomboid 5 homolog 2 (Drosophila) (RHBDF2), transcript variant 1, mRNA [NM_024599]	A_23_P329870	0.00219	0.0206	2.837	1.504
ASB17	Homo sapiens ankyrin repeat and SOCS box containing 17 (ASB17), transcript variant 1, mRNA [NM_080868]	A_23_P344444	0.00221	0.0206	3.127	1.645
FAM19A2	Homo sapiens family with sequence similarity 19 (chemokine (C-C motif)-like), member A2 (FAM19A2), mRNA [NM_178539]	A_24_P297551	0.0022	0.0206	7.408	2.889
RAB18	Homo sapiens RAB18, member RAS oncogene family (RAB18), mRNA [NM_021252]	A_24_P132787	0.00219	0.0206	2.721	1.444

LOC284120	ALU1_HUMAN (P39188) Alu subfamily J sequence contamination warning entry, partial (10%) [THC2604076]	A_23_P250516	0.00223	0.0207	4.542	2.183
CNTNAP3B	Unknown	A_21_P0013565	0.00223	0.0207	2.24	1.164
PNPLA1	Homo sapiens patatin-like phospholipase domain containing 1 (PNPLA1), transcript variant 3, mRNA [NM_001145717]	A_32_P526255	0.00224	0.0208	3.354	1.746
LRTOMT	Homo sapiens leucine rich transmembrane and 0-methyltransferase domain containing (LRTOMT), transcript variant 1, mRNA [NM_145309]	A_23_P98763	0.00225	0.0208	2.39	1.257
FASTKD3	Homo sapiens FAST kinase domains 3 (FASTKD3), transcript variant 1, mRNA [NM_024091]	A_23_P58489	0.00225	0.0208	2.102	1.072
hCG_2045899	Homo sapiens leucine rich repeat containing 70 (LRRC70), mRNA [NM_181506]	A_23_P41664	0.00224	0.0208	3.358	1.748
UGT2B10	Homo sapiens UDP glucuronosyltransferase 2 family, polypeptide B10 (UGT2B10), transcript variant 1, mRNA [NM_001075]	A_23_P7342	0.00224	0.0208	3.679	1.879
PCDH24	Homo sapiens cadherin-related family member 2 (CDHR2), transcript variant 2, mRNA [NM_017675]	A_23_P133338	0.00227	0.0209	6.734	2.751
H6PD	Homo sapiens hexose-6-phosphate dehydrogenase (glucose 1-dehydrogenase) (H6PD), mRNA [NM_004285]	A_24_P626850	0.00226	0.0209	3.801	1.926
C7orf23	Homo sapiens chromosome 7 open reading frame 23 (C7orf23), mRNA [NM_024315]	A_23_P157283	0.00227	0.0209	4.835	2.273
GCH1	Homo sapiens GTP cyclohydrolase 1 (GCH1), transcript variant 1, mRNA [NM_000161]	A_24_P167642	0.00226	0.0209	3.681	1.88
LRG1	Homo sapiens leucine-rich alpha-2-glycoprotein 1 (LRG1), mRNA [NM_052972]	A_23_P50638	0.0023	0.0211	2.115	1.08
CPT1C	Homo sapiens carnitine palmitoyltransferase 1C (CPT1C), transcript variant 3, mRNA [NM_001199752]	A_33_P3392391	0.0023	0.0211	2.856	1.514
LOC100133732	Unknown	A_33_P3363959	0.00231	0.0212	5.544	2.471
C20orf107	Homo sapiens chromosome 20 open reading frame 107 (C20orf107), mRNA [NM_001013646]	A_33_P3404014	0.00231	0.0212	2.805	1.488
TSPAN13	Homo sapiens tetraspanin 13 (TSPAN13), mRNA [NM_014399]	A_23_P168610	0.00232	0.0212	2.195	1.134
TMEM22	Homo sapiens transmembrane protein 22 (TMEM22), transcript variant 1, mRNA [NM_025246]	A_24_P371281	0.00232	0.0212	5.49	2.457
GTPBP1	Homo sapiens GTP binding protein 1 (GTPBP1), mRNA [NM_004286]	A_24_P168760	0.00232	0.0212	2.067	1.048
YPEL1	Homo sapiens yippee-like 1 (Drosophila) (YPEL1), mRNA [NM_013313]	A_33_P3257165	0.00234	0.0213	3.864	1.95
EPB41L4B	Homo sapiens erythrocyte membrane protein band 4.1 like 4B (EPB41L4B), transcript variant 1, mRNA [NM_018424]	A_23_P216556	0.00236	0.0214	4.68	2.226
C3orf35	Homo sapiens chromosome 3 open reading frame 35 (C3orf35), transcript variant B, mRNA [NM_178339]	A_32_P430695	0.00235	0.0214	2.184	1.127
DYSFIP1	Homo sapiens protein phosphatase 1, regulatory subunit 27 (PPP1R27), mRNA [NM_001007533]	A_33_P3287472	0.00235	0.0214	3.305	1.725
GALNTL4	Homo sapiens UDP-N-acetyl-alpha-D-galactosamine:polypeptide N-acetylgalactosaminyltransferase-like 4 (GALNTL4), mRNA [NM_198516]	A_23_P139418	0.00239	0.0216	4.052	2.019
SLC12A6	Homo sapiens solute carrier family 12 (potassium/chloride transporters), member 6 (SLC12A6), transcript variant 5, mRNA [NM_001042496]	A_33_P3212112	0.00239	0.0216	2.857	1.515
C19orf67	PREDICTED: Homo sapiens chromosome 19 open reading frame 67 (C19orf67), mRNA [XM_003403704]	A_24_P910688	0.00241	0.0217	3.121	1.642
SLC22A18AS	Homo sapiens solute carrier family 22 (organic cation transporter), member 18 antisense (SLC22A18AS), mRNA [NM_007105]	A_33_P3316587	0.00242	0.0217	2.842	1.507
HECW2	Homo sapiens HECT, C2 and WW domain containing E3 ubiquitin protein ligase 2 (HECW2), mRNA [NM_020760]	A_33_P3270311	0.00243	0.0218	8.875	3.15
CNRIP1	Homo sapiens cannabinoid receptor interacting protein 1 (CNRIP1), transcript variant CRIP1a, mRNA [NM_015463]	A_23_P329353	0.00244	0.0218	2.545	1.347
LOC126987	Unknown	A_33_P3286162	0.00245	0.0219	2.73	1.449
POLR2K	Homo sapiens polymerase (RNA) II (DNA directed) polypeptide K, 7.0kDa (POLR2K), mRNA [NM_005034]	A_23_P157449	0.00247	0.022	2.505	1.325
TPST1	Homo sapiens tyrosylprotein sulfotransferase 1 (TPST1), mRNA [NM_003596]	A_23_P145965	0.00247	0.022	10.671	3.416
CRTAM	Homo sapiens cytotoxic and regulatory T cell molecule (CRTAM), mRNA [NM_019604]	A_23_P305092	0.00246	0.022	3.272	1.71
BTN2A1	Homo sapiens butyrophilin, subfamily 2, member A1 (BTN2A1), transcript variant 4, mRNA [NM_001197234]	A_21_P0004860	0.00252	0.0222	2.146	1.102
CD247	Homo sapiens CD247 molecule (CD247), transcript variant 1, mRNA [NM_198053]	A_23_P34676	0.00252	0.0222	2.475	1.308

DFNB31	Homo sapiens deafness, autosomal recessive 31 (DFNB31), transcript variant 1, mRNA [NM_015404]	A_23_P83351	0.00253	0.0223	2.461	1.299
MYOZ1	Homo sapiens myozenin 1 (MYOZ1), mRNA [NM_021245]	A_23_P1320	0.00253	0.0223	3.269	1.709
MPP6	Homo sapiens membrane protein, palmitoylated 6 (MAGUK p55 subfamily member 6) (MPP6), mRNA [NM_016447]	A_23_P71053	0.00253	0.0223	2.34	1.227
SLC14A2	Homo sapiens solute carrier family 14 (urea transporter), member 2 (SLC14A2), transcript variant 1, mRNA [NM_007163]	A_23_P27353	0.00255	0.0224	2.518	1.332
ADAP2	Homo sapiens ArfGAP with dual PH domains 2 (ADAP2), mRNA [NM_018404]	A_23_P49816	0.00259	0.0226	3.701	1.888
ZFYVE16	Homo sapiens zinc finger, FYVE domain containing 16 (ZFYVE16), transcript variant 2, mRNA [NM_001105251]	A_24_P286054	0.00259	0.0226	2.286	1.193
NAV3	Homo sapiens neuron navigator 3 (NAV3), mRNA [NM_014903]	A_23_P13740	0.00258	0.0226	14.438	3.852
A_19_P00316256	Unknown	A_19_P00316256	0.00261	0.0227	2.716	1.442
TMEM194A	Homo sapiens transmembrane protein 194A (TMEM194A), transcript variant 2, mRNA [NM_015257]	A_24_P126628	0.00262	0.0228	2.055	1.039
PRSS21	Homo sapiens protease, serine, 21 (testisin) (PRSS21), transcript variant 3, mRNA [NM_144957]	A_24_P339126	0.00268	0.023	2.158	1.11
FLJ77644	Unknown	A_32_P101860	0.00267	0.023	3.575	1.838
IGSF3	Homo sapiens immunoglobulin superfamily, member 3 (IGSF3), transcript variant 2, mRNA [NM_001007237]	A_21_P0012337	0.00268	0.023	4.346	2.12
CD68	Homo sapiens CD68 molecule (CD68), transcript variant 1, mRNA [NM_001251]	A_23_P15394	0.00267	0.023	3.533	1.821
HSD11B1	Homo sapiens hydroxysteroid (11-beta) dehydrogenase 1 (HSD11B1), transcript variant 2, mRNA [NM_181755]	A_23_P63209	0.00266	0.023	33.62	5.071
AQPEP	Homo sapiens laeverin (AQPEP), mRNA [NM_173800]	A_33_P3251522	0.00269	0.0231	2.37	1.245
HK3	Homo sapiens hexokinase 3 (white cell) (HK3), nuclear gene encoding mitochondrial protein, mRNA [NM_002115]	A_23_P213584	0.00271	0.0232	9.338	3.223
ZCCHC11	Homo sapiens zinc finger, CCHC domain containing 11 (ZCCHC11), transcript variant 1, mRNA [NM_001009881]	A_23_P34433	0.0027	0.0232	1.549	0.631
DSTN	Homo sapiens destrin (actin depolymerizing factor) (DSTN), transcript variant 2, mRNA [NM_001011546]	A_23_P408095	0.00271	0.0232	2.273	1.184
ENST00000396039	par-6 partitioning defective 6 homolog beta (C. elegans) [Source:HGNC Symbol;Acc:16245] [ENST00000396039]	A_33_P3227457	0.00274	0.0233	2.303	1.204
SLC2A6	Homo sapiens solute carrier family 2 (facilitated glucose transporter), member 6 (SLC2A6), transcript variant 1, mRNA [NM_017585]	A_33_P3398912	0.00274	0.0233	19.699	4.3
LOC644936	Homo sapiens POTE ankyrin domain family, member M (POTEM), mRNA [NM_001145442]	A_24_P238744	0.00275	0.0234	2.233	1.159
LOC387763	Homo sapiens chromosome 11 open reading frame 96 (C11orf96), mRNA [NM_001145033]	A_32_P74409	0.00276	0.0234	4.311	2.108
C2orf15	Homo sapiens chromosome 2 open reading frame 15 (C2orf15), mRNA [NM_144706]	A_33_P3216853	0.00276	0.0234	5.509	2.462
EML1	Homo sapiens echinoderm microtubule associated protein like 1 (EML1), transcript variant 1, mRNA [NM_001008707]	A_23_P205746	0.00275	0.0234	4.527	2.179
VKORC1L1	Homo sapiens vitamin K epoxide reductase complex, subunit 1-like 1 (VKORC1L1), mRNA [NM_173517]	A_23_P413815	0.00278	0.0235	2.877	1.524
DB335107	DB335107 SYNOV4 Homo sapiens cDNA clone SYNOV4009599 3', mRNA sequence [DB335107]	A_33_P3395314	0.00279	0.0235	3.638	1.863
VCL	Homo sapiens vinculin (VCL), transcript variant 1, mRNA [NM_014000]	A_24_P47182	0.00279	0.0235	2.017	1.012
PRICKLE1	Homo sapiens prickle homolog 1 (Drosophila) (PRICKLE1), transcript variant 1, mRNA [NM_153026]	A_23_P408285	0.00277	0.0235	8.655	3.114
RND3	Homo sapiens Rho family GTPase 3 (RND3), mRNA [NM_005168]	A_23_P142849	0.00277	0.0235	2.943	1.557
FERMT2	Homo sapiens fermitin family member 2 (FERMT2), transcript variant 3, mRNA [NM_001135000]	A_33_P3344482	0.00282	0.0237	4.765	2.253
WDR74	WD repeat domain 74 [Source:HGNC Symbol;Acc:25529] [ENST00000538098]	A_33_P3265394	0.00284	0.0238	7.066	2.821
CA13	Homo sapiens carbonic anhydrase XIII (CA13), mRNA [NM_198584]	A_23_P381714	0.00287	0.0239	2.138	1.096
KCND1	Homo sapiens potassium voltage-gated channel, Shal-related subfamily, member 1 (KCND1), mRNA [NM_004979]	A_23_P315772	0.00287	0.0239	4.308	2.107
FBXO9	Homo sapiens F-box protein 9 (FBXO9), transcript variant 2, mRNA [NM_033480]	A_23_P254120	0.00288	0.024	2.473	1.306
A_33_P3615955	Unknown	A_33_P3615955	0.0029	0.0241	2.696	1.431

PFKFB3	Homo sapiens 6-phosphofructo-2-kinase/fructose-2,6-biphosphatase 3 (PFKFB3), transcript variant 1, mRNA [NM_004566]	A_24_P261259	0.0029	0.0241	2.193	1.133
CR736977	CR736977 Soares_testis_NHT Homo sapiens cDNA clone IMAGp971B2176 ; IMAGE:1755160 5', mRNA sequence [CR736977]	A_33_P3414868	0.00291	0.0242	4.324	2.112
FAM89A	Homo sapiens family with sequence similarity 89, member A (FAM89A), mRNA [NM_198552]	A_24_P418408	0.00294	0.0243	2.238	1.162
TMEM168	Homo sapiens transmembrane protein 168 (TMEM168), mRNA [NM_022484]	A_24_P414952	0.00294	0.0243	2.75	1.459
CGRRF1	Homo sapiens cell growth regulator with ring finger domain 1 (CGRRF1), mRNA [NM_006568]	A_23_P37283	0.00293	0.0243	2.545	1.348
TRIB2	Homo sapiens tribbles homolog 2 (Drosophila) (TRIB2), transcript variant 1, mRNA [NM_021643]	A_24_P396753	0.00296	0.0244	2.149	1.103
ADPGK	Homo sapiens ADP-dependent glucokinase (ADPGK), transcript variant 1, mRNA [NM_031284]	A_33_P3406927	0.00296	0.0244	2.169	1.117
FBXL5	Homo sapiens F-box and leucine-rich repeat protein 5 (FBXL5), transcript variant 1, mRNA [NM_012161]	A_23_P213247	0.00297	0.0245	2.025	1.018
KANK4	Homo sapiens KN motif and ankyrin repeat domains 4 (KANK4), mRNA [NM_181712]	A_32_P51237	0.00297	0.0245	3.512	1.812
FXYD6	Homo sapiens FXYD domain containing ion transport regulator 6 (FXYD6), transcript variant 1, mRNA [NM_022003]	A_23_P150394	0.00301	0.0246	2.421	1.276
VTCN1	Homo sapiens V-set domain containing T cell activation inhibitor 1 (VTCN1), mRNA [NM_024626]	A_23_P518	0.00299	0.0246	9.851	3.3
DHFRL1	Homo sapiens dihydrofolate reductase-like 1 (DHFRL1), transcript variant 2, mRNA [NM_176815]	A_24_P186065	0.00301	0.0246	2.43	1.281
CARD17	Homo sapiens caspase recruitment domain family, member 17 (CARD17), mRNA [NM_001007232]	A_24_P192805	0.00299	0.0246	44.1	5.463
KIAA0746	Homo sapiens sel-1 suppressor of lin-12-like 3 (C. elegans) (SEL1L3), mRNA [NM_015187]	A_23_P426021	0.00301	0.0246	3.569	1.835
IRX5	Homo sapiens iroquois homeobox 5 (IRX5), transcript variant 1, mRNA [NM_005853]	A_24_P48057	0.00299	0.0246	2.501	1.322
POTEF	Homo sapiens POTE ankyrin domain family, member F (POTEF), mRNA [NM_001099771]	A_24_P59220	0.00306	0.0249	2.765	1.467
PKIA	Homo sapiens protein kinase (cAMP-dependent, catalytic) inhibitor alpha (PKIA), transcript variant 1, mRNA [NM_006823]	A_23_P31765	0.00308	0.025	2.261	1.177
DUSP22	Homo sapiens dual specificity phosphatase 22 (DUSP22), mRNA [NM_020185]	A_23_P120254	0.0031	0.0251	2.185	1.128
LOC100652777	PREDICTED: Homo sapiens group 10 secretory phospholipase A2-like (LOC100652777), mRNA [XM_003403464]	A_21_P0014013	0.00311	0.0251	2.502	1.323
CCDC144A	Homo sapiens coiled-coil domain containing 144A (CCDC144A), mRNA [NM_014695]	A_33_P3311750	0.00313	0.0252	3.983	1.994
FBXO48	Homo sapiens F-box protein 48 (FBXO48), mRNA [NM_001024680]	A_33_P3214745	0.00313	0.0252	2.57	1.362
CD44	Homo sapiens CD44 molecule (Indian blood group) (CD44), transcript variant 1, mRNA [NM_000610]	A_33_P3294509	0.00314	0.0252	20.962	4.39
SCNN1A	Homo sapiens sodium channel, nonvoltage-gated 1 alpha (SCNN1A), transcript variant 1, mRNA [NM_001038]	A_23_P128323	0.00313	0.0252	3.56	1.832
HLA-DRB5	Homo sapiens major histocompatibility complex, class II, DR beta 5 (HLA-DRB5), mRNA [NM_002125]	A_23_P45099	0.00315	0.0253	2.496	1.319
AOC3	Homo sapiens amine oxidase, copper containing 3 (vascular adhesion protein 1) (AOC3), mRNA [NM_003734]	A_23_P426305	0.00319	0.0254	3.343	1.741
TRIM38	Homo sapiens tripartite motif containing 38 (TRIM38), mRNA [NM_006355]	A_24_P294851	0.00317	0.0254	4.058	2.021
TAGAP	Homo sapiens T-cell activation RhoGTPase activating protein (TAGAP), transcript variant 2, mRNA [NM_054114]	A_24_P354724	0.00317	0.0254	3.749	1.907
C9orf72	Homo sapiens chromosome 9 open reading frame 72 (C9orf72), transcript variant 2, mRNA [NM_145005]	A_23_P405873	0.00318	0.0254	4.803	2.264
TMEM55A	Homo sapiens transmembrane protein 55A (TMEM55A), mRNA [NM_018710]	A_23_P422083	0.00317	0.0254	2.902	1.537
THC2709213	Unknown	A_33_P3233489	0.00321	0.0255	2.107	1.075
BPNT1	Homo sapiens 3'(2'), 5'-bisphosphate nucleotidase 1 (BPNT1), mRNA [NM_006085]	A_23_P104046	0.00322	0.0256	2.223	1.152
PTAFR	Homo sapiens platelet-activating factor receptor (PTAFR), transcript variant 3, mRNA [NM_000952]	A_24_P102821	0.00324	0.0256	4.197	2.07
PLD3	Homo sapiens phospholipase D family, member 3 (PLD3), transcript variant 2, mRNA [NM_012268]	A_23_P27515	0.00324	0.0256	2.254	1.173
FBXL2	Homo sapiens F-box and leucine-rich repeat protein 2 (FBXL2), transcript variant 1, mRNA [NM_012157]	A_23_P17955	0.00325	0.0257	2.606	1.382
CFB	Homo sapiens complement factor B (CFB), mRNA [NM_001710]	A_23_P156687	0.00325	0.0257	6.497	2.7
DSG1	Homo sapiens desmoglein 1 (DSG1), mRNA [NM_001942]	A_33_P3265189	0.00327	0.0258	4.699	2.232
GFRA3	Homo sapiens GDNF family receptor alpha 3 (GFRA3), mRNA [NM_001496]	A_23_P41987	0.00331	0.0259	8.478	3.084
IFI35	Homo sapiens interferon-induced protein 35 (IFI35), mRNA [NM_005533]	A_23_P152782	0.00331	0.0259	2.219	1.15

CCDC113	Homo sapiens coiled-coil domain containing 113 (CCDC113), transcript variant 1, mRNA [NM_014157]	A_24_P73730	0.00333	0.026	2.246	1.167
LOC729549	Unknown	A_33_P3247252	0.00337	0.0261	4.728	2.241
POTEE	Homo sapiens POTE ankyrin domain family, member E (POTEE), mRNA [NM_001083538]	A_24_P410017	0.00341	0.0263	2.157	1.109
AIF1L	Homo sapiens allograft inflammatory factor 1-like (AIF1L), transcript variant 3, mRNA [NM_001185095]	A_23_P392384	0.00341	0.0263	10.648	3.413
CCT6B	Homo sapiens chaperonin containing TCP1, subunit 6B (zeta 2) (CCT6B), transcript variant 1, mRNA [NM_006584]	A_23_P4082	0.00342	0.0264	2.163	1.113
CCDC82	Homo sapiens coiled-coil domain containing 82 (CCDC82), mRNA [NM_024725]	A_23_P312246	0.00342	0.0264	2.014	1.01
KBTBD11	Homo sapiens kelch repeat and BTB (POZ) domain containing 11 (KBTBD11), mRNA [NM_014867]	A_23_P94319	0.00343	0.0264	20.082	4.328
LOC388279	Unknown	A_33_P3364443	0.00343	0.0264	2.159	1.11
EPS8	Homo sapiens epidermal growth factor receptor pathway substrate 8 (EPS8), mRNA [NM_004447]	A_23_P136347	0.00344	0.0264	6.087	2.606
C9orf84	Homo sapiens chromosome 9 open reading frame 84 (C9orf84), transcript variant 1, mRNA [NM_173521]	A_32_P341615	0.00346	0.0265	5.019	2.327
AGPAT9	Homo sapiens 1-acylglycerol-3-phosphate O-acyltransferase 9 (AGPAT9), mRNA [NM_032717]	A_23_P69810	0.00348	0.0266	4.066	2.024
SLC35D2	Homo sapiens solute carrier family 35, member D2 (SLC35D2), mRNA [NM_007001]	A_23_P32029	0.00348	0.0266	2.139	1.097
A_19_P00809624	Homo sapiens family with sequence similarity 200, member B (FAM200B), mRNA [NM_001145191]	A_19_P00809624	0.00348	0.0266	3.476	1.797
PHLDA1	Homo sapiens pleckstrin homology-like domain, family A, member 1 (PHLDA1), mRNA [NM_007350]	A_24_P915692	0.00347	0.0266	20.338	4.346
MOXD1	Homo sapiens monooxygenase, DBH-like 1 (MOXD1), transcript variant 2, mRNA [NM_015529]	A_23_P31064	0.00353	0.0268	7.145	2.837
ZDHHC2	Homo sapiens zinc finger, DHHC-type containing 2 (ZDHHC2), mRNA [NM_016353]	A_33_P3267665	0.00352	0.0268	4.537	2.182
DNAJA1	Homo sapiens DnaJ (Hsp40) homolog, subfamily A, member 1 (DNAJA1), mRNA [NM_001539]	A_24_P9671	0.00356	0.0269	2.692	1.428
CSTA	Homo sapiens cystatin A (stefin A) (CSTA), mRNA [NM_005213]	A_23_P41114	0.00356	0.0269	32.651	5.029
MPZL3	myelin protein zero-like 3 [Source:HGNC Symbol;Acc:27279] [ENST00000278949]	A_24_P270033	0.00356	0.0269	2.685	1.425
ATP6V1H	Homo sapiens ATPase, H+ transporting, lysosomal 50/57kDa, V1 subunit H (ATP6V1H), transcript variant 1, mRNA [NM_015941]	A_24_P363679	0.00355	0.0269	2.261	1.177
ENST00000298876	Homo sapiens chromosome 12 open reading frame 70 (C12orf70), mRNA [NM_001145010]	A_23_P389250	0.00358	0.027	7.285	2.865
MDM1	Homo sapiens Mdm1 nuclear protein homolog (mouse) (MDM1), transcript variant 2, mRNA [NM_020128]	A_23_P204782	0.0036	0.0271	3.219	1.686
GEM	Homo sapiens GTP binding protein overexpressed in skeletal muscle (GEM), transcript variant 1, mRNA [NM_005261]	A_23_P257043	0.00361	0.0271	4.572	2.193
ITGA9	Homo sapiens integrin, alpha 9 (ITGA9), mRNA [NM_002207]	A_23_P252193	0.00364	0.0272	3.118	1.64
ULK2	Homo sapiens unc-51-like kinase 2 (C. elegans) (ULK2), transcript variant 1, mRNA [NM_014683]	A_23_P55107	0.00364	0.0272	3.072	1.619
TRPS1	Homo sapiens trichorhinophalangeal syndrome I (TRPS1), mRNA [NM_014112]	A_23_P134755	0.00364	0.0272	2.653	1.407
GPX8	Homo sapiens glutathione peroxidase 8 (putative) (GPX8), mRNA [NM_001008397]	A_23_P122052	0.00363	0.0272	4.629	2.211
C13orf16	Homo sapiens chromosome 13 open reading frame 16 (C13orf16), mRNA [NM_152324]	A_24_P56330	0.00366	0.0273	2.822	1.497
TTC14	Homo sapiens tetratricopeptide repeat domain 14 (TTC14), transcript variant 2, mRNA [NM_001042601]	A_23_P212511	0.00368	0.0274	2.032	1.023
C4BPA	Homo sapiens complement component 4 binding protein, alpha (C4BPA), mRNA [NM_000715]	A_23_P97541	0.00367	0.0274	16.186	4.017
LOC648556	Homo sapiens uncharacterized gastric protein ZA43P mRNA, partial cds. [AF264629]	A_33_P3715909	0.00368	0.0274	2.045	1.032
FOSL2	Homo sapiens FOS-like antigen 2 (FOSL2), mRNA [NM_005253]	A_23_P348121	0.00373	0.0276	19.464	4.283
TCTN2	Homo sapiens tectonic family member 2 (TCTN2), transcript variant 1, mRNA [NM_024809]	A_23_P64712	0.00375	0.0277	2.62	1.39
CLRN2	Homo sapiens clarin 2 (CLRN2), mRNA [NM_001079827]	A_33_P3236077	0.00378	0.0279	3.4	1.766
LOC646821	Unknown	A_33_P3415526	0.00379	0.0279	2.231	1.157
RNFT1	Homo sapiens ring finger protein, transmembrane 1 (RNFT1), mRNA [NM_016125]	A_23_P207299	0.00379	0.0279	2.097	1.068
MYOZ3	Homo sapiens myozenin 3 (MYOZ3), transcript variant 2, mRNA [NM_133371]	A_32_P88415	0.0038	0.028	2.171	1.118
C22orf23	Homo sapiens chromosome 22 open reading frame 23 (C22orf23), transcript variant 1, mRNA [NM_032561]	A_24_P336137	0.00381	0.028	2.479	1.31
LOC100130301	Q3JGI8_BURP1 (Q3JGI8) Permease, partial (4%) [THC2705386]	A_33_P3403723	0.00381	0.028	3.758	1.91
KCNE3	Homo sapiens potassium voltage-gated channel, Isk-related family, member 3 (KCNE3), mRNA [NM_005472]	A_23_P24948	0.00381	0.028	3.536	1.822
PLIN4	Homo sapiens perilipin 4 (PLIN4), mRNA [NM_001080400]	A_33_P3400763	0.00383	0.0281	4.458	2.156
ENST00000406220	Uncharacterized protein [Source:UniProtKB/TrEMBL;Acc:B5MCU5] [ENST00000406220]	A_33_P3370615	0.00383	0.0281	4.304	2.106

MYO1F	Homo sapiens myosin IF (MYO1F), mRNA [NM_012335]	A_23_P142447	0.00386	0.0282	3.166	1.663
PACRGL	Unknown	A_21_P0014718	0.00388	0.0283	2.265	1.18
PTPRK	Homo sapiens protein tyrosine phosphatase, receptor type, K (PTPRK), transcript variant 2, mRNA [NM_002844]	A_32_P99100	0.00387	0.0283	5.348	2.419
KLKBL4	Homo sapiens protease, serine, 54 (PRSS54), mRNA [NM_001080492]	A_23_P88776	0.0039	0.0284	4.554	2.187
ARHGAP24	Rho GTPase activating protein 24 [Source:HGNC Symbol;Acc:25361] [ENST00000509709]	A_33_P3236030	0.0039	0.0284	2.035	1.025
GPR160	Homo sapiens G protein-coupled receptor 160 (GPR160), mRNA [NM_014373]	A_23_P167005	0.0039	0.0284	2.156	1.109
A_33_P3240144	Unknown	A_33_P3240144	0.00392	0.0285	3.344	1.742
UBR1	Homo sapiens ubiquitin protein ligase E3 component n-recognin 1 (UBR1), mRNA [NM_174916]	A_23_P152066	0.00397	0.0286	2.513	1.33
ERO1L	Homo sapiens ERO1-like (S. cerevisiae) (ERO1L), mRNA [NM_014584]	A_23_P106145	0.00397	0.0286	2.031	1.022
PCOLCE2	Homo sapiens procollagen C-endopeptidase enhancer 2 (PCOLCE2), mRNA [NM_013363]	A_23_P57709	0.00396	0.0286	6.109	2.611
PDE8B	Homo sapiens phosphodiesterase 8B (PDE8B), transcript variant 1, mRNA [NM_003719]	A_24_P197537	0.00395	0.0286	2.215	1.147
SPAG17	Homo sapiens sperm associated antigen 17 (SPAG17), mRNA [NM_206996]	A_23_P319783	0.004	0.0288	4.887	2.289
ISCA1	Homo sapiens iron-sulfur cluster assembly 1 homolog (S. cerevisiae) (ISCA1), mRNA [NM_030940]	A_23_P216766	0.00403	0.0289	2.065	1.046
GPD2	Homo sapiens glycerol-3-phosphate dehydrogenase 2 (mitochondrial) (GPD2), nuclear gene encoding mitochondrial protein, transcript variant 1, mRNA [NM_001083112]	A_33_P3354569	0.00403	0.0289	3.443	1.784
LOC144874	Homo sapiens G protein-coupled receptor 180 (GPR180), mRNA [NM_180989]	A_33_P3666797	0.00402	0.0289	2.001	1.001
SIRT5	Homo sapiens sirtuin 5 (SIRT5), transcript variant 1, mRNA [NM_012241]	A_32_P151366	0.00405	0.029	2.026	1.019
SEPP1	Homo sapiens selenoprotein P, plasma, 1 (SEPP1), transcript variant 1, mRNA [NM_005410]	A_23_P121926	0.00405	0.029	3.253	1.702
LOC553137	Homo sapiens cDNA FLJ41422 fis, clone BRHIP2003048. [AK123416]	A_33_P3275973	0.00411	0.0292	6.629	2.729
SLC26A8	Homo sapiens solute carrier family 26, member 8 (SLC26A8), transcript variant 1, mRNA [NM_052961]	A_23_P30950	0.0041	0.0292	9.808	3.294
OSBPL11	Homo sapiens oxysterol binding protein-like 11 (OSBPL11), mRNA [NM_022776]	A_23_P6914	0.0041	0.0292	2.183	1.126
PGR	Homo sapiens progesterone receptor (PGR), transcript variant 2, mRNA [NM_000926]	A_23_P138938	0.0041	0.0292	2.644	1.403
SRPX2	Homo sapiens sushi-repeat containing protein, X-linked 2 (SRPX2), mRNA [NM_014467]	A_23_P136978	0.00417	0.0294	8.629	3.109
LOC399804	AGENCOURT_10185273 NIH_MGC_126 Homo sapiens cDNA clone IMAGE:6562423 5', mRNA sequence [BU535024]	A_33_P3360525	0.00416	0.0294	2.078	1.055
ENST00000368971	Q2M2B7_HUMAN (Q2M2B7) Sestrin 1, partial (68%) [THC2527965]	A_33_P3331267	0.00416	0.0294	3.119	1.641
IRS1	Homo sapiens insulin receptor substrate 1 (IRS1), mRNA [NM_005544]	A_24_P225679	0.00416	0.0294	3.133	1.648
HNMT	Homo sapiens histamine N-methyltransferase (HNMT), transcript variant 2, mRNA [NM_001024074]	A_24_P915806	0.00416	0.0294	3.142	1.652
SMARCA1	Homo sapiens SWI/SNF related, matrix associated, actin dependent regulator of chromatin, subfamily a, member 1 (SMARCA1), transcript variant 1, mRNA [NM_003069]	A_23_P44244	0.0042	0.0295	6.022	2.59
ACTG1	Homo sapiens actin, gamma 1 (ACTG1), transcript variant 2, mRNA [NM_001614]	A_32_P156963	0.0042	0.0295	2.41	1.269
IL4	Homo sapiens interleukin 4 (IL4), transcript variant 1, mRNA [NM_000589]	A_23_P213706	0.0042	0.0295	3.646	1.866
KNG1	Homo sapiens kininogen 1 (KNG1), transcript variant 2, mRNA [NM_000893]	A_23_P212258	0.00419	0.0295	16.772	4.068
ABCC2	Homo sapiens ATP-binding cassette, sub-family C (CFTR/MRP), member 2 (ABCC2), mRNA [NM_000392]	A_23_P44569	0.00422	0.0296	3.729	1.899
VGLL4	Homo sapiens vestigial like 4 (Drosophila) (VGLL4), transcript variant 2, mRNA [NM_014667]	A_23_P132595	0.00423	0.0296	2.912	1.542
CYB5B	Homo sapiens cytochrome b5 type B (outer mitochondrial membrane) (CYB5B), nuclear gene encoding mitochondrial protein, mRNA [NM_030579]	A_33_P3357535	0.00422	0.0296	2.051	1.036
COL12A1	Homo sapiens collagen, type XII, alpha 1 (COL12A1), transcript variant long, mRNA [NM_004370]	A_23_P214168	0.00422	0.0296	4.3	2.104
PKM2	Homo sapiens pyruvate kinase, muscle (PKM2), transcript variant 2, mRNA [NM_182470]	A_23_P399501	0.00421	0.0296	2.471	1.305
P2RX4	Homo sapiens purinergic receptor P2X, ligand-gated ion channel, 4 (P2RX4), mRNA [NM_002560]	A_33_P3362088	0.00427	0.0297	2.007	1.005
ENST00000395936	peripheral myelin protein 22 [Source:HGNC Symbol;Acc:9118] [ENST00000395936]	A_33_P3274930	0.00427	0.0297	2.895	1.533
NFATC4	Homo sapiens nuclear factor of activated T-cells, cytoplasmic, calcineurin-dependent 4 (NFATC4), transcript variant 1, mRNA [NM_001136022]	A_33_P3250083	0.00425	0.0297	4.652	2.218
ENST00000378938	glycerol kinase [Source:HGNC Symbol;Acc:4289] [ENST00000378938]	A_33_P3376264	0.00427	0.0297	3.637	1.863

CFH	Homo sapiens complement factor H (CFH), nuclear gene encoding mitochondrial protein, transcript variant 2, mRNA [NM_001014975]	A_33_P3367692	0.00426	0.0297	4.088	2.031
PLCB1	Homo sapiens phospholipase C, beta 1 (phosphoinositide-specific) (PLCB1), transcript variant 2, mRNA [NM_182734]	A_24_P941643	0.00426	0.0297	4.073	2.026
LOC100129397	Homo sapiens cDNA FLJ38522 fis, clone HCHON2000818. [AK095841]	A_33_P3300361	0.00428	0.0298	3.74	1.903
ENST00000378648	liver expressed antimicrobial peptide 2 [Source:HGNC Symbol;Acc:29571] [ENST00000483190]	A_33_P3341259	0.0043	0.0299	2.074	1.052
LOC100128916	Q4T080_TETNG (Q4T080) Chromosome undetermined SCAF11300, whole genome shotgun sequence. (Fragment), partial (8%) [THC2611572]	A_33_P3395422	0.0043	0.0299	3.95	1.982
BET1	Homo sapiens blocked early in transport 1 homolog (S. cerevisiae) (BET1), mRNA [NM_005868]	A_33_P3397905	0.00432	0.0299	2.157	1.109
TSPAN12	Homo sapiens tetraspanin 12 (TSPAN12), mRNA [NM_012338]	A_23_P145984	0.00432	0.0299	4.445	2.152
CP	Homo sapiens ceruloplasmin (ferroxidase) (CP), mRNA [NM_000096]	A_33_P3296587	0.00435	0.03	2.693	1.429
RBM7	Homo sapiens RNA binding motif protein 7 (RBM7), mRNA [NM_016090]	A_23_P138975	0.0044	0.0302	2.475	1.308
FAM105A	Homo sapiens family with sequence similarity 105, member A (FAM105A), mRNA [NM_019018]	A_23_P133438	0.0044	0.0302	6.183	2.628
FAM101B	Homo sapiens family with sequence similarity 101, member B (FAM101B), mRNA [NM_182705]	A_24_P299318	0.00442	0.0303	2.362	1.24
C6orf174	Homo sapiens chromosome 6 open reading frame 174 (C6orf174), mRNA [NM_001012279]	A_33_P3284453	0.00442	0.0303	2.429	1.28
C22orf39	Homo sapiens chromosome 22 open reading frame 39 (C22orf39), transcript variant 2, mRNA [NM_001166242]	A_21_P0000062	0.00443	0.0303	2.983	1.577
PTPN22	Homo sapiens protein tyrosine phosphatase, non-receptor type 22 (lymphoid) (PTPN22), transcript variant 2, mRNA [NM_012411]	A_23_P201181	0.00443	0.0303	19.739	4.303
PLCE1	Homo sapiens phospholipase C, epsilon 1 (PLCE1), transcript variant 1, mRNA [NM_016341]	A_23_P35617	0.00445	0.0304	3.631	1.86
KRT12	Homo sapiens keratin 12 (KRT12), mRNA [NM_000223]	A_23_P55448	0.00445	0.0304	7.959	2.993
FNDC3B	Homo sapiens fibronectin type III domain containing 3B (FNDC3B), transcript variant 1, mRNA [NM_022763]	A_24_P223124	0.00453	0.0307	1.974	0.981
NANOS1	Homo sapiens nanos homolog 1 (Drosophila) (NANOS1), mRNA [NM_199461]	A_24_P383609	0.00454	0.0308	2.469	1.304
WDR47	Homo sapiens WD repeat domain 47 (WDR47), transcript variant 2, mRNA [NM_014969]	A_23_P23748	0.00455	0.0308	2.329	1.22
C15orf33	Homo sapiens chromosome 15 open reading frame 33 (C15orf33), mRNA [NM_152647]	A_33_P3361758	0.00458	0.0309	5.201	2.379
GK5	Homo sapiens glycerol kinase 5 (putative) (GK5), transcript variant 1, mRNA [NM_001039547]	A_33_P3779229	0.00461	0.031	2.729	1.448
LOC647264	Uncharacterized protein [Source:UniProtKB/TrEMBL;Acc:F5H8E9] [ENST00000453638]	A_33_P3373446	0.00463	0.0311	3.874	1.954
SYCE2	Homo sapiens synaptonemal complex central element protein 2 (SYCE2), mRNA [NM_001105578]	A_32_P203528	0.00464	0.0312	4.136	2.048
ZNF91	Homo sapiens zinc finger protein 91 (ZNF91), mRNA [NM_003430]	A_33_P3217230	0.00465	0.0312	3.981	1.993
RBM11	Homo sapiens RNA binding motif protein 11 (RBM11), mRNA [NM_144770]	A_23_P342000	0.00468	0.0313	4.231	2.081
GGT1	Homo sapiens gamma-glutamyltransferase 1 (GGT1), transcript variant 1, mRNA [NM_005265]	A_21_P0012283	0.00467	0.0313	4.559	2.189
LAMA1	Homo sapiens laminin, alpha 1 (LAMA1), mRNA [NM_005559]	A_32_P313405	0.00471	0.0314	6.845	2.775
C21orf7	chromosome 21 open reading frame 7 [Source:HGNC Symbol;Acc:16457] [ENST00000399926]	A_33_P3238305	0.00469	0.0314	2.068	1.048
ADD3	Homo sapiens adducin 3 (gamma) (ADD3), transcript variant 1, mRNA [NM_016824]	A_24_P90097	0.00471	0.0314	2.898	1.535
ENST00000380201	DDRGK domain containing 1 [Source:HGNC Symbol;Acc:16110] [ENST00000380201]	A_33_P3299781	0.00475	0.0315	2.187	1.129
RAB27A	Homo sapiens RAB27A, member RAS oncogene family (RAB27A), transcript variant 1, mRNA [NM_004580]	A_24_P373174	0.00474	0.0315	2.972	1.571
COL7A1	Homo sapiens collagen, type VII, alpha 1 (COL7A1), mRNA [NM_000094]	A_23_P144071	0.00477	0.0316	2.649	1.406
C1QTNF6	Homo sapiens C1q and tumor necrosis factor related protein 6 (C1QTNF6), transcript variant 1, mRNA [NM_031910]	A_24_P211565	0.00476	0.0316	3.692	1.884
KCNH1	Homo sapiens potassium voltage-gated channel, subfamily H (eag-related), member 1 (KCNH1), transcript variant 1, mRNA [NM_172362]	A_23_P74943	0.00476	0.0316	2.965	1.568
A_19_P00806473	Q49AT5_HUMAN (Q49AT5) CPXM2 protein, partial (11%) [THC2741013]	A_19_P00806473	0.0048	0.0317	6.658	2.735
SPINK8	Homo sapiens serine peptidase inhibitor, Kazal type 8 (putative) (SPINK8), mRNA [NM_001080525]	A_33_P3258542	0.00479	0.0317	10.613	3.408
C4orf47	Homo sapiens chromosome 4 open reading frame 47 (C4orf47), mRNA [NM_001114357]	A_33_P3247175	0.00482	0.0317	2.645	1.403

LOC100130882	DA967691 STOMA2 Homo sapiens cDNA clone STOMA2006371 5', mRNA sequence [DA967691]	A_33_P3213832	0.00485	0.0318	2.335	1.224
AIM1	Homo sapiens absent in melanoma 1 (AIM1), mRNA [NM_001624]	A_23_P70785	0.00485	0.0318	5.125	2.358
RNASE6	Homo sapiens ribonuclease, RNase A family, k6 (RNASE6), mRNA [NM_005615]	A_23_P3014	0.00488	0.032	2.017	1.012
EMILIN2	Homo sapiens elastin microfibril interfacer 2 (EMILIN2), mRNA [NM_032048]	A_23_P27315	0.00488	0.032	2.618	1.388
FBXO36	Homo sapiens F-box protein 36 (FBXO36), mRNA [NM_174899]	A_33_P3401093	0.00488	0.032	2.149	1.104
FCGR2A	Homo sapiens Fc fragment of IgG, low affinity IIa, receptor (CD32) (FCGR2A), transcript variant 1, mRNA [NM_001136219]	A_33_P3403576	0.00493	0.0321	4.463	2.158
CCDC19	Homo sapiens coiled-coil domain containing 19 (CCDC19), mRNA [NM_012337]	A_23_P62642	0.00495	0.0322	2.509	1.327
ISG20	Homo sapiens interferon stimulated exonuclease gene 20kDa (ISG20), mRNA [NM_002201]	A_23_P32404	0.00494	0.0322	3.749	1.907
SEC61A2	Homo sapiens Sec61 alpha 2 subunit (S. cerevisiae) (SEC61A2), transcript variant 1, mRNA [NM_018144]	A_24_P415280	0.00496	0.0323	2.259	1.176
RANBP3L	Homo sapiens RAN binding protein 3-like (RANBP3L), transcript variant 1, mRNA [NM_001161429]	A_33_P3360072	0.00501	0.0324	2.824	1.498
KIF9	kinesin family member 9 [Source:HGNC Symbol;Acc:16666] [ENST00000487440]	A_33_P3356857	0.00499	0.0324	3.399	1.765
GGT5	Homo sapiens gamma-glutamyltransferase 5 (GGT5), transcript variant 1, mRNA [NM_001099781]	A_33_P3255304	0.00501	0.0324	5.276	2.399
OSCP1	Homo sapiens organic solute carrier partner 1 (OSCP1), transcript variant 1, mRNA [NM_145047]	A_33_P3289696	0.00503	0.0325	2.536	1.342
ENST00000399753	selenoprotein X, 1 [Source:HGNC Symbol;Acc:14133] [ENST00000399753]	A_33_P3325296	0.00503	0.0325	3.163	1.661
CITED2	Homo sapiens Cbp/p300-interacting transactivator, with Glu/Asp-rich carboxy-terminal domain, 2 (CITED2), transcript variant 1, mRNA [NM_006079]	A_23_P214969	0.00506	0.0326	2.136	1.095
CHI3L2	Homo sapiens chitinase 3-like 2 (CHI3L2), transcript variant 3, mRNA [NM_001025199]	A_23_P12082	0.00505	0.0326	11.892	3.572
FETUB	Homo sapiens fetuin B (FETUB), mRNA [NM_014375]	A_23_P144126	0.00511	0.0328	8.86	3.147
SNX10	Homo sapiens sorting nexin 10 (SNX10), transcript variant 2, mRNA [NM_013322]	A_24_P98109	0.00511	0.0328	2.466	1.302
ENST00000406691	Homo sapiens cDNA, FLJ99805. [AK309764]	A_33_P3233165	0.00512	0.0328	3.062	1.614
ZNF589	Homo sapiens zinc finger protein 589 (ZNF589), mRNA [NM_016089]	A_33_P3350306	0.00514	0.0329	2.007	1.005
UBAP1	Homo sapiens ubiquitin associated protein 1 (UBAP1), transcript variant 1, mRNA [NM_016525]	A_23_P123866	0.00518	0.033	2.14	1.097
UNC5C	Homo sapiens unc-5 homolog C (C. elegans) (UNC5C), mRNA [NM_003728]	A_23_P69617	0.00522	0.0332	9.042	3.177
LAIR2	Homo sapiens leukocyte-associated immunoglobulin-like receptor 2 (LAIR2), transcript variant 1, mRNA [NM_002288]	A_23_P209129	0.00523	0.0332	6.97	2.801
TUBA3D	Homo sapiens tubulin, alpha 3d (TUBA3D), mRNA [NM_080386]	A_23_P56736	0.00523	0.0332	2.673	1.419
CYP3A4	Homo sapiens cytochrome P450, family 3, subfamily A, polypeptide 4 (CYP3A4), transcript variant 1, mRNA [NM_017460]	A_33_P3251342	0.00521	0.0332	4.672	2.224
RASGRP1	Homo sapiens RAS guanyl releasing protein 1 (calcium and DAG-regulated) (RASGRP1), transcript variant 1, mRNA [NM_005739]	A_23_P124642	0.00522	0.0332	10.178	3.347
SC5DL	Homo sapiens sterol-C5-desaturase (ERG3 delta-5-desaturase homolog, S. cerevisiae)-like (SC5DL), transcript variant 2, mRNA [NM_001024956]	A_32_P41026	0.00521	0.0332	3.161	1.66
VPS13C	Homo sapiens vacuolar protein sorting 13 homolog C (S. cerevisiae) (VPS13C), transcript variant 2A, mRNA [NM_020821]	A_23_P206228	0.00526	0.0334	3.006	1.588
CD164	Homo sapiens CD164 molecule, sialomucin (CD164), transcript variant 1, mRNA [NM_006016]	A_23_P254756	0.00528	0.0334	2.418	1.274
FER1L3	Homo sapiens myoferlin (MYOF), transcript variant 1, mRNA [NM_013451]	A_23_P354387	0.00526	0.0334	8.748	3.129
GZF1	Homo sapiens GDNF-inducible zinc finger protein 1 (GZF1), mRNA [NM_022482]	A_23_P433188	0.00532	0.0336	1.781	0.833
ANO6	Homo sapiens anoctamin 6 (ANO6), transcript variant 1, mRNA [NM_001025356]	A_23_P389118	0.00532	0.0336	2.134	1.094
PGM5	Homo sapiens phosphoglucomutase 5 (PGM5), mRNA [NM_021965]	A_21_P0013658	0.00534	0.0337	4.012	2.004
SLC2A14	solute carrier family 2 (facilitated glucose transporter), member 14 [Source:HGNC Symbol;Acc:18301] [ENST00000431042]	A_32_P47754	0.00538	0.0338	2.333	1.222
A_33_P3395206	Unknown	A_33_P3395206	0.0054	0.0338	2.526	1.337
C14orf79	Homo sapiens chromosome 14 open reading frame 79 (C14orf79), mRNA [NM_174891]	A_23_P376870	0.00538	0.0338	2.234	1.16
SPPL2A	Homo sapiens signal peptide peptidase-like 2A (SPPL2A), mRNA [NM_032802]	A_23_P77073	0.00546	0.034	2.486	1.314
CTBS	Homo sapiens chitobiase, di-N-acetyl- (CTBS), mRNA [NM_004388]	A_24_P940135	0.00544	0.034	2.531	1.339

PLA2G4A	Homo sapiens phospholipase A2, group IVA (cytosolic, calcium-dependent) (PLA2G4A), mRNA [NM_024420]	A_23_P11685	0.00546	0.034	5.798	2.536
ARMETL1	Homo sapiens cerebral dopamine neurotrophic factor (CDNF), mRNA [NM_001029954]	A_24_P93309	0.00552	0.0342	2.182	1.126
AP3S2	Homo sapiens adaptor-related protein complex 3, sigma 2 subunit (AP3S2), transcript variant 1, mRNA [NM_005829]	A_24_P287691	0.00553	0.0343	2.079	1.056
MAPK13	Homo sapiens mitogen-activated protein kinase 13 (MAPK13), mRNA [NM_002754]	A_24_P406132	0.00554	0.0343	8.538	3.094
HLA-E	Homo sapiens major histocompatibility complex, class I, E (HLA-E), mRNA [NM_005516]	A_32_P460973	0.00555	0.0343	2.114	1.08
KLHL13	Homo sapiens kelch-like 13 (Drosophila) (KLHL13), transcript variant 1, mRNA [NM_033495]	A_23_P159974	0.0056	0.0345	3.051	1.609
TMOD4	Homo sapiens tropomodulin 4 (muscle) (TMOD4), mRNA [NM_013353]	A_23_P126605	0.00569	0.0348	2.241	1.164
GLT25D2	Homo sapiens glycosyltransferase 25 domain containing 2 (GLT25D2), mRNA [NM_015101]	A_24_P62505	0.00567	0.0348	4.196	2.069
TWF1	Homo sapiens twinfilin, actin-binding protein, homolog 1 (Drosophila) (TWF1), transcript variant 2, mRNA [NM_002822]	A_23_P48166	0.0057	0.0349	2.008	1.006
FOXP1	Homo sapiens forkhead box P1 (FOXP1), transcript variant 1, mRNA [NM_032682]	A_23_P155257	0.0057	0.0349	2.654	1.408
C14orf1	Homo sapiens chromosome 14 open reading frame 1 (C14orf1), mRNA [NM_007176]	A_23_P25935	0.00574	0.035	2.757	1.463
DW009619	MGC10nvl.1.1.1.1.H10.F.1 NIH_MGC_331 Homo sapiens cDNA clone MGC10nvl.1.1.1.1.H10, mRNA sequence [DW009619]	A_23_P24688	0.00574	0.035	12.518	3.646
IL1RL1	Homo sapiens interleukin 1 receptor-like 1 (IL1RL1), transcript variant 1, mRNA [NM_016232]	A_23_P51126	0.00574	0.035	30.277	4.92
HIPK2	Homo sapiens homeodomain interacting protein kinase 2 (HIPK2), transcript variant 1, mRNA [NM_022740]	A_24_P681011	0.00576	0.0351	2.011	1.008
HLA-DRB4	Homo sapiens major histocompatibility complex, class II, DR beta 4 (HLA-DRB4), mRNA [NM_021983]	A_24_P370472	0.00576	0.0351	3.374	1.754
ECHDC3	Homo sapiens enoyl CoA hydratase domain containing 3 (ECHDC3), nuclear gene encoding mitochondrial protein, mRNA [NM_024693]	A_24_P343621	0.00583	0.0354	2.134	1.093
LOC340900	Homo sapiens endogenous Bornavirus-like nucleoprotein 1 (EBLN1), mRNA [NM_001199938]	A_33_P3286273	0.00583	0.0354	5.091	2.348
ABAT	Homo sapiens 4-aminobutyrate aminotransferase (ABAT), nuclear gene encoding mitochondrial protein, transcript variant 2, mRNA [NM_000663]	A_33_P3268487	0.00585	0.0354	3.026	1.597
HEBP1	Homo sapiens heme binding protein 1 (HEBP1), mRNA [NM_015987]	A_23_P117082	0.00587	0.0355	2.145	1.101
TTC25	Homo sapiens tetratricopeptide repeat domain 25 (TTC25), mRNA [NM_031421]	A_23_P73150	0.00588	0.0355	2.763	1.466
TYSND1	Homo sapiens trypsin domain containing 1 (TYSND1), transcript variant 1, mRNA [NM_173555]	A_33_P3351101	0.0059	0.0356	2.456	1.296
LONRF2	Homo sapiens LON peptidase N-terminal domain and ring finger 2 (LONRF2), mRNA [NM_198461]	A_33_P3315519	0.00594	0.0357	4.468	2.16
VPS39	Homo sapiens vacuolar protein sorting 39 homolog (S. cerevisiae) (VPS39), mRNA [NM_015289]	A_24_P167825	0.00595	0.0358	2.069	1.049
LOC100130348	Homo sapiens uncharacterized LOC100130348 (LOC100130348), mRNA [NM_001242631]	A_21_P0000180	0.00598	0.0359	2.672	1.418
LOC100133869	Unknown	A_24_P152325	0.00604	0.0361	3.063	1.615
FOXA1	Homo sapiens forkhead box A1 (FOXA1), mRNA [NM_004496]	A_23_P37127	0.00606	0.0362	3.305	1.725
A_33_P3400023	Unknown	A_33_P3400023	0.00605	0.0362	3.455	1.789
LOC100127997	solute carrier family 37 (glycerol-3-phosphate transporter), member 3 [Source:HGNC Symbol;Acc:20651] [ENST00000493423]	A_33_P3259293	0.00611	0.0364	2.69	1.428
CARD16	Homo sapiens caspase recruitment domain family, member 16 (CARD16), transcript variant 1, mRNA [NM_001017534]	A_23_P64173	0.00619	0.0367	49.21	5.621
FBLN5	Homo sapiens fibulin 5 (FBLN5), mRNA [NM_006329]	A_23_P151805	0.00624	0.0368	3.144	1.653
OR51E2	Homo sapiens olfactory receptor, family 51, subfamily E, member 2 (OR51E2), mRNA [NM_030774]	A_24_P235756	0.00626	0.0369	4.536	2.181
HUS1B	Homo sapiens HUS1 checkpoint homolog b (S. pombe) (HUS1B), mRNA [NM_148959]	A_23_P133739	0.00634	0.0372	2.043	1.031
C16orf5	Homo sapiens chromosome 16 open reading frame 5 (C16orf5), transcript variant 1, mRNA [NM_001199054]	A_33_P3400943	0.00635	0.0372	3.217	1.686
PLEKHA2	Homo sapiens pleckstrin homology domain containing, family A (phosphoinositide binding specific) member 2 (PLEKHA2), mRNA [NM_021623]	A_33_P3339860	0.00635	0.0372	2.088	1.062
YPEL2	Homo sapiens yippee-like 2 (Drosophila) (YPEL2), mRNA [NM_001005404]	A_24_P787947	0.00634	0.0372	4.588	2.198
C8orf66	Homo sapiens mRNA; cDNA DKFZp434O0331 (from clone DKFZp434O0331). [AL834492]	A_33_P3482249	0.0064	0.0374	3.27	1.709

ADRBK2	Homo sapiens adrenergic, beta, receptor kinase 2 (ADRBK2), mRNA [NM_005160]	A_33_P3220919	0.00641	0.0374	3.765	1.913
RGS18	Homo sapiens regulator of G-protein signaling 18 (RGS18), mRNA [NM_130782]	A_23_P302550	0.0064	0.0374	27.282	4.77
ANTXR2	Homo sapiens anthrax toxin receptor 2 (ANTXR2), transcript variant 1, mRNA [NM_058172]	A_23_P170733	0.00648	0.0377	2.06	1.043
CLCN4	Homo sapiens chloride channel 4 (CLCN4), mRNA [NM_001830]	A_33_P3367596	0.00648	0.0377	13.058	3.707
SPP1	Homo sapiens secreted phosphoprotein 1 (SPP1), transcript variant 1, mRNA [NM_001040058]	A_23_P7313	0.0065	0.0377	2.388	1.256
FOXO1	Homo sapiens forkhead box O1 (FOXO1), mRNA [NM_002015]	A_24_P22079	0.00651	0.0378	2.709	1.438
SERP2	Homo sapiens stress-associated endoplasmic reticulum protein family member 2 (SERP2), mRNA [NM_001010897]	A_23_P139965	0.00656	0.0379	3.148	1.655
RAP2A	Homo sapiens RAP2A, member of RAS oncogene family (RAP2A), mRNA [NM_021033]	A_24_P81965	0.00663	0.0382	2.56	1.356
TRIM43	Homo sapiens tripartite motif containing 43 (TRIM43), mRNA [NM_138800]	A_33_P3248163	0.00666	0.0383	3.169	1.664
LOC652554	Unknown	A_33_P3310276	0.00667	0.0383	2.666	1.415
GKN1	Homo sapiens gastrokine 1 (GKN1), mRNA [NM_019617]	A_23_P44436	0.00674	0.0386	3.727	1.898
FBXO3	Homo sapiens CD59 molecule, complement regulatory protein (CD59), transcript variant 1, mRNA [NM_203330]	A_24_P784765	0.00677	0.0387	2.242	1.165
JAM2	Homo sapiens junctional adhesion molecule 2 (JAM2), mRNA [NM_021219]	A_33_P3226212	0.0068	0.0388	2.94	1.556
AK126778	Homo sapiens cDNA FLJ44826 fis, clone BRACE3046762. [AK126778]	A_33_P3373203	0.00682	0.0388	2.612	1.385
DAPL1	Homo sapiens death associated protein-like 1 (DAPL1), mRNA [NM_001017920]	A_23_P165598	0.0068	0.0388	14.801	3.888
SVOPL	Homo sapiens SVOP-like (SVOPL), transcript variant 2, mRNA [NM_174959]	A_23_P307860	0.00684	0.0389	3.611	1.852
CCRL1	Homo sapiens chemokine (C-C motif) receptor-like 1 (CCRL1), transcript variant 1, mRNA [NM_178445]	A_23_P6909	0.00683	0.0389	3.415	1.772
PPL	Homo sapiens periplakin (PPL), mRNA [NM_002705]	A_23_P106906	0.00685	0.039	5.574	2.479
SH3BGR	Homo sapiens SH3 domain binding glutamic acid-rich protein (SH3BGR), transcript variant 1, mRNA [NM_007341]	A_33_P3327822	0.00689	0.0391	2.3	1.202
PGLYRP1	Homo sapiens peptidoglycan recognition protein 1 (PGLYRP1), mRNA [NM_005091]	A_23_P208747	0.00691	0.0392	6.013	2.588
MEIS2	Homo sapiens Meis homeobox 2 (MEIS2), transcript variant a, mRNA [NM_170677]	A_23_P88602	0.00693	0.0392	2.071	1.05
AX746564	Unknown	A_33_P3282740	0.00693	0.0392	2.848	1.51
A_19_P00320841	Homo sapiens chromosome 17 open reading frame 51 (C17orf51), mRNA [NM_001113434]	A_19_P00320841	0.00694	0.0393	2.356	1.236
MAP2K1	Homo sapiens mitogen-activated protein kinase kinase 1 (MAP2K1), mRNA [NM_002755]	A_23_P20248	0.00699	0.0395	3.117	1.64
GRAMD1C	Homo sapiens GRAM domain containing 1C (GRAMD1C), transcript variant 1, mRNA [NM_017577]	A_23_P253012	0.00707	0.0397	6.135	2.617
LRRN4CL	Homo sapiens LRRN4 C-terminal like (LRRN4CL), mRNA [NM_203422]	A_33_P3221129	0.00706	0.0397	3.395	1.763
KITLG	Homo sapiens KIT ligand (KITLG), transcript variant b, mRNA [NM_000899]	A_24_P133253	0.00706	0.0397	22.957	4.521
KIF20A	Homo sapiens kinesin family member 20A (KIF20A), mRNA [NM_005733]	A_23_P256956	0.00714	0.0399	2.293	1.197
SPG11	Homo sapiens spastic paraplegia 11 (autosomal recessive) (SPG11), transcript variant 1, mRNA [NM_025137]	A_23_P65699	0.00717	0.04	2.013	1.009
SELS	Homo sapiens selenoprotein S (SELS), transcript variant 2, mRNA [NM_018445]	A_33_P3251289	0.00717	0.04	2.052	1.037
SPAM1	Homo sapiens sperm adhesion molecule 1 (PH-20 hyaluronidase, zona pellucida binding) (SPAM1), transcript variant 3, mRNA [NM_001174044]	A_33_P3377609	0.00716	0.04	3.541	1.824
STYK1	Homo sapiens serine/threonine/tyrosine kinase 1 (STYK1), mRNA [NM_018423]	A_23_P13822	0.00723	0.0402	3.986	1.995
PHEX	Homo sapiens phosphate regulating endopeptidase homolog, X-linked (PHEX), mRNA [NM_000444]	A_23_P114084	0.00725	0.0403	2.562	1.357
C17orf78	Homo sapiens chromosome 17 open reading frame 78 (C17orf78), mRNA [NM_173625]	A_23_P335388	0.00729	0.0404	2.185	1.128
IFIT1	Homo sapiens interferon-induced protein with tetratricopeptide repeats 1 (IFIT1), transcript variant 2, mRNA [NM_001548]	A_23_P52266	0.00728	0.0404	18.826	4.235
DOPEY2	Homo sapiens dopey family member 2 (DOPEY2), mRNA [NM_005128]	A_23_P253586	0.00736	0.0407	2.943	1.557
BBS12	Homo sapiens Bardet-Biedl syndrome 12 (BBS12), transcript variant 2, mRNA [NM_152618]	A_32_P235796	0.00736	0.0407	2.378	1.25
FAM134B	Homo sapiens family with sequence similarity 134, member B (FAM134B), transcript variant 1, mRNA [NM_001034850]	A_23_P167599	0.00741	0.0408	8.337	3.06
HPS3	Homo sapiens Hermansky-Pudlak syndrome 3 (HPS3), mRNA [NM_032383]	A_23_P40821	0.00743	0.0409	2.34	1.227

MYD88	Homo sapiens myeloid differentiation primary response gene (88) (MYD88), transcript variant 2, mRNA [NM_002468]	A_23_P362659	0.00746	0.041	2.331	1.221
GBP2	Homo sapiens guanylate binding protein 2, interferon-inducible (GBP2), mRNA [NM_004120]	A_23_P85693	0.00746	0.041	11.274	3.495
THC2714081	Unknown	A_33_P3296220	0.00757	0.0414	2.352	1.234
EPB41L4A	Homo sapiens erythrocyte membrane protein band 4.1 like 4A (EPB41L4A), mRNA [NM_022140]	A_33_P3321697	0.0076	0.0415	6.046	2.596
LHX4	Homo sapiens LIM homeobox 4 (LHX4), mRNA [NM_033343]	A_23_P23869	0.00766	0.0417	3.835	1.939
LONRF1	Homo sapiens LON peptidase N-terminal domain and ring finger 1 (LONRF1), mRNA [NM_152271]	A_23_P94216	0.00766	0.0417	3.316	1.729
A_33_P3269328	Unknown	A_33_P3269328	0.00766	0.0417	4.12	2.043
GCC2	Homo sapiens GRIP and coiled-coil domain containing 2 (GCC2), transcript variant 1, mRNA [NM_181453]	A_24_P85942	0.00779	0.0422	2.061	1.043
PLXNB1	Homo sapiens plexin B1 (PLXNB1), transcript variant 1, mRNA [NM_002673]	A_23_P57961	0.00783	0.0423	2.576	1.365
LOC100133319	Homo sapiens PRO1804 mRNA, complete cds. [AF132201]	A_33_P3398074	0.00787	0.0424	2.853	1.512
CEACAM1	Homo sapiens carcinoembryonic antigen-related cell adhesion molecule 1 (biliary glycoprotein) (CEACAM1), transcript variant 1, mRNA [NM_001712]	A_24_P382319	0.00786	0.0424	19.319	4.272
ZNF655	Homo sapiens zinc finger protein 655 (ZNF655), transcript variant 2, mRNA [NM_024061]	A_23_P215819	0.00794	0.0427	2.058	1.041
STOX2	Homo sapiens storkhead box 2 (STOX2), mRNA [NM_020225]	A_24_P163237	0.00798	0.0428	2.218	1.149
CEBPA	Homo sapiens CCAAT/enhancer binding protein (C/EBP), alpha (CEBPA), mRNA [NM_004364]	A_24_P224727	0.00808	0.0431	3.218	1.686
CGA	Homo sapiens glycoprotein hormones, alpha polypeptide (CGA), transcript variant 2, mRNA [NM_000735]	A_23_P42386	0.00812	0.0433	3.579	1.839
HADH	Homo sapiens hydroxyacyl-CoA dehydrogenase (HADH), nuclear gene encoding mitochondrial protein, transcript variant 2, mRNA [NM_005327]	A_23_P167227	0.00814	0.0433	2.028	1.02
PCOTH	Homo sapiens C1QTNF9B antisense RNA 1 (non-protein coding) (C1QTNF9B-AS1), transcript variant 1, mRNA [NM_001014442]	A_33_P3531206	0.00812	0.0433	4.071	2.026
LOC729680	Homo sapiens cDNA FLJ33345 fis, clone BRACE2003713. [AK090664]	A_32_P46840	0.00823	0.0436	2.169	1.117
TUBB4	Homo sapiens tubulin, beta 4A class IVa (TUBB4A), mRNA [NM_006087]	A_23_P113656	0.00821	0.0436	3.447	1.785
RUNX1	Homo sapiens runt-related transcription factor 1 (RUNX1), transcript variant 2, mRNA [NM_001001890]	A_33_P3211818	0.00826	0.0437	2.209	1.143
GAS2L3	Homo sapiens growth arrest-specific 2 like 3 (GAS2L3), mRNA [NM_174942]	A_32_P189204	0.00824	0.0437	2.999	1.585
OPTN	Homo sapiens optineurin (OPTN), transcript variant 1, mRNA [NM_001008211]	A_33_P3333317	0.00831	0.0439	3.126	1.644
LOC100133008	Unknown	A_33_P3370521	0.00834	0.044	2.419	1.274
PAQR5	Homo sapiens progestin and adipoQ receptor family member V (PAQR5), transcript variant 1, mRNA [NM_001104554]	A_33_P3368750	0.00838	0.0441	6.922	2.791
TMEM45A	Homo sapiens transmembrane protein 45A (TMEM45A), mRNA [NM_018004]	A_33_P3344831	0.00841	0.0442	3.972	1.99
HBD	Homo sapiens hemoglobin, delta (HBD), mRNA [NM_000519]	A_24_P75190	0.00846	0.0443	3.356	1.747
DBP	Homo sapiens D site of albumin promoter (albumin D-box) binding protein (DBP), mRNA [NM_001352]	A_23_P130753	0.00857	0.0447	2.395	1.26
PIWIL4	Homo sapiens piwi-like 4 (Drosophila) (PIWIL4), mRNA [NM_152431]	A_23_P427760	0.00867	0.045	4.829	2.272
RALB	Homo sapiens v-ral simian leukemia viral oncogene homolog B (ras related; GTP binding protein) (RALB), mRNA [NM_002881]	A_33_P3356502	0.00867	0.045	2.086	1.061
BCO2	Homo sapiens beta-carotene oxygenase 2 (BCO2), transcript variant 1, mRNA [NM_031938]	A_33_P3888485	0.0087	0.0451	2.304	1.204
IRAK3	Homo sapiens interleukin-1 receptor-associated kinase 3 (IRAK3), transcript variant 1, mRNA [NM_007199]	A_23_P162300	0.00868	0.0451	3.234	1.693
FAM59A	Homo sapiens family with sequence similarity 59, member A (FAM59A), transcript variant 2, mRNA [NM_022751]	A_23_P66948	0.00874	0.0452	4.222	2.078
ENST00000378941	glycerol kinase [Source:HGNC Symbol;Acc:4289] [ENST00000378941]	A_33_P3376273	0.00875	0.0453	2.231	1.158
SLC30A1	Homo sapiens solute carrier family 30 (zinc transporter), member 1 (SLC30A1), mRNA [NM_021194]	A_23_P23815	0.0088	0.0454	2.049	1.035
SNX18	Homo sapiens sorting nexin 18 (SNX18), transcript variant 2, mRNA [NM_052870]	A_33_P3250128	0.00883	0.0455	2.666	1.415
LYPLA1	Homo sapiens lysophospholipase I (LYPLA1), mRNA [NM_006330]	A_23_P214091	0.00885	0.0456	2.07	1.05
FAM90A7	Homo sapiens family with sequence similarity 90, member A7 (FAM90A7), mRNA [NM_001136572]	A_24_P515319	0.00885	0.0456	3.405	1.768
ENST00000404616	Unknown	A_33_P3366550	0.00886	0.0456	2.419	1.274
MYO1A	Homo sapiens myosin IA (MYO1A), mRNA [NM_005379]	A_23_P162288	0.00888	0.0457	2.589	1.372

NFAM1	Homo sapiens NFAT activating protein with ITAM motif 1 (NFAM1), mRNA [NM_145912]	A_33_P3292769	0.0089	0.0457	3.734	1.901
TNPO3	Homo sapiens transportin 3 (TNPO3), transcript variant 1, mRNA [NM_012470]	A_33_P3370132	0.00887	0.0457	2.327	1.219
SH2D1B	Homo sapiens SH2 domain containing 1B (SH2D1B), mRNA [NM_053282]	A_23_P351148	0.00897	0.046	7.172	2.842
INPP1	Homo sapiens inositol polyphosphate-1-phosphatase (INPP1), transcript variant 2, mRNA [NM_002194]	A_32_P44453	0.00899	0.0461	11.69	3.547
INTU	Homo sapiens inturned planar cell polarity effector homolog (Drosophila) (INTU), mRNA [NM_015693]	A_32_P118372	0.00903	0.0462	2.347	1.231
TMEM119	Homo sapiens transmembrane protein 119 (TMEM119), mRNA [NM_181724]	A_33_P3395605	0.00902	0.0462	2.813	1.492
KIRREL3	Homo sapiens kin of IRRE like 3 (Drosophila) (KIRREL3), transcript variant 1, mRNA [NM_032531]	A_23_P104741	0.00911	0.0465	9.491	3.247
LOC100130363	BJ995728 human hepatoblastoma cDNA Homo sapiens cDNA clone hmft-3130 5', mRNA sequence [BJ995728]	A_33_P3413224	0.00912	0.0465	2.249	1.169
ENST00000331596	Synthetic construct DNA, clone: pF1KE0813, Homo sapiens OLFR1110 gene for olfactory receptor 1110, without stop codon, in Flexi system. [AB529247]	A_23_P127697	0.00914	0.0466	3.124	1.643
UPP2	Homo sapiens uridine phosphorylase 2 (UPP2), transcript variant 1, mRNA [NM_173355]	A_23_P357316	0.00923	0.0469	2.947	1.559
CXorf27	Homo sapiens chromosome X open reading frame 27 (CXorf27), mRNA [NM_012274]	A_24_P281872	0.00922	0.0469	4.572	2.193
LOC100130943	Unknown	A_33_P3405474	0.00923	0.0469	3.714	1.893
TFF3	Homo sapiens trefoil factor 3 (intestinal) (TFF3), mRNA [NM_003226]	A_33_P3334305	0.00931	0.0471	2.579	1.367
RAB44	Homo sapiens cDNA FLJ43093 fis, clone CORDB1000140. [AK125083]	A_33_P3349912	0.00936	0.0473	2.86	1.516
PRDX2	Homo sapiens peroxiredoxin 2 (PRDX2), nuclear gene encoding mitochondrial protein, transcript variant 3, mRNA [NM_181738]	A_24_P168416	0.00942	0.0475	2.338	1.225
MEGF9	Homo sapiens multiple EGF-like-domains 9 (MEGF9), mRNA [NM_001080497]	A_32_P129894	0.00945	0.0476	3.309	1.727
MYCBPAP	Homo sapiens MYCBP associated protein (MYCBPAP), mRNA [NM_032133]	A_33_P3361388	0.00946	0.0477	2.524	1.336
IRS2	Homo sapiens insulin receptor substrate 2 (IRS2), mRNA [NM_003749]	A_24_P154037	0.00946	0.0477	2.156	1.108
E2F7	Homo sapiens E2F transcription factor 7 (E2F7), mRNA [NM_203394]	A_32_P210202	0.0095	0.0478	2.039	1.028
PTGS2	Homo sapiens prostaglandin-endoperoxide synthase 2 (prostaglandin G/H synthase and cyclooxygenase) (PTGS2), mRNA [NM_000963]	A_24_P250922	0.00951	0.0478	6.324	2.661
PTGES	Homo sapiens prostaglandin E synthase (PTGES), mRNA [NM_004878]	A_24_P403417	0.00949	0.0478	3.318	1.73
CA6	Homo sapiens carbonic anhydrase VI (CA6), mRNA [NM_001215]	A_24_P376760	0.00949	0.0478	4.115	2.041
KIAA1841	Homo sapiens KIAA1841 (KIAA1841), transcript variant 1, mRNA [NM_001129993]	A_32_P149298	0.00953	0.0479	2.691	1.428
ATF3	Homo sapiens activating transcription factor 3 (ATF3), transcript variant 4, mRNA [NM_001040619]	A_23_P34915	0.00957	0.048	3.083	1.625
GM2A	Homo sapiens GM2 ganglioside activator (GM2A), transcript variant 1, mRNA [NM_000405]	A_24_P925314	0.00962	0.0482	2.233	1.159
OR8G5	Homo sapiens olfactory receptor, family 8, subfamily G, member 5 (OR8G5), mRNA [NM_001005198]	A_33_P3262094	0.00965	0.0482	5.392	2.431
CLGN	Homo sapiens calmegin (CLGN), transcript variant 1, mRNA [NM_004362]	A_23_P18684	0.00967	0.0483	13.456	3.75
RTN1	Homo sapiens reticulon 1 (RTN1), transcript variant 1, mRNA [NM_021136]	A_23_P140290	0.00967	0.0483	4.313	2.109
ODZ2	Homo sapiens odz, odd Oz/ten-m homolog 2 (Drosophila) (ODZ2), mRNA [NM_001122679]	A_24_P299474	0.0097	0.0484	5.572	2.478
ZC3H12B	Homo sapiens zinc finger CCCH-type containing 12B (ZC3H12B), mRNA [NM_001010888]	A_33_P3224735	0.00971	0.0484	9.766	3.288
FOXG1	Homo sapiens forkhead box G1 (FOXG1), mRNA [NM_005249]	A_23_P205428	0.00975	0.0485	2.576	1.365
AKR1C3	Homo sapiens aldo-keto reductase family 1, member C3 (3-alpha hydroxysteroid dehydrogenase, type II) (AKR1C3), mRNA [NM_003739]	A_23_P138541	0.00974	0.0485	9.789	3.291
XPO6	Homo sapiens exportin 6 (XPO6), mRNA [NM_015171]	A_23_P325438	0.00979	0.0487	2.177	1.122
SNAPC5	Homo sapiens small nuclear RNA activating complex, polypeptide 5, 19kDa (SNAPC5), mRNA [NM_006049]	A_23_P383977	0.00985	0.0488	2.212	1.146
B4GALT1	Homo sapiens UDP-Gal:betaGlcNAc beta 1,4- galactosyltransferase, polypeptide 1 (B4GALT1), mRNA [NM_001497]	A_23_P135271	0.00982	0.0488	2.142	1.099
WDFY3	WD repeat and FYVE domain containing 3 [Source:HGNC Symbol;Acc:20751] [ENST00000426414]	A_24_P83379	0.01	0.0493	2.392	1.258
ARSG	Homo sapiens arylsulfatase G (ARSG), mRNA [NM_014960]	A_24_P339416	0.0101	0.0496	3.981	1.993
RASGRP2	Homo sapiens RAS guanyl releasing protein 2 (calcium and DAG-regulated) (RASGRP2), transcript variant 2, mRNA [NM_153819]	A_23_P64058	0.0101	0.0496	2.786	1.478

KCTD1	Homo sapiens potassium channel tetramerisation domain containing 1 (KCTD1), transcript variant 2, mRNA [NM_198991]	A_23_P130343	0.0102	0.0499	4.67	2.223
