# Supplementary material for: CEBPA-regulated lncRNAs, new players in the study of acute myeloid leukemia
Source: J Hematol Oncol. 2014 Sep 25;7:69. doi: 10.1186/s13045-014-0069-1 (PMC4177583; doi:10.1186/s13045-014-0069-1)
Supplement: Additional file 4: Table S2. — CEBPA-regulated mRNAs with significant differential expression (absolute fold change ≥ 2 and adjusted P value ≤ 0.05) identified in K562. (A) Up-regulated mRNAs. (B) Down-regulated mcRNAs. [file 13045_2014_69_MOESM4_ESM.zip › 13045_2014_69_add1/13045_2014_69_add8.pdf]

Supplementary Table S2B. CEBPA-downregulated coding genes						
Name	Description	ProbeID	p.value	Adj.p.value	Fold_Change	Log2_Ratio
SERPINF1	Homo sapiens serpin peptidase inhibitor, clade F (alpha-2 antiplasmin, pigment epithelium derived factor), member 1 (SERPINF1), mRNA [NM_002615]	A_23_P100660	1.18E-07	0.0000862	-3.934	-1.976
PCYOX1L	Homo sapiens prenylcysteine oxidase 1 like (PCYOX1L), mRNA [NM_024028]	A_23_P30275	1.79E-07	0.000113	-4.588	-2.198
IKZF4	Homo sapiens IKAROS family zinc finger 4 (Eos) (IKZF4), mRNA [NM_022465]	A_23_P378288	3.4E-07	0.000158	-4.988	-2.318
DECR2	Homo sapiens 2,4-dienoyl CoA reductase 2, peroxisomal (DECR2), mRNA [NM_020664]	A_23_P100315	3.63E-07	0.000163	-2.365	-1.242
ACCN2	Homo sapiens amiloride-sensitive cation channel 2, neuronal (ACCN2), transcript variant 1, mRNA [NM_020039]	A_23_P204751	3.8E-07	0.000167	-12.036	-3.589
TMIGD2	Homo sapiens transmembrane and immunoglobulin domain containing 2 (TMIGD2), transcript variant 1, mRNA [NM_144615]	A_33_P3415698	4.47E-07	0.00018	-3.506	-1.81
CST3	Homo sapiens cystatin C (CST3), mRNA [NM_000099]	A_33_P3228266	5.35E-07	0.000191	-2.096	-1.067
SLC30A3	Homo sapiens solute carrier family 30 (zinc transporter), member 3 (SLC30A3), mRNA [NM_003459]	A_23_P302568	6.14E-07	0.000199	-4.915	-2.297
RASA4	Homo sapiens RAS p21 protein activator 4 (RASA4), transcript variant 1, mRNA [NM_006989]	A_33_P3342443	6.97E-07	0.000215	-6.034	-2.593
DOT1L	Homo sapiens DOT1-like, histone H3 methyltransferase (S. cerevisiae) (DOT1L), mRNA [NM_032482]	A_23_P101737	1.07E-06	0.000258	-1.987	-0.991
ZNF33A	Homo sapiens zinc finger protein 33A (ZNF33A), transcript variant 1, mRNA [NM_006954]	A_33_P3238196	1.23E-06	0.00028	-2.443	-1.289
DGAT1	Homo sapiens diacylglycerol O-acyltransferase 1 (DGAT1), mRNA [NM_012079]	A_23_P112162	1.4E-06	0.000303	-1.879	-0.91
C9orf7	Homo sapiens chromosome 9 open reading frame 7 (C9orf7), transcript variant 3, mRNA [NM_001242369]	A_33_P3218625	1.53E-06	0.000315	-2.985	-1.578
NPM2	Homo sapiens nucleophosmin/nucleoplasmin 2 (NPM2), mRNA [NM_182795]	A_33_P3272479	1.61E-06	0.000326	-2.519	-1.333
ATP4A	Homo sapiens ATPase, H+/K+ exchanging, alpha polypeptide (ATP4A), mRNA [NM_000704]	A_23_P430728	1.65E-06	0.000328	-7.934	-2.988
ACVRL1	Homo sapiens activin A receptor type II-like 1 (ACVRL1), transcript variant 1, mRNA [NM_000020]	A_24_P945113	1.64E-06	0.000328	-7.61	-2.928
PHF21B	Homo sapiens PHD finger protein 21B (PHF21B), transcript variant 1, mRNA [NM_138415]	A_33_P3280666	1.88E-06	0.000358	-2.052	-1.037
BTBD12	Homo sapiens SLX4 structure-specific endonuclease subunit homolog (S. cerevisiae) (SLX4), mRNA [NM_032444]	A_23_P371129	2.13E-06	0.000392	-2.202	-1.139
PPAPDC3	Homo sapiens phosphatidic acid phosphatase type 2 domain containing 3 (PPAPDC3), mRNA [NM_032728]	A_23_P157736	2.29E-06	0.000397	-6.628	-2.729
TDRKH	Homo sapiens tudor and KH domain containing (TDRKH), transcript variant 3, mRNA [NM_006862]	A_23_P46351	2.34E-06	0.000402	-2.897	-1.535
PLXNB2	Homo sapiens plexin B2 (PLXNB2), mRNA [NM_012401]	A_24_P70888	2.46E-06	0.000407	-3.57	-1.836
PLCB2	Homo sapiens phospholipase C, beta 2 (PLCB2), mRNA [NM_004573]	A_33_P3260614	2.54E-06	0.000413	-3.853	-1.946
LHPP	Homo sapiens phospholysine phosphohistidine inorganic pyrophosphate phosphatase (LHPP), transcript variant 1, mRNA [NM_022126]	A_23_P75299	2.62E-06	0.000423	-4.433	-2.148

FAM83A	Homo sapiens family with sequence similarity 83, member A (FAM83A), transcript variant 1, mRNA [NM_032899]	A_23_P216052	2.65E-06	0.000423	-5.688	-2.508
RCOR3	Homo sapiens REST corepressor 3 (RCOR3), transcript variant 4, mRNA [NM_018254]	A_32_P167122	2.74E-06	0.000425	-2.366	-1.242
C1orf167	chromosome 1 open reading frame 167 [Source:HGNC Symbol;Acc:25262] [ENST00000433342]	A_24_P306594	2.75E-06	0.000425	-18.738	-4.228
LOC728295	Unknown	A_24_P778906	3.03E-06	0.000444	-7.108	-2.829
PRRT2	Homo sapiens proline-rich transmembrane protein 2 (PRRT2), mRNA [NM_145239]	A_23_P66017	3.22E-06	0.000461	-7.954	-2.992
CCDC14	Homo sapiens coiled-coil domain containing 14 (CCDC14), mRNA [NM_022757]	A_23_P384056	3.24E-06	0.000463	-1.891	-0.919
AHNAK	Homo sapiens AHNAK nucleoprotein (AHNAK), transcript variant 1, mRNA [NM_001620]	A_24_P943393	3.31E-06	0.000469	-5.942	-2.571
CGREF1	Homo sapiens cell growth regulator with EF-hand domain 1 (CGREF1), transcript variant 1, mRNA [NM_006569]	A_33_P3227793	3.4E-06	0.000476	-3.723	-1.896
GSTM3	Homo sapiens glutathione S-transferase mu 3 (brain) (GSTM3), transcript variant 1, mRNA [NM_000849]	A_23_P12343	3.51E-06	0.000483	-5.264	-2.396
PBX4	Homo sapiens pre-B-cell leukemia homeobox 4 (PBX4), transcript variant 1, mRNA [NM_025245]	A_23_P90419	3.51E-06	0.000483	-2.757	-1.463
MESP2	Homo sapiens mesoderm posterior 2 homolog (mouse) (MESP2), mRNA [NM_001039958]	A_33_P3214463	3.54E-06	0.000486	-2.581	-1.368
ASMTL	Homo sapiens acetylserotonin O-methyltransferase-like (ASMTL), transcript variant 1, mRNA [NM_004192]	A_23_P159539	3.61E-06	0.000493	-3.643	-1.865
LEPREL2	Homo sapiens leprecan-like 2 (LEPREL2), mRNA [NM_014262]	A_33_P3507542	3.83E-06	0.000511	-4.104	-2.037
WDR66	Homo sapiens WD repeat domain 66 (WDR66), transcript variant 1, mRNA [NM_144668]	A_23_P363275	4.66E-06	0.000576	-4.708	-2.235
ASAP3	Homo sapiens ArfGAP with SH3 domain, ankyrin repeat and PH domain 3 (ASAP3), transcript variant 1, mRNA [NM_017707]	A_23_P114689	4.82E-06	0.000587	-3.134	-1.648
C1orf100	Homo sapiens chromosome 1 open reading frame 100 (C1orf100), mRNA [NM_001012970]	A_32_P8925	4.9E-06	0.00059	-10.772	-3.429
DNMT3A	Homo sapiens DNA (cytosine-5-)-methyltransferase 3 alpha (DNMT3A), transcript variant 1, mRNA [NM_175629]	A_23_P154500	4.88E-06	0.00059	-2.771	-1.47
C10orf125	Homo sapiens chromosome 10 open reading frame 125 (C10orf125), transcript variant 2, mRNA [NM_198472]	A_24_P186379	4.97E-06	0.000595	-2.158	-1.109
A_19_P0080006‘	Unknown	A_19_P00800061	5.27E-06	0.000615	-10.685	-3.418
QPRT	Homo sapiens quinolinate phosphoribosyltransferase (QPRT), mRNA [NM_014298]	A_23_P34233	5.61E-06	0.000639	-2.421	-1.276
MECP2	Homo sapiens methyl CpG binding protein 2 (Rett syndrome) (MECP2), transcript variant 1, mRNA [NM_004992]	A_24_P237486	5.87E-06	0.000654	-2.205	-1.14
NUDT14	Homo sapiens nudix (nucleoside diphosphate linked moiety X)-type motif 14 (NUDT14), mRNA [NM_177533]	A_23_P414978	6.23E-06	0.000683	-3.799	-1.926
PDE4DIP	Homo sapiens phosphodiesterase 4D interacting protein (PDE4DIP), transcript variant 9, mRNA [NM_001198834]	A_33_P3272090	6.28E-06	0.000687	-2.437	-1.285
BRD3	Homo sapiens bromodomain containing 3 (BRD3), mRNA [NM_007371]	A_23_P216689	6.45E-06	0.000696	-2.232	-1.158
JUP	Homo sapiens junction plakoglobin (JUP), transcript variant 1, mRNA [NM_002230]	A_23_P501822	6.81E-06	0.000721	-2.224	-1.153

CCDC8	Homo sapiens coiled-coil domain containing 8 (CCDC8), mRNA [NM_032040]	A_33_P3402020	6.96E-06	0.000732	-2.224	-1.153
KANK3	Homo sapiens KN motif and ankyrin repeat domains 3 (KANK3), mRNA [NM_198471]	A_23_P131089	7.26E-06	0.000744	-4.478	-2.163
ART5	Homo sapiens ADP-ribosyltransferase 5 (ART5), transcript variant 1, mRNA [NM_053017]	A_23_P427122	7.39E-06	0.000746	-5.462	-2.449
GALK1	Homo sapiens galactokinase 1 (GALK1), mRNA [NM_000154]	A_23_P141126	7.34E-06	0.000746	-2.07	-1.05
LOC389831	Homo sapiens uncharacterized LOC389831 (LOC389831), mRNA [NM_001242480]	A_33_P3402056	7.38E-06	0.000746	-2.086	-1.061
SSPO	Homo sapiens SCO-spondin homolog (Bos taurus) (SSPO), mRNA [NM_198455]	A_23_P353048	7.44E-06	0.000747	-10.181	-3.348
FAM43B	Homo sapiens family with sequence similarity 43, member B (FAM43B), mRNA [NM_207334]	A_32_P125338	7.44E-06	0.000747	-7.977	-2.996
GGN	Homo sapiens gametogenetin (GGN), mRNA [NM_152657]	A_33_P3329984	7.41E-06	0.000747	-5.477	-2.453
CRABP2	Homo sapiens cellular retinoic acid binding protein 2 (CRABP2), transcript variant 1, mRNA [NM_001878]	A_23_P115064	7.62E-06	0.000756	-4.682	-2.227
KIAA0895L	Homo sapiens KIAA0895-like (KIAA0895L), mRNA [NM_001040715]	A_24_P331655	7.68E-06	0.000759	-2.72	-1.444
CALHM3	Homo sapiens calcium homeostasis modulator 3 (CALHM3), mRNA [NM_001129742]	A_33_P3241204	7.94E-06	0.000781	-5.788	-2.533
DHDH	Homo sapiens dihydrodiol dehydrogenase (dimeric) (DHDH), mRNA [NM_014475]	A_23_P67367	8.16E-06	0.000795	-3.145	-1.653
AF461897	Homo sapiens FP15331 mRNA, complete cds. [AF461897]	A_33_P3236686	8.22E-06	0.000798	-2.638	-1.399
IQSEC2	Homo sapiens IQ motif and Sec7 domain 2 (IQSEC2), transcript variant 2, mRNA [NM_015075]	A_23_P330788	8.6E-06	0.00082	-2.79	-1.48
RNF157	Homo sapiens ring finger protein 157 (RNF157), mRNA [NM_052916]	A_32_P57810	8.72E-06	0.000825	-3.625	-1.858
ZMIZ1	Homo sapiens zinc finger, MIZ-type containing 1 (ZMIZ1), mRNA [NM_020338]	A_23_P138435	9.04E-06	0.000844	-3.181	-1.67
HPN	Homo sapiens hepsin (HPN), transcript variant 1, mRNA [NM_182983]	A_23_P406782	9.28E-06	0.000854	-8.79	-3.136
EPB41L1	Homo sapiens erythrocyte membrane protein band 4.1-like 1 (EPB41L1), transcript variant 2, mRNA [NM_177996]	A_33_P3228510	9.24E-06	0.000854	-2.874	-1.523
TTC5	Homo sapiens tetratricopeptide repeat domain 5 (TTC5), mRNA [NM_138376]	A_33_P3347108	9.33E-06	0.000857	-2.132	-1.092
MZF1	Homo sapiens myeloid zinc finger 1 (MZF1), transcript variant 2, mRNA [NM_198055]	A_33_P3243168	9.69E-06	0.000877	-2.239	-1.163
C3orf21	Homo sapiens xyloside xylosyltransferase 1 (XXYLT1), mRNA [NM_152531]	A_33_P3221438	9.92E-06	0.000889	-3.34	-1.74
TBXA2R	Homo sapiens thromboxane A2 receptor (TBXA2R), transcript variant a, mRNA [NM_001060]	A_23_P90357	1.03E-05	0.000899	-5.367	-2.424
PIH1D1	Homo sapiens PIH1 domain containing 1 (PIH1D1), mRNA [NM_017916]	A_33_P3255409	1.02E-05	0.000899	-2.362	-1.24
VWA1	Homo sapiens von Willebrand factor A domain containing 1 (VWA1), transcript variant 1, mRNA [NM_022834]	A_33_P3395743	1.02E-05	0.000899	-4.855	-2.279
ANTXR1	Homo sapiens anthrax toxin receptor 1 (ANTXR1), transcript variant 2, mRNA [NM_053034]	A_23_P84576	1.04E-05	0.000901	-4.663	-2.221
ZNF541	Homo sapiens zinc finger protein 541 (ZNF541), mRNA [NM_001101419]	A_23_P50517	1.05E-05	0.000904	-5.622	-2.491
ZNF219	Homo sapiens zinc finger protein 219 (ZNF219), transcript variant 1, mRNA [NM_016423]	A_24_P35891	1.07E-05	0.000913	-2.521	-1.334

PRPF40B	Homo sapiens PRP40 pre-mRNA processing factor 40 homolog B (S. cerevisiae) (PRPF40B), transcript variant 1, mRNA [NM_001031698]	A_33_P3311205	1.09E-05	0.000919	-2.099	-1.069
LLGL2	Homo sapiens lethal giant larvae homolog 2 (Drosophila) (LLGL2), transcript variant 3, mRNA [NM_001031803]	A_33_P3372074	0.000011	0.000923	-2.536	-1.342
LOC440330	Homo sapiens, clone IMAGE:3543963, mRNA. [BC004968]	A_32_P351277	1.18E-05	0.00095	-3.784	-1.92
AEBP1	Homo sapiens AE binding protein 1 (AEBP1), mRNA [NM_001129]	A_23_P157299	1.19E-05	0.00095	-6.752	-2.755
KRT18P42	Unknown	A_24_P84970	1.23E-05	0.000973	-2.177	-1.123
RTN2	Homo sapiens reticulon 2 (RTN2), transcript variant 1, mRNA [NM_005619]	A_23_P89902	1.25E-05	0.000984	-3.171	-1.665
SLC4A3	Homo sapiens solute carrier family 4, anion exchanger, member 3 (SLC4A3), transcript variant 1, mRNA [NM_005070]	A_23_P39647	1.26E-05	0.000987	-3.092	-1.629
PSRC1	Homo sapiens proline/serine-rich coiled-coil 1 (PSRC1), transcript variant 1, mRNA [NM_032636]	A_23_P46539	1.32E-05	0.00102	-2.779	-1.475
ZBTB3	Homo sapiens zinc finger and BTB domain containing 3 (ZBTB3), mRNA [NM_024784]	A_23_P430181	1.34E-05	0.00102	-2.09	-1.063
INF2	Homo sapiens inverted formin, FH2 and WH2 domain containing (INF2), transcript variant 1, mRNA [NM_022489]	A_23_P394836	1.38E-05	0.00105	-3.125	-1.644
CHEK2	Homo sapiens CHK2 checkpoint homolog (S. pombe) (CHEK2), transcript variant 3, mRNA [NM_001005735]	A_21_P0011475	1.39E-05	0.00105	-1.614	-0.691
BNIP3L	Homo sapiens BCL2/adenovirus E1B 19kDa interacting protein 3-like (BNIP3L), mRNA [NM_004331]	A_33_P3396951	1.45E-05	0.00108	-3.123	-1.643
VEZT	Homo sapiens vezatin, adherens junctions transmembrane protein (VEZT), transcript variant 1, mRNA [NM_017599]	A_23_P204609	1.47E-05	0.00109	-1.593	-0.672
PPIP5K1	Homo sapiens diphosphoinositol pentakisphosphate kinase 1 (PPIP5K1), transcript variant 5, mRNA [NM_001130858]	A_33_P3420020	1.49E-05	0.0011	-2.976	-1.573
C14orf65	Homo sapiens coiled-coil domain containing 85C (CCDC85C), mRNA [NM_001144995]	A_23_P37391	1.52E-05	0.00111	-4.046	-2.017
ENST000002886	Homo sapiens family with sequence similarity 59, member B (FAM59B), transcript variant 2, mRNA [NM_001191033]	A_23_P325924	1.58E-05	0.00113	-2.106	-1.074
LAMB2	Homo sapiens laminin, beta 2 (laminin S) (LAMB2), mRNA [NM_002292]	A_33_P3223780	1.57E-05	0.00113	-5.019	-2.327
HS6ST1	Homo sapiens heparan sulfate 6-O-sulfotransferase 1 (HS6ST1), mRNA [NM_004807]	A_24_P8220	1.63E-05	0.00114	-2.707	-1.437
C2orf77	Homo sapiens chromosome 2 open reading frame 77 (C2orf77), mRNA [NM_001085447]	A_33_P3238636	1.62E-05	0.00114	-7.57	-2.92
CEP250	Homo sapiens centrosomal protein 250kDa (CEP250), mRNA [NM_007186]	A_23_P102832	1.83E-05	0.00122	-2.051	-1.036
MAST1	Homo sapiens microtubule associated serine/threonine kinase 1 (MAST1), mRNA [NM_014975]	A_23_P101480	1.83E-05	0.00122	-2.354	-1.235
CEP68	centrosomal protein 68kDa [Source:HGNC Symbol;Acc:29076] [ENST00000537589]	A_33_P3265224	1.82E-05	0.00122	-2.06	-1.042
PPM1N	Homo sapiens protein phosphatase, Mg2+/Mn2+ dependent, 1N (putative) (PPM1N), mRNA [NM_001080401]	A_33_P3279681	1.84E-05	0.00123	-5.249	-2.392
CELSR2	Homo sapiens cadherin, EGF LAG seven-pass G-type receptor 2 (flamingo homolog, Drosophila) (CELSR2), mRNA [NM_001408]	A_24_P182620	1.85E-05	0.00123	-3.155	-1.658
FBF1	Homo sapiens Fas (TNFRSF6) binding factor 1 (FBF1), mRNA [NM_001080542]	A_24_P87458	1.88E-05	0.00124	-4.064	-2.023
SPIRE2	Homo sapiens spire homolog 2 (Drosophila) (SPIRE2), mRNA [NM_032451]	A_33_P3210203	1.89E-05	0.00124	-3.054	-1.611

SOCS1	Homo sapiens suppressor of cytokine signaling 1 (SOCS1), mRNA [NM_003745]	A_23_P420196	1.94E-05	0.00126	-3.71	-1.891
AHNAK2	Homo sapiens AHNAK nucleoprotein 2 (AHNAK2), mRNA [NM_138420]	A_24_P273253	1.98E-05	0.00128	-2.945	-1.558
MUC1	Homo sapiens mucin 1, cell surface associated (MUC1), transcript variant 1, mRNA [NM_002456]	A_23_P137856	1.99E-05	0.00129	-4.103	-2.037
ENST000003784	chromosome 16 open reading frame 85 [Source:HGNC Symbol;Acc:33799] [ENST00000378416]	A_33_P3398111	2.03E-05	0.0013	-3.467	-1.794
RALGDS	Homo sapiens ral guanine nucleotide dissociation stimulator (RALGDS), transcript variant 2, mRNA [NM_001042368]	A_23_P135184	2.03E-05	0.0013	-2.404	-1.266
IGF2	Homo sapiens insulin-like growth factor 2 (somatomedin A) (IGF2), transcript variant 1, mRNA [NM_000612]	A_23_P150609	2.08E-05	0.00132	-17.28	-4.111
CST5	Homo sapiens cystatin D (CST5), mRNA [NM_001900]	A_23_P170453	2.19E-05	0.00136	-2.065	-1.046
TAL2	Homo sapiens T-cell acute lymphocytic leukemia 2 (TAL2), mRNA [NM_005421]	A_23_P409449	2.24E-05	0.00137	-2.402	-1.264
FAM33A	Homo sapiens spindle and kinetochore associated complex subunit 2 (SKA2), transcript variant 1, mRNA [NM_182620]	A_32_P55860	2.24E-05	0.00137	-2.093	-1.066
BAI2	Homo sapiens brain-specific angiogenesis inhibitor 2 (BAI2), mRNA [NM_001703]	A_33_P3270445	2.23E-05	0.00137	-6.566	-2.715
PERLD1	Homo sapiens post-GPI attachment to proteins 3 (PGAP3), mRNA [NM_033419]	A_24_P275828	0.000023	0.0014	-2.912	-1.542
RNF187	Homo sapiens ring finger protein 187 (RNF187), mRNA [NM_001010858]	A_24_P23995	2.34E-05	0.00141	-2.348	-1.231
KRT18P49	Unknown	A_24_P230057	2.32E-05	0.00141	-2.896	-1.534
SHD	Homo sapiens Src homology 2 domain containing transforming protein D (SHD), mRNA [NM_020209]	A_23_P142255	2.34E-05	0.00141	-4.08	-2.029
LPHN1	Homo sapiens latrophilin 1 (LPHN1), transcript variant 1, mRNA [NM_001008701]	A_23_P391926	2.41E-05	0.00144	-3.627	-1.859
GSTM2	Homo sapiens glutathione S-transferase mu 2 (muscle) (GSTM2), transcript variant 1, mRNA [NM_000848]	A_33_P3410351	2.43E-05	0.00145	-4.027	-2.01
GLOD5	Homo sapiens glyoxalase domain containing 5 (GLOD5), mRNA [NM_001080489]	A_33_P3320563	2.48E-05	0.00147	-7.255	-2.859
ENST000003684	Unknown	A_33_P3237835	2.51E-05	0.00148	-3.893	-1.961
PCDHGB3	Homo sapiens protocadherin gamma subfamily B, 3 (PCDHGB3), transcript variant 2, mRNA [NM_032097]	A_23_P395418	2.54E-05	0.00149	-4.637	-2.213
APC2	Homo sapiens adenomatosis polyposis coli 2 (APC2), mRNA [NM_005883]	A_23_P101905	2.59E-05	0.0015	-4.177	-2.062
EPPK1	Homo sapiens epiplakin 1 (EPPK1), mRNA [NM_031308]	A_24_P357169	2.56E-05	0.0015	-3.041	-1.604
ZC3H12D	Homo sapiens zinc finger CCCH-type containing 12D (ZC3H12D), mRNA [NM_207360]	A_24_P109176	2.58E-05	0.0015	-12.9	-3.689
BCKDHB	Homo sapiens branched chain keto acid dehydrogenase E1, beta polypeptide (BCKDHB), nuclear gene encoding mitochondrial protein, transcript variant 2, mRNA [NM_000056]	A_23_P93464	2.59E-05	0.0015	-2.368	-1.244
CBX7	Homo sapiens chromobox homolog 7 (CBX7), mRNA [NM_175709]	A_23_P250735	2.65E-05	0.00153	-2.008	-1.005
RCOR2	Homo sapiens REST corepressor 2 (RCOR2), mRNA [NM_173587]	A_33_P3211929	2.68E-05	0.00153	-3.949	-1.981
VANGL2	Homo sapiens vang-like 2 (van gogh, Drosophila) (VANGL2), mRNA [NM_020335]	A_33_P3282005	2.69E-05	0.00153	-5.983	-2.581
RDM1	Homo sapiens RAD52 motif 1 (RDM1), transcript variant 2, mRNA [NM_001034836]	A_23_P141447	2.69E-05	0.00153	-4.217	-2.076

FANCL	Homo sapiens Fanconi anemia, complementation group L (FANCL), transcript variant 2, mRNA [NM_018062]	A_23_P131383	2.75E-05	0.00155	-3.077	-1.621
C20orf112	Homo sapiens chromosome 20 open reading frame 112 (C20orf112), mRNA [NM_080616]	A_33_P3271284	2.74E-05	0.00155	-3.146	-1.653
ADAMTS14	Homo sapiens ADAM metallopeptidase with thrombospondin type 1 motif, 14 (ADAMTS14), transcript variant 1, mRNA [NM_139155]	A_24_P275073	0.000028	0.00157	-5.956	-2.574
RSAD2	Homo sapiens radical S-adenosyl methionine domain containing 2 (RSAD2), mRNA [NM_080657]	A_24_P28722	2.88E-05	0.0016	-2.966	-1.569
LOC100131582	Unknown	A_33_P3282988	2.97E-05	0.00163	-6.688	-2.742
FZD2	Homo sapiens frizzled family receptor 2 (FZD2), mRNA [NM_001466]	A_23_P141362	2.98E-05	0.00163	-5.137	-2.361
AGRN	Homo sapiens agrin (AGRN), mRNA [NM_198576]	A_23_P343411	0.00003	0.00164	-3.94	-1.978
NLGN2	Homo sapiens neuroligin 2 (NLGN2), mRNA [NM_020795]	A_23_P207125	3.04E-05	0.00165	-2.413	-1.271
EFNA1	Homo sapiens ephrin-A1 (EFNA1), transcript variant 1, mRNA [NM_004428]	A_23_P113005	3.09E-05	0.00167	-5.812	-2.539
SLC9A3R2	Homo sapiens solute carrier family 9 (sodium/hydrogen exchanger), member 3 regulator 2 (SLC9A3R2), transcript variant 2, mRNA [NM_004785]	A_24_P364838	3.15E-05	0.00168	-3.479	-1.799
CPOX	Homo sapiens coproporphyrinogen oxidase (CPOX), mRNA [NM_000097]	A_33_P3301025	3.15E-05	0.00168	-2.466	-1.302
NRM	Homo sapiens nurim (nuclear envelope membrane protein) (NRM), mRNA [NM_007243]	A_23_P8055	3.18E-05	0.00169	-3.959	-1.985
FLJ40125	Homo sapiens protein phosphatase, Mg2+/Mn2+ dependent, 1N (putative) (PPM1N), mRNA [NM_001080401]	A_23_P141893	3.19E-05	0.0017	-5.199	-2.378
AK123797	Homo sapiens cDNA FLJ41803 fis, clone NHNPC2002749. [AK123797]	A_33_P3353190	3.26E-05	0.00172	-5.835	-2.545
THC2677526	Unknown	A_33_P3380236	3.25E-05	0.00172	-4.403	-2.138
SPTBN4	Homo sapiens spectrin, beta, non-erythrocytic 4 (SPTBN4), transcript variant sigma5, mRNA [NM_025213]	A_24_P238420	3.34E-05	0.00174	-5.427	-2.44
INPP5J	Homo sapiens inositol polyphosphate-5-phosphatase J (INPP5J), mRNA [NM_001002837]	A_24_P284805	3.33E-05	0.00174	-7.346	-2.877
NME3	Homo sapiens non-metastatic cells 3, protein expressed in (NME3), mRNA [NM_002513]	A_23_P152115	3.44E-05	0.00176	-3.142	-1.652
TRIM47	Homo sapiens tripartite motif containing 47 (TRIM47), mRNA [NM_033452]	A_33_P3235053	3.44E-05	0.00176	-5.014	-2.326
ECE2	Homo sapiens endothelin converting enzyme 2 (ECE2), transcript variant 1, mRNA [NM_014693]	A_23_P377299	0.000034	0.00176	-8.199	-3.035
ENST000004152	Unknown	A_33_P3347241	3.46E-05	0.00177	-4.862	-2.282
SCN4A	Homo sapiens sodium channel, voltage-gated, type IV, alpha subunit (SCN4A), mRNA [NM_000334]	A_24_P270999	3.48E-05	0.00177	-10.067	-3.332
GSTO2	Homo sapiens glutathione S-transferase omega 2 (GSTO2), transcript variant 1, mRNA [NM_183239]	A_23_P202206	3.55E-05	0.00179	-5.613	-2.489
SH2D2A	Homo sapiens SH2 domain containing 2A (SH2D2A), transcript variant 2, mRNA [NM_003975]	A_23_P160618	3.52E-05	0.00179	-3.023	-1.596
ENST000003906	immunoglobulin heavy variable 1-46 [Source:HGNC Symbol;Acc:5554] [ENST00000390622]	A_32_P190951	3.61E-05	0.00181	-4.03	-2.011
CNNM3	Homo sapiens cyclin M3 (CNNM3), transcript variant 1, mRNA [NM_017623]	A_33_P3255274	3.69E-05	0.00183	-2.028	-1.02
CASKIN2	Homo sapiens CASK interacting protein 2 (CASKIN2), transcript variant 1, mRNA [NM_020753]	A_23_P44363	3.69E-05	0.00183	-5.669	-2.503
SPTBN2	Homo sapiens spectrin, beta, non-erythrocytic 2 (SPTBN2), mRNA [NM_006946]	A_23_P98282	3.71E-05	0.00184	-2.144	-1.1

MICAL3	Homo sapiens microtubule associated monoxygenase, calponin and LIM domain containing 3 (MICAL3), transcript variant 1, mRNA [NM_015241]	A_24_P366082	3.75E-05	0.00185	-2.651	-1.407
SMO	Homo sapiens smoothened, frizzled family receptor (SMO), mRNA [NM_005631]	A_23_P70818	3.89E-05	0.00188	-5.063	-2.34
MOBKL1A	Homo sapiens MOB kinase activator 1B (MOB1B), transcript variant 2, mRNA [NM_173468]	A_23_P347059	3.89E-05	0.00188	-2.157	-1.109
FAM38A	Homo sapiens piezo-type mechanosensitive ion channel component 1 (PIEZO1), mRNA [NM_001142864]	A_23_P140738	3.96E-05	0.0019	-3.025	-1.597
HERC2	Homo sapiens hect domain and RLD 2 (HERC2), mRNA [NM_004667]	A_21_P0011431	4.02E-05	0.00191	-2.211	-1.145
FAM62A	Homo sapiens extended synaptotagmin-like protein 1 (ESYT1), transcript variant 2, mRNA [NM_015292]	A_23_P150852	4.01E-05	0.00191	-2.827	-1.499
LAMA5	Homo sapiens laminin, alpha 5 (LAMA5), mRNA [NM_005560]	A_23_P109269	4.06E-05	0.00192	-2.744	-1.456
DBNDD2	Homo sapiens dysbindin (dystrobrevin binding protein 1) domain containing 2 (DBNDD2), transcript variant 6, mRNA [NM_001048226]	A_23_P28772	4.06E-05	0.00192	-2.601	-1.379
RFK	Homo sapiens riboflavin kinase (RFK), mRNA [NM_018339]	A_23_P216708	4.07E-05	0.00192	-4.266	-2.093
FAM63A	Homo sapiens family with sequence similarity 63, member A (FAM63A), transcript variant 2, mRNA [NM_001040217]	A_23_P160546	4.08E-05	0.00192	-2.182	-1.125
GPIHBP1	Homo sapiens glycosylphosphatidylinositol anchored high density lipoprotein binding protein 1 (GPIHBP1), mRNA [NM_178172]	A_23_P72697	4.05E-05	0.00192	-2.203	-1.139
TMEM9	Homo sapiens transmembrane protein 9 (TMEM9), mRNA [NM_016456]	A_23_P114952	4.14E-05	0.00194	-2.086	-1.06
GSTM4	Homo sapiens glutathione S-transferase mu 4 (GSTM4), transcript variant 2, mRNA [NM_147148]	A_23_P217917	4.25E-05	0.00197	-2.224	-1.153
TEAD2	Homo sapiens TEA domain family member 2 (TEAD2), mRNA [NM_003598]	A_23_P119344	4.33E-05	0.00199	-4.63	-2.211
OBSL1	Homo sapiens obscurin-like 1 (OBSL1), transcript variant 1, mRNA [NM_015311]	A_33_P3241428	4.36E-05	0.002	-3.503	-1.808
GAPVD1	Homo sapiens GTPase activating protein and VPS9 domains 1 (GAPVD1), mRNA [NM_015635]	A_23_P372660	4.38E-05	0.002	-1.605	-0.682
USH1G	Homo sapiens Usher syndrome 1G (autosomal recessive) (USH1G), mRNA [NM_173477]	A_33_P3401711	4.49E-05	0.00204	-10.439	-3.384
MPP2	Homo sapiens membrane protein, palmitoylated 2 (MAGUK p55 subfamily member 2) (MPP2), mRNA [NM_005374]	A_23_P394259	4.52E-05	0.00204	-3.918	-1.97
ANPEP	Homo sapiens alanyl (membrane) aminopeptidase (ANPEP), mRNA [NM_001150]	A_23_P88626	4.54E-05	0.00205	-2.592	-1.374
ATXN7L1	Homo sapiens ataxin 7-like 1 (ATXN7L1), transcript variant 1, mRNA [NM_020725]	A_32_P179740	0.000046	0.00207	-6.184	-2.628
SMARCB1	Homo sapiens SWI/SNF related, matrix associated, actin dependent regulator of chromatin, subfamily b, member 1 (SMARCB1), transcript variant 1, mRNA [NM_003073]	A_33_P3232508	4.62E-05	0.00207	-2.074	-1.052
KCNN1	Homo sapiens potassium intermediate/small conductance calcium-activated channel, subfamily N, member 1 (KCNN1), mRNA [NM_002248]	A_23_P119573	4.61E-05	0.00207	-13.329	-3.736
AK126796	PREDICTED: Homo sapiens hypothetical protein LOC100506255 (LOC100506255), mRNA [XM_003403636]	A_33_P3422471	4.69E-05	0.00208	-4.506	-2.172
RIMS3	Homo sapiens regulating synaptic membrane exocytosis 3 (RIMS3), mRNA [NM_014747]	A_23_P319583	4.67E-05	0.00208	-7.328	-2.873
KIAA0182	Homo sapiens KIAA0182 (KIAA0182), transcript variant 1, mRNA [NM_014615]	A_23_P152420	4.75E-05	0.00209	-3.503	-1.809

SYT3	Homo sapiens synaptotagmin III (SYT3), transcript variant 1, mRNA [NM_032298]	A_23_P55917	4.77E-05	0.00209	-2.494	-1.319
KRT18	Homo sapiens keratin 18 (KRT18), transcript variant 1, mRNA [NM_000224]	A_32_P151544	4.83E-05	0.00211	-2.413	-1.271
POGZ	Homo sapiens pogo transposable element with ZNF domain (POGZ), transcript variant 1, mRNA [NM_015100]	A_23_P500734	4.89E-05	0.00213	-1.592	-0.67
DBN1	Homo sapiens drebrin 1 (DBN1), transcript variant 2, mRNA [NM_080881]	A_23_P156284	4.95E-05	0.00214	-2.226	-1.155
PROS1	Homo sapiens protein S (alpha) (PROS1), mRNA [NM_000313]	A_23_P73114	5.04E-05	0.00215	-2.349	-1.232
TSPAN5	Homo sapiens tetraspanin 5 (TSPAN5), mRNA [NM_005723]	A_33_P3325349	5.13E-05	0.00218	-3.01	-1.59
FIGNL1	Homo sapiens fidgetin-like 1 (FIGNL1), transcript variant 1, mRNA [NM_001042762]	A_23_P302681	5.19E-05	0.00219	-2.193	-1.133
CR607463	full-length cDNA clone CS0DD005YE10 of Neuroblastoma Cot 50-normalized of Homo sapiens (human) [CR607463]	A_33_P3367112	0.000052	0.00219	-2.852	-1.512
SUFU	Homo sapiens suppressor of fused homolog (Drosophila) (SUFU), transcript variant 1, mRNA [NM_016169]	A_23_P425304	5.25E-05	0.0022	-2.885	-1.528
VASH1	Homo sapiens vasohibin 1 (VASH1), mRNA [NM_014909]	A_23_P77000	5.27E-05	0.00221	-3.502	-1.808
IHPK2	Homo sapiens inositol hexakisphosphate kinase 2 (IP6K2), transcript variant 3, mRNA [NM_001005910]	A_23_P301138	5.33E-05	0.00223	-2.533	-1.341
TEPP	Homo sapiens testis, prostate and placenta expressed (TEPP), transcript variant 1, mRNA [NM_199046]	A_33_P3415888	5.35E-05	0.00224	-3.599	-1.848
CDK2	Homo sapiens cyclin-dependent kinase 2 (CDK2), transcript variant 1, mRNA [NM_001798]	A_23_P98898	5.45E-05	0.00226	-2.544	-1.347
C17orf28	Homo sapiens chromosome 17 open reading frame 28 (C17orf28), mRNA [NM_030630]	A_33_P3274935	5.52E-05	0.00229	-5.695	-2.51
LASS4	Homo sapiens ceramide synthase 4 (CERS4), mRNA [NM_024552]	A_23_P153867	5.62E-05	0.00231	-3.179	-1.668
SLC29A4	Homo sapiens solute carrier family 29 (nucleoside transporters), member 4 (SLC29A4), transcript variant 1, mRNA [NM_001040661]	A_23_P168551	5.65E-05	0.00232	-4.198	-2.07
NECAB3	Homo sapiens N-terminal EF-hand calcium binding protein 3 (NECAB3), transcript variant 2, mRNA [NM_031232]	A_23_P68628	5.68E-05	0.00233	-2.388	-1.256
PPP3CB	protein phosphatase 3, catalytic subunit, beta isozyme [Source:HGNC Symbol;Acc:9315] [ENST00000394822]	A_33_P3397399	0.000057	0.00233	-2.515	-1.331
C19orf22	Homo sapiens chromosome 19 open reading frame 22 (C19orf22), mRNA [NM_138774]	A_23_P315286	5.92E-05	0.00238	-2.639	-1.4
CKAP2	Homo sapiens cytoskeleton associated protein 2 (CKAP2), transcript variant 1, mRNA [NM_018204]	A_24_P99090	6.04E-05	0.00242	-2.361	-1.24
MEX3B	Homo sapiens mex-3 homolog B (C. elegans) (MEX3B), mRNA [NM_032246]	A_24_P65060	6.14E-05	0.00244	-3.114	-1.639
ENST000003944	dehydrogenase/reductase (SDR family) member 11 [Source:HGNC Symbol;Acc:28639] [ENST00000394445]	A_33_P3253960	6.13E-05	0.00244	-2.559	-1.355
PRR12	Homo sapiens proline rich 12 (PRR12), mRNA [NM_020719]	A_23_P55948	6.23E-05	0.00247	-2.112	-1.079
CASS4	Homo sapiens Cas scaffolding protein family member 4 (CASS4), transcript variant 2, mRNA [NM_020356]	A_23_P91283	6.26E-05	0.00247	-4.713	-2.237
MPP3	Homo sapiens membrane protein, palmitoylated 3 (MAGUK p55 subfamily member 3) (MPP3), transcript variant 1, mRNA [NM_001932]	A_23_P141345	6.27E-05	0.00248	-4.024	-2.009
PIP5K1C	Homo sapiens phosphatidylinositol-4-phosphate 5-kinase, type I, gamma (PIP5K1C), transcript variant 2, mRNA [NM_012398]	A_23_P330262	0.000064	0.0025	-2.31	-1.208
VKORC1	Homo sapiens vitamin K epoxide reductase complex, subunit 1 (VKORC1), transcript variant 2, mRNA [NM_206824]	A_23_P100486	6.47E-05	0.00252	-2.188	-1.13

TNK2	Homo sapiens tyrosine kinase, non-receptor, 2 (TNK2), transcript variant 1, mRNA [NM_005781]	A_23_P61633	6.52E-05	0.00252	-2.129	-1.09
HIC2	Homo sapiens hypermethylated in cancer 2 (HIC2), mRNA [NM_015094]	A_33_P3379017	6.48E-05	0.00252	-2.21	-1.144
LOC338653	Homo sapiens partial mRNA for KCNQ1OT2 protein. [AJ251642]	A_33_P3794213	6.67E-05	0.00257	-10.809	-3.434
DAND5	Homo sapiens DAN domain family, member 5 (DAND5), mRNA [NM_152654]	A_23_P435636	0.000067	0.00258	-2.937	-1.555
CCDC116	Homo sapiens coiled-coil domain containing 116 (CCDC116), mRNA [NM_152612]	A_23_P342108	6.81E-05	0.0026	-2.929	-1.55
ZFHX2	Homo sapiens zinc finger homeobox 2 (ZFHX2), mRNA [NM_033400]	A_32_P226205	0.000068	0.0026	-5.93	-2.568
ARRB1	Homo sapiens arrestin, beta 1 (ARRB1), transcript variant 1, mRNA [NM_004041]	A_24_P386622	6.81E-05	0.0026	-3.796	-1.925
MAD1L1	Homo sapiens MAD1 mitotic arrest deficient-like 1 (yeast) (MAD1L1), transcript variant 1, mRNA [NM_003550]	A_21_P0012258	6.91E-05	0.00263	-3.412	-1.77
UBE2T	Homo sapiens ubiquitin-conjugating enzyme E2T (putative) (UBE2T), mRNA [NM_014176]	A_23_P115482	0.000069	0.00263	-2.141	-1.098
C19orf64	Homo sapiens GIPC PDZ domain containing family, member 3 (GIPC3), mRNA [NM_133261]	A_24_P118527	6.98E-05	0.00265	-3.271	-1.71
BCR	Homo sapiens breakpoint cluster region (BCR), transcript variant 1, mRNA [NM_004327]	A_32_P56713	7.01E-05	0.00266	-2.292	-1.197
ST3GAL2	Homo sapiens ST3 beta-galactoside alpha-2,3-sialyltransferase 2 (ST3GAL2), mRNA [NM_006927]	A_24_P48177	7.02E-05	0.00266	-2.369	-1.244
GPRASP2	Homo sapiens G protein-coupled receptor associated sorting protein 2 (GPRASP2), transcript variant 1, mRNA [NM_001004051]	A_23_P408167	7.09E-05	0.00267	-5.3	-2.406
KLK1	Homo sapiens kallikrein 1 (KLK1), mRNA [NM_002257]	A_23_P16252	7.17E-05	0.00269	-4.887	-2.289
GPR155	Homo sapiens G protein-coupled receptor 155 (GPR155), transcript variant 9, mRNA [NM_001033045]	A_32_P132317	7.24E-05	0.00271	-2.938	-1.555
AQP7	Homo sapiens aquaporin 7 (AQP7), mRNA [NM_001170]	A_23_P158041	0.000073	0.00272	-6.513	-2.703
LOC100128281	Homo sapiens cDNA FLJ46495 fis, clone THYMU3028461. [AK128353]	A_33_P3243307	7.38E-05	0.00273	-3.755	-1.909
EPB49	Homo sapiens erythrocyte membrane protein band 4.9 (dematin) (EPB49), transcript variant 1, mRNA [NM_001978]	A_33_P3381870	7.47E-05	0.00275	-2.978	-1.574
ENST000004009	Homo sapiens cDNA FLJ43227 fis, clone HCHON2000212. [AK125217]	A_33_P3322236	7.53E-05	0.00276	-2.497	-1.32
SNX16	Homo sapiens sorting nexin 16 (SNX16), transcript variant 1, mRNA [NM_022133]	A_24_P320880	7.51E-05	0.00276	-2.245	-1.167
MUM1	Homo sapiens melanoma associated antigen (mutated) 1 (MUM1), transcript variant 1, mRNA [NM_032853]	A_23_P208961	7.58E-05	0.00277	-2.442	-1.288
MED16	Homo sapiens mediator complex subunit 16 (MED16), mRNA [NM_005481]	A_23_P56170	7.65E-05	0.00278	-2.297	-1.2
NSUN4	Homo sapiens NOP2/Sun domain family, member 4 (NSUN4), mRNA [NM_199044]	A_33_P3242264	7.64E-05	0.00278	-2.044	-1.032
GPR162	Homo sapiens G protein-coupled receptor 162 (GPR162), transcript variant A-2, mRNA [NM_019858]	A_33_P3243439	7.65E-05	0.00278	-9.381	-3.23
LOC441666	Homo sapiens zinc finger protein 91 pseudogene (LOC441666), non-coding RNA [NR_024380]	A_33_P3245517	0.000077	0.00279	-3.391	-1.762
KLK8	Homo sapiens kallikrein-related peptidase 8 (KLK8), transcript variant 2, mRNA [NM_144505]	A_23_P369343	0.000077	0.00279	-8.674	-3.117
ZNF451	Homo sapiens zinc finger protein 451 (ZNF451), transcript variant 1, mRNA [NM_001031623]	A_23_P356122	0.000079	0.00283	-1.825	-0.868
ZNF575	Homo sapiens zinc finger protein 575 (ZNF575), mRNA [NM_174945]	A_23_P401084	8.05E-05	0.00286	-5.38	-2.428

ANKRD9	Homo sapiens ankyrin repeat domain 9 (ANKRD9), mRNA [NM_152326]	A_33_P3263666	8.07E-05	0.00286	-2.006	-1.005
CSPG4	Homo sapiens chondroitin sulfate proteoglycan 4 (CSPG4), mRNA [NM_001897]	A_23_P21976	8.13E-05	0.00287	-12.807	-3.679
ZNF33B	Homo sapiens zinc finger protein 33B (ZNF33B), mRNA [NM_006955]	A_33_P3296871	8.19E-05	0.00288	-2.366	-1.243
PLEKHB1	Homo sapiens pleckstrin homology domain containing, family B (evectins) member 1 (PLEKHB1), transcript variant 1, mRNA [NM_021200]	A_33_P3308332	8.35E-05	0.00291	-15.943	-3.995
TNFRSF13C	Homo sapiens tumor necrosis factor receptor superfamily, member 13C (TNFRSF13C), mRNA [NM_052945]	A_23_P91764	0.000084	0.00292	-3.558	-1.831
A_33_P3417472	Unknown	A_33_P3417472	8.39E-05	0.00292	-15.201	-3.926
MICALCL	Homo sapiens MICAL C-terminal like (MICALCL), mRNA [NM_032867]	A_23_P2041	8.55E-05	0.00295	-5.251	-2.392
TBC1D24	Homo sapiens TBC1 domain family, member 24 (TBC1D24), transcript variant 2, mRNA [NM_020705]	A_23_P391637	8.53E-05	0.00295	-2.308	-1.207
MLL	Homo sapiens myeloid/lymphoid or mixed-lineage leukemia (trithorax homolog, Drosophila) (MLL), transcript variant 1, mRNA [NM_001197104]	A_33_P3307307	8.55E-05	0.00295	-2.004	-1.003
CIRBP	Homo sapiens cold inducible RNA binding protein (CIRBP), transcript variant 2, non-coding RNA [NR_023312]	A_23_P377616	0.000088	0.00299	-3.407	-1.768
TRIM11	Homo sapiens tripartite motif containing 11 (TRIM11), mRNA [NM_145214]	A_23_P115124	8.74E-05	0.00299	-2.338	-1.225
PLXND1	Homo sapiens plexin D1 (PLXND1), mRNA [NM_015103]	A_24_P376391	8.79E-05	0.00299	-3.489	-1.803
GALNT6	Homo sapiens UDP-N-acetyl-alpha-D-galactosamine:polypeptide N-acetylgalactosaminyltransferase 6 (GalNAc-T6) (GALNT6), mRNA [NM_007210]	A_33_P3215768	8.76E-05	0.00299	-2.722	-1.444
ARHGAP9	Homo sapiens Rho GTPase activating protein 9 (ARHGAP9), transcript variant 1, mRNA [NM_032496]	A_23_P64661	8.73E-05	0.00299	-2.839	-1.505
NFIC	Homo sapiens nuclear factor I/C (CCAAT-binding transcription factor) (NFIC), transcript variant 2, mRNA [NM_205843]	A_33_P3249509	8.92E-05	0.00301	-2.646	-1.404
CABIN1	Homo sapiens calcineurin binding protein 1 (CABIN1), transcript variant 2, mRNA [NM_012295]	A_24_P336113	8.87E-05	0.00301	-1.991	-0.994
SMC1A	Homo sapiens structural maintenance of chromosomes 1A (SMC1A), mRNA [NM_006306]	A_24_P942604	9.07E-05	0.00304	-2.325	-1.217
ST6GALNAC4	Homo sapiens ST6 (alpha-N-acetyl-neuraminyl-2,3-beta-galactosyl-1,3)-N-acetylgalactosaminide alpha-2,6-sialyltransferase 4 (ST6GALNAC4), transcript variant 1, mRNA [NM_175039]	A_23_P94736	9.05E-05	0.00304	-3.626	-1.858
SSBP3	Homo sapiens single stranded DNA binding protein 3 (SSBP3), transcript variant 3, mRNA [NM_001009955]	A_23_P500333	9.04E-05	0.00304	-3.008	-1.589
TNNT1	Homo sapiens troponin T type 1 (skeletal, slow) (TNNT1), transcript variant 1, mRNA [NM_003283]	A_33_P3397865	9.19E-05	0.00306	-3.367	-1.751
ZNF792	Homo sapiens zinc finger protein 792 (ZNF792), mRNA [NM_175872]	A_33_P3399864	9.12E-05	0.00306	-4.565	-2.191
RAB26	Homo sapiens RAB26, member RAS oncogene family (RAB26), mRNA [NM_014353]	A_33_P3209229	0.000093	0.00308	-4.4	-2.137
PYGO2	Homo sapiens pygopus homolog 2 (Drosophila) (PYGO2), mRNA [NM_138300]	A_23_P411953	9.38E-05	0.00309	-2.089	-1.063
TOR2A	Homo sapiens torsin family 2, member A (TOR2A), transcript variant 2, mRNA [NM_130459]	A_23_P60534	9.36E-05	0.00309	-2.138	-1.096
PLK3	Homo sapiens polo-like kinase 3 (PLK3), mRNA [NM_004073]	A_23_P51646	9.44E-05	0.00309	-2.551	-1.351
SMCR6	Homo sapiens mRNA; cDNA DKFZp686J19223 (from clone DKFZp686J19223). [BX647090]	A_33_P3877728	9.48E-05	0.0031	-4.21	-2.074

SPATS2	Homo sapiens spermatogenesis associated, serine-rich 2 (SPATS2), mRNA [NM_023071]	A_33_P3808996	9.47E-05	0.0031	-2.356	-1.236
CISH	Homo sapiens cytokine inducible SH2-containing protein (CISH), transcript variant 2, mRNA [NM_145071]	A_23_P144096	9.49E-05	0.00311	-2.947	-1.559
TMEM141	Homo sapiens transmembrane protein 141 (TMEM141), mRNA [NM_032928]	A_23_P94591	9.53E-05	0.00311	-2.513	-1.329
SARDH	Homo sapiens sarcosine dehydrogenase (SARDH), nuclear gene encoding mitochondrial protein, transcript variant 2, mRNA [NM_001134707]	A_33_P3257861	9.87E-05	0.00317	-4.003	-2.001
FGF11	Homo sapiens fibroblast growth factor 11 (FGF11), mRNA [NM_004112]	A_33_P3333282	9.84E-05	0.00317	-2.894	-1.533
FAM189B	Homo sapiens family with sequence similarity 189, member B (FAM189B), transcript variant 1, mRNA [NM_006589]	A_33_P3359160	9.89E-05	0.00317	-2.194	-1.134
THC2545454	Homo sapiens Mdm2 p53 binding protein homolog (mouse) (MDM2), transcript variant MDM2, mRNA [NM_002392]	A_24_P925664	0.0001	0.00318	-2.109	-1.077
SP1	Homo sapiens Sp1 transcription factor (SP1), transcript variant 1, mRNA [NM_138473]	A_33_P3330283	0.0001	0.00318	-2.111	-1.078
ZNF496	Homo sapiens zinc finger protein 496 (ZNF496), mRNA [NM_032752]	A_33_P3400244	9.98E-05	0.00318	-2.49	-1.316
HTR4	Homo sapiens 5-hydroxytryptamine (serotonin) receptor 4 (HTR4), transcript variant d, mRNA [NM_001040172]	A_23_P133637	0.0001	0.00318	-2.412	-1.27
CMPK2	Homo sapiens cytidine monophosphate (UMP-CMP) kinase 2, mitochondrial (CMPK2), nuclear gene encoding mitochondrial protein, mRNA [NM_207315]	A_33_P3401826	0.000102	0.00321	-7.959	-2.993
ENST000003756	chromosome 20 open reading frame 112 [Source:HGNC Symbol;Acc:16106] [ENST00000485364]	A_24_P101800	0.000103	0.00323	-4.245	-2.086
GAMT	Homo sapiens guanidinoacetate N-methyltransferase (GAMT), transcript variant 2, mRNA [NM_138924]	A_24_P19228	0.000104	0.00325	-2.729	-1.448
APOL4	Homo sapiens apolipoprotein L, 4 (APOL4), transcript variant a, mRNA [NM_030643]	A_33_P3276615	0.000104	0.00325	-4.827	-2.271
ENST000004009	Uncharacterized protein [Source:UniProtKB/TrEMBL;Acc:A8MVZ5] [ENST00000400948]	A_33_P3264731	0.000104	0.00325	-2.534	-1.341
ICAM5	Homo sapiens intercellular adhesion molecule 5, telencephalin (ICAM5), mRNA [NM_003259]	A_23_P119143	0.000104	0.00325	-2.562	-1.358
CHD9	Homo sapiens chromodomain helicase DNA binding protein 9 (CHD9), mRNA [NM_025134]	A_24_P942250	0.000105	0.00328	-2.126	-1.088
BPA-1	Homo sapiens BPA-1 mRNA for brain peptide A1, complete cds. [AB088847]	A_33_P3813818	0.000107	0.00332	-4.337	-2.117
NRSN2	Homo sapiens neurensin 2 (NRSN2), mRNA [NM_024958]	A_23_P143274	0.000109	0.00334	-2.78	-1.475
tcag7.1177	Homo sapiens GATS, stromal antigen 3 opposite strand (GATS), transcript variant 1, mRNA [NM_178831]	A_24_P281580	0.000109	0.00334	-3.365	-1.751
ENDOD1	Homo sapiens endonuclease domain containing 1 (ENDOD1), mRNA [NM_015036]	A_24_P189533	0.00011	0.00335	-3.368	-1.752
KIAA1522	Homo sapiens KIAA1522 (KIAA1522), transcript variant 1, mRNA [NM_020888]	A_23_P410965	0.00011	0.00335	-3.368	-1.752
LRFN3	Homo sapiens leucine rich repeat and fibronectin type III domain containing 3 (LRFN3), mRNA [NM_024509]	A_23_P50775	0.000111	0.00337	-5.967	-2.577
CCNO	Homo sapiens cyclin O (CCNO), mRNA [NM_021147]	A_23_P92860	0.000111	0.00337	-2.469	-1.304
PGLYRP4	Homo sapiens peptidoglycan recognition protein 4 (PGLYRP4), mRNA [NM_020393]	A_24_P231302	0.000112	0.00338	-9.031	-3.175
RASIP1	Homo sapiens Ras interacting protein 1 (RASIP1), mRNA [NM_017805]	A_23_P119353	0.000114	0.00341	-2.894	-1.533

CACNB3	Homo sapiens calcium channel, voltage-dependent, beta 3 subunit (CACNB3), transcript variant 1, mRNA [NM_000725]	A_23_P204016	0.000115	0.00344	-3.552	-1.829
LCN15	Homo sapiens lipocalin 15 (LCN15), mRNA [NM_203347]	A_33_P3263938	0.000117	0.00348	-5.044	-2.335
A_33_P3295932	Unknown	A_33_P3295932	0.000119	0.00351	-3.164	-1.662
GNG7	Homo sapiens guanine nucleotide binding protein (G protein), gamma 7 (GNG7), mRNA [NM_052847]	A_23_P153897	0.000119	0.00351	-5.128	-2.358
C20orf54	Homo sapiens chromosome 20 open reading frame 54 (C20orf54), mRNA [NM_033409]	A_23_P57110	0.00012	0.00353	-6.365	-2.67
PPP1R14A	Homo sapiens protein phosphatase 1, regulatory (inhibitor) subunit 14A (PPP1R14A), transcript variant 1, mRNA [NM_033256]	A_33_P3335682	0.00012	0.00353	-2.403	-1.265
LOC254057	Homo sapiens cDNA: FLJ21000 fis, clone CAE03359. [AK024653]	A_33_P3613516	0.000121	0.00354	-2.604	-1.38
GRAMD4	Homo sapiens GRAM domain containing 4 (GRAMD4), mRNA [NM_015124]	A_24_P23258	0.000121	0.00354	-3.493	-1.804
LOC100128918	Homo sapiens chromosome 6 open reading frame 132 (C6orf132), mRNA [NM_001164446]	A_32_P218355	0.000123	0.00358	-2.193	-1.133
UCP2	Homo sapiens uncoupling protein 2 (mitochondrial, proton carrier) (UCP2), nuclear gene encoding mitochondrial protein, mRNA [NM_003355]	A_23_P47704	0.000127	0.00366	-3.159	-1.66
RASGRP4	Homo sapiens RAS guanyl releasing protein 4 (RASGRP4), transcript variant a, mRNA [NM_170604]	A_23_P165007	0.000127	0.00366	-4.343	-2.119
CAMK2B	Homo sapiens calcium/calmodulin-dependent protein kinase II beta (CAMK2B), transcript variant 6, mRNA [NM_172082]	A_23_P42882	0.000127	0.00366	-5.485	-2.455
IFI27	Homo sapiens interferon, alpha-inducible protein 27 (IFI27), transcript variant 2, mRNA [NM_005532]	A_24_P270460	0.000127	0.00366	-8.102	-3.018
FMNL3	Homo sapiens formin-like 3 (FMNL3), transcript variant 1, mRNA [NM_175736]	A_33_P3415923	0.000128	0.00367	-2.034	-1.024
PTH2	Homo sapiens parathyroid hormone 2 (PTH2), mRNA [NM_178449]	A_23_P357374	0.000129	0.00368	-2.58	-1.367
GIPC1	Homo sapiens GIPC PDZ domain containing family, member 1 (GIPC1), transcript variant 1, mRNA [NM_005716]	A_33_P3390102	0.000129	0.00368	-2.238	-1.162
SERTAD3	Homo sapiens SERTA domain containing 3 (SERTAD3), transcript variant 2, mRNA [NM_203344]	A_23_P141960	0.000129	0.00368	-2.116	-1.081
KCNAB2	Homo sapiens potassium voltage-gated channel, shaker-related subfamily, beta member 2 (KCNAB2), transcript variant 1, mRNA [NM_003636]	A_24_P151	0.000129	0.00368	-4.535	-2.181
HIST1H2AB	Homo sapiens histone cluster 1, H2ab (HIST1H2AB), mRNA [NM_003513]	A_24_P223384	0.00013	0.0037	-2.64	-1.401
SAPS2	Homo sapiens protein phosphatase 6, regulatory subunit 2 (PPP6R2), transcript variant 1, mRNA [NM_001242898]	A_33_P3397279	0.00013	0.0037	-3.293	-1.719
ACAP3	Homo sapiens ArfGAP with coiled-coil, ankyrin repeat and PH domains 3 (ACAP3), mRNA [NM_030649]	A_33_P3398564	0.000131	0.00372	-2.347	-1.231
RNF144A	Homo sapiens ring finger protein 144A (RNF144A), mRNA [NM_014746]	A_24_P37264	0.000132	0.00374	-7.986	-2.997
DYRK3	Homo sapiens dual-specificity tyrosine-(Y)-phosphorylation regulated kinase 3 (DYRK3), transcript variant 2, mRNA [NM_001004023]	A_24_P345209	0.000133	0.00375	-2.278	-1.188
TMEM51	Homo sapiens transmembrane protein 51 (TMEM51), transcript variant 1, mRNA [NM_001136216]	A_33_P3363560	0.000133	0.00375	-5.688	-2.508
PBRM1	Homo sapiens polybromo 1 (PBRM1), transcript variant 2, mRNA [NM_018313]	A_33_P3330453	0.000134	0.00376	-1.625	-0.701
BC043411	Homo sapiens, clone IMAGE:6155889, mRNA. [BC043411]	A_33_P3233869	0.000135	0.00378	-3.332	-1.736
GRIN2D	Homo sapiens glutamate receptor, ionotropic, N-methyl D-aspartate 2D (GRIN2D), mRNA [NM_000836]	A_33_P3402868	0.000136	0.00379	-2.575	-1.365

UBAP2L	Homo sapiens ubiquitin associated protein 2-like (UBAP2L), transcript variant 1, mRNA [NM_014847]	A_33_P3310989	0.000138	0.00382	-2.085	-1.06
QRICH2	Homo sapiens glutamine rich 2 (QRICH2), mRNA [NM_032134]	A_33_P3306442	0.000139	0.00384	-2.166	-1.115
ZFP62	Homo sapiens zinc finger protein 62 homolog (mouse) (ZFP62), transcript variant 1, mRNA [NM_152283]	A_23_P69877	0.000141	0.00388	-1.579	-0.659
SLC35E2	Homo sapiens solute carrier family 35, member E2 (SLC35E2), transcript variant 1, mRNA [NM_182838]	A_24_P113815	0.000142	0.00389	-2.399	-1.262
MLST8	Homo sapiens MTOR associated protein, LST8 homolog (S. cerevisiae) (MLST8), transcript variant 1, mRNA [NM_022372]	A_33_P3256054	0.000144	0.00392	-2.334	-1.223
CDC42EP1	Homo sapiens CDC42 effector protein (Rho GTPase binding) 1 (CDC42EP1), mRNA [NM_152243]	A_33_P3407424	0.000146	0.00396	-3.468	-1.794
RAD51L3	Homo sapiens RAD51 homolog D (S. cerevisiae) (RAD51D), transcript variant 6, mRNA [NM_001142571]	A_24_P356509	0.000147	0.00398	-2.297	-1.2
DPY19L3	Homo sapiens dpy-19-like 3 (C. elegans) (DPY19L3), transcript variant 1, mRNA [NM_207325]	A_33_P3209816	0.000148	0.00399	-2.135	-1.094
PTGS1	Homo sapiens prostaglandin-endoperoxide synthase 1 (prostaglandin G/H synthase and cyclooxygenase) (PTGS1), transcript variant 1, mRNA [NM_000962]	A_24_P64167	0.000148	0.00399	-11.864	-3.569
C11orf67	Homo sapiens chromosome 11 open reading frame 67 (C11orf67), mRNA [NM_024684]	A_24_P49383	0.000149	0.004	-2.244	-1.166
ISYNA1	Homo sapiens inositol-3-phosphate synthase 1 (ISYNA1), transcript variant 1, mRNA [NM_016368]	A_23_P5131	0.00015	0.00402	-2.93	-1.551
FBXL8	Homo sapiens F-box and leucine-rich repeat protein 8 (FBXL8), mRNA [NM_018378]	A_33_P3284586	0.000151	0.00404	-2.074	-1.052
LOC100130248	Homo sapiens cDNA FLJ42222 fis, clone THYMU2039411. [AK124216]	A_33_P3408137	0.000151	0.00404	-4.478	-2.163
ACOT11	Homo sapiens acyl-CoA thioesterase 11 (ACOT11), transcript variant 2, mRNA [NM_147161]	A_23_P417415	0.000151	0.00404	-2.684	-1.425
GPA33	Homo sapiens glycoprotein A33 (transmembrane) (GPA33), mRNA [NM_005814]	A_24_P319374	0.000152	0.00405	-3.094	-1.63
EPB41	Homo sapiens erythrocyte membrane protein band 4.1 (elliptocytosis 1, RH-linked) (EPB41), transcript variant 4, mRNA [NM_203342]	A_23_P351	0.000155	0.00409	-2.63	-1.395
PNKP	Homo sapiens polynucleotide kinase 3'-phosphatase (PNKP), mRNA [NM_007254]	A_33_P3402918	0.000155	0.00409	-2.36	-1.239
CAPN12	Homo sapiens actinin, alpha 4 (ACTN4), mRNA [NM_004924]	A_23_P101655	0.000155	0.00409	-2.159	-1.111
REEP2	Homo sapiens receptor accessory protein 2 (REEP2), mRNA [NM_016606]	A_23_P19182	0.000155	0.00409	-4.167	-2.059
SELENBP1	Homo sapiens selenium binding protein 1 (SELENBP1), mRNA [NM_003944]	A_23_P86021	0.000156	0.0041	-3.171	-1.665
CBX5	Homo sapiens chromobox homolog 5 (CBX5), transcript variant 1, mRNA [NM_001127322]	A_24_P664995	0.000156	0.0041	-3.015	-1.592
FKBP14	Homo sapiens FK506 binding protein 14, 22 kDa (FKBP14), mRNA [NM_017946]	A_23_P215341	0.000156	0.0041	-2.859	-1.516
A_19_P00317887	Homo sapiens myosin phosphatase Rho interacting protein (MPRIP), transcript variant 2, mRNA [NM_201274]	A_19_P00317887	0.000158	0.00414	-2.122	-1.086
P4HA2	Homo sapiens prolyl 4-hydroxylase, alpha polypeptide II (P4HA2), transcript variant 1, mRNA [NM_004199]	A_23_P30363	0.00016	0.00416	-2.118	-1.083
TCF7	Homo sapiens transcription factor 7 (T-cell specific, HMG-box) (TCF7), transcript variant 1, mRNA [NM_003202]	A_23_P7582	0.00016	0.00416	-2.139	-1.097

FPGT	Homo sapiens fucose-1-phosphate guanylyltransferase (FPGT), transcript variant 1, mRNA [NM_003838]	A_33_P3388815	0.000161	0.00418	-2.017	-1.012
SETMAR	Homo sapiens SET domain and mariner transposase fusion gene (SETMAR), transcript variant 1, mRNA [NM_006515]	A_23_P80643	0.000162	0.00419	-2.084	-1.06
ERCC2	Homo sapiens excision repair cross-complementing rodent repair deficiency, complementation group 2 (ERCC2), transcript variant 1, mRNA [NM_000400]	A_23_P130488	0.000162	0.00419	-3.209	-1.682
RMND5A	Homo sapiens required for meiotic nuclear division 5 homolog A (S. cerevisiae) (RMND5A), mRNA [NM_022780]	A_33_P3237699	0.000162	0.00419	-1.43	-0.516
SLC1A7	Homo sapiens solute carrier family 1 (glutamate transporter), member 7 (SLC1A7), mRNA [NM_006671]	A_23_P325562	0.000163	0.00421	-5.768	-2.528
HIST1H3H	Homo sapiens histone cluster 1, H3h (HIST1H3H), mRNA [NM_003536]	A_33_P3404989	0.000164	0.00423	-2.368	-1.244
PROCR	Homo sapiens protein C receptor, endothelial (PROCR), mRNA [NM_006404]	A_23_P80040	0.000165	0.00424	-5.555	-2.474
C1orf2	Homo sapiens family with sequence similarity 189, member B (FAM189B), transcript variant 1, mRNA [NM_006589]	A_23_P103690	0.000166	0.00425	-2.148	-1.103
DPF1	Homo sapiens D4, zinc and double PHD fingers family 1 (DPF1), transcript variant 1, mRNA [NM_001135155]	A_33_P3317277	0.000166	0.00425	-2.706	-1.436
BCOR	Homo sapiens BCL6 corepressor (BCOR), transcript variant 1, mRNA [NM_017745]	A_23_P159741	0.000167	0.00427	-2.785	-1.478
BRSK2	Homo sapiens BR serine/threonine kinase 2 (BRSK2), mRNA [NM_003957]	A_33_P3273480	0.000169	0.00431	-2.767	-1.468
DHH	Homo sapiens desert hedgehog (DHH), mRNA [NM_021044]	A_33_P3338740	0.000169	0.00431	-11.791	-3.56
ZSWIM5	Homo sapiens zinc finger, SWIM-type containing 5 (ZSWIM5), mRNA [NM_020883]	A_23_P383118	0.000171	0.00434	-6.142	-2.619
SFRS14	Homo sapiens SURP and G patch domain containing 2 (SUGP2), transcript variant 1, mRNA [NM_001017392]	A_23_P16562	0.000172	0.00435	-1.73	-0.791
ASPSCR1	Homo sapiens alveolar soft part sarcoma chromosome region, candidate 1 (ASPSCR1), transcript variant 2, mRNA [NM_001251888]	A_33_P3228573	0.000173	0.00435	-2.094	-1.066
FAM178B	Homo sapiens family with sequence similarity 178, member B (FAM178B), transcript variant A, mRNA [NM_001122646]	A_33_P3287119	0.000172	0.00435	-7.999	-3
PQLC1	Homo sapiens PQ loop repeat containing 1 (PQLC1), transcript variant 1, mRNA [NM_025078]	A_33_P3245824	0.000173	0.00435	-2.062	-1.044
GPD1L	Homo sapiens glycerol-3-phosphate dehydrogenase 1-like (GPD1L), mRNA [NM_015141]	A_23_P318284	0.000175	0.00438	-2.737	-1.452
TIAF1	Homo sapiens myosin XVIIIA (MYO18A), transcript variant 1, mRNA [NM_078471]	A_23_P78122	0.000176	0.00439	-2.425	-1.278
BC035371	Uncharacterized protein [Source:UniProtKB/TrEMBL;Acc:C9JMR9] [ENST00000542270]	A_24_P376422	0.000179	0.00444	-4.232	-2.081
CIT	Homo sapiens citron (rho-interacting, serine/threonine kinase 21) (CIT), transcript variant 1, mRNA [NM_001206999]	A_33_P3312301	0.000179	0.00444	-2.133	-1.093
DCXR	Homo sapiens dicarbonyl/L-xylulose reductase (DCXR), transcript variant 1, mRNA [NM_016286]	A_23_P44166	0.000181	0.00448	-2.213	-1.146
CARD9	Homo sapiens caspase recruitment domain family, member 9 (CARD9), transcript variant 1, mRNA [NM_052813]	A_23_P500433	0.000181	0.00448	-4.869	-2.284
ARHGEF16	Homo sapiens Rho guanine nucleotide exchange factor (GEF) 16 (ARHGEF16), mRNA [NM_014448]	A_23_P114670	0.000182	0.00449	-4.937	-2.304
LOC100132147	Homo sapiens cDNA clone IMAGE:4816083, partial cds. [BC036435]	A_33_P3260175	0.000184	0.00453	-4.94	-2.304

CALM3	Homo sapiens calmodulin 3 (phosphorylase kinase, delta) (CALM3), mRNA [NM_005184]	A_23_P4944	0.000185	0.00454	-2.741	-1.455
SSX2IP	Homo sapiens synovial sarcoma, X breakpoint 2 interacting protein (SSX2IP), transcript variant 5, mRNA [NM_014021]	A_23_P201376	0.000186	0.00455	-2.022	-1.016
SPINK4	Homo sapiens serine peptidase inhibitor, Kazal type 4 (SPINK4), mRNA [NM_014471]	A_23_P71880	0.000186	0.00455	-19.192	-4.262
A_33_P3281176	Unknown	A_33_P3281176	0.000186	0.00455	-2.451	-1.294
A_19_P0080846´	Homo sapiens zinc finger protein 37A (ZNF37A), transcript variant 1, mRNA [NM_001007094]	A_19_P00808461	0.000187	0.00456	-3.123	-1.643
ZNF678	Homo sapiens zinc finger protein 678 (ZNF678), transcript variant 1, mRNA [NM_178549]	A_33_P3243667	0.000189	0.0046	-4.714	-2.237
ITGB4	Homo sapiens integrin, beta 4 (ITGB4), transcript variant 1, mRNA [NM_000213]	A_33_P3377364	0.000189	0.0046	-2.919	-1.545
ARVCF	Homo sapiens armadillo repeat gene deleted in velocardiofacial syndrome (ARVCF), mRNA [NM_001670]	A_24_P416961	0.000191	0.00462	-4.123	-2.044
GYPB	Homo sapiens glycophorin B (MNS blood group) (GYPB), mRNA [NM_002100]	A_33_P3362915	0.000192	0.00464	-8.867	-3.148
LDLRAP1	Homo sapiens low density lipoprotein receptor adaptor protein 1 (LDLRAP1), mRNA [NM_015627]	A_32_P216548	0.000193	0.00465	-2.348	-1.231
ATP8B2	Homo sapiens ATPase, class I, type 8B, member 2 (ATP8B2), transcript variant 2, mRNA [NM_001005855]	A_24_P366749	0.000195	0.00468	-2.826	-1.499
CHD3	Homo sapiens chromodomain helicase DNA binding protein 3 (CHD3), transcript variant 3, mRNA [NM_001005271]	A_33_P3239287	0.000196	0.0047	-2.15	-1.105
ZNF326	Homo sapiens zinc finger protein 326 (ZNF326), transcript variant 3, mRNA [NM_182975]	A_24_P83437	0.000197	0.00471	-2.615	-1.387
CARD11	Homo sapiens caspase recruitment domain family, member 11 (CARD11), mRNA [NM_032415]	A_23_P82324	0.000197	0.00471	-2.939	-1.555
UNKL	Homo sapiens unkempt homolog (Drosophila)-like (UNKL), transcript variant 3, mRNA [NM_023076]	A_23_P100141	0.000199	0.00474	-2.396	-1.261
ADAM15	Homo sapiens ADAM metallopeptidase domain 15 (ADAM15), transcript variant 6, mRNA [NM_207197]	A_33_P3380693	0.0002	0.00476	-2.066	-1.047
NMU	Homo sapiens neuromedin U (NMU), mRNA [NM_006681]	A_23_P69537	0.000202	0.00479	-2.809	-1.49
ESPNL	Homo sapiens espin-like (ESPNL), mRNA [NM_194312]	A_24_P170983	0.000202	0.00479	-3.063	-1.615
THEM4	Homo sapiens thioesterase superfamily member 4 (THEM4), mRNA [NM_053055]	A_23_P149375	0.000205	0.00482	-1.666	-0.737
LOC100131702	Homo sapiens cDNA FLJ45037 fis, clone BRAWH3019820. [AK126983]	A_33_P3374388	0.000205	0.00482	-2.157	-1.109
GPC2	Homo sapiens glypican 2 (GPC2), mRNA [NM_152742]	A_23_P251795	0.000206	0.00483	-2.914	-1.543
RRBP1	Homo sapiens ribosome binding protein 1 homolog 180kDa (dog) (RRBP1), transcript variant 1, mRNA [NM_001042576]	A_23_P120566	0.000208	0.00486	-1.937	-0.954
PGBD2	Homo sapiens piggyBac transposable element derived 2 (PGBD2), transcript variant 1, mRNA [NM_170725]	A_33_P3286608	0.000208	0.00486	-3.07	-1.618
KCNN4	Homo sapiens potassium intermediate/small conductance calcium-activated channel, subfamily N, member 4 (KCNN4), mRNA [NM_002250]	A_23_P67529	0.000208	0.00486	-7.584	-2.923
CADM4	Homo sapiens cell adhesion molecule 4 (CADM4), mRNA [NM_145296]	A_23_P5064	0.00021	0.00488	-3.552	-1.829
S100A2	Homo sapiens S100 calcium binding protein A2 (S100A2), mRNA [NM_005978]	A_33_P3376249	0.00021	0.00488	-4.498	-2.169

NES	Homo sapiens nestin (NES), mRNA [NM_006617]	A_33_P3358233	0.000211	0.00489	-3.707	-1.89
CENPT	Homo sapiens centromere protein T (CENPT), mRNA [NM_025082]	A_23_P163580	0.000214	0.00495	-2.531	-1.34
APOBEC3F	Homo sapiens apolipoprotein B mRNA editing enzyme, catalytic polypeptide-like 3F (APOBEC3F), transcript variant 2, mRNA [NM_001006666]	A_33_P3262181	0.000216	0.00498	-4.164	-2.058
DDAH2	Homo sapiens dimethylarginine dimethylaminohydrolase 2 (DDAH2), mRNA [NM_013974]	A_23_P19482	0.000217	0.00499	-3.123	-1.643
RADIL	Homo sapiens Ras association and DIL domains (RADIL), mRNA [NM_018059]	A_23_P111860	0.000218	0.005	-3.916	-1.969
EPHX2	Homo sapiens epoxide hydrolase 2, cytoplasmic (EPHX2), mRNA [NM_001979]	A_23_P8834	0.000219	0.00501	-2.51	-1.328
CENPF	Homo sapiens centromere protein F, 350/400kDa (mitosin) (CENPF), mRNA [NM_016343]	A_23_P401	0.00022	0.00502	-1.754	-0.81
KIAA0427	Homo sapiens CBP80/20-dependent translation initiation factor (CTIF), transcript variant 1, mRNA [NM_014772]	A_23_P207967	0.000221	0.00504	-2.339	-1.226
C14orf73	Homo sapiens exocyst complex component 3-like 4 (EXOC3L4), mRNA [NM_001077594]	A_33_P3360972	0.000221	0.00504	-7.296	-2.867
PRKXP1	Homo sapiens cDNA FLJ38474 fis, clone FEBRA2022255. [AK095793]	A_33_P3618429	0.000222	0.00505	-2.097	-1.068
A_19_P0032130(	Homo sapiens ring finger protein 213 (RNF213), transcript variant 1, mRNA [NM_020914]	A_19_P00321306	0.000222	0.00505	-2.229	-1.157
FLJ35767	Homo sapiens testis expressed 19 (TEX19), mRNA [NM_207459]	A_23_P375165	0.000225	0.00508	-5.504	-2.461
A_19_P00803342	Homo sapiens zinc finger protein 678 (ZNF678), transcript variant 1, mRNA [NM_178549]	A_19_P00803342	0.000227	0.0051	-2.595	-1.376
DCAKD	Homo sapiens dephospho-CoA kinase domain containing (DCAKD), transcript variant 1, mRNA [NM_024819]	A_24_P82493	0.000228	0.00512	-2.705	-1.436
TAOK2	Homo sapiens TAO kinase 2 (TAOK2), transcript variant 2, mRNA [NM_004783]	A_23_P308673	0.000229	0.00513	-1.815	-0.86
ZNF175	Homo sapiens zinc finger protein 175 (ZNF175), mRNA [NM_007147]	A_23_P332374	0.000229	0.00513	-2.667	-1.415
DPYSL5	Homo sapiens dihydropyrimidinase-like 5 (DPYSL5), mRNA [NM_020134]	A_23_P210224	0.000231	0.00515	-6.512	-2.703
TRIM65	Homo sapiens tripartite motif containing 65 (TRIM65), mRNA [NM_173547]	A_33_P3331346	0.000231	0.00515	-2.5	-1.322
SLC29A1	Homo sapiens solute carrier family 29 (nucleoside transporters), member 1 (SLC29A1), nuclear gene encoding mitochondrial protein, transcript variant 1, mRNA [NM_001078177]	A_23_P133694	0.000231	0.00515	-2.9	-1.536
CA11	Homo sapiens carbonic anhydrase XI (CA11), mRNA [NM_001217]	A_23_P78782	0.00023	0.00515	-3.732	-1.9
PRMT7	Homo sapiens protein arginine methyltransferase 7 (PRMT7), transcript variant 1, mRNA [NM_019023]	A_23_P77430	0.000232	0.00516	-2.096	-1.068
AX746567	Homo sapiens cDNA FLJ33356 fis, clone BRACE2005160. [AK090675]	A_33_P3273819	0.000234	0.00519	-5.923	-2.566
SNTA1	Homo sapiens syntrophin, alpha 1 (dystrophin-associated protein A1, 59kDa, acidic component) (SNTA1), mRNA [NM_003098]	A_24_P322709	0.000236	0.00521	-2.582	-1.369
ZNF37A	Homo sapiens zinc finger protein 37A (ZNF37A), transcript variant 1, mRNA [NM_001007094]	A_21_P0010941	0.000236	0.00521	-2.94	-1.556
ENO2	Homo sapiens enolase 2 (gamma, neuronal) (ENO2), mRNA [NM_001975]	A_24_P236091	0.000239	0.00524	-9.216	-3.204
DPY19L1	Homo sapiens dpy-19-like 1 (C. elegans) (DPY19L1), mRNA [NM_015283]	A_23_P394448	0.000239	0.00524	-2.267	-1.181
INPP5F	Homo sapiens inositol polyphosphate-5-phosphatase F (INPP5F), transcript variant 3, mRNA [NM_001243195]	A_33_P3237096	0.000238	0.00524	-2.259	-1.176
GPR146	Homo sapiens G protein-coupled receptor 146 (GPR146), mRNA [NM_138445]	A_23_P20035	0.000241	0.00526	-4.09	-2.032

GAL3ST4	Homo sapiens galactose-3-O-sulfotransferase 4 (GAL3ST4), mRNA [NM_024637]	A_23_P31240	0.000242	0.00527	-3.822	-1.934
TMCC2	PREDICTED: Homo sapiens hypothetical protein LOC100506871 (LOC100506871), mRNA [XM_003118502]	A_33_P3244895	0.000242	0.00527	-2.638	-1.4
GPR52	Homo sapiens G protein-coupled receptor 52 (GPR52), mRNA [NM_005684]	A_23_P85534	0.000243	0.00529	-4.207	-2.073
SEMA3G	Homo sapiens sema domain, immunoglobulin domain (Ig), short basic domain, secreted, (semaphorin) 3G (SEMA3G), mRNA [NM_020163]	A_23_P6818	0.000246	0.00532	-7.158	-2.839
C9orf102	Homo sapiens chromosome 9 open reading frame 102 (C9orf102), mRNA [NM_001010895]	A_33_P3298406	0.000245	0.00532	-1.859	-0.895
LOC728805	Homo sapiens cDNA clone IMAGE:5266004. [BC035175]	A_33_P3404934	0.000246	0.00532	-26.521	-4.729
A_33_P3396858	Unknown	A_33_P3396858	0.000246	0.00532	-2.953	-1.562
ZNF385A	Homo sapiens zinc finger protein 385A (ZNF385A), transcript variant 3, mRNA [NM_015481]	A_24_P403734	0.000247	0.00534	-4.103	-2.037
SORBS1	Homo sapiens sorbin and SH3 domain containing 1 (SORBS1), transcript variant 3, mRNA [NM_001034954]	A_24_P317907	0.000248	0.00535	-5.184	-2.374
A_33_P3410225	Unknown	A_33_P3410225	0.000249	0.00537	-4.836	-2.274
HCFC1R1	Homo sapiens host cell factor C1 regulator 1 (XPO1 dependent) (HCFC1R1), transcript variant 1, mRNA [NM_017885]	A_33_P3350207	0.000252	0.00542	-2.521	-1.334
LOC442249	Unknown	A_24_P256063	0.000253	0.00544	-2.697	-1.431
ANKRD23	Homo sapiens ankyrin repeat domain 23 (ANKRD23), mRNA [NM_144994]	A_33_P3277753	0.000254	0.00545	-2.47	-1.305
A_19_P0031744	Homo sapiens zinc finger protein 678 (ZNF678), transcript variant 1, mRNA [NM_178549]	A_19_P00317449	0.000256	0.00547	-2.5	-1.322
ENST000003891	Homo sapiens cDNA clone IMAGE:5172245, containing frame-shift errors. [BC031633]	A_33_P3304865	0.000258	0.00549	-2.602	-1.38
HIST1H2AG	Homo sapiens histone cluster 1, H2ag (HIST1H2AG), mRNA [NM_021064]	A_21_P0000197	0.000257	0.00549	-3.006	-1.588
HCN3	Homo sapiens hyperpolarization activated cyclic nucleotide-gated potassium channel 3 (HCN3), mRNA [NM_020897]	A_23_P34827	0.000259	0.00551	-2.133	-1.093
BCAM	Homo sapiens basal cell adhesion molecule (Lutheran blood group) (BCAM), transcript variant 2, mRNA [NM_001013257]	A_33_P3291097	0.00026	0.00552	-7.802	-2.964
MAZ	Homo sapiens MYC-associated zinc finger protein (purine-binding transcription factor) (MAZ), transcript variant 2, mRNA [NM_001042539]	A_23_P33433	0.000261	0.00554	-2.284	-1.192
ALDH16A1	Homo sapiens aldehyde dehydrogenase 16 family, member A1 (ALDH16A1), transcript variant 1, mRNA [NM_153329]	A_24_P5743	0.000264	0.00557	-3.264	-1.706
PHPT1	Homo sapiens phosphohistidine phosphatase 1 (PHPT1), transcript variant 3, mRNA [NM_014172]	A_23_P83192	0.000268	0.00562	-2.256	-1.173
LOC100131150	Homo sapiens cDNA FLJ38875 fis, clone MESAN2013936. [AK096194]	A_33_P3250253	0.000268	0.00562	-2.587	-1.371
C12orf57	Homo sapiens chromosome 12 open reading frame 57 (C12orf57), mRNA [NM_138425]	A_23_P350551	0.000269	0.00564	-2.051	-1.036
CCM2	Homo sapiens cerebral cavernous malformation 2 (CCM2), transcript variant 2, mRNA [NM_031443]	A_33_P3209476	0.000271	0.00566	-2.059	-1.042
C14orf93	Homo sapiens chromosome 14 open reading frame 93 (C14orf93), transcript variant 1, mRNA [NM_021944]	A_23_P48581	0.000272	0.00568	-2.143	-1.1
ENST000003404	Unknown	A_33_P3402611	0.000272	0.00568	-3.259	-1.705
RILP	Homo sapiens Rab interacting lysosomal protein (RILP), mRNA [NM_031430]	A_24_P185117	0.000272	0.00568	-3.452	-1.787
DLG4	Homo sapiens discs, large homolog 4 (Drosophila) (DLG4), transcript variant 1, mRNA [NM_001365]	A_23_P411102	0.000273	0.00569	-4.296	-2.103

WIPF3	WAS/WASL interacting protein family, member 3 [Source:HGNC Symbol;Acc:22004] [ENST00000409123]	A_33_P3274299	0.000273	0.00569	-3.93	-1.975
KIF5A	Homo sapiens kinesin family member 5A (KIF5A), mRNA [NM_004984]	A_23_P218025	0.000275	0.00572	-6.319	-2.66
HOXC4	Homo sapiens homeobox C4 (HOXC4), transcript variant 1, mRNA [NM_014620]	A_33_P3300975	0.000277	0.00572	-2.771	-1.47
C1orf190	Homo sapiens chromosome 1 open reading frame 190 (C1orf190), mRNA [NM_001013615]	A_33_P3400823	0.00028	0.00576	-3.878	-1.955
NAV1	Homo sapiens neuron navigator 1 (NAV1), transcript variant 1, mRNA [NM_020443]	A_24_P102880	0.000282	0.00579	-6.228	-2.639
LINGO3	Homo sapiens leucine rich repeat and Ig domain containing 3 (LINGO3), mRNA [NM_001101391]	A_33_P3302448	0.000283	0.0058	-7.992	-2.999
ERI2	Homo sapiens ERI1 exoribonuclease family member 2 (ERI2), transcript variant 1, mRNA [NM_001142725]	A_33_P3277437	0.000284	0.00582	-3.417	-1.773
WNK2	Homo sapiens WNK lysine deficient protein kinase 2 (WNK2), mRNA [NM_006648]	A_33_P3351175	0.000285	0.00583	-7.536	-2.914
ATP1B2	Homo sapiens ATPase, Na+/K+ transporting, beta 2 polypeptide (ATP1B2), mRNA [NM_001678]	A_24_P31275	0.000289	0.00588	-10.619	-3.409
A_33_P3293734	Unknown	A_33_P3293734	0.000289	0.00588	-2.161	-1.112
LMNA	Homo sapiens lamin A/C (LMNA), transcript variant 2, mRNA [NM_005572]	A_23_P34835	0.00029	0.0059	-2.396	-1.26
TMEM161A	Homo sapiens transmembrane protein 161A (TMEM161A), mRNA [NM_017814]	A_33_P3320368	0.000291	0.00592	-2.371	-1.246
DOK4	Homo sapiens docking protein 4 (DOK4), mRNA [NM_018110]	A_24_P48069	0.000291	0.00592	-2.19	-1.131
ESCO2	Homo sapiens establishment of cohesion 1 homolog 2 (S. cerevisiae) (ESCO2), mRNA [NM_001017420]	A_33_P3326210	0.000294	0.00595	-2.313	-1.21
MYL7	Homo sapiens myosin, light chain 7, regulatory (MYL7), mRNA [NM_021223]	A_23_P59738	0.000298	0.00601	-2.967	-1.569
TCEAL4	Homo sapiens transcription elongation factor A (SII)-like 4 (TCEAL4), transcript variant 1, mRNA [NM_024863]	A_23_P259166	0.000298	0.00601	-3.456	-1.789
EFNB3	Homo sapiens ephrin-B3 (EFNB3), mRNA [NM_001406]	A_24_P940006	0.000303	0.00607	-6.81	-2.768
HIST1H3E	Homo sapiens histone cluster 1, H3e (HIST1H3E), mRNA [NM_003532]	A_23_P70445	0.000303	0.00607	-2.661	-1.412
TRAK2	Homo sapiens trafficking protein, kinesin binding 2 (TRAK2), mRNA [NM_015049]	A_23_P209426	0.000303	0.00607	-2.49	-1.316
MXRA8	Homo sapiens matrix-remodelling associated 8 (MXRA8), mRNA [NM_032348]	A_24_P353905	0.000305	0.00609	-5.235	-2.388
LAPTM4B	Homo sapiens lysosomal protein transmembrane 4 beta (LAPTM4B), mRNA [NM_018407]	A_24_P414999	0.000307	0.0061	-2.158	-1.109
ARL15	Homo sapiens ADP-ribosylation factor-like 15 (ARL15), mRNA [NM_019087]	A_24_P303199	0.000306	0.0061	-2.087	-1.061
TMEM190	Homo sapiens transmembrane protein 190 (TMEM190), mRNA [NM_139172]	A_23_P107775	0.000312	0.00616	-2.443	-1.289
U2AF1L4	Homo sapiens U2 small nuclear RNA auxiliary factor 1-like 4 (U2AF1L4), transcript variant 2, mRNA [NM_144987]	A_23_P326296	0.000316	0.00621	-2.718	-1.442
LMNB2	Homo sapiens lamin B2 (LMNB2), mRNA [NM_032737]	A_23_P67725	0.000316	0.00621	-2.15	-1.104
METTL7A	Homo sapiens methyltransferase like 7A (METTL7A), mRNA [NM_014033]	A_23_P415021	0.000318	0.00624	-2.797	-1.484
LOC390213	double C2-like domains, gamma, pseudogene [Source:HGNC Symbol;Acc:37962] [ENST00000495263]	A_23_P63972	0.00032	0.00627	-4.2	-2.071
SNX32	Homo sapiens sorting nexin 32 (SNX32), mRNA [NM_152760]	A_33_P3388958	0.00032	0.00627	-2.194	-1.134
SNX26	Homo sapiens Rho GTPase activating protein 33 (ARHGAP33), transcript variant 1, mRNA [NM_052948]	A_23_P404730	0.000321	0.00628	-3.668	-1.875

METTL10	Homo sapiens methyltransferase like 10 (METTL10), mRNA [NM_212554]	A_24_P86868	0.000321	0.00628	-1.905	-0.93
FDXR	Homo sapiens ferredoxin reductase (FDXR), nuclear gene encoding mitochondrial protein, transcript variant 2, mRNA [NM_004110]	A_23_P38154	0.000323	0.00629	-4.419	-2.144
WDYHV1	Homo sapiens WDYHV motif containing 1 (WDYHV1), mRNA [NM_018024]	A_23_P71415	0.000322	0.00629	-2.032	-1.023
A_19_P0080821	Homo sapiens zinc finger protein 678 (ZNF678), transcript variant 1, mRNA [NM_178549]	A_19_P00808213	0.000322	0.00629	-2.824	-1.498
ABCB10	Homo sapiens ATP-binding cassette, sub-family B (MDR/TAP), member 10 (ABCB10), nuclear gene encoding mitochondrial protein, mRNA [NM_012089]	A_32_P23624	0.000324	0.00631	-2.07	-1.049
TMEM233	Homo sapiens transmembrane protein 233 (TMEM233), mRNA [NM_001136534]	A_33_P3441021	0.000326	0.00634	-7.754	-2.955
NF1	Homo sapiens neurofibromin 1 (NF1), transcript variant 1, mRNA [NM_001042492]	A_24_P917026	0.000327	0.00635	-2.004	-1.003
EHMT2	Homo sapiens euchromatic histone-lysine N-methyltransferase 2 (EHMT2), transcript variant NG36/G9a, mRNA [NM_006709]	A_23_P214638	0.000329	0.00637	-2.072	-1.051
SERPINH1	Homo sapiens serpin peptidase inhibitor, clade H (heat shock protein 47), member 1, (collagen binding protein 1) (SERPINH1), transcript variant 1, mRNA [NM_001207014]	A_33_P3269203	0.000329	0.00637	-2.778	-1.474
NARF	Homo sapiens nuclear prelamin A recognition factor (NARF), transcript variant 3, mRNA [NM_001038618]	A_23_P218317	0.000332	0.00642	-2.458	-1.297
TMEM103	Homo sapiens chromosome 3 open reading frame 75 (C3orf75), mRNA [NM_001031703]	A_23_P212310	0.000335	0.00646	-2.405	-1.266
LRRC20	Homo sapiens leucine rich repeat containing 20 (LRRC20), transcript variant 3, mRNA [NM_018205]	A_23_P61487	0.000336	0.00647	-2.12	-1.084
INTS6	Homo sapiens integrator complex subunit 6 (INTS6), transcript variant 3, mRNA [NM_001039938]	A_33_P3316078	0.000336	0.00647	-3.605	-1.85
AXL	Homo sapiens AXL receptor tyrosine kinase (AXL), transcript variant 1, mRNA [NM_021913]	A_23_P208389	0.000336	0.00647	-2.198	-1.136
FIS1	Homo sapiens fission 1 (mitochondrial outer membrane) homolog (S. cerevisiae) (FIS1), nuclear gene encoding mitochondrial protein, mRNA [NM_016068]	A_24_P277955	0.00034	0.00651	-2.631	-1.395
DEAF1	Homo sapiens deformed epidermal autoregulatory factor 1 (Drosophila) (DEAF1), mRNA [NM_021008]	A_23_P124044	0.00034	0.00651	-2.518	-1.332
GSTM1	Homo sapiens glutathione S-transferase mu 1 (GSTM1), transcript variant 2, mRNA [NM_146421]	A_23_P115407	0.000339	0.00651	-2.575	-1.364
C20orf117	Homo sapiens KIAA0889 (KIAA0889), transcript variant 2, mRNA [NM_199181]	A_33_P3366064	0.000342	0.00652	-4.986	-2.318
FHAD1	Homo sapiens forkhead-associated (FHA) phosphopeptide binding domain 1 (FHAD1), mRNA [NM_052929]	A_33_P3244585	0.000341	0.00652	-7.279	-2.864
KRT8	keratin 8 [Source:HGNC Symbol;Acc:6446] [ENST00000551318]	A_33_P3372368	0.000342	0.00652	-3.166	-1.663
PIGQ	Homo sapiens phosphatidylinositol glycan anchor biosynthesis, class Q (PIGQ), transcript variant 2, mRNA [NM_004204]	A_23_P502609	0.000344	0.00653	-2.238	-1.162
DIDO1	Homo sapiens death inducer-obliterator 1 (DIDO1), transcript variant 1, mRNA [NM_022105]	A_23_P395426	0.000346	0.00656	-2.711	-1.439
EXOC6	Homo sapiens exocyst complex component 6 (EXOC6), transcript variant 1, mRNA [NM_019053]	A_23_P169576	0.000348	0.00659	-1.548	-0.631

PTPRU	Homo sapiens protein tyrosine phosphatase, receptor type, U (PTPRU), transcript variant 3, mRNA [NM_005704]	A_33_P3309491	0.000349	0.00659	-4.266	-2.093
SLC9A5	Homo sapiens solute carrier family 9 (sodium/hydrogen exchanger), member 5 (SLC9A5), mRNA [NM_004594]	A_33_P3227691	0.000351	0.00662	-2.805	-1.488
SELV	Homo sapiens selenoprotein V (SELV), mRNA [NM_182704]	A_32_P174025	0.000354	0.00665	-12.61	-3.657
PPOX	Homo sapiens protoporphyrinogen oxidase (PPOX), nuclear gene encoding mitochondrial protein, transcript variant 1, mRNA [NM_000309]	A_23_P201400	0.000354	0.00665	-4.297	-2.103
BRWD3	Homo sapiens bromodomain and WD repeat domain containing 3 (BRWD3), mRNA [NM_153252]	A_32_P489130	0.000354	0.00665	-3.131	-1.647
AIF1	Homo sapiens allograft inflammatory factor 1 (AIF1), transcript variant 2, mRNA [NM_004847]	A_23_P214627	0.000353	0.00665	-4.828	-2.271
NBEAL2	Homo sapiens neurobeachin-like 2 (NBEAL2), mRNA [NM_015175]	A_23_P409168	0.000355	0.00667	-2.719	-1.443
SLC48A1	Homo sapiens solute carrier family 48 (heme transporter), member 1 (SLC48A1), mRNA [NM_017842]	A_33_P3353552	0.000356	0.00667	-2.428	-1.28
PCGF5	Homo sapiens polycomb group ring finger 5 (PCGF5), mRNA [NM_032373]	A_24_P880043	0.000361	0.00673	-2.047	-1.033
DDX26B	Homo sapiens DEAD/H (Asp-Glu-Ala-Asp/His) box polypeptide 26B (DDX26B), mRNA [NM_182540]	A_33_P3290800	0.000367	0.0068	-3.311	-1.727
A_33_P3386723	Unknown	A_33_P3386723	0.000367	0.0068	-2.783	-1.477
ENST000003668	pyrroline-5-carboxylate reductase family, member 2 [Source:HGNC Symbol;Acc:30262] [ENST00000366823]	A_33_P3251518	0.000367	0.0068	-2.858	-1.515
MBOAT7	Homo sapiens membrane bound O-acyltransferase domain containing 7 (MBOAT7), transcript variant 1, mRNA [NM_024298]	A_23_P208516	0.000374	0.00688	-2.637	-1.399
GRLF1	Homo sapiens Rho GTPase activating protein 35 (ARHGAP35), mRNA [NM_004491]	A_33_P3367481	0.000375	0.0069	-1.735	-0.795
ZDHHC13	Homo sapiens zinc finger, DHHC-type containing 13 (ZDHHC13), transcript variant 1, mRNA [NM_019028]	A_23_P13065	0.000376	0.00691	-2.139	-1.097
PACS2	Homo sapiens phosphofurin acidic cluster sorting protein 2 (PACS2), transcript variant 1, mRNA [NM_001100913]	A_24_P393372	0.000377	0.00692	-2.74	-1.454
ZNF70	Homo sapiens zinc finger protein 70 (ZNF70), mRNA [NM_021916]	A_24_P941625	0.000378	0.00693	-2.13	-1.091
ZNF367	Homo sapiens zinc finger protein 367 (ZNF367), mRNA [NM_153695]	A_23_P410625	0.000378	0.00693	-2.524	-1.336
ADAM22	Homo sapiens ADAM metallopeptidase domain 22 (ADAM22), transcript variant 5, mRNA [NM_021721]	A_24_P243741	0.000379	0.00694	-4.912	-2.296
SILV	Homo sapiens premelanosome protein (PMEL), transcript variant 3, mRNA [NM_006928]	A_23_P312851	0.000381	0.00696	-4.769	-2.254
POLE	Homo sapiens polymerase (DNA directed), epsilon (POLE), mRNA [NM_006231]	A_33_P3255794	0.000381	0.00696	-3.035	-1.602
PVRL2	Homo sapiens poliovirus receptor-related 2 (herpesvirus entry mediator B) (PVRL2), transcript variant delta, mRNA [NM_001042724]	A_23_P208293	0.000381	0.00696	-2.079	-1.056
ENST000003997	Unknown	A_32_P79190	0.000386	0.00702	-4.281	-2.098
RNF168	Homo sapiens ring finger protein 168 (RNF168), mRNA [NM_152617]	A_23_P416112	0.000386	0.00702	-2.345	-1.23
THC2502236	phosphodiesterase 4D interacting protein [Source:HGNC Symbol;Acc:15580] [ENST00000529945]	A_33_P3337931	0.000389	0.00706	-2.476	-1.308
KSR1	Homo sapiens kinase suppressor of ras 1 (KSR1), mRNA [NM_014238]	A_23_P207774	0.00039	0.00706	-2.548	-1.349
HIST1H3A	Homo sapiens histone cluster 1, H3a (HIST1H3A), mRNA [NM_003529]	A_23_P111037	0.000391	0.00707	-2.53	-1.339
MFSD2B	Homo sapiens major facilitator superfamily domain containing 2B (MFSD2B), mRNA [NM_001080473]	A_33_P3398693	0.000391	0.00707	-3.862	-1.949

FLJ32224	Homo sapiens cDNA FLJ32224 fis, clone PLACE6004336. [AK056786]	A_33_P3694746	0.000392	0.00708	-5.854	-2.549
TIE1	Homo sapiens tyrosine kinase with immunoglobulin-like and EGF-like domains 1 (TIE1), mRNA [NM_005424]	A_23_P126416	0.000394	0.00711	-6.928	-2.792
SPIN4	Homo sapiens spindlin family, member 4 (SPIN4), mRNA [NM_001012968]	A_33_P3392698	0.000395	0.00713	-3.038	-1.603
APOBEC3C	Homo sapiens apolipoprotein B mRNA editing enzyme, catalytic polypeptide-like 3C (APOBEC3C), mRNA [NM_014508]	A_23_P120931	0.000396	0.00714	-2.185	-1.128
AK122666	Homo sapiens cDNA FLJ16106 fis, clone THYMU1000496, moderately similar to KINESIN-LIKE PROTEIN KIF1C. [AK122666]	A_33_P3244117	0.000396	0.00714	-9.281	-3.214
TF	Homo sapiens transferrin (TF), mRNA [NM_001063]	A_23_P212508	0.000397	0.00714	-3.569	-1.836
MYH10	Homo sapiens myosin, heavy chain 10, non-muscle (MYH10), mRNA [NM_005964]	A_33_P3235004	0.000398	0.00715	-2.583	-1.369
BC036435	Homo sapiens cDNA clone IMAGE:4816083, partial cds. [BC036435]	A_24_P631848	0.000403	0.00721	-3.328	-1.734
OR7D2	Homo sapiens olfactory receptor, family 7, subfamily D, member 2 (OR7D2), mRNA [NM_175883]	A_23_P410408	0.000404	0.00722	-2.82	-1.496
STK31	Homo sapiens serine/threonine kinase 31 (STK31), transcript variant 2, mRNA [NM_032944]	A_33_P3221498	0.000404	0.00722	-3.265	-1.707
CD19	Homo sapiens CD19 molecule (CD19), transcript variant 2, mRNA [NM_001770]	A_23_P113572	0.000404	0.00722	-3.183	-1.67
MYO18A	Homo sapiens myosin XVIIIA (MYO18A), transcript variant 1, mRNA [NM_078471]	A_33_P3238157	0.000405	0.00723	-2.327	-1.218
C2orf34	Homo sapiens calmodulin-lysine N-methyltransferase (CAMKMT), mRNA [NM_024766]	A_33_P3241657	0.000406	0.00725	-1.545	-0.627
A_19_P0080407	Homo sapiens ring finger protein 213 (RNF213), transcript variant 1, mRNA [NM_020914]	A_19_P00804072	0.000408	0.00727	-2.274	-1.185
DDHD2	Homo sapiens DDHD domain containing 2 (DDHD2), transcript variant 3, mRNA [NM_001164234]	A_21_P0000039	0.000408	0.00727	-2.281	-1.19
A_33_P3383371	Unknown	A_33_P3383371	0.000411	0.0073	-2.178	-1.123
LOC100507419	PREDICTED: Homo sapiens hypothetical LOC100507419 (LOC100507419), miscRNA [XR_132604]	A_21_P0014800	0.000412	0.00731	-4.575	-2.194
SPC24	Homo sapiens SPC24, NDC80 kinetochore complex component, homolog (S. cerevisiae) (SPC24), mRNA [NM_182513]	A_33_P3376116	0.000412	0.00731	-3.634	-1.862
FXYD5	Homo sapiens FXYD domain containing ion transport regulator 5 (FXYD5), transcript variant 1, mRNA [NM_144779]	A_24_P194081	0.000414	0.00734	-1.611	-0.688
A_33_P3240295	Unknown	A_33_P3240295	0.000416	0.00737	-2.643	-1.402
CALML3	Homo sapiens calmodulin-like 3 (CALML3), mRNA [NM_005185]	A_23_P393080	0.000419	0.0074	-3.349	-1.744
DNAJC5G	Homo sapiens DnaJ (Hsp40) homolog, subfamily C, member 5 gamma (DNAJC5G), mRNA [NM_173650]	A_23_P417921	0.000422	0.00743	-4.294	-2.102
PLA2G5	Homo sapiens phospholipase A2, group V (PLA2G5), mRNA [NM_000929]	A_23_P103465	0.000422	0.00743	-2.284	-1.191
SNAP23	Homo sapiens synaptosomal-associated protein, 23kDa (SNAP23), transcript variant 1, mRNA [NM_003825]	A_33_P3305974	0.000429	0.00751	-2.342	-1.228
THRAP3	Homo sapiens thyroid hormone receptor associated protein 3 (THRAP3), mRNA [NM_005119]	A_21_P0012374	0.000432	0.00754	-2.334	-1.223
C5orf4	Homo sapiens chromosome 5 open reading frame 4 (C5orf4), mRNA [NM_032385]	A_23_P501831	0.000433	0.00755	-3.579	-1.839
PDE9A	Homo sapiens cDNA FLJ90181 fis, clone MAMMA1000706. [AK074662]	A_33_P3419594	0.000435	0.00757	-2.812	-1.491

ACY1	Homo sapiens aminoacylase 1 (ACY1), transcript variant 1, mRNA [NM_000666]	A_23_P57868	0.00044	0.00763	-2.175	-1.121
TRAPPC9	Homo sapiens trafficking protein particle complex 9 (TRAPPC9), transcript variant 1, mRNA [NM_031466]	A_23_P73420	0.000442	0.00765	-2.276	-1.187
GPR44	Homo sapiens G protein-coupled receptor 44 (GPR44), mRNA [NM_004778]	A_24_P115932	0.000443	0.00765	-3.755	-1.909
ZBTB46	Homo sapiens zinc finger and BTB domain containing 46 (ZBTB46), mRNA [NM_025224]	A_33_P3266674	0.000446	0.00769	-3.198	-1.677
BIK	Homo sapiens BCL2-interacting killer (apoptosis-inducing) (BIK), mRNA [NM_001197]	A_23_P404667	0.000448	0.00771	-4.082	-2.029
TK1	Homo sapiens thymidine kinase 1, soluble (TK1), mRNA [NM_003258]	A_23_P107421	0.000452	0.00777	-2.132	-1.092
ARHGEF2	Homo sapiens Rho/Rac guanine nucleotide exchange factor (GEF) 2 (ARHGEF2), transcript variant 3, mRNA [NM_004723]	A_23_P51699	0.000453	0.00778	-1.882	-0.912
STIM2	Homo sapiens stromal interaction molecule 2 (STIM2), transcript variant 2, mRNA [NM_020860]	A_23_P81048	0.000455	0.0078	-2.023	-1.016
PRELID2	Homo sapiens PRELI domain containing 2 (PRELID2), transcript variant 3, mRNA [NM_138492]	A_24_P346101	0.000457	0.00783	-2.732	-1.45
DIRAS1	Homo sapiens DIRAS family, GTP-binding RAS-like 1 (DIRAS1), mRNA [NM_145173]	A_23_P386942	0.000459	0.00785	-3.157	-1.659
HLTF	Homo sapiens helicase-like transcription factor (HLTF), transcript variant 1, mRNA [NM_003071]	A_24_P277155	0.000464	0.0079	-2.135	-1.094
KLRC3	Homo sapiens killer cell lectin-like receptor subfamily C, member 3 (KLRC3), transcript variant 2, mRNA [NM_007333]	A_23_P128281	0.000465	0.00791	-5.901	-2.561
SEMA7A	Homo sapiens semaphorin 7A, GPI membrane anchor (John Milton Hagen blood group) (SEMA7A), transcript variant 1, mRNA [NM_003612]	A_23_P106389	0.000466	0.00791	-5.544	-2.471
KRT18P19	Unknown	A_24_P247454	0.000466	0.00791	-2.565	-1.359
FLJ36208	Homo sapiens NHL repeat containing 4 (NHLRC4), mRNA [NM_176677]	A_24_P316257	0.000469	0.00793	-2.218	-1.149
HICE1	Homo sapiens HAUS augmin-like complex, subunit 8 (HAUS8), transcript variant 1, mRNA [NM_033417]	A_23_P141965	0.00047	0.00794	-2.063	-1.045
FBXO44	Homo sapiens F-box protein 44 (FBXO44), transcript variant 4, mRNA [NM_001014765]	A_33_P3237235	0.000471	0.00794	-3.165	-1.662
TDRD3	tudor domain containing 3 [Source:HGNC Symbol;Acc:20612] [ENST00000484389]	A_33_P3359791	0.00047	0.00794	-2.861	-1.517
AZI1	Homo sapiens 5-azacytidine induced 1 (AZI1), transcript variant 1, mRNA [NM_014984]	A_23_P319270	0.000475	0.00799	-2.995	-1.583
ENG	Homo sapiens endoglin (ENG), transcript variant 2, mRNA [NM_000118]	A_23_P83328	0.000476	0.008	-3.153	-1.657
PLCXD1	Homo sapiens phosphatidylinositol-specific phospholipase C, X domain containing 1 (PLCXD1), transcript variant 1, mRNA [NM_018390]	A_23_P61180	0.000476	0.008	-2.044	-1.031
FAM98B	Homo sapiens family with sequence similarity 98, member B (FAM98B), transcript variant 2, mRNA [NM_001042429]	A_33_P3378772	0.000477	0.00801	-2.132	-1.092
HEATR7A	Homo sapiens HEAT repeat containing 7A (HEATR7A), transcript variant 1, mRNA [NM_032450]	A_33_P3404189	0.000479	0.00803	-1.875	-0.907
SPACA3	Homo sapiens sperm acrosome associated 3 (SPACA3), mRNA [NM_173847]	A_23_P317244	0.000483	0.00807	-2.664	-1.414
LEPR	Homo sapiens leptin receptor (LEPR), transcript variant 5, mRNA [NM_001198688]	A_33_P3303449	0.00049	0.00815	-5.933	-2.569
SUSD2	Homo sapiens sushi domain containing 2 (SUSD2), mRNA [NM_019601]	A_23_P314101	0.000498	0.00823	-2.816	-1.494

TNKS1BP1	Homo sapiens tankyrase 1 binding protein 1, 182kDa (TNKS1BP1), mRNA [NM_033396]	A_23_P418597	0.0005	0.00825	-3.055	-1.611
MYLC2PL	Homo sapiens myosin, light chain 10, regulatory (MYL10), mRNA [NM_138403]	A_23_P393015	0.000499	0.00825	-8.836	-3.143
SSBP4	Homo sapiens single stranded DNA binding protein 4 (SSBP4), transcript variant 1, mRNA [NM_032627]	A_24_P410086	0.000501	0.00826	-2.24	-1.164
SLC13A4	Homo sapiens solute carrier family 13 (sodium/sulfate symporters), member 4 (SLC13A4), mRNA [NM_012450]	A_23_P19778	0.000505	0.00831	-3.022	-1.595
CPNE5	Homo sapiens copine V (CPNE5), mRNA [NM_020939]	A_23_P360804	0.000506	0.00831	-4.49	-2.167
SEMA3F	Homo sapiens sema domain, immunoglobulin domain (Ig), short basic domain, secreted, (semaphorin) 3F (SEMA3F), mRNA [NM_004186]	A_33_P3813128	0.000508	0.00833	-4.393	-2.135
GALNT12	Homo sapiens UDP-N-acetyl-alpha-D-galactosamine:polypeptide N-acetylgalactosaminyltransferase 12 (GalNAc-T12) (GALNT12), mRNA [NM_024642]	A_23_P415652	0.000511	0.00836	-2.586	-1.37
FRAG1	Homo sapiens post-GPI attachment to proteins 2 (PGAP2), transcript variant 1, mRNA [NM_014489]	A_23_P64560	0.000511	0.00836	-2.603	-1.38
TNFRSF25	Homo sapiens tumor necrosis factor receptor superfamily, member 25 (TNFRSF25), transcript variant 1, mRNA [NM_148965]	A_23_P126844	0.000512	0.00837	-3.445	-1.784
LIN7B	Homo sapiens lin-7 homolog B (C. elegans) (LIN7B), mRNA [NM_022165]	A_23_P164912	0.000513	0.00838	-2.159	-1.11
C11orf9	Homo sapiens chromosome 11 open reading frame 9 (C11orf9), transcript variant 1, mRNA [NM_013279]	A_23_P75790	0.000515	0.0084	-2.426	-1.279
ITFG2	Homo sapiens integrin alpha FG-GAP repeat containing 2 (ITFG2), mRNA [NM_018463]	A_23_P204417	0.000515	0.0084	-2.176	-1.122
WDR86	Homo sapiens WD repeat domain 86 (WDR86), mRNA [NM_198285]	A_33_P3341601	0.000515	0.0084	-4.036	-2.013
DEGS2	Homo sapiens degenerative spermatocyte homolog 2, lipid desaturase (Drosophila) (DEGS2), mRNA [NM_206918]	A_33_P3290174	0.000521	0.00846	-2.483	-1.312
ICAM4	Homo sapiens intercellular adhesion molecule 4 (Landsteiner-Wiener blood group) (ICAM4), transcript variant 2, mRNA [NM_022377]	A_33_P3363355	0.00052	0.00846	-2.987	-1.579
FKBP1B	Homo sapiens FK506 binding protein 1B, 12.6 kDa (FKBP1B), transcript variant 2, mRNA [NM_054033]	A_23_P142631	0.000522	0.00846	-2.222	-1.152
HYI	Homo sapiens hydroxypyruvate isomerase (putative) (HYI), transcript variant 1, mRNA [NM_031207]	A_23_P160582	0.000527	0.00852	-3.696	-1.886
C19orf55	Homo sapiens chromosome 19 open reading frame 55 (C19orf55), mRNA [NM_001039887]	A_24_P57170	0.000527	0.00852	-3.122	-1.642
AKAP11	Homo sapiens A kinase (PRKA) anchor protein 11 (AKAP11), mRNA [NM_016248]	A_23_P204929	0.000533	0.00858	-1.84	-0.879
ZNF428	Homo sapiens zinc finger protein 428 (ZNF428), mRNA [NM_182498]	A_33_P3339051	0.000533	0.00858	-2.196	-1.135
LOC100129503	Uncharacterized protein [Source:UniProtKB/TrEMBL;Acc:Q9HBN7] [ENST00000321930]	A_33_P3235034	0.000533	0.00858	-2.139	-1.097
ASXL2	Homo sapiens additional sex combs like 2 (Drosophila) (ASXL2), mRNA [NM_018263]	A_23_P210300	0.000554	0.00881	-1.54	-0.623
LRP5	Homo sapiens low density lipoprotein receptor-related protein 5 (LRP5), mRNA [NM_002335]	A_23_P1505	0.000555	0.00881	-2.195	-1.134
BIN1	Homo sapiens bridging integrator 1 (BIN1), transcript variant 4, mRNA [NM_139346]	A_23_P165333	0.000554	0.00881	-4.987	-2.318
SDC3	Homo sapiens syndecan 3 (SDC3), mRNA [NM_014654]	A_33_P3241884	0.000553	0.00881	-2.476	-1.308

EMID1	Homo sapiens EMI domain containing 1 (EMID1), mRNA [NM_133455]	A_24_P14634	0.000556	0.00882	-2.703	-1.435
LOC388588	Homo sapiens uncharacterized LOC388588 (LOC388588), mRNA [NM_001163724]	A_33_P3394868	0.000561	0.00888	-4.441	-2.151
ANK1	Homo sapiens ankyrin 1, erythrocytic (ANK1), transcript variant 3, mRNA [NM_000037]	A_23_P216108	0.000563	0.00891	-3.241	-1.697
RAB6B	Homo sapiens RAB6B, member RAS oncogene family (RAB6B), mRNA [NM_016577]	A_24_P475349	0.000565	0.00893	-10.853	-3.44
ZNF551	Homo sapiens zinc finger protein 551 (ZNF551), mRNA [NM_138347]	A_24_P68019	0.000568	0.00895	-2.747	-1.458
PRKX	Homo sapiens protein kinase, X-linked (PRKX), mRNA [NM_005044]	A_23_P217339	0.000569	0.00897	-2.187	-1.129
SIPA1	Homo sapiens signal-induced proliferation-associated 1 (SIPA1), transcript variant 1, mRNA [NM_153253]	A_23_P127460	0.00057	0.00897	-2.973	-1.572
TIMM13	Homo sapiens translocase of inner mitochondrial membrane 13 homolog (yeast) (TIMM13), nuclear gene encoding mitochondrial protein, mRNA [NM_012458]	A_33_P3413845	0.000573	0.00901	-3.139	-1.65
NACAD	Homo sapiens NAC alpha domain containing (NACAD), mRNA [NM_001146334]	A_23_P93938	0.000575	0.00903	-2.536	-1.343
MTHFR	Homo sapiens methylenetetrahydrofolate reductase (NAD(P)H) (MTHFR), mRNA [NM_005957]	A_23_P400078	0.000579	0.00906	-2.623	-1.391
A_33_P3402071	Unknown	A_33_P3402071	0.000579	0.00906	-2.68	-1.422
COL11A2	Homo sapiens collagen, type XI, alpha 2 (COL11A2), transcript variant 1, mRNA [NM_080680]	A_33_P3216442	0.000581	0.00908	-4.572	-2.193
GDF11	Homo sapiens growth differentiation factor 11 (GDF11), mRNA [NM_005811]	A_23_P76102	0.000581	0.00908	-5.87	-2.553
NFIX	Homo sapiens nuclear factor I/X (CCAAT-binding transcription factor) (NFIX), mRNA [NM_002501]	A_33_P3884230	0.000588	0.00914	-4.044	-2.016
HM13	Homo sapiens histocompatibility (minor) 13 (HM13), transcript variant 4, mRNA [NM_178582]	A_23_P396867	0.00059	0.00916	-2.165	-1.114
STK25	Homo sapiens serine/threonine kinase 25 (STK25), mRNA [NM_006374]	A_23_P252653	0.00059	0.00916	-2.254	-1.172
NEK1	Homo sapiens NIMA (never in mitosis gene a)-related kinase 1 (NEK1), transcript variant 2, mRNA [NM_012224]	A_23_P124427	0.000591	0.00917	-1.953	-0.966
GTF2I	Homo sapiens general transcription factor IIi (GTF2I), transcript variant 1, mRNA [NM_032999]	A_21_P0013236	0.000594	0.00919	-3.663	-1.873
PLEKHF1	Homo sapiens pleckstrin homology domain containing, family F (with FYVE domain) member 1 (PLEKHF1), mRNA [NM_024310]	A_23_P79134	0.000598	0.00923	-2.192	-1.132
FANCC	Homo sapiens Fanconi anemia, complementation group C (FANCC), transcript variant 1, mRNA [NM_000136]	A_23_P32021	0.00061	0.00934	-1.542	-0.625
THC2719035	Unknown	A_33_P3405904	0.000611	0.00935	-2.25	-1.17
A_19_P0031623ͅ	Homo sapiens stathmin 1 (STMN1), transcript variant 4, mRNA [NM_001145454]	A_19_P00316238	0.000612	0.00936	-3.618	-1.855
LOC222070	Homo sapiens ubiquitin-conjugating enzyme E2D 4 (putative) (UBE2D4), mRNA [NM_015983]	A_33_P3637909	0.000614	0.00937	-3.013	-1.591
C16orf11	Homo sapiens chromosome 16 open reading frame 11 (C16orf11), mRNA [NM_145270]	A_33_P3420633	0.000615	0.00938	-9.865	-3.302
ADSSL1	Homo sapiens adenylosuccinate synthase like 1 (ADSSL1), transcript variant 1, mRNA [NM_199165]	A_23_P76823	0.00062	0.00943	-2.377	-1.249
MESP1	Homo sapiens mesoderm posterior 1 homolog (mouse) (MESP1), mRNA [NM_018670]	A_33_P3214466	0.000626	0.00949	-4.861	-2.281

CAND2	Homo sapiens cullin-associated and neddylation-dissociated 2 (putative) (CAND2), transcript variant 2, mRNA [NM_012298]	A_23_P250102	0.000627	0.00949	-2.368	-1.243
ZYG11B	Homo sapiens zyg-11 homolog B (C. elegans) (ZYG11B), mRNA [NM_024646]	A_23_P426140	0.000629	0.00952	-2.356	-1.236
BMP2K	Homo sapiens BMP2 inducible kinase (BMP2K), transcript variant 2, mRNA [NM_017593]	A_23_P334282	0.00063	0.00952	-2.484	-1.313
GATA5	Homo sapiens GATA binding protein 5 (GATA5), mRNA [NM_080473]	A_23_P371835	0.000631	0.00953	-6.003	-2.586
LYL1	Homo sapiens lymphoblastic leukemia derived sequence 1 (LYL1), mRNA [NM_005583]	A_33_P3215422	0.000631	0.00953	-5.01	-2.325
PTPRS	Homo sapiens protein tyrosine phosphatase, receptor type, S (PTPRS), transcript variant 1, mRNA [NM_002850]	A_33_P3309468	0.000632	0.00954	-2.289	-1.195
C9orf142	Homo sapiens chromosome 9 open reading frame 142 (C9orf142), mRNA [NM_183241]	A_23_P312646	0.000635	0.00957	-2.218	-1.149
EGFL8	Homo sapiens EGF-like-domain, multiple 8 (EGFL8), transcript variant 1, mRNA [NM_030652]	A_23_P424582	0.000637	0.00959	-2.144	-1.1
ZNF765	Homo sapiens zinc finger protein 765 (ZNF765), mRNA [NM_001040185]	A_33_P3273309	0.000641	0.00963	-2.276	-1.187
ENST000002643	chromosome 4 open reading frame 21 [Source:HGNC Symbol;Acc:25654] [ENST00000264370]	A_33_P3316835	0.000644	0.00966	-2.704	-1.435
RHPN2	Homo sapiens rhophilin, Rho GTPase binding protein 2 (RHPN2), mRNA [NM_033103]	A_23_P119464	0.000648	0.00971	-4.411	-2.141
RABEP2	Homo sapiens rabaptin, RAB GTPase binding effector protein 2 (RABEP2), mRNA [NM_024816]	A_33_P3239152	0.000648	0.00971	-2.84	-1.506
NOTCH3	Homo sapiens notch 3 (NOTCH3), mRNA [NM_000435]	A_33_P3313055	0.00065	0.00972	-2.055	-1.039
TESC	Homo sapiens tescalcin (TESC), transcript variant 1, mRNA [NM_017899]	A_23_P76538	0.000654	0.00975	-10.188	-3.349
ESPL1	Homo sapiens extra spindle pole bodies homolog 1 (S. cerevisiae) (ESPL1), mRNA [NM_012291]	A_23_P32707	0.000654	0.00975	-2.046	-1.033
RNFT2	Homo sapiens ring finger protein, transmembrane 2 (RNFT2), transcript variant 2, mRNA [NM_032814]	A_23_P204158	0.000653	0.00975	-2.213	-1.146
CERK	Homo sapiens ceramide kinase (CERK), mRNA [NM_022766]	A_24_P62237	0.000655	0.00976	-2.485	-1.313
ST6GALNAC1	Homo sapiens ST6 (alpha-N-acetyl-neuraminyl-2,3-beta-galactosyl-1,3)-N-acetylgalactosaminide alpha-2,6-sialyltransferase 1 (ST6GALNAC1), mRNA [NM_018414]	A_23_P54968	0.000655	0.00976	-5.557	-2.474
INTS9	Homo sapiens integrator complex subunit 9 (INTS9), transcript variant 1, mRNA [NM_018250]	A_23_P8848	0.000657	0.00978	-2.422	-1.276
SUV420H2	Homo sapiens suppressor of variegation 4-20 homolog 2 (Drosophila) (SUV420H2), mRNA [NM_032701]	A_33_P3359473	0.000658	0.00979	-2.202	-1.139
ENST000003396	Unknown	A_24_P818268	0.000662	0.00983	-2.92	-1.546
CLCF1	Homo sapiens cardiotrophin-like cytokine factor 1 (CLCF1), transcript variant 1, mRNA [NM_013246]	A_23_P138760	0.000663	0.00984	-2.082	-1.058
AK058117	PREDICTED: Homo sapiens ankyrin repeat domain-containing protein 18B-like (LOC100287922), partial miscRNA [XR_132563]	A_33_P3297525	0.000665	0.00986	-3.467	-1.794
HIST1H2AI	Homo sapiens histone cluster 1, H2ai (HIST1H2AI), mRNA [NM_003509]	A_33_P3360216	0.000668	0.00989	-3.06	-1.613
TMEM145	Homo sapiens transmembrane protein 145 (TMEM145), mRNA [NM_173633]	A_23_P67127	0.000677	0.00997	-4.579	-2.195
ENST000003257	Homo sapiens cDNA FLJ90199 fis, clone MAMMA1001609. [AK074680]	A_24_P195669	0.000679	0.00999	-4.541	-2.183
AK027667	Homo sapiens cDNA FLJ14761 fis, clone NT2RP3003302. [AK027667]	A_24_P178631	0.000683	0.01	-2.766	-1.468
RAG1	Homo sapiens recombination activating gene 1 (RAG1), mRNA [NM_000448]	A_23_P360744	0.000684	0.01	-3.604	-1.849

MEX3A	Homo sapiens mex-3 homolog A (C. elegans) (MEX3A), mRNA [NM_001093725]	A_32_P96036	0.00069	0.0101	-4.412	-2.141
TCF25	Homo sapiens transcription factor 25 (basic helix-loop-helix) (TCF25), mRNA [NM_014972]	A_23_P49220	0.000689	0.0101	-2.696	-1.431
USP2	Homo sapiens ubiquitin specific peptidase 2 (USP2), transcript variant 2, mRNA [NM_171997]	A_23_P24966	0.000688	0.0101	-5.164	-2.368
A_33_P3281716	Unknown	A_33_P3281716	0.000687	0.0101	-2.121	-1.085
TMEM106C	Homo sapiens transmembrane protein 106C (TMEM106C), transcript variant 2, mRNA [NM_024056]	A_23_P48175	0.000691	0.0101	-2.36	-1.239
NXF3	Homo sapiens nuclear RNA export factor 3 (NXF3), mRNA [NM_022052]	A_23_P171336	0.000702	0.0102	-5.49	-2.457
KIAA0226	Homo sapiens KIAA0226 (KIAA0226), transcript variant 1, mRNA [NM_001145642]	A_23_P304171	0.000698	0.0102	-2.381	-1.252
TNRC18	Homo sapiens trinucleotide repeat containing 18 (TNRC18), mRNA [NM_001080495]	A_23_P377434	0.000705	0.0102	-2.482	-1.311
CRIP1	Homo sapiens cysteine-rich protein 1 (intestinal) (CRIP1), mRNA [NM_001311]	A_23_P44674	0.000698	0.0102	-5.077	-2.344
CDC42BPG	Homo sapiens CDC42 binding protein kinase gamma (DMPK-like) (CDC42BPG), mRNA [NM_017525]	A_33_P3264224	0.0007	0.0102	-3.712	-1.892
CD79B	Homo sapiens CD79b molecule, immunoglobulin-associated beta (CD79B), transcript variant 3, mRNA [NM_001039933]	A_23_P207201	0.000704	0.0102	-10.609	-3.407
GNAZ	Homo sapiens guanine nucleotide binding protein (G protein), alpha z polypeptide (GNAZ), mRNA [NM_002073]	A_23_P416581	0.000706	0.0102	-12.311	-3.622
MPND	Homo sapiens MPN domain containing (MPND), transcript variant 1, mRNA [NM_032868]	A_24_P44931	0.000714	0.0103	-2.149	-1.104
GYPC	Homo sapiens glycophorin C (Gerbich blood group) (GYPC), transcript variant 1, mRNA [NM_002101]	A_24_P139901	0.00072	0.0103	-4.285	-2.099
SF1	splicing factor 1 [Source:HGNC Symbol;Acc:12950] [ENST00000463343]	A_24_P235783	0.000713	0.0103	-2.693	-1.429
UNK	Homo sapiens unkempt homolog (Drosophila) (UNK), transcript variant 1, mRNA [NM_001080419]	A_32_P514790	0.000716	0.0103	-2.576	-1.365
FAM20B	Homo sapiens family with sequence similarity 20, member B (FAM20B), mRNA [NM_014864]	A_24_P148043	0.000713	0.0103	-2.095	-1.067
SH3D20	Homo sapiens Rho GTPase activating protein 27 (ARHGAP27), transcript variant 3, mRNA [NM_174919]	A_33_P3334102	0.000713	0.0103	-2.119	-1.083
PLEKHH2	Homo sapiens pleckstrin homology domain containing, family H (with MyTH4 domain) member 2 (PLEKHH2), mRNA [NM_172069]	A_33_P3315719	0.000715	0.0103	-3.697	-1.886
C16orf55	Homo sapiens chromosome 16 open reading frame 55 (C16orf55), mRNA [NM_153025]	A_32_P107493	0.000733	0.0105	-2.148	-1.103
FBXO31	Homo sapiens chromosome 16 open reading frame 95 (C16orf95), transcript variant 1, mRNA [NM_001195124]	A_23_P89030	0.000732	0.0105	-4.44	-2.151
A_19_P0031953	NEK4_HUMAN (P51957) Serine/threonine-protein kinase Nek4 (NimA-related protein kinase 4) (Serine/threonine-protein kinase 2) (Serine/threonine-protein kinase NRK2) , partial (5%) [THC2563770]	A_19_P00319532	0.000741	0.0105	-3.145	-1.653
CA8	Homo sapiens carbonic anhydrase VIII (CA8), mRNA [NM_004056]	A_23_P83838	0.000732	0.0105	-4.627	-2.21
ZNF614	Homo sapiens zinc finger protein 614 (ZNF614), mRNA [NM_025040]	A_23_P355993	0.000752	0.0106	-2.53	-1.339
HIST1H3B	Homo sapiens histone cluster 1, H3b (HIST1H3B), mRNA [NM_003537]	A_23_P93258	0.000746	0.0106	-2.329	-1.22
NKX2-3	Homo sapiens NK2 homeobox 3 (NKX2-3), mRNA [NM_145285]	A_24_P38702	0.000744	0.0106	-2.498	-1.321

THC2642791	smg-7 homolog, nonsense mediated mRNA decay factor (C. elegans) [Source:HGNC Symbol;Acc:16792] [ENST00000440812]	A_33_P3356035	0.000745	0.0106	-3.718	-1.894
C5orf28	Homo sapiens chromosome 5 open reading frame 28 (C5orf28), mRNA [NM_022483]	A_23_P121875	0.000752	0.0106	-2.097	-1.068
FADS6	Homo sapiens fatty acid desaturase domain family, member 6 (FADS6), mRNA [NM_178128]	A_24_P211842	0.000756	0.0107	-4.202	-2.071
ZMYND8	zinc finger, MYND-type containing 8 [Source:HGNC Symbol;Acc:9397] [ENST00000468376]	A_23_P369666	0.00076	0.0107	-3.639	-1.864
NACC1	Homo sapiens nucleus accumbens associated 1, BEN and BTB (POZ) domain containing (NACC1), mRNA [NM_052876]	A_24_P915007	0.000755	0.0107	-2.164	-1.113
MERTK	Homo sapiens c-mer proto-oncogene tyrosine kinase (MERTK), mRNA [NM_006343]	A_33_P3402091	0.000763	0.0107	-2.053	-1.038
FLJ45482	Homo sapiens cDNA FLJ45482 fis, clone BRTHA2001953. [AK127393]	A_33_P3272412	0.000755	0.0107	-3.871	-1.953
OTUD3	Homo sapiens OTU domain containing 3 (OTUD3), mRNA [NM_015207]	A_24_P941188	0.000764	0.0108	-2.182	-1.125
CRLF1	Homo sapiens cytokine receptor-like factor 1 (CRLF1), mRNA [NM_004750]	A_33_P3252286	0.00077	0.0108	-3.2	-1.678
A_33_P3419735	Unknown	A_33_P3419735	0.000767	0.0108	-2.461	-1.299
IL17RC	Homo sapiens interleukin 17 receptor C (IL17RC), transcript variant 2, mRNA [NM_153461]	A_23_P166775	0.000767	0.0108	-3.054	-1.611
CRTAP	Homo sapiens cartilage associated protein (CRTAP), mRNA [NM_006371]	A_24_P71661	0.000786	0.0109	-3.993	-1.998
SETD8	Homo sapiens SET domain containing (lysine methyltransferase) 8 (SETD8), mRNA [NM_020382]	A_32_P191859	0.000782	0.0109	-2.614	-1.386
IMMT	inner membrane protein, mitochondrial [Source:HGNC Symbol;Acc:6047] [ENST00000474969]	A_33_P3314471	0.000784	0.0109	-2.839	-1.505
TRIM45	Homo sapiens tripartite motif containing 45 (TRIM45), transcript variant 1, mRNA [NM_025188]	A_33_P3234197	0.000785	0.0109	-2.569	-1.361
RAD54L	Homo sapiens RAD54-like (S. cerevisiae) (RAD54L), transcript variant 1, mRNA [NM_003579]	A_23_P74115	0.000778	0.0109	-2.744	-1.456
CENPN	Homo sapiens centromere protein N (CENPN), transcript variant 3, mRNA [NM_018455]	A_23_P88740	0.000789	0.011	-1.347	-0.43
ZNF524	Homo sapiens zinc finger protein 524 (ZNF524), mRNA [NM_153219]	A_23_P376735	0.000794	0.011	-2.125	-1.088
SARS2	Homo sapiens seryl-tRNA synthetase 2, mitochondrial (SARS2), nuclear gene encoding mitochondrial protein, transcript variant 2, mRNA [NM_017827]	A_23_P90484	0.000794	0.011	-2.352	-1.234
AX747140	Homo sapiens cDNA FLJ34386 fis, clone HCHON1000166. [AK091705]	A_33_P3262884	0.000791	0.011	-2.563	-1.358
UBE2D4	Homo sapiens ubiquitin-conjugating enzyme E2D 4 (putative) (UBE2D4), mRNA [NM_015983]	A_23_P374767	0.000804	0.0111	-4.01	-2.004
FAM65C	Homo sapiens family with sequence similarity 65, member C (FAM65C), mRNA [NM_080829]	A_33_P3238290	0.000799	0.0111	-10.374	-3.375
OGFOD2	Homo sapiens 2-oxoglutarate and iron-dependent oxygenase domain containing 2 (OGFOD2), mRNA [NM_024623]	A_33_P3245927	0.00081	0.0111	-1.87	-0.903
ECM1	Homo sapiens extracellular matrix protein 1 (ECM1), transcript variant 1, mRNA [NM_004425]	A_23_P160559	0.00081	0.0111	-7.937	-2.989
AQP10	Homo sapiens aquaporin 10 (AQP10), mRNA [NM_080429]	A_23_P126613	0.000808	0.0111	-21.503	-4.426
ENST000004037	Unknown	A_24_P281643	0.000817	0.0112	-3.309	-1.726
GYPE	Homo sapiens glycophorin E (MNS blood group) (GYPE), transcript variant 1, mRNA [NM_002102]	A_23_P41304	0.000821	0.0112	-11.117	-3.475

HIST1H2AJ	Homo sapiens histone cluster 1, H2aj (HIST1H2AJ), mRNA [NM_021066]	A_33_P3344086	0.000818	0.0112	-2.388	-1.256
ANKRD27	Homo sapiens ankyrin repeat domain 27 (VPS9 domain) (ANKRD27), mRNA [NM_032139]	A_33_P3280192	0.000822	0.0112	-3.437	-1.781
GALNTL1	Homo sapiens UDP-N-acetyl-alpha-D-galactosamine:polypeptide N-acetylgalactosaminyltransferase-like 1 (GALNTL1), transcript variant 2, mRNA [NM_020692]	A_23_P76749	0.00083	0.0113	-11.705	-3.549
POLD1	Homo sapiens polymerase (DNA directed), delta 1, catalytic subunit 125kDa (POLD1), mRNA [NM_002691]	A_23_P50455	0.000825	0.0113	-2.178	-1.123
LOC100130998	Homo sapiens cDNA FLJ38645 fis, clone HHDPC2005794. [AK095964]	A_24_P187859	0.000829	0.0113	-3.303	-1.724
MYNN	Homo sapiens myoneurin (MYNN), transcript variant 3, mRNA [NM_001185119]	A_33_P3303104	0.000833	0.0113	-2.938	-1.555
SF3A2	Homo sapiens splicing factor 3a, subunit 2, 66kDa (SF3A2), mRNA [NM_007165]	A_33_P3210079	0.000835	0.0114	-2.169	-1.117
JPH4	Homo sapiens junctophilin 4 (JPH4), transcript variant 1, mRNA [NM_032452]	A_33_P3219279	0.000834	0.0114	-5.201	-2.379
APLP1	Homo sapiens amyloid beta (A4) precursor-like protein 1 (APLP1), transcript variant 2, mRNA [NM_005166]	A_23_P27983	0.000846	0.0115	-3.209	-1.682
NPDC1	Homo sapiens neural proliferation, differentiation and control, 1 (NPDC1), mRNA [NM_015392]	A_23_P146572	0.000848	0.0115	-7.471	-2.901
ENST000002856	Homo sapiens MARVEL domain containing 1 (MARVELD1), mRNA [NM_031484]	A_23_P138725	0.000853	0.0115	-2.526	-1.337
OXR1	Homo sapiens oxidation resistance 1 (OXR1), transcript variant 4, mRNA [NM_001198533]	A_21_P0014793	0.000853	0.0115	-1.796	-0.845
AK2P2	Unknown	A_24_P358131	0.000858	0.0116	-2.544	-1.347
KIAA1586	Homo sapiens KIAA1586 (KIAA1586), mRNA [NM_020931]	A_24_P101047	0.000857	0.0116	-3.111	-1.637
ISPD	Homo sapiens isoprenoid synthase domain containing (ISPD), transcript variant 1, mRNA [NM_001101426]	A_33_P3362503	0.000861	0.0116	-2.668	-1.416
HSPA12B	Homo sapiens heat shock 70kD protein 12B (HSPA12B), transcript variant 1, mRNA [NM_052970]	A_23_P91334	0.000867	0.0116	-5.016	-2.327
CMIP	Homo sapiens c-Maf inducing protein (CMIP), transcript variant 1, mRNA [NM_198390]	A_23_P371239	0.000891	0.0118	-2.047	-1.033
LOC284219	Homo sapiens cDNA FLJ37117 fis, clone BRACE2022270. [AK094436]	A_33_P3527721	0.000911	0.012	-4.24	-2.084
PNPO	Homo sapiens pyridoxamine 5'-phosphate oxidase (PNPO), mRNA [NM_018129]	A_33_P3243175	0.000904	0.012	-2.121	-1.085
NCKIPSD	Homo sapiens NCK interacting protein with SH3 domain (NCKIPSD), transcript variant 2, mRNA [NM_184231]	A_23_P29638	0.000922	0.0121	-2.316	-1.212
SPN	Homo sapiens sialophorin (SPN), transcript variant 1, mRNA [NM_001030288]	A_33_P3404706	0.00092	0.0121	-2.5	-1.322
CLCNKA	Homo sapiens chloride channel Ka (CLCNKA), transcript variant 1, mRNA [NM_004070]	A_24_P2361	0.000923	0.0121	-2.126	-1.088
LMBRD2	Homo sapiens LMBR1 domain containing 2 (LMBRD2), mRNA [NM_001007527]	A_23_P327698	0.000936	0.0122	-2.214	-1.147
HAPLN3	Homo sapiens hyaluronan and proteoglycan link protein 3 (HAPLN3), mRNA [NM_178232]	A_33_P3259393	0.000935	0.0122	-2.978	-1.575
KIT	Homo sapiens v-kit Hardy-Zuckerman 4 feline sarcoma viral oncogene homolog (KIT), transcript variant 2, mRNA [NM_001093772]	A_33_P3303245	0.000926	0.0122	-6.331	-2.663
UBL4A	Homo sapiens ubiquitin-like 4A (UBL4A), mRNA [NM_014235]	A_33_P3232006	0.000935	0.0122	-2.141	-1.098

MXI1	Homo sapiens MAX interactor 1 (MXI1), transcript variant 1, mRNA [NM_005962]	A_33_P3263902	0.000933	0.0122	-4.573	-2.193
GNG8	Homo sapiens guanine nucleotide binding protein (G protein), gamma 8 (GNG8), mRNA [NM_033258]	A_24_P79070	0.000935	0.0122	-3.541	-1.824
LASS1	Homo sapiens ceramide synthase 1 (CERS1), transcript variant 2, mRNA [NM_198207]	A_33_P3408320	0.000946	0.0123	-3.079	-1.622
ENST000003666	tripartite motif containing 11 [Source:HGNC Symbol;Acc:16281] [ENST00000366699]	A_24_P50908	0.000952	0.0124	-2.022	-1.016
ENST000003949	Homo sapiens cDNA FLJ43630 fis, clone SPLEN2030479. [AK125618]	A_33_P3286002	0.000958	0.0124	-2.245	-1.167
SH3PXD2A	Homo sapiens SH3 and PX domains 2A (SH3PXD2A), mRNA [NM_014631]	A_23_P35456	0.000954	0.0124	-3.272	-1.71
E2F3	Homo sapiens E2F transcription factor 3 (E2F3), transcript variant 1, mRNA [NM_001949]	A_23_P385034	0.000965	0.0125	-1.935	-0.952
ENST000003690	TIA1 cytotoxic granule-associated RNA binding protein-like 1 [Source:HGNC Symbol;Acc:11804] [ENST00000369086]	A_33_P3293869	0.000962	0.0125	-4.107	-2.038
ZNRF1	Homo sapiens zinc and ring finger 1 (ZNRF1), mRNA [NM_032268]	A_24_P16610	0.000982	0.0126	-2.429	-1.281
PFKFB4	Homo sapiens 6-phosphofructo-2-kinase/fructose-2,6-biphosphatase 4 (PFKFB4), mRNA [NM_004567]	A_24_P362904	0.000974	0.0126	-4.115	-2.041
WNK4	Homo sapiens WNK lysine deficient protein kinase 4 (WNK4), mRNA [NM_032387]	A_24_P327181	0.000977	0.0126	-7.173	-2.842
ZMYM3	Homo sapiens zinc finger, MYM-type 3 (ZMYM3), transcript variant 2, mRNA [NM_005096]	A_24_P16815	0.00098	0.0126	-4.091	-2.033
STH	Homo sapiens saitohin (STH), mRNA [NM_001007532]	A_24_P256512	0.000986	0.0126	-5.325	-2.413
THC2494281	MPP8_HUMAN (Q99549) M-phase phosphoprotein 8, partial (48%) [THC2494281]	A_33_P3306973	0.000976	0.0126	-2.616	-1.387
EVI5L	Homo sapiens ecotropic viral integration site 5-like (EVI5L), transcript variant 2, mRNA [NM_145245]	A_33_P3250028	0.000976	0.0126	-3.183	-1.67
A_33_P3282213	Unknown	A_33_P3282213	0.000985	0.0126	-4.076	-2.027
CDC27	Homo sapiens cell division cycle 27 homolog (S. cerevisiae) (CDC27), transcript variant 2, mRNA [NM_001256]	A_23_P66777	0.000989	0.0127	-1.863	-0.897
RABGAP1L	Homo sapiens RAB GTPase activating protein 1-like (RABGAP1L), transcript variant 1, mRNA [NM_014857]	A_24_P49349	0.000993	0.0127	-2.379	-1.251
CAPRIN1	Homo sapiens cell cycle associated protein 1 (CAPRIN1), transcript variant 2, mRNA [NM_203364]	A_24_P902728	0.000988	0.0127	-1.91	-0.934
PTCD2	Homo sapiens pentatricopeptide repeat domain 2 (PTCD2), mRNA [NM_024754]	A_24_P191847	0.00101	0.0128	-2.225	-1.154
CFDP1	Homo sapiens craniofacial development protein 1 (CFDP1), mRNA [NM_006324]	A_23_P89123	0.00101	0.0128	-2.053	-1.038
CLIP3	Homo sapiens CAP-GLY domain containing linker protein 3 (CLIP3), transcript variant 2, mRNA [NM_015526]	A_23_P50786	0.00101	0.0128	-4.96	-2.31
CBX6	Homo sapiens chromobox homolog 6 (CBX6), mRNA [NM_014292]	A_33_P3270485	0.00101	0.0128	-2.608	-1.383
ZNF260	Homo sapiens zinc finger protein 260 (ZNF260), transcript variant 1, mRNA [NM_001012756]	A_33_P3414122	0.00101	0.0128	-1.693	-0.759
ST8SIA6	Homo sapiens ST8 alpha-N-acetyl-neuraminide alpha-2,8-sialyltransferase 6 (ST8SIA6), mRNA [NM_001004470]	A_33_P3403963	0.00102	0.0129	-2.059	-1.042
ZNF814	Homo sapiens zinc finger protein 814 (ZNF814), mRNA [NM_001144989]	A_33_P3228757	0.00102	0.0129	-2.03	-1.022

EPT1	Homo sapiens ethanolaminephosphotransferase 1 (CDP-ethanolamine-specific) (EPT1), mRNA [NM_033505]	A_33_P3230176	0.00102	0.0129	-3.565	-1.834
A_33_P3346526	Unknown	A_33_P3346526	0.00102	0.0129	-3.618	-1.855
ARFRP1	Homo sapiens ADP-ribosylation factor related protein 1 (ARFRP1), transcript variant 2, mRNA [NM_001134758]	A_33_P3242743	0.00102	0.0129	-2.208	-1.143
MARCH8	Homo sapiens membrane-associated ring finger (C3HC4) 8 (MARCH8), transcript variant 6, mRNA [NM_001002265]	A_23_P338495	0.00102	0.0129	-2.006	-1.004
HRC	Homo sapiens histidine rich calcium binding protein (HRC), mRNA [NM_002152]	A_23_P142125	0.00103	0.013	-8.211	-3.038
ZNF215	Homo sapiens zinc finger protein 215 (ZNF215), mRNA [NM_013250]	A_23_P53057	0.00104	0.0131	-2.026	-1.019
HIST1H2AM	Homo sapiens histone cluster 1, H2am (HIST1H2AM), mRNA [NM_003514]	A_32_P221799	0.00104	0.0131	-2.177	-1.122
PTPRH	Homo sapiens protein tyrosine phosphatase, receptor type, H (PTPRH), transcript variant 1, mRNA [NM_002842]	A_23_P101642	0.00104	0.0131	-2.04	-1.028
TMEM175	Homo sapiens transmembrane protein 175 (TMEM175), mRNA [NM_032326]	A_33_P3293266	0.00105	0.0132	-2.602	-1.379
ENST000003722	phosphatidic acid phosphatase type 2 domain containing 3 [Source:HGNC Symbol;Acc:28174] [ENST00000372261]	A_33_P3227079	0.00105	0.0132	-3.767	-1.913
LOC677759	full-length cDNA clone CS0DK001YC14 of HeLa cells Cot 25-normalized of Homo sapiens (human) [CR609725]	A_33_P3797820	0.00105	0.0132	-3.042	-1.605
SIPA1L3	Homo sapiens signal-induced proliferation-associated 1 like 3 (SIPA1L3), mRNA [NM_015073]	A_24_P202558	0.00105	0.0132	-3.276	-1.712
TAF8	TAF8 RNA polymerase II, TATA box binding protein (TBP)-associated factor, 43kDa [Source:HGNC Symbol;Acc:17300] [ENST00000372978]	A_24_P307974	0.00107	0.0133	-2.222	-1.152
ABCC5	Homo sapiens ATP-binding cassette, sub-family C (CFTR/MRP), member 5 (ABCC5), transcript variant 1, mRNA [NM_005688]	A_23_P258221	0.00107	0.0133	-2.178	-1.123
hCG_1745121	Homo sapiens isoprenoid synthase domain containing (ISPD), transcript variant 1, mRNA [NM_001101426]	A_24_P833129	0.00107	0.0133	-2.471	-1.305
DMAP1	Homo sapiens DNA methyltransferase 1 associated protein 1 (DMAP1), transcript variant 1, mRNA [NM_019100]	A_33_P3321417	0.00106	0.0133	-2.257	-1.174
MYL10	Homo sapiens myosin, light chain 10, regulatory (MYL10), mRNA [NM_138403]	A_33_P3369098	0.00106	0.0133	-8.414	-3.073
EFCAB4B	Homo sapiens EF-hand calcium binding domain 4B (EFCAB4B), transcript variant 3, mRNA [NM_032680]	A_23_P128351	0.00107	0.0133	-3.063	-1.615
ELAVL1	Homo sapiens ELAV (embryonic lethal, abnormal vision, Drosophila)-like 1 (Hu antigen R) (ELAVL1), mRNA [NM_001419]	A_23_P388681	0.00109	0.0134	-2.173	-1.12
FANCA	Homo sapiens Fanconi anemia, complementation group A (FANCA), transcript variant 1, mRNA [NM_000135]	A_23_P206441	0.00109	0.0134	-1.71	-0.774
ZHX3	Homo sapiens zinc fingers and homeoboxes 3 (ZHX3), mRNA [NM_015035]	A_33_P3342106	0.00108	0.0134	-2.467	-1.303
MCM10	Homo sapiens minichromosome maintenance complex component 10 (MCM10), transcript variant 1, mRNA [NM_182751]	A_33_P3867534	0.00109	0.0134	-4	-2
SP6	Homo sapiens Sp6 transcription factor (SP6), mRNA [NM_199262]	A_33_P3282075	0.00108	0.0134	-3.253	-1.702
TMEM132A	Homo sapiens transmembrane protein 132A (TMEM132A), transcript variant 1, mRNA [NM_017870]	A_23_P24716	0.00108	0.0134	-15.962	-3.997
HIST1H2AH	Homo sapiens histone cluster 1, H2ah (HIST1H2AH), mRNA [NM_080596]	A_23_P81859	0.0011	0.0135	-2.363	-1.24
ZBTB42	Homo sapiens zinc finger and BTB domain containing 42 (ZBTB42), mRNA [NM_001137601]	A_33_P3289128	0.0011	0.0135	-2.001	-1.001

C1orf159	chromosome 1 open reading frame 159 [Source:HGNC Symbol;Acc:26062] [ENST00000379319]	A_33_P3299081	0.0011	0.0135	-2.763	-1.466
LOC441426	PREDICTED: Homo sapiens hypothetical protein LOC100289528 (LOC100289528), mRNA [XM_002344257]	A_33_P3411637	0.0011	0.0135	-2.568	-1.361
PIK3C2B	Homo sapiens phosphoinositide-3-kinase, class 2, beta polypeptide (PIK3C2B), mRNA [NM_002646]	A_23_P200710	0.0011	0.0135	-3.656	-1.87
TNFRSF6B	Homo sapiens tumor necrosis factor receptor superfamily, member 6b, decoy (TNFRSF6B), mRNA [NM_003823]	A_23_P218646	0.00112	0.0136	-2.348	-1.231
LDB2	Homo sapiens LIM domain binding 2 (LDB2), transcript variant 1, mRNA [NM_001290]	A_23_P125253	0.00111	0.0136	-7.549	-2.916
KRT18P40	Unknown	A_24_P401601	0.00111	0.0136	-2.621	-1.39
CENPM	Homo sapiens centromere protein M (CENPM), transcript variant 1, mRNA [NM_024053]	A_33_P3387831	0.00111	0.0136	-3.575	-1.838
ANO9	Homo sapiens anoctamin 9 (ANO9), mRNA [NM_001012302]	A_24_P8109	0.00112	0.0136	-2.639	-1.4
ASTN2	Homo sapiens astrotactin 2 (ASTN2), transcript variant 4, mRNA [NM_198188]	A_33_P3371904	0.00113	0.0137	-2.01	-1.007
A_21_P0014392	Unknown	A_21_P0014392	0.00113	0.0137	-3.089	-1.627
FAM117B	Homo sapiens family with sequence similarity 117, member B (FAM117B), mRNA [NM_173511]	A_32_P195401	0.00113	0.0137	-1.498	-0.583
MTSS1L	Homo sapiens metastasis suppressor 1-like (MTSS1L), mRNA [NM_138383]	A_23_P403745	0.00114	0.0138	-4.495	-2.168
THC2553947	AF079557 poly(ADP-ribose) glycohydrolase {Mus musculus} (exp=-1; wgp=0; cg=0), partial (3%) [THC2553947]	A_33_P3223965	0.00114	0.0138	-2.456	-1.296
PACSIN1	Homo sapiens protein kinase C and casein kinase substrate in neurons 1 (PACSIN1), transcript variant 1, mRNA [NM_020804]	A_23_P258088	0.00114	0.0138	-21.871	-4.451
XYLB	Homo sapiens xylulokinase homolog (H. influenzae) (XYLB), mRNA [NM_005108]	A_23_P212423	0.00116	0.0139	-2.127	-1.089
FLJ11710	Homo sapiens cDNA FLJ11710 fis, clone HEMBA1005149. [AK021772]	A_33_P3710442	0.00115	0.0139	-4.871	-2.284
GYPA	Homo sapiens glycophorin A (MNS blood group) (GYPA), mRNA [NM_002099]	A_33_P3362933	0.00116	0.0139	-9.403	-3.233
MKI67	Homo sapiens antigen identified by monoclonal antibody Ki-67 (MKI67), transcript variant 1, mRNA [NM_002417]	A_24_P346855	0.00116	0.0139	-1.925	-0.945
SAC3D1	Homo sapiens SAC3 domain containing 1 (SAC3D1), mRNA [NM_013299]	A_23_P348298	0.00117	0.014	-2.38	-1.251
NHEDC1	Homo sapiens solute carrier family 9, subfamily B (cation proton antiporter 2), member 1 (SLC9B1), nuclear gene encoding mitochondrial protein, transcript variant 1, mRNA [NM_139173]	A_23_P415611	0.00117	0.014	-3.584	-1.842
S100A3	Homo sapiens S100 calcium binding protein A3 (S100A3), mRNA [NM_002960]	A_23_P104073	0.00117	0.014	-7.292	-2.866
RNF145	Homo sapiens ring finger protein 145 (RNF145), transcript variant 2, mRNA [NM_144726]	A_24_P144773	0.00117	0.014	-2.934	-1.553
STK11	Homo sapiens serine/threonine kinase 11 (STK11), mRNA [NM_000455]	A_33_P3389148	0.00118	0.0141	-2.075	-1.053
A_33_P3395403	Unknown	A_33_P3395403	0.0012	0.0142	-2.838	-1.505
PVT1	Q67VC5_ORYSA (Q67VC5) DeliriumA-like, partial (3%) [THC2536575]	A_21_P0005951	0.0012	0.0142	-3.82	-1.934
C1orf145	Homo sapiens chromosome 1 open reading frame 145, mRNA (cDNA clone IMAGE:5204063). [BC027909]	A_24_P15898	0.00122	0.0144	-3.457	-1.79
NAP1L5	Homo sapiens nucleosome assembly protein 1-like 5 (NAP1L5), mRNA [NM_153757]	A_23_P144369	0.00122	0.0144	-3.466	-1.793

FBLN2	Homo sapiens fibulin 2 (FBLN2), transcript variant 1, mRNA [NM_001004019]	A_23_P143981	0.00122	0.0144	-80.361	-6.328
TAF6L	Homo sapiens TAF6-like RNA polymerase II, p300/CBP-associated factor (PCAF)-associated factor, 65kDa (TAF6L), mRNA [NM_006473]	A_33_P3266080	0.00124	0.0145	-2.372	-1.246
REEP6	Homo sapiens receptor accessory protein 6 (REEP6), mRNA [NM_138393]	A_33_P3262789	0.00124	0.0145	-2.871	-1.522
USP31	Homo sapiens ubiquitin specific peptidase 31 (USP31), mRNA [NM_020718]	A_24_P390583	0.00125	0.0146	-3.066	-1.616
SLC4A11	Homo sapiens solute carrier family 4, sodium borate transporter, member 11 (SLC4A11), transcript variant 2, mRNA [NM_032034]	A_23_P154688	0.00125	0.0146	-2.865	-1.518
RNASE1	Homo sapiens ribonuclease, RNase A family, 1 (pancreatic) (RNASE1), transcript variant 3, mRNA [NM_198232]	A_23_P48596	0.00125	0.0146	-6.716	-2.748
LMTK3	Homo sapiens lemur tyrosine kinase 3 (LMTK3), mRNA [NM_001080434]	A_33_P3266928	0.00125	0.0146	-2.671	-1.418
ZNF497	Homo sapiens zinc finger protein 497 (ZNF497), mRNA [NM_198458]	A_33_P3323323	0.00126	0.0147	-2.364	-1.241
ENTPD2	Homo sapiens ectonucleoside triphosphate diphosphohydrolase 2 (ENTPD2), transcript variant 1, mRNA [NM_203468]	A_33_P3278649	0.00127	0.0147	-3.432	-1.779
COL6A1	Homo sapiens collagen, type VI, alpha 1 (COL6A1), mRNA [NM_001848]	A_32_P32254	0.00128	0.0148	-8.53	-3.093
TSEN54	Homo sapiens tRNA splicing endonuclease 54 homolog (S. cerevisiae) (TSEN54), mRNA [NM_207346]	A_23_P147277	0.00128	0.0148	-2.661	-1.412
PLEKHG5	Homo sapiens pleckstrin homology domain containing, family G (with RhoGef domain) member 5 (PLEKHG5), transcript variant 2, mRNA [NM_198681]	A_33_P3272539	0.00128	0.0148	-1.334	-0.416
ANKRD17	Homo sapiens ankyrin repeat domain 17 (ANKRD17), transcript variant 1, mRNA [NM_032217]	A_33_P3412538	0.00128	0.0148	-1.522	-0.606
RMND5B	Homo sapiens required for meiotic nuclear division 5 homolog B (S. cerevisiae) (RMND5B), mRNA [NM_022762]	A_33_P3258723	0.00131	0.015	-2.145	-1.101
PLCB3	Homo sapiens phospholipase C, beta 3 (phosphatidylinositol-specific) (PLCB3), transcript variant 1, mRNA [NM_000932]	A_33_P3260623	0.00131	0.015	-2.2	-1.137
CBX2	Homo sapiens chromobox homolog 2 (CBX2), transcript variant 1, mRNA [NM_005189]	A_33_P3423949	0.00131	0.015	-2.368	-1.244
ABCC4	Homo sapiens ATP-binding cassette, sub-family C (CFTR/MRP), member 4 (ABCC4), transcript variant 1, mRNA [NM_005845]	A_24_P16913	0.00131	0.015	-3.934	-1.976
TAC3	Homo sapiens tachykinin 3 (TAC3), transcript variant 1, mRNA [NM_013251]	A_23_P2283	0.0013	0.015	-9.844	-3.299
ANXA9	Homo sapiens annexin A9 (ANXA9), mRNA [NM_003568]	A_23_P103617	0.0013	0.015	-2.086	-1.061
MST1R	Homo sapiens macrophage stimulating 1 receptor (c-met-related tyrosine kinase) (MST1R), transcript variant 1, mRNA [NM_002447]	A_23_P256312	0.00131	0.015	-2.135	-1.094
CPA5	Homo sapiens carboxypeptidase A5 (CPA5), transcript variant 3, mRNA [NM_001127442]	A_33_P3228600	0.00132	0.0151	-2.743	-1.456
CCNB1IP1	Homo sapiens cyclin B1 interacting protein 1, E3 ubiquitin protein ligase (CCNB1IP1), transcript variant 3, mRNA [NM_182851]	A_23_P76882	0.00132	0.0151	-2.047	-1.034
LAGE3	Homo sapiens L antigen family, member 3 (LAGE3), mRNA [NM_006014]	A_23_P73763	0.00133	0.0152	-2.121	-1.085
BBS9	Homo sapiens Bardet-Biedl syndrome 9 (BBS9), transcript variant 1, mRNA [NM_014451]	A_33_P3360087	0.00133	0.0152	-2.146	-1.101
C6orf108	Homo sapiens chromosome 6 open reading frame 108 (C6orf108), transcript variant 2, mRNA [NM_199184]	A_33_P3372451	0.00134	0.0152	-2.095	-1.067
ANKRD20A2	Homo sapiens ankyrin repeat domain 20 family, member A2 (ANKRD20A2), mRNA [NM_001012421]	A_21_P0010770	0.00134	0.0152	-2.712	-1.439
RNF123	Homo sapiens ring finger protein 123 (RNF123), mRNA [NM_022064]	A_21_P0010755	0.00134	0.0152	-2.217	-1.148
HSD11B1L	Homo sapiens hydroxysteroid (11-beta) dehydrogenase 1-like (HSD11B1L), transcript variant b, mRNA [NM_198706]	A_23_P141992	0.00133	0.0152	-2.127	-1.088

AK128007	Homo sapiens cDNA FLJ46126 fis, clone TESTI2041362. [AK128007]	A_33_P3331031	0.00135	0.0153	-3.608	-1.851
ZMYM6	Homo sapiens zinc finger, MYM-type 6 (ZMYM6), mRNA [NM_007167]	A_23_P201845	0.00137	0.0154	-2.516	-1.331
C1orf113	SH3 domain containing 21 [Source:HGNC Symbol;Acc:26236] [ENST00000480549]	A_23_P307536	0.00137	0.0154	-2.451	-1.293
A_19_P00811234	Unknown	A_19_P00811234	0.00137	0.0154	-2.355	-1.236
CDC14A	Homo sapiens CDC14 cell division cycle 14 homolog A (S. cerevisiae) (CDC14A), transcript variant 3, mRNA [NM_033313]	A_23_P405110	0.00137	0.0154	-2.6	-1.378
SSBP2	Homo sapiens single-stranded DNA binding protein 2 (SSBP2), mRNA [NM_012446]	A_23_P33791	0.00138	0.0155	-2.141	-1.098
C3orf75	Homo sapiens chromosome 3 open reading frame 75 (C3orf75), mRNA [NM_001031703]	A_33_P3348313	0.00138	0.0155	-2.38	-1.251
PAQR4	Homo sapiens progestin and adipoQ receptor family member IV (PAQR4), mRNA [NM_152341]	A_23_P397341	0.00138	0.0155	-3.183	-1.67
CDKN1C	Homo sapiens cyclin-dependent kinase inhibitor 1C (p57, Kip2) (CDKN1C), transcript variant 1, mRNA [NM_000076]	A_23_P428129	0.00139	0.0156	-3.225	-1.689
PODNL1	Homo sapiens podocan-like 1 (PODNL1), transcript variant 1, mRNA [NM_024825]	A_23_P433798	0.0014	0.0157	-5.416	-2.437
ULBP1	Homo sapiens UL16 binding protein 1 (ULBP1), mRNA [NM_025218]	A_33_P3422802	0.0014	0.0157	-10.87	-3.442
NUDT8	Homo sapiens nudix (nucleoside diphosphate linked moiety X)-type motif 8 (NUDT8), transcript variant 1, mRNA [NM_001243750]	A_33_P3384932	0.0014	0.0157	-4.64	-2.214
HIST2H3A	Homo sapiens histone cluster 2, H3a (HIST2H3A), mRNA [NM_001005464]	A_33_P3257678	0.00141	0.0157	-2.589	-1.372
SLC9B1	Homo sapiens solute carrier family 9, subfamily B (cation proton antiporter 2), member 1 (SLC9B1), nuclear gene encoding mitochondrial protein, transcript variant 1, mRNA [NM_139173]	A_21_P0010939	0.0014	0.0157	-4.768	-2.254
BCL7A	Homo sapiens B-cell CLL/lymphoma 7A (BCL7A), transcript variant 1, mRNA [NM_020993]	A_24_P203056	0.0014	0.0157	-3.312	-1.728
HIST1H2AK	Homo sapiens histone cluster 1, H2ak (HIST1H2AK), mRNA [NM_003510]	A_24_P217848	0.00143	0.0158	-2.215	-1.148
HIST1H3J	Homo sapiens histone cluster 1, H3j (HIST1H3J), mRNA [NM_003535]	A_23_P93282	0.00143	0.0158	-2.268	-1.182
HN1L	Homo sapiens hematological and neurological expressed 1-like (HN1L), mRNA [NM_144570]	A_23_P434900	0.00143	0.0158	-1.99	-0.993
ENST000004894	phosphoribosyl pyrophosphate synthetase 2 [Source:HGNC Symbol;Acc:9465] [ENST00000489404]	A_33_P3411122	0.00143	0.0158	-2.61	-1.384
ENST000004096	polymerase (RNA) II (DNA directed) polypeptide D [Source:HGNC Symbol;Acc:9191] [ENST00000409698]	A_33_P3351811	0.00142	0.0158	-2.152	-1.106
LOC644919	human full-length cDNA clone CS0DC023YN15 of Neuroblastoma of Homo sapiens (human). [BX248273]	A_33_P3576034	0.00144	0.0159	-5.941	-2.571
SREBF1	Homo sapiens sterol regulatory element binding transcription factor 1 (SREBF1), transcript variant 1, mRNA [NM_001005291]	A_23_P129786	0.00146	0.016	-2.444	-1.289
ZNF321	Homo sapiens ZNF816-ZNF321P readthrough (ZNF816-ZNF321P), mRNA [NM_001202473]	A_33_P3220113	0.00145	0.016	-2	-1
HIST1H2BF	Homo sapiens histone cluster 1, H2bf (HIST1H2BF), mRNA [NM_003522]	A_33_P3229122	0.00145	0.016	-3.254	-1.702
CBFA2T3	Homo sapiens core-binding factor, runt domain, alpha subunit 2; translocated to, 3 (CBFA2T3), transcript variant 1, mRNA [NM_005187]	A_23_P500741	0.00145	0.016	-4.698	-2.232
LMBR1	Homo sapiens limb region 1 homolog (mouse) (LMBR1), mRNA [NM_022458]	A_23_P215214	0.00147	0.0161	-2.203	-1.14
ZNF362	Homo sapiens zinc finger protein 362 (ZNF362), mRNA [NM_152493]	A_23_P137543	0.00147	0.0161	-3.019	-1.594

KLK13	Homo sapiens kallikrein-related peptidase 13 (KLK13), mRNA [NM_015596]	A_24_P416645	0.00148	0.0162	-2.903	-1.537
SIGIRR	Homo sapiens single immunoglobulin and toll-interleukin 1 receptor (TIR) domain (SIGIRR), transcript variant 3, mRNA [NM_021805]	A_23_P84344	0.00149	0.0162	-1.876	-0.907
LOC727927	Unknown	A_33_P3414022	0.00149	0.0162	-5.281	-2.401
KIAA1984	Homo sapiens KIAA1984 (KIAA1984), mRNA [NM_001039374]	A_33_P3326730	0.00149	0.0162	-3.363	-1.75
LAG3	Homo sapiens lymphocyte-activation gene 3 (LAG3), mRNA [NM_002286]	A_23_P116942	0.00148	0.0162	-2.164	-1.114
CD2AP	Homo sapiens CD2-associated protein (CD2AP), mRNA [NM_012120]	A_32_P155811	0.00148	0.0162	-2.959	-1.565
ZFAT	Homo sapiens zinc finger and AT hook domain containing (ZFAT), transcript variant 1, mRNA [NM_020863]	A_32_P109922	0.0015	0.0163	-1.541	-0.624
ETHE1	Homo sapiens ethylmalonic encephalopathy 1 (ETHE1), nuclear gene encoding mitochondrial protein, mRNA [NM_014297]	A_23_P142294	0.0015	0.0163	-2.312	-1.209
SLC2A11	Homo sapiens solute carrier family 2 (facilitated glucose transporter), member 11 (SLC2A11), transcript variant 1, mRNA [NM_030807]	A_33_P3268649	0.00151	0.0163	-2.156	-1.108
ENHO	Homo sapiens energy homeostasis associated (ENHO), mRNA [NM_198573]	A_33_P3417626	0.0015	0.0163	-6.368	-2.671
POLR3H	Homo sapiens polymerase (RNA) III (DNA directed) polypeptide H (22.9kD) (POLR3H), transcript variant 1, mRNA [NM_138338]	A_24_P322847	0.0015	0.0163	-2.08	-1.057
ST3GAL5	Homo sapiens ST3 beta-galactoside alpha-2,3-sialyltransferase 5 (ST3GAL5), transcript variant 1, mRNA [NM_003896]	A_23_P136573	0.00151	0.0163	-8.109	-3.02
IGFBP4	Homo sapiens insulin-like growth factor binding protein 4 (IGFBP4), mRNA [NM_001552]	A_24_P382187	0.0015	0.0163	-2.875	-1.524
ZAP70	Homo sapiens zeta-chain (TCR) associated protein kinase 70kDa (ZAP70), transcript variant 1, mRNA [NM_001079]	A_24_P169234	0.00152	0.0164	-5.812	-2.539
S100A1	Homo sapiens S100 calcium binding protein A1 (S100A1), mRNA [NM_006271]	A_23_P383227	0.00152	0.0164	-16.459	-4.041
ABHD11	Homo sapiens abhydrolase domain containing 11 (ABHD11), nuclear gene encoding mitochondrial protein, transcript variant 1, mRNA [NM_148912]	A_23_P315933	0.00154	0.0165	-2.346	-1.23
ZNF469	Homo sapiens zinc finger protein 469 (ZNF469), mRNA [NM_001127464]	A_32_P116556	0.00153	0.0165	-5.948	-2.572
MACROD1	Homo sapiens MACRO domain containing 1 (MACROD1), mRNA [NM_014067]	A_23_P86855	0.00153	0.0165	-2.394	-1.259
AGPAT5	Homo sapiens 1-acylglycerol-3-phosphate O-acyltransferase 5 (lysophosphatidic acid acyltransferase, epsilon) (AGPAT5), mRNA [NM_018361]	A_33_P3217218	0.00154	0.0165	-3.461	-1.791
LOC100131594	Homo sapiens cDNA FLJ41314 fis, clone BRAMY2042918. [AK123308]	A_33_P3395428	0.00154	0.0165	-3.551	-1.828
LOC643988	Homo sapiens uncharacterized LOC643988 (LOC643988), mRNA [NM_001242659]	A_33_P3368049	0.00155	0.0166	-2.479	-1.31
MAPT	Homo sapiens microtubule-associated protein tau (MAPT), transcript variant 6, mRNA [NM_001123066]	A_21_P0014031	0.00155	0.0166	-2.736	-1.452
MAX	Homo sapiens MYC associated factor X (MAX), transcript variant 6, mRNA [NM_197957]	A_23_P151662	0.00156	0.0167	-2.779	-1.475
TMOD1	Homo sapiens tropomodulin 1 (TMOD1), transcript variant 1, mRNA [NM_003275]	A_23_P112289	0.00158	0.0168	-5.785	-2.532
LCK	Homo sapiens lymphocyte-specific protein tyrosine kinase (LCK), transcript variant 2, mRNA [NM_005356]	A_23_P103361	0.00157	0.0168	-4.307	-2.107
ZNF780A	Homo sapiens zinc finger protein 780A (ZNF780A), transcript variant 2, mRNA [NM_001010880]	A_23_P320530	0.0016	0.0169	-2.253	-1.172

SEPT5	Homo sapiens glycoprotein Ib (platelet), beta polypeptide (GP1BB), mRNA [NM_000407]	A_23_P29124	0.00159	0.0169	-3.432	-1.779
PRR5	Homo sapiens proline rich 5 (renal) (PRR5), transcript variant 2, mRNA [NM_015366]	A_23_P80382	0.0016	0.0169	-3.621	-1.856
RREB1	Homo sapiens ras responsive element binding protein 1 (RREB1), transcript variant 4, mRNA [NM_001003700]	A_33_P3345623	0.00159	0.0169	-2.517	-1.332
ZFPM1	Homo sapiens zinc finger protein, multitype 1 (ZFPM1), mRNA [NM_153813]	A_33_P3311979	0.00159	0.0169	-3.823	-1.935
CCDC7	Homo sapiens coiled-coil domain containing 7 (CCDC7), transcript variant 1, mRNA [NM_145023]	A_23_P374294	0.00161	0.017	-4.132	-2.047
A_33_P3356637	Unknown	A_33_P3356637	0.00162	0.0171	-2.834	-1.503
S100A16	Homo sapiens S100 calcium binding protein A16 (S100A16), mRNA [NM_080388]	A_23_P147918	0.00164	0.0172	-7.176	-2.843
EGFL7	Homo sapiens EGF-like-domain, multiple 7 (EGFL7), transcript variant 2, mRNA [NM_201446]	A_32_P210642	0.00163	0.0172	-2.815	-1.493
A_19_P0080840{	Homo sapiens methyl-CpG binding domain protein 5 (MBD5), mRNA [NM_018328]	A_19_P00808408	0.00166	0.0173	-3.031	-1.6
LOC100128009	Unknown	A_33_P3287084	0.00165	0.0173	-2.189	-1.13
CCRL2	Homo sapiens chemokine (C-C motif) receptor-like 2 (CCRL2), transcript variant 1, mRNA [NM_003965]	A_23_P69310	0.00165	0.0173	-2.234	-1.16
THC2511130	Unknown	A_33_P3248035	0.00167	0.0174	-2.757	-1.463
PTRF	Homo sapiens polymerase I and transcript release factor (PTRF), mRNA [NM_012232]	A_23_P394064	0.00168	0.0175	-2.062	-1.044
BEX4	Homo sapiens brain expressed, X-linked 4 (BEX4), transcript variant 2, mRNA [NM_001080425]	A_24_P40551	0.0017	0.0176	-2.124	-1.087
RBM4B	Homo sapiens RNA binding motif protein 4B (RBM4B), mRNA [NM_031492]	A_23_P1638	0.0017	0.0176	-2.114	-1.08
LOC100128519	Q7T2T6_BOTJR (Q7T2T6) Ribosomal protein, partial (70%) [THC2506656]	A_32_P159535	0.0017	0.0176	-2.633	-1.397
ENST000003693	Homo sapiens mRNA for FLJ00310 protein. [AK090412]	A_24_P187056	0.0017	0.0176	-3.5	-1.807
ENST000003793	BC030699 SHC SH2-domain binding protein 1 {Homo sapiens} (exp=-1; wgp=0; cg=0), partial (14%) [THC2657541]	A_32_P370026	0.00169	0.0176	-2.158	-1.109
RBM3	Homo sapiens RNA binding motif (RNP1, RRM) protein 3 (RBM3), mRNA [NM_006743]	A_23_P148308	0.0017	0.0176	-2.263	-1.178
UROD	Homo sapiens uroporphyrinogen decarboxylase (UROD), transcript variant 1, mRNA [NM_000374]	A_23_P63050	0.00171	0.0177	-3.531	-1.82
SLC12A4	Homo sapiens solute carrier family 12 (potassium/chloride transporters), member 4 (SLC12A4), transcript variant 1, mRNA [NM_005072]	A_23_P389391	0.00171	0.0177	-2.015	-1.011
RASA3	Homo sapiens RAS p21 protein activator 3 (RASA3), mRNA [NM_007368]	A_33_P3262515	0.00171	0.0177	-2.017	-1.012
ABCB6	Homo sapiens ATP-binding cassette, sub-family B (MDR/TAP), member 6 (ABCB6), nuclear gene encoding mitochondrial protein, mRNA [NM_005689]	A_23_P5441	0.00173	0.0178	-4.661	-2.221
DUSP3	Homo sapiens dual specificity phosphatase 3 (DUSP3), mRNA [NM_004090]	A_23_P129956	0.00173	0.0178	-2.017	-1.012
C16orf62	Homo sapiens chromosome 16 open reading frame 62 (C16orf62), mRNA [NM_020314]	A_33_P3318122	0.00174	0.0178	-2.013	-1.01
PNPLA7	Homo sapiens patatin-like phospholipase domain containing 7 (PNPLA7), transcript variant 1, mRNA [NM_001098537]	A_33_P3666884	0.00172	0.0178	-2.246	-1.167
THC2717143	HS1188J21 FSH primary response (LRPR1 homolog, rat) 1 {Homo sapiens} (exp=0; wgp=1; cg=0), partial (35%) [THC2717143]	A_33_P3255597	0.00174	0.0178	-2.595	-1.376

CPT1A	Homo sapiens carnitine palmitoyltransferase 1A (liver) (CPT1A), nuclear gene encoding mitochondrial protein, transcript variant 2, mRNA [NM_001031847]	A_23_P104563	0.00173	0.0178	-5.048	-2.336
GNPTAB	Homo sapiens N-acetylglucosamine-1-phosphate transferase, alpha and beta subunits (GNPTAB), mRNA [NM_024312]	A_24_P281975	0.00172	0.0178	-1.787	-0.838
APEX2	Homo sapiens APEX nuclease (apurinic/apyrimidinic endonuclease) 2 (APEX2), nuclear gene encoding mitochondrial protein, mRNA [NM_014481]	A_23_P256682	0.00172	0.0178	-2.295	-1.198
SIRT4	Homo sapiens sirtuin 4 (SIRT4), mRNA [NM_012240]	A_23_P99226	0.00175	0.0179	-2.047	-1.034
C9orf30	Homo sapiens chromosome 9 open reading frame 30 (C9orf30), transcript variant 4, mRNA [NM_001198807]	A_33_P3380056	0.00175	0.0179	-2.209	-1.144
SCARF1	Homo sapiens scavenger receptor class F, member 1 (SCARF1), transcript variant 5, mRNA [NM_145352]	A_23_P15414	0.00176	0.018	-3.038	-1.603
NAG	Homo sapiens neuroblastoma amplified sequence (NBAS), mRNA [NM_015909]	A_23_P332509	0.00178	0.0181	-2.095	-1.067
YIF1B	Homo sapiens Yip1 interacting factor homolog B (S. cerevisiae) (YIF1B), transcript variant 2, mRNA [NM_033557]	A_23_P153628	0.00178	0.0181	-2.407	-1.267
CIB2	Homo sapiens calcium and integrin binding family member 2 (CIB2), mRNA [NM_006383]	A_33_P3308914	0.00177	0.0181	-3.639	-1.863
B4GALNT1	Homo sapiens beta-1,4-N-acetyl-galactosaminyl transferase 1 (B4GALNT1), mRNA [NM_001478]	A_33_P3846177	0.00177	0.0181	-3.65	-1.868
HOXB9	Homo sapiens homeobox B9 (HOXB9), mRNA [NM_024017]	A_23_P27013	0.0018	0.0183	-2.258	-1.175
ACCN4	Homo sapiens amiloride-sensitive cation channel 4, pituitary (ACCN4), transcript variant 1, mRNA [NM_018674]	A_33_P3250730	0.00181	0.0183	-12.548	-3.649
ZNF248	Homo sapiens zinc finger protein 248 (ZNF248), mRNA [NM_021045]	A_33_P3243717	0.00181	0.0183	-2.536	-1.343
FAM65A	Homo sapiens family with sequence similarity 65, member A (FAM65A), transcript variant 1, mRNA [NM_024519]	A_32_P230868	0.00182	0.0184	-2.618	-1.388
CREB3L2	Homo sapiens cAMP responsive element binding protein 3-like 2 (CREB3L2), mRNA [NM_194071]	A_33_P3320888	0.00183	0.0184	-1.926	-0.945
MAPK11	Homo sapiens mitogen-activated protein kinase 11 (MAPK11), mRNA [NM_002751]	A_33_P3409513	0.00182	0.0184	-2.604	-1.381
ENST000003933	carnitine O-acetyltransferase [Source:HGNC Symbol;Acc:2342] [ENST00000393384]	A_33_P3415843	0.00182	0.0184	-2.398	-1.262
ICAM2	Homo sapiens intercellular adhesion molecule 2 (ICAM2), transcript variant 5, mRNA [NM_000873]	A_23_P152655	0.00183	0.0184	-10.726	-3.423
SLC25A42	Homo sapiens solute carrier family 25, member 42 (SLC25A42), mRNA [NM_178526]	A_23_P436526	0.00184	0.0185	-2.362	-1.24
PIM2	Homo sapiens pim-2 oncogene (PIM2), mRNA [NM_006875]	A_24_P379104	0.00185	0.0186	-2.024	-1.017
RNF208	Homo sapiens ring finger protein 208 (RNF208), mRNA [NM_031297]	A_33_P3379669	0.00186	0.0186	-1.846	-0.885
PTGDS	Homo sapiens prostaglandin D2 synthase 21kDa (brain) (PTGDS), mRNA [NM_000954]	A_33_P3298159	0.00185	0.0186	-2.29	-1.195
ENST000003587	histone cluster 1, H2ai [Source:HGNC Symbol;Acc:4725] [ENST00000358739]	A_33_P3393135	0.00185	0.0186	-3.055	-1.611
FUBP3	Homo sapiens far upstream element (FUSE) binding protein 3 (FUBP3), mRNA [NM_003934]	A_24_P203710	0.00187	0.0187	-1.441	-0.527
TSC2	Homo sapiens tuberous sclerosis 2 (TSC2), transcript variant 1, mRNA [NM_000548]	A_23_P66110	0.00187	0.0187	-1.581	-0.66
CGN	Homo sapiens cingulin (CGN), mRNA [NM_020770]	A_33_P3329974	0.00187	0.0187	-3.698	-1.887

ENST000004021	TATA box binding protein (TBP)-associated factor, RNA polymerase I, B, 63kDa [Source:HGNC Symbol;Acc:11533] [ENST00000402170]	A_33_P3392802	0.00187	0.0187	-3.648	-1.867
MPP1	Homo sapiens membrane protein, palmitoylated 1, 55kDa (MPP1), transcript variant 1, mRNA [NM_002436]	A_23_P171296	0.00187	0.0187	-1.855	-0.891
LTBP3	Homo sapiens latent transforming growth factor beta binding protein 3 (LTBP3), transcript variant 2, mRNA [NM_021070]	A_24_P298360	0.00189	0.0188	-2.453	-1.295
SPC25	Homo sapiens SPC25, NDC80 kinetochore complex component, homolog (S. cerevisiae) (SPC25), mRNA [NM_020675]	A_23_P51085	0.00189	0.0188	-2.005	-1.004
RAB37	Homo sapiens RAB37, member RAS oncogene family (RAB37), transcript variant 3, mRNA [NM_175738]	A_23_P414654	0.00188	0.0188	-5.221	-2.384
EHD2	Homo sapiens EH-domain containing 2 (EHD2), mRNA [NM_014601]	A_24_P156113	0.00192	0.019	-3.83	-1.938
RHD	Homo sapiens Rh blood group, D antigen (RHD), transcript variant 1, mRNA [NM_016124]	A_24_P673333	0.00191	0.019	-7.271	-2.862
IARS	Homo sapiens isoleucyl-tRNA synthetase (IARS), transcript variant long, mRNA [NM_013417]	A_33_P3209346	0.00191	0.019	-4.02	-2.007
B3GNT4	Homo sapiens UDP-GlcNAc:betaGal beta-1,3-N-acetylglucosaminyltransferase 4 (B3GNT4), mRNA [NM_030765]	A_23_P76071	0.00193	0.0191	-3.163	-1.661
AHDC1	Homo sapiens AT hook, DNA binding motif, containing 1 (AHDC1), mRNA [NM_001029882]	A_23_P115331	0.00193	0.0191	-4.445	-2.152
KIF19	Homo sapiens kinesin family member 19 (KIF19), mRNA [NM_153209]	A_32_P100830	0.00194	0.0191	-4.764	-2.252
AX748312	Homo sapiens cDNA FLJ36480 fis, clone THYMU2017449. [AK093799]	A_33_P3416127	0.00193	0.0191	-2.614	-1.386
VAPB	Homo sapiens VAMP (vesicle-associated membrane protein)-associated protein B and C (VAPB), transcript variant 1, mRNA [NM_004738]	A_33_P3365586	0.00193	0.0191	-3.33	-1.735
ENST000003402	Unknown	A_33_P3340454	0.00193	0.0191	-3.642	-1.865
CLCN2	Homo sapiens chloride channel 2 (CLCN2), transcript variant 1, mRNA [NM_004366]	A_21_P0000083	0.00194	0.0191	-2.217	-1.148
ENST000003707	zinc finger protein 451 [Source:HGNC Symbol;Acc:21091] [ENST00000370702]	A_33_P3257533	0.00195	0.0192	-5.502	-2.46
MAP1B	Homo sapiens microtubule-associated protein 1B (MAP1B), mRNA [NM_005909]	A_24_P879740	0.00195	0.0192	-3.856	-1.947
NCLN	Homo sapiens nicalin (NCLN), mRNA [NM_020170]	A_24_P339664	0.00197	0.0193	-2.05	-1.035
THOC6	Homo sapiens THO complex 6 homolog (Drosophila) (THOC6), transcript variant 1, mRNA [NM_024339]	A_23_P37949	0.00197	0.0193	-2.039	-1.028
SBK1	Homo sapiens SH3-binding domain kinase 1 (SBK1), mRNA [NM_001024401]	A_33_P3366336	0.00197	0.0193	-5.741	-2.521
MTA2	Homo sapiens metastasis associated 1 family, member 2 (MTA2), mRNA [NM_004739]	A_23_P203420	0.00197	0.0193	-2	-1
SLC25A29	Homo sapiens solute carrier family 25, member 29 (SLC25A29), nuclear gene encoding mitochondrial protein, mRNA [NM_001039355]	A_23_P77048	0.00198	0.0194	-2.135	-1.094
ATP8B3	Homo sapiens ATPase, aminophospholipid transporter, class I, type 8B, member 3 (ATP8B3), transcript variant 1, mRNA [NM_138813]	A_33_P3266646	0.00199	0.0194	-4.503	-2.171
C7orf41	Homo sapiens chromosome 7 open reading frame 41 (C7orf41), mRNA [NM_152793]	A_33_P3247644	0.00198	0.0194	-2.23	-1.157
CKB	Homo sapiens creatine kinase, brain (CKB), mRNA [NM_001823]	A_23_P25674	0.00199	0.0194	-3.628	-1.859
CXXC5	Homo sapiens CXXC finger protein 5 (CXXC5), mRNA [NM_016463]	A_23_P399001	0.00201	0.0195	-5.404	-2.434

SLC35F3	Homo sapiens solute carrier family 35, member F3 (SLC35F3), mRNA [NM_173508]	A_23_P422212	0.00202	0.0196	-6.443	-2.688
NFKBIL2	Homo sapiens tonsoku-like, DNA repair protein (TONSL), mRNA [NM_013432]	A_23_P216355	0.00202	0.0196	-2.263	-1.178
DNAJC17	Homo sapiens DnaJ (Hsp40) homolog, subfamily C, member 17 (DNAJC17), mRNA [NM_018163]	A_23_P140698	0.00202	0.0196	-2.286	-1.193
JAK2	Homo sapiens Janus kinase 2 (JAK2), mRNA [NM_004972]	A_33_P3878772	0.00202	0.0196	-2.545	-1.348
ZNF566	Homo sapiens zinc finger protein 566 (ZNF566), transcript variant 3, mRNA [NM_032838]	A_23_P306956	0.00204	0.0197	-4.032	-2.011
FOXO4	Homo sapiens forkhead box O4 (FOXO4), transcript variant 1, mRNA [NM_005938]	A_33_P3249793	0.00204	0.0197	-2.895	-1.534
C8G	Homo sapiens complement component 8, gamma polypeptide (C8G), mRNA [NM_000606]	A_33_P3319006	0.00204	0.0197	-1.804	-0.851
A_33_P3212839	Unknown	A_33_P3212839	0.00203	0.0197	-2.167	-1.116
ALDH4A1	Homo sapiens aldehyde dehydrogenase 4 family, member A1 (ALDH4A1), nuclear gene encoding mitochondrial protein, transcript variant P5CDhL, mRNA [NM_003748]	A_23_P170337	0.00205	0.0198	-3.256	-1.703
PIM1	Homo sapiens pim-1 oncogene (PIM1), transcript variant 1, mRNA [NM_002648]	A_23_P345118	0.00207	0.0199	-3.637	-1.863
STARD8	Homo sapiens StAR-related lipid transfer (START) domain containing 8 (STARD8), transcript variant 2, mRNA [NM_014725]	A_23_P387630	0.00207	0.0199	-3.244	-1.698
ACTL6B	Homo sapiens transferrin receptor 2 (TFR2), transcript variant 1, mRNA [NM_003227]	A_23_P122896	0.00207	0.0199	-3.892	-1.961
LYG2	Homo sapiens lysozyme G-like 2 (LYG2), mRNA [NM_175735]	A_23_P5703	0.00209	0.02	-5.072	-2.343
KCNK6	Homo sapiens potassium channel, subfamily K, member 6 (KCNK6), mRNA [NM_004823]	A_23_P50591	0.00209	0.02	-3.929	-1.974
MURC	Homo sapiens muscle-related coiled-coil protein (MURC), mRNA [NM_001018116]	A_33_P3335147	0.00211	0.0201	-2.623	-1.391
DCUN1D2	Homo sapiens DCN1, defective in cullin neddylation 1, domain containing 2 (S. cerevisiae) (DCUN1D2), mRNA [NM_001014283]	A_33_P3300346	0.0021	0.0201	-2.56	-1.356
SYT12	Homo sapiens synaptotagmin XII (SYT12), transcript variant 1, mRNA [NM_177963]	A_23_P421306	0.00213	0.0202	-4.301	-2.105
A_33_P3349002	Unknown	A_33_P3349002	0.00213	0.0202	-4.33	-2.114
TSPAN4	Homo sapiens tetraspanin 4 (TSPAN4), transcript variant 1, mRNA [NM_001025237]	A_33_P3413216	0.00213	0.0202	-2.014	-1.01
RAB3A	Homo sapiens RAB3A, member RAS oncogene family (RAB3A), mRNA [NM_002866]	A_33_P3314550	0.00212	0.0202	-6.599	-2.722
HSPG2	Homo sapiens heparan sulfate proteoglycan 2 (HSPG2), mRNA [NM_005529]	A_33_P3380618	0.00213	0.0202	-2.004	-1.003
TRIM7	Homo sapiens tripartite motif containing 7 (TRIM7), transcript variant 6, mRNA [NM_033342]	A_23_P30315	0.00214	0.0203	-3.376	-1.755
PYCR1	Homo sapiens pyrroline-5-carboxylate reductase 1 (PYCR1), transcript variant 1, mRNA [NM_006907]	A_23_P130194	0.00215	0.0203	-2.118	-1.083
HOXC13	Homo sapiens homeobox C13 (HOXC13), mRNA [NM_017410]	A_23_P64808	0.00215	0.0203	-3.363	-1.75
MYB	v-myb myeloblastosis viral oncogene homolog (avian) [Source:HGNC Symbol;Acc:7545] [ENST00000528345]	A_33_P3311795	0.00215	0.0203	-2.928	-1.55

TMPO	Homo sapiens thymopoietin (TMPO), transcript variant 2, mRNA [NM_001032283]	A_33_P3412613	0.00215	0.0203	-2.81	-1.491
EXOSC2	Homo sapiens exosome component 2 (EXOSC2), mRNA [NM_014285]	A_23_P216396	0.00217	0.0204	-2.001	-1.001
FAM152B	Homo sapiens PPPDE peptidase domain containing 2 (PPPDE2), mRNA [NM_015704]	A_23_P356330	0.00217	0.0204	-2.353	-1.235
CASP2	Homo sapiens caspase 2, apoptosis-related cysteine peptidase (CASP2), transcript variant 1, mRNA [NM_032982]	A_23_P387943	0.00216	0.0204	-1.86	-0.895
TUBGCP3	Homo sapiens tubulin, gamma complex associated protein 3 (TUBGCP3), mRNA [NM_006322]	A_23_P76705	0.00218	0.0205	-2.569	-1.361
TAF1	Homo sapiens TAF1 RNA polymerase II, TATA box binding protein (TBP)-associated factor, 250kDa (TAF1), transcript variant 1, mRNA [NM_004606]	A_23_P11237	0.00218	0.0205	-2.019	-1.014
A_19_P0031950[illegible]	Unknown	A_19_P00319503	0.00219	0.0206	-4.446	-2.153
LFNG	Homo sapiens LFNG O-fucosylpeptide 3-beta-N-acetylglucosaminyltransferase (LFNG), transcript variant 2, mRNA [NM_001040168]	A_23_P434518	0.00219	0.0206	-2.153	-1.106
SNN	Homo sapiens stannin (SNN), mRNA [NM_003498]	A_24_P30923	0.00221	0.0206	-2.592	-1.374
UNQ3118	Homo sapiens clone DNA86575 GRTR3118 (UNQ3118) mRNA, complete cds. [AY358225]	A_33_P3225610	0.0022	0.0206	-2.085	-1.06
AK094801	Homo sapiens cDNA FLJ37482 fis, clone BRAWH2013941. [AK094801]	A_33_P3214785	0.00221	0.0206	-2.736	-1.452
OCIAD2	Homo sapiens OCIA domain containing 2 (OCIAD2), transcript variant 1, mRNA [NM_001014446]	A_23_P121702	0.0022	0.0206	-4.431	-2.148
KLRG1	Homo sapiens killer cell lectin-like receptor subfamily G, member 1 (KLRG1), mRNA [NM_005810]	A_23_P64898	0.0022	0.0206	-2.312	-1.209
FGFR3	Homo sapiens fibroblast growth factor receptor 3 (FGFR3), transcript variant 1, mRNA [NM_000142]	A_23_P500501	0.0022	0.0206	-4.559	-2.189
ATP7A	Homo sapiens ATPase, Cu++ transporting, alpha polypeptide (ATP7A), mRNA [NM_000052]	A_33_P3327772	0.00222	0.0207	-1.561	-0.643
A_33_P3366321	Unknown	A_33_P3366321	0.00222	0.0207	-3.271	-1.71
LAMP3	Homo sapiens lysosomal-associated membrane protein 3 (LAMP3), mRNA [NM_014398]	A_23_P29773	0.00223	0.0207	-3.093	-1.629
RFC3	Homo sapiens replication factor C (activator 1) 3, 38kDa (RFC3), transcript variant 1, mRNA [NM_002915]	A_23_P14193	0.00224	0.0208	-2.061	-1.043
TSTA3	Homo sapiens tissue specific transplantation antigen P35B (TSTA3), mRNA [NM_003313]	A_23_P94301	0.00225	0.0208	-2.397	-1.261
LOC441528	HCG1981372, isoform CRA_cNovel proteinUncharacterized protein [Source:UniProtKB/TrEMBL;Acc:B1B108] [ENST00000483918]	A_33_P3252326	0.00225	0.0208	-3.545	-1.826
LOC402509	Unknown	A_33_P3386019	0.00224	0.0208	-4.746	-2.247
CROCC	Homo sapiens ciliary rootlet coiled-coil, rootletin (CROCC), mRNA [NM_014675]	A_33_P3663705	0.00224	0.0208	-2.248	-1.169
LOC644992	Unknown	A_33_P3329352	0.00225	0.0208	-2.059	-1.042
MFGE8	Homo sapiens milk fat globule-EGF factor 8 protein (MFGE8), transcript variant 1, mRNA [NM_005928]	A_24_P133584	0.00224	0.0208	-2.785	-1.478
RASSF5	Homo sapiens Ras association (RalGDS/AF-6) domain family member 5 (RASSF5), transcript variant 1, mRNA [NM_182663]	A_24_P171268	0.00226	0.0209	-1.752	-0.809
GOLIM4	Homo sapiens golgi integral membrane protein 4 (GOLIM4), mRNA [NM_014498]	A_24_P191664	0.00228	0.021	-2.818	-1.495

MYO19	Homo sapiens myosin XIX (MYO19), transcript variant 3, mRNA [NM_001033580]	A_23_P100868	0.00229	0.0211	-2.748	-1.458
SLC43A1	Homo sapiens solute carrier family 43, member 1 (SLC43A1), transcript variant 1, mRNA [NM_003627]	A_23_P52939	0.00231	0.0212	-2.777	-1.473
PHF14	Homo sapiens PHD finger protein 14 (PHF14), transcript variant 2, mRNA [NM_014660]	A_23_P134384	0.00231	0.0212	-2.094	-1.066
LOC644422	Unknown	A_24_P315444	0.00231	0.0212	-2.279	-1.189
NUP62CL	Homo sapiens nucleoporin 62kDa C-terminal like (NUP62CL), transcript variant 1, mRNA [NM_017681]	A_23_P33914	0.00232	0.0212	-2.033	-1.024
LOC286434	HCG1981372, isoform CRA_cNovel proteinUncharacterized protein [Source:UniProtKB/TrEMBL;Acc:B1B108] [ENST00000492160]	A_33_P3252322	0.00233	0.0213	-2.492	-1.318
ISX	Homo sapiens intestine-specific homeobox (ISX), mRNA [NM_001008494]	A_32_P217140	0.00233	0.0213	-8.463	-3.081
THC2524024	inner membrane protein, mitochondrial [Source:HGNC Symbol;Acc:6047] [ENST00000486633]	A_33_P3314466	0.00234	0.0213	-2.425	-1.278
ZNF530	Homo sapiens zinc finger protein 530 (ZNF530), mRNA [NM_020880]	A_33_P3299421	0.00234	0.0213	-2.217	-1.149
ACSBG1	Homo sapiens acyl-CoA synthetase bubblegum family member 1 (ACSBG1), transcript variant 1, mRNA [NM_015162]	A_23_P54488	0.00233	0.0213	-4.762	-2.251
SNAI3	Homo sapiens snail homolog 3 (Drosophila) (SNAI3), mRNA [NM_178310]	A_23_P170574	0.00236	0.0214	-2.593	-1.375
E2F1	Homo sapiens E2F transcription factor 1 (E2F1), mRNA [NM_005225]	A_23_P80032	0.00235	0.0214	-2.471	-1.305
LOC646160	Unknown	A_33_P3210857	0.00235	0.0214	-8.084	-3.015
LOC100130009	PREDICTED: Homo sapiens high mobility group protein HMG-I/HMG-Y-like (LOC100130009), mRNA [XM_001718914]	A_33_P3373985	0.00235	0.0214	-2.077	-1.055
AMOTL1	Homo sapiens angiomotin like 1 (AMOTL1), mRNA [NM_130847]	A_33_P3271196	0.00236	0.0214	-1.502	-0.587
LOC286109	Homo sapiens cDNA FLJ34853 fis, clone NT2NE2012199. [AK092172]	A_33_P3651911	0.00236	0.0214	-2.235	-1.16
NGEF	Homo sapiens neuronal guanine nucleotide exchange factor (NGEF), transcript variant 1, mRNA [NM_019850]	A_23_P102364	0.00236	0.0214	-3.189	-1.673
NUDT13	Homo sapiens nudix (nucleoside diphosphate linked moiety X)-type motif 13 (NUDT13), mRNA [NM_015901]	A_23_P161324	0.00237	0.0215	-2.145	-1.101
BLVRB	Homo sapiens biliverdin reductase B (flavin reductase (NADPH)) (BLVRB), mRNA [NM_000713]	A_33_P3360728	0.00238	0.0215	-2.912	-1.542
HIST1H4L	Homo sapiens histone cluster 1, H4l (HIST1H4L), mRNA [NM_003546]	A_33_P3351851	0.00237	0.0215	-4.052	-2.019
ENST000003934	Homo sapiens cDNA FLJ43991 fis, clone TESTI4019843, highly similar to Rattus norvegicus huntingtin-associated protein interacting protein (duo) (Hapip). [AK125979]	A_24_P410797	0.00239	0.0216	-5.312	-2.409
PKMYT1	Homo sapiens protein kinase, membrane associated tyrosine/threonine 1 (PKMYT1), transcript variant 2, mRNA [NM_182687]	A_33_P3397443	0.0024	0.0216	-2.374	-1.247
HIST1H2AC	Homo sapiens histone cluster 1, H2ac (HIST1H2AC), mRNA [NM_003512]	A_33_P3344127	0.0024	0.0216	-2.094	-1.066
TPST2	Homo sapiens tyrosylprotein sulfotransferase 2 (TPST2), transcript variant 1, mRNA [NM_001008566]	A_23_P132226	0.0024	0.0216	-2.226	-1.155
FLJ22374	Homo sapiens family with sequence similarity 188, member B (FAM188B), mRNA [NM_032222]	A_24_P126425	0.00242	0.0217	-4.388	-2.134
PNMT	Homo sapiens phenylethanolamine N-methyltransferase (PNMT), mRNA [NM_002686]	A_23_P100642	0.00241	0.0217	-6.646	-2.732
C16orf35	Homo sapiens nitrogen permease regulator-like 3 (S. cerevisiae) (NPRL3), transcript variant 2, mRNA [NM_001039476]	A_23_P100326	0.00242	0.0217	-3.927	-1.973

LCORL	Homo sapiens ligand dependent nuclear receptor corepressor-like (LCORL), transcript variant 1, mRNA [NM_001166139]	A_33_P3333890	0.00241	0.0217	-2.699	-1.432
TMEM97	Homo sapiens transmembrane protein 97 (TMEM97), mRNA [NM_014573]	A_24_P190168	0.00241	0.0217	-2.333	-1.222
LOC100652769	PREDICTED: Homo sapiens hypothetical protein LOC100652769 (LOC100652769), mRNA [XM_003403489]	A_21_P0014019	0.00243	0.0218	-2.761	-1.465
MAGI1	Homo sapiens membrane associated guanylate kinase, WW and PDZ domain containing 1 (MAGI1), transcript variant 2, mRNA [NM_004742]	A_24_P75220	0.00243	0.0218	-3.928	-1.974
TBX1	Homo sapiens T-box 1 (TBX1), transcript variant C, mRNA [NM_080647]	A_23_P211345	0.00245	0.0219	-2.815	-1.493
C20orf108	Homo sapiens chromosome 20 open reading frame 108 (C20orf108), mRNA [NM_080821]	A_23_P68486	0.00245	0.0219	-2.196	-1.135
TMEFF1	Homo sapiens C9orf30-TMEFF1 readthrough (C9orf30-TMEFF1), mRNA [NM_001198812]	A_33_P3219475	0.00245	0.0219	-3.209	-1.682
LOC284422	Homo sapiens chromosome 19 open reading frame 77 (C19orf77), mRNA [NM_001136503]	A_23_P397120	0.00246	0.022	-10.978	-3.457
BC021296	tripartite motif containing 16 [Source:HGNC Symbol;Acc:17241] [ENST00000494759]	A_33_P3416687	0.00249	0.0221	-2.059	-1.042
HSF4	Homo sapiens heat shock transcription factor 4 (HSF4), transcript variant 2, mRNA [NM_001040667]	A_23_P3592	0.00249	0.0221	-2.055	-1.039
ENST000004062	BCL6 corepressor [Source:HGNC Symbol;Acc:20893] [ENST00000501455]	A_23_P405707	0.00248	0.0221	-2.959	-1.565
UHRF1	Homo sapiens ubiquitin-like with PHD and ring finger domains 1 (UHRF1), transcript variant 2, mRNA [NM_013282]	A_33_P3379454	0.00248	0.0221	-3.917	-1.97
MSMP	Homo sapiens microseminoprotein, prostate associated (MSMP), mRNA [NM_001044264]	A_33_P3307113	0.00248	0.0221	-2.154	-1.107
JAK3	Homo sapiens Janus kinase 3 (JAK3), mRNA [NM_000215]	A_24_P59667	0.00251	0.0222	-5.388	-2.43
A_33_P3212316	Unknown	A_33_P3212316	0.00252	0.0222	-2.196	-1.135
LY6G6F	Homo sapiens lymphocyte antigen 6 complex, locus G6F (LY6G6F), mRNA [NM_001003693]	A_33_P3214334	0.00251	0.0222	-3.864	-1.95
LOC389217	PREDICTED: Homo sapiens protein SET-like (LOC389217), mRNA [XM_001717714]	A_33_P3238390	0.00251	0.0222	-2.402	-1.265
EXTL1	Homo sapiens exostoses (multiple)-like 1 (EXTL1), mRNA [NM_004455]	A_23_P62999	0.00254	0.0223	-4.504	-2.171
LHX6	Homo sapiens LIM homeobox 6 (LHX6), transcript variant 1, mRNA [NM_014368]	A_23_P32175	0.00253	0.0223	-2.852	-1.512
LOC645534	Unknown	A_33_P3378697	0.00253	0.0223	-2.163	-1.113
KIAA1539	Homo sapiens KIAA1539 (KIAA1539), mRNA [NM_025182]	A_24_P346762	0.00255	0.0224	-2.071	-1.05
GATA1	Homo sapiens GATA binding protein 1 (globin transcription factor 1) (GATA1), mRNA [NM_002049]	A_24_P374244	0.00256	0.0225	-4.182	-2.064
CDH15	Homo sapiens cadherin 15, type 1, M-cadherin (myotubule) (CDH15), mRNA [NM_004933]	A_32_P25357	0.00257	0.0225	-5.187	-2.375
PARD6A	Homo sapiens par-6 partitioning defective 6 homolog alpha (C. elegans) (PARD6A), transcript variant 1, mRNA [NM_016948]	A_23_P140821	0.0026	0.0226	-3.742	-1.904
LOC100130539	PREDICTED: Homo sapiens hypothetical protein LOC100130539 (LOC100130539), mRNA [XM_001724322]	A_33_P3258146	0.0026	0.0226	-2.057	-1.04
FAM188B	Homo sapiens family with sequence similarity 188, member B (FAM188B), mRNA [NM_032222]	A_33_P3326713	0.00258	0.0226	-2.97	-1.57
AP1B1	Homo sapiens adaptor-related protein complex 1, beta 1 subunit (AP1B1), transcript variant 1, mRNA [NM_001127]	A_33_P3317593	0.00258	0.0226	-2.061	-1.043

ZNF782	Homo sapiens zinc finger protein 782 (ZNF782), mRNA [NM_001001662]	A_33_P3326672	0.00258	0.0226	-2.163	-1.113
IDUA	Homo sapiens iduronidase, alpha-L- (IDUA), mRNA [NM_000203]	A_33_P3392087	0.0026	0.0226	-2.54	-1.345
PWWP2A	Homo sapiens PWWP domain containing 2A (PWWP2A), transcript variant 1, mRNA [NM_052927]	A_33_P3275943	0.0026	0.0226	-3.207	-1.681
PTPRN	Homo sapiens protein tyrosine phosphatase, receptor type, N (PTPRN), transcript variant 1, mRNA [NM_002846]	A_23_P90722	0.00259	0.0226	-2.119	-1.084
BAHCC1	Homo sapiens BAH domain and coiled-coil containing 1 (BAHCC1), mRNA [NM_001080519]	A_23_P49539	0.0026	0.0226	-2.277	-1.187
ARHGDIG	Homo sapiens Rho GDP dissociation inhibitor (GDI) gamma (ARHGDIG), mRNA [NM_001176]	A_24_P215882	0.00259	0.0226	-3.33	-1.735
KCNC4	Homo sapiens potassium voltage-gated channel, Shaw-related subfamily, member 4 (KCNC4), transcript variant 3, mRNA [NM_001039574]	A_23_P12079	0.0026	0.0226	-2.839	-1.505
EXO1	Homo sapiens exonuclease 1 (EXO1), transcript variant 3, mRNA [NM_003686]	A_23_P23303	0.00261	0.0227	-2.09	-1.064
ENST000003732	chromodomain helicase DNA binding protein 6 [Source:HGNC Symbol;Acc:19057] [ENST00000373222]	A_23_P102607	0.00263	0.0228	-2.369	-1.244
PHYHD1	Homo sapiens phytanoyl-CoA dioxygenase domain containing 1 (PHYHD1), transcript variant 2, mRNA [NM_174933]	A_23_P307844	0.00263	0.0228	-5.152	-2.365
TMEM91	Homo sapiens transmembrane protein 91 (TMEM91), transcript variant 5, mRNA [NM_001098824]	A_33_P3363620	0.00262	0.0228	-2.038	-1.027
JAG2	Homo sapiens jagged 2 (JAG2), transcript variant 1, mRNA [NM_002226]	A_23_P106024	0.00262	0.0228	-2.556	-1.354
ZNF467	Homo sapiens zinc finger protein 467 (ZNF467), mRNA [NM_207336]	A_24_P916718	0.00264	0.0229	-3.656	-1.87
A_19_P0031858	PREDICTED: Homo sapiens hypothetical protein LOC100652853 (LOC100652853), mRNA [XM_003403534]	A_19_P00318587	0.00264	0.0229	-2.404	-1.265
C6orf25	Homo sapiens chromosome 6 open reading frame 25 (C6orf25), transcript variant 7, mRNA [NM_138277]	A_32_P168349	0.00266	0.023	-15.425	-3.947
FAM135A	Homo sapiens family with sequence similarity 135, member A (FAM135A), transcript variant 2, mRNA [NM_020819]	A_23_P58862	0.00266	0.023	-2.291	-1.196
PIF1	Homo sapiens PIF1 5'-to-3' DNA helicase homolog (S. cerevisiae) (PIF1), mRNA [NM_025049]	A_23_P416468	0.00266	0.023	-2.475	-1.307
KTELC1	Homo sapiens protein O-glucosyltransferase 1 (POGLUT1), transcript variant 1, mRNA [NM_152305]	A_32_P26376	0.00267	0.023	-2.785	-1.478
ZNF420	Homo sapiens zinc finger protein 420 (ZNF420), mRNA [NM_144689]	A_33_P3395389	0.00268	0.023	-2.556	-1.354
STIL	Homo sapiens SCL/TAL1 interrupting locus (STIL), transcript variant 1, mRNA [NM_001048166]	A_33_P3400477	0.00267	0.023	-2.451	-1.293
PRIM1	Homo sapiens primase, DNA, polypeptide 1 (49kDa) (PRIM1), mRNA [NM_000946]	A_23_P25019	0.00267	0.023	-2.095	-1.067
BEGAIN	Homo sapiens brain-enriched guanylate kinase-associated homolog (rat) (BEGAIN), transcript variant 1, mRNA [NM_001159531]	A_33_P3321205	0.00269	0.0231	-4.087	-2.031
LOC100129094	PREDICTED: Homo sapiens butyrophilin related 1 (BUTR1), mRNA [XM_001722031]	A_24_P42039	0.0027	0.0232	-3.692	-1.884
ATPAF2	Homo sapiens ATP synthase mitochondrial F1 complex assembly factor 2 (ATPAF2), nuclear gene encoding mitochondrial protein, mRNA [NM_145691]	A_23_P418493	0.00272	0.0232	-2.19	-1.131
NUP210	Homo sapiens nucleoporin 210kDa (NUP210), mRNA [NM_024923]	A_23_P212159	0.00271	0.0232	-2.157	-1.109
ZNF397OS	Homo sapiens zinc finger and SCAN domain containing 30 (ZSCAN30), mRNA [NM_001166012]	A_33_P3398667	0.00272	0.0232	-1.456	-0.542

NAP1L4	nucleosome assembly protein 1-like 4 [Source:HGNC Symbol;Acc:7640] [ENST00000534372]	A_33_P3363938	0.00274	0.0233	-2.529	-1.339
H3F3A	Homo sapiens H3 histone, family 3A (H3F3A), mRNA [NM_002107]	A_23_P23141	0.00275	0.0234	-2.014	-1.01
ZNF124	Homo sapiens zinc finger protein 124 (ZNF124), transcript variant 1, mRNA [NM_003431]	A_23_P52176	0.00275	0.0234	-2.669	-1.416
SNPH	Homo sapiens syntaphilin (SNPH), mRNA [NM_014723]	A_23_P102706	0.00277	0.0235	-4.801	-2.263
C8orf55	Homo sapiens chromosome 8 open reading frame 55 (C8orf55), mRNA [NM_016647]	A_33_P3249748	0.00278	0.0235	-3.557	-1.831
GTPBP6	Homo sapiens GTP binding protein 6 (putative) (GTPBP6), mRNA [NM_012227]	A_23_P113523	0.00278	0.0235	-2.134	-1.093
CRIP2	Homo sapiens cysteine-rich protein 2 (CRIP2), mRNA [NM_001312]	A_23_P112798	0.00281	0.0236	-3.655	-1.87
AK057820	Novel proteinUncharacterized protein [Source:UniProtKB/TrEMBL;Acc:Q4V308] [ENST00000417488]	A_32_P4792	0.00281	0.0236	-2.638	-1.4
ENST000004370	transmembrane protein 178 [Source:HGNC Symbol;Acc:28517] [ENST00000437068]	A_33_P3249716	0.0028	0.0236	-2.217	-1.149
HOXB3	Homo sapiens homeobox B3 (HOXB3), mRNA [NM_002146]	A_23_P316511	0.00281	0.0236	-2.825	-1.499
RAB23	Homo sapiens RAB23, member RAS oncogene family (RAB23), transcript variant 1, mRNA [NM_016277]	A_23_P70794	0.0028	0.0236	-2.454	-1.295
SEPT8	Homo sapiens septin 8 (SEPT8), transcript variant 2, mRNA [NM_015146]	A_23_P415827	0.00283	0.0237	-2.467	-1.303
EXOC3L2	Homo sapiens exocyst complex component 3-like 2 (EXOC3L2), mRNA [NM_138568]	A_33_P3215392	0.00282	0.0237	-3.625	-1.858
CUL4B	Homo sapiens cullin 4B (CUL4B), transcript variant 1, mRNA [NM_003588]	A_23_P422178	0.00284	0.0238	-2.061	-1.043
WHSC1	Homo sapiens Wolf-Hirschhorn syndrome candidate 1 (WHSC1), transcript variant 7, mRNA [NM_133334]	A_23_P398530	0.00287	0.0239	-2.427	-1.279
ENST000003932	c-mer proto-oncogene tyrosine kinase [Source:HGNC Symbol;Acc:7027] [ENST00000393237]	A_33_P3402086	0.00286	0.0239	-4.136	-2.048
VWDE	Homo sapiens von Willebrand factor D and EGF domains (VWDE), mRNA [NM_001135924]	A_23_P381505	0.00288	0.024	-3.863	-1.95
AGAP1	Homo sapiens ArfGAP with GTPase domain, ankyrin repeat and PH domain 1 (AGAP1), transcript variant 1, mRNA [NM_001037131]	A_23_P108404	0.00289	0.0241	-2.195	-1.134
A_33_P3316088	Unknown	A_33_P3316088	0.0029	0.0241	-2.663	-1.413
CYP21A2	Homo sapiens cytochrome P450, family 21, subfamily A, polypeptide 2 (CYP21A2), transcript variant 1, mRNA [NM_000500]	A_23_P257478	0.00289	0.0241	-1.481	-0.567
SLC24A1	Homo sapiens solute carrier family 24 (sodium/potassium/calcium exchanger), member 1 (SLC24A1), mRNA [NM_004727]	A_33_P3417670	0.00291	0.0242	-3.317	-1.73
RAD21L1	Homo sapiens RAD21-like 1 (S. pombe) (RAD21L1), mRNA [NM_001136566]	A_33_P3419567	0.00294	0.0243	-3.489	-1.803
RBAK	Homo sapiens RB-associated KRAB zinc finger (RBAK), transcript variant 1, mRNA [NM_021163]	A_33_P3353622	0.00293	0.0243	-2.284	-1.192
CLCN3	Homo sapiens chloride channel 3 (CLCN3), transcript variant e, mRNA [NM_173872]	A_23_P124476	0.00294	0.0243	-1.885	-0.915
LOC728558	Q3RU97_RALME (Q3RU97) Sulfate transporter/antisigma-factor antagonist STAS:Sulphate transporter, partial (3%) [THC2519244]	A_33_P3232861	0.00295	0.0244	-2.605	-1.381
PI4KA	BC018120 PIK4CA protein {Homo sapiens} (exp=-1; wgp=0; cg=0), partial (15%) [THC2545921]	A_21_P0012342	0.00295	0.0244	-3.631	-1.86
NTHL1	Homo sapiens nth endonuclease III-like 1 (E. coli) (NTHL1), mRNA [NM_002528]	A_23_P88904	0.00295	0.0244	-2.21	-1.144

SYP	Homo sapiens synaptophysin (SYP), mRNA [NM_003179]	A_23_P136936	0.00295	0.0244	-2.707	-1.436
C15orf39	Homo sapiens chromosome 15 open reading frame 39 (C15orf39), mRNA [NM_015492]	A_23_P37514	0.00297	0.0245	-2.571	-1.362
SLC25A17	Homo sapiens solute carrier family 25 (mitochondrial carrier; peroxisomal membrane protein, 34kDa), member 17 (SLC25A17), nuclear gene encoding mitochondrial protein, mRNA [NM_006358]	A_23_P57547	0.00298	0.0245	-2.293	-1.197
A_19_P0031905(	Unknown	A_19_P00319050	0.00299	0.0246	-4.176	-2.062
ABHD1	Homo sapiens abhydrolase domain containing 1 (ABHD1), mRNA [NM_032604]	A_23_P17242	0.003	0.0246	-4.202	-2.071
BGLAP	Homo sapiens bone gamma-carboxyglutamate (gla) protein (BGLAP), mRNA [NM_199173]	A_24_P336551	0.00301	0.0246	-2.11	-1.077
DNAI2	Homo sapiens dynein, axonemal, intermediate chain 2 (DNAI2), transcript variant 1, mRNA [NM_023036]	A_33_P3285629	0.00299	0.0246	-8.373	-3.066
TYW3	Homo sapiens tRNA-yW synthesizing protein 3 homolog (S. cerevisiae) (TYW3), transcript variant 1, mRNA [NM_138467]	A_24_P396327	0.00301	0.0246	-2.119	-1.083
SEC11C	Homo sapiens SEC11 homolog C (S. cerevisiae) (SEC11C), mRNA [NM_033280]	A_23_P208009	0.00299	0.0246	-2.114	-1.08
KIAA1257	Homo sapiens KIAA1257 (KIAA1257), mRNA [NM_020741]	A_32_P163739	0.00303	0.0247	-2.883	-1.528
GCAT	Homo sapiens glycine C-acetyltransferase (GCAT), nuclear gene encoding mitochondrial protein, transcript variant 2, mRNA [NM_014291]	A_23_P40657	0.00302	0.0247	-3.886	-1.958
ESR2	Homo sapiens estrogen receptor 2 (ER beta) (ESR2), transcript variant a, mRNA [NM_001437]	A_23_P54100	0.00303	0.0247	-3.348	-1.743
GAB1	Homo sapiens GRB2-associated binding protein 1 (GAB1), transcript variant 1, mRNA [NM_207123]	A_23_P335239	0.00303	0.0247	-2.297	-1.2
FBXO2	Homo sapiens F-box protein 2 (FBXO2), mRNA [NM_012168]	A_23_P45999	0.00305	0.0248	-2.715	-1.441
UBAC1	Homo sapiens UBA domain containing 1 (UBAC1), mRNA [NM_016172]	A_23_P60324	0.00304	0.0248	-3.243	-1.697
GIN1	Homo sapiens gypsy retrotransposon integrase 1 (GIN1), mRNA [NM_017676]	A_33_P3382910	0.00305	0.0248	-2.018	-1.013
B4GALNT4	Homo sapiens beta-1,4-N-acetyl-galactosaminyl transferase 4 (B4GALNT4), mRNA [NM_178537]	A_33_P3317603	0.00305	0.0248	-5.597	-2.485
HIST1H1E	Homo sapiens histone cluster 1, H1e (HIST1H1E), mRNA [NM_005321]	A_23_P7976	0.00306	0.0249	-2.198	-1.136
A_33_P3231670	Homo sapiens ring finger protein 208 (RNF208), mRNA [NM_031297]	A_33_P3231670	0.00307	0.0249	-2.504	-1.324
CLSPN	Homo sapiens claspin (CLSPN), transcript variant 1, mRNA [NM_022111]	A_23_P126212	0.00308	0.025	-3.527	-1.818
PDCL2	Homo sapiens phosducin-like 2 (PDCL2), mRNA [NM_152401]	A_33_P3236517	0.00308	0.025	-2.255	-1.173
CCDC107	Homo sapiens coiled-coil domain containing 107 (CCDC107), transcript variant A, mRNA [NM_174923]	A_33_P3312877	0.00309	0.025	-2.168	-1.116
C1orf93	Homo sapiens chromosome 1 open reading frame 93 (C1orf93), transcript variant 1, mRNA [NM_001195736]	A_33_P3367332	0.00309	0.025	-5.472	-2.452
KANK2	Homo sapiens KN motif and ankyrin repeat domains 2 (KANK2), transcript variant 1, mRNA [NM_015493]	A_23_P50426	0.00308	0.025	-3.032	-1.6
ZBTB22	Homo sapiens zinc finger and BTB domain containing 22 (ZBTB22), transcript variant 1, mRNA [NM_005453]	A_23_P111188	0.00311	0.0251	-2.203	-1.139
PRR3	Homo sapiens proline rich 3 (PRR3), transcript variant 1, mRNA [NM_025263]	A_33_P3362616	0.0031	0.0251	-2.65	-1.406
PTK7	Homo sapiens PTK7 protein tyrosine kinase 7 (PTK7), transcript variant PTK7-1, mRNA [NM_002821]	A_24_P320545	0.00313	0.0252	-2.946	-1.559

LRRC29	Homo sapiens leucine rich repeat containing 29 (LRRC29), transcript variant 1, mRNA [NM_012163]	A_23_P49181	0.00313	0.0252	-2.447	-1.291
MCM2	Homo sapiens minichromosome maintenance complex component 2 (MCM2), mRNA [NM_004526]	A_32_P103633	0.00314	0.0252	-2.012	-1.009
CHCHD6	Homo sapiens coiled-coil-helix-coiled-coil-helix domain containing 6 (CHCHD6), mRNA [NM_032343]	A_33_P3235975	0.00313	0.0252	-2.029	-1.021
IDH3G	Homo sapiens isocitrate dehydrogenase 3 (NAD+) gamma (IDH3G), nuclear gene encoding mitochondrial protein, transcript variant 1, mRNA [NM_004135]	A_23_P34115	0.00316	0.0253	-2.071	-1.05
MAP4K2	Homo sapiens mitogen-activated protein kinase kinase kinase kinase 2 (MAP4K2), mRNA [NM_004579]	A_24_P287075	0.00315	0.0253	-2.246	-1.167
CHPF	chondroitin polymerizing factor [Source:HGNC Symbol;Acc:24291] [ENST00000373891]	A_33_P3262012	0.00316	0.0253	-2.74	-1.454
RBBP5	Homo sapiens retinoblastoma binding protein 5 (RBBP5), transcript variant 3, mRNA [NM_001193273]	A_33_P3235640	0.00316	0.0253	-2.146	-1.102
OSBP2	Homo sapiens oxysterol binding protein 2 (OSBP2), transcript variant 1, mRNA [NM_030758]	A_23_P321935	0.00315	0.0253	-5.941	-2.571
ENST000002932	Homo sapiens cDNA clone IMAGE:40078433. [BC128044]	A_24_P369898	0.00319	0.0254	-2.555	-1.353
RAI1	Homo sapiens retinoic acid induced 1 (RAI1), mRNA [NM_030665]	A_33_P3320503	0.00318	0.0254	-2.142	-1.099
SENP7	Homo sapiens SUMO1/sentrin specific peptidase 7 (SENP7), transcript variant 1, mRNA [NM_020654]	A_33_P3333507	0.00317	0.0254	-4.371	-2.128
SLC2A8	Homo sapiens solute carrier family 2 (facilitated glucose transporter), member 8 (SLC2A8), mRNA [NM_014580]	A_23_P252783	0.00324	0.0256	-2.629	-1.395
HMGB3	Homo sapiens high mobility group box 3 (HMGB3), mRNA [NM_005342]	A_33_P3319041	0.00326	0.0257	-2.633	-1.396
LOC123855	Unknown	A_33_P3326151	0.00325	0.0257	-5.107	-2.352
HIST1H4E	Homo sapiens histone cluster 1, H4e (HIST1H4E), mRNA [NM_003545]	A_23_P415411	0.00329	0.0258	-2.192	-1.132
FAIM2	Homo sapiens Fas apoptotic inhibitory molecule 2 (FAIM2), mRNA [NM_012306]	A_33_P3248982	0.00331	0.0259	-9.892	-3.306
MYLK	Homo sapiens myosin light chain kinase (MYLK), transcript variant 1, mRNA [NM_053025]	A_23_P143817	0.00333	0.026	-4.042	-2.015
THC2536940	Q8IWY7_HUMAN (Q8IWY7) Tau-tubulin kinase, partial (6%) [THC2536940]	A_33_P3303430	0.00333	0.026	-2.057	-1.04
EBI3	Homo sapiens Epstein-Barr virus induced 3 (EBI3), mRNA [NM_005755]	A_23_P119478	0.00333	0.026	-9.27	-3.213
NAB1	Homo sapiens NGFI-A binding protein 1 (EGR1 binding protein 1) (NAB1), mRNA [NM_005966]	A_24_P191417	0.00332	0.026	-1.697	-0.763
EPRS	Homo sapiens glutamyl-prolyl-tRNA synthetase (EPRS), mRNA [NM_004446]	A_23_P97632	0.00332	0.026	-2.173	-1.12
HIST1H1D	Homo sapiens histone cluster 1, H1d (HIST1H1D), mRNA [NM_005320]	A_24_P260639	0.00336	0.0261	-2.349	-1.232
MATK	Homo sapiens megakaryocyte-associated tyrosine kinase (MATK), transcript variant 1, mRNA [NM_139355]	A_23_P50678	0.00336	0.0261	-2.741	-1.455
KBTBD4	Homo sapiens kelch repeat and BTB (POZ) domain containing 4 (KBTBD4), transcript variant 2, mRNA [NM_016506]	A_23_P202696	0.00338	0.0262	-2.294	-1.198
PAN3	Homo sapiens PAN3 poly(A) specific ribonuclease subunit homolog (S. cerevisiae) (PAN3), mRNA [NM_175854]	A_33_P3259403	0.00339	0.0262	-2.146	-1.102
FADS2	Homo sapiens fatty acid desaturase 2 (FADS2), mRNA [NM_004265]	A_23_P98580	0.00338	0.0262	-4.22	-2.077
PELI3	Homo sapiens pellino homolog 3 (Drosophila) (PELI3), transcript variant 1, mRNA [NM_145065]	A_23_P104692	0.00341	0.0263	-2.269	-1.182

ATXN2L	Homo sapiens ataxin 2-like (ATXN2L), transcript variant E, mRNA [NM_148416]	A_23_P147439	0.00341	0.0263	-3.606	-1.85
AY358103	huntingtin interacting protein 1 [Source:HGNC Symbol;Acc:4913] [ENST00000479835]	A_33_P3343485	0.00341	0.0263	-2.804	-1.488
STAT5A	Homo sapiens signal transducer and activator of transcription 5A (STAT5A), mRNA [NM_003152]	A_23_P207367	0.0034	0.0263	-2.728	-1.448
PNCK	Homo sapiens pregnancy up-regulated non-ubiquitously expressed CaM kinase (PNCK), transcript variant 1, mRNA [NM_001039582]	A_23_P62377	0.00342	0.0264	-4.262	-2.092
COL29A1	Homo sapiens collagen, type VI, alpha 5 (COL6A5), mRNA [NM_153264]	A_23_P404698	0.00344	0.0264	-2.46	-1.298
CLTB	Homo sapiens clathrin, light chain B (CLTB), transcript variant 2, mRNA [NM_007097]	A_33_P3328666	0.00343	0.0264	-2.279	-1.189
TAS2R14	Homo sapiens taste receptor, type 2, member 14 (TAS2R14), mRNA [NM_023922]	A_23_P105495	0.00344	0.0264	-3.519	-1.815
AMHR2	Homo sapiens anti-Mullerian hormone receptor, type II (AMHR2), transcript variant 1, mRNA [NM_020547]	A_24_P115990	0.00349	0.0266	-5.827	-2.543
ATG4C	Homo sapiens ATG4 autophagy related 4 homolog C (S. cerevisiae) (ATG4C), transcript variant 1, mRNA [NM_032852]	A_33_P3396807	0.00348	0.0266	-3.309	-1.727
PDIK1L	Homo sapiens PDLIM1 interacting kinase 1 like (PDIK1L), transcript variant 3, mRNA [NM_001243533]	A_33_P3302280	0.00348	0.0266	-2.357	-1.237
TMEM41A	transmembrane protein 41A [Source:HGNC Symbol;Acc:30544] [ENST00000467061]	A_33_P3327567	0.00347	0.0266	-3.287	-1.717
WDHD1	Homo sapiens WD repeat and HMG-box DNA binding protein 1 (WDHD1), transcript variant 1, mRNA [NM_007086]	A_23_P25873	0.00347	0.0266	-2.04	-1.029
PDE11A	Homo sapiens phosphodiesterase 11A (PDE11A), transcript variant 4, mRNA [NM_016953]	A_32_P116857	0.00348	0.0266	-2.316	-1.212
IFITM5	Homo sapiens interferon induced transmembrane protein 5 (IFITM5), mRNA [NM_001025295]	A_33_P3352995	0.0035	0.0267	-3.87	-1.952
CHD1L	Homo sapiens chromodomain helicase DNA binding protein 1-like (CHD1L), mRNA [NM_004284]	A_33_P3315258	0.00351	0.0267	-2.237	-1.161
WNT10B	Homo sapiens wingless-type MMTV integration site family, member 10B (WNT10B), mRNA [NM_003394]	A_23_P162322	0.00353	0.0268	-2.361	-1.239
FHOD1	Homo sapiens formin homology 2 domain containing 1 (FHOD1), mRNA [NM_013241]	A_23_P37778	0.00353	0.0268	-2.584	-1.369
C12orf51	Homo sapiens chromosome 12 open reading frame 51 (C12orf51), mRNA [NM_001109662]	A_23_P162702	0.00356	0.0269	-1.638	-0.712
C18orf54	Homo sapiens chromosome 18 open reading frame 54 (C18orf54), mRNA [NM_173529]	A_32_P162374	0.00355	0.0269	-2.333	-1.222
GRASP	Homo sapiens GRP1 (general receptor for phosphoinositides 1)-associated scaffold protein (GRASP), mRNA [NM_181711]	A_33_P3299416	0.00356	0.0269	-2.672	-1.418
RAP1GAP	Homo sapiens RAP1 GTPase activating protein (RAP1GAP), transcript variant 3, mRNA [NM_002885]	A_23_P45976	0.00355	0.0269	-3.615	-1.854
LOC100130463	Homo sapiens cDNA FLJ42307 fis, clone TRACH2005066. [AK124300]	A_33_P3331711	0.00358	0.027	-2.009	-1.007
NAPRT1	Homo sapiens nicotinate phosphoribosyltransferase domain containing 1 (NAPRT1), mRNA [NM_145201]	A_23_P43238	0.0036	0.0271	-2.289	-1.195
COL6A2	Homo sapiens collagen, type VI, alpha 2 (COL6A2), transcript variant 2C2a', mRNA [NM_058175]	A_23_P310956	0.0036	0.0271	-2.47	-1.305

LRRC56	Homo sapiens leucine rich repeat containing 56 (LRRC56), mRNA [NM_198075]	A_23_P368996	0.00359	0.0271	-2.468	-1.303
DSCC1	Homo sapiens defective in sister chromatid cohesion 1 homolog (S. cerevisiae) (DSCC1), mRNA [NM_024094]	A_23_P252740	0.00361	0.0271	-2.182	-1.126
FOXN2	Homo sapiens forkhead box N2 (FOXN2), mRNA [NM_002158]	A_32_P140898	0.0036	0.0271	-3.21	-1.683
MAMSTR	Homo sapiens MEF2 activating motif and SAP domain containing transcriptional regulator (MAMSTR), transcript variant 2, mRNA [NM_182574]	A_33_P3329444	0.00361	0.0271	-2.196	-1.135
DOLPP1	Homo sapiens dolichyl pyrophosphate phosphatase 1 (DOLPP1), transcript variant 1, mRNA [NM_020438]	A_23_P386764	0.00364	0.0272	-2.349	-1.232
ALAS2	Homo sapiens aminolevulinate, delta-, synthase 2 (ALAS2), nuclear gene encoding mitochondrial protein, transcript variant 1, mRNA [NM_000032]	A_32_P385587	0.00364	0.0272	-12.18	-3.606
BRI3BP	Homo sapiens BRI3 binding protein (BRI3BP), mRNA [NM_080626]	A_32_P831181	0.00364	0.0272	-2.301	-1.203
ORC2L	Homo sapiens origin recognition complex, subunit 2 (ORC2), transcript variant 1, mRNA [NM_006190]	A_33_P3381410	0.00363	0.0272	-2.43	-1.281
NFATC2	Homo sapiens nuclear factor of activated T-cells, cytoplasmic, calcineurin-dependent 2 (NFATC2), transcript variant 1, mRNA [NM_012340]	A_33_P3215333	0.00362	0.0272	-2.546	-1.348
SLC5A10	Homo sapiens solute carrier family 5 (sodium/glucose cotransporter), member 10 (SLC5A10), transcript variant 1, mRNA [NM_152351]	A_23_P328022	0.00365	0.0273	-4.685	-2.228
PODXL2	Homo sapiens podocalyxin-like 2 (PODXL2), mRNA [NM_015720]	A_33_P3296366	0.00365	0.0273	-2.373	-1.247
ENST000003751	thiosulfate sulfurtransferase (rhodanese)-like domain containing 2 [Source:HGNC Symbol;Acc:30087] [ENST00000375163]	A_33_P3349576	0.00366	0.0273	-3.551	-1.828
TMEM206	Homo sapiens transmembrane protein 206 (TMEM206), transcript variant 2, mRNA [NM_018252]	A_23_P717	0.00368	0.0274	-2.203	-1.14
RHAG	Homo sapiens Rh-associated glycoprotein (RHAG), mRNA [NM_000324]	A_23_P256572	0.0037	0.0275	-6.119	-2.613
NTSR1	Homo sapiens neurotensin receptor 1 (high affinity) (NTSR1), mRNA [NM_002531]	A_23_P371039	0.00369	0.0275	-4.319	-2.111
C2orf7	Homo sapiens protease-associated domain containing 1 (PRADC1), mRNA [NM_032319]	A_23_P131596	0.00373	0.0276	-2.025	-1.018
MAP4K1	Homo sapiens mitogen-activated protein kinase kinase kinase kinase 1 (MAP4K1), transcript variant 1, mRNA [NM_001042600]	A_23_P5002	0.00373	0.0276	-2.133	-1.093
BAG1	Homo sapiens BCL2-associated athanogene (BAG1), transcript variant 1, mRNA [NM_001172415]	A_33_P3290919	0.00372	0.0276	-2.357	-1.237
A_33_P3375894	Unknown	A_33_P3375894	0.00372	0.0276	-2.811	-1.491
EFR3B	Homo sapiens EFR3 homolog B (S. cerevisiae) (EFR3B), mRNA [NM_014971]	A_32_P83049	0.00374	0.0277	-2.785	-1.478
A_33_P3409294	Unknown	A_33_P3409294	0.00375	0.0277	-2.882	-1.527
IDH2	Homo sapiens isocitrate dehydrogenase 2 (NADP+), mitochondrial (IDH2), nuclear gene encoding mitochondrial protein, mRNA [NM_002168]	A_23_P129209	0.00376	0.0278	-2.339	-1.226
NP106769	GB	A_33_P3224345	0.00376	0.0278	-3.344	-1.742
CD3D	Homo sapiens CD3d molecule, delta (CD3-TCR complex) (CD3D), transcript variant 1, mRNA [NM_000732]	A_33_P3375541	0.00377	0.0278	-3.463	-1.792
C19orf54	Homo sapiens chromosome 19 open reading frame 54 (C19orf54), mRNA [NM_198476]	A_33_P3401782	0.00378	0.0279	-2.177	-1.122
SOHLH1	Homo sapiens spermatogenesis and oogenesis specific basic helix-loop-helix 1 (SOHLH1), transcript variant 2, mRNA [NM_001012415]	A_24_P307964	0.0038	0.028	-4.522	-2.177

TRANK1	Homo sapiens tetratricopeptide repeat and ankyrin repeat containing 1 (TRANK1), mRNA [NM_014831]	A_21_P0012438	0.00382	0.0281	-2.351	-1.234
CCL25	Homo sapiens chemokine (C-C motif) ligand 25 (CCL25), transcript variant 1, mRNA [NM_005624]	A_23_P55828	0.00383	0.0281	-4.302	-2.105
RCN3	Homo sapiens reticulocalbin 3, EF-hand calcium binding domain (RCN3), mRNA [NM_020650]	A_23_P67339	0.00385	0.0282	-3.149	-1.655
DQX1	Homo sapiens DEAQ box RNA-dependent ATPase 1 (DQX1), mRNA [NM_133637]	A_23_P56659	0.00385	0.0282	-2.686	-1.425
RAB3B	Homo sapiens RAB3B, member RAS oncogene family (RAB3B), mRNA [NM_002867]	A_23_P388168	0.00385	0.0282	-6.443	-2.688
KCNC3	Homo sapiens potassium voltage-gated channel, Shaw-related subfamily, member 3 (KCNC3), mRNA [NM_004977]	A_23_P101516	0.00388	0.0283	-3.003	-1.587
DNAJC9	Homo sapiens DnaJ (Hsp40) homolog, subfamily C, member 9 (DNAJC9), mRNA [NM_015190]	A_23_P104372	0.00389	0.0284	-2.355	-1.236
PCK2	Homo sapiens phosphoenolpyruvate carboxykinase 2 (mitochondrial) (PCK2), nuclear gene encoding mitochondrial protein, transcript variant 2, mRNA [NM_001018073]	A_33_P3268532	0.0039	0.0284	-6.987	-2.805
A_33_P3228499	Unknown	A_33_P3228499	0.00393	0.0285	-2.83	-1.501
CARD10	Homo sapiens caspase recruitment domain family, member 10 (CARD10), mRNA [NM_014550]	A_23_P434890	0.00392	0.0285	-2.555	-1.353
CCNE1	Homo sapiens cyclin E1 (CCNE1), mRNA [NM_001238]	A_23_P209200	0.00393	0.0285	-2.697	-1.432
ZFAND2B	Homo sapiens zinc finger, AN1-type domain 2B (ZFAND2B), mRNA [NM_138802]	A_23_P131299	0.00394	0.0286	-2.108	-1.076
A_19_P0080267	Homo sapiens coiled-coil domain containing 7 (CCDC7), transcript variant 1, mRNA [NM_145023]	A_19_P00802679	0.00395	0.0286	-3.9	-1.963
C1QL4	Homo sapiens complement component 1, q subcomponent-like 4 (C1QL4), mRNA [NM_001008223]	A_33_P3400843	0.00394	0.0286	-3.352	-1.745
NPSA	Homo sapiens prostate-specific antigen mRNA, complete cds. [AF527974]	A_33_P3677814	0.00395	0.0286	-3.041	-1.605
A_33_P3345931	PREDICTED: Homo sapiens hypothetical protein LOC728503 (LOC728503), mRNA [XM_001127575]	A_33_P3345931	0.00395	0.0286	-2.212	-1.145
MBD1	Homo sapiens methyl-CpG binding domain protein 1 (MBD1), transcript variant 13, mRNA [NM_001204151]	A_33_P3398927	0.00395	0.0286	-1.348	-0.431
NNT	Homo sapiens nicotinamide nucleotide transhydrogenase (NNT), nuclear gene encoding mitochondrial protein, transcript variant 2, mRNA [NM_182977]	A_23_P70148	0.00397	0.0286	-2.51	-1.328
RAB40B	Homo sapiens RAB40B, member RAS oncogene family (RAB40B), mRNA [NM_006822]	A_23_P129801	0.00396	0.0286	-2.366	-1.243
CUZD1	Homo sapiens CUB and zona pellucida-like domains 1 (CUZD1), transcript variant 1, mRNA [NM_022034]	A_23_P47058	0.00398	0.0287	-2.551	-1.351
FAM46C	Homo sapiens family with sequence similarity 46, member C (FAM46C), mRNA [NM_017709]	A_33_P3314176	0.00399	0.0287	-5.461	-2.449
LATS1	Homo sapiens LATS, large tumor suppressor, homolog 1 (Drosophila) (LATS1), mRNA [NM_004690]	A_33_P3233764	0.00399	0.0287	-2.095	-1.067
ZCCHC14	Homo sapiens zinc finger, CCHC domain containing 14 (ZCCHC14), mRNA [NM_015144]	A_33_P3216319	0.00398	0.0287	-2.161	-1.112

UPF3A	Homo sapiens UPF3 regulator of nonsense transcripts homolog A (yeast) (UPF3A), transcript variant 1, mRNA [NM_023011]	A_21_P0011576	0.00398	0.0287	-1.639	-0.713
SLC38A10	Homo sapiens solute carrier family 38, member 10 (SLC38A10), transcript variant 2, mRNA [NM_138570]	A_23_P379630	0.00401	0.0288	-2.557	-1.355
C1orf135	Homo sapiens chromosome 1 open reading frame 135 (C1orf135), mRNA [NM_024037]	A_23_P160537	0.00401	0.0288	-2.094	-1.066
THC2707829	OPA1 antisense RNA 1 (non-protein coding) [Source:HGNC Symbol;Acc:40421] [ENST00000433105]	A_33_P3219860	0.00401	0.0288	-2.458	-1.298
PPFIA3	Homo sapiens protein tyrosine phosphatase, receptor type, f polypeptide (PTPRF), interacting protein (liprin), alpha 3 (PPFIA3), mRNA [NM_003660]	A_23_P320553	0.004	0.0288	-2.259	-1.176
MANEAL	Homo sapiens mannosidase, endo-alpha-like (MANEAL), transcript variant 1, mRNA [NM_001031740]	A_23_P391228	0.00408	0.0291	-2.526	-1.337
TEAD4	Homo sapiens TEA domain family member 4 (TEAD4), transcript variant 1, mRNA [NM_003213]	A_23_P94795	0.00407	0.0291	-2.859	-1.515
AF074986	Homo sapiens full length insert cDNA YH73H08. [AF074986]	A_32_P28223	0.00408	0.0291	-2.03	-1.021
WWP2	Homo sapiens WW domain containing E3 ubiquitin protein ligase 2 (WWP2), transcript variant 3, mRNA [NM_199423]	A_24_P342086	0.00411	0.0292	-2.058	-1.041
CCDC28B	Homo sapiens coiled-coil domain containing 28B (CCDC28B), mRNA [NM_024296]	A_23_P62764	0.00411	0.0292	-4.479	-2.163
FAM115C	Homo sapiens family with sequence similarity 115, member C (FAM115C), transcript variant 2, mRNA [NM_173678]	A_32_P214925	0.0041	0.0292	-3.109	-1.637
ENTPD8	Homo sapiens ectonucleoside triphosphate diphosphohydrolase 8 (ENTPD8), transcript variant 1, mRNA [NM_001033113]	A_33_P3420224	0.0041	0.0292	-2.44	-1.287
INPP5D	Homo sapiens inositol polyphosphate-5-phosphatase, 145kDa (INPP5D), transcript variant 1, mRNA [NM_001017915]	A_23_P61149	0.0041	0.0292	-2.223	-1.153
MCAM	Homo sapiens melanoma cell adhesion molecule (MCAM), mRNA [NM_006500]	A_23_P162171	0.00409	0.0292	-2.021	-1.015
STAB1	Homo sapiens stabilin 1 (STAB1), mRNA [NM_015136]	A_23_P32500	0.00414	0.0293	-3.653	-1.869
CLIP2	Homo sapiens CAP-GLY domain containing linker protein 2 (CLIP2), transcript variant 1, mRNA [NM_003388]	A_33_P3272169	0.00412	0.0293	-3.687	-1.882
CYP2F1	Homo sapiens cytochrome P450, family 2, subfamily F, polypeptide 1 (CYP2F1), mRNA [NM_000774]	A_23_P89981	0.00412	0.0293	-4.084	-2.03
GDF1	Homo sapiens growth differentiation factor 1 (GDF1), mRNA [NM_001492]	A_33_P3420816	0.00417	0.0294	-3.897	-1.963
ACSL6	Homo sapiens acyl-CoA synthetase long-chain family member 6 (ACSL6), transcript variant 2, mRNA [NM_001009185]	A_33_P3390656	0.00416	0.0294	-6.823	-2.77
SOS1	Homo sapiens son of sevenless homolog 1 (Drosophila) (SOS1), mRNA [NM_005633]	A_23_P343808	0.00415	0.0294	-1.849	-0.887
SYT5	Homo sapiens synaptotagmin V (SYT5), mRNA [NM_003180]	A_23_P78849	0.00417	0.0294	-2.408	-1.268
MRAP2	Homo sapiens melanocortin 2 receptor accessory protein 2 (MRAP2), mRNA [NM_138409]	A_23_P357207	0.0042	0.0295	-7.076	-2.823
APC	Homo sapiens adenomatous polyposis coli (APC), transcript variant 3, mRNA [NM_000038]	A_23_P70213	0.0042	0.0295	-1.721	-0.783
PHYHIP	Homo sapiens phytanoyl-CoA 2-hydroxylase interacting protein (PHYHIP), transcript variant 2, mRNA [NM_014759]	A_23_P433229	0.0042	0.0295	-2.604	-1.381
USP21	Homo sapiens ubiquitin specific peptidase 21 (USP21), transcript variant 3, mRNA [NM_001014443]	A_24_P139620	0.0042	0.0295	-2.478	-1.309

KLF6	Homo sapiens Kruppel-like factor 6 (KLF6), transcript variant B, mRNA [NM_001160124]	A_33_P3367615	0.00419	0.0295	-3.11	-1.637
COL18A1	Homo sapiens collagen, type XVIII, alpha 1 (COL18A1), transcript variant 1, mRNA [NM_030582]	A_23_P211212	0.00423	0.0296	-6.412	-2.681
ZNF25	Homo sapiens zinc finger protein 25 (ZNF25), mRNA [NM_145011]	A_24_P69691	0.00422	0.0296	-2.279	-1.188
THC2680929	ALU1_HUMAN (P39188) Alu subfamily J sequence contamination warning entry, partial (5%) [THC2680929]	A_33_P3397910	0.00423	0.0296	-2.98	-1.575
CACNA1G	Homo sapiens calcium channel, voltage-dependent, T type, alpha 1G subunit (CACNA1G), transcript variant 1, mRNA [NM_018896]	A_23_P107247	0.00421	0.0296	-2.629	-1.395
PPIL4	Homo sapiens peptidylprolyl isomerase (cyclophilin)-like 4 (PPIL4), mRNA [NM_139126]	A_23_P42507	0.00427	0.0297	-2.754	-1.461
ERMAP	Homo sapiens erythroblast membrane-associated protein (Scianna blood group) (ERMAP), transcript variant 1, mRNA [NM_001017922]	A_24_P105391	0.00426	0.0297	-5.328	-2.413
THUMPD2	THUMP domain containing 2 [Source:HGNC Symbol;Acc:14890] [ENST00000403537]	A_33_P3424384	0.00426	0.0297	-2.288	-1.194
RANBP2	Homo sapiens RAN binding protein 2 (RANBP2), mRNA [NM_006267]	A_33_P3308740	0.00426	0.0297	-2.112	-1.078
RAC3	Homo sapiens ras-related C3 botulinum toxin substrate 3 (rho family, small GTP binding protein Rac3) (RAC3), mRNA [NM_005052]	A_33_P3295523	0.00426	0.0297	-2.636	-1.398
PAX8	Homo sapiens paired box 8 (PAX8), transcript variant PAX8A, mRNA [NM_003466]	A_33_P3234809	0.00424	0.0297	-2.073	-1.052
ZWINT	Homo sapiens ZW10 interactor (ZWINT), transcript variant 2, mRNA [NM_032997]	A_33_P3212994	0.00426	0.0297	-2.19	-1.131
TLE2	Homo sapiens transducin-like enhancer of split 2 (E(sp1) homolog, Drosophila) (TLE2), transcript variant 1, mRNA [NM_003260]	A_23_P153676	0.00424	0.0297	-2.188	-1.13
DRD4	Homo sapiens dopamine receptor D4 (DRD4), mRNA [NM_000797]	A_33_P3373765	0.00428	0.0298	-2.437	-1.285
FEZ1	Homo sapiens fasciculation and elongation protein zeta 1 (zygin I) (FEZ1), transcript variant 1, mRNA [NM_005103]	A_23_P202881	0.00432	0.0299	-2.344	-1.229
CANT1	Homo sapiens calcium activated nucleotidase 1 (CANT1), transcript variant 1, mRNA [NM_138793]	A_24_P116669	0.00436	0.03	-2.107	-1.075
SPINT2	Homo sapiens serine peptidase inhibitor, Kunitz type, 2 (SPINT2), transcript variant a, mRNA [NM_021102]	A_23_P27795	0.00436	0.03	-3.532	-1.821
A_33_P3266889	Unknown	A_33_P3266889	0.00435	0.03	-2.045	-1.032
ANKRD20A3	Homo sapiens ankyrin repeat domain 20 family, member A3 (ANKRD20A3), mRNA [NM_001012419]	A_21_P0010647	0.00436	0.03	-3.51	-1.812
TYMS	Homo sapiens thymidylate synthetase (TYMS), mRNA [NM_001071]	A_23_P50096	0.00435	0.03	-2.515	-1.331
TMC6	Homo sapiens transmembrane channel-like 6 (TMC6), transcript variant 2, mRNA [NM_007267]	A_23_P101013	0.00436	0.03	-3.893	-1.961
DLX1	Homo sapiens distal-less homeobox 1 (DLX1), transcript variant 1, mRNA [NM_178120]	A_32_P142818	0.00438	0.0301	-3.629	-1.86
ZNF780B	Homo sapiens zinc finger protein 780B (ZNF780B), mRNA [NM_001005851]	A_33_P3312638	0.00437	0.0301	-2.77	-1.47
ENST000004783	Homo sapiens mRNA; cDNA DKFZp686O24114 (from clone DKFZp686O24114). [BX640643]	A_33_P3333554	0.00437	0.0301	-2.946	-1.559
TM7SF2	Homo sapiens transmembrane 7 superfamily member 2 (TM7SF2), mRNA [NM_003273]	A_23_P116037	0.00438	0.0301	-2.21	-1.144
ZNF791	Homo sapiens zinc finger protein 791 (ZNF791), mRNA [NM_153358]	A_24_P382401	0.00444	0.0303	-2.171	-1.118
TMEM52	Homo sapiens transmembrane protein 52 (TMEM52), mRNA [NM_178545]	A_33_P3251073	0.00442	0.0303	-2.203	-1.139

ATXN3	Homo sapiens ataxin 3 (ATXN3), transcript variant reference, mRNA [NM_004993]	A_33_P3320762	0.00444	0.0303	-1.547	-0.63
PITX1	Homo sapiens paired-like homeodomain 1 (PITX1), mRNA [NM_002653]	A_33_P3240328	0.00442	0.0303	-2	-1
A_33_P3284584	Unknown	A_33_P3284584	0.00445	0.0304	-3.62	-1.856
EPOR	Homo sapiens erythropoietin receptor (EPOR), transcript variant 1, mRNA [NM_000121]	A_23_P367899	0.00447	0.0305	-3.22	-1.687
OSBPL5	Homo sapiens oxysterol binding protein-like 5 (OSBPL5), transcript variant 1, mRNA [NM_020896]	A_23_P53081	0.00449	0.0305	-2.343	-1.229
MOBKL2B	Homo sapiens MOB kinase activator 3B (MOB3B), mRNA [NM_024761]	A_24_P32085	0.00451	0.0306	-5.063	-2.34
RNF160	Homo sapiens listerin E3 ubiquitin protein ligase 1 (LTN1), mRNA [NM_015565]	A_32_P115505	0.00451	0.0306	-2.908	-1.54
RTN4RL1	Homo sapiens reticulon 4 receptor-like 1 (RTN4RL1), mRNA [NM_178568]	A_23_P66481	0.0045	0.0306	-7.334	-2.875
MAN1C1	Homo sapiens mannosidase, alpha, class 1C, member 1 (MAN1C1), mRNA [NM_020379]	A_23_P103601	0.00453	0.0307	-2.445	-1.29
DMBX1	Homo sapiens diencephalon/mesencephalon homeobox 1 (DMBX1), transcript variant 2, mRNA [NM_147192]	A_23_P500542	0.00456	0.0308	-2.997	-1.584
SYDE2	Homo sapiens synapse defective 1, Rho GTPase, homolog 2 (C. elegans) (SYDE2), mRNA [NM_032184]	A_33_P3412767	0.00456	0.0308	-2.541	-1.345
GDF3	Homo sapiens growth differentiation factor 3 (GDF3), mRNA [NM_020634]	A_23_P72817	0.00458	0.0309	-3.753	-1.908
SMC6	Homo sapiens structural maintenance of chromosomes 6 (SMC6), transcript variant 2, mRNA [NM_024624]	A_23_P209962	0.00465	0.0312	-2.041	-1.03
POLR1A	Homo sapiens polymerase (RNA) I polypeptide A, 194kDa (POLR1A), mRNA [NM_015425]	A_33_P3610123	0.00464	0.0312	-2.113	-1.079
KIAA0317	human full-length cDNA 5-PRIME end of clone CS0CAP004YO05 of Thymus of Homo sapiens (human). [BX248747]	A_33_P3424143	0.00464	0.0312	-2.06	-1.042
WDR62	Homo sapiens WD repeat domain 62 (WDR62), transcript variant 1, mRNA [NM_001083961]	A_33_P3254606	0.00464	0.0312	-3.297	-1.721
CDCA3	Homo sapiens cell division cycle associated 3 (CDCA3), mRNA [NM_031299]	A_24_P218979	0.00465	0.0312	-2.36	-1.239
PTGIR	Homo sapiens prostaglandin I2 (prostacyclin) receptor (IP) (PTGIR), mRNA [NM_000960]	A_23_P340848	0.00469	0.0314	-8.991	-3.169
GINS2	Homo sapiens GINS complex subunit 2 (Psf2 homolog) (GINS2), mRNA [NM_016095]	A_23_P118246	0.00473	0.0315	-2.333	-1.222
SPIN2B	Homo sapiens spindlin family, member 2B (SPIN2B), transcript variant 3, mRNA [NM_001006683]	A_24_P194508	0.00478	0.0316	-2.084	-1.059
HOXC12	Homo sapiens homeobox C12 (HOXC12), mRNA [NM_173860]	A_23_P335981	0.00477	0.0316	-3.124	-1.643
PIGM	Homo sapiens phosphatidylinositol glycan anchor biosynthesis, class M (PIGM), mRNA [NM_145167]	A_33_P3386965	0.00476	0.0316	-2.455	-1.296
FSD1	Homo sapiens fibronectin type III and SPRY domain containing 1 (FSD1), mRNA [NM_024333]	A_33_P3414789	0.00477	0.0316	-2.2	-1.138
AK056253	Homo sapiens cDNA FLJ44722 fis, clone BRACE3022847. [AK126677]	A_33_P3343828	0.00478	0.0316	-2.187	-1.129
NME4	Homo sapiens non-metastatic cells 4, protein expressed in (NME4), nuclear gene encoding mitochondrial protein, mRNA [NM_005009]	A_24_P210829	0.00477	0.0316	-2.62	-1.39
TUBAL3	Homo sapiens tubulin, alpha-like 3 (TUBAL3), transcript variant 1, mRNA [NM_024803]	A_33_P3332252	0.00481	0.0317	-2.233	-1.159
PIKFYVE	Homo sapiens phosphoinositide kinase, FYVE finger containing (PIKFYVE), transcript variant 4, mRNA [NM_001178000]	A_33_P3348973	0.00482	0.0317	-2.137	-1.095

GPR174	Homo sapiens G protein-coupled receptor 174 (GPR174), mRNA [NM_032553]	A_23_P11070	0.00479	0.0317	-2.199	-1.137
CD320	Homo sapiens CD320 molecule (CD320), transcript variant 1, mRNA [NM_016579]	A_23_P119698	0.00479	0.0317	-2.536	-1.343
B3GNT3	Homo sapiens UDP-GlcNAc:betaGal beta-1,3-N-acetylglucosaminyltransferase 3 (B3GNT3), mRNA [NM_014256]	A_23_P78980	0.00485	0.0318	-7.244	-2.857
GAB3	Homo sapiens GRB2-associated binding protein 3 (GAB3), transcript variant 1, mRNA [NM_001081573]	A_33_P3251369	0.00485	0.0318	-2.446	-1.291
ENST000003686	chromatin target of PRMT1 [Source:HGNC Symbol;Acc:24511] [ENST00000368686]	A_33_P3243957	0.00484	0.0318	-2.433	-1.283
HESX1	Homo sapiens HESX homeobox 1 (HESX1), mRNA [NM_003865]	A_23_P121106	0.00487	0.0319	-2.627	-1.393
STON2	Homo sapiens stonin 2 (STON2), mRNA [NM_033104]	A_23_P309837	0.00487	0.0319	-2.579	-1.367
CLCC1	Homo sapiens chloride channel CLIC-like 1 (CLCC1), transcript variant 1, mRNA [NM_001048210]	A_33_P3224250	0.0049	0.032	-2.266	-1.18
AFMID	Homo sapiens arylformamidase (AFMID), transcript variant 2, mRNA [NM_001010982]	A_24_P33156	0.00488	0.032	-2.838	-1.505
WDR20	Homo sapiens WD repeat domain 20 (WDR20), transcript variant 7, mRNA [NM_001242417]	A_23_P205255	0.00491	0.0321	-2.176	-1.122
ENST000003292	WASH and IL9R antisense RNA 1 (non-protein coding) [Source:HGNC Symbol;Acc:38513] [ENST00000399966]	A_33_P3277890	0.00493	0.0321	-2.668	-1.416
ONECUT3	Homo sapiens one cut homeobox 3 (ONECUT3), mRNA [NM_001080488]	A_33_P3238969	0.00492	0.0321	-3.179	-1.669
C6orf126	Homo sapiens chromosome 6 open reading frame 126 (C6orf126), mRNA [NM_207409]	A_33_P3350493	0.00492	0.0321	-2.293	-1.197
CYP2S1	Homo sapiens cytochrome P450, family 2, subfamily S, polypeptide 1 (CYP2S1), mRNA [NM_030622]	A_23_P101374	0.00493	0.0321	-2.476	-1.308
PCDHGB4	Homo sapiens protocadherin gamma subfamily B, 4 (PCDHGB4), transcript variant 2, mRNA [NM_032098]	A_23_P359588	0.00496	0.0323	-2.597	-1.377
FXYD7	Homo sapiens FXYD domain containing ion transport regulator 7 (FXYD7), mRNA [NM_022006]	A_23_P119611	0.00498	0.0323	-3.445	-1.784
PCSK6	Homo sapiens proprotein convertase subtilisin/kexin type 6 (PCSK6), transcript variant 3, mRNA [NM_138322]	A_24_P189997	0.00497	0.0323	-2.43	-1.281
APOA1	Homo sapiens apolipoprotein A-I (APOA1), mRNA [NM_000039]	A_33_P3298539	0.00496	0.0323	-2.307	-1.206
NFE2	Homo sapiens nuclear factor (erythroid-derived 2), 45kDa (NFE2), transcript variant 1, mRNA [NM_006163]	A_23_P13753	0.005	0.0324	-2.508	-1.327
KRT19	Homo sapiens keratin 19 (KRT19), mRNA [NM_002276]	A_23_P66798	0.00501	0.0324	-3.165	-1.662
PRO0628	Homo sapiens, clone IMAGE:4399587, mRNA. [BC015320]	A_33_P3324137	0.00504	0.0325	-2.153	-1.106
GPATCH1	Homo sapiens G patch domain containing 1 (GPATCH1), mRNA [NM_018025]	A_23_P27822	0.00505	0.0326	-2.095	-1.067
CDCP1	Homo sapiens CUB domain containing protein 1 (CDCP1), transcript variant 1, mRNA [NM_022842]	A_23_P113613	0.00507	0.0327	-2.655	-1.409
ENST000004036	Homo sapiens PAS domain containing serine/threonine kinase (PASK), transcript variant 5, mRNA [NM_001252124]	A_23_P408239	0.0051	0.0328	-2.086	-1.061
OBSCN	obscurin, cytoskeletal calmodulin and titin-interacting RhoGEF [Source:HGNC Symbol;Acc:15719] [ENST00000366706]	A_33_P3239367	0.00512	0.0328	-2.293	-1.197
MYBL2	Homo sapiens v-myb myeloblastosis viral oncogene homolog (avian)-like 2 (MYBL2), mRNA [NM_002466]	A_23_P143190	0.00513	0.0329	-2.715	-1.441

BMP1	Homo sapiens bone morphogenetic protein 1 (BMP1), transcript variant 1, mRNA [NM_001199]	A_24_P129417	0.00513	0.0329	-2.409	-1.269
PAGE1	Homo sapiens P antigen family, member 1 (prostate associated) (PAGE1), mRNA [NM_003785]	A_24_P314337	0.00513	0.0329	-2.701	-1.433
DCHS1	Homo sapiens dachsous 1 (Drosophila) (DCHS1), mRNA [NM_003737]	A_23_P98645	0.00514	0.0329	-4.739	-2.245
SFXN3	Homo sapiens sideroflexin 3 (SFXN3), mRNA [NM_030971]	A_23_P202138	0.00516	0.033	-3.037	-1.603
NAT6	Homo sapiens N-acetyltransferase 6 (GCN5-related) (NAT6), transcript variant 1, mRNA [NM_012191]	A_23_P212400	0.00516	0.033	-2.074	-1.052
SEPT9	Homo sapiens septin 9 (SEPT9), transcript variant 3, mRNA [NM_006640]	A_33_P3368188	0.00516	0.033	-3.717	-1.894
BCL9L	Homo sapiens B-cell CLL/lymphoma 9-like (BCL9L), mRNA [NM_182557]	A_33_P3405957	0.0052	0.0331	-2.193	-1.133
FKBP9	Homo sapiens FK506 binding protein 9, 63 kDa (FKBP9), mRNA [NM_007270]	A_21_P0013306	0.00523	0.0332	-2.567	-1.36
THC2661723	T-cell lymphoma invasion and metastasis 2 [Source:HGNC Symbol;Acc:11806] [ENST00000535064]	A_33_P3282374	0.0053	0.0335	-3.788	-1.922
CCDC48	Homo sapiens coiled-coil domain containing 48 (CCDC48), mRNA [NM_024768]	A_23_P166566	0.00533	0.0336	-5.696	-2.51
ZNF544	Homo sapiens zinc finger protein 544 (ZNF544), mRNA [NM_014480]	A_23_P141863	0.00531	0.0336	-2.013	-1.009
UPF2	Homo sapiens UPF2 regulator of nonsense transcripts homolog (yeast) (UPF2), transcript variant 1, mRNA [NM_080599]	A_33_P3279049	0.00531	0.0336	-2.471	-1.305
ALX1	Homo sapiens ALX homeobox 1 (ALX1), mRNA [NM_006982]	A_23_P83798	0.00535	0.0337	-3.335	-1.738
ZNF211	Homo sapiens zinc finger protein 211 (ZNF211), transcript variant 2, mRNA [NM_198855]	A_23_P130482	0.00534	0.0337	-2.04	-1.029
PRKCQ	Homo sapiens protein kinase C, theta (PRKCQ), transcript variant 1, mRNA [NM_006257]	A_23_P1374	0.00536	0.0337	-2.189	-1.13
LOC147645	Homo sapiens V-set and immunoglobulin domain containing 10 like (VSIG10L), mRNA [NM_001163922]	A_23_P101246	0.00538	0.0338	-2.634	-1.397
LOC644613	Homo sapiens hypothetical protein LOC644613, mRNA (cDNA clone IMAGE:4177475), partial cds. [BC025792]	A_33_P3699445	0.0054	0.0338	-2.278	-1.188
GFI1B	Homo sapiens growth factor independent 1B transcription repressor (GFI1B), transcript variant 1, mRNA [NM_004188]	A_23_P216845	0.00539	0.0338	-7.661	-2.938
KIAA1545	Homo sapiens fibrosin-like 1 (FBRSL1), mRNA [NM_001142641]	A_23_P368934	0.00541	0.0339	-2.909	-1.541
CR605783	Homo sapiens cDNA FLJ36124 fis, clone TESTI2022952. [AK093443]	A_33_P3397314	0.00542	0.0339	-2.111	-1.078
STON1	Homo sapiens stonin 1 (STON1), transcript variant 2, mRNA [NM_006873]	A_23_P302005	0.00546	0.034	-2.827	-1.499
LPCAT1	Homo sapiens lysophosphatidylcholine acyltransferase 1 (LPCAT1), mRNA [NM_024830]	A_24_P406006	0.00545	0.034	-2.005	-1.004
LOC100134147	Unknown	A_33_P3282649	0.00544	0.034	-2.487	-1.314
CPXM1	Homo sapiens carboxypeptidase X (M14 family), member 1 (CPXM1), transcript variant 1, mRNA [NM_019609]	A_21_P0000096	0.00545	0.034	-14.268	-3.835
ZNF573	Homo sapiens zinc finger protein 573 (ZNF573), transcript variant 1, mRNA [NM_152360]	A_23_P339079	0.00544	0.034	-2.256	-1.174
ZNF3	Homo sapiens zinc finger protein 3 (ZNF3), transcript variant 1, mRNA [NM_017715]	A_33_P3239532	0.00547	0.0341	-2.299	-1.201
STX2	Homo sapiens syntaxin 2 (STX2), transcript variant 1, mRNA [NM_001980]	A_24_P364296	0.0055	0.0342	-2.533	-1.341
DGCR8	Homo sapiens DiGeorge syndrome critical region gene 8 (DGCR8), transcript variant 1, mRNA [NM_022720]	A_23_P211355	0.00551	0.0342	-2.177	-1.122

CHST6	Homo sapiens carbohydrate (N-acetylglucosamine 6-O) sulfotransferase 6 (CHST6), mRNA [NM_021615]	A_23_P106922	0.00554	0.0343	-3.863	-1.95
FAM49A	Homo sapiens family with sequence similarity 49, member A (FAM49A), mRNA [NM_030797]	A_23_P21560	0.00553	0.0343	-2.064	-1.045
CCDC151	Homo sapiens coiled-coil domain containing 151 (CCDC151), mRNA [NM_145045]	A_23_P326893	0.00558	0.0344	-3.362	-1.749
SLAMF6	Homo sapiens SLAM family member 6 (SLAMF6), transcript variant 1, mRNA [NM_001184714]	A_33_P3303857	0.00557	0.0344	-4.373	-2.129
SARS	Homo sapiens seryl-tRNA synthetase (SARS), transcript variant 2, non-coding RNA [NR_034072]	A_21_P0000629	0.00561	0.0345	-2.719	-1.443
INTS2	Homo sapiens integrator complex subunit 2 (INTS2), transcript variant 1, mRNA [NM_020748]	A_23_P420269	0.0056	0.0345	-2.062	-1.044
LGI3	Homo sapiens leucine-rich repeat LGI family, member 3 (LGI3), mRNA [NM_139278]	A_23_P21086	0.00559	0.0345	-2.194	-1.134
A_33_P3250323	Unknown	A_33_P3250323	0.00561	0.0345	-2.871	-1.522
ABCA2	Homo sapiens ATP-binding cassette, sub-family A (ABC1), member 2 (ABCA2), transcript variant 1, mRNA [NM_001606]	A_23_P43504	0.00562	0.0346	-2.239	-1.163
ENST000003794	Homo sapiens cDNA clone IMAGE:5538783. [BC040611]	A_33_P3397579	0.00562	0.0346	-2.734	-1.451
A_33_P3384628	Unknown	A_33_P3384628	0.00562	0.0346	-2.672	-1.418
CES3	Homo sapiens carboxylesterase 3 (CES3), transcript variant 1, mRNA [NM_024922]	A_24_P90881	0.00566	0.0348	-2.269	-1.182
ELK3	Homo sapiens ELK3, ETS-domain protein (SRF accessory protein 2) (ELK3), mRNA [NM_005230]	A_23_P338325	0.00568	0.0348	-2.01	-1.008
LOC100509263	PREDICTED: Homo sapiens hypothetical protein LOC100509263 (LOC100509263), mRNA [XM_003403443]	A_21_P0014006	0.00571	0.0349	-2.789	-1.48
ANAPC1	Homo sapiens anaphase promoting complex subunit 1 (ANAPC1), mRNA [NM_022662]	A_24_P186346	0.00574	0.035	-2.036	-1.026
MGA	Homo sapiens MAX gene associated (MGA), transcript variant 2, mRNA [NM_001080541]	A_33_P3330323	0.00572	0.035	-2.298	-1.201
CD52	Homo sapiens CD52 molecule (CD52), mRNA [NM_001803]	A_23_P85800	0.00574	0.035	-4.902	-2.293
TSEN15	Homo sapiens tRNA splicing endonuclease 15 homolog (S. cerevisiae) (TSEN15), transcript variant 3, non-coding RNA [NR_023349]	A_33_P3214436	0.00576	0.0351	-3.164	-1.662
CDC42EP4	Homo sapiens CDC42 effector protein (Rho GTPase binding) 4 (CDC42EP4), mRNA [NM_012121]	A_23_P66891	0.00577	0.0351	-2.001	-1.001
C10orf95	Homo sapiens chromosome 10 open reading frame 95 (C10orf95), mRNA [NM_024886]	A_23_P138480	0.00579	0.0352	-3.13	-1.646
ENST000004356	Unknown	A_33_P3304824	0.00579	0.0352	-2.035	-1.025
HINT2	Homo sapiens histidine triad nucleotide binding protein 2 (HINT2), nuclear gene encoding mitochondrial protein, mRNA [NM_032593]	A_23_P157784	0.00581	0.0353	-2.449	-1.292
FSCN1	Homo sapiens fascin homolog 1, actin-bundling protein (Strongylocentrotus purpuratus) (FSCN1), mRNA [NM_003088]	A_33_P3411075	0.00581	0.0353	-2.475	-1.307
NFATC1	Homo sapiens nuclear factor of activated T-cells, cytoplasmic, calcineurin-dependent 1 (NFATC1), transcript variant 3, mRNA [NM_172387]	A_23_P300150	0.00584	0.0354	-2.216	-1.148
PCYT2	Homo sapiens phosphate cytidylyltransferase 2, ethanolamine (PCYT2), transcript variant 1, mRNA [NM_001184917]	A_33_P3394809	0.00585	0.0354	-3.39	-1.761

HYAL2	Homo sapiens hyaluronoglucosaminidase 2 (HYAL2), transcript variant 2, mRNA [NM_033158]	A_33_P3365357	0.00584	0.0354	-2.262	-1.177
EIF4EBP1	Homo sapiens eukaryotic translation initiation factor 4E binding protein 1 (EIF4EBP1), mRNA [NM_004095]	A_23_P22224	0.00588	0.0355	-2.342	-1.228
GHRHR	Homo sapiens growth hormone releasing hormone receptor (GHRHR), mRNA [NM_000823]	A_33_P3232655	0.00586	0.0355	-3.708	-1.891
STMN3	Homo sapiens stathmin-like 3 (STMN3), mRNA [NM_015894]	A_24_P678104	0.0059	0.0356	-4.228	-2.08
C17orf101	Homo sapiens chromosome 17 open reading frame 101 (C17orf101), transcript variant 1, mRNA [NM_024648]	A_24_P364954	0.00592	0.0357	-2.47	-1.304
ONECUT2	Homo sapiens one cut homeobox 2 (ONECUT2), mRNA [NM_004852]	A_32_P124708	0.00593	0.0357	-4.222	-2.078
PIK3R3	Homo sapiens phosphoinositide-3-kinase, regulatory subunit 3 (gamma) (PIK3R3), transcript variant 1, mRNA [NM_003629]	A_33_P3593774	0.00592	0.0357	-7.774	-2.959
ZNF385C	Homo sapiens zinc finger protein 385C (ZNF385C), mRNA [NM_001242704]	A_33_P3340580	0.00592	0.0357	-2.596	-1.376
LOC401037	Homo sapiens cDNA FLJ31684 fis, clone NT2RI2005358. [AK056246]	A_33_P3662000	0.00595	0.0358	-3.312	-1.728
PPME1	Homo sapiens protein phosphatase methylesterase 1 (PPME1), mRNA [NM_016147]	A_24_P4705	0.00596	0.0358	-2.639	-1.4
TSPAN32	Homo sapiens tetraspanin 32 (TSPAN32), mRNA [NM_139022]	A_23_P501722	0.00596	0.0358	-9.755	-3.286
NBL1	Homo sapiens C1orf151-NBL1 readthrough (C1orf151-NBL1), transcript variant 1, mRNA [NM_001204088]	A_33_P3236881	0.00599	0.0359	-2.105	-1.074
RBP5	Homo sapiens retinol binding protein 5, cellular (RBP5), mRNA [NM_031491]	A_24_P386746	0.006	0.036	-8.573	-3.1
SLC44A2	Homo sapiens solute carrier family 44, member 2 (SLC44A2), transcript variant 1, mRNA [NM_020428]	A_24_P10657	0.00603	0.0361	-2.014	-1.01
AX746535	Homo sapiens cDNA FLJ33297 fis, clone BNGH42001406. [AK090616]	A_33_P3544856	0.00606	0.0362	-8.541	-3.094
HSD3B7	Homo sapiens hydroxy-delta-5-steroid dehydrogenase, 3 beta- and steroid delta-isomerase 7 (HSD3B7), transcript variant 2, mRNA [NM_001142777]	A_33_P3251776	0.00609	0.0363	-2.203	-1.14
HTR1F	Homo sapiens 5-hydroxytryptamine (serotonin) receptor 1F (HTR1F), mRNA [NM_000866]	A_33_P3406836	0.00609	0.0363	-2.256	-1.174
COX7A1	Homo sapiens cytochrome c oxidase subunit VIIa polypeptide 1 (muscle) (COX7A1), nuclear gene encoding mitochondrial protein, mRNA [NM_001864]	A_23_P67661	0.00609	0.0363	-5.5	-2.459
AK097991	Homo sapiens cDNA FLJ40672 fis, clone THYMU2021509. [AK097991]	A_33_P3294801	0.0061	0.0364	-2.52	-1.333
ENST000003398	serine/threonine kinase 3 [Source:HGNC Symbol;Acc:11406] [ENST00000424861]	A_33_P3231363	0.00611	0.0364	-2.542	-1.346
KCTD17	Homo sapiens potassium channel tetramerisation domain containing 17 (KCTD17), mRNA [NM_024681]	A_24_P108779	0.0061	0.0364	-2.316	-1.212
EPR1	Human effector cell protease receptor-1 (EPR-1) mRNA, partial cds. [L26245]	A_24_P923381	0.00614	0.0365	-2.423	-1.277
ZNF550	Homo sapiens zinc finger protein 550 (ZNF550), mRNA [NM_001039654]	A_33_P3299386	0.00614	0.0365	-2.074	-1.053
NRXN2	Homo sapiens neurexin 2 (NRXN2), transcript variant alpha-2, mRNA [NM_138732]	A_24_P261470	0.00615	0.0365	-3.521	-1.816
ENST000003166	GB	A_33_P3361157	0.00616	0.0366	-2.963	-1.567
MARCH2	Homo sapiens membrane-associated ring finger (C3HC4) 2 (MARCH2), transcript variant 1, mRNA [NM_016496]	A_23_P33683	0.00617	0.0366	-2.003	-1.002
ITGA2B	Homo sapiens integrin, alpha 2b (platelet glycoprotein IIb of IIb/IIIa complex, antigen CD41) (ITGA2B), mRNA [NM_000419]	A_24_P65373	0.0062	0.0367	-2.879	-1.526
IFRD2	Homo sapiens interferon-related developmental regulator 2 (IFRD2), mRNA [NM_006764]	A_23_P92132	0.00619	0.0367	-2.401	-1.264

SNRNP40	Homo sapiens small nuclear ribonucleoprotein 40kDa (U5) (SNRNP40), mRNA [NM_004814]	A_23_P45913	0.00623	0.0368	-2.103	-1.072
LOC100131355	Homo sapiens cDNA FLJ42223 fis, clone THYMU2039989. [AK124217]	A_33_P3254751	0.00622	0.0368	-3.17	-1.664
A_19_P00321562	Homo sapiens chromosome 7 open reading frame 71 (C7orf71), mRNA [NM_001145531]	A_19_P00321562	0.00624	0.0368	-4.773	-2.255
C7orf71	Homo sapiens chromosome 7 open reading frame 71 (C7orf71), mRNA [NM_001145531]	A_33_P3401003	0.00624	0.0368	-3.015	-1.592
ENST000003765	proline rich 3 [Source:HGNC Symbol;Acc:21149] [ENST00000461523]	A_33_P3257170	0.00627	0.0369	-2.19	-1.131
TFDP1	Homo sapiens transcription factor Dp-1 (TFDP1), transcript variant 1, mRNA [NM_007111]	A_32_P199301	0.0063	0.0371	-2.553	-1.352
BRPF3	Homo sapiens bromodomain and PHD finger containing, 3 (BRPF3), mRNA [NM_015695]	A_33_P3375077	0.0063	0.0371	-3.119	-1.641
PGM2L1	Homo sapiens phosphoglucomutase 2-like 1 (PGM2L1), mRNA [NM_173582]	A_33_P3363260	0.00638	0.0373	-2.122	-1.085
MGC12488	Homo sapiens, clone IMAGE:3932794, mRNA. [BC005372]	A_33_P3526315	0.00638	0.0373	-2.138	-1.096
IGANRP	PREDICTED: Homo sapiens hypothetical protein LOC100652933 (LOC100652933), mRNA [XM_003403523]	A_21_P0014026	0.00638	0.0373	-4.274	-2.096
ARHGAP28	Homo sapiens Rho GTPase activating protein 28 (ARHGAP28), mRNA [NM_001010000]	A_23_P130359	0.00637	0.0373	-2.081	-1.058
FKBP10	Homo sapiens FK506 binding protein 10, 65 kDa (FKBP10), mRNA [NM_021939]	A_23_P15727	0.0064	0.0374	-6.673	-2.738
FAM126A	Homo sapiens family with sequence similarity 126, member A (FAM126A), mRNA [NM_032581]	A_23_P8582	0.0064	0.0374	-2.255	-1.173
LRRC4B	Homo sapiens leucine rich repeat containing 4B (LRRC4B), mRNA [NM_001080457]	A_23_P419503	0.00641	0.0374	-11.649	-3.542
DB511925	DB511925 RIKEN full-length enriched human cDNA library, testis Homo sapiens cDNA clone H013027K15 3', mRNA sequence [DB511925]	A_33_P3366733	0.00639	0.0374	-2.465	-1.301
RBM15	Homo sapiens RNA binding motif protein 15 (RBM15), transcript variant 1, mRNA [NM_022768]	A_23_P34877	0.00646	0.0376	-2.496	-1.32
HAGHL	Homo sapiens hydroxyacylglutathione hydrolase-like (HAGHL), transcript variant 2, mRNA [NM_032304]	A_24_P356373	0.00648	0.0377	-2.423	-1.277
TSKU	Homo sapiens tsukushi small leucine rich proteoglycan homolog (Xenopus laevis) (TSKU), mRNA [NM_015516]	A_23_P162142	0.00648	0.0377	-2.987	-1.579
HOXC5	Homo sapiens homeobox C5 (HOXC5), transcript variant 1, mRNA [NM_018953]	A_23_P162355	0.00652	0.0378	-2.098	-1.069
SLC1A5	Homo sapiens solute carrier family 1 (neutral amino acid transporter), member 5 (SLC1A5), transcript variant 1, mRNA [NM_005628]	A_23_P55998	0.00653	0.0378	-2.943	-1.557
A_33_P3248077	Unknown	A_33_P3248077	0.00653	0.0378	-2.976	-1.573
DHRS4L1	Homo sapiens dehydrogenase/reductase (SDR family) member 4 like 1 (DHRS4L1), mRNA [NM_001082488]	A_33_P3359368	0.00652	0.0378	-2.189	-1.13
SLC39A2	Homo sapiens solute carrier family 39 (zinc transporter), member 2 (SLC39A2), transcript variant 1, mRNA [NM_014579]	A_23_P128855	0.00656	0.0379	-2.98	-1.575
THC2570832	general transcription factor IIi [Source:HGNC Symbol;Acc:4659] [ENST00000473333]	A_33_P3421183	0.00656	0.0379	-2.231	-1.158
AK1	Homo sapiens adenylate kinase 1 (AK1), mRNA [NM_000476]	A_23_P217088	0.00654	0.0379	-2.941	-1.556
LARP2	Homo sapiens La ribonucleoprotein domain family, member 1B (LARP1B), transcript variant 1, mRNA [NM_018078]	A_23_P7253	0.00657	0.038	-2.11	-1.077

HEPACAM2	Homo sapiens HEPACAM family member 2 (HEPACAM2), transcript variant 1, mRNA [NM_001039372]	A_32_P118397	0.00658	0.038	-17.243	-4.108
GATA2	Homo sapiens GATA binding protein 2 (GATA2), transcript variant 1, mRNA [NM_001145661]	A_33_P3550894	0.00662	0.0381	-7.927	-2.987
LOC100509323	PREDICTED: Homo sapiens baculoviral IAP repeat-containing protein 1-like (LOC100509323), mRNA [XM_003119950]	A_21_P0013988	0.00661	0.0381	-2.319	-1.214
SYNGAP1	Homo sapiens synaptic Ras GTPase activating protein 1 (SYNGAP1), mRNA [NM_006772]	A_24_P9346	0.00666	0.0383	-2.515	-1.331
S100A7	Homo sapiens S100 calcium binding protein A7 (S100A7), mRNA [NM_002963]	A_23_P103310	0.00666	0.0383	-2.644	-1.403
CASC5	Homo sapiens cancer susceptibility candidate 5 (CASC5), transcript variant 1, mRNA [NM_170589]	A_23_P100127	0.00665	0.0383	-2.33	-1.221
SLC25A39	Homo sapiens solute carrier family 25, member 39 (SLC25A39), transcript variant 2, mRNA [NM_016016]	A_23_P100764	0.00665	0.0383	-2.628	-1.394
GRIN2C	Homo sapiens glutamate receptor, ionotropic, N-methyl D-aspartate 2C (GRIN2C), mRNA [NM_000835]	A_23_P49546	0.00666	0.0383	-4.021	-2.007
RAB15	Homo sapiens RAB15, member RAS onocogene family (RAB15), mRNA [NM_198686]	A_24_P193295	0.0067	0.0384	-3.147	-1.654
FBXO11	Homo sapiens F-box protein 11 (FBXO11), transcript variant 4, mRNA [NM_001190274]	A_23_P356101	0.00672	0.0385	-2.014	-1.01
CDK8	Homo sapiens cyclin-dependent kinase 8 (CDK8), mRNA [NM_001260]	A_33_P3264780	0.00676	0.0387	-2.162	-1.112
GANC	Homo sapiens glucosidase, alpha; neutral C (GANC), mRNA [NM_198141]	A_33_P3295108	0.00678	0.0387	-2.109	-1.077
HSN2	Homo sapiens WNK lysine deficient protein kinase 1 (WNK1), transcript variant 3, mRNA [NM_213655]	A_33_P3510335	0.00678	0.0387	-2.405	-1.266
MAPKSP1	Homo sapiens late endosomal/lysosomal adaptor, MAPK and MTOR activator 3 (LAMTOR3), transcript variant 1, mRNA [NM_021970]	A_33_P3251480	0.00677	0.0387	-2.497	-1.32
LOC645726	HSU09087 thymopoietin beta {Homo sapiens} (exp=-1; wgp=0; cg=0), partial (28%) [THC2520108]	A_32_P204722	0.0068	0.0388	-2.453	-1.294
FAM111B	Homo sapiens family with sequence similarity 111, member B (FAM111B), transcript variant 1, mRNA [NM_198947]	A_33_P3270657	0.00681	0.0388	-3.938	-1.977
EMP3	Homo sapiens epithelial membrane protein 3 (EMP3), mRNA [NM_001425]	A_23_P119362	0.00689	0.0391	-2.107	-1.075
C15orf52	Homo sapiens chromosome 15 open reading frame 52 (C15orf52), mRNA [NM_207380]	A_23_P163467	0.00691	0.0392	-3.362	-1.749
GPT2	Homo sapiens glutamic pyruvate transaminase (alanine aminotransferase) 2 (GPT2), transcript variant 1, mRNA [NM_133443]	A_23_P37892	0.00692	0.0392	-3.577	-1.839
E2F6	Homo sapiens E2F transcription factor 6 (E2F6), mRNA [NM_198256]	A_33_P3416301	0.00691	0.0392	-2.526	-1.337
SLC6A9	Homo sapiens solute carrier family 6 (neurotransmitter transporter, glycine), member 9 (SLC6A9), transcript variant 2, mRNA [NM_201649]	A_33_P3402615	0.0069	0.0392	-7.846	-2.972
C19orf61	Homo sapiens smg-9 homolog, nonsense mediated mRNA decay factor (C. elegans) (SMG9), mRNA [NM_019108]	A_23_P56127	0.00698	0.0394	-2.21	-1.144
ALDOC	Homo sapiens aldolase C, fructose-bisphosphate (ALDOC), mRNA [NM_005165]	A_23_P78108	0.00699	0.0395	-4.082	-2.029
LRP8	Homo sapiens low density lipoprotein receptor-related protein 8, apolipoprotein e receptor (LRP8), transcript variant 2, mRNA [NM_033300]	A_23_P200222	0.00701	0.0395	-4.028	-2.01
PSPC1	paraspeckle component 1 [Source:HGNC Symbol;Acc:20320] [ENST00000338910]	A_33_P3232173	0.007	0.0395	-2.61	-1.384

MCM9	Homo sapiens minichromosome maintenance complex component 9 (MCM9), transcript variant 1, mRNA [NM_017696]	A_33_P3254555	0.00705	0.0396	-1.93	-0.949
A_33_P3365845	nucleoporin like 1 [Source:HGNC Symbol;Acc:20261] [ENST00000466694]	A_33_P3365845	0.00703	0.0396	-2.778	-1.474
SORD	Homo sapiens sorbitol dehydrogenase (SORD), transcript variant 1, mRNA [NM_003104]	A_23_P77103	0.00704	0.0396	-2.822	-1.496
NT5M	Homo sapiens 5',3'-nucleotidase, mitochondrial (NT5M), nuclear gene encoding mitochondrial protein, mRNA [NM_020201]	A_33_P3242863	0.00706	0.0397	-2.526	-1.337
ING5	Homo sapiens inhibitor of growth family, member 5 (ING5), mRNA [NM_032329]	A_24_P34545	0.00706	0.0397	-2.026	-1.019
TAL1	Homo sapiens T-cell acute lymphocytic leukemia 1 (TAL1), mRNA [NM_003189]	A_23_P63371	0.0071	0.0398	-2.609	-1.383
HIST2H2BE	Homo sapiens histone cluster 2, H2be (HIST2H2BE), mRNA [NM_003528]	A_33_P3229246	0.00711	0.0398	-2.38	-1.251
LIMS2	Homo sapiens LIM and senescent cell antigen-like domains 2 (LIMS2), transcript variant 5, mRNA [NM_001161404]	A_33_P3268304	0.00717	0.04	-2.435	-1.284
GDAP1	Homo sapiens ganglioside-induced differentiation-associated protein 1 (GDAP1), transcript variant 1, mRNA [NM_018972]	A_33_P3422659	0.00715	0.04	-3.368	-1.752
TRPV6	Homo sapiens transient receptor potential cation channel, subfamily V, member 6 (TRPV6), mRNA [NM_018646]	A_33_P3364854	0.00719	0.0401	-4.499	-2.17
PLEKHM1	Homo sapiens pleckstrin homology domain containing, family M (with RUN domain) member 1 (PLEKHM1), transcript variant 1, mRNA [NM_014798]	A_33_P3405897	0.00721	0.0401	-2.298	-1.2
CGB2	Homo sapiens chorionic gonadotropin, beta polypeptide 2 (CGB2), mRNA [NM_033378]	A_23_P27709	0.00722	0.0402	-2.464	-1.301
PDLIM2	Homo sapiens PDZ and LIM domain 2 (mystique) (PDLIM2), transcript variant 3, mRNA [NM_198042]	A_33_P3311371	0.00722	0.0402	-2.754	-1.461
ZNF525	Homo sapiens zinc finger protein 525 (ZNF525), non-coding RNA [NR_003699]	A_33_P3273285	0.00731	0.0405	-2.178	-1.123
FLJ39632	human full-length cDNA 5-PRIME end of clone CS0DK007YB08 of HeLa cells of Homo sapiens (human). [BX248778]	A_33_P3237850	0.00738	0.0407	-2.212	-1.145
A_33_P3387786	Unknown	A_33_P3387786	0.00742	0.0409	-2.496	-1.319
NSF	Homo sapiens N-ethylmaleimide-sensitive factor (NSF), transcript variant 1, mRNA [NM_006178]	A_33_P3284108	0.00742	0.0409	-2.614	-1.386
KDM4C	Homo sapiens lysine (K)-specific demethylase 4C (KDM4C), transcript variant 3, mRNA [NM_001146695]	A_21_P0000019	0.00746	0.041	-2.212	-1.145
CRB2	Homo sapiens crumbs homolog 2 (Drosophila) (CRB2), mRNA [NM_173689]	A_24_P326398	0.0075	0.0411	-2.69	-1.428
SLC30A10	Homo sapiens solute carrier family 30, member 10 (SLC30A10), mRNA [NM_018713]	A_33_P3269885	0.00749	0.0411	-6.445	-2.688
HIST1H4D	Homo sapiens histone cluster 1, H4d (HIST1H4D), mRNA [NM_003539]	A_33_P3410836	0.00758	0.0414	-2.12	-1.084
LOC100128994	Unknown	A_33_P3318763	0.00761	0.0415	-2.475	-1.308
USP5	Homo sapiens ubiquitin specific peptidase 5 (isopeptidase T) (USP5), transcript variant 2, mRNA [NM_003481]	A_23_P64954	0.00764	0.0416	-2.141	-1.098
LOC100288271	Unknown	A_33_P3391756	0.00764	0.0416	-3.221	-1.688
AMBRA1	Homo sapiens autophagy/beclin-1 regulator 1 (AMBRA1), mRNA [NM_017749]	A_33_P3293169	0.00763	0.0416	-2.058	-1.041
BCL2L1	Homo sapiens BCL2-like 1 (BCL2L1), nuclear gene encoding mitochondrial protein, transcript variant 1, mRNA [NM_138578]	A_23_P210886	0.00763	0.0416	-2.768	-1.469

KIFC3	Homo sapiens kinesin family member C3 (KIFC3), transcript variant 1, mRNA [NM_005550]	A_23_P54576	0.00766	0.0417	-2.16	-1.111
CUL9	cullin 9 [Source:HGNC Symbol;Acc:15982] [ENST00000451399]	A_33_P3305058	0.00768	0.0418	-2.898	-1.535
ZNF134	Homo sapiens zinc finger protein 134 (ZNF134), mRNA [NM_003435]	A_33_P3259662	0.00769	0.0418	-2.187	-1.129
FECH	Homo sapiens ferrochelatase (FECH), nuclear gene encoding mitochondrial protein, transcript variant 1, mRNA [NM_001012515]	A_33_P3214635	0.00773	0.042	-2.413	-1.271
ENST000004015	Unknown	A_24_P143653	0.00776	0.0421	-2.007	-1.005
TNXB	Homo sapiens tenascin XB (TNXB), transcript variant XB, mRNA [NM_019105]	A_33_P3381338	0.00777	0.0421	-3.171	-1.665
CTNND1	Homo sapiens catenin (cadherin-associated protein), delta 1 (CTNND1), transcript variant 16, mRNA [NM_001206885]	A_33_P3209716	0.0078	0.0422	-2.147	-1.102
SLC6A4	Homo sapiens solute carrier family 6 (neurotransmitter transporter, serotonin), member 4 (SLC6A4), mRNA [NM_001045]	A_23_P152995	0.00783	0.0423	-3.446	-1.785
ENST000003246	chromosome 20 open reading frame 108 [Source:HGNC Symbol;Acc:16102] [ENST00000437418]	A_33_P3306352	0.00783	0.0423	-2.79	-1.48
KRT18P28	Unknown	A_24_P169843	0.00785	0.0424	-2.225	-1.154
AK123980	aarF domain containing kinase 3 [Source:HGNC Symbol;Acc:16812] [ENST00000366779]	A_33_P3372924	0.00785	0.0424	-2.318	-1.213
CALML4	Homo sapiens calmodulin-like 4 (CALML4), transcript variant 1, mRNA [NM_033429]	A_23_P129188	0.00789	0.0425	-2.648	-1.405
MAP1A	Homo sapiens microtubule-associated protein 1A (MAP1A), mRNA [NM_002373]	A_23_P163455	0.0079	0.0426	-6.674	-2.739
SHF	Homo sapiens Src homology 2 domain containing F (SHF), mRNA [NM_138356]	A_32_P183904	0.00793	0.0426	-2.791	-1.481
LOC100131326	fatty acid desaturase 2 [Source:HGNC Symbol;Acc:3575] [ENST00000517839]	A_33_P3360326	0.00795	0.0427	-2.775	-1.473
PTMA	Homo sapiens prothymosin, alpha (PTMA), transcript variant 1, mRNA [NM_001099285]	A_33_P3346403	0.008	0.0429	-2.471	-1.305
SC65	Homo sapiens leprecan-like 4 (LEPREL4), mRNA [NM_006455]	A_33_P3257030	0.00801	0.0429	-4.361	-2.125
PRF1	Homo sapiens perforin 1 (pore forming protein) (PRF1), transcript variant 1, mRNA [NM_005041]	A_23_P1473	0.00802	0.0429	-4.15	-2.053
A_19_P0032056	ALU2_HUMAN (P39189) Alu subfamily SB sequence contamination warning entry, partial (8%) [THC2492568]	A_19_P00320562	0.00803	0.043	-2.764	-1.467
GPR135	Homo sapiens G protein-coupled receptor 135 (GPR135), mRNA [NM_022571]	A_23_P205575	0.00804	0.043	-2.104	-1.073
FAM81B	Homo sapiens family with sequence similarity 81, member B (FAM81B), mRNA [NM_152548]	A_23_P360354	0.00808	0.0431	-4.509	-2.173
EFCAB3	Homo sapiens EF-hand calcium binding domain 3 (EFCAB3), transcript variant 1, mRNA [NM_001144933]	A_33_P3264042	0.00807	0.0431	-2.615	-1.387
MYL4	Homo sapiens myosin, light chain 4, alkali; atrial, embryonic (MYL4), transcript variant 2, mRNA [NM_002476]	A_24_P151032	0.00811	0.0432	-8.952	-3.162
TMEM173	Homo sapiens transmembrane protein 173 (TMEM173), nuclear gene encoding mitochondrial protein, mRNA [NM_198282]	A_23_P61371	0.00811	0.0432	-2.698	-1.432
LOC100130764	Unknown	A_33_P3323544	0.00812	0.0433	-2.896	-1.534
MON1A	Homo sapiens MON1 homolog A (yeast) (MON1A), transcript variant 1, mRNA [NM_032355]	A_23_P41075	0.00812	0.0433	-2.024	-1.017

RHCE	Homo sapiens Rh blood group, CcEe antigens (RHCE), transcript variant 1, mRNA [NM_020485]	A_23_P62634	0.00816	0.0434	-3.65	-1.868
AK309941	arginine and glutamate rich 1 [Source:HGNC Symbol;Acc:25482] [ENST00000426600]	A_33_P3343101	0.00816	0.0434	-2.289	-1.195
PLK5P	Homo sapiens polo-like kinase 5 (PLK5), mRNA [NM_001243079]	A_32_P50223	0.00821	0.0436	-2.359	-1.238
Z39353	HSC18A052 normalized infant brain cDNA Homo sapiens cDNA clone c-18a05 3', mRNA sequence [Z39353]	A_33_P3473108	0.0082	0.0436	-2.447	-1.291
TNS1	Homo sapiens tensin 1 (TNS1), mRNA [NM_022648]	A_33_P3209491	0.0082	0.0436	-2.212	-1.145
C17orf96	Homo sapiens chromosome 17 open reading frame 96 (C17orf96), mRNA [NM_001130677]	A_33_P3393766	0.00823	0.0436	-2.541	-1.346
C9orf97	Homo sapiens thiosulfate sulfurtransferase (rhodanese)-like domain containing 2 (TSTD2), mRNA [NM_139246]	A_24_P295330	0.0083	0.0439	-5.126	-2.358
PA2G4	Homo sapiens proliferation-associated 2G4, 38kDa (PA2G4), mRNA [NM_006191]	A_33_P3227209	0.00832	0.0439	-2.032	-1.023
FOLR1	Homo sapiens folate receptor 1 (adult) (FOLR1), transcript variant 1, mRNA [NM_016725]	A_23_P53176	0.00832	0.0439	-2.874	-1.523
PRDM2	Homo sapiens PR domain containing 2, with ZNF domain (PRDM2), transcript variant 2, mRNA [NM_015866]	A_33_P3342076	0.00833	0.044	-2.093	-1.066
ZNF740	Homo sapiens zinc finger protein 740 (ZNF740), mRNA [NM_001004304]	A_33_P3334108	0.00837	0.0441	-2.077	-1.055
A_21_P0014035	PREDICTED: Homo sapiens hypothetical protein LOC100653234 (LOC100653234), mRNA [XM_003403644]	A_21_P0014035	0.00836	0.0441	-3.92	-1.971
TYRO3	Homo sapiens TYRO3 protein tyrosine kinase (TYRO3), mRNA [NM_006293]	A_23_P54517	0.00837	0.0441	-2.652	-1.407
AB527908	cysteine-rich protein 1 (intestinal) [Source:HGNC Symbol;Acc:2360] [ENST00000330233]	A_33_P3354965	0.00842	0.0442	-2.146	-1.101
LAMB3	Homo sapiens laminin, beta 3 (LAMB3), transcript variant 2, mRNA [NM_001017402]	A_33_P3338121	0.00842	0.0442	-3.054	-1.61
CD163L1	Homo sapiens CD163 molecule-like 1 (CD163L1), mRNA [NM_174941]	A_23_P61466	0.0084	0.0442	-2.368	-1.244
NELF	Homo sapiens nasal embryonic LHRH factor (NELF), transcript variant 2, mRNA [NM_015537]	A_23_P32064	0.00846	0.0443	-2.587	-1.371
C10orf128	chromosome 10 open reading frame 128 [Source:HGNC Symbol;Acc:27274] [ENST00000374148]	A_33_P3341906	0.00844	0.0443	-4.54	-2.183
CD84	Homo sapiens CD84 molecule (CD84), transcript variant 2, mRNA [NM_003874]	A_24_P362193	0.00843	0.0443	-3.842	-1.942
FEV	Homo sapiens FEV (ETS oncogene family) (FEV), mRNA [NM_017521]	A_23_P5460	0.00848	0.0444	-3.085	-1.625
IZUMO1	Homo sapiens izumo sperm-egg fusion 1 (IZUMO1), mRNA [NM_182575]	A_24_P340247	0.00848	0.0444	-3.395	-1.763
RNF217	Homo sapiens ring finger protein 217 (RNF217), mRNA [NM_152553]	A_23_P353005	0.00847	0.0444	-2.181	-1.125
PSMG4	Homo sapiens proteasome (prosome, macropain) assembly chaperone 4 (PSMG4), transcript variant 3, mRNA [NM_001135750]	A_33_P3259053	0.00847	0.0444	-2.367	-1.243
NLE1	Homo sapiens notchless homolog 1 (Drosophila) (NLE1), transcript variant 2, mRNA [NM_001014445]	A_23_P141315	0.00851	0.0445	-2.234	-1.16
TMEM59L	Homo sapiens transmembrane protein 59-like (TMEM59L), mRNA [NM_012109]	A_33_P3302245	0.00855	0.0446	-2.282	-1.19
A_19_P0031705[illegible]	Homo sapiens ring finger protein 213 (RNF213), transcript variant 1, mRNA [NM_020914]	A_19_P00317052	0.00854	0.0446	-2.534	-1.341
DISC1	Homo sapiens disrupted in schizophrenia 1 (DISC1), transcript variant q, mRNA [NM_001164554]	A_21_P0000052	0.00854	0.0446	-2.742	-1.455

CDT1	Homo sapiens chromatin licensing and DNA replication factor 1 (CDT1), mRNA [NM_030928]	A_33_P3386262	0.00863	0.0449	-2.005	-1.004
LOC100128055	SMCA5_HUMAN (O60264) SWI/SNF-related matrix-associated actin-dependent regulator of chromatin subfamily A member 5 (SWI/SNF-related matrix-associated actin-dependent regulator of chromatin A5) (Sucrose nonfermenting protein 2 homolog) (hSNF2 , partial (5%) [THC2670760]	A_33_P3288589	0.00866	0.045	-3.608	-1.851
BCL6B	Homo sapiens B-cell CLL/lymphoma 6, member B (BCL6B), mRNA [NM_181844]	A_23_P324813	0.00876	0.0453	-4.41	-2.141
HIST1H4A	Homo sapiens histone cluster 1, H4a (HIST1H4A), mRNA [NM_003538]	A_33_P3344229	0.00876	0.0453	-2.23	-1.157
DA858408	DA858408 PLACE7 Homo sapiens cDNA clone PLACE7009954 5', mRNA sequence [DA858408]	A_33_P3209200	0.0088	0.0454	-2.907	-1.54
PTS	Homo sapiens 6-pyruvoyltetrahydropterin synthase (PTS), mRNA [NM_000317]	A_23_P127579	0.00888	0.0457	-2.28	-1.189
CAB39	Homo sapiens calcium binding protein 39 (CAB39), transcript variant 2, mRNA [NM_001130849]	A_33_P3316293	0.00887	0.0457	-2.591	-1.373
DKFZp686M1136	Homo sapiens mRNA; cDNA DKFZp686M1136 (from clone DKFZp686M1136). [BX647230]	A_33_P3736195	0.00887	0.0457	-4.354	-2.122
TTYH2	Homo sapiens tweety homolog 2 (Drosophila) (TTYH2), transcript variant 1, mRNA [NM_032646]	A_23_P66432	0.0089	0.0457	-4.693	-2.23
ALOX12	Homo sapiens arachidonate 12-lipoxygenase (ALOX12), mRNA [NM_000697]	A_23_P152906	0.00891	0.0458	-6.308	-2.657
ENST000003915	Homo sapiens mRNA for FLJ00363 protein. [AK090442]	A_24_P298737	0.00898	0.046	-2.538	-1.344
KDELC2	Homo sapiens KDEL (Lys-Asp-Glu-Leu) containing 2 (KDELC2), mRNA [NM_153705]	A_33_P3214849	0.00898	0.046	-1.811	-0.857
NAA16	Homo sapiens N(alpha)-acetyltransferase 16, NatA auxiliary subunit (NAA16), transcript variant 1, mRNA [NM_024561]	A_33_P3361811	0.00903	0.0462	-2.621	-1.39
ENST000003957	retinoic acid induced 1 [Source:HGNC Symbol;Acc:9834] [ENST00000395776]	A_33_P3320493	0.00912	0.0465	-3.143	-1.652
LOC100287092	Unknown	A_33_P3402495	0.00912	0.0465	-3.652	-1.869
DNMT3B	Homo sapiens DNA (cytosine-5-)-methyltransferase 3 beta (DNMT3B), transcript variant 6, mRNA [NM_175850]	A_23_P28953	0.00915	0.0466	-2.017	-1.012
PPP2R1B	Homo sapiens protein phosphatase 2, regulatory subunit A, beta (PPP2R1B), transcript variant 1, mRNA [NM_002716]	A_24_P98762	0.00915	0.0466	-2.194	-1.133
ZNF584	zinc finger protein 584 [Source:HGNC Symbol;Acc:27318] [ENST00000322834]	A_33_P3228642	0.00917	0.0467	-2.458	-1.298
HBQ1	Homo sapiens hemoglobin, theta 1 (HBQ1), mRNA [NM_005331]	A_23_P49254	0.00923	0.0469	-4.976	-2.315
SELI	Homo sapiens ethanolaminephosphotransferase 1 (CDP-ethanolamine-specific) (EPT1), mRNA [NM_033505]	A_24_P940803	0.00931	0.0471	-2.397	-1.261
RTBDN	Homo sapiens retbindin (RTBDN), transcript variant 2, mRNA [NM_031429]	A_23_P130653	0.00931	0.0471	-2.209	-1.143
E2F2	Homo sapiens E2F transcription factor 2 (E2F2), mRNA [NM_004091]	A_23_P408955	0.0093	0.0471	-2.176	-1.122
ITGB1	Homo sapiens integrin, beta 1 (fibronectin receptor, beta polypeptide, antigen CD29 includes MDF2, MSK12) (ITGB1), transcript variant 1E, mRNA [NM_133376]	A_23_P104199	0.00942	0.0475	-2.39	-1.257
DCAF6	Homo sapiens DDB1 and CUL4 associated factor 6 (DCAF6), transcript variant 2, mRNA [NM_001017977]	A_33_P3294449	0.00945	0.0476	-2.939	-1.555

SLC4A9	Homo sapiens solute carrier family 4, sodium bicarbonate cotransporter, member 9 (SLC4A9), mRNA [NM_031467]	A_23_P257176	0.0095	0.0478	-2.336	-1.224
GPR32	Homo sapiens G protein-coupled receptor 32 (GPR32), mRNA [NM_001506]	A_23_P142096	0.00951	0.0478	-2.276	-1.186
SUPT16HP	Unknown	A_24_P255845	0.00958	0.048	-2.04	-1.029
KLF1	Homo sapiens Kruppel-like factor 1 (erythroid) (KLF1), mRNA [NM_006563]	A_33_P3410925	0.00958	0.048	-9.719	-3.281
ENTPD3	Homo sapiens ectonucleoside triphosphate diphosphohydrolase 3 (ENTPD3), mRNA [NM_001248]	A_23_P212469	0.00961	0.0481	-3.145	-1.653
C17orf99	Homo sapiens chromosome 17 open reading frame 99 (C17orf99), mRNA [NM_001163075]	A_33_P3384133	0.00963	0.0482	-9.908	-3.309
KIAA1432	Homo sapiens KIAA1432 (KIAA1432), transcript variant 1, mRNA [NM_020829]	A_33_P3358521	0.00965	0.0482	-2.039	-1.028
MMEL1	Homo sapiens membrane metallo-endopeptidase-like 1 (MMEL1), mRNA [NM_033467]	A_33_P3777207	0.00962	0.0482	-3.342	-1.741
NPTXR	Homo sapiens neuronal pentraxin receptor (NPTXR), mRNA [NM_014293]	A_23_P29282	0.00963	0.0482	-2.166	-1.115
LOC729983	Unknown	A_32_P152696	0.00966	0.0483	-2.463	-1.3
LINGO1	Homo sapiens leucine rich repeat and Ig domain containing 1 (LINGO1), mRNA [NM_032808]	A_24_P112941	0.00968	0.0483	-4.342	-2.118
LOC100128567	Homo sapiens cDNA FLJ27128 fis, clone SPL07659. [AK130638]	A_33_P3881056	0.00966	0.0483	-4.703	-2.233
TAS2R45	Homo sapiens taste receptor, type 2, member 45 (TAS2R45), mRNA [NM_176886]	A_23_P303851	0.00967	0.0483	-4.879	-2.286
RTEL1	Homo sapiens regulator of telomere elongation helicase 1 (RTEL1), transcript variant 1, mRNA [NM_016434]	A_23_P378526	0.00974	0.0485	-2.607	-1.382
SLC39A3	Homo sapiens solute carrier family 39 (zinc transporter), member 3 (SLC39A3), transcript variant 1, mRNA [NM_144564]	A_24_P382467	0.00977	0.0486	-3.456	-1.789
GPSM1	Homo sapiens G-protein signaling modulator 1 (GPSM1), transcript variant 2, mRNA [NM_015597]	A_33_P3293247	0.00978	0.0486	-2.74	-1.454
PAGE4	Homo sapiens P antigen family, member 4 (prostate associated) (PAGE4), mRNA [NM_007003]	A_24_P254506	0.00983	0.0488	-3.437	-1.781
ENST000003778	histone cluster 1, H4c [Source:HGNC Symbol;Acc:4787] [ENST00000377803]	A_33_P3378880	0.00987	0.0489	-2.471	-1.305
KHK	Homo sapiens ketohexokinase (fructokinase) (KHK), transcript variant a, mRNA [NM_000221]	A_33_P3309984	0.00986	0.0489	-2.694	-1.43
C22orf36	Homo sapiens chromosome 22 open reading frame 36 (C22orf36), mRNA [NM_207644]	A_32_P198325	0.00989	0.049	-2.287	-1.194
NOL12	Homo sapiens nucleolar protein 12 (NOL12), mRNA [NM_024313]	A_33_P3417820	0.00992	0.0491	-2.064	-1.045
A_33_P3345001	Unknown	A_33_P3345001	0.00993	0.0491	-2.112	-1.079
ACTL8	Homo sapiens actin-like 8 (ACTL8), mRNA [NM_030812]	A_32_P176018	0.0101	0.0496	-3.044	-1.606
ATP2A3	Homo sapiens ATPase, Ca++ transporting, ubiquitous (ATP2A3), transcript variant 5, mRNA [NM_174953]	A_23_P207632	0.0101	0.0496	-2.396	-1.261
C19orf45	Homo sapiens chromosome 19 open reading frame 45 (C19orf45), mRNA [NM_198534]	A_33_P3319886	0.0101	0.0496	-2.198	-1.136
PATE3	Homo sapiens prostate and testis expressed 3 (PATE3), mRNA [NM_001129883]	A_33_P3420904	0.0101	0.0496	-2.216	-1.148
SOD3	Homo sapiens superoxide dismutase 3, extracellular (SOD3), mRNA [NM_003102]	A_23_P254741	0.0101	0.0496	-10.444	-3.385

CAMKK1	Homo sapiens calcium/calmodulin-dependent protein kinase kinase 1, alpha (CAMKK1), transcript variant 1, mRNA [NM_032294]	A_23_P431933	0.0101	0.0496	-2.149	-1.104
STEAP3	Homo sapiens STEAP family member 3, metalloreductase (STEAP3), transcript variant 1, mRNA [NM_182915]	A_23_P90601	0.0102	0.0499	-3.31	-1.727
C1QL1	Homo sapiens complement component 1, q subcomponent-like 1 (C1QL1), mRNA [NM_006688]	A_23_P77993	0.0102	0.0499	-5.948	-2.572
