# Supplementary material for: CEBPA-regulated lncRNAs, new players in the study of acute myeloid leukemia
Source: J Hematol Oncol. 2014 Sep 25;7:69. doi: 10.1186/s13045-014-0069-1 (PMC4177583; doi:10.1186/s13045-014-0069-1)
Supplement: Additional file 6: Table S3. — Chromosomal coordinates and TCONS names of validated C/EBPα -up regulated (Lnc-CUs) and -down-regulated (Lnc-DCs) lncRNAs. [file 13045_2014_69_MOESM6_ESM.docx]

**Table S3**. Chromosomal coordinates and TCONS names of validated C/EBPα -up regulated (Lnc-CUs) and -down-regulated (Lnc-DCs) lncRNAs

| **LncRNA** | **Chromosomal location** | **TCONS** |
| --- | --- | --- |
| Lnc-CU1 | chr5:163875251-163894693 | TCONS_00011079 |
| Lnc-CU2 | chr13:97591376-97601835 | TCONS_l2_00007295 |
| Lnc-CU3 | chr16:53399372-53404882 | TCONS_I2_00010170 |
| Lnc-CU4 | chr1:25436949-25439611 | TCONS_00000489 |
| Lnc-CU5 | chr14:73927912-73930338 | TCONS_00022790 |
| Lnc-CU6 | chr1:184638176-184641354 | TCONS_00001757 |
| Lnc-CU7 | chr12:132302149-132303828 | TCONS_00021009 |
| Lnc-CU8 | chr2:102578558-102603782 | TCONS_l2_00013854 |
| Lnc-CU9 | chr7:138912216-138914980 | TCONS_l2_00026920 |
| Lnc-CU10 | chr15:68126648-68131217 | TCONS_00023281 |
| Lnc-CU11 | chr10:13457202-13465133 | TCONS_00017790 |
| Lnc-CU12 | chr5:90598846-90610200 | TCONS_00009724 |
| Lnc-CU13 | chr8:61878746-61940021 | TCONS_00014726 |
| Lnc-CU14 | chr6:113944401-113953599 | TCONS_00012257 |
| Lnc-CU15 | chr6:6891670-6899654 | TCONS_00011670 |
| Lnc-CU16 | chr14:73925556-73930341 | TCONS_00022787 |
| Lnc-CU17 | chr3:193551438-193555388 | TCONS_00006739 |
| Lnc-CU18 | chr3:187553660-187574106 | TCONS_00006729 |
| Lnc-CU19 | chr1:171584011-171593241 | TCONS_00001200 |
| Lnc-CU20 | chr4:124571422-124851561 | NR_027105.2 |
| Lnc-CU21 | chr6:80773211-80780323 | TCONS_l2_00024305 |
| Lnc-DC1 | chr7:123175306-123175887 | TCONS_00013572 |
| Lnc-DC2 | chr21:16133802-16135504 | TCONS_00029079 |
| Lnc-DC3 | chr21:21629065-21631133 | TCONS_00028879 |
| Lnc-DC4 | chr1:27852316-27857072 | TCONS_00000171 |
| Lnc-DC5 | chr21:16134080-16135509 | TCONS_00029080 |
| Lnc-DC6 | chr15:91382899-91396016 | TCONS_00023505 |
| Lnc-DC7 | chr4:31351906-31353347 | TCONS_00007503 |
| Lnc-DC8 | chr19:28927600-28957414 | TCONS_l2_00013005 |
