# Supplementary material for: CEBPA-regulated lncRNAs, new players in the study of acute myeloid leukemia
Source: J Hematol Oncol. 2014 Sep 25;7:69. doi: 10.1186/s13045-014-0069-1 (PMC4177583; doi:10.1186/s13045-014-0069-1)
Supplement: Additional file 7: Figure S3. — Overlap between lncRNAs identified in this study used previously generated ChIP data sets for CEBPB and CEBPD in K562 cells. [file 13045_2014_69_MOESM7_ESM.pptx]

## Slide 1
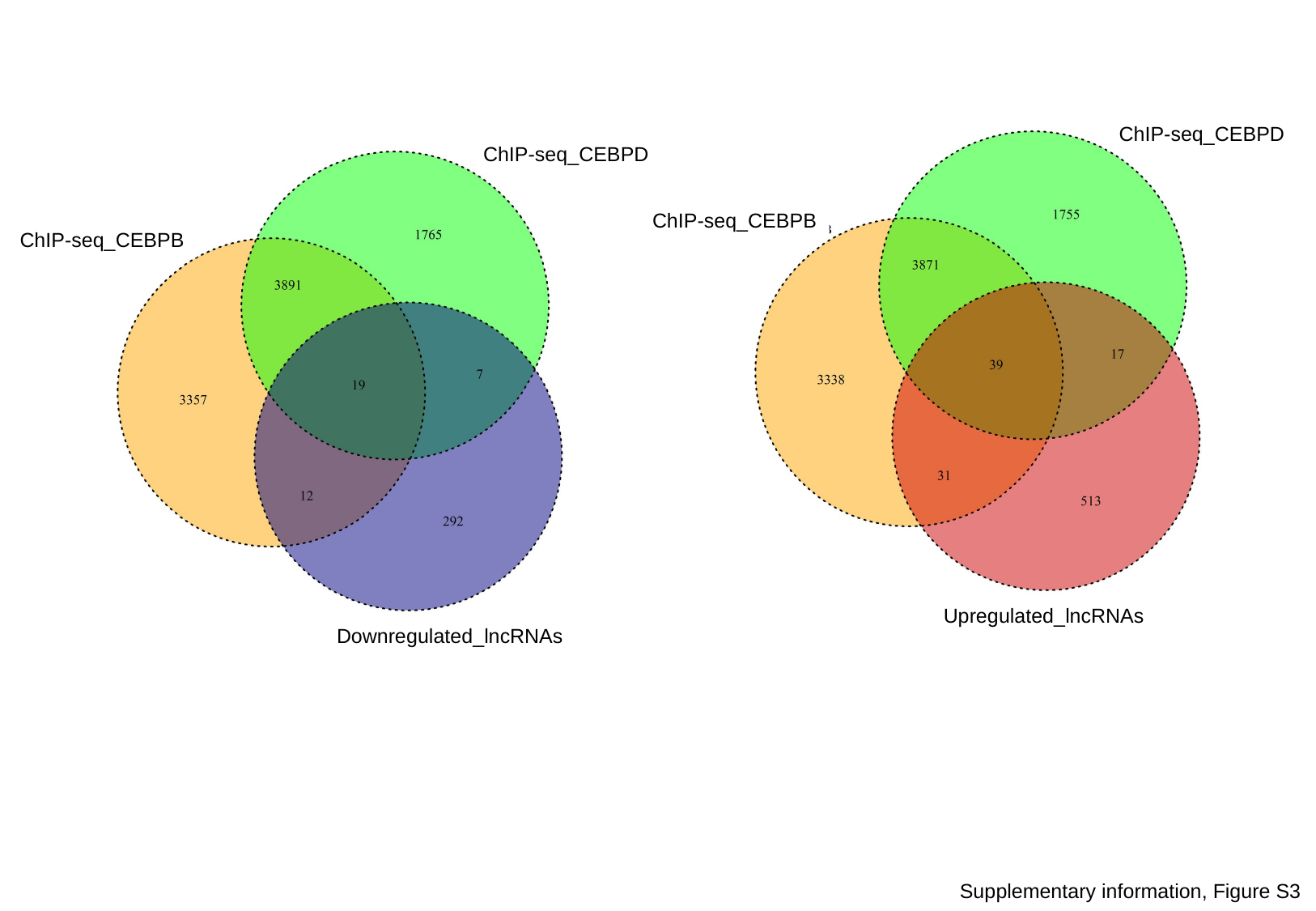

ChIP-seq_CEBPD
ChIP-seq_CEBPD
ChIP-seq_CEBPB
ChIP-seq_CEBPB
Upregulated_lncRNAs
Downregulated_lncRNAs
Supplementary information, Figure S3
